# Biosynthesis of saponin defensive compounds in sea cucumbers

**DOI:** 10.1038/s41589-022-01054-y

**Published:** 2022-06-27

**Authors:** Ramesha Thimmappa, Shi Wang, Minyan Zheng, Rajesh Chandra Misra, Ancheng C. Huang, Gerhard Saalbach, Yaqing Chang, Zunchun Zhou, Veronica Hinman, Zhenmin Bao, Anne Osbourn

**Affiliations:** 1 Department of Biochemistry and Metabolism, John Innes Centre, Norwich Research Park, Norwich, UK; 2 Sars-Fang Centre and MOE Key Laboratory of Marine Genetics and Breeding, Ocean University of China and National Laboratory for Marine Science and Technology, Qingdao, China; 3 Department of Biological Sciences, Carnegie Mellon University, Pittsburgh, PA USA; 4 College of Fisheries and Life Science, Dalian Ocean University, Dalian, China; 5 Liaoning Ocean and Fisheries Science Research Institute, Dalian, China; 6 Present Address: Amity Institute of Genome Engineering, Amity University Uttar Pradesh, Noida, India; 7 Present Address: Department of Biology, School of Life Sciences, Southern University of Science and Technology, Shenzhen, China

**Keywords:** Enzymes, Biosynthesis, Natural products, Chemical ecology

## Abstract

Soft-bodied slow-moving sea creatures such as sea stars and sea cucumbers lack an adaptive immune system and have instead evolved the ability to make specialized protective chemicals (glycosylated steroids and triterpenes) as part of their innate immune system. This raises the intriguing question of how these biosynthetic pathways have evolved. Sea star saponins are steroidal, while those of the sea cucumber are triterpenoid. Sterol biosynthesis in animals involves cyclization of 2,3-oxidosqualene to lanosterol by the oxidosqualene cyclase (OSC) enzyme lanosterol synthase (LSS). Here we show that sea cucumbers lack LSS and instead have two divergent OSCs that produce triterpene saponins and that are likely to have evolved from an ancestral LSS by gene duplication and neofunctionalization. We further show that sea cucumbers make alternate sterols that confer protection against self-poisoning by their own saponins. Collectively, these events have enabled sea cucumbers to evolve the ability to produce saponins and saponin-resistant sterols concomitantly.

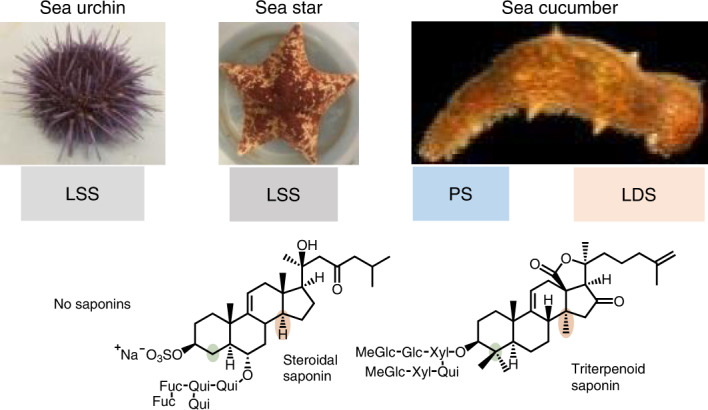

## Abstract (web summary)

Sea stars and sea cucumbers biosynthesize protective glycosylated steroids and triterpenes via divergent oxidosqualene cyclases (OSCs) that produce these distinct saponins in different species as well as in different tissues of a single species.

## Main

**Fig. 1:**
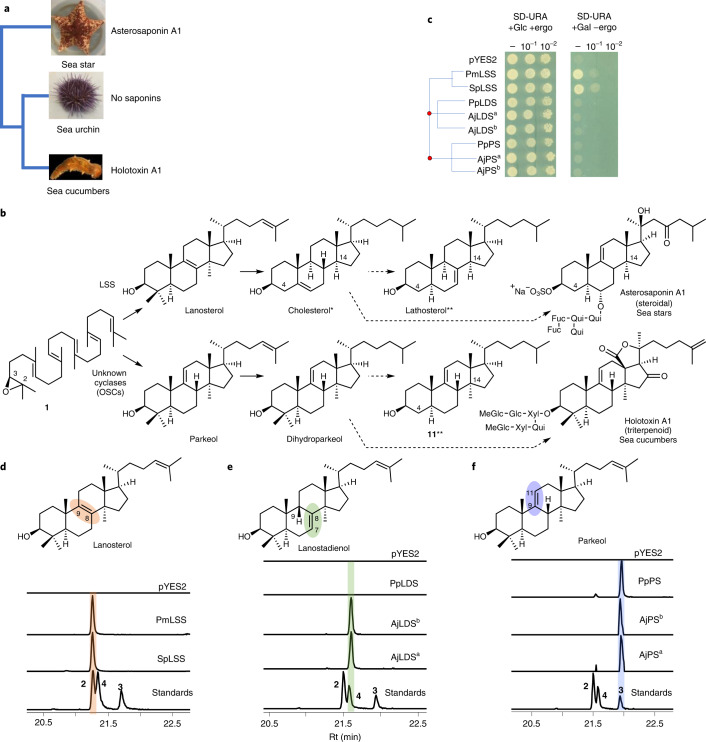
Evolution of divergent OSCs in sea cucumbers. **a**, Presence or absence of saponin chemical defenses in slow-moving, soft-bodied echinoderms. The tree is drawn as in ref. ^17^. **b**, Biosynthetic origin of steroidal and triterpene saponins and usual and unusual sterols in sea stars and sea cucumbers. *Usual sterol, characterized by a common C5 unsaturation as well as the absence of methyl groups at carbon positions 4 and 14. **Unusual sterols with C7 and C9(11) unsaturation. Solid and dashed arrows represent single and multiple steps, respectively. Glc, glucose. Fuc, fucose; MeGlc, methyl glucose; Xyl, xylose; Qui, quinovose. **c**, Complementation of the LSS-deficient yeast strain Gil77 with cloned OSC genes. pYES2, empty vector control. Yeast was spotted from stock cultures undiluted (−) and diluted tenfold and 100-fold. Ergo, ergosterol; Gal, galactose; SD-URA, synthetic defined medium without uracil. **d**–**f**, GC–MS profile of yeast extracts expressing clade I OSC candidates (**d**), clade II OSC1 (**e**) and OSC2 candidates (**f**). In the LDS experiments, ketoconazole (50 µg ml^−1^) was included in the medium to limit in vivo modifications of OSC1 products by the endogenous yeast CYP51 enzyme (Extended Data Fig. 2c and Methods). GC–MS peaks in **d**–**f** were extracted ion chromatograms for the ion *m*/*z* 426. The corresponding total ion chromatograms and mass spectra are shown in Extended Data Fig. 2b–h. The lower chromatograms in **d**–**f** show GC traces for an equimolar mixture of the standards lanosterol, lanostadienol and parkeol. Pm, sea star *P. miniata*; Sp, sea urchin *S. purpuratus*; Aj, sea cucumber *A. japonicus*; Pp, sea cucumber *P. parvimensis*. Superscripts ‘a’ and ‘b’ for *A. japonicus* LDS and *A. japonicus* PS denote different accessions of *A. japonicus*. Rt; retention time.

Echinoderms lack adaptive immunity and therefore rely exclusively on innate immunity for growth and survival in the hostile benthic environment^1^. As part of this innate immunity, soft-bodied slow-moving echinoderms such as sea cucumbers and sea stars produce specialized metabolites (glycosylated steroids and triterpenes, also known as saponins) that provide chemical defense against potential assailants^2,3^. By contrast, the closely related sea urchins are protected by spines and do not make such compounds^4^ (Fig. 1a). When stressed, as a first line of defense, some sea cucumbers expel sticky threads called Cuvierian tubules, which entangle their enemies and immobilize them^5,6^. Immobilized victims eventually die due to the saponins of these tubules^6^. In addition to defense, saponins also have other biological functions in sea creatures, including in reproduction, spawning and chemical communication with symbionts^7–9^. Many animals use toxins as chemical defenses. These protective compounds are usually either sequestered from food or produced by endosymbionts. By contrast, echinoderms biosynthesize their toxins themselves^10^. Saponins have antifungal activity that is attributed to their ability to form complexes with membrane sterols such as cholesterol (**5**), therefore causing membrane permeabilization and cell death. Sea stars and sea cucumbers make alternate, unusual sterols (lathosterol (**7**) and 14α-methylcholest-9(11)-en-3β-ol (**11**)) that protect against potential self-poisoning by endogenous saponins (Fig. 1b)^11^. Sea cucumbers are a food delicacy in South Asia, and their extracts (of which saponins are important bioactive components) are highly valued for their medicinal properties. For these reasons, sea cucumber cultivation is a multimillion-dollar industry^12^. Despite the biological and commercial importance of sea cucumber saponins, the biosynthetic pathways for these compounds and how they have evolved in marine animals are unknown.

Sea star saponins are steroidal (for example, asterosaponin A1 (**8**)), while those made by sea cucumbers are triterpenoid (for example, holotoxin A1 (**13**)), the major difference being the presence or absence of methyl groups at carbon positions C4 and C14 (Fig. 1b and Supplementary Fig. 1). Both types of compounds originate from the linear precursor 2,3-oxidosqualene (**1**)^13^ (Fig. 1b). In animals, the first committed step in sterol biosynthesis involves cyclization of **1** to lanosterol (**2**) by OSC enzymes known as LSSs (Fig. 1b). Lanosterol is subsequently converted to the essential sterol cholesterol, which also serves as a precursor for steroidal saponins in sea stars. Triterpenoid saponins are widespread in plants but rare in animals, sea cucumbers being a noteworthy exception. In plants, the OSC gene family has expanded and diversified to produce an array of diverse triterpene scaffolds, with an average of 10–15 OSC genes per diploid plant genome^13^. By contrast, animals normally have a single OSC gene that encodes the LSS required for the synthesis of essential sterols^13^. The enzymes for the biosynthesis of steroids and triterpenoids and the saponins derived from these scaffolds in marine animals are not known (Fig. 1b). Here we take a genome-mining approach to investigate the origins of steroids and triterpenoids in marine animals. We identify candidate OSC genes from sea stars, sea urchins and sea cucumbers, functionally characterize these by heterologous expression in yeast and determine their product specificities (for sterol precursors or triterpene scaffolds). We further investigate the likely biological roles of two divergent non-LSS OSCs in sea cucumbers. Our study provides insights into the emergence of triterpene biosynthesis in the sea cucumber lineage and the coevolution of this with the ability to produce unusual sterols that are saponin resistant and therefore are likely to provide protection against self-poisoning.

## Results

### Discovery and functional analysis of echinoderm OSCs

**Extended Data Fig. 1:**
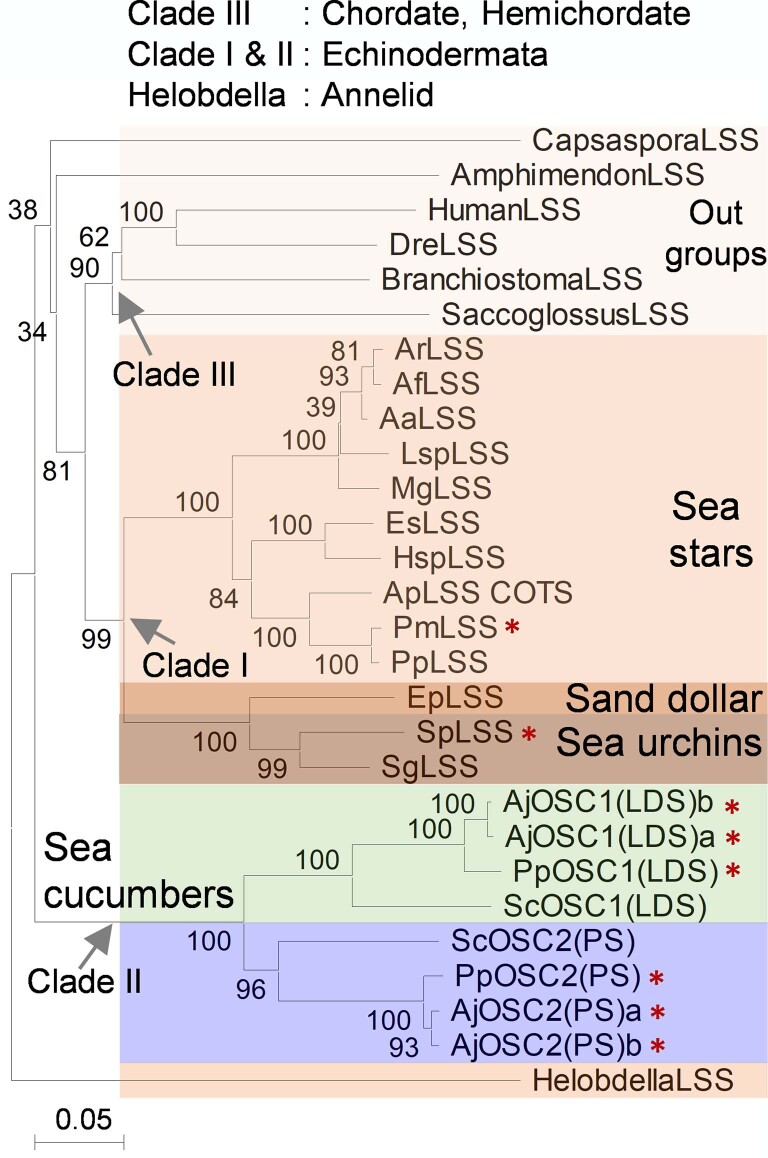
Phylogeny of predicted amino acid sequences of echinoderm OSCs. Major clades are colored. Candidates with red stars were functionally characterized in yeast in this study. Scale bar 0.1 amino acid substitutions per site. OSCs sequence details are provided in Supplementary Table 2.

A sea cucumber genome sequence has recently been released^14^. To investigate the occurrence and types of OSCs in echinoderms and the holozoan lineage more broadly, we mined the sequenced genomes of sea stars^15,16^, sea urchins^16^ and sea cucumbers^14,17^ for predicted OSC genes using human *LSS* as a template (Supplementary Table 1). Phylogenetic analysis revealed two distinct clades within the Echinodermata: OSC genes from sea stars and sea urchins group together in clade I, while those from sea cucumbers form a distinct cluster, which we have named clade II (Extended Data Fig. 1). The differentiation of these latter OSC genes from those in clade I is suggestive of functional divergence (Extended Data Fig. 1). Two OSC genes were recovered from each of three different sea cucumber species. Sea cucumbers are unusual in having two OSC genes; all other animals have only one (Supplementary Table 2).

**Extended Data Fig. 2:**
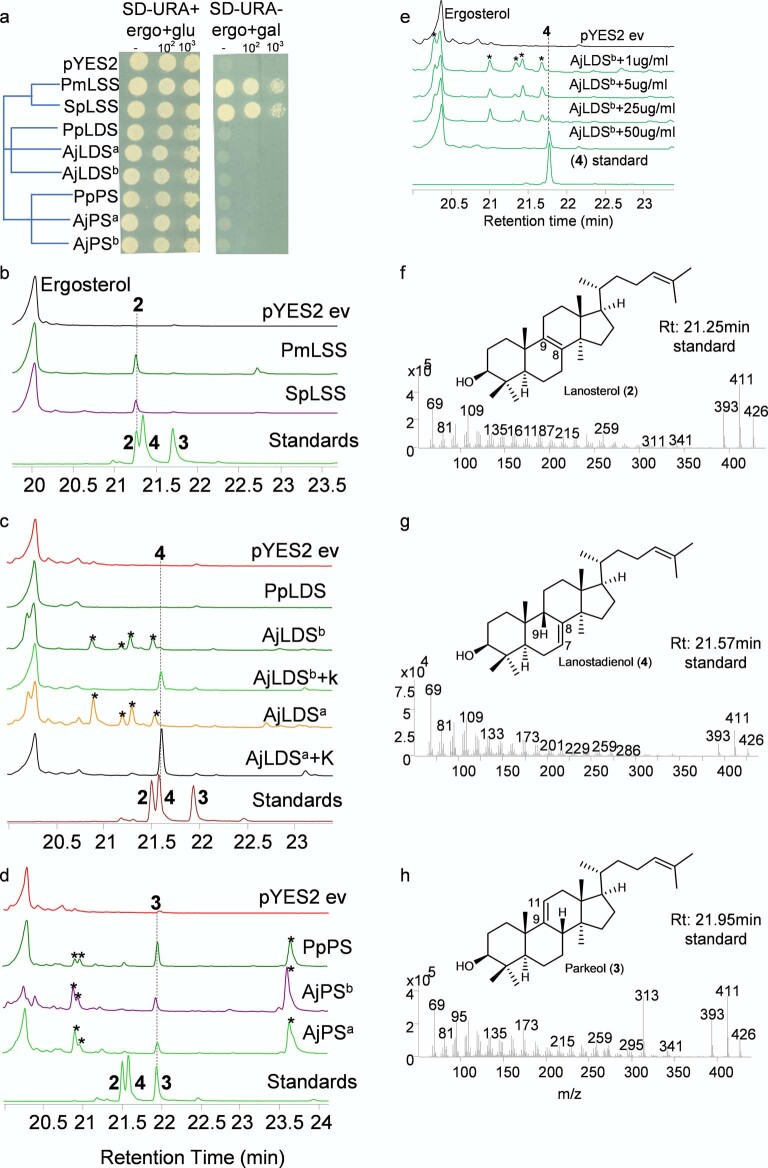
Functional analysis of sea urchin, sea star and sea cucumber OSCs in yeast. **a**, Complementation of the LSS-deficient yeast strain (Gil77) with echinoderm OSCs. Yeast transformants spotted undiluted (−), 10- and 100-folds dilution and growth recorded 7 days after spotting. GC-MS total ion chromatograms (TICs) of yeast extracts expressing **b**, sea star PmLSS and sea urchin SpLSS, **c**, sea cucumber LDS, **d**, sea cucumber PS. Superscripts ‘a’ and ‘b’ indicate different accessions of *A. japonicus*. Peaks marked with asterisks in **c** and **d** indicate background modifications of OSC products in yeast. **e**, GC-MS TICs of yeast extract expressing AjLDS^b^ treated with different concentrations of ketoconazole (CYP51 inhibitor) in the medium (1-50 µg/ml). **f-h**, GC mass spectra of lanosterol (**2**), lanostadienol (**4**) and parkeol (**3**). pYES2, yeast containing the empty vector (ev). Standards, **2**, lanosterol; **4**, lanostadienol; **3**, parkeol. Ergo = ergosterol, glu = glucose and gal = galactose.

The genes for the OSCs marked with red asterisks in Extended Data Fig. 1 were cloned, and their functions were determined by expression in the LSS-deficient yeast strain Gil77 (ref. ^18^). This enabled us to evaluate the OSCs for their ability to complement LSS deficiency in vivo and also to investigate the nature of the **1** cyclization products generated by gas chromatography (GC)–MS analysis of yeast cell extracts. OSCs were expressed under the control of the galactose-responsive *GAL1* promoter, which is repressed in the presence of glucose. In yeast, lanosterol is the precursor for the biosynthesis of the essential sterol ergosterol^18^. In the presence of exogenously supplied ergosterol and glucose, all yeast strains containing the different OSC constructs grew (Fig. 1c and Extended Data Fig. 2a). In the presence of galactose and the absence of exogenous ergosterol, two of the OSCs tested (sea star *Patiria miniata* LSS and sea urchin *Strongylocentrotus purpuratus* LSS) complemented the growth of Gil77, suggesting that they are functional LSSs (Fig. 1c). GC–MS analysis confirmed the presence of lanosterol in extracts from the strains expressing these two OSCs (Fig. 1d and Extended Data Fig. 2b,f).

None of the OSCs from sea cucumbers restored Gil77 growth in the absence of ergosterol (Fig. 1c and Extended Data Fig. 2a). This may be because either they were not expressed in functional form or alternatively because they make products other than lanosterol. GC–MS analysis of yeast extracts (cultured with ergosterol and galactose supplementation) revealed that two OSCs from the *Apostichopus japonicus* sea cucumber accessions (*A. japonicus* LDS^a^ and *A. japonicus* LDS^b^) both yielded a new peak with a retention time of 21.6 min (Fig. 1e and Extended Data Fig. 2c,e,g). Large-scale yeast expression, purification and NMR characterization showed this to be 9β-lanosta-7,24-dienol (lanostadienol (**4**)), a very closely related isomer of lanosterol with a double bond at the Δ7(8) carbon position as opposed to the Δ8(9) position (Supplementary Figs. 2a–d and 3a,b and Supplementary Table 3). These OSCs were therefore named lanostadienol synthases (LDSs). No activity was observed for the *Parastichopus parvimensis* sea cucumber candidate OSC *P. parvimensis* LDS (Fig. 1e). GC–MS analysis of extracts from yeast expressing *P. parvimensis* PS and *A. japonicus* PS^a^ and *A. japonicus* PS^b^ revealed a new peak that did not match the retention time of either lanosterol or lanostadienol (Fig. 1f and Extended Data Fig. 2d,h), which was subsequently shown by NMR to be 8β-lanosta-9,24-dienol (parkeol (**3**)) (Supplementary Fig. 3c and Supplementary Table 4). Parkeol is another close isomer of lanosterol and lanostadienol with the relevant double bond at the Δ9(11) position. These OSCs were therefore named parkeol synthases (PSs). There is no report of a dedicated lanostadienol synthase (LDS) as yet from any other organism. A single PS of unknown biological function has been reported in rice^19^. The identification of a dedicated PS in sea cucumbers is suggestive of a role for this enzyme in parkeol-type triterpene saponin biosynthesis as shown in Fig. 1b, right. The role of LDS in sea cucumbers is unknown.

### Analysis of sea cucumber saponins

**Fig. 2:**
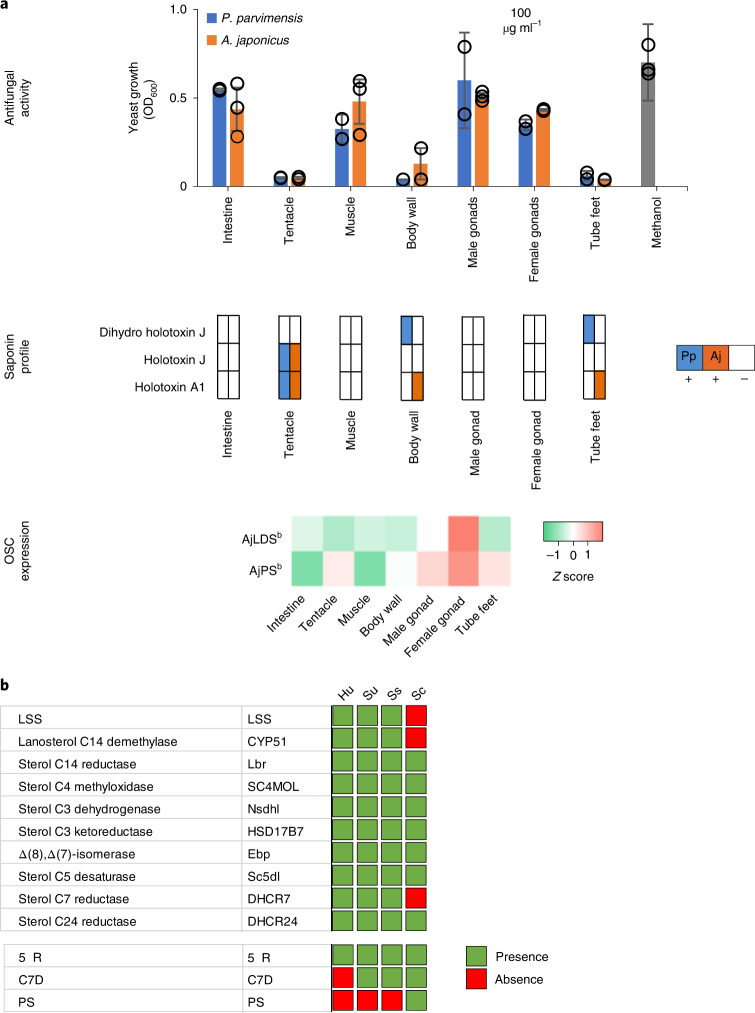
Biosynthesis of defense saponins in sea cucumbers. **a**, Antifungal activity, saponin profiles and OSC transcript levels for different sea cucumber tissues. Top, yeast growth (mean ± s.d., *P. parvimensis*, *n* = 2; *A. japonicus*, *n* = 3). Across all tissues, a 100 µg ml^−1^ crude extract was used with methanol as a control. Middle, presence (+) or absence (−) of saponins in *P. parvimensis* and *A. japonicus* based on LC–MS profiles (Extended Data Fig. 4a). Bottom, heatmap showing green (low) to red (high) OSC gene expression generated from reads per kb of exon model per million mapped reads (RPKM) values (*A. japonicus*, *n* = 3). RPKM mean ± s.d. values are given in the source data. OD_600_, optical density at 600 nm. **b**, Comparison of sea cucumber (Sc) sterol-biosynthetic genes with those of humans (Hu), sea urchins (Su) and sea stars (Ss). Presence or absence of sterol genes was scored based on pairwise ortholog identity as shown in Supplementary Table 8. Source data

To investigate the roles of PS and LDS OSCs, we analyzed different sea cucumber tissues for antifungal activity, saponins and OSC gene expression to establish whether expression of *LDS* and *PS* genes is correlated with bioactivity and/or saponin content. Sea cucumber saponins are highly antifungal^2^. We first evaluated extracts from different adult tissues of *P. parvimensis* and *A. japonicus* for inhibition of yeast growth as a sensitive but indirect measure of saponin content. Strong antifungal activity was detected for extracts from the tentacles, body walls and tube feet but not for the intestines, muscles or male or female gonads, with similar results for both sea cucumber species (Fig. 2a, top).

**Extended Data Fig. 3:**
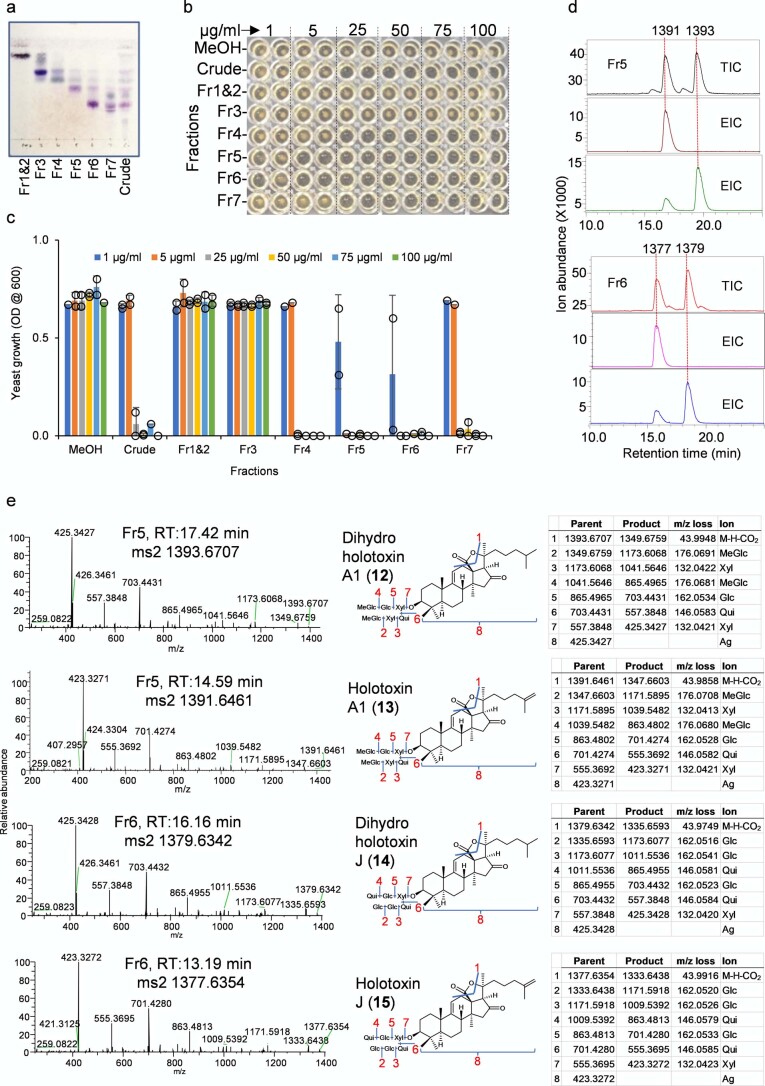
Characterization of sea cucumber saponins. **a**, Thin layer chromatography (TLC) of *P. parvimenis* sea cucumber crude extract and its semi-pure fractions (Fr1-Fr7). **b**, 96 well yeast growth inhibition (YGI) assay. **c**, Effect of sea cucumber crude extract and its semi-pure fractions on yeast growth (1-100 µg/ml). Optical density (OD600) was shown as mean ± SD (error bar) with n = 2 replicates. Fr, fraction (see Source Extended Data Fig. 3). **d**, LC-MS (−) total (TIC) and extracted ion chromatograms (EIC) of fractions Fr5 and Fr6. Saponin peaks of Fr5 and Fr6 are marked with their masses. Fr5; (M-H)^-^ m/z 1391 and m/z 1393, Fr6; (M-H)^-^ m/z 1377 and m/z 1379. **e**, Q Exactive high resolution LC-MS^2^ mass fragmentation spectra of saponins observed in fr5 (1391 and 1393) and fr6 (1377 and 1379), their deduced saponin structures showing nature of fragmentation (blue lines and red numbers) and high-resolution masses of parent and product ions etc. Glc; glucose, MeGlc; methyl glucose, Qui; quinovose, Xyl; xylose, Ag; aglycone. Aglycone mass is without the δ lactone moiety because it is lost as CO_2_ (M-H-CO_2_). Structural details on saponin sugar chains are given in Supplementary Fig. 4. Source data

To facilitate saponin identification in crude extracts, we isolated pure saponins from adult *P. parvimensis* sea cucumbers by large-scale extraction and purification, coupled with yeast growth inhibition and chromatographic analysis. Of the fractions tested, fractions 5 and 6 showed strong yeast growth inhibition (Extended Data Fig. 3a–c). These fractions each contained two major saponins as revealed by LC–MS (negative ion mode) (Extended Data Fig. 3d). These ions were further subjected to high-resolution LC–MS and MS^2^ analysis using a Q Exactive Plus Hybrid Quadrupole-Orbitrap mass spectrometer. Mass fragmentation of saponin *m*/*z* 1,393.6707 revealed it to be dihydro holotoxin A1 (**12**), and mass fragmentation of saponin *m*/*z* 1,391.6461 revealed it to be holotoxin A1 (Extended Data Fig. 3e), previously known saponins from *P. parvimensis*^20^ and *A. japonicus*^21^. However, mass fragmentation revealed *m*/*z* 1,379.6342 and *m*/*z* 1,377.6354 to be new holotoxins (Extended Data Fig. 3e). These two new holotoxins each have two quinovose sugar residues as opposed to the single quinovose in all other currently known holotoxins^20,21^ and hence were named dihydro holotoxin J (*m*/*z* 1,379.6342, **14**) and holotoxin J (*m*/*z* 1,377.6354, **15**) (Extended Data Fig. 3e). All four saponins are parkeol type with Δ9(11) functionality in the aglycone scaffold, and their structures are clearly consistent with the high-resolution LC–MS^2^ fragmentation pattern shown in Extended Data Fig. 3e.

**Extended Data Fig. 4:**
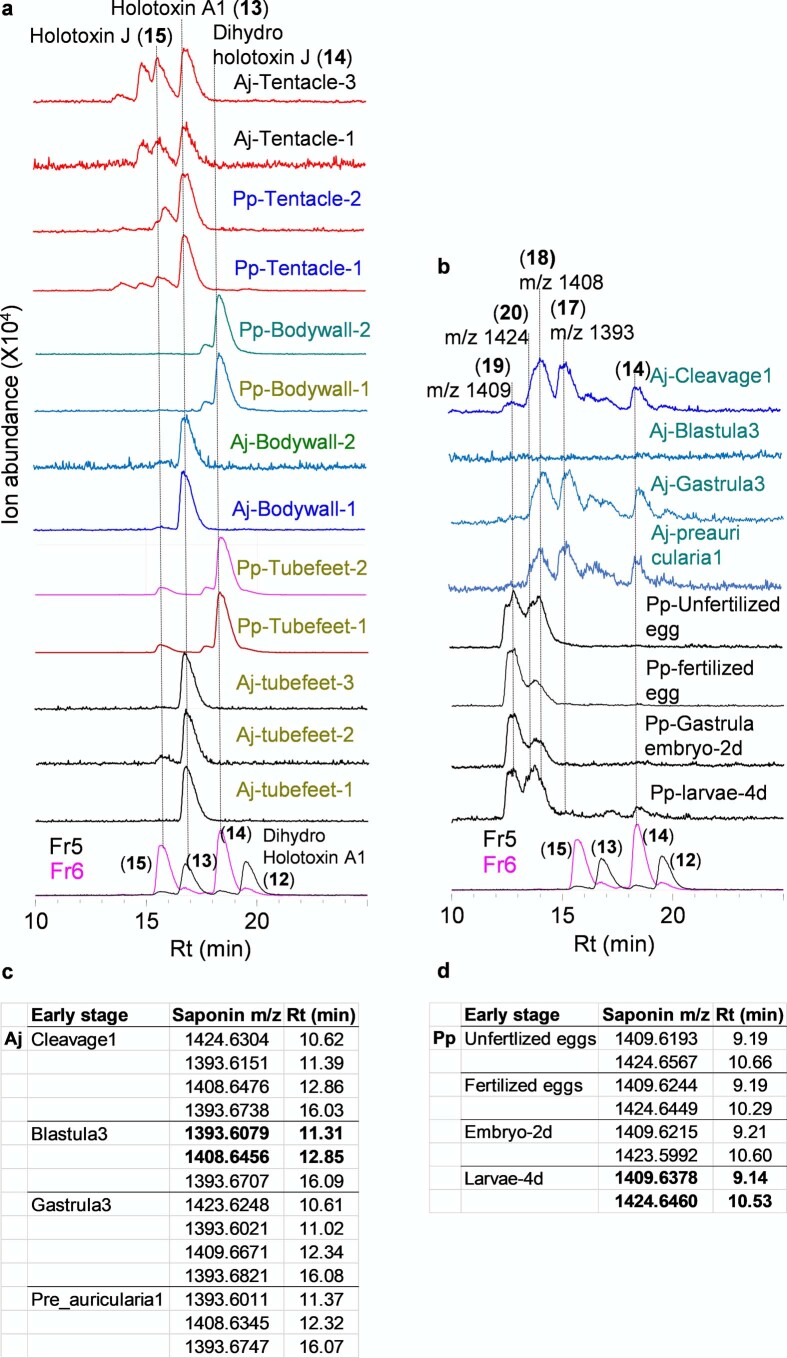
LC-MS (−) analysis of sea cucumber tissue extracts. **a**, LC-MS (−) TICs of sea cucumber adult tissue extracts and **b**, early stages. All tissue wise replicate profiles are shown. Aj, *A. japonicus* n = 3; Pp, *P. parvimensis* n = 2. Rt; retention time. Saponin peaks with same retention times and masses are connected by a dotted line and annotated with compound names or their masses. **c-d**, Q Exactive high resolution LC-MS analysis showing detection of saponin ions in *A. japonicus* (Aj) and *P. parvimensis* (Pp) early-stage extracts. Saponin ions highlighted in bold were subjected for LC-MS^2^ and its corresponding data shown in Extended Data Fig. 5b.

Using these saponins as standards, we next analyzed the tissue extracts for saponin content. LC–MS^2^ analysis confirmed the presence of parkeol-type saponins with Δ9(11) functionality in the aglycone scaffold, consistent with a role for PS in saponin biosynthesis in these tissues (Fig. 2a and Extended Data Fig. 4a). These included dihydro holotoxin A1, holotoxin A1, dihydro holotoxin J and holotoxin J. Holotoxin A1 and holotoxin J were found in extracts from the tentacles of both species. Holotoxin A1 was also detected in extracts from the body wall and tube feet of *A. japonicus*, while dihydro holotoxin J was predominant in extracts from the corresponding tissue types of *P. parvimensis* (Fig. 2a, middle, and Extended Data Fig. 4a). Selective accumulation of saponins in outer epidermal tissues (that is, tentacles, body wall and tube feet) may be anticipated to provide a first line of defense against pathogens or predators in the ocean. In fact, it has been shown that sea cucumber extracts inhibit the growth of surface-growing and pathogenic fungi of sea cucumbers, consistent with a direct role in defense^22^. Saponins were not detected in extracts from the intestines, muscles or male or female gonads of either species, in line with the lack of antifungal activity in these extracts (Fig. 2a, top and middle, and Extended Data Fig. 4a). Analysis of transcript levels of the two *A. japonicus* OSC genes revealed that *A. japonicus PS*^*b*^ was expressed at high levels in saponin-producing tissues, while *A. japonicus LDS*^*b*^ was not, consistent with a role for PS in saponin biosynthesis (Fig. 2a, bottom). Neither OSC gene was expressed in the intestines or muscles, tissues that lack detectable levels of saponins. By contrast, both *A. japonicus PS*^*b*^ and *A. japonicus LDS*^*b*^ were highly expressed in the gonads, but gonad extracts had little or no inhibitory activity toward yeast, suggesting that PS and LDS may have alternate roles in this tissue (for example, in steroid hormone biosynthesis or other functions) (Fig. 2a).

**Fig. 3:**
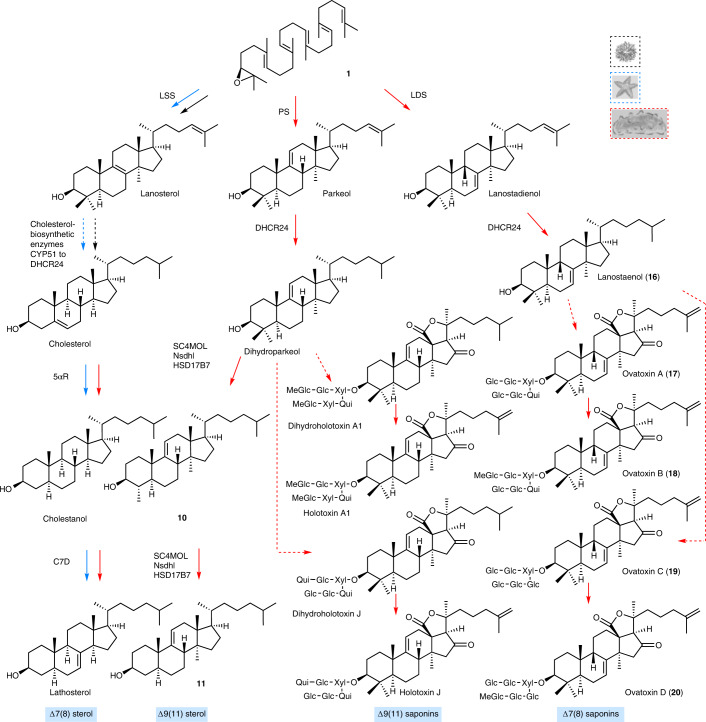
Sea cucumbers synthesize diverse triterpene saponins and unusual sterols. Roles of PS, LDS and C7D in the biosynthesis of unusual sterols (Δ7(8) and Δ9(11)) and triterpene saponins (Δ7(8) and Δ9(11)). The different colored arrows represent taxa-specific sterol- or saponin-biosynthetic routes. Solid and dashed arrows represent single and multi-step reactions, respectively.

**Extended Data Fig. 5:**
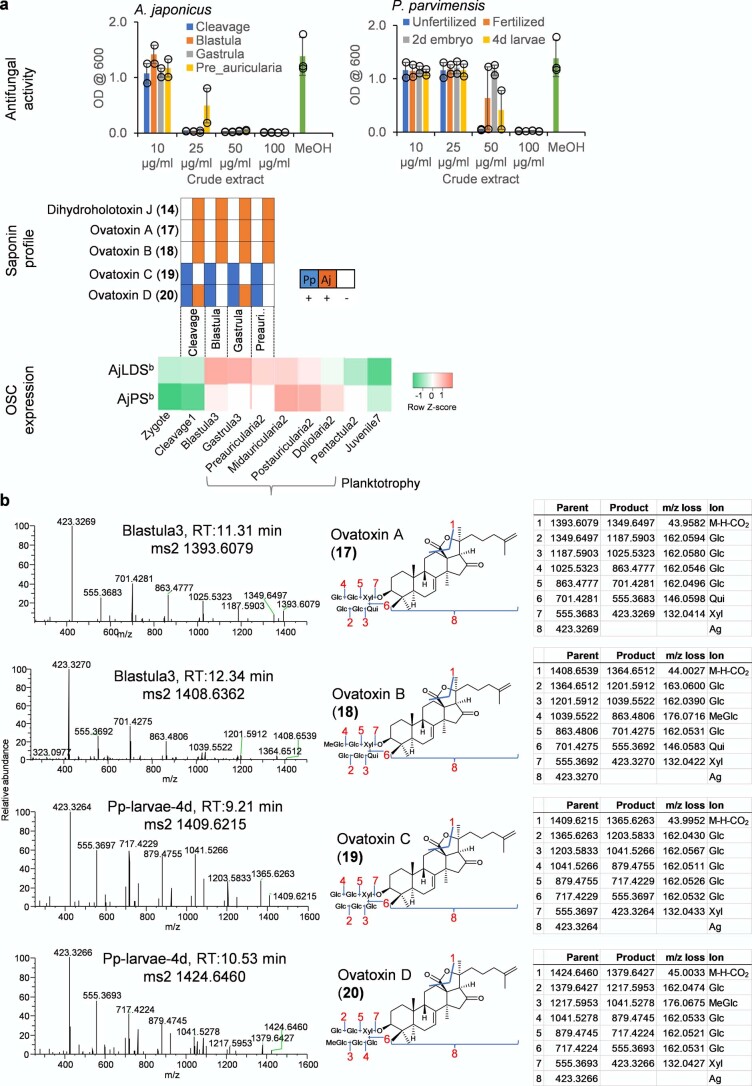
Antifungal activity, saponin content and OSC gene expression in sea cucumber juvenile stages. **a**, Top, inhibition of yeast growth by crude extracts of early growth stages of *A. japonicus* and *P. parvimensis*. Methanol (MeOH) used as a control (mean ± SD, n = 2) (see Source Extended Data Fig. 5). Middle, presence (+) or absence (−) of saponins in *P. parvimensis* (Pp) and *A. japonicus* (Aj) based on LC-MS profiles shown in Extended Data Fig. 4b–d. Bottom, heat map showing low (green) to high (red) OSC gene expression generated from RPKM values (*Aj*, n = 3). **b**, Q Exactive high resolution LC-MS^2^ mass fragmentation spectra of saponins observed in early-stage extracts, their deduced saponin structures and high-resolution masses of parent and product ions observed. Glc; glucose, MeGlc; methyl glucose, Qui; quinovose, Xyl; xylose, Ag; aglycone. Structural details on saponin sugar chains are given in Supplementary Fig. 4. Source data

The early growth stages of *A. japonicus* show distinct waves of expression of *A. japonicus LDS*^*b*^ and *A. japonicus PS*^*b*^ in the transition from zygote to juvenile (Extended Data Fig. 5a, bottom). Expression of OSC genes during the planktotrophic stage of larval development, in which larvae actively feed on algae and plankton in the top water column, could be associated with the production of saponins that counter predation. Compared to extracts from adult stages, extracts from the early growth stage showed strong yeast growth inhibition as well as distinct saponin peaks in LC–MS (Extended Data Fig. 4b–d, Extended Data Fig. 5 and Supplementary Fig. 4). NMR structural characterization of these saponins was not feasible due to low abundance of the compounds and source material. However, based on high-resolution LC–MS and MS^2^ analyses and correlation with *A. japonicus LDS*^*b*^ expression at these growth stages compared to adult stages, we identified these as new saponins, ovatoxins A–D (**17**–**20**) (Extended Data Fig. 5b). Because ovatoxin A–D accumulation correlates with *A. japonicus LDS*^*b*^ expression, these compounds are likely LDS type with Δ7(8) functionality in the saponin aglycone, providing evidence for a role for LDS in the biosynthesis of previously undescribed saponins in early growth stages (Fig. 3). Under laboratory conditions, sea cucumber eggs are known to be highly unpalatable to predatory fish and tunicates^23^, again suggesting a direct defense role of inherent saponins in early growth stages. Collectively, these results indicate that PS and LDS have distinct roles in the biosynthesis of parkeol-type saponins with Δ9(11) functionality and LDS-type saponins with Δ7(8) functionality, respectively (Fig. 3), as part of the sea cucumber chemical defense arsenal.

### Sea cucumbers make unusual sterols

**Extended Data Fig. 6:**
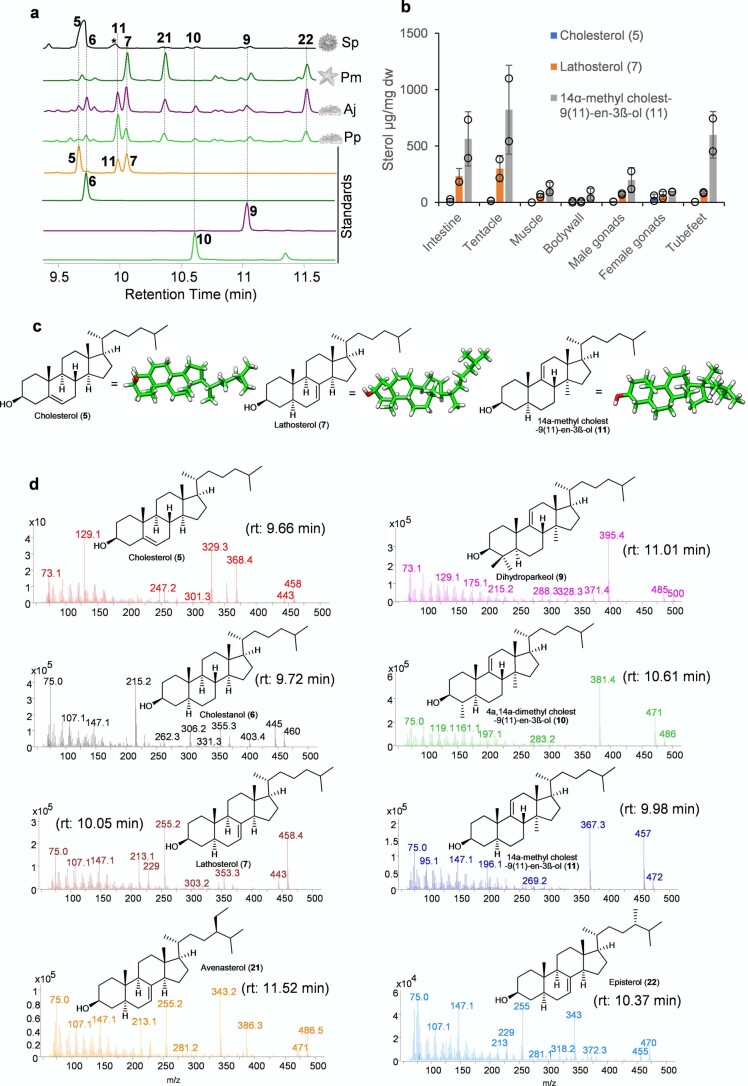
Sea stars and sea cucumbers make unusual sterols. **a**, Representative GC-MS profiles (TICs) of sea urchin, sea star and sea cucumber adult tissues. The peaks at 10.1, 10.4 and 11.52 min are lathosterol (**7)**, episterol (**22**), and avenasterol (**21**), respectively. Peak eluting at 9.98 min unique to sea cucumbers and was characterized to be 14α-methylcholest-9(11)-en-3β-ol (**11**) (Supplementary Tables 5–7). **b**, Levels of cholesterol (**5**), lathosterol (**7**) and 14α-methylcholest-9(11)-en-3β-ol (**11**) in adult tissues of *A. japonicus* (mean ± SD, n = 2). Dw, dry weight (see Source Extended Data Fig. 6). **c**, Unusual sterols renders sea star and sea cucumber membranes saponin resistant. 3D conformations of cholesterol (**5**), lathosterol (**7**) and 14α-methylcholest-9(11)-en-3β-ol (**11**) showing flat conformation of side chain in **5** and bent conformation in **7** and **11**. 3D conformations were optimized using Frog2 server with default parameters^60^. **d**, GC mass spectrum of the sterols observed. Avenasterol (**21**) and episterol (**22**) and were identified based on known spectra in the literature and others based on authentic standards. All sterols are TMS derivatives. Source data

Saponins form complexes with cholesterol, thus causing loss of membrane integrity and cell death. However, sea cucumbers have evolved the ability to produce high levels of lathosterol and **11** sterols rather than cholesterol, which may enable them to be resistant to their own saponins (Extended Data Fig. 6a,b). Sea urchins do not make saponins and lack lathosterol and **11** sterols (Extended Data Fig. 6a). External application of sea cucumber saponins to sea urchin eggs results in egg mortality^24,25^. By contrast, application of sea star saponins to sea star eggs does not cause mortality, implying that intrinsic lathosterol can confer resistance to saponin self-toxicity^25^. The alternative double-bond positions (Δ7(8) and Δ9(11)) as well as the conformation of the acyclic tail of lathosterol and **11** sterols may hinder the formation of stable interactions with saponins in membranes when compared with Δ5 cholesterol (Extended Data Fig. 6c).

**Extended Data Fig. 7:**
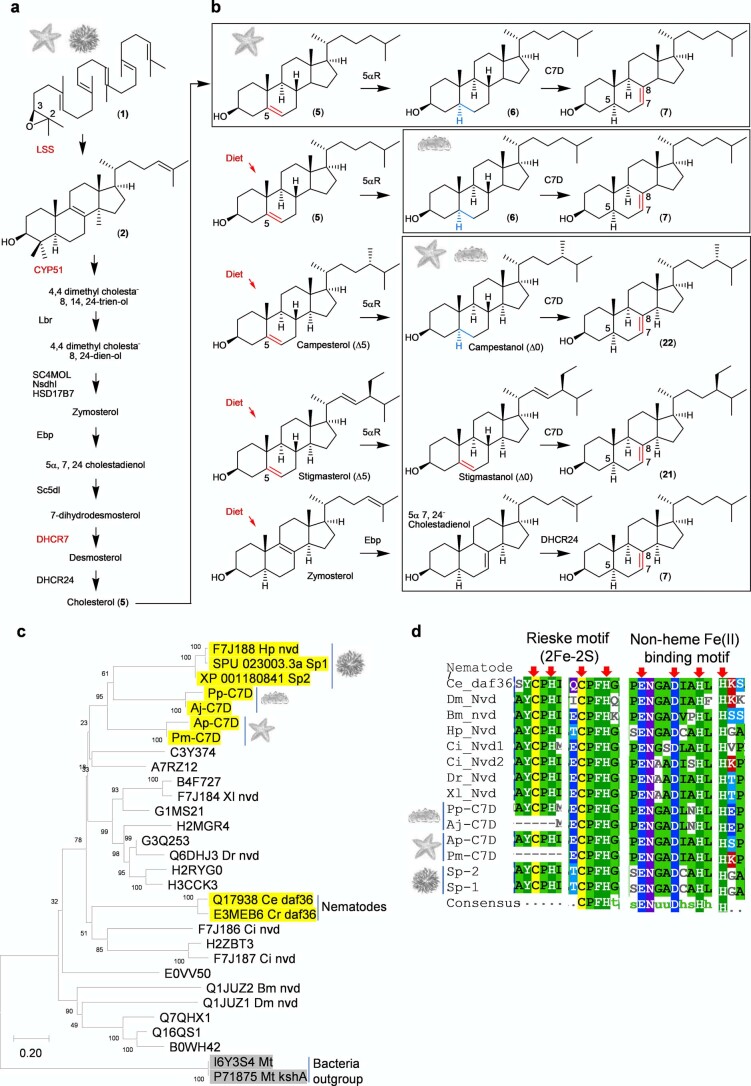
Sea stars and sea cucumbers evolved unusual sterol pathways. **a**, Canonical cholesterol biosynthetic pathway of animals including sea urchins and sea stars. **b**, Sea star and sea cucumber specific biosynthetic pathway of Δ7 sterols, lathosterol (**7)**, avenasterol (**21**) and episterol (**22**). Sea stars make lathosterol (**7**) from cholesterol (**5**) that is made *de novo* whereas sea cucumbers make lathosterol (**7**) through diet derived cholesterol (**5**) or other Δ5 sterols. **c**, Neighbor-joining tree of C7Ds (cholesterol 7-desaturse) hits from echinoderms and other functionally characterized sequences. Sequence names are uniprot sequence identifiers. **d**, Sequence alignment of C7D/DAF36 active site motifs of echinoderm as well as other functionally characterized sequences. Residue positions marked with red arrows are part of the Rieske motif which co-ordinates iron and sulfur required in cholesterol 7-desaturation.

The genes required for lathosterol and **11** sterol biosynthesis are currently unknown. As knowledge of lathosterol and **11** sterol biosynthesis will be critical for understanding how sea cucumbers have evolved saponin resistance, we predicted the biosynthetic route to these sterols based on pathway intermediates characterized in this study and enzymes reported in the literature with the aim of identifying the corresponding genes (Extended Data Fig. 6a,d, Supplementary Fig. 5a–e and Supplementary Tables 5–8). First, in **11** biosynthesis, detection of dihydroparkeol (**9**) and 4α,14α-dimethylcholest-9(11)-en-3β-ol (**10**) (Extended Data Fig. 6a) intermediates implies that **11** is derived from the triterpene precursor parkeol through (1) side-chain double-bond reduction of parkeol by a sterol C24 reductase (DHCR24) leading to dihydroparkeol and (2) eventual C4 demethylation of dihydroparkeol mediated by the C4-demethylation complex (SC4MOL, Nsdh and HSD17B7), which results in formation of the final **11** sterol (Fig. 3). The sterol **11** is unique to sea cucumbers, implying that it has a special role therein^26^. Secondly, in lathosterol biosynthesis, in the absence of a cholesterol-biosynthetic pathway, sea cucumbers must take up cholesterol from their diet, whereas sea stars synthesize cholesterol and also convert de novo synthesized or diet-derived Δ5 sterols to Δ7 sterols (Figs. 2b and 3 and Extended Data Fig. 7a,b). Detection of cholestanol (**6**) suggests that cholesterol 5α-reduction (mediated by a common 5α-sterol reductase (5αR)) precedes C7 desaturation in lathosterol biosynthesis (Fig. 3 and Extended Data Fig. 6a). A literature search revealed that a bona fide cholesterol 7 desaturase (C7D, known as DAF36 in nematodes) is known in nematodes^27^. Using this as an inquiry sequence, we identified C7D hits in echinoderms but not in humans (Fig. 2b). Phylogenetic analysis revealed that the echinoderm hits grouped with the nematode DAF36 (Extended Data Fig. 7c). Key amino acid residues known to be required for function were also conserved, suggesting that the echinoderm hits were likely sterol C7 desaturases (Extended Data Fig. 7d). C7D and PS play crucial roles in lathosterol and **11** sterol biosynthesis, respectively (Figs. 2b and 3). Collectively, our results suggest that PS and LDS have distinct and non-overlapping roles in vivo. To investigate this further, we carried out whole-mount mRNA in situ hybridization of *P. parvimensis* embryos and larvae to establish the spatial expression patterns of the *PS* and *LDS* OSC genes. In 2-d-old embryos, *P. parvimensis LDS* expression was detected in the ciliary bands, while *P. parvimensis PS* was expressed in the mouth region, consistent with distinct roles for these two OSCs (Supplementary Fig. 6a–c).

### Mutational analysis of determinants of OSC product specificity

**Fig. 4:**
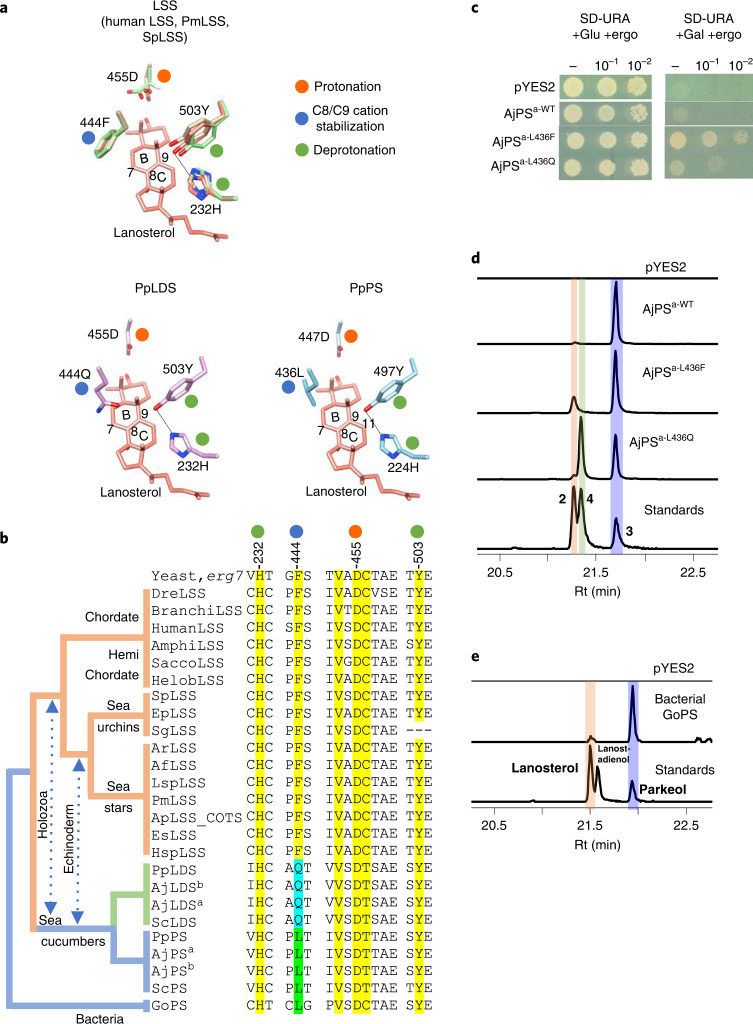
A single active residue underlies functional divergence of LDS and PS from LSS in echinoderms. **a**, Superpositioned homology models of sea star (*P. miniata* LSS), sea urchin (*S. purpuratus* LSS) and divergent sea cucumber OSCs (*P. parvimensis* LDS and *P. parvimensis* PS) showing variation at position 444 position near the B and/or C rings of lanosterol. Colored circles next to amino acid residues in the models represent different cyclization roles. Dashed lines represent a hydrogen bond between Y503 and H232, and numbering on B and/or C rings of lanosterol represents cationic regions. **b**, Structure-based sequence alignment of position 444 and its associated positions across echinoderm and holozoan OSCs. Clades are colored according to OSC product specificity. OSC sequence numbering is according to that of human LSS. Further information about the sequences used is provided in Supplementary Table 2. GoPS, *G. obscuriglobus* PS, Dre, *Danio rerio*; Branchi, *Branchiostoma floridae*; Amphi, *Amphimedon queenslandica*; Sacco, *Saccoglossus kowalevskii*; Helob, *Helobdella robusta*; Af, *Asterias forbesi*; Ar, *Asterias rubens*; Lsp, *Leptasterias sp*.; Hsp, *Henricia sp.*; Es, *Echinaster spinulosus*; Ap, *Acanthaster plancii* (COTS); Ep, *Echinarachnius parma*; Sg, *Sphaerechinus granularis*; Sc, *Stichopus chloronotus*. **c**, Complementation of an LSS-deficient yeast strain with *A. japonicus PS*^*a*^ wild type (WT) and mutants thereof. Yeast was spotted from stock cultures undiluted (−) and diluted tenfold and 100-fold. **d**,**e**, GC–MS profiles of yeast extracts from strains expressing *A. japonicus* PS^a^ wild type and active site mutants (**d**) and the bacterial OSC *G. obscuriglobus* PS (**e**). The corresponding total ion chromatograms for **d**,**e** are shown in Extended Data Figs. 8d and 9c, respectively. Standards were lanosterol, lanostadienol and parkeol.

**Extended Data Fig. 8:**
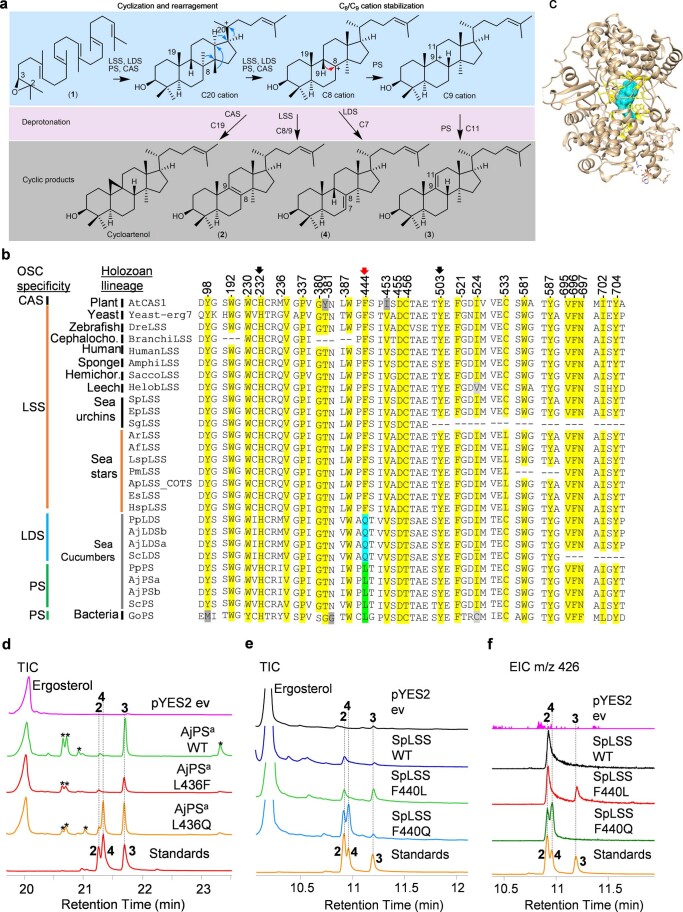
A single active site residue (position 444) determines cyclization mechanism and product specificity of LSS, LDS and PS OSCs. **a**, The cyclization mechanism for lanosterol (**2**), lanostadienol (**4**), parkeol (**3**) and cycloartenol. Protonation, cyclization, and rearrangement of 2, 3 oxidosqualene (**1**) to a central protosteryl cation (C20). Rearrangement of C20 cation lead to either C8 or C9 cations depending on type of OSC involved. Deprotonation at C7 of C8 cation lead to lanostadienol (**4**) and deprotonation at C11 of C9 cation lead to parkeol (**3**). Lanosterol (**2**) and cycloartenol are derived from C8 and C9 cations, respectively. **b**, OSC sequence alignment of 24 active site residue positions of echinoderms and others (sequences given in Supplementary Notes 2). **c**, OSC active site region is shown in yellow and region encompassing 5Å around lanosterol is in blue. **d**, GC-MS TICs of yeast extracts expressing AjPS^a-^WT and its mutants AjPS^a^-L436F, AjPS^a^-L436Q. Peaks marked with asterisks are undesired modifications of the PS product in yeast. **e-f**, TICs and EICs (m/z 426) of yeast extracts expressing SpLSS-WT and its mutants SpLSS-F440L, SpLSS-F440Q. Standards: **2**, lanosterol; **4**, lanostadienol; **3**, parkeol.

Our results suggest that PS is responsible for the first committed step in the biosynthesis of Δ9(11) saponins and Δ9(11) sterols and that LDS is responsible for Δ7(8) saponin biosynthesis (Fig. 3). Unraveling the evolutionary origins of LDS and PS may shed light on the origin of Δ9(11) and Δ7(8) triterpene saponins and Δ9(11) sterols in sea cucumbers. To this end, functional divergence of the LDS and PS OSCs from the highly conserved ancestral LSSs prompted us to investigate the active site residues of these enzymes. The cyclization products generated by LSS, LDS and PS enzymes differ in the position of the double bond in the tetracyclic region. The different isomeric products are a result of different termination points of 1,2-shift sequences originating from a common tetracyclic cationic intermediate (the protosteryl cation). This implies that these OSCs likely differ or diverge either in their kinetic promotion of the final deprotonation (which gives the neutral alkene) or in their ability to stabilize the relevant preceding cations (Extended Data Fig. 8a). To determine whether these OSCs differ in active site amino acid residues that might govern the site of deprotonation or C8 or C9 cation stabilization, we developed homology models for *P. miniata* LSS, *S. purpuratus* LSS, *P. parvimensis* LDS and *P. parvimensis* PS using the human LSS–lanosterol (Protein Data Bank 1W6K) complex as a template^28^. Superimposition of the models revealed a single amino acid residue within 5 Å of lanosterol that differed in LSS (444F), LDS (444Q) and PS (436L) (Fig. 4a and Extended Data Fig. 8b). This revealed that residue 444 is in a particularly promising position to control C8 and C9 cation stabilization directly, which in turn could influence the position of deprotonation during the reaction pathway (Fig. 4a). Next, using structure-based sequence alignment, we verified variability at the 444th residue position across echinoderm as well as the holozoan lineages. Comparison of amino acid residue positions within 5 Å of lanosterol (highlighted in yellow) revealed that residue 444 alone differentiated LDSs and PSs from LSSs (Fig. 4b and Extended Data Fig. 8b,c). Residues 232H and 503Y were within hydrogen bonding distance and are believed to form a catalytic dyad responsible for deprotonation in the formation of lanosterol^28^ (Fig. 4a). H232 and 503Y were invariant across LSS, LDS and PS OSCs, suggesting that variation at residue 444 alone determines product specificity (Fig. 4b). Natural selection may therefore have favored this mutation, leading to the origin of the divergent OSCs LDS and PS and to the emergence of Δ9(11) (**12**–**15**) and Δ7(8) (**17**–**20**) triterpene saponins as well as **11** sterol in sea cucumbers.

**Extended Data Fig. 9:**
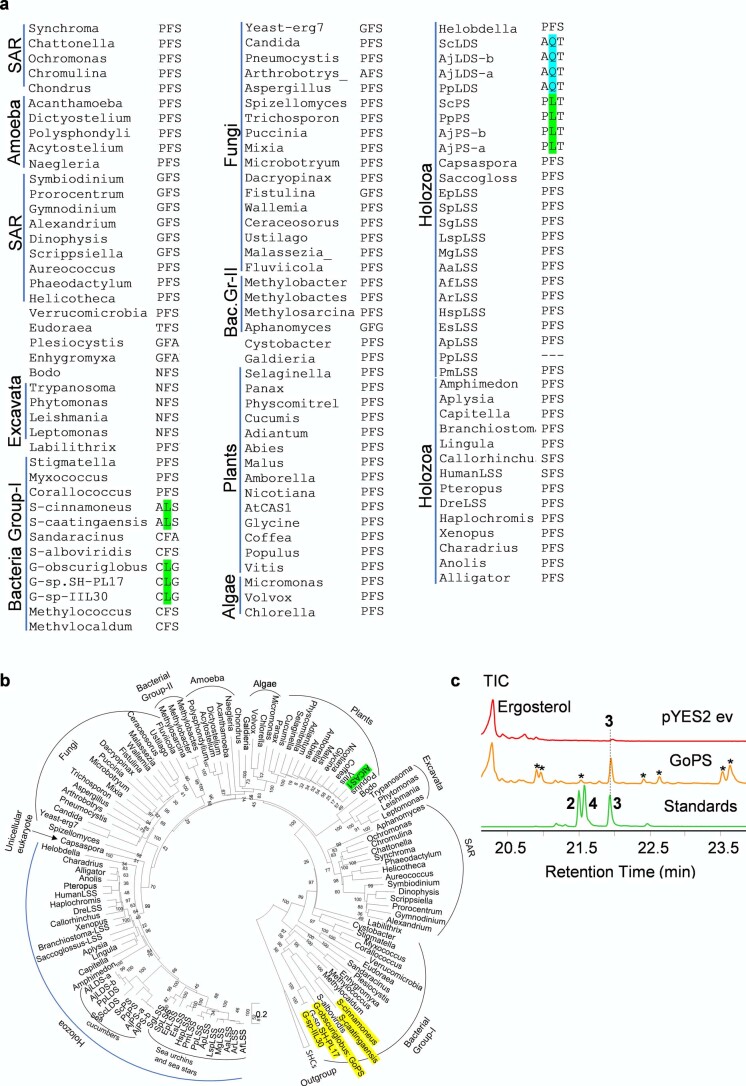
Discovery of *Gemmata obscuriglobus* parkeol synthase (GoPS). **a**, OSCs sequence alignment of residue position 444 from diverse taxa. Bacterial group-I OSCs with variation at residue position 444 are shown in green. Sequences used in phylogeny and sequence alignment are given in Supplementary Notes 2. **b**, Neighbor-joining tree of representative OSCs from diverse taxa. Holozoan clade (blue arc) includes all animal representatives. Bacterial squalene hopane cyclases (SHC) used as outgroups. Bacterial group-I OSCs with ‘L’ natural mutation (yellow). Bootstrap percentages higher than 50 are shown (500 replicates). **c**, GC-MS TICs of yeast extract expressing GoPS. Peaks marked with asterisks are undesired modifications of the PS product in yeast. Standards, **2**, lanosterol; **4**, lanostadienol; **3**, parkeol.

**Extended Data Fig. 10:**
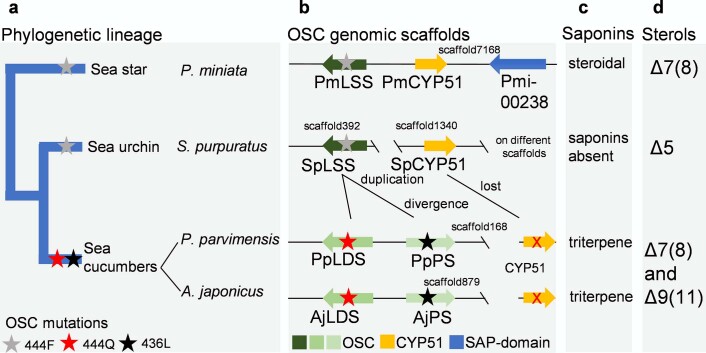
Model for evolutionary origin of saponins and unusual sterols in sea stars and sea cucumbers. **a**, Phylogeny of sea stars, sea urchins and sea cucumbers based on ref. ^16^. **b**, Genomic neighborhood of OSC genes. OSC genes are shown in different shades of green, and CYP51 genes in orange. Red cross in CYP51 denote gene loss. Colored stars denote OSC mutations at amino acid residue position 444. Scaffolds and gene modules are not to scale. **c**, Presence, or absence of saponins in sea urchins, sea stars and sea cucumbers. **d**, Major sterols of sea urchins, sea stars and sea cucumbers.

Next, we examined experimentally in three ways whether the nature of the amino acid residue at position 444 does indeed determine the product specificity of LSS, LDS and PS enzymes. First, an amino acid residue in the structurally homologous position was replaced with a residue from the equivalent position of another OSC. *A. japonicus* PS^a^ was chosen as a representative PS, and mutant versions with the equivalent LSS or LDS residues (*A. japonicus* PS^a-L436F^ and *A. japonicus* PS^a-L436Q^) were generated and expressed in yeast. As expected, the wild-type *A. japonicus* PS^a^ enzyme neither restored the ability of Gil77 to grow in the absence of exogenous ergosterol nor yielded lanosterol (Fig. 4c,d and Extended Data Fig. 8d). However, the mutant *A. japonicus* PS^a-L436F^ variant could restore the growth of Gil77 (Fig. 3c), indicating that it is able to produce lanosterol in vivo. GC–MS results confirmed the presence of lanosterol in extracts from yeast expressing this mutant PS (Fig. 4d and Extended Data Fig. 8d). The mutant variant *A. japonicus* PS^a-L436Q^ synthesizes lanostadienol in addition to parkeol but not lanosterol (Fig. 4d and Extended Data Fig. 8d). Second, we introduced corresponding mutations in *S. purpuratus* LSS to determine whether these mutations recapitulated PS- or LDS-like activity in LSS. The *S. purpuratus* LSS wild-type enzyme does not synthesize detectable levels of parkeol or lanostadienol. Interestingly, however, the *S. purpuratus* LSS^F440L^ mutant synthesizes lanostadienol in addition to lanosterol, and the *S. purpuratus* LSS^F440Q^ mutant synthesizes lanostadienol in addition to lanosterol (Extended Data Fig. 8e,f). Third, we investigated whether any other OSCs in the UniProt proteome^29^ have Q or L at the position equivalent to 444 instead of the conserved F. An exhaustive search revealed five hits, all with L at this position (Extended Data Fig. 9a). These five hits, all of which are proteins of unknown function, fell into the category of bacterial group I OSCs based on our phylogenetic analysis (Extended Data Fig. 9b). We cloned one of these from *Gemmata obscuriglobus* strain DSM5831^T^ (*G. obscuriglobus* OSC) and expressed it in yeast. GC–MS analysis revealed that *G. obscuriglobus* PS synthesizes parkeol as we predicted, confirming the pivotal role of 444L in parkeol product specificity (Fig. 4e and Extended Data Fig. 9c). Together, our results clearly implicate residue 444 in OSC product specificity and are consistent with a scenario in which LDS and PS enzymes have evolved from an ancestral sea cucumber LSS by gene duplication and neofunctionalization. The *PS* and *LDS* genes are in tandem in the *P. parvimensis* and *A. japonicus* sea cucumber genomes, suggesting that they may have arisen by gene duplication and divergence, and this may have occurred in parallel with sterol 14α-demethylase (CYP51) loss in sea cucumbers (Extended Data Fig. 10a–d). CYP51 enzymes are highly conserved across the animal, plant and fungal kingdoms and govern a key step in essential sterol biosynthesis^30^. However, unlike sea stars and sea urchins, sea cucumbers lack a *CYP51* gene.

## Discussion

Here we show that sea cucumbers lack LSS, an OSC that is essential for normal sterol biosynthesis in animals and that is highly conserved across other members of the animal kingdom. Instead, they have two divergent OSCs (PS and LDS) that respectively produce parkeol-type triterpene saponins in epidermal tissues and a new class of lanostadienol-type saponins (ovatoxins) in juvenile tissues and that are likely to have evolved from an ancestral LSS by gene duplication and neofunctionalization. Saponins are able to form complexes with membrane sterols, thus causing membrane disruption with associated cell death. Our results show that sea cucumbers produce high levels of the unusual sterols lathosterol and **11** sterols, rather than the typical animal sterol cholesterol. These sterols are saponin resistant and hence are likely to confer protection against self-poisoning. Collectively, our studies suggest that sea cucumbers have evolved the ability to produce saponins as well as saponin-resistant sterols concomitantly. The discovery of the key enzymes LSS, PS, LDS and C7D should now expedite the discovery and characterization of other downstream enzymes required for saponin biosynthesis in the sea cucumber.

## Methods

### Sea cucumber sampling and maintenance

#### Culturing and collecting adult tissues and early-stage samples of *P. parvimensis*

Adult animals of *P. parvimensis* sea cucumbers were collected off the southern Californian coast, USA. Spawning was induced in adult sea cucumbers by intra-coelomic injection of 100 nM NGLWY-amide, followed by heat shock in room temperature sea water as described previously^31^. Fertilization was conducted by mixing freshly shed eggs with dilute sperm. Embryos were cultured in artificial seawater at 15 °C until the desired stage. Late gastrula–larva-stage embryos were collected at 2 d post-fertilization (dpf), and early auricularia larvae were collected at 4 dpf. Nine different adult tissues were collected from *P. parvimensis* sea cucumbers and flash-frozen until further use.

#### Culturing and collecting adult tissues and early-stage samples of *A. japonicus*

Adult *A. japonicus* sea cucumbers were collected from the coast of Liaoning, China and acclimatized in a seawater aquarium (~500 l) at 15 °C for 1 week before use. They were fed once a day during this period with mixed feed ingredients including 40% fresh sea mud, 30% *Sargassum thunbergii* and 30% sea cucumber compound feed (An-yuan). Major adult organs (intestine, tentacle, muscle, body wall, male gonads, female gonads and tube feet) were dissected from three healthy adult sea cucumbers and flash-frozen in liquid nitrogen for further use. To obtain the early-stage materials of *A. japonicus*, artificial fertilization of sexually matured adults was performed as described previously^32^. Larval culture was developed based on a procedure described previously^32^. Embryos (zygote, cleavages, blastulae and gastrulae), larvae (auricularia, doliolaria and pentactula) and juveniles were sampled and flash-frozen until use.

### Oxidosqualene cyclase mining

Genome and transcriptome resources^14–17,33,34^ were used to mine for predicted echinoderm OSC sequences. Echinobase^16^ hosts genome sequences and ovary transcriptome data for diverse species of Echinodermata, including sea urchins, sand dollars, sea stars and sea cucumbers. These available echinoderm sequence resources^16^ were mined using the human *LSS* sequence as a template (UniProt ID P48449)^28^, and predicted full-length OSC open reading frames (ORFs) (~2.2 kb; ~720 amino acids) were recovered. In cases in which only partial OSC hits were identified, multiple small contigs were assembled to give full-length OSC ORFs. Contig assembly was performed using the ContigExpress tool in Vector NTI (version 11.5.3), and ORF or gene prediction was carried out using Fgenesh^35^. In this study, two different accessions of *A. japonicus* were used for OSC mining. Sequence resources for the accession ‘a’ were derived from Echinobase^16^ and Reich et al.^33^ (BioProject accession no. PRJNA236087). We recovered two OSCs from this accession and annotated them with the superscript ‘a’ throughout the study. The *A. japonicus* accession ‘b’ was derived from Li et al.^14^ (BioProject accession no. PRJNA413998). OSC hits from this accession were annotated with the superscript ‘b’. A predicted full-length *LSS* from Echinobase (*S. purpuratus LSS*)^16^ was also assembled from sea urchins. All contigs used in full-length OSC assembly and their associated details are listed in Supplementary Tables 1 and 2. Further, predicted OSC amino acid sequences were scanned for the presence of the key active site motif DCTAE, which is implicated in protonation of **1** and initiation of the cyclization reaction^36^.

### Phylogenetic analysis

OSC amino acid sequences were aligned using ClustalW (version 2.1) with default parameters as implemented in the program MEGA7 (version 10.2.6)^37^ and the CLC Genomics Workbench (version 9.5.3). Positions with gaps and missing data were eliminated. Evolutionary distances were computed using the JTT matrix-based method^38^. Phylogenetic analysis of OSC sequences was carried out using a neighbor-joining tree with 1,000 bootstrap replicates^39^.

### Cloning

*P. miniata LSS* was cloned from cDNA of embryos (2 d old)*. P. parvimensis OSC1* and *P. parvimensis OSC2* were cloned from mixed cDNA of *P. parvimensis* early developmental stages (embryos, 2 d old; larvae, 4 d old). These were amplified by PCR using primers with attB1 and attB2 adaptors and cloned into pDONOR207 (gentamicin resistance, Invitrogen) through a BP recombination reaction. PCR conditions were as follows: initial denaturation (95 °C, 1 min), 30 cycles of 95 °C for 30 s, 58 °C for 1 min and 72 °C for 4 min; final elongation step of 7 min at 72 °C. The PCR reaction mix contained 2 μl cDNA, 10 μM of each primer, 250 μM of each dNTP, 1× Phusion buffer and 1 U Phusion DNA polymerase (NEB). After the BP reaction and transformation, colony PCR was performed using GoTaq Green Master Mix (Promega), and positive clones were validated by sequencing. Positive clones were sequenced using three sets of primers covering the entire length of the gene (~2.2 kb). The bacterial *OSC* candidate was cloned from genomic DNA of the *G. obscuriglobus* DSM5831^T^ strain (*G. obscuriglobus OSC*). Error-free clones were then cloned into pYES2 (ampicillin resistance) through yeast homologous recombination. All primers used are listed in Supplementary Table 9 (Sigma-Aldrich).

### Gene assembly using yeast homologous recombination

The *S. purpuratus LSS*, *A. japonicus OSC1*^*a*^*, A. japonicus OSC2*^*a*^, *A. japonicus OSC1*^*b*^ and *A. japonicus OSC2*^*b*^ coding sequences were synthesized as gBlocks by Integrated DNA Technologies. The gBlocks were then recombined to give full-length genes by homologous recombination in yeast^40^. All yeast homologous recombination and expression analysis was carried out in the yeast strain Gil77 (*gal2 hem3-6 erg7 ura3-167*)^18^. The gBlocks were amplified by PCR using the primers listed in Supplementary Table 9. Each primer contains a region that overlaps with the pYES2 vector sequences, with the 5′ end of the forward primer overlapping with the *GAL1* promoter and the 5′ end of the reverse primer overlapping with the *CYC1* terminator, whereas the 3′ ends of the primers overlap with the beginning and end of the respective gBlocks. Amplified gBlocks were then cotransformed into Gil77 together with the pYES2 vector (ampicillin resistance, Invitrogen) linearized with XbaI and HindIII (Invitrogen). Yeast transformation was performed using a standard protocol (Yeastmaker Yeast Transformation System 2). Transformation resulted in in vivo recombination between the pYES2 vector and the gBlock OSC fragments, leading to generation of full-length expression constructs. Plasmids were recovered from the yeast and transformed back into *Escherichia coli* (DH5α), and their sequences were verified through sequencing. All primers used are listed in Supplementary Table 9.

### Yeast expression

Yeast expression was carried out using the strain Gil77 (ref. ^18^). Yeast strains containing different expression constructs were grown in selective medium (SD-URA with 2% glucose and supplements) (5 ml) at 28 °C until saturation (~2 d). The supplements included 20 µg ml^−1^ ergosterol (Fluka), 13 µg ml^−1^ hemin (Sigma-Aldrich) and 5 mg ml^−1^ Tween-80 (Sigma-Aldrich). Next, cells were pelleted, washed with water (5 ml), transferred to induction medium (SD-URA with 2% galactose) and incubated for a further 2 d to allow OSC expression and accumulation of triterpenes. Yeast pellets were washed once with dH_2_O and stored at −80 °C until extraction.

### Yeast lanosterol synthase complementation

Gil77 is an LSS-mutant strain and is unable to grow in the absence of exogenously supplied sterol (ergosterol)^18^. This phenotype can be rescued by complementation with an LSS OSC because OSC-derived lanosterol is converted to ergosterol by endogenous yeast enzymes. For complementation analysis, Gil77 OSC transformants were spotted onto the following media in tenfold serial dilutions: (1) SD-URA glucose plates supplemented with ergosterol, Tween-80 and hemin and (2) SD-URA with galactose and without ergosterol. The *GAL1* promoter of the pYES2 construct drives gene expression in the presence of galactose and is repressed in the presence of glucose. Thus, any OSC that synthesizes lanosterol in vivo is expected to complement Gil77 growth in the absence of exogenous ergosterol and the presence of galactose.

### Triterpene extraction

Frozen yeast pellets (~50 mg, fresh weight) were mixed with 0.5 ml saponification reagent (20% KOH in 50% ethanol, vol/vol) and incubated at 65 °C for 2 h before extraction with an equal volume of hexane (0.5 ml). The hexane extraction was repeated (2×) to maximize triterpene recovery. The combined extract was dried under nitrogen gas, and the residue was dissolved in 0.5 ml *n*-hexane. For rapid qualitative analysis, extracts were run on thin-layer chromatography (TLC) plates (70644, Fluka) using a hexane:ethyl acetate (6:1, vol/vol) solvent system. Compounds were visualized by spraying the plates with acetic acid:H_2_SO_4_:*p*-anisaldehyde (48:1:1, vol/vol/vol) and heating them to 120 °C for 5 min on a TLC plate heater (CAMAG).

### Gas chromatography–mass spectrometry analysis

For GC–MS analysis, 100-µl aliquots of *n*-hexane extracts from yeast experiments were transferred to 150-µl vial inserts and analyzed using an Agilent GC (7890B)–MSD (5977A) with a robotic multi-purpose auto-sampler. Samples were run on an HP-5MS column (inner diameter, 30 m × 0.25 mm; 0.25-µm film, Phenomenex). The injector port, source and transfer line temperatures were set to 250 °C. An oven temperature program from 80 °C (2 min) to 290 °C (30 min) at 20 °C min^−1^ was used. The carrier gas was helium; the flow rate was 1.2 ml min^−1^. Samples (2 µl) were injected in splitless mode. For sterol quantification, the following gradient was used. Pulse pressure was set to 20 psi for 0.5 min after injection, and the inlet was purged after 0.5 min with a split vent flow at 100 ml min^−1^. Samples (2 µl) were injected in a pulsed split mode (5:1 split ratio). The injector port, source and transfer line temperatures were set to 250 °C. An oven temperature program from 150 °C (2 min) to 270 °C (6.5 min) at 20 °C min^−1^ was used, followed by 300 °C at 4 °C min^−1^ over 14 min, ramping up to 360 °C at 40 °C min^−1^ and then holding at 360 °C for 7 min. The carrier gas was helium, and the flow rate was 1.0 ml min^−1^. The output was used to search the NIST (version 8) library to assign identity to common peaks in the GC–MS traces. All experiments were repeated to confirm reproducibility of the triterpene profiles.

### Inhibition of endogenous yeast ERG11

In LDS OSC experiments, ketoconazole was used to control undesired OSC product modifications by the endogenous yeast enzyme ERG11. Ketoconazole is a lanosterol 14α-demethylase (ERG11–CYP51) inhibitor. Ketoconazole (K1003, Sigma-Aldrich) was dissolved in DMSO and used in SD-URA medium with 2% galactose (50 µg ml^−1^ = ~100 µM).

### Triterpene standards

Lanosterol was purchased commercially (L5768, Sigma-Aldrich), while lanostadienol and parkeol were purified in the present study (see below). These compounds were dissolved and diluted to 0.5 mg ml^−1^ in *n*-hexane and used as standards in GC–MS analysis.

### Purification of parkeol and lanostadienol from yeast

Yeast strains expressing LDS or PS were grown (5 l) in minimal medium (SD-URA with 2% glucose and supplements) and induced (SD-URA with 2% galactose) for 2 d as described above. Cells were then pelleted by centrifuging at 4,000 r.p.m. for 15 min (SLC6000 rotor, Sorvall Evolution) and washed once with dH_2_O (500 ml) before extraction. Washed cell pellets (~50–60 g fresh weight) were resuspended in 500 ml saponification reagent (20% KOH in 50% ethanol, vol/vol) and incubated in a water bath (65 °C for 2 h) before extraction with an equal volume of hexane. Hexane extraction was repeated (3×) to maximize triterpene recovery. The extract was then dried in vacuo using a rotary evaporator. The organic residue (~200–300 mg) was dry loaded onto pre-made silica columns (Biotage SNAP Ultra 10g, 21 × 55 mm, 25 µm) that had been equilibrated with hexane. Separation was carried out using an advanced automated flash chromatographic purification system, Isolera One (Biotage). Chromatographic runs were carried out using a gradient of ethyl acetate (solvent B) in hexane (solvent A). Gradient elution was initiated with hexane for ten column volumes and changed to ethyl acetate in hexane (2–30%, vol/vol) over 25 column volumes, and fractions (5 ml) were collected throughout the run. Fractions were assessed for triterpene content using TLC (silica gel 60 F_254_ plates, Merck). The fractions containing the relevant triterpenes were pooled, and purity was assessed by GC–MS (as described above). Based on the purity, purification was repeated to ensure clear separation of closely resolving compounds. Fractions containing pure parkeol and lanostadienol were combined, dried using a rotary evaporator (BUCHI) and subjected to NMR.

### Nuclear magnetic resonance analysis

One-dimensional and two-dimensional NMR spectra (^1^H, ^13^C, DEPT135, COSY, HSQC, HMBC and NOESY) were acquired on a Bruker 400-MHz TopSpin NMR spectrometer. All signals were acquired at 298 K. Samples were dissolved in deuterated chloroform (CDCl_3_) for data acquisition, and calibration was performed by referencing to either residual solvent ^1^H and ^13^C signals or tetramethylsilane. Compound identities were confirmed by comparing their ^1^H and/or ^13^C NMR data with those reported previously in the literature^41,42^.

### Large-scale saponin extraction and purification from processed sea cucumbers

Processed *P. parvimensis* sea cucumbers (~1.75 kg, dry weight) (WK Distribution) were soaked in distilled water for 2 d to allow swelling and softening. Soft and swollen sea cucumbers were pulverized and soaked in 100% ethanol (vol/vol) for 2 weeks. The ethanol extract was filtered (Whatman no. 1 filter paper) and dried in vacuo using a Rotavapor R-300 (BUCHI) at 30 °C. The remaining aqueous layer was extracted with *n*-hexane. The *n*-hexane extract was used in the purification of sterols as described below. After *n*-hexane extraction, the aqueous extract was partitioned with *n*-butanol (1:5 ratio, vol/vol) and centrifuged (4,000 r.p.m., SLC6000 rotor, Sorvall Evolution) to enable phase separation. *n*-butanol partitioning was repeated (3×) to ensure efficient recovery of saponins. The combined *n*-butanol extracts were dried under vacuum at 56 °C using a Rotavapor R-300 (BUCHI). The resulting extract (greasy yellowish) was freeze-dried to remove any residual *n*-butanol. The freeze-dried extract (~3 g) was dry loaded onto a normal-phase silica column (Biotage SNAP Ultra 100g, 39 × 157 mm, 25 µm) and separated using an advanced automated flash chromatographic purification system (Isolera One). Separation was carried out with a gradient of dichloromethane (DCM, solvent A) and methanol (7.5% water in methanol, solvent B). The run was initiated with DCM, changed to 15% methanol (vol/vol) in DCM (vol/vol) over 50 column volumes and then changed to 30% methanol (vol/vol) in DCM (vol/vol). During this process, 22-ml fractions were collected, dried and analyzed for saponins by TLC (Silica gel 60G glass plates, 20 × 20 cm, Merck) using avenacin A-1 from oats^43^ as a tracking standard. Saponins were visualized by spraying the plates with acetic acid:H_2_SO_4_:*p*-anisaldehyde (48:1:1, vol/vol/vol) and heating them to 120 °C for 5 min on a TLC plate heater (CAMAG).

### Yeast growth-inhibition assays

Yeast growth-inhibition assays were carried out using the yeast strain Y21900 (BY4743; *MATa*/*MATα*;*ura3Δ0*/*ura3Δ0*;*leu2Δ0*/*leu2Δ0*;*his3Δ1*/*his3Δ1*;*met15Δ0*/*MET15*;*LYS2*/*lys2Δ0*;*YHR072w*/*YHR072w*::kanMX4, Euroscarf). Y21900 was grown in YPD medium. A stock solution of crude extracts and saponin standards (1 mg ml^−1^) were prepared in methanol, and lower concentrations were prepared by serial dilution. All yeast growth-inhibition assays were carried out in 96-well plates (skirted, round bottom, Sterilin 96). Adult tissues and samples from early growth stages of *P. parvimensis* (*n* = 2) and *A. japonicus* (*n* = 3) were used in the analysis. Two technical replicates were also included to account for low-volume pipetting errors, etc. Sea cucumber early developmental-stage samples consisted of several hundred embryos or larvae per sample. Assays were carried out in 100 µl YPD medium seeded with 1 µl yeast culture (100-fold dilution of an overnight culture). Saponins (1–100 µg ml^−1^, 1–5 µl) and crude extracts (25–100 µg ml^−1^, 1–5 µl) were added at the beginning of the culture period, and plates were incubated in a shaking incubator (30 °C overnight, 100 r.p.m.). Growth was recorded by measuring OD_600_ using a FLUOstar Omega multidetector microplate reader (BMG LABTECH) with default settings. Sample OD values were corrected against OD values of wells containing YPD medium alone (called ‘blank corrected’).

### Saponin analysis and characterization using the Q Exactive Plus Hybrid Quadrupole-Orbitrap mass spectrometer

UHPLC–MS analysis of saponins was carried out on a Q Exactive mass spectrometer (Thermo Scientific). Chromatography was performed using a Kinetex 2.6-μm XB-C18 100-Å, 50-mm × 2.1-mm (Phenomenex) column kept at 30 °C. Water containing 0.1% formic acid and acetonitrile containing 0.1% formic acid were used as mobile phases A and B, respectively, with a flow rate of 0.3 ml min^−1^ and an injection volume of 10 µl. A gradient elution program was applied as follows: 0–5 min, linear increase of 0–30% B; 5–28 min, linear increase of 30–50% B; 28–33 min, linear increase of 50–100% B; 33–34 min, linear drop from 100% to 20% B; 1-min hold for re-equilibration, giving a total run time of 35 min. A full scan in combination with data-dependent MS^2^ scans (full MS/dd-MS^2^, top three) was applied. MS detection was performed in a negative ionization ESI range of 100–2,000 *m*/*z* and at a mass resolution of 70,000. Targeted selected ion monitoring and parallel reaction monitoring acquisition were performed with mass resolution set at 35,000 FWHM and a mass isolation window of 1.6 *m*/*z* and an AGC of 3 × 10^6^. In parallel reaction monitoring mode, data were acquired according to a predetermined inclusion list containing the accurate masses of known saponins. Data were acquired and processed using Thermo Scientific Xcalibur software (version 4.3.73.11). Saponin mass fragmentation spectra were compared with previously reported saponins from *P. parvimensis*^20^ and *A. japonicus*^21^.

### Small-scale saponin extraction from adult tissues and early-stage samples

To establish whether saponin accumulation is tissue specific, at a small scale, saponin activity and saponin content were analyzed by yeast growth-inhibition assays and LC–MS over a range of sea cucumber tissues and early growth stages. Seven adult tissues were collected from two different species of sea cucumbers, *P. parvimensis* (*n* = 2) and *A. japonicus* (*n* = 3). Tissue samples were collected in 1.5-ml Eppendorf tubes, freeze-dried and homogenized using a GenoGrinder (1,500 r.p.m., 1 min). Homogenized tissues of a known weight (~2–3 mg, dry weight) were extracted twice with methanol (200 μl) in a sonication bath (EMAG ultrasonic cleaner Emmi 12 HC, 45 min). Combined extracts were centrifuged (14,680*g*, 5 min), and supernatants were transferred to preweighed 1.5-ml Eppendorf tubes. Extracts were dried in vacuo using a ROTAVAC (EZ-2 Series Evaporator, Genevac) at 30 °C for 45 min. Samples were then freeze-dried to remove any residual methanol. Stock solutions (1 mg ml^−1^ and 100 μg ml^−1^, wt/vol) were prepared and serially diluted in methanol.

### Liquid chromatography–mass spectrometry analysis of extracts from adult tissues and early-stage samples

An LC method was designed with a flow rate of 0.3 ml min^−1^ (2.6-μm XB-C18 100-Å, 50 mm × 2.1 mm) (Phenomenex). The solvents used were solvent A (water with 0.1% formic acid) and solvent B (acetonitrile with 0.1% formic acid). Sea cucumber adult tissues and early-stage extracts (100 μg ml^−1^) were analyzed using LC–MS–CAD (instrument, Prominence HPLC system, single quadrupole mass spectrometer, LCMS-2020, Shimadzu) both in positive and negative ion modes. The gradient was as follows: 30% B, 0–5 min; 20–50% B from 5 to 28 min; 50–100% B from 28 to 30 min; 100% B from 30 to 33 min; 100–20% B from 33 to 34 min; 20% B from 34 to 35 min; injection volume, 10 µl. LC–MS LabSolutions (version 3) (Shimadzu) was used to analyze chromatograms.

### Purification and characterization of unusual sterols from sea cucumbers

The crude *n*-hexane extract recovered from phase separation during large-scale saponin extraction was dried in vacuo in a rotovapor (BUCHI). Around 2 g of the greasy yellowish crude extract was dry loaded onto a normal-phase silica column (Biotage SNAP Ultra 100g, 39 × 157 mm, 25 µm) that had been pre-equilibrated with *n*-hexane. Separation was carried out using Isolera One (Biotage). Chromatographic separation was carried with a gradient of 0–3% ethyl acetate (solvent B) in *n-*hexane (solvent A) over 60 column volumes, followed by a gradient of 3–20% solvent B over ten column volumes. Fractions (22 ml) were collected and evaluated by TLC as described earlier. Fractions with sterols of interest were pooled, and purity was assessed by GC–MS analysis as described earlier. Purified compounds were subjected to NMR in CDCl_3_ (Sigma-Aldrich). Compound identities were confirmed by comparing their ^1^H or ^13^C NMR data with those reported previously^44–50^.

### Analysis of sterols from sea urchins, sea stars and sea cucumbers

Sterols were extracted from different adult tissues of sea cucumbers, sea urchins and sea stars (2 mg, dry weight). Dried tissues were extracted in ethyl acetate (100 µl) with 10 µg ml^−1^ internal standard (hexatriacontane-d74, Sigma-Aldrich) in a sonicated water bath (EMAG ultrasonic cleaner Emmi 12 HC, 30 min). Extracts were centrifuged (2,000 r.p.m., 2 min), and the supernatants were dried under nitrogen gas for 15 min. The dried residues were derivatized using a 1-(trimethylsilyl)imidazole–pyridine mixture (92718, Sigma-Aldrich) at 75 °C for 30 min. Next, samples were transferred to 150-µl vial inserts and analyzed by GC–MS using a 14-min gradient as described in earlier sections. The standards cholesterol (Sigma-Aldrich), lathosterol (Sigma-Aldrich) and **11** (this study) were also derivatized in a similar manner with 10 µg ml^−1^ internal standard and used in the study.

### Sterol quantification

A series of different concentrations of cholesterol, lathosterol and **11** were prepared in *n*-hexane from a 1 mg ml^−1^ stock. Ion *m*/*z* 66 was used as a quantifier for the internal standard. Ions *m*/*z* 129, 459 and 457 were used as quantifiers for cholesterol, lathosterol and **11** sterols, respectively. Calibration curves of all sterol standards had a regression coefficient of *R*^2^ > 0.99. Qualifiers were automatically selected by MS Quantitative Analysis software (Agilent). Automated data generated by MS Quantitative Analysis software were confirmed by manual inspection of peak mass spectra and retention times. Calibration curves and quantification was performed using MS Quantitative Analysis software as implemented in MassHunter Chemstation (version B.07.00) (Agilent).

### *A. japonicus LDS* and *A. japonicus PS* RNA-seq expression analysis

*A. japonicus* adult organs (intestine, tentacle, muscle, body wall, male gonads, female gonads and tube feet), embryonic-stage samples (zygote, cleavages, blastulae and gastrulae), larvae (auricularia, doliolaria and pentactula) and juvenile samples were used for RNA-seq library construction. Total mRNA for each tissue type was extracted following the protocol described earlier^51^. RNA-seq libraries were constructed using the NEBNext mRNA Library Prep kit (NEB), following the manufacturer’s instructions. The prepared libraries were subjected to paired-end 100-bp sequencing using the Illumina HiSeq 2000 platform. Sequencing reads were first filtered by removing those containing undetermined bases (‘N’) or excessive numbers of low-quality positions and then aligned to the *A. japonicus* genome using STAR aligner with its default parameters^52^. Raw counts of aligned reads per gene were obtained using HTSeq (version 0.6.1)^53^. Gene expression levels were represented in the form of RPKM.

### Quantitative reverse transcription analysis of *LDS* and *PS* transcripts

For OSC expression (*P. parvimensis LDS* and *P. parvimensis PS*) analysis, total RNA was extracted from different adult tissues and early developmental-stage samples of *P. parvimensis* using TRI Reagent (T9424, Sigma-Aldrich). RNA samples were treated with DNase I (Roche). First-strand cDNA synthesis was carried out using the SuperScript II Reverse Transcriptase kit according to the manufacturer’s instructions (Invitrogen). Primers used for quantitative reverse transcription analysis are listed in Supplementary Table 9.

### Whole-mount mRNA in situ analysis

#### Synthesis of *P. parvimensis LDS* and *P. parvimensis PS* probes

RNA was isolated from 2-dpf and 4-dpf larvae of *P. parvimensis* using the Total Mammalian RNA Miniprep kit (Sigma-Aldrich) and converted to first-strand cDNA with the iScript Select cDNA Synthesis kit (Bio-Rad). Primers were designed against the *P. parvimensis* sea cucumber OSC genes *LDS* and *PS* such that the PCR products were 500–1,000 nucleotides long and the reverse primers had T7 or SP6 tails. The cDNA from the desired stages were used as a template for PCR. Antisense digoxigenin (DIG)-labeled RNA probes were synthesized from PCR products specific to *P. parvimensis LDS* or *P. parvimensis PS* using the DIG RNA Labeling kit (Roche). The primers used are described in Supplementary Table 9.

#### Whole-mount in situ hybridization

Whole-mount in situ hybridization was performed as described earlier with the following modifications^54^. Pre-hybridization, hybridization and post-hybridization washes were performed at 58 °C. Optimal probe concentrations were determined experimentally (~0.2 ng μl^−1^). After blocking, embryos were incubated overnight at 4 °C in 2% blocking reagent (Roche) in malic acid buffer with the addition of a 1:2,000 dilution of anti-DIG alkaline phosphatase-conjugated antibody (Roche). Embryos were mounted in antifade reagent in glycerol–PBS from the SlowFade Antifade kit (Invitrogen) and imaged using a Leica DFC420 C camera on a Leica DMI4000 B microscope.

### Homology modeling

Using human LSS as a template (Protein Data Bank 1W6K)^28^, homology models of *P. miniata* LSS, *S. purpuratus* LSS, *P. parvimensis* LDS and *P. parvimensis* PS were developed to identify variations in the active site that govern product specificities of these OSCs. Homology models were generated using the Phyre2 (version 2.0)^55^ and I-TASSER (version 5.0)^56^ servers with default parameters. The models obtained were subjected to stereochemical validation using tools embedded in Chimera (version 1.15)^57^ and visualized using Chimera as well as PyMOL (version 2.0). Protein sequences were aligned using ClustalW (version 2.1), and active site residues were manually annotated using functional information available for human LSS^28^.

### Site-directed mutagenesis

Site-directed mutagenesis was carried out to establish whether residue position 444 (F, Q or L) determines product specificity in sea cucumber OSCs. The primer-design strategy and PCR conditions used were as described earlier^58^. Transfer-PCR (TPCR) was used in site-directed mutagenesis reactions^59^. Briefly, TPCR conditions include initial denaturation (95 °C, 1 min); 13 cycles of 95 °C for 30 s, 60 °C for 1 min and 72 °C for 1.5 min; and then 20 cycles of 95 °C for 30 s, 67 °C for 1 min and 72 °C for 4 min. Reactions were completed with a final elongation step of 7 min at 72 °C. PCR components included 5–10 ng pDONOR or pYES2 plasmid DNA, 20 nM mutagenic primers, 250 μM of each dNTP, 1× Phusion buffer and 1 U Phusion DNA polymerase (NEB). At the end of the TPCR reaction, 1 μl DpnI was added to a 10-μl reaction and incubated at 37 °C for 1–2 h. An aliquot (5 μl) of reaction mixture was transformed into *E. coli* (DH5α, Invitrogen), and positive transformants were verified through sequencing. Primers used for mutagenesis are listed in Supplementary Table 9.

### Reporting summary

Further information on research design is available in the Nature Research Reporting Summary linked to this article.

## Online content

Any methods, additional references, Nature Research reporting summaries, source data, extended data, supplementary information, acknowledgements, peer review information; details of author contributions and competing interests; and statements of data and code availability are available at 10.1038/s41589-022-01054-y.

## Supplementary information

Supplementary Information Supplementary Figs. 1–6, Tables 1–9 and Notes 1–3

Reporting Summary

## Data Availability

Data supporting the findings of this work are available within the paper and its Supplementary Information. The datasets, constructs and chemical standards generated and analyzed during the current study are available from the corresponding author upon request. Sea cucumber material is available from co-authors V.H. (Carnegie Mellon University, email veronica@cmu.edu) and S.W. (Ocean University of China, email swang@ouc.edu.cn). The sequences of genes characterized in this project will be deposited in the European Nucleotide Archive, and accession numbers will be shared before publication. Source data are provided with this paper. Sea urchin, sea star and sea cucumber OSC sequences have been deposited in GenBank under the following ids ON478348-ON478355.

## Source data

Source Data Fig. 2 Fig. 2a, top, yeast growth inhibition (OD_600_) of adult tissue crude extracts. Fig. 2a, bottom, RNA-seq FPKM expression values of *LDS* and *PS* OSC genes.
Source Data Extended Data Fig. 3 Yeast growth inhibition (OD_600_) of crude extract and saponin fractions.
Source Data Extended Data Fig. 5 Yeast growth inhibition (OD_600_) of early-stage extracts of *P. parvimensis* and *A. japonicus* sea cucumbers.
Source Data Extended Data Fig. 6 Quantification of sterols in *A. japonicus* adult tissue extracts.
