# Supplementary material for: Biosynthesis of saponin defensive compounds in sea cucumbers
Source: Nat Chem Biol. 2022 Jun 27;18(7):774–81. doi: 10.1038/s41589-022-01054-y (PMC9236903; doi:10.1038/s41589-022-01054-y)
Supplement: Supplementary file 1 — Supplementary Figs. 1–6, Tables 1–9 and Notes 1–3 [file 41589_2022_1054_MOESM1_ESM.pdf]

nature portfolio

https://doi.org/10.1038/s41589-022-01054-y

Supplementary information

Biosynthesis of saponin defensive compounds in sea cucumbers

In the format provided by the authors and unedited

Supplementary information

Biosynthesis of saponin defensive compounds in sea cucumbers

Ramesha Thimmappa[1#], Shi Wang[2ǂ], Minyan Zheng[3ǂ], Rajesh Chandra Misra[1], Ancheng C. Huang[1$], Gerhard Saalbach[1], Yaqing Chang[4], Zunchun Zhou[5], Veronica Hinman[3], Zhenmin Bao[2], Anne Osbourn[1*]

Supplementary Figures

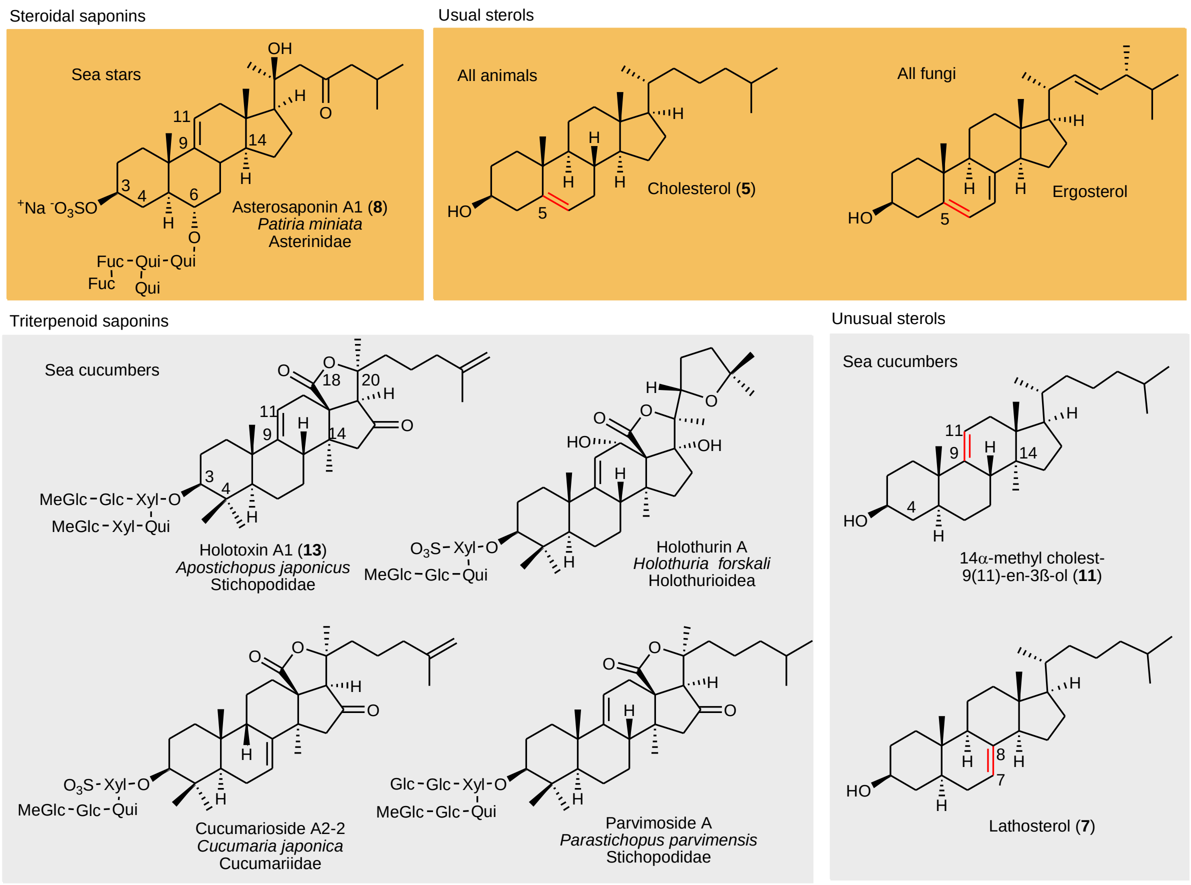

Supplementary Fig. 1: Some examples of sea star and sea cucumber saponins and sterols. Sea star saponins are steroidal and sea cucumber saponins are triterpenoid. Diverse saponins are shown with variations in the aglycone, sugar chain, sulfation and δ18-20 lactone group. All domains of life except bacteria make sterols characterized by a common C5 unsaturation and absence of methyl groups at carbon positions 4 and 14 (called usual sterols). Sea stars and sea cucumbers make uncommon sterols with C7 and C9(11) unsaturation (called unusual sterols).

a

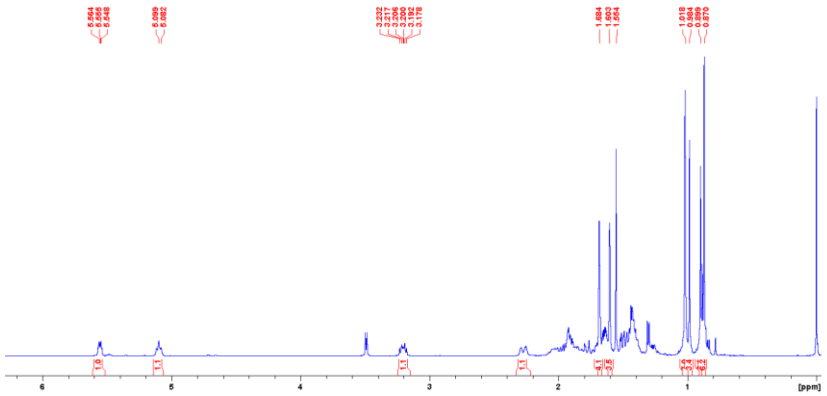

^{1}H NMR of lanostadienol (**4**)

b

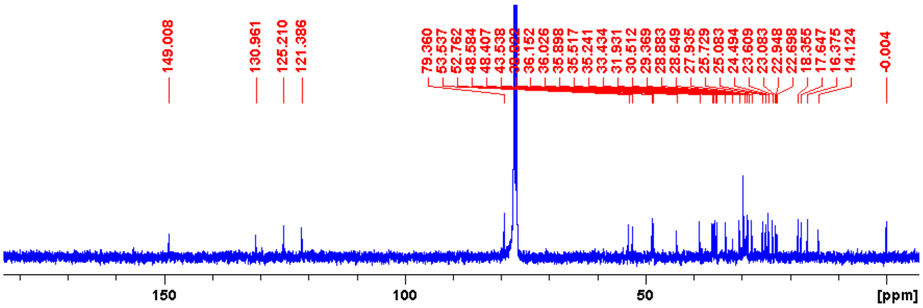

^{13}C NMR of lanostadienol (**4**)

c

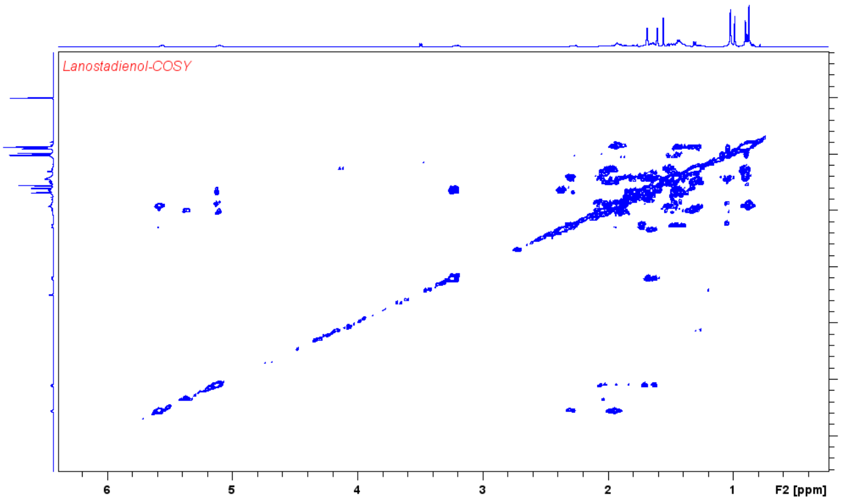

COSY of lanostadienol (**4**)

d

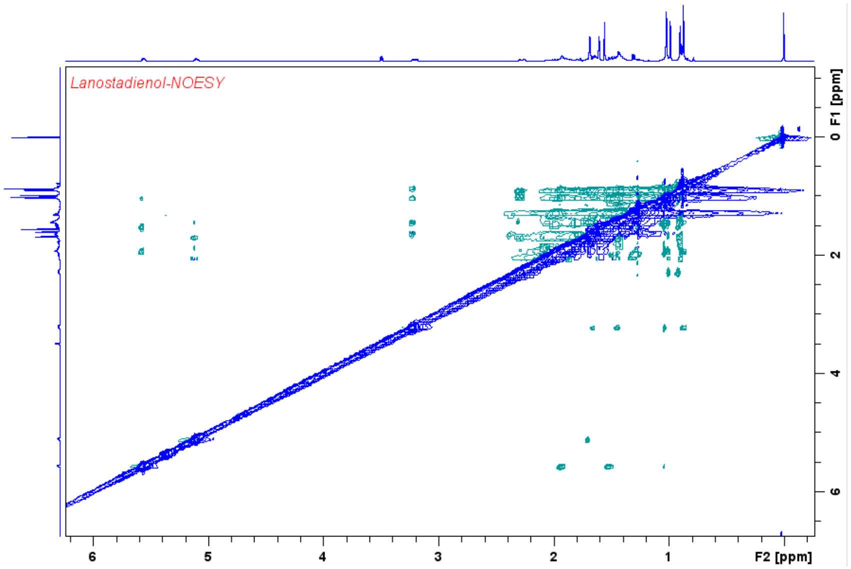

NOESY of lanostadienol (**4**)

Supplementary Fig. 2: NMR characterization of the LDS product. a, ^{1}H NMR spectrum of the LDS product, lanostadienol (**4**). **b**, ^{13}C NMR spectrum of lanostadienol (**4**) (see Supplementary Table 3). **c,** COSY of lanostadienol (**4**). **d**, NOESY of lanostadienol (**4**).

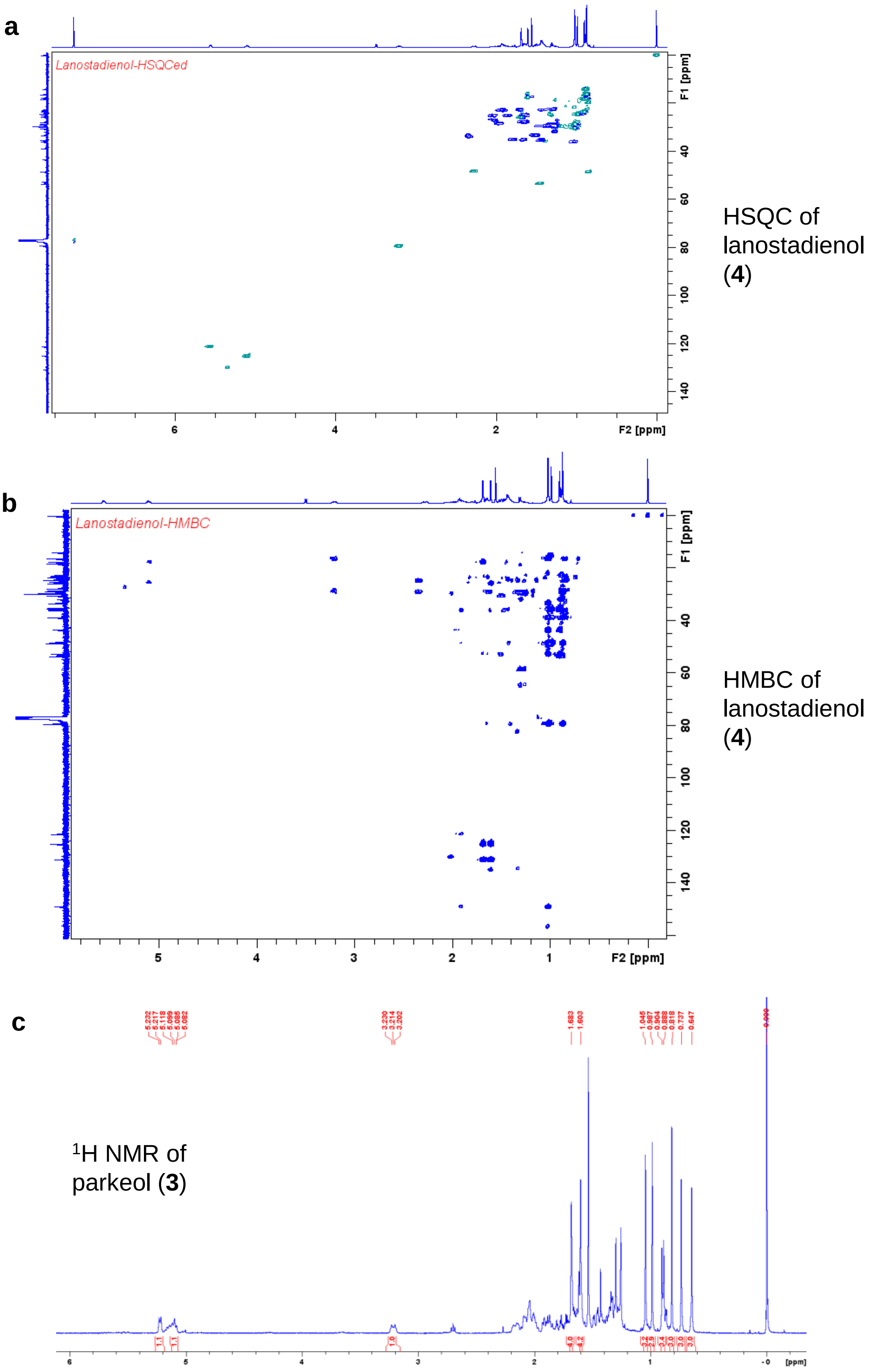

Supplementary Fig. 3: NMR characterization of the LDS and PS products. a, HSQC of lanostadienol (**4**). **b**, HMBC of lanostadineol (**4**). **c**, ^{1}H NMR spectrum of parkeol (**3**) (see Supplementary Table 4).

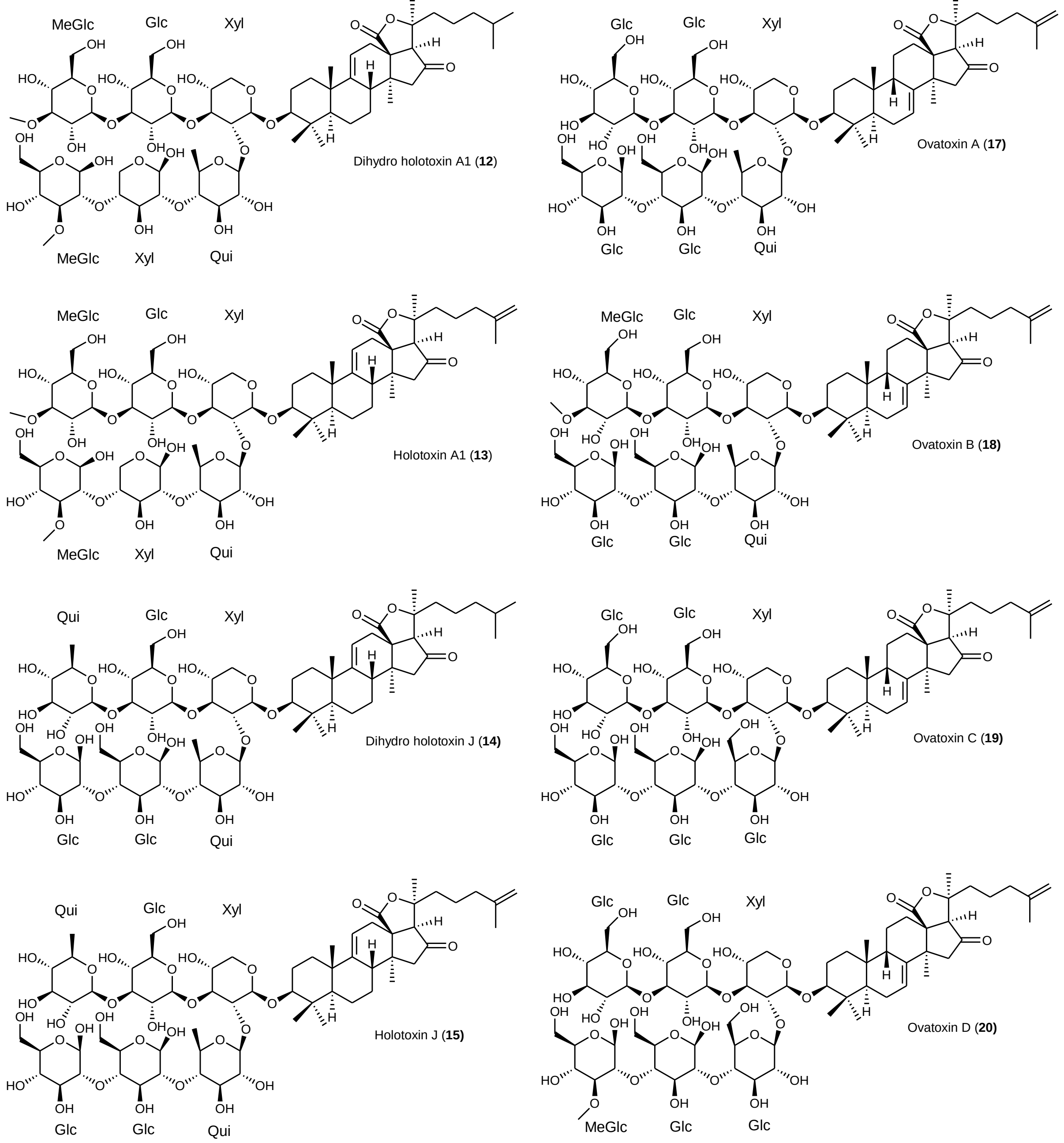

Supplementary Fig. 4: Sea cucumber saponins observed in the current study.

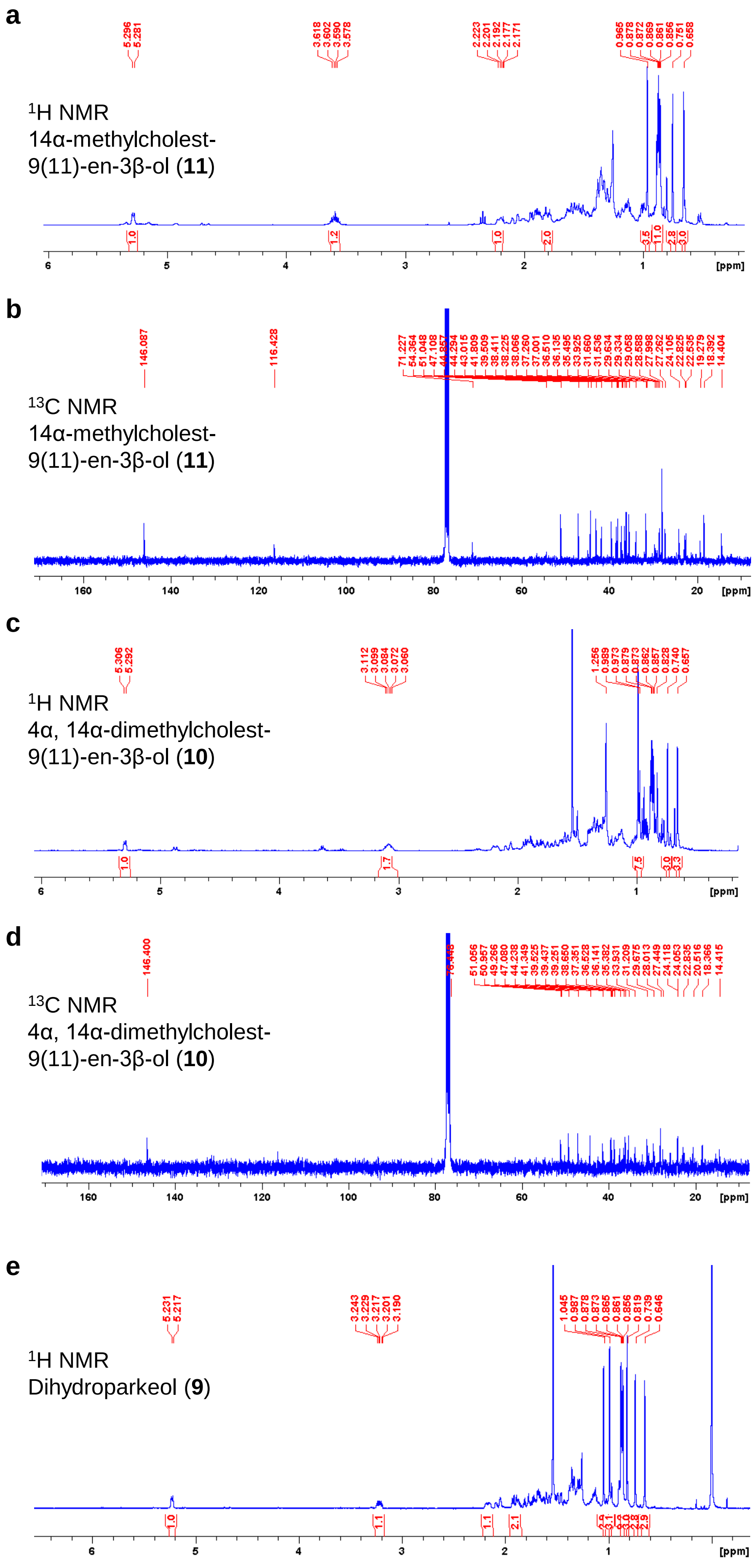
a
^{1}H NMR
14α-methylcholest-
9(11)-en-3β-ol (**11**)
[ppm]
b
^{13}C NMR
14α-methylcholest-
9(11)-en-3β-ol (**11**)
[ppm]
c
^{1}H NMR
4α, 14α-dimethylcholest-
9(11)-en-3β-ol (**10**)
[ppm]
d
^{13}C NMR
4α, 14α-dimethylcholest-
9(11)-en-3β-ol (**10**)
[ppm]
e
^{1}H NMR
Dihydroparkeol (**9**)
[ppm]

Supplementary Fig. 5: NMR characterization of unusual sterols from sea cucumbers. a, ^{1}H NMR spectrum of 14α-methylcholest-9(11)-en-3β-ol (**11**). **b**, ^{13}C NMR spectrum of **11** (see Supplementary Table 5)**.** **c**, ^{1}H NMR spectrum of 4α, 14α-dimethylcholest-9(11)-en-3β-ol (**10**). **d**, ^{13}C NMR spectrum of **10** (see Supplementary Table 6). **e**, ^{1}H NMR spectrum of dihydroparkeol (**9**) (see Supplementary Table 7).

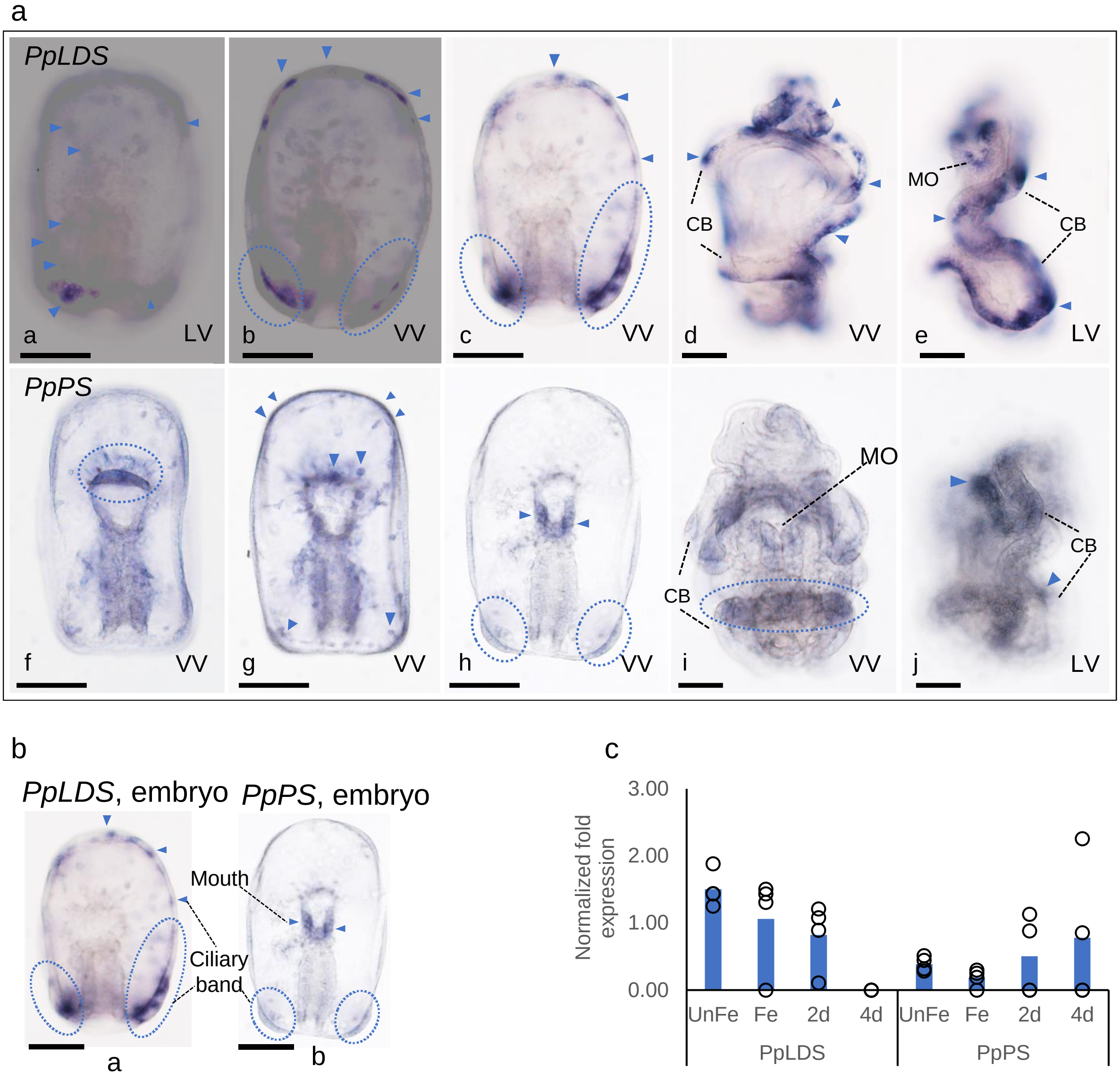

Supplementary Fig. 6: Expression of *PpLDS* and *PpPS* OSC genes in sea cucumbers. a, Whole mount *in situ* mRNA hybridization of *PpLDS* and *PpPS*. a-c, 2-day larva, expression of *PpLDS* on the ectoderm and ciliary band domain is shown by the arrows and dashed circles. d-e, 4-day larva, expression of *PpLDS* is observed at the mouth and along the ciliary bands. f-h, 2-day larva, *PpPS* is expressed in the ectoderm, along the presumptive mouth and foregut, and some mesodermal cells. i-j, 4-day larva, expression of *PpPS* is observed at the mouth and along the ciliary bands. All larvae were oriented with anterior ends facing upwards and posterior ends downwards. CB, ciliary bands; MO, mouth; VV, ventral view; LV, lateral view. **b**, mRNA *in situ* localization of *LDS* and *PS* expression in *P. parvimensis* embryos. mRNA expression is marked by arrows and dashed circles. **c**, Actin- normalized expression of *PpLDS* and *PpPS* in unfertilized embryos (UnFe), fertilized embryos (Fe), 2-day old embryo (2d) and 4-day old embryo (4d). Normalized fold expression (mean, n = 2).

Supplementary Tables

Supplementary Table 1: Contigs used in assembly of full-length OSCs. Contig sequences are given in supplementary notes 1.

Taxa	Species	Contig Id	GenBank Id	OSC	Nucleotides (bp)	Protonation motif	Source
Sea stars	*Echinaster spinulosus*	AMR0024.75465.0.8	gb|GAVE01093487.1	EsLSS	3517	DCTAE	Echniobase
		AMR0024.75465.0.5	gb|GAVE01093484.1		3542		Echniobase
		AMR0024.75465.0.4	gb|GAVE01093483.1		3465		Echniobase
	Henricia sp.	AMR0023.73778.3.31	gb|GAVP01109444.1	HspLSS	3479	DCTAE	Echniobase
		AMR0023.73778.3.29	gb|GAVP01109442.1		3462		Echniobase
		AMR0023.73778.3.27	gb|GAVP01109440.1		2723		Echniobase
	Asterias forbesi	AMR9007.32030.7.1	gb|GAUS01043709.1	AfLSS	2756	DCTAE	Echniobase
	Asterias rubens	AMR0022.38627.0.1	gb|GAUU01031164.1	ArLSS	2469	DCTAE	Echniobase
	Leptasterias sp.	AMR0026.43782.2.3	gb|GAVC01057989.1	LspLSS	2508	DCTAE	Echniobase
		AMR0026.43782.2.6	gb|GAVC01057992.1		2411		Echniobase
		AMR0026.43782.2.7	gb|GAVC01057993.1		1483		Echniobase
	Patiria miniata	AMR9002.36632.0.1	gb|GAWB01045303.1	PmLSS	1800	DCTAE	Echniobase
	Patiria pectinifera	AMR0034.73258.0.2	gb|GAVK01073940.1	PmLSS	1838	DCTAE	Echniobase
	Marthasterias glacialis	AMR0029.77368.0.1	gb|GAVI01039758.1	MgLSS	2023	DCTAE	Echniobase
	Asterias amurensis	AMR0036.44053.0.2	gb|GAVJ01039023.1	AaLSS	1381	DCTAE	Echniobase
		AMR0036.44053.0.3	gb|GAVJ01039024.1		1375		Echniobase
		AMR0036.44053.0.1	gb|GAVJ01039022.1		1264		Echniobase
	Acanthaster plancii	oki.2.251.t1		ApLSS COTS	2121	DCTAE	marinegenomics.oist.jp/cots
Sand dollar	*Echinarachnius parma*	AMR0025.59677.1.23	gb|GAVF01081824.1	EpLSS	4265	DCTAE	Echniobase
		AMR0025.59677.1.19	gb|GAVF01081820.1		2934		Echniobase
		AMR0025.59677.1.18	gb|GAVF01081819.1		4466		Echniobase
		AMR0025.59677.1.17	gb|GAVF01081818.1		3135		Echniobase
Sea urchins	*Sphaerechinus granularis*	AMR0032.60851.0.6	gb|GAVR01073116.1	SgLSS	1597	DCTAE	Echniobase
		AMR0032.60851.0.1	gb|GAVR01073111.1		1551		Echniobase
		AMR0032.60851.0.8	gb|GAVR01073118.1		1490		Echniobase
	Strongylocentrotus purpuratus	WHL22.494761.2		SpLSS	4272	DCTAE	Echniobase
		WHL22.494761.3			1632		Echniobase
Sea cucumbers	*Parastichopus parvimensis*	Locus_10303_Transcript_8/65		PpOSC1	3281	DTTAE	Echniobase
		Locus_10303_Transcript_47/65			2623		Echniobase
		Locus_2545_Transcript_27/30		PpOSC2	3258	DTSAE	Echniobase
		Locus_2545_Transcript_7/30			2630		Echniobase
	Apostichopus japonicus	AMR0033.53287.0.1	gb|GAVS01052291.1	AjOSC1	1748	DTTAE	Echniobase
		AMR0033.53287.0.5	gb|GAVS01052295.1		1073		Echniobase
		AMR0033.53287.0.7	gb|GAVS01052297.1	AjOSC2	1214	DTSAE	Echniobase
		AMR0033.53287.0.4	gb|GAVS01052294.1		2558		Echniobase
		AMR0033.53287.0.2	gb|GAVS01052292.1		1159		Echniobase
		AMR0033.53287.0.6	gb|GAVS01052296.1		890		Echniobase
	Stichopus chloronotus	1621490		ScOSC1	2225	DTTAE	EchinoDB
		1593798			2667		EchinoDB
		1619393			2111		EchinoDB
		1579729		ScOSC2	2217	DTSAE	EchinoDB
		3222879			2659		EchinoDB
		1613179			2488		EchinoDB
Bacteria	*Gemmata obscuriglobus*	gcontig_1106221709355	WP_010036461.1	GoOSC	1953	DCTAE	NCBI Reference Sequence

Supplementary Table 2: OSC sequences used in the phylogenetic tree shown in Extended Data Fig. 1.

Taxa	Species	Abbreviation	Common name	Taxonomic group	G/T	OSC length	OSC	Sequence id
Outgroups	*Capsaspora owczarzaki*	Capsaspora	Single cell eukaryote	Opisthokonta	G	Full	Capsaspora LSS	XP_004364346.1
	Danio rerio	Dre	Zebrafish	Chordata	G	Full	DreLSS	NP_001077036.1
	Branchiostoma floridae	Branchiostoma	Lancelet	Cephalochordata	G	Full	Branchiostom aLSS	XP_002606162.1
	Homo sapiens	Human	Human	Chordata	G	Full	HumanLSS	AJ239031
	Amphimedon queenslandica	Amph	Sponge	Porifera	G	Full	Amphimedon LSS	XP_003383177.1
	Saccoglossus kowalevskii	Saccoglossus	Acorn worm	Hemichordata	G	Full	Saccoglossus LSS	XP_006825099.1
	Helobdella robusta	Helobdella	Leech	Annelida	G	Full	Helobdella LSS	XP_009020449.1
Sea stars	*Asterias amurensis*	Aa	Japanese sea star	Echinodermata	T	Full	AaLSS	this study
	Marthasterias glacialis	Mg	Spiny star fish	Echinodermata	T	Full	MgLSS	
	Asterias forbesi	Af	Sea star	Echinodermata	T	Full	AfLSS	
	Asterias rubens	Ar	Sea star	Echinodermata	T	Full	ArLSS	
	Leptasterias sp.	Lsp	Brooding sea star	Echinodermata	T	Partial	LspLSS	
	Patiria miniata	Pm	Sea star	Echinodermata	G & T	Full	PmLSS	
	Patiria pectinifera	Pp	Sea star	Echinodermata	T	Partial	PpLSS	
	Henricia sp.	Hsp	Blood sea star	Echinodermata	T	Full	HspLSS	
	Echinaster spinulosus	Es	Spine sea star	Echinodermata	T	Full	EsLSS	
	Acanthaster plancii	Ap	Crown of thorns star	Echinodermata	G & T	Full	ApLSS	
Sand dollar	*Echinarachnius parma*	Ep	Sand dollar	Echinodermata	T	Full	EpLSS	this study
Sea urchins	*Sphaerechinus granularis*	Sg	Purple sea urchin	Echinodermata	T	Partial	SgLSS	this study
	Strongylocentrotus purpuratus	Spu	Common sea urchin	Echinodermata	G & T	Full	SpLSS	this study
Sea cucucumbers	*Parastichopus parvimensis*	Pp	Warty sea cucumber	Echinodermata	G & T	Full	PpOSC1	this study
						Full	PpOSC2	
	Apostichopus japonicus	Aj	Japanese sea cucumber	Echinodermata	G & T	Full	AjOSC1	this study
						Full	AjOSC2	
	Stichopus chloronotus	Sc	Greenfish sea cucucumber	Echinodermata	T	Partial	ScOSC1	this study
						Full	ScOSC2	

Note: G = genome, T = transcriptome.

Supplementary Table 3: ^1H (400 MHz) and ^{13}C (100 MHz) NMR (in $CDCl_3$) data for lanostadienol (**4**) and its acetate form.

	**δC (ppm)**			**δH (ppm) (multiplicity, *J* in Hz, number of H)**	
Position	Acetate (Literatur[41])	**4**	Position	Acetate	**4**
C1	35.18	35.24	H1	1.518 (m, 1H);<br>1.439 (m, 1H)	1.793 (m, 1H);<br>1.661 (m, 1H)
C2	24.32	27.94	H2	1.661 (m, 1H);<br>1.661(m, 1H)	1.645 (m, 2H)
C3	81.13	79.36	H3	4.466 (m, 1H)	3.205 (m, 1H)
C4	37.69	38.82	H4		
C5	48.58	48.58	H5	0.945 (m, 1H)	0.842 (m, 1H)
C6	22.92	23.08	H6	1.911 (m, 1H);<br>1.910 (m, 1H)	1.924 (m, 2H)
C7	121.12	121.38	H7	5.544 (m, 1H)	5.555 (m, 1H)
C8	149.09	149	H8		
C9	48.4	48.41	H9	2.283 (br d, 1H)	2.72 (br d, 13.6, 1H)
C10	35.73	35.9	H10		
C11	22.92	22.95	H11	1.421 (m, 1H);<br>1.704 (m, 1H)	1.424 (m, 1H);<br>1.694 (m, 1H)
C12	35.2	35.52	H12	1.799 (br dd, 13, 10, 1H);<br>1.657 (br ddd, 13, 10, 8, 1H)	1.432 (m, 2H)
C13	43.5	43.54	H13		
C14	52.76	52.76	H14		
C15	33.39	33.43	H15	1.513 (m, 1H);<br>1.478 (m, 1H)	1.505 (m, 2H)
C16	28.62	28.65	H16	1.958 (m, 1H);<br>1.266 (dddd, 13.5, 10.2, 8.0, 3.0, 1H)	1.967 (m, 1H);<br>1.266 (m, 1H)
C17	53.5	53.54	H17	1.465 (br q, 9.6, 1H)	1.465 (m, 1H)
C18	23.61	23.61	H18	0.898 (s, 3H)	0.899 (s, 3H)
C19	24.48	24.49	H19	1.007 (d, 0.6, 3H)	1.018 (s, 3H)
C20	36	36.03	H20	1.393 (m, 1H)	1.393 (m, 1H)
C21	18.32	18.36	H21	0.877 (d, 6.5, 3H)	0.879 (d, 6.4, 3H)
C22	36.12	36.15	H22	1.427 (dddd, 13.3, 10.0, 6.5, 2.5, 1H);<br>1.026 (dddd, 13.3, 11, 9, 4.8, 1H)	1.031(m, 2H)
C23	25.05	25.08	H23	1.859 (m, 1H);<br>2.037 (m, 1H)	1.861 (m, 1H);<br>2.055 (m, 1H)
C24	125.19	125.21	H24	5.099 (ddseptet, 7.3, 6.9, 1.4, 1H)	5.099 (br dd, 7.6, 6.8, 1H)
C25	130.93	130.96	H25		
C26	25.71	25.73	H26	1.684 (qq, 1.3, 0.4, 3H)	1.684 (br s, 3H)
C27	17.63	17.65	H27	1.604 (qq, 1.3, 0.4, 3H)	1.607 (s, 3H)
C28	28.75	28.88	H28	0.898 (s, 3H)	0.870 (s, 3H)
C29	17.48	16.38	H29	0.941 (s, 3H)	0.984 (s, 3H)
C30	30.53	30.51	H30	1.016 (d, 1.0, 3H)	1.018 (s, 3H)

Supplementary Table 4: ^{1}H (400 MHz) NMR (in $CDCl_3$) data for parkeol (**3**).

	Parkeol (3)	
	**δH (ppm) (number of H, multiplicity, *J* in Hz)**	
Position	Literature[41]	This work
H30	0.65 (3H, s)	0.65 (3H, s)
H18	0.74 (3H, s)	0.74 (3H, s)
H29	0.82 (3H, s)	0.82 (3H, s)
H21	0.9 (3H, d)	0.9 (3H, d, 6.4)
H28	0.99 (3H, s)	0.99 (3H, s)
H19	1.04 (3H, s)	1.04 (3H, s)
H26	1.60 (3H, s)	1.60 (3H, s)
H27	1.68 (3H, s)	1.68 (3H, s)
H3	3.22 (1H, dd)	3.22 (1H, m)
H24	5.09 (1H, t)	5.10 (1H, m)
H11	5.22 (1H, d)	5.23 (1H, d, 6.0)

Supplementary Table 5: ^{13}C (100 MHz) NMR (in $CDCl_3$) data for 14α-methylcholest-9(11)-en-3β-ol (**11**) and its acetate form.

	**δC (ppm)**	
Position	Acetate of **11** (Literature[48])	**(11)**
C1	35.3	35.5
C2	27.24	27.26
C3	73.6	71.23
C4	34.29	38.07
C5	42.88	44.29
C6	28.54	28.59
C7	28.03	28
C8	41.87	41.81
C9	145.85	146.09
C10	38.07	38.41
C11	116.63	116.43
C12	37.34	37.26
C13	44.35	44.86
C14	47.16	47.11
C15	33.97	33.93
C16	27.64	31.66
C17	51.11	51.05
C18	14.45	14.4
C19	19.21	19.28
C20	36.19	36.14
C21	18.43	18.39
C22	36.57	36.51
C23	24.15	24.11
C24	39.56	39.51
C25	28.03	28
C26	22.84	22.83
C27	22.56	22.54
C28		
C29	18.43	18.39

Supplementary Table 6: ^{13}C (100 MHz) NMR (in $CDCl_3$) data for 4α, 14α-dimethylcholest-9(11)-en-3β-ol (**10**) and its acetate form from literature[48].

	**δC (ppm)**	
Position	**10** (Literature[48])	**10** this work
C1	35.52	35.38
C2	31.34	31.21
C3	76.53	76.45
C4	39.58	39.44
C5	49.46	49.29
C6	24.17	24.12
C7	28.09	27.45
C8	41.52	41.35
C9	146.57	146.4
C10	38.76	38.65
C11	116.39	116.4
C12	37.52	37.35
C13	44.39	44.24
C14	47.2	47.08
C15	34.03	33.93
C16	27.54	27.45
C17	51.21	51.06
C18	14.5	14.42
C19	20.57	20.52
C20	36.21	36.14
C21	18.46	18.37
C22	36.65	36.53
C23	24.17	24.05
C24	39.63	39.53
C25	28.09	28.01
C26	22.83	22.84
C27	22.57	22.57
C28	15.34	15.31
C29	18.46	18.37

Supplementary Table 7: ^{1}H (400 MHz) NMR data for dihydroparkeol (**9**).

	Parkeol (**3**)		Dihydroparkeol (**9**)
Position	Literature[41]	This work	This work
H30	0.65 (3H, s)	0.65 (3H, s)	0.65 (3H, s)
H18	0.74 (3H, s)	0.74 (3H, s)	0.74 (3H, s)
H29	0.82 (3H, s)	0.82 (3H, s)	0.82 (3H, s)
H21	0.9 (3H, d)	0.9 (3H, d, 6.4)	0.863 (3H, d, 6.4)
H28	0.99 (3H, s)	0.99 (3H, s)	0.99 (3H, s)
H19	1.04 (3H, s)	1.04 (3H, s)	1.04 (3H, s)
H26	1.60 (3H, s)	1.60 (3H, s)	0.870 (3H, d, 6.4)
H27	1.68 (3H, s)	1.68 (3H, s)	0.874 (3H, d, 6.4)
H3	3.22 (1H, dd)	3.22 (1H, m)	3.22 (1H, m)
H24	5.09 (1H, t)	5.10 (1H, m)	
H11	5.22 (1H, d)	5.23 (1H, d, 6.0)	5.23 (1H, d, 6.0)

Supplementary Table 8: Pairwise percent amino acid sequence identity of cholesterol (**5**) pathway genes from humans, sea urchins, sea stars and sea cucumbers.

	HsCYP51	SpCYP51	PmCYP51	Pp CYP51 hit	Aj CYP51 hit
HsCYP51	100				
SpCYP51	68	100			
PmCYP51	63	67	100		
Pp CYP51 hit	**22**	**20**	**19**	100	
Aj CYP51 hit	**22**	**22**	**22**	57	100

	HsLbr	SpLbr	PmLbr	PpLbr	AjLbr
HsLbr	100				
SpLbr	45	100			
PmLbr	44	54	100		
PpLbr	44	53	53	100	
AjLbr	37	46	49	87	100

	HsDHCR24	SpDHCR24	PmDHCR24	PpDHCR24	AjDHCR24-1
HsDHCR24	100				
SpDHCR24	64	100			
PmDHCR24	67	68	100		
PpDHCR24	58	63	59	100	
AjDHCR24-1	57	60	59	88	100

	HsC4mso	Spc4mso	PmC4mso	PpC4mso	AjC4mso
HsC4mso	100				
Spc4mso	56	100			
PmC4mso	59	59	100		
PpC4mso	55	53	56	100	
AjC4mso	59	65	65	100	100

	HsNSDHL	SpNSDHL	PmNSDHL	PpNSDHL	AjNSDHL
HsNSDHL	100				
SpNSDHL	63	100			
PmNSDHL	67	66	100		
PpNSDHL	57	57	58	100	
AjNSDHL	57	57	58	98	100

	HsHSD17B7	SpHSD17B7	PmHSD17B7	PpHSD17B7-1	AjHSD17B7
HsHSD17B7	100				
SpHSD17B7	49	100			
PmHSD17B7	40	46	100		
PpHSD17B7-	44	45	46	100	
AjHSD17B7	44	45	47	93	100

	HsEbp	SpEbp	PmEbp	PpEbp	AjEbp
HsEbp	100				
SpEbp	42	100			
PmEbp	37	41	100		
PpEbp	32	34	28	100	
AjEbp	35	35	29	95	100

	HsSC5d	SpSc5d	PmSc5d	PpSc5d	AjSc5d
HsSC5d	100				
SpSc5d	59	100			
PmSc5d	59	60	100		
PpSc5d	56	57	55	100	
AjSc5d	56	58	55	97	100

	HsDHCR7	SpDHCR7	PmDHCR7	Aj DHCR7 hit	Pp DHCR7 hit
HsDHCR7	100				
SpDHCR7	55	100			
PmDHCR7	59	60	100		
Aj DHCR7 hit	**24**	**25**	**25**	100	
Pp DHCR7 hit	**31**	**32**	**30**	87	100

	HsC7D hit	SpC7D	PmC7D	PpC7D	AjC7D
HsC7D hit	100				
SpC7D	**19**	100			
PmC7D	**18**	49	100		
PpC7D	**18**	46	54	100	
AjC7D	**19**	51	53	98	100

	HsSRD5A1	SpSRD5A1	PmSRD5A1	PpSRD5A1	AjSRD5A1
HsSRD5A1	100				
SpSRD5A1	43	100			
PmSRD5A1	48	53	100		
PpSRD5A1	45	49	57	100	
AjSRD5A1	39	44	49	97	100

Pairwise identities <32% are marked in red (CYP51 and DHCR7 hits from sea cucumbers and DAF36 hit in humans). This implies potential absence of those genes in the corresponding taxa. LSS, lanosterol synthase; CYP51, lanosterol 14α demethylase; Lbr, sterol C14 reductase; SC4MOL(C4mso), sterol C4 methyloxidase; Nsdhl, sterol C3 dehydrogenase; HSD17B7, sterol C3 ketoreductase; Ebp, delta (8), delta (7)-isomerase; Sc5dl, sterol C-5 desaturase; DHCR7, sterol C-7 reductase; DHCR24, sterol C24 reductase; PS, parkeol synthase; LDS, lanostadienol synthase; 5αR, 5α-sterol reductase and C7D (DAF36), cholesterol-7 desaturase. Human (Hs = *Homo sapiens*), Sea urchin (Sp = *Strongylocentrotus purpuratus*), Sea star (Pm = *Patiria miniata*), Sea cucumbers (Pp = *Parastichopus parvimensis*), (Aj = *Apostichopus japonicus*). Sequences used are provided in supplementary notes 3.

Supplementary Table 9: Primers used in the study.

Purpose	Name	Sequence (5'-3')
cDNA cloning	PmLSS-F	ATGAGCGGCCGACGCAACAGAGGAG
	PmLSS-R	CTACTGCACCAAGGAGGAAAGCACC
	PpLDS-F	ATGCAGTCCGATAGCGACCA
	PpLDS-R	CTAAAATACCAATTTAGCGAGCTG
	PpPS-F	ATGTCTGGGTCAAGGAGAATTAATC
	PpPS-R	TTATACATGGGGGATCTTTTCCCAG
gDNA cloning	GoPS-F	ATGCCGCACGATCCCACTGCCCGCG
	GoPS-R	TCAACTTTTCGCAGTTGTACCCGAA
Gap repair cloning	PpLDS-Gap.NT-F	ACTACTAGCAGCTGTAATACGACTCACTATAGGGAATATTATGCAGTCCGATAGCGACCA
	PpLDS-Gap-CT1.R	GAATGTAAGCGTGACATAACTAATTACATGATGCGGCCCTCTATTCCTTGGAAGACGTGTA
	PpPS-Gap.NT-F	ACTACTAGCAGCTGTAATACGACTCACTATAGGGAATATTATGTCTGGGTCAAGGAGAATTAATC
	PpPS-Gap-CT1.R	GAATGTAAGCGTGACATAACTAATTACATGATGCGGCCCTTTATACATGGGGGATCTTTTCCCAG
	GoPS-Gap.NT-F	ACTACTAGCAGCTGTAATACGACTCACTATAGGGAATATTATGCCGCACGATCCCACTGCCCGCG
	GoPS-Gap.CT.R	GAATGTAAGCGTGACATAACTAATTACATGATGCGGCCCTTCAACTTTTCGCAGTTGTACCCGAA
Gap repair cloning using gBlocks	SpLSS-Gap.NT-F	ACTACTAGCAGCTGTAATACGACTCACTATAGGGAATATTATGTCTGAAAAGAAGAATCGAGGAG
	SpLSS-middleR	CAAAGGCTGTATCCCAAAGCTGAGAGCCATTGGTTCCCTG
	SpLSS-middleF	CAGGGAACCAATGGCTCTCAGCTTTGGGATACAGCCTTTG
	SpLSS-Gap.CT.R	GAATGTAAGCGTGACATAACTAATTACATGATGCGGCCCTCTAGAGTTTTTCCCAGTCTGAGCTG
	AjLDS^{a}-Gap.NT-F	ACTACTAGCAGCTGTAATACGACTCACTATAGGGAATATTATGCAGTCCGATAATGACCACA
	AjLDS^{a}-middleR	ATAGCAGCAAAGGAAGTGTCCCAAACTTGATTACCGTTAGTGCCCTGCAC
	AjLDS^{a}-middleF	GTGCAGGGCACTAACGGTAATCAAGTTTGGGACACTTCCTTTGCTGCTAT
	AjLDS^{a}-Gap.CT.R	GAATGTAAGCGTGACATAACTAATTACATGATGCGGCCCTCTAAAATACCAATTTAGCAATCTGC
	AjPS^{a}-Gap.NT-F	ACTACTAGCAGCTGTAATACGACTCACTATAGGGAATATTATGCCTGGGTCGAGGAGAATTG
	AjPS^{a}-middleR	ATAGCCATCGCAGCGTACGAGACGTCCCATATCTGCGTTCCATTTGTACC
	AjPS^{a}-middleF	GGTACAAATGGAACGCAGATATGGGACGTCTCGTACGCTGCGATGGCTAT
	AjPS^{a}-Gap.CT.R	GAATGTAAGCGTGACATAACTAATTACATGATGCGGCCCTTTATACATGGGGGATCTTTTCCCA
	AjLDS^{b}-Gap.NT-F	ACTACTAGCAGCTGTAATACGACTCACTATAGGGAATATTATGCAGTCCGATAATGACCACA
	AjLDS^{b}-middleR	ATAGCAGCAAAGGAAGTGTCCCAAACTTGATTACCGTTAGTGCCCTGCAC
	AjLDS^{b}-middleF	GTGCAGGGCACTAACGGTAATCAAGTTTGGGACACTTCCTTTGCTGCTAT
	AjLDS^{b}-Gap.CT.R	GAATGTAAGCGTGACATAACTAATTACATGATGCGGCCCTCTAAAATACCAATTTAGCAATCTGC
	AjPS^{b}-Gap.NT-F	ACTACTAGCAGCTGTAATACGACTCACTATAGGGAATATTATGCCTGGGCTGAGGAGAATTG
	AjPS^{b}-middleR	ATAGCCATCGCAGCGTACGAGACGTCCCATATCTGCGTTCCGTTTGTACC
	AjPS^{b}-middleF	GGTACAAACGGAACGCAGATATGGGACGTCTCGTACGCTGCGATGGCTAT
	AjPS^{b}-Gap.CT.R	GAATGTAAGCGTGACATAACTAATTACATGATGCGGCCCTTTATACATGGGGGATCTTTTCCCA
Gateway cloning	PpLDS-GW-F	**GGGGACAAGTTTGTACAAAAAAGCAGGCT**TAATGCAGTCCGATAGCGACC
	PpLDS-GW-R	**GGGGACCACTTTGTACAAGAAAGCTGGGT**ACTAAAATACCAATTTAGCGA
	PpPS-GW-F	**GGGGACAAGTTTGTACAAAAAAGCAGGCT**TAATGTCTGGGTCAAGGAGAA
	PpPS-GW-R	**GGGGACCACTTTGTACAAGAAAGCTGGGT**ATTATACATGGGGGATCTTTT
	AjLDS^{a}-GW-F	**GGGGACAAGTTTGTACAAAAAAGCAGGCT**TAATGCAGTCCGATAATGACCA
	AjLDS^{a}-GW-R	**GGGGACCACTTTGTACAAGAAAGCTGGGT**ACTAAAATACCAATTTAGCAA
	AjPS^{a}-GW-F	**GGGGACAAGTTTGTACAAAAAAGCAGGCT**TAATGCCTGGGTCGAGGAGAAT
	AjPS^{a}-GW-R	**GGGGACCACTTTGTACAAGAAAGCTGGGT**ATTATACATGGGGGATCTTTTC
	SpLSS-Gw.F	**GGGGACAAGTTTGTACAAAAAAGCAGGCTA**TGTCTGAAAAGAAGAATCGAGG
	SpLSS-Gw.R	**GGGGACCACTTTGTACAAGAAAGCTGGGT**CTAGAGTTTTTCCCAGTCTGAGC
Mutagenesis	AjLDS^{a}-Q444F-F	CAAGGGAGGATGGGCTttcACAATGAGGGAGCACG
	AjLDS^{a}-R	CCTCGAAGGAACCGTCCGGTAACTGATTGGATC
	AjLDS^{a}-Q444L-F	CAAGGGAGGATGGGCTCtcACAATGAGGGAGCACG
	AjLDS^{a}-R	CCTCGAAGGAACCGTCCGGTAACTGATTGGATC
	AjPS^{a}-L436F-F	CAAAGGAGGCTGGCCTtTCACGACAAGAGATCACG
	AjPS^{a}-R	CCTCGAAGGAACCGTCCGGTAACTGATTGGATC
	AjPS^{a}-L436Q-F	CAAAGGAGGCTGGCCTCagACGACAAGAGATCACG
	AjPS^{a}-R	CCTCGAAGGAACCGTCCGGTAACTGATTGGATC
	SpLSS-F440Q-F	CAAGGGTGGTTATCCC**CAA**TCAACCAAAGACTGTGG
	SpLSS-F440Q-R	CCACAGTCTTTGGTTGA**TTG**GGGATAACCACCCTTG
	SpLSS-F440L-F	GGGTGGTTATCCCTT**A**TCAACCAAAGACTG
	SpLSS-F440L-R	CAGTCTTTGGTTGA**T**AAGGGATAACCACCC
mRNA *in situ* probes	PpLDS-F	ATCTGAGGAGAGTTCAGGCT
	PpLDS-R	TAATACGACTCACTATAGGGTCCGTGCTCTCTCATTGTCT
	PpPKS-F	TGATGGTACACTCGAAAGGG
	PpPKS-R	TAATACGACTCACTATAGGGCCATGGAGTAAAGCCAGCTA
qRT	PpLDS-qRT-F	CCCAGGAAACCATTTCTG
	PpLDS-qRT-R	CTAAAATACCAATTTAGCGAG
	GAPDH-qRT-F	AGTTGAAGGGTGTTTTGG
	GAPDH-qRT-R	TTAAGCCTTGGCAACGTG
	PpPS-qRT-F	GTGAGGTATCCTGATGAA
	PpPS-qRT-R	TTATACATGGGGGATCTT
Sequencing	PmLSS-middleF	TTCTTAGAAGCAGGAGCAGC
	PpLDS-middleF	GCGCAAGAAGATGAACTG
	PpPS-middleF	ATACTACGCTCCAGAGGATA
	AjLDS^{a}-middleF	ATGAACTGATAATGGACCT
	AjPS^{a}-middleF	AGGAATTATTTGTCGAGGA
	AjLDS^{b}-middleF	ATGAACTGATAATGGACCT
	AjPS^{b}-middleF	GAATGTCGTATCTCTACGGC
	SpLSS-middleF	GAGGCTGGCGGCGAGGGAGAGTTC
	GAL1-F	AATATACCTCTATACTTTAACGTC
	pYES2-R	GCGTGAATGTAAGCGTGAC

Supplementary notes

Supplementary Notes 1: OSC contigs used in full length transcript assembly.

```
>gi|637916804|gb|GAVE01093487.1|
TTCAATCAATGTTCATTTCAGAACAAATCTCACTTTATGAGAACATGCATGAATGTAGCAGCCTGCTGTACGATA
TCCACCACAAAAACTTTGAATGTCTCAATGCATTTTCAGCACATAATTACACCCATGCTGTAGGGACACCATTAA
TTGTGTGTGCAGACCGTAACGCAGCCATTTGACATCCGTCAAGCTAATCAAGGGCTTAGTTCCCAATTAATATAT
CATGGGTTTTATTTTTGTGAAATTAAGTCTTTTGTATTTGTCCACATGAAAGTTAATAAAATGTGTATAATACTG
TATTATAATTCCTGTGCAGTCCTTGTCTGACATGTATGTTCATTTTGCAAATTAACCATCAATAATTTTTAAACA
CGTTAATTGAAATCAGTCGGGCATTCCCACGATTATTGCACAAGTTTTTCATCCTGAGTAAAATCAACACACCCC
GTTGATTGGAAATTATATTCTACTTATTAAAAAAGAAAATTAAAATTCATAAATTTCCCCAAATAGGAATCTCTT
TCAATCGTGTAATGACAAGCAATATTTCAGTCCCTTGAGATCTTTTAAATAATGTCGGTTTCCTGCATGGTTTGT
GACATATAATTAAATGAATTAAATATTCAACGATGTGTCACAAGGATCTCGATCATTGGGCAGATGCTGAATCCA
CTGGGCACCATTGTATACTCCTTCAGGTTTTCTCTACATGTTGCATGGGAGGACAACCAAGTATAGTAAAGAAAG
CACCGTTTGGTACGCTACCTAATGATCACTAAAATAAAATAACCAAGAATGGATAAAAGCAAGCTTCTTTGAGCC
TCTGCGACAAGTTCTCCAGAGTTGTATGGCCTTGGGCTGCTTTCGATGATTGTTTAAAAATTACACGAGCCAATG
GTAGCCGGCTAAATCATTTCCCAGCCATAGCCGTTTGCTGCAGAAGTCTGACACCCTAGCTTCTCATCAGTATCC
CCAGCCTTACCTCATAGCCCAGGCACCTTGCTACTCCTTGTCGCCATACAGTCGGGCGTAGCGACCCAGCGCCCA
TATTGGAAACACGTTACGGTAACTCGTGTAGCTGATGGCACAACTTTTGTTGAATACTCCTTTGATATTCTCCTG
AGCCCAGTCACCGTTTGGTAGTTGTCGGTCCATGATGACCTTAATCCCACGATCAATAACGTCCTTGTCTGGAAA
TCTGACAGCCATGAGGCCCATCACAGCCCATGCCGTGTTGACAACTTGCGACGTCTTGCTCTGGATGTAACGACG
CTCCTCACAGGATTCGAACTCCTCACCCCATCCTCCATCTTCCATCTGCCTGGACAGGACGAAGGCACAAGCTCT
CTTGACTTCAGGAGTTGCTGTCATTGTTTCATAGCTATGGCCCATGCAGGCATAGGCTTCCAGACCAAACCATGT
TGCATAGGTGAAGCACACACCCCAAGAGCCTTCCCAAGATCCATCTGCTTTTTGCTTCTTAGCGATGTACGACAA
ACCATTGTCTAACGTGGACCGGATGTCATCCTTGCGATAATCGGGATACAAGTCGCTGAATTTCTTGAGTGATTG
CATGACAGCTGACGTCAGCTCAACGTAGGTGTAGTCTATCATGATGTCACCAAAAACTTCCGATGGATTCAGCTG
TTCCAGAAGCATGCCCCCTCTCGTTGTTTCATAGGTAGCAAATCCACCGTCATTATTACGCATGTTCAACATCAC
ATCGACAGCTTGGCAGTGTCTGTCGTTAGATATAGGCTCCTTGATGACAGTCCCCATTGTCTCCAGCATGAGAGC
CGCCTTAAGCCCCTCGGCAGTGCAGTCAGCCACGATCCAGCCACAATCACGTGTACTAAAAGGATAACCTCCCTT
GTTCATCTGTCTGTAGTATTTTTGGTAGTTGGGTGGGTTTTCTGGGATCTGGGTAAGTTTTAGGAAGTCATGTGT
CCGCTGCAAACAGTCTTGAAGATCGGGGTTTGTGGATGCTCCAGCTTCTAGGAGAGCTTGGGCTGCAAAAGCTGT
ATCCCACAGTTGGGAACCGTTGGTACCCTGCATTTTCATTCCGTCCAGCCCAATCCATAAATAGTCTGGAATTCG
TTGTACATGCTGCTTGAAAACCTCCGAGTCCGGACCGTCTGTCATCCACCTTATGAGCATGTTGATCACTTTGGA
AATAGGTCCAATACTGATGCAATTGGTGAATTGGTCGTCTGCTCTAATGTGGTCCAAACACTCGTCCAAAGCTTG
CTGACGGAAGGCGGGAAAATGGAAGGATTCATACACATCAAGAATACCAAAAAGCACATTGTAGAGCCAGCTGTG
TGGCGTGTACAAATCAACCTTTGCGATGTTGTTTCTTTGTTTTGGCCAGTCGATGGTAGAATAATCCTCGACATA
AAGTTCCTTGCGTAGCTCCCTAATCAAATCGTCTTCCTCGGCCTGAAACTTGCGTCCGTAACAGTAGCTCATGGG
GAGATAGACTTGCCTGCAGTGGCACCAGAGAGTGGAGGGGTGGGCCGGGATCCATTGGGGGAACAACCACAATTC
TGGAAAGAGTGTGTGCATACCATCCCAGCTGTAAACATTGAGCACCGACAGCCAGAATTTTCCCCATGACGGTAT
GGCAACAGCGCCCCCTAACGAGTGAAGCAGTTTCCGGCACTTCACCATGTCTGGGTCATCTGGTGGGACATCTAG
CAATCGCATAGCCACGTAGTTTGTTGCACAACCAAAGACTGTGGGCGGGCCCTCAATATGTAGTCCCCAGCCTCC
ATCCTGACACTGCACTGAACGGAGATATCGAACCATTTCTTTTCTGAAAGCCTCTGGTAATTCTGTTTTGGTGAT
GTAGCACACAATTAATAGTCCAGGCATAAGAAACAGCGGTCCACCGTAGTCCCCCGACCAATGACCATCCTCCGT
TTGAAGTTTGGAGTAGAAGATGGAACCATTGGTTGCTGCTTCTTGGGCTGAGGTGGGTTTGGCGAAGGGTGGAGC
TTCTTGGCTAATGTCCAAACCCAGACTAAATTTCTCCACCAGATTCTGTTCTCGGTCAATGGTTTCTCCATCTTC
GATGTACCGCCATGTCTGACGACCTTCCACATTGGACAGACGCCATCTTGTGAGGTCTGTAGCGGGCTCCGTCTT
GTGGGGGCCTCCCCTGTTTCGCCGGCCGCTCATATCGATGACTTGGAGGGCCTACAAATATGGATTCCGCACACA
ACGACCGTCCACTGGGCTAGAGTGTCACCTCAGCTCTGCTCTGCTCTGTACATGTAAAAGCAAATATACCTCCGA
TTCTGTAGCGAGTAGTGAATCAACAGGCTCACCGTCAGACCGTGAAGTCACGCAACTACAATTTATAGGCCTAGT
TTTGCACCGGCCAGAGACGAGCATACAGTATGTGCATGTACTGCTACTGTATCATGTACACACAAATGTCTCCTT
CATCACTTGAAGTCTGGTTGCACCAGAGACCTGAAGGCAGAAGGAGAAATGTGCAGGTACATGTACA
>gi|637916807|gb|GAVE01093484.1|
TTCAATCAATGTTCATTTCAGAACAAATCTCACTTTATGAGAACATGCATGAATGTAGCAGCCTGCTGTACGATA
TCCACCACAAAAACTTTGAATGTCTCAATGCATTTTCAGCACATAATTACACCCATGCTGTAGGGACACCATTAA
TTGTGTGTGCAGACCGTAACGCAGCCATTTGACATCCGTCAAGCTAATCAAGGGCTTAGTTCCCAATTAATATAT
CATGGGTTTTATTTTTGTGAAATTAAGTCTTTTGTATTTGTCCACATGAAAGTTAATAAAATGTGTATAATACTG
TATTATAATTCCTGTGCAGTCCTTGTCTGACATGTATGTTCATTTTGCAAATTAACCATCAATAATTTTTAAACA
CGTTAATTGAAATCAGTCGGGCATTCCCACGATTATTGCACAAGTTTTTCATCCTGAGTAAAATCAACACACCCC
GTTGATTGGAAATTATATTCTACTTATTAAAAAAGAAAATTAAAATTCATAAATTTCCCCAAATAGGAATCTCTT
```

TCAATCGTGTAATGACAAGCAATATTTCAGTCCCTTGAGATCTTTTAAATAATGTCGGTTTCCTGCATGGTTTGT
GACATATAATTAAATGAATTAAATATTCAACGATGTGTCACAAGGATCTCGATCATTGGGCAGATGCTGAATCCA
CTGGGCACCATTGTATACTCCTTCAGGTTTTCTCTACATGTTGCATGGGAGGACAACCAAGTATAGTAAAGAAAG
CACCGTTTGGTACGCTACCTAATGATCACTAAAATAAAATAACCAAGAATGGATAAAAGCAAGCTTCTTTGAGCC
TCTGCGACAAGTTCTCCAGAGTCGTATGTTGGACGTTTTTAAACTCTTTGATGGCCTTGGGCTGCTTTCGATGAT
TGTTTAAAAATTACACGAGCCAATGGTAGCCGGCTAAATCATTTCCCAGCCATAGCCATTTGCTGCAGAAGTCTG
GCACCCTAGCTTCTCATCAGTATCCCCAGCCTTACCTCATAGCCCAGGCACCTTGCTACTCCTTGTTGCCATACA
GTCGGGCATAGCGACCCAGCGCCCATATTGGAAACACGTTACGGTAACTCGTGTAGCTGATGGCACAACTTTTGT
TGAATACTCCTTTGATATTCTCCTGAGCCCAGTCACCGTTCGGTAGCTGTCGGTCCATGATGACCTTAATCCCAC
GATCAATAACGTCCTTGTCTGGAAATCTGACAGCCATGAGGCCCATGACAGCCCATGCTGTGTTGACAACTTGCG
ACGTCTTGCTCTGGATGTAACGACGCTCCTCGCAGGATTCAAACTCCTCACCCCATCCTCCATCTTCCATCTGCC
TGGACAGGACGAAGGCACAAGCTCTCTTGACTTCAGGAGTTGCTGTCATTGTTTCATAGCTATGGCCCATGCAGG
CATAGGCTTCCAGACCAAACCATGTTGCATAGGTGAAGCACACACCCCAAGAGCCTTCCCAAGATCCATCTGCTT
TTTGCTTCTTAGCGATGTACGACAAACCATTGTCTAACGTGGACCGGATGTCATCCTTGCGATAATCGGGATACA
AGTCGCTGAATTTCTTGAGTGATTGCATGACAGCTGACGTCAGCTCAACGTAGGTGTAGTCTATCATGATGTCAC
CAAAAACTTCCGATGGATTCAGCTGTTCCAGAAGCATGCCCCCTCTCGTTGTTTCATAGGTAGCAAATCCACCGT
CATTATTACGCATGTTCAACATCACATCGACAGCTTGGCAGTGTCTGTCGTTAGATATAGGCTCCTTGATGACAG
TCCCCATTGTCTCCAGCATGAGAGCCGCCTTAAGCCCCTCGGCAGTGCAGTCAGCCACGATCCAGCCACAATCAC
GTGTACTAAAAGGATAACCTCCCTTGTTCATCTGTCTGTAGTATTTTTGGTAGTTGGGTGGGTTTTCTGGGATCT
GGGTAAGTTTTAGGAAGTCATGTGTCCGCTGCAAACAGTCTTGAAGATCGGGGTTTGTGGATGCTCCAGCTTCTA
GGAGAGCTTGGGCTGCAAAAGCTGTATCCCACAGTTGGGAACCGTTGGTACCCTGCATTTTCATTCCGTCCAGCC
CAATCCATAAATAGTCTGGAATTCGTTGTACATGCTGCTTGAAAACCTCCGAGTCCGGACCGTCTGTCATCCACC
TTATGAGCATGTTGATCACTTTGGAAATAGGTCCAATACTGATGCAATTGGTGAATTGGTCGTCTGCTCTAATGT
GGTCCAAACACTCGTCCAAAGCTTGCTGACGGAAGGCGGAAAATGGAAGGATTCATACACATCAAGAATACCAA
AAAGCACATTGTAGAGCCAGCTGTGTGGCGTGTACAAATCAACCTTTGCGATGTTGTTTCTTTGTTTTGGCCAGT
CGATGGTAGAATAATCCTCGACATAAAGTTCCTTGCGTAGCTCCCTAATCAAATCGTCTTCCTCGGCCTGAAACT
TGCGTCCGTAACAGTAGCTCATGGGGAGATAGACTTGCCTGCAGTGGCACCAGAGAGTGGAGGGGTGGGCCGGGA
TCCATTGGGGGAACAACCACAATTCTGGAAAGAGTGTGTGCATACCATCCCAGCTGTAAACATTGAGCACCGACA
GCCAGAATTTTCCCCATGACGGTATGGCAACAGCGCCCCCTAACGAGTGAAGCAGTTTCCGGCACTTCACCATGT
CTGGGTCATCTGGTGGGACATCTAGCAATCGCATAGCCACGTAGTTTGTTGCACAACCAAAGACTGTGGGCGGGC
CCTCAATATGTAGTCCCCAGCCTCCATCCTGACACTGCACTGAACGGAGATATCGAACCATTTCTTTTCTGAAAG
CCTCTGGTAATTCTGTTTTGGTGATGTAGCACACAATTAATAGTCCAGGCATAAGAAACAGCGGTCCACCGTAGT
CCCCCGACCAATGACCATCCTCCGTTTGAAGTTTGGAGTAGAAGATGGAACCATTGGTTGCTGCTTCTTGGGCTG
AGGTGGGTTTGGCGAAGGGTGGAGCTTCTTGGCTAATGTCCAAACCCAGACTAAATTTCTCCACCAGATTCTGTT
CTCGGTCAATGGTTTCTCCATCTTCGATGTACCGCCATGTCTGACGACCTTCCACATTGGACAGACGCCATCTTG
TGAGGTCTGTAGCGGGCTCCGTCTTGTGGGGGCCTCCCCTGTTTCGCCGGCCGCTCATATCGATGACTTGGAGGG
CCTACAAATATGGATTCCGCACACAACGACCGTCCACTGGGCTAGAGTGTCACCTCAGCTCTGCTCTGCTCTGTA
CATGTAAAAGCAAATATACCTCCGATTCTGTAGCGAGTAGTGAATCAACAGGCTCACCGTCAGACCGTGAAGTCA
CGCAACTACAATTTATAGGCCTAGTTTTGCACCGGCCAGAGACGAGCATACAGTATGTGCATGTACTGCTACTGT
ATCATGTACACACAAATGTCTCCTTCATCACTTGAAGTCTGGTTGCACCAGAGACCTGAAGGCAGAAGGAGAAAT
GTGCAGGTACATGTACA
>gi|637916808|gb|GAVE01093483.1|
TTCAATCAATGTTCATTTCAGAACAAATCTCACTTTATGAGAACATGCATGAATGTAGCAGCCTGCTGTACGATA
TCCACCACAAAAACTTTGAATGTCTCAATGCATTTTCAGCACATAATTACACCCATGCTGTAGGGACACCATTAA
TTGTGTGTGCAGACCGTAACGCAGCCATTTGACATCCGTCAAGCTAATCAAGGGCTTAGTTCCCAATTAATATAT
CATGGGTTTTATTTTTGTGAAATTAAGTCTTTTGTATTTGTCCACATGAAAGTTAATAAAATGTGTATAATACTG
TATTATAATTCCTGTGCAGTCCTTGTCTGACATGTATGTTCATTTTGCAAATTAACCATCAATAATTTTTAAACA
CGTTAATTGAAATCAGTCGGGCATTCCCACGATTATTGCACAAGTTTTTCATCCTGAGTAAAATCAACACACCCC
GTTGATTGGAAATTATATTCTACTTATTAAAAAAGAAAATTAAAATTCATAAATTTCCCCAAATAGGAATCTCTT
TCAATCGTGTAATGACAAGCAATATTTCAGTCCCTTGAGATCTTTTAAATAATGTCGGTTTCCTGCATGGTTTGT
GACATATAATTAAATGAATTAAATATTCAACGATGTGTCACAAGGATCTCGATCATTGGGCAGATGCTGAATCCA
CTGGGCACCATTGTATACTCCTTCAGGTTTTCTCTACATGTTGCATGGGAGGACAACCAAGTATAGTAAAGAAAG
CACCGTTTGGTACGCTACCTAATGATCACTAAAATAAAATAACCAAGAATGGATAAAAGCAAGCTTCTTTGAGCC
TCTGCGACAAGTTCTCCAGAGTCGTATGTTGGACGTTTTTAAACTCTTTGATGGCCTTGGGCTGCTTTCGATGAT
TGTTTAAAAATTACACGAGCCAATGGTAGCCGGCTAAATCATTTCCCAGCCATAGCCATTTGCTGCAGAAGTCTG
GCACCCTAGCTTCTCATCAGTATCCCCAGCCTTACCTCATAGCCCAGGCACCTTGCTACTCCTTGTTGCCATACA
GTCGGGCATAGCGACCCAGCGCCCATATTGGAAACACGTTACGGTAACTCGTGTAGCTGATGGCACAACTTTTGT
TGAATACTCCTTTGATATTCTCCTGAGCCCAGTCACCGTTCGGTAGCTGTCGGTCCATGATGACCTTAATCCCAC
GATCAATAACGTCCTTGTCTGGAAATCTGACAGCCATGAGGCCCATGACAGCCCATGCTGTGTTGACAACTTGCG
ACGTCTTGCTCTGGATGTAACGACGCTCCTCGCAGGATTCAAACTCCTCACCCCATCCTCCATCTTCCATCTGCC
TGGACAGGACGAAGGCACAAGCTCTCTTGACTTCAGGAGTTGCTGTCATTGTTTCATAGCTATGGCCCATGCAGG

CATAGGCTTCCAGACCAAACCATGTTGCATAGGTGAAGCACACACCCCAAGAGCCTTCCCAAGATCCATCTGCTT
TTTGCTTCTTAGCGATGTACGACAAACCATTGTCTAACGTGGACCGGATGTCATCCTTGCGATAATCGGGATACA
AGTCGCTGAATTTCTTGAGTGATTGCATGACAGCTGACGTCAGCTCAACGTAGGTGTAGTCTATCATGATGTCAC
CAAAAACTTCCGATGGATTCAGCTGTTCCAGAAGCATGCCCCCTCTCGTTGTTTCATAGGTAGCAAATCCACCGT
CATTATTACGCATGTTCAACATCACATCGACAGCTTGGCAGTGTCTGTCGTTAGATATAGGCTCCTTGATGACAG
TCCCCATTGTCTCCAGCATGAGAGCCGCCTTAAGCCCCTCGGCAGTGCAGTCAGCCACGATCCAGCCACAATCAC
GTGTACTAAAAGGATAACCTCCCTTGTTCATCTGTCTGTAGTATTTTTGGTAGTTGGGTGGGTTTTCTGGGATCT
GGGTAAGTTTTAGGAAGTCATGTGTCCGCTGCAAACAGTCTTGAAGATCGGGGTTTGTGGATGCTCCAGCTTCTA
GGAGAGCTTGGGCTGCAAAAGCTGTATCCCACAGTTGGGAACCGTTGGTACCCTGCATTTTCATTCCGTCCAGCC
CAATCCATAAATAGTCTGGAATTCGTTGTACATGCTGCTTGAAAACCTCCGAGTCCGGACCGTCTGTCATCCACC
TTATGAGCATGTTGATCACTTTGGAAATAGGTCCAATACTGATGCAATTGGTGAATTGGTCGTCTGCTCTAATGT
GGTCCAAACACTCGTCCAAAGCTTGCTGACGGAAGGCGGGAAAATGGAAGGATTCATACACATCAAGAATACCAA
AAAGCACATTGTAGAGCCAGCTGTGTGGCGTGTACAAATCAACCTTTGCGATGTTGTTTCTTTGTTTTGGCCAGT
CGATGGTAGAATAATCCTCGACATAAAGTTCCTTGCGTAGCTCCCTAATCAAATCGTCTTCCTCGGCCTGAAACT
TGCGTCCGTAACAGTAGCTCATGGGGAGATAGACTTGCCTGCAGTGGCACCAGAGAGTGGAGGGGTGGGCCGGGA
TCCATTGGGGGAACAACCACAATTCTGGAAAGAGTGTGTGCATACCATCCCAGCTGTAAACATTGAGCACCGACA
GCCAGAATTTTCCCCATGACGGTATGGCAACAGCGCCCCCTAACGAGTGAAGCAGTTTCCGGCACTTCACCATGT
CTGGGTCATCTGGTGGGACATCTAGCAATCGCATAGCCACGTAGTTTGTTGCACAACCAAAGACTGTGGGCGGGC
CCTCAATATGTAGTCCCCAGCCTCCATCCTGACACTGCACTGAACGGAGATATCGAACCATTTCTTTTCTGAAAG
CCTCTGGTAATTCTGTTTTGGTGATGTAGCACACAATTAATAGTCCAGGCATAAGAAACAGCGGTCCACCGTAGT
CCCCCGACCAATGACCATCCTCCGTTTGAAGTTTGGAGTAGAAGATGGAACCATTGGTTGCTGCTTCTTGGGCTG
AGGTGGGTTTGGCGAAGGGTGGAGCTTCTTGGCTAATGTCCAAACCCAGACTAAATTTCTCCACCAGATTCTGTT
CTCGGTCAATGGTTTCTCCATCTTCGATGTACCGCCATGTCTGACGACCTTCCACATTGGACAGACGCCATCTTG
TGAGGTCTGTAGCGGGCTCCGTCTTGTGGGGGCCTCCCCTGTTTCGCCGGCCGCTCATATCGATGACTTGGAGGG
CCTACAAATATGGATTCCTCCGATTCTGTAGCGAGTAGTGAATCAACAGGCTCACCGTCAGACCGTGAAGTCACG
CAACTACAATTTATAGGCCTAGTTTTGCACCGGCCAGAGACGAGCATACAGTATGTGCATGTACTGCTACTGTAT
CATGTACACACAAATGTCTCCTTCATCACTTGAAGTCTGGTTGCACCAGAGACCTGAAGGCAGAAGGAGAAATGT
GCAGGTACATGTACA
>gi|638755796|gb|GAVP01109444.1|
TGGGCAAAGTAACAAGCACAACATTTGGTATTGTGCCTAATCACCACTGGAATGAAATAACCAAGAATACATAGT
ATATTTTATTAAGTGTTGGGTCCTCATTTACAAAATACTAGAAAGCTTCTTTGGTGTTTCTCTTAAACACATCCG
CGACAAGTACTATAGTTAGCTGAATTCCTTCAAAATCTTAAGTTGACAGTTTTTAGACTGTTTTGAGGGTGTAGG
GCTGCTTTCAGATTTTTCGTTATAGCTTGAAATTTACACACAAGTCAATAGTAGCCACCAAATCCCATAGACTTG
TGTATTATTTCCCAGCCATAGCCATTTGCTGGAGAGGTCTGGCACCCTACCTCCTCATCAGTATCCACAACCTTA
CCTCTTAGAGAAGCCAAGGTAGCCAAGTCAGGCACCTTCCTATTCTTTGTTTCCATAAAGACGTGCATATCGACC
CAGTGCCCATATTGGAAAGACGTTACGGTAATTCGTATAGCTGATGGCACAACTTTTGTTGAATACTCCTTTGAT
ATTTTCCTGAGCCCAATCACCGTTTGGCAGCTGTCGGTCCATGATGACTTTAATTCCGCGATCAATAACGTCTTT
TTCTGGAAATCTGACAGCCATAAGGCCCATGACAGCCCAAGCCGTGTTGACAACTTGCGACGTTTTGCTCTGGAC
GTAACGACGCACTTCGCATGATTCGAACTCCTCACCCCATCCCCCATCCTCCATCTGTCTGGACAGGAGGAAGGC
GCATGCTCTCTTGATTTCAGGAGTCGCTGTCGTTGTTTCATATGTATGGCCCATGCAGGCATAGGCTTCCAGACC
AAACCATGTCGCATAAGTGAAGCACACACCCCAGGATCCTTCCCATGATCCATCTGTCTTCTGCTTCTTAGCGAT
GTACGACAAACCGTTGTCCAATGTGGACCGGACGTCGTCCTTGCGATATTCGGGATACAACTCACTGAATTTCTT
AAGTGACTGCATGACAGCCGAAGTCAGCTCCACATAGGTATAGTCTATCATGATGTCACCAAAAACTTCTGATGG
ATTCATCTGCTCCAGAATCATGCCCCCTCTCATTGTTTCATAGGTCGCAAATCCGCCGTCAGGATTACGCATGTT
CAACATCACATCGACAGCTTGGCAGTGTCTGTCCTTAGATATTGGCTCTTTAACGGCAGTCCCCTGTGTCTCTAG
CATAAGGGCCGCTTTGAGCCCTTCGGCAGTGCAGTCAGCAACAATCCAGCCACAATCTCGTGTACTGAAGGGATA
ACCTCCCTTGTTCATCTGCCTGTAGTACTTTTGGTAGTTGGGTGGGTTTTCTGGGATCTGGGAAAGCTTCAGGAA
GTCATGTGTCCGTTGCAAACAGTCATGAAGATCGGCGTTTGTGGATGCTCCAGCATCAAGGAGTGCTTGGGCTGC
AAAAGCTGTATCCCAGAGCTGTGAGCCGTTGGTACCCTGCATTTTCATACCATCTAGTCCAATCCATAAATAGTC
CGGGATTCTTTGCACATGTTTCTTGAAAACCTCAGAGTCTGGGCCGTCTGTCATCCACCTCACAAGCATGTTGAT
GACTTTGGAAATAGGCCCAATGCTGATGCACTTTGTGAATTGGTCGTCTGCCCTGATGTGGTCCAAGCATTCATC
CAAGGCTTGCTGGCGGAAAGCAGAAAAATGGAAGGACTCGTACACATCAAGGGTACCAAAAACCACATTGTACAA
CCAGCTGTGAGGCGTGTACAAATCAACTTTGGCGATGTTGTTTCTTTGTTTCGACCAGTTGATAGTAGAATAATC
CTCGACATAAAGTTCCTTGCGTAGTTCTCTAATCAAATCATCTTCTTCGGCCTGAAACTTGCGTCCATAGCAGTA
GCTCATGGGGAGATAGACTTGCCTGCAATGGCACCAGATGGTGGAGGGGTGGGCGGGGATCCATTGGGGGAACAA
CCACAATTCTGGAAAGAGTGTGTGCATACCGTCCCAGCTGTAGACGTTGAGCACCGACAGCCAGAACTTGCCCCA
TGATGGTATGGCAACAGCGCCCCCTAATGAGTGAAGCAGTTTTCGGCATTTCATCAGGTCTGGGTCATCTGGCGA
AACATCCAGTAATCGCATGGCCACGTAGTTTGTTGCACAGCCAAAGACTGTGGGCGGGCCTTCAATATGCAGTCC
CCAGCCTCCATCTTGACATTGCACAGAGCGGAGATATCGCACCATTTCTTTTCTGAAAGCTTCTGGTAATTCTGT
TTTGGTGATGTAGCACACGATTAGGAGCCCTGGCATAAGGAACAATGGTCCGCCATAGTCTCCGGACCAATGACC
ATCCTCCGTTTGCAATCTGGAGTAGAAGATGGAGCCATTAGTTGCTGCTTCTTGGGCTGAGGTGGGTTTGGGGAA

GGGCGGGGCTTCTTTGCTAATGTCTAAGCCCAGACTAAACTTCTCCACCAGATTCTGTTCTCTGTCGATAGTTTC
CCCTTCTTCAATGTACCGCCATGTCTGCCGACCGTCAACGTTAGACAACCTCCATCTTGTGAGGTCTGTAACGGG
CTCCGTCTTGTGGGGACCTCCTCTGTTTCGGCGGCCGCTCATATCGATGAATTGGGGGCCTACAAATATGGAATT
CCCTACGAAGTGTAGCAACTTTCGCTGATTTTAAGCATACCGACATTTGTTGCAAAGCCGCTCATAAATTGGGTG
AATTTGCCAACGGAACATCGGCAGTCAGTTGGCCACTTTCGAACCGATTTGCTAGTTCCGAATAGGGCGCCCTCT
CTGCACAGACTACATGGAGATTTTTTCGCCCAACTTCATAGATATGCCTAGGCCTACTACTAGATGTCAGCAATT
ATAATTGTGACAAAAGCTGCACTATGAAGTGCTTAAAATGGGGGAAAGAGGGCACCACCTACACCTTTGTACAAA
AAAAGGAGAAAAATCAAAAGCAGGTGAAAATGGAAATTTTATCTGCTTGAAAGCACCATTTTTTCCTTTAATTCA
TCATTCTTGAATATTTGTTTGAAAACCATTTTTTTTTTTAGGTCCAGCTCCAGAATTAAGCCTCGTCTTGTTCCG
AGTGATACATGTAGCCTAGTATCCTCGATCCTCCATAATCTGCGCAACTTACACTTTAATATGCACGCGCGCGCA
TTGGCCGGCAAACATTAAGGTGAGTGCATAGAAATAGGACCGCGGGGATTTAGGGGTTCTGACGATACAAATTTC
AGCTGTTGTTGAAATATTTCAGTCAGTGACAGGCAGGGGTTTTCTGTGGTAGTGATAATCATTCAAAAATGGAAA
CATCACCGATGACTGTACTTGAAAATGTTGACGTTGACACAAACAAGTAAAGCAGACTCGAACAAAACAGACGAC
AGCGTTGCATGCGCATTCTCTTAGCGCCTAGAACAAGAAGCTTTATGAAATAGGGCCTTGAAGTGAACATTCGGG
CTATCCTTAATCCAAAATTTTTGATTAGG
>gi|638755798|gb|GAVP01109442.1|
TGGGCAAAGTAACAAGCACAACATTTGGTATTGTGCCTAATCACCACTGGAATGAAATAACCAAGAATACATAGT
ATATTTTATTAAGTGTTGGGTCCTCATTTACAAAATACTAGAAAGCTTCTTTGGTGTTTCTCTTAAACACATCCG
CGACAAGTACTATAGTTAGCTGAATTCCTTCAAAATCTTAAGTTGACAGTTTTTAGACTGTTTTGAGGGTGTAGG
GCTGCTTTCAGATTTTTCGTTATAGCTTGAAATTTACACACAAGTCAATAGTAGCCACCAAATCCCATAGACTTG
TGTATTATTTCCCAGCCATAGCCATTTGCTGGAGAGGTCTGGCACCCTACCTCCTCATCAGTATCCACAACCTTA
CCTCTTAGAGAAGCCAAGGTAGCCAAGTCAGGCACCTTCCTATTCTTTGTTTCCATAAAGACGTGCATATCGACC
CAGTGCCCATATTGGAAAGACGTTACGGTAATTCGTATAGCTGATGGCACAACTTTTGTTGAATACTCCTTTGAT
ATTTTCCTGAGCCCAATCACCGTTTGGCAGCTGTCGGTCCATGATGACTTTAATTCCGCGATCAATAACGTCTTT
TTCTGGAAATCTGACAGCCATAAGGCCCATGACAGCCCAAGCCGTGTTGACAACTTGCGACGTTTTGCTCTGGAC
GTAACGACGCACTTCGCATGATTCGAACTCCTCACCCCATCCCCCATCCTCCATCTGTCTGGACAGGAGGAAGGC
GCATGCTCTCTTGATTTCAGGAGTCGCTGTCGTTGTTTCATATGTATGGCCCATGCAGGCATAGGCTTCCAGACC
AAACCATGTCGCATAAGTGAAGCACACACCCCAGGATCCTTCCCATGATCCATCTGTCTTCTGCTTCTTAGCGAT
GTACGACAAACCGTTGTCCAATGTGGACCGGACGTCGTCCTTGCGATATTCGGGATACAACTCACTGAATTTCTT
AAGTGACTGCATGACAGCCGAAGTCAGCTCCACATAGGTATAGTCTATCATGATGTCACCAAAAACTTCTGATGG
ATTCATCTGCTCCAGAATCATGCCCCCTCTCATTGTTTCATAGGTCGCAAATCCGCCGTCAGGATTACGCATGTT
CAACATCACATCGACAGCTTGGCAGTGTCTGTCCTTAGATATTGGCTCTTTAACGGCAGTCCCCTGTGTCTCTAG
CATAAGGGCCGCTTTGAGCCCTTCGGCAGTGCAGTCAGCAACAATCCAGCCACAATCTCGTGTACTGAAGGGATA
ACCTCCCTTGTTCATCTGCCTGTAGTACTTTTGGTAGTTGGGTGGGTTTTCTGGGATCTGGGAAAGCTTCAGGAA
GTCATGTGTCCGTTGCAAACAGTCATGAAGATCGGCGTTTGTGGATGCTCCAGCATCAAGGAGTGCTTGGGCTGC
AAAAGCTGTATCCCAGAGCTGTGAGCCGTTGGTACCCTGCATTTTCATACCATCTAGTCCAATCCATAAATAGTC
CGGGATTCTTTGCACATGTTTCTTGAAAACCTCAGAGTCTGGGCCGTCTGTCATCCACCTCACAAGCATGTTGAT
GACTTTGGAAATAGGCCCAATGCTGATGCACTTTGTGAATTGGTCGTCTGCCCTGATGTGGTCCAAGCATTCATC
CAAGGCTTGCTGGCGGAAAGCAGAAAAATGGAAGGACTCGTACACATCAAGGGTACCAAAAACCACATTGTACAA
CCAGCTGTGAGGCGTGTACAAATCAACTTTGGCGATGTTGTTTCTTTGTTTCGACCAGTTGATAGTAGAATAATC
CTCGACATAAAGTTCCTTGCGTAGTTCTCTAATCAAATCATCTTCTTCGGCCTGAAACTTGCGTCCATAGCAGTA
GCTCATGGGGAGATAGACTTGCCTGCAATGGCACCAGATGGTGGAGGGGTGGGCGGGGATCCATTGGGGGAACAA
CCACAATTCTGGAAAGAGTGTGTGCATACCGTCCCAGCTGTAGACGTTGAGCACCGACAGCCAGAACTTGCCCCA
TGATGGTATGGCAACAGCGCCCCCTAATGAGTGAAGCAGTTTTCGGCATTTCATCAGGTCTGGGTCATCTGGCGA
AACATCCAGTAATCGCATGGCCACGTAGTTTGTTGCACAGCCAAAGACTGTGGGCGGGCCTTCAATATGCAGTCC
CCAGCCTCCATCTTGACATTGCACAGAGCGGAGATATCGCACCATTTCTTTTCTGAAAGCTTCTGGTAATTCTGT
TTTGGTGATGTAGCACACGATTAGGAGCCCTGGCATAAGGAACAATGGTCCGCCATAGTCTCCGGACCAATGACC
ATCCTCCGTTTGCAATCTGGAGTAGAAGATGGAGCCATTAGTTGCTGCTTCTTGGGCTGAGGTGGGTTTGGGGAA
GGGCGGGGCTTCTTTGCTAATGTCTAAGCCCAGACTAAACTTCTCCACCAGATTCTGTTCTCTGTCGATAGTTTC
CCCTTCTTCAATGTACCGCCATGTCTGCCGACCGTCAACGTTAGACAACCTCCATCTTGTGAGGTCTGTAACGGG
CTCCGTCTTGTGGGGACCTCCTCTGTTTCGGCGGCCGCTCATATCGATGAATTGGGGGCCCTACGAAGTGTAGCA
ACTTTCGCTGATTTTAAGCATACCGACATTTGTTGCAAAGCCGCTCATAAATTGGGTGAATTTGCCAACGGAACA
TCGGCAGTCAGTTGGCCACTTTCGAACCGATTTGCTAGTTCCGAATAGGGCGCCCTCTCTGCACAGACTACATGG
AGATTTTTTCGCCCAACTTCATAGATATGCCTAGGCCTACTACTAGATGTCAGCAATTATAATTGTGACAAAAGC
TGCACTATGAAGTGCTTAAAATGGGGGAAAGAGGGCACCACCTACACCTTTGTACAAAAAAAGGAGAAAAATCAA
AAGCAGGTGAAAATGGAAATTTTATCTGCTTGAAAGCACCATTTTTTCCTTTAATTCATCATTCTTGAATATTTG
TTTGAAAACCATTTTTTTTTTTAGGTCCAGCTCCAGAATTAAGCCTCGTCTTGTTCCGAGTGATACATGTAGCCT
AGTATCCTCGATCCTCCATAATCTGCGCAACTTACACTTTAATATGCACGCGCGCGCATTGGCCGGCAAACATTA
AGGTGAGTGCATAGAAATAGGACCGCGGGGATTTAGGGGTTCTGACGATACAAATTTCAGCTGTTGTTGAAATAT
TTCAGTCAGTGACAGGCAGGGGTTTTCTGTGGTAGTGATAATCATTCAAAAATGGAAACATCACCGATGACTGTA
CTTGAAAATGTTGACGTTGACACAAACAAGTAAAGCAGACTCGAACAAAACAGACGACAGCGTTGCATGCGCATT

CTCTTAGCGCCTAGAACAAGAAGCTTTATGAAATAGGGCCTTGAAGTGAACATTCGGGCTATCCTTAATCCAAAA
TTTTTGATTAGG
>gi|638755800|gb|GAVP01109440.1|
TGGGCAAAGTAACAAGCACAACATTTGGTATTGTGCCTAATCACCACTGGAATGAAATAACCAAGAATACATAGT
ATATTTTATTAAGTGTTGGGTCCTCATTTACAAAATACTAGAAAGCTTCTTTGGTGTTTCTCTTAAACACATCCG
CGACAAGTACTATAGTTAGCTGAATTCCTTCAAAATCTTAAGTTGACAGTTTTTAGACTGTTTTGAGGGTGTAGG
GCTGCTTTCAGATTTTTCGTTATAGCTTGAAATTTACACACAAGTCAATAGTAGCCACCAAATCCCATAGACTTG
TGTATTATTTCCCAGCCATAGCCATTTGCTGGAGAGGTCTGGCACCCTACCTCCTCATCAGTATCCACAACCTTA
CCTCTTAGAGAAGCCAAGGTAGCCAAGTCAGGCACCTTCCTATTCTTTGTTTCCATAAAGACGTGCATATCGACC
CAGTGCCCATATTGGAAAGACGTTACGGTAATTCGTATAGCTGATGGCACAACTTTTGTTGAATACTCCTTTGAT
ATTTTCCTGAGCCCAATCACCGTTTGGCAGCTGTCGGTCCATGATGACTTTAATTCCGCGATCAATAACGTCTTT
TTCTGGAAATCTGACAGCCATAAGGCCCATGACAGCCCAAGCCGTGTTGACAACTTGCGACGTTTTGCTCTGGAC
GTAACGACGCACTTCGCATGATTCGAACTCCTCACCCCATCCCCCATCCTCCATCTGTCTGGACAGGAGGAAGGC
GCATGCTCTCTTGATTTCAGGAGTCGCTGTCGTTGTTTCATATGTATGGCCCATGCAGGCATAGGCTTCCAGACC
AAACCATGTCGCATAAGTGAAGCACACACCCCAGGATCCTTCCCATGATCCATCTGTCTTCTGCTTCTTAGCGAT
GTACGACAAACCGTTGTCCAATGTGGACCGGACGTCGTCCTTGCGATATTCGGGATACAACTCACTGAATTTCTT
AAGTGACTGCATGACAGCCGAAGTCAGCTCCACATAGGTATAGTCTATCATGATGTCACCAAAAACTTCTGATGG
ATTCATCTGCTCCAGAATCATGCCCCCTCTCATTGTTTCATAGGTCGCAAATCCGCCGTCAGGATTACGCATGTT
CAACATCACATCGACAGCTTGGCAGTGTCTGTCCTTAGATATTGGCTCTTTAACGGCAGTCCCCTGTGTCTCTAG
CATAAGGGCCGCTTTGAGCCCTTCGGCAGTGCAGTCAGCAACAATCCAGCCACAATCTCGTGTACTGAAGGGATA
ACCTCCCTTGTTCATCTGCCTGTAGTACTTTTGGTAGTTGGGTGGGTTTTCTGGGATCTGGGAAAGCTTCAGGAA
GTCATGTGTCCGTTGCAAACAGTCATGAAGATCGGCGTTTGTGGATGCTCCAGCATCAAGGAGTGCTTGGGCTGC
AAAAGCTGTATCCCAGAGCTGTGAGCCGTTGGTACCCTGCATTTTCATACCATCTAGTCCAATCCATAAATAGTC
CGGGATTCTTTGCACATGTTTCTTGAAAACCTCAGAGTCTGGGCCGTCTGTCATCCACCTCACAAGCATGTTGAT
GACTTTGGAAATAGGCCCAATGCTGATGCACTTTGTGAATTGGTCGTCTGCCCTGATGTGGTCCAAGCATTCATC
CAAGGCTTGCTGGCGGAAAGCAGAAAAATGGAAGGACTCGTACACATCAAGGGTACCAAAAACCACATTGTACAA
CCAGCTGTGAGGCGTGTACAAATCAACTTTGGCGATGTTGTTTCTTTGTTTCGACCAGTTGATAGTAGAATAATC
CTCGACATAAAGTTCCTTGCGTAGTTCTCTAATCAAATCATCTTCTTCGGCCTGAAACTTGCGTCCATAGCAGTA
GCTCATGGGGAGATAGACTTGCCTGCAATGGCACCAGATGGTGGAGGGGTGGGCGGGGATCCATTGGGGGAACAA
CCACAATTCTGGAAAGAGTGTGTGCATACCGTCCCAGCTGTAGACGTTGAGCACCGACAGCCAGAACTTGCCCCA
TGATGGTATGGCAACAGCGCCCCCTAATGAGTGAAGCAGTTTTCGGCATTTCATCAGGTCTGGGTCATCTGGCGA
AACATCCAGTAATCGCATGGCCACGTAGTTTGTTGCACAGCCAAAGACTGTGGGCGGGCCTTCAATATGCAGTCC
CCAGCCTCCATCTTGACATTGCACAGAGCGGAGATATCGCACCATTTCTTTTCTGAAAGCTTCTGGTAATTCTGT
TTTGGTGATGTAGCACACGATTAGGAGCCCTGGCATAAGGAACAATGGTCCGCCATAGTCTCCGGACCAATGACC
ATCCTCCGTTTGCAATCTGGAGTAGAAGATGGAGCCATTAGTTGCTGCTTCTTGGGCTGAGGTGGGTTTGGGGAA
GGGCGGGGCTTCTTTGCTAATGTCTAAGCCCAGACTAAACTTCTCCACCAGATTCTGTTCTCTGTCGATAGTTTC
CCCTTCTTCAATGTACCGCCATGTCTGCCGACCGTCAACGTTAGACAACCTCCATCTTGTGAGGTCTGTAACGGG
CTCCGTCTTGTGGGGACCTCCTCTGTTTCGGCGGCCGCTCATATCGATGAATTGGGGGCCTACAAATATGGAATT
CGTCCACGATGAAATCACGCACAGAAATGTTATAATTCAGCTAAATTGAAGCGGCATTTGAGGACTCAACCACGG
CCGGCCACGAGCTGTTTATGTTG
>gi|637707158|gb|GAUS01043709.1|
AGGAAATGAAATTGATGTTTGAATGAGTAATTTTTGAACCAATTCAAGTTCATATCTTGTGTGAACAACTTTAAC
AATGAAATATATTCCATGGTGTAGTTTTTAATCAAAGTTGATATCAAAATTCCGGGAAATCATTACAAAATTACT
TGAATGAAGAACAACTTCAACAGCAAGAACTTCATGAGTAGGTGGAGCATTGGTTTGGTTATTAATAGTTCAAAG
ATCAAAGGTCGTGTACACACTTTAAGTTGCTGAATGTCATCCATGCACACACACACATCATGCAGTTCGTGCATG
CAGTATTGAAGTAAACTCTTTGAAAGAGTTATGCTGCCGGCCTTTTAATCAACTTTTATGAGCAAGACAACTCTA
GAGGCTTTATTGGAGAGAAGATTCAAAAGTTGGACCCAACATGAGCGGCCGACGGAACCGAGGAGGTCCCCACAA
AACAGAGCCAGCTACCGATCTCACAAGATGGCGCCTCTCTAATGTCGATGGACGGCAGACGTGGCGCTTCATCGA
GGAGGGTGAACCAGTGGACAGAGAGCAAAACTTTGTTGAGAAGTTTAGCTTGGGCTTAGACATTAGTGAGGAGGC
GCCACCCTTGGCCAAACCAGCCACAGCCCAAGAAGCAGCGACTAATGGCATGCTGTTTTACTCCAAGCTTCAAAC
AGAAGATGGCCACTGGAGTGGGGACTATGGGGGGCCTCTCTTCCTTATGCCAGGTCTACTTATAGTCTGCTACAT
CACAAAGACTGAACTACCAGATGCAGTTAAGAAGGAGATGGTCCGGTACCTGCGCTCTGTTCAGTGTCAAGATGG
CGGATGGGGTTTACATATTGAAGGCCCGCCTACGGTCTTTGGTTGCGCAACCAACTATGTCGCTATGAGACTCCT
TGGTATTGGTCCGGAAGATCCAGACCTGATGAAATGTAGGAAGCTTCTTCATTCCTTAGGAGGCGCTGCTGCTAT
ACCTTCTTGGGGCAAGTTCTGGCTGTCGGTTCTGAATGTTTATCAGTGGGATGGAATGCACACCCTCTTTCCTGA
GCTCTGGAATTTTCCAACGTATACTCCCGCTCATCCTTCTACCATCTGGTGCCATTGCAGACAGGTTTACCTGCC
GATGAGTTACTGCTATGGGATCAAATTCCAGGCTGAACCAGATGACTTGATCATGGATCTCAGGATGGAGCTTTA
TGTGGAGGACTATTACACCATCAATTGGCCAGCACAGCGAAGCAACATAGCACAAGTGGACTTGTACACGCCCCA
TAGTTGGCTGTATAACTTAGCATTCGGGTGCCTAGATATATATGAGACGTACCATAGTACTAAATGGCGGCAGAG
TGCACTAGACGAGTGCCTTGACCACATCAAAGCTGACGATGAGTTCACTCAATGCATCAGTATCGGACCTATCTC
CAAAGTGATCAACATGTTGGTGCGATGGATGACAGACGGACCGGACTCGGCCATCTTCAAACAACATGTTGAGCG

TATTCCTGATTATTTGTGGATCGGTTTGGATGGAATGAAAATGCAGGGCACCAATGGCTCCCAGTTGTGGGACAC
GGCATTTGCTGCCCAAGCCTTCCTAGAGGGAGGGGCTGCAAAGAACGAGTCACTCCATGAGTGCCTTCAAAGGAC
TCACGATTTCCTCAAACTCACCCAGATCCCAGAAAACCCACCAAATTATCAGAAGTACTACAGGCAGATGAACAA
GGGTGGGTACCCATTTAGCACACGTGACTGCGGTTGGATCGTTGCTGATTGTACTGCAGAGGGACTCAAGTCTGC
GCTGATGATTGAACAGCGATGTCCATTTCTCAAGGACCACATCACACAAGAAAGACACCAAGAAGCTGTGGATAT
TATGCTGAACATGCGAAATAGTGACGGCGGATTTGCAACGTACGAGACCATGCGAGGGGGCGTAATTCTTGAGAA
ACTTAATCCATCAGAAGTCTTTGGTGACATCATGATAGACTACACCTACGTAGAACTTACCTCGGCTGTTTTGCA
ATCACTCAAGAAGTTCAGTGACATTTATCCCGATTATCGCCAAGCGGAAATCCGAGCTACACTGGACCGTGGGCT
GTTGTACATTGCTGATAAACAGAAGCGGGATGGATCATGGGAAGGTTCCTGGGGTGTTTGCTTCACTTATGCCGC
CTGGTTTGGTCTGGAAGCCTACGCCTGCATGGGATACAGCTACAAAACATCGACAGCATCCAGCGAGGTAAAGAG
GGCGTGTGCTTTCCTCCTCTCTCGTCAGATGGAAGACGGAGGATGGGGAGAGGAGTTTGAATCTTGTGAAGAACG
TCGTTACATCCAGAGTAAAACCTCGCAGGTTGTCAACACGGCATGGTCTGTACTGGCACTCATGGCTGTCAGATT
TCCAGAGCGTGAAGTCATCGATCGCGGCATTCGGGTCATCAGAGACAGGCAGCTGCCGAACGGGGATTGGGCTCA
GGAGAGTATAAAAGGCGTGTTCAACAAGTCATGTGCAATCAGCTACACCAGCTATCGCAACCTGTTCCCAATATG
GGCTCTGGGTCGATATGCAAGGATCTACGGTGACGATGCATAGAGGTATAGGGATGTACAATCATGTACCCTTAG
CCGAAGCTATGGCTGTTAAGATATCATTCAGGGGTCGATTTCACAAAGGGTTTTTAAAACTAGTTCTTACTTGGA
GCGTCCTAGGAGATATTGAAAACATAAGGCTAGTCCTAACTCAAGATAAGACAAGT
>gi|637859443|gb|GAUU01031164.1|
CATCCACACACACACACATCATGCAGTTCGTGCATGCAGTATTGAAGTAAACTCTTTGAAAGAGTTATGCTGCCG
GCCTTTTAATCAACTTTTATGAGCTGATAAATAGACATCTCTAGAGGTTTTATTGGAGAGAAGATTCAAAAGTTG
GACCCAACATGAGCGGCCGACGGAACCGAGGAGGTCCCCACAAAACAGAGCCAGCTACCGATCTCACAAGATGGC
GCCTCTCTAATGTCGATGGACGGCAGACGTGGCGCTTCATCGAGGAGGGTGAACCAGTGGACAGAGAGCAAAACT
TTGTTGAGAAGTTTAGCTTGGGCTTAGACATTAGTGAGGAGGCGCCACCCTTGGCCAAACCAGCCACAGCCCATA
AAGCAGCGACTAATGGCATGCTGTTTTACTCCAAGCTTCAAACAGAAGATGGCCACTGGAGTGGGGACTATGGGG
GGCCTCTCTTCCTTATGCCAGGTCTACTTATAGTCTGCTACATCACAAAGACTGAACTACCAGATGCAGTTAAGA
AGGAGATGGTCCGGTACCTGCGCTCTGTTCAGTGTCAAGATGGCGGATGGGGTTTACATGTTGAAGGCCCGCCTA
CGGTCTTTGGTTGCGCAACCAACTATGTCGCTATGAGACTCCTTGGTATTGGTCCGGAAGATCCAGACCTGATGA
AATGTAGGAAGCTTCTTCATTCCTTAGGAGGCGCTGCCGCTATACCTTCTTGGGGCAAGTTCTGGCTGTCGGTTC
TGAATGTTTATCAGTGGGATGGAATGCACACCCTCTTTCCTGAGCTCTGGAATTTTCCAACGTATACTCCCGCTC
ATCCTTCTACCATCTGGTGCCATTGCAGACAGGTTTACCTGCCGATGAGTTACTGCTATGGGATCAAATTCCAGG
CTGAACCAGATGACTTGATCATGGATCTCAGGATGGAGCTTTATGTGGAGGACTATTACACCATCAATTGGCCAG
CACAGCGAAGCAACATAGCACAAGTGGACTTGTACACGCCCCATAGTTGGCTGTATAACTTAGCATTCGGGTGCC
TAGATATATATGAGAAGTACCACAGTACTAAATGGCGGCAGAGTGCGCTAGACGAGTGCCTTGACCACATCAAAG
CTGACGATGAGTTCACTCAATGCATCAGTATCGGACCTATCTCTAAAGTGATCAACATGTTGGTGCGATGGATGA
CAGACGGACCGGACTCGGCCATCTTCAAACAACATGTTGAGCGTATTCCTGATTATTTGTGGATCGGTTTGGATG
GAATGAAAATGCAGGGCACCAATGGCTCCCAGTTGTGGGACACGGCATTTGCTGCCCAAGCCTTCCTAGAGGGAG
GGGCTGCAAAGAACGAGTCACTCCATGAATGCCTTCAAAGGACTCACGATTTCTTCAAACTCACCCAGATCCCAG
AAAACCCACCAAATTATCAGAAGTACTACAGGCAGATGAACAAGGGTGGGTACCCATTCAGCACACGTGACTGCG
GTTGGATCGTTGCTGATTGTACTGCAGAGGGACTCAAGTCTGCGCTGATGATTGAACAGCAATGTCCATTTCTCA
AGGACCACATCACACAAGAAAGACACCAAGAAGCTGTGGATGTTATGCTGAACATGCGAAATAGCGACGGCGGAT
TTGCAACGTACGAGACCATGCGAGGGGGCGTAATTCTTGAGAAACTTAATCCATCAGAAGTCTTTGGTGACATCA
TGATAGACTACACCTACGTAGAACTTACCTCGGCTGTTTTGCAATCACTCAAGAAGTTCAGTGACATTTATCCCG
ATTATCGCCAAGCGGAAATCCGAGCTACACTGGACCGTGGGCTGTTGTACATCGCTGATAAACAGAAGCGGGATG
GATCATGGGAAGGTTCCTGGGGTGTTTGCTTCACTTATGCCGCCTGGTTTGGTCTGGAAGCCTACGCCTGCATGG
GATACAGCTACAAAACATCGACAGCATCCAGCGAGGTAAAGAGGGCGTGTGCTTTCCTCCTCTCTCGTCAGATGG
AAGACGGAGGATGGGGAGAGGAGTTTGAATCTTGTGAAGAACGTCGCTACATCCAGAGTAAAACCTCGCAGGTTG
TCAACACTGCATGGTCTGTACTGGCACTCATGGCTGTCAGATTTCCAGAGCGTGAAGTCATCGATCGAGGCATTC
GGGTCATCAGAGACAGGCAGCTGCCGAACGGGGATTGGGCTCAGGAGAGTATAAAAGGCGTGTTCAACAAGTCAT
GTGCAATCAGCTACACCAGCTACCGCAACCTGTTCCCAATATGGGCTCTGGGTCGATATGCAAGGATCTACGGTG
ACGATGCATAGAGGTATAGGGATGTACAATCATGTACCCTTAGCCAAAGCTATGGCTGTTAAGATATCATTCAGG
GGTCGATTTCACAAAGGGTTAGGACCTGTCTTATCTCGAGTTTGGCAGGACTAGCCTTAAGTTTTTAAT
>gi|638932906|gb|GAVC01057989.1|
CTGTGTCCGAATTGGCAGCTATGGATGTTGCAGAGTGTTGCTATGAGCACCCTATATGCTTGTTCACATGTCAAT
CACAGCACGACCAAAGAATACATGACCACTACACTCAACAGTAAGCCACATCCATAAGCCACTCCAGGCAGACAC
ACCTCTACGCATGGTCACCATAGATCTGAGCATATCGACCCAAAGCCCATATTGGGAACAGGTTACGGTAGCTGG
TGTAGCTGATTGCACATGACTTGTTGAACACACCTTTTATGTTCTCCTGAGCCCAATCTCCGTTTGGCAGCTGTC
TGTCTCTGATGACCTGGATGCCTCGATCAATGACGTCACGCTCTGGAAATCTGACAGCCATGAGTGCCAGTACAG
ACCAAGCCGTGTTGACAACCTGTGAGGTTTTACTCTGGATGTAGCGACGTTCTTCACAAGATTCAAACTCTTCTC
CCCAACCTCCATCTTCCATCTGACGAGAGAGGAGGAAAGCACATGCCCTCTTTACCTCGCTGGATGCCGTCGATG
TCTCATAGCTGTATCCCATACAGGCGTAGGCTTCCAAACCAAACCAGGCAGCATAAGTGAAGCAAACACCCCAGG
AACCTTCCCACGATCCATCCCGCCTCTGTTTAAGAGCTATGTACAACAGTCCACGGTCCAGTGTAGCTCGGATTT

CAGCTTGACGGTACTCTGGATAAATGTCACTGAACTTTTTGAGCGATTGCATGACGGCCGAGGTGAGTTCTACAT
AGGTATAGTCTATCATAATGTCACCAAAGACTTCCGATGGATTGAGTTTTTCAAGAATAATACCCCCTCGCATGG
TTTCGTAAGTTGCAAATCCACCGTCACTATTTCGCATGTTCAGCATAACATCCACAGCTTCTTGGTGTCTCTCTT
GAGTGATGTGGTCCTTGAGAAAAGGACAGTGCTGTTCAATCATTAGTGCAGACTTGAGTCCCTCAGCAGTGCAGT
CAGCAACGATCCAACCGCAGTCACGTGTGCTGAATGGGTACCCACCCTTGTTCATCTGCCTGTAGTACTTCTGAT
AATTGGGAGGGTTTTCTGGAATCTGGGTGAGTTTGAAGAAATCATGAGTCCTCTGAAGGCACTGGTGGAGTGATT
CGTTCTTTGCAGCTCCTCCCTCCAAGAAGGCCTGAGCAGCAAACGCTGTGTCCCACAACTGGGAGCCATTAGTGC
CCTGCATTTTCATTCCATCCAAACCAATCCATAAATAATCAGAAATACGCTCAACATGTTGTTTGAAGATCTCGG
AGTCTGGTCCGTCTGTAATCCATCGCACTAACATGTTGATTACTTTGGAGATAGGTCCAATACTGATGCATTGAG
TGAACTCGTCATCAGCTTTGATGTGGTCTAGGCATTCCTCTAGCGCCCTCTGGCGCCCTTTAGCAATATGGTACT
TCTCATATTTATCTAAGCAACCAAATGTTAAGCTATACAGCCAGCTATGGGGCGTGTACAAGTCCACTTTGGCTA
TGTTGCTCCGCTGTGCCGGCCAATCAATGGTGTAATAATCCTCCACATAAAGCTCCATCCTGAGGTCCATGATCA
AGTCATCTGGTTCAGCCTGAAACTTGATCCCATAGCAGTAACTCATCGGCAGGTACACCTGTCTGCAATGGCACC
AGATGGTAGAAGGATGTGCTGGAAAAAATGTTGGAAAGTTCCAGATCTCAGGAAAGAGGGTGTGCATTCCATCCC
ACTCATACACATTCAGAACCGACAGCCAGAACTTACCCCAAGAAGGGATAGCCGCAGCACCTCCTAAGGAATGAA
GAAGCTTCCTACATTTCATCAGATCTGGATCTTCAGGACCAATACCCAGGAGTCTCATGGCGACGTAGTTGGTTG
CACAGCCAAAGACCGTAGGCGGGCCTTCAACATGTAAACCCCAACCACCATCTTGACACTGAACAGAGCGCAGGT
ACCGGACCATCTCCTTCTTAACTGCATCTGGCAATTCAGTCTTTGTGATGTAGCAGACTGTCAGAAGACCTGGCA
TAAGGAAGAGAGGTCCACCGTAGTCTCCGCTCCAGTGGCCATCTTCAGTTTGAAGCTTGGAGTAAAACAGCATAC
CATTAGTTGCTGCTTCTTGGGCTGTGGCTGGCTTGGCCAGGGGCGGTGCCTCCTCACTAATATCTAGGCCCAAGC
TAAACTTCTCAACAAAGTTTTGCTCTCTGTCCACTGCTTCACCTTCTTCAATGAAACGCCATGTCTGCCTGCCAT
CTACATTAGAGAGGCGCCACTTTGTGAGATCAGTGGCTGGCTCTGTTTTGTGAGGACCTCCTCGGTTCCTTCGGC
CGCTCATGTTTGGTTAACTTTGGCCTGTATTTATATGCCTCCCAGGCTCGGTGGAGCAATCCCACAAACTATTTG
GATTTGGAGATCAGCAATACGAAACTATACACTAGTGAATTATCTGGTCAAGGTGAATAGACCGTATCGAATATG
ATGACGTTACCTTTCAATAATACCCACAACATG
>gi|638932902|gb|GAVC01057992.1|
CTGTGTCCGAATTGGCAGCTATGGATGTTGCAGAGTGTTGCTATGAGCACCCTATATGCTTGTTCACATGTCAAT
CACAGCACGACCAAAGAATACATGACCACTACACTCAACAGTAAGCCACATCCATAAGCCACTCCAGGCAGACAC
ACCTCTACGCATGGTCACCATAGATCTGAGCATATCGACCCAAAGCCCATATTGGGAACAGGTTACGGTAGCTGG
TGTAGCTGATTGCACATGACTTGTTGAACACACCTTTTATGTTCTCCTGAGCCCAATCTCCGTTTGGCAGCTGTC
TGTCTCTGATGACCTGGATGCCTCGATCAATGACGTCACGCTCTGGAAATCTGACAGCCATGAGTGCCAGTACAG
ACCAAGCCGTGTTGACAACCTGTGAGGTTTTACTCTGGATGTAGCGACGTTCTTCACAAGATTCAAACTCTTCTC
CCCAACCTCCATCTTCCATCTGACGAGAGAGGAGGAAAGCACATGCCCTCTTTACCTCGCTGGATGCCGTCGATG
TCTCATAGCTGTATCCCATACAGGCGTAGGCTTCCAAACCAAACCAGGCAGCATAAGTGAAGCAAACACCCCAGG
AACCTTCCCACGATCCATCCCGCCTCTGTTTAAGAGCTATGTACAACAGTCCACGGTCCAGTGTAGCTCGGATTT
CAGCTTGACGGTACTCTGGATAAATGTCACTGAACTTTTTGAGCGATTGCATGACGGCCGAGGTGAGTTCTACAT
AGGTATAGTCTATCATAATGTCACAACATCCACAGCTTCTTGGTGTCTCTCTTGAGTGATGTGGTCCTTGAGAAA
AGGACAGTGCTGTTCAATCATTAGTGCAGACTTGAGTCCCTCAGCAGTGCAGTCAGCAACGATCCAACCGCAGTC
ACGTGTGCTGAATGGGTACCCACCCTTGTTCATCTGCCTGTAGTACTTCTGATAATTGGGAGGGTTTTCTGGAAT
CTGGGTGAGTTTGAAGAAATCATGAGTCCTCTGAAGGCACTGGTGGAGTGATTCGTTCTTTGCAGCTCCTCCCTC
CAAGAAGGCCTGAGCAGCAAACGCTGTGTCCCACAACTGGGAGCCATTAGTGCCCTGCATTTTCATTCCATCCAA
ACCAATCCATAAATAATCAGAAATACGCTCAACATGTTGTTTGAAGATCTCGGAGTCTGGTCCGTCTGTAATCCA
TCGCACTAACATGTTGATTACTTTGGAGATAGGTCCAATACTGATGCATTGAGTGAACTCGTCATCAGCTTTGAT
GTGGTCTAGGCATTCCTCTAGCGCCCTCTGGCGCCCTTTAGCAATATGGTACTTCTCATATTTATCTAAGCAACC
AAATGTTAAGCTATACAGCCAGCTATGGGGCGTGTACAAGTCCACTTTGGCTATGTTGCTCCGCTGTGCCGGCCA
ATCAATGGTGTAATAATCCTCCACATAAAGCTCCATCCTGAGGTCCATGATCAAGTCATCTGGTTCAGCCTGAAA
CTTGATCCCATAGCAGTAACTCATCGGCAGGTACACCTGTCTGCAATGGCACCAGATGGTAGAAGGATGTGCTGG
AAAAAATGTTGGAAAGTTCCAGATCTCAGGAAAGAGGGTGTGCATTCCATCCCACTCATACACATTCAGAACCGA
CAGCCAGAACTTACCCCAAGAAGGGATAGCCGCAGCACCTCCTAAGGAATGAAGAAGCTTCCTACATTTCATCAG
ATCTGGATCTTCAGGACCAATACCCAGGAGTCTCATGGCGACGTAGTTGGTTGCACAGCCAAAGACCGTAGGCGG
GCCTTCAACATGTAAACCCCAACCACCATCTTGACACTGAACAGAGCGCAGGTACCGGACCATCTCCTTCTTAAC
TGCATCTGGCAATTCAGTCTTTGTGATGTAGCAGACTGTCAGAAGACCTGGCATAAGGAAGAGAGGTCCACCGTA
GTCTCCGCTCCAGTGGCCATCTTCAGTTTGAAGCTTGGAGTAAAACAGCATACCATTAGTTGCTGCTTCTTGGGC
TGTGGCTGGCTTGGCCAGGGGCGGTGCCTCCTCACTAATATCTAGGCCCAAGCTAAACTTCTCAACAAAGTTTTG
CTCTCTGTCCACTGCTTCACCTTCTTCAATGAAACGCCATGTCTGCCTGCCATCTACATTAGAGAGGCGCCACTT
TGTGAGATCAGTGGCTGGCTCTGTTTTGTGAGGACCTCCTCGGTTCCTTCGGCCGCTCATGTTTGGTTAACTTTG
GCCTGTATTTATATGCCTCCCAGGCTCGGTGGAGCAATCCCACAAACTATTTGGATTTGGAGATCAGCAATACGA
AACTATACACTAGTGAATTATCTGGTCAAGGTGAATAGACCGTATCGAATATGATGACGTTACCTTTCAATAATA
CCCACAACATG
>gi|638932900|gb|GAVC01057993.1|

CTGTGTCCGAATTGGCAGCTATGGATGTTGCAGAGTGTTGCTATGAGCACCCTATATGCTTGTTCACATGTCAAT
CACAGCACGACCAAAGAATACATGACCACTACACTCAACAGTAAGCCACATCCATAAGCCACTCCAGGCAGACAC
ACCTCTACGCATGGTCACCATAGATCTGAGCATATCGACCCAAAGCCCATATTGGGAACAGGTTACGGTAGCTGG
TGTAGCTGATTGCACATGACTTGTTGAACACACCTTTTATGTTCTCCTGAGCCCAATCTCCGTTTGGCAGCTGTC
TGTCTCTGATGACCTGGATGCCTCGATCAATGACGTCACGCTCTGGAAATCTGACAGCCATGAGTGCCAGTACAG
ACCAAGCCGTGTTGACAACCTGTGAGGTTTTACTCTGGATGTAGCGACGTTCTTCACAAGATTCAAACTCTTCTC
CCCAACCTCCATCTTCCATCTGACGAGAGAGGAGGAAAGCACATGCCCTCTTTACCTCGCTGGATGCCGTCGATG
TCTCATAGCTGTATCCCATACAGGCGTAGGCTTCCAAACCAAACCAGGCAGCATAAGTGAAGCAAACACCCCAGG
AACCTTCCCACGATCCATCCCGCCTCTGTTTAAGAGCTATGTACAACAGTCCACGGTCCAGTGTAGCTCGGATTT
CAGCTTGACGGTACTCTGGATAAATGTCACTGAACTTTTTGAGCGATTGCATGACGGCCGAGGTGAGTTCTACAT
AGGTATAGTCTATCATAATGTCACCAAAGACTTCCGATGGATTGAGTTTTTCAAGAATAATACCCCCTCGCATGG
TTTCGTAAGTTGCAAATCCACCGTCACTATTTCGCATGTTCAGCATAACATCCACAGCTTCTTGGTGTCTCTCTT
GAGTGATGTGGTCCTTGAGAAAAGGACAGTGCTGTTCAATCATTAGTGCAGACTTGAGTCCCTCAGCAGTGCAGT
CAGCAACGATCCAACCGCAGTCACGTGTGCTGAATGGGTACCCACCCTTGTTCATCTGCCTGTAGTACTTCTGAT
AATTGGGAGGGTTTTCTGGAATCTGGGTGAGTTTGAAGAAATCATGAGTCCTCTGAAGGCACTGGTGGAGTGATT
CGTTCTTTGCAGCTCCTCCCTCCAAGAAGGCCTGAGCAGCAAACGCTGTGTCCCACAACTGGGAGCCATTAGTGC
CCTGCATTTTCATTCCATCCAAACCAATCCATAAATAATCAGAAATACGCTCAACATGTTGTTTGAAGATCTCGG
AGTCTGGTCCGTCTGTAATCCATCGCACTAACATGTTGATTACTTTGGAGATCTTTCCGTTGACTATCTACAAGT
CTGCATCTGTAAATGTACCAAATATACCCCTTCTGTAGTCTGATTTCTGCGTAAAGTCATTTGCAACCTCTGTTA
AACTCAGCTTGTCGACTATTTCTTTGTGGATGTGTAATACCATATAGTTATTGAGTCG
>gi|638682784|gb|GAWB01045303.1|
TGGAGATACATCGAGGTGGCGAACAGAATATATGGACTTTGCCTTTTTGCTGTGTCTGGTTCGTTTCAAGAGATG
AAATAACACATTTTTGGGGTGCGGACTACAGAATAACGATTATCACTATGAGCGGCCGACGCAACAGAGGAGGTC
CACACAAGACAGAGCCGGTCACTGACCTGACAAGATGGCGTCTGTCCAACGTGGACGGCCGACAAACATGGAGAT
ACATCGAGGAAGGAGAACCCATCGAAAGAGAGCAGAACTTGGTGGAAAAGTTCAGCTTAGGACTGGATATTAGCA
AAGAAGCCCCGCCCTTCCCCAAACCCACCACACCCCAAGAGGCAGCCACCAATGGGATGGTCTTCTACTCAAGGC
TCCAGACGGAAGACGGCCACTGGAGCGGAGACTATGGGGGGCCTCTCTTCCTCATGCCAGGACTGCTTATTGTGT
GCTACATCACCAAGACAGAATTGCAGGATGCCGTCAAGAAGGAAATGGTGCGATATCTGCGCTCCGTCCAGTGTC
AAGATGGCGGATGGGGGCTACATATCGAAGGCCCGCCCACAGTCTTTGGTTGCGCCACTAACTACGTCGCGATGA
GACTGCTGGGCGTTGCCGCCGACGACCCGGACCTCATCAAATGCAGGAAGTTGCTTCATTCGCTAGGAGGAGCCG
CGGCCATTCCGTCCTGGGGCAAGTTCTGGCTGTCGGTCCTCAATGTCTACAGCTGGGATGGTATGCATACACTCT
TCCCTGAACTCTGGCTGTTTCCGACCTGGATCCCCGCCCATCCGTCCACCATCTGGTGCCACTGCAGACAAGTCT
ATCTACCCATGAGTTACTGCTATGGCATCAAGTATCAAGCTGAAGAAGATGATCTGATCAGGGAACTGAGAAAGG
AACTCTATACTCAAGACTACCATACCATCGACTGGCCCGCACAGAGGGATAACATAGCCAAGGTGGATCTGTACA
CCCCTCACAGCTGGCTCTACAATCTGGCGTTTGGTTTCCTGGATGTCTATGAGCCCTATCATTCCACTGCCTTCC
GTCAGAGGGCTCTTGACGAATGCCTTGATCACATCCGAGCTGACGATCGCTTCACCAAGTGCATTAGTATTGGAC
CTATATCAAAAGTCATCAACATGTTGGTCAGATGGATTACTGAAGGAGCAGACTCGGAGGTCTTCAAGCAGCACG
TGGAACGCATCCCGGATTACCTATGGATTGGCCTGGATGGCATGAAAATGCAGGGAACCAACGGTTCACAGCTGT
GGGACACGGCCTTCGCCGCCCAGGCTTTCTTAGAAGCAGGAGCAGCAACCAACAAGGAGCTTCACGAATGCTTAC
AGCGAACCCACGACTTCCTGAAACTCACTCAGATACCCGACAATCCGCCCAACTATCAAAAGTACTACCGACAAA
TGAACAAGGGGGGTTATCCATTCAGTACCCGCGACTGCGGCTGGATTGTGGCCGACTGTACAGCCGAGGGGCTCA
AGGCAGCGTTGATGCTGGAGACAATGTGCCCCACCGTCAAAGATCACATCGAGAAAGATAGGCACTACGAAGCCG
TGGATGTGATACTGAATATGCGAAACAAAGATGGCGGATTTGCCACCTATGAGACCATGAGAGGAGGTATCATCC
TTGAAAAACTCAACCCTTCCGAAGTTTTTGGCGATATCATGATTGACTACACCTACGTTGAGCTGACGTCGGCCG
TGATGCAATGCCTGAAGAAATTCAGTGAGCTGTATCCTGAGTATCGCAAGGACGAGGTCAGATCCACGCTGGACA
GGGGCCTGTTGTACAT
>gi|638577308|gb|GAVK01073940.1|
TGCGTGCGCGAGGAGAATATATGGACTTTGCCTTTTTGCTGTGTCTGGTTCGTTTCAAGAGATGAAATAACACAT
TTTTGGGGTGCGGACTACAGAATAACGATTATCACTATGAGCGGCCGACGCAACAGAGGAGGTCCACACAAGACA
GAGCCGGTCACTGACCTGACGCGATGGCGTCTGTCCAACGTGGACGGCCGACAAACATGGAGATACATCGAGGAA
GGCGAACCCATCGAAAGAGAACAGAACTTGGTGGAGAAGTTCAGCTTAGGATTGGATATTAGCAAAGAAGCCCCG
CCCTTCCCCAAACCCACCACGCCCCAAGAGGCAGCCACCAATGGGATGGTCTTCTACTCAAGGCTCCAGACGGAA
GACGGCCACTGGAGCGGAGACTATGGGGGGCCTCTCTTCCTCATGCCAGGACTGCTTATTGTGTGCTACATCACC
AAGACAGAATTGCAGGATGCCGTCAAGAAGGAAATGGTGCGATATCTGCGCTCCGTTCAGTGTCAAGATGGCGGA
TGGGGGCTACATATTGAAGGCCCGGCTACAGTCTTTGGTTGCGCCACTAACTACGTCGCGATGAGGCTGCTGGGT
GTTGCCGCCGACGACCCGGACCTCATCAAATGCAGGAAGTTGCTTCATTCGCTAGGAGGAGCTGCGGCCATTCCA
TCCTGGGGCAAGTTCTGGTTGTCGGTTCTCAATGTCTACAGCTGGGACGGTATGCATACGCTCTTCCCTGAACTC
TGGCTGTTTCCGACCTGGATCCCCGCCCATCCGTCCACTATCTGGTGCCACTGCAGACAAGTCTATCTACCCATG
AGTTACTGCTATGGCATCAAGTATCAAGCTGAAGAAGATGAGCTGATCAGGGAACTAAGAAAGGAACTCTATACT
CAAGACTACCATACCATCGACTGGCCAGCACAGAGGGATAACATAGCCAAGGTGGATCTGTACACACCTCACAGC
TGGCTCTACAATCTGGCGTTTGGTTTCCTGGATGTCTATGAGCCCTATCATTCCACCGCCTTCCGTCAGAGGGCT

CTTGACGAGTGCCTGGATCACATCCGAGCTGACGATCGCTTCACCAAGTGCATTAGTATTGGACCTATATCCAAG
GTCATCAACATGTTGGTCAGATGGATTACTGAAGGAGCAGACTCGGAGATCTTCAAGCAGCATGTGGAACGCATC
CCGGATTACCTATGGATTGGTCTAGATGGCATGAAAATGCAGGGAACCAACGGTTCACAGCTGTGGGACACGGCC
TTCACTGCCCAGGCTTTCTTAGAAGCAGGAGCAGCCACAAACAAGGAGCTACACGAATGCTTACAACGAACCCAC
GACTTCCTGAAGCTCACTCAGATACCGGACAATCCGCCCAACTATCAAAAGTACTACCGACAAATGAACAAGGGG
GGTTATCCATTTAGTACCCGCG
>gi|638344845|gb|GAVI01039758.1|
GAATTTTCCAACATTTTTTCCAGCACATCCTTCTACCATCTGGTGCCATTGCAGACAGGTGTACCTGCCGATGAG
TTACTGCTATGGGATCAAATTTCAGGCTGAACCAGATGACTTGATCATGGATCTCAGGATGGAGCTGTATGTGGA
GGATTATTACACCATTAATTGGCCGGCACAAAGGAGCAACATAGCCAAAGTTGACTTGTACACGCCCCATAGCTG
GCTGTATAACTTGGCGTTTGGTTGCTTAGATATATATGAAAAGTACCATTTTGCTGATTGGCGCCAGAGGGCACT
AGACGAATGCCTCGACCACATCAAAGCTGACGACAAGTTCACTAAATGCATCAGTATTGGACCTATCTCAAAAGT
GATCAACATGTTGGTGCGATGGATTACAGATGGACCGGACTCAGAGATCTTCAAACAACATGTTGAGCGCATCCC
TGATTATTTATGGATTGGCTTGGATGGAATGAAAATGCAGGGTACCAATGGCTCCCAGTTGTGGGACACGGCATT
TGCTGCTCAGGCCTTCCTAGAGGGAGGAGCTTCAAAGATCGAATCACTCCATCAGAGCCTTCAGAGAACCCACGA
TTTCTTCAAACTCACTCAGATTCCAGAAAACCCTCCGAATTATCAGAAGTACTACAGGCAAATGAACAAGGGTGG
TTATCCGTTCAGCACACGTGACTGTGGTTGGATCGTTGCTGACTGCACAGCGGAGGGACTCAAGTCTGCTCTGAT
GATTGAACAGCTCTGTCCCTTCCTCAAGGACCACATCACCCAAGAGAGACACCAAGAAGCTGTGGACGTTATGCT
GAACATGCGAAATAGCGATGGCGGATTTGCAACTTACGAGACAATGCGAGGGGGTGTTATTCTTGAAAAACTCAA
TCCATCGGAGGTTTTTGGAGACATCATGATAGATTACACCTACGTAGAACTCACCTCGGCCGTCATGCAATCTCT
CAAGAAGTTCAGTGACATTTATCCCGACTACAGACAAGCTGAAATCAGAGCTACACTGGACCGTGGACTGTTGTA
CATCGCTCGTAAACAAAGAACTGACGGATCGTGGGAAGGTTCCTGGGGTGTTTGCTTCACTTATGCTGCCTGGTT
TGGTCTTGAAGCCTACGCCTGCATGGGATACAGCTATGAAACATCGACAGCATCCAGCGAGGTAAAGAGGGCGTG
TGCTTTTCTCCTCTCTCGTCAGATGGAAGACGGAGGTTGGGGAGAGGAGTTTGAATCTTGCGAAGAACGTCGGTA
CATCCAGAGTAAAACTTCACAGGTTGTCAACACGGCATGGGCTGTACTGGCTCTCATGGCTGTCAGATTTCCAGA
GCGTGAAGTCATCGATCGAGGCATCAGGGTGATCAGAGACAGACAGCTTTCGAATGGGGATTGGGCTCAGGAGAA
TATTAAAGGTGTGTTCAACAAGTCATGTGCAATCAGCTATACCAGCTATCGTAACCTGTTCCCAATATGGGCTTT
GGGTCGTTATGCACGGATCTATGGTGACGATGCATAGGGTGTGTCTGCAAGGGGTGGCTTTGGCTGTGGCTATGG
CTGTGGCTGAAGTATAGAAATTTACAGTCATGTATGCTAAGCCAAAGCCATATCTATTATTGAGTGGCTGTGGCT
AGACGAGCTGTCAAATGATGATTGAGTGACATTGGCTGTTGGAAGTAGCGCCTATGTTGGAATTTACAGTCATAT
ATCCTCAGCCAAAGCCAAGGTTGTTAATAAATCATTCAGGGCCTTGAGTATATTATATTATTATTTTGTGAAGTA
TTTGGAATTGCCTCAGTTACTCTTACAGAGATGTGTTTTTAACAAATCAACTCCAAAATGTTGGCAAAAATATGA
AAAAAATGTGAGGCTTGCTGCCCCTTGTTATTTTCACAGGGCCCCGCCCTGTAAATAATTATAATTCTTTATGCC
AGGGTGGTACATGTATTTAAAAAAACAAATCTAAGCTTGGTTTAAAATGGCATTTAAATATTTTAAATAGCTG
>gi|638493931|gb|GAVJ01039023.1|
CATTCGGTTGCCTAGATATATATGAGAAGTACCATAGTGCTAAATGGCGGCAGAGTGCGCTAGACGAGTGCCTTG
ACCACATCAAAGCTGACGATGAGTTCACTCAATGCATCAGTATCGGACCTATCTCCAAAGTGATCAACATGTTGG
TGCGATGGATGACAGACGGACCGGACTCGGCCATATTCAAACAACATGTTGAGCGTATTCCTGATTATTTATGGA
TCGGTTTGGATGGAATGAAAATGCAGGGCACCAATGGCTCCCAGTTGTGGGACACAGCGTTTGCCGCCCAGGCCT
TCCTAGAGGGAGGGGCTGCAAAGAACGAGTCACTCCATGAATGCCTTCAAAGGACTCATGATTTCTTCAAACTCA
CCCAGATCCCAGAAAACCCACCAAATTATCAGAAGTACTACAGGCAGATGAACAAGGGTGGGTACCCATTCAGCA
CGCGTGACTGCGGTTGGATCGTTGCTGACTGTACTGCAGAGGGACTCAAGTCTGCGCTGATGATTGAGCAGCGAT
GTCCATTTCTCAAGGACCACATCTCACAAGAAAGACACCAAGAAGCTGTGGATGTTATGCTGAACATGCGAAATA
GCGACGGCGGATTTGCAACGTACGAGACCATGCGAGGGGGCGTAATTCTTGAGAAACTTAATCCATCAGAAGTCT
TTGGTGACATCATGATAGACTACACCTACGTAGAACTCACCTCGGCTGTCATGCAATCACTCAAGAAGTTCAGCG
ACATTTATCCTGATTATCGCCAAGCGGAAATCCGAGCTACACTGGACCGTGGGCTGTTGTACATCGCTGATAAAC
AGAAGCGGGATGGATCATGGGAAGGTTCCTGGGGTGTTTGCTTCACTTATGCCGCCTGGTTTGGTCTGGAAGCCT
ACGCCTGCATGGGATACAGCTACGAAACATCGACGGCATCCAGCGAGGTAAAGAGGGCGTGTGCTTTCCTCCTCT
CTCGTCAGATGGAAGATGGAGGATGGGGCGAGGAGTTTGAATCTTGTGAAGAACGTCGCTACATACAGAGTAAAA
CCTCGCAGGTTGTCAACACGGCATGGTCTGTACTGGCACTCATGGCTGTCAGATTTCCAGAGCGTGAAGTCATCG
ATCGAGGCATTCGGGTCATCAGAGACAGGCAGCTGCCGAACGGGGATTGGGCTCAGGAGAGTATAAAAGGCGTGT
TCAACAAGTCATGTGCAATCAGCTACACCAGCTACCGCAACCTGTTCCCAATATGGGCTCTGGGTCGATATGCAA
GGATCTACTGTGACGATGCATAGAGGTATAGGGATGTACAATCATGTACCCTTAGCTAAAGCTATGGCTTGTTAG
AAATCATTCAGGGGGCGATTTCACAAAGAGT
>gi|638493930|gb|GAVJ01039024.1|
TCATATATATCTAGGCAACCGAATGCTAAGTTATACAGCCAACTATGGGGCGTGTACAAGTCCACTTGTGCTATG
TTGCTTCGCTGTGCTGGCCAATTGATGGTGTAATAGTCCTCCACGTAAAGCTCCATCCTGAGATCCATGATCAAG
TCATCTGGTTCAGCCTGGAATTTGATCCCATAGCAGTAACTCATCGGCAGGTAAACCTGTCTGCAATGGCACCAG
ATGGTAGAAGGATGAGCGGGAGTATACGTTGGAAAATTCCAGAGCTCAGGAAAGAGGGTGTGCATTCCATCCCAC
TGATAAACATTCAGAACCGACAGCCAGAACTTGCCCCAAGAAGGTATAGCCGCAGCACCTCCTAAGGAATGAAGA
AGCTTCCTACATTTCATCAGGTCTGGATCTTCTGGACCAATACCAAGGAGTCTCATAGCGACATAGTTGGTTGCG

CAACCAAAGACCGTAGGCGGGCCTTCAACATGTAAACCCCATCCGCCATCTTGACACTGAACAGAGCGTAGGTAC
CGGACCATCTCCTTCTTAACTGCATCTGGTAGTTCAGTCTTTGTGATGTAGCAGACTATAAGTAGACCTGGCATA
AGGAAGAGAGGTCCCCCATAGTCTCCACTCCAGTGGCCGTCTTCTGTTTGAAGCTTGGAGTAAAACAGCATGCCA
TTAGTCGCTGCTTCTTGGGCTGTGGCTGGTTTGGCCAAGGGTGGTGCCTCTTCACTAATGTCTAAGCCCAAGCTA
AACTTCTCAACAAAGTTTTGCTCTCTGTCCACTGGTTCACCCTCCTCGATGAAGCGCCAGGTCTGCCGTCCATCG
ACATTAGAGAGGCGCCATCTTGTGAGATCAGTAGCTGGCTCTGTTTTGTGGGGACCTCCTCGGTTTCGTCGGCCG
CTCATGTTGGGTCCAACTTTTGAATCTTCTCTGCAATAAAACCTCTAGAGATGTCTATTTATCAGCTCATAAAAG
TCGACAAAGTAAGGCCAGCAACACAACTCGTTCAAAGAGTTTACTTCAAAACTGCATGCACGACCTGCATGATGC
GTATGGATCATTAGATGGGGATGACATTCAGCAACTCAAATTGTGTACACGACCTTTGATCTTTGAACTATAAAT
AAACAAACCAAAAAGCTCCACCTACATGCAGTTCTTGCTGTTGGAATTGTTCTTTATTAAAGTATTTTTGTTACG
ATTTCGCAGCCTTTTTGTCGTCAACTTTGATTAAAAACTACACTACGGAATTAGTTTCATTGTCACAGCTGTTCA
CACAAGATAAGAACTTGAATTGGTTCCATATTACTCATTTTTACACATTATTTCCTGTCTCCTGTGTGCAGAAGA
AGCTGCAGTCCCTGGGTCAAAAAAG
>gi|638493932|gb|GAVJ01039022.1|
TCATATATATCTAGGCAACCGAATGCTAAGTTATACAGCCAACTATGGGGCGTGTACAAGTCCACTTGTGCTATG
TTGCTTCGCTGTGCTGGCCAATTGATGGTGTAATAGTCCTCCACGTAAAGCTCCATCCTGAGATCCATGATCAAG
TCATCTGGTTCAGCCTGGAATTTGATCCCATAGCAGTAACTCATCGGCAGGTAAACCTGTCTGCAATGGCACCAG
ATGGTAGAAGGATGAGCGGGAGTATACGTTGGAAAATTCCAGAGCTCAGGAAAGAGGGTGTGCATTCCATCCCAC
TGATAAACATTCAGAACCGACAGCCAGAACTTGCCCCAAGAAGGTATAGCCGCAGCACCTCCTAAGGAATGAAGA
AGCTTCCTACATTTCATCAGGTCTGGATCTTCTGGACCAATACCAAGGAGTCTCATAGCGACATAGTTGGTTGCG
CAACCAAAGACCGTAGGCGGGCCTTCAACATGTAAACCCCATCCGCCATCTTGACACTGAACAGAGCGTAGGTAC
CGGACCATCTCCTTCTTAACTGCATCTGGTAGTTCAGTCTTTGTGATGTAGCAGACTATAAGTAGACCTGGCATA
AGGAAGAGAGGTCCCCCATAGTCTCCACTCCAGTGGCCGTCTTCTGTTTGAAGCTTGGAGTAAAACAGCATGCCA
TTAGTCGCTGCTTCTTGGGCTGTGGCTGGTTTGGCCAAGGGTGGTGCCTCTTCACTAATGTCTAAGCCCAAGCTA
AACTTCTCAACAAAGTTTTGCTCTCTGTCCACTGGTTCACCCTCCTCGATGAAGCGCCAGGTCTGCCGTCCATCG
ACATTAGAGAGGCGCCATCTTGTGAGATCAGTAGCTGGCTCTGTTTTGTGGGGACCTCCTCGGTTTCGTCGGCCG
CTCATGTTGGGTCCAACTTTTGAATCTTCTCTGCAATAAAACCTCTAGAGATGTCTATTTATCAGCTCATAAAAG
TCGACAAAGTAAGGCCAGCAACACAACTCGTTCAAAGAGTTTACTTCAAAACTGCATGCACGACATGCAGTTCTT
GCTGTTGGAATTGTTCTTTATTAAAGTATTTTTGTTACGATTTCGCAGCCTTTTTGTCGTCAACTTTGATTAAAA
ACTACACTACGGAATTAGTTTCATTGTCACAGCTGTTCACACAAGATAAGAACTTGAATTGGTTCCATATTACTC
ATTTTTACACATTATTTCCTGTCTCCTGTGTGCAGAAGAAGCTGCAGTCCCTGGGTCAAAAAAG
>gi|638027500|gb|GAVF01081824.1|
CTGGCCCCATATTCATAAAACCCCACTAGCTTAGTTCCATGCTTAGGCTATTTTGGAAAGCAACGAGTAGTCTTC
TCACTAGGGTTAGTTGGAGTCACATTTGTTACTACCCAGGAACTCTAGTTGGTAACTAAAATGACCACTAGTTGC
ATACTTTGTCCTATCATTCAAAACCGACTTAGACATGAAACTAAGCTAGCTGGGTATCATGAGTATCGGCCCTTG
CATGATCTATTTTTAAAAGTTCCAATCAACACTACTACATTTGCTCTTAAGTGTTGTTTGGAACATACTTCAAAT
GTTAACTTTGACACAAAGAGAATAATATAGTATACAATTGTATACATGAGACTATTTTATTGATATTTTTTTGTA
TTTTGTTTATTTTTACCTAAATAGAGAGTAATGCTGTTGTTCATGTCAGATTTAGAGCAGTTTGGAATTATGTTT
ATCGGTGTTCCTTCATTCAGTGCACATTGTGAACATTTTATTAGTGGTAACATTTTATATGGTTTCAATTGTCTT
TTTAAAAGTGAGTTTAAAATTTACGAAAATGTATTTCCAAAATGAGATAATTTACGTTTTTAGAGAAAAAAAGAG
AGAAGTAAATGTGTACATGATTTATTCAATAGTTTTTGATGAATTTGGAAGACAGATATGTACATAAATTTAAAT
GTACATGCATACTCCTGACTAGGTGTCATTTATGAATGGGGAGGGCGTGATAGGCAGTTCAGATTTTGATGCATA
TATTTGGGTTTGGTGTTCAATTTTTGTTAAATGAAAGTAAACTGCTGAAAACAAAACGCAACATACAGTTACGCA
CAAAGTGATTAAAAGAGATGCGATGCATTTAGTAGTGATATTACAATTTCCATGTCCTTGTGGGATAAGGATATT
GTAATTGATTCAATTTTGATTGAAAATATTATGAATTATGCAATCTGACTCGCGTTGAGTGTATTCATGTATTAC
CTCATATTGAGTGTCGGGTACTTTTTCATCCCAAAGATTTCAAACAAAGATAATGTGGTAATAATAATACTACTA
ATAATAATAATTATACATTTATATCGCGCGCACATACTAAGATAATAACACAATGAAATATATACATACTACATT
ACAACAAAGATATCAATACATATATCATAAATATCAGTCATAATAATACTCATGGACATGAACTTTTGTAGGTAC
ATTTATATTTGAATATGAATAAGAAAAAAAATAATGGAAAAAACGAATAATAAGAAGACTATTTGTAATCGATCG
ATCACATGTTATGATCGTTCATGATCGATCACAAACCGGCTATCGCGCGTTACGTCCGACCCCTTCTCGTCGCGT
CGGATGTCGACACCGATATCGTGACGCGAGAACCATGTCGTACAAGAAGAATTTTGGTGGGCCTTACAAGACTGA
TCCAGCTACTGACCTCACCCGATGGCGTCTGACCAATGTCAATGGCCGGCAGACATGGCGGTACATACCGGAGGG
AGAGGAGCCCGACCGGCCACAGAACTTTGTGGAGAAATTTGCTCTTGGATTGAGCATAGATGAGTTGACCCCGCC
TCTCTCAAAGGCAAAGACGGCGAAGGAAGCAGCCAAGAATGGGATGCGATTCTACTCCAAACTTCAGACCGAGGA
TGGCCATTGGTCTGGTGATTACGGAGGACCTCTCTTCCTCCTTCCAGGCCTGATCATTGTGTGTTACATCACAGG
AGTGGTTCTCCCCGAGGCTTCCAAGAAGGAGATGATTCGTTATCTGAGGTCCGTGCAGTGTCCGGATGGCGGCTG
GGGCCTTCACATTGAAGACCACGCCACAGTGTTTGGGACAGCTATGAACTACGTGACTATGAGACTTCTTGGTGT
ATCGAAAGATGATAAGGATCTAAAAAAGGCCAGAAAACTGCTCATGGAAATGGGTGGAGCAGAGAGTATTCCTTC
TTGGGGCAAGTTCTGGCTGTGTGTGCTCAACCTATACAAATGGGAGGGCATGCACTGTCTGTTTCCAGAAATATG
GCTGTTCCCATCTTGGATGCCAGCCCACCCCTCATCCATCTGGTGCCACTGCAGACAGGTCTATCTTCCGATGGG
CTACTTCTATGGGGCTAAGTTCCAGGCCCAAGAGGATGGCCTCATCAGAGAACTCAGGAACGAGATATTCAAAGA

GGATTTTGCAACCATCAACTGGCCAGCGCAGAGAGATAACATCTCCAAGTATGATCTGTACACTCCGCACAGCTG
GCTTTACACCATTGCCATGGCCATTTTGGATTTCTATGAGAAGTTTCACTCCACCTGGTTGAGACAAAAGGCTCT
GGATTTCTGCTATGATCACATCAAAGCGGATGACACTTTCACAAAGGGCATCAGTATAGGACCGATCTCGAAGGT
GATTCAGATGCTGGTCAGATGGCATGTAGATGGGCCAGAGTCAGATGCCTTCAAGATGCACAGGGATCGTATCCC
AGACTATCTATGGATTGGATTGGATGGGATGAAAATGCAGGGAACAAATGGCTCACAACTCTGGGACACCGCTTT
TGCAGTCCAAGCATTCATTGAGGCTGGGGCAAACACAGAAGAGGAATTCCGCGACACTCTACGCAACGCTCACGG
CTTCCTACGCAACACGCAAATCCCAGACAACCCTCCAGACTACCAGAAGTACTACCGACAAATGAACAAGGGAGG
GTATCCTTTCTCTACCAAAGACTGTGGCTGGATTGTAGCCGATTGCACGGCAGAAGGTCTCAAATCCGCCATGCT
ACTGGAGGAGAAGTGTCCTTTTGTTACTGACCTCATTGGCAAGGAGAGGCACTGTTTGGCTGTCGATGTCTTGAT
AGACATGAGGAATCCCGACGGTGGTTATGCAACGTACGAAACTACCAGAGGAGGTTACATACTGGAAAAGCTCAA
CCCCTCCGAGGTCTTCGGTGATATCATGATCGACTACACCTATGTTGAGTGTACAGCAGCTGCCATGCTTGCATT
GAAGCACTTCCAGGACCAGCACCCAGAGTACAGACGAGATGAAATCAACTCTGTGCTGGATACTGGATTGGATTT
CATCAAGGGAATTCAACGTCCTGATGGATCATGGGAAGGGTCGTGGGGTGTTTGCTTCACCTACGGAGCTTGGTT
TGGTCTGGAGGGACTCGGATGCATGGGAATGCGGTACGATCGCGACACTGCTACGCCAGAAGTGAAGAAAGCGTG
TGCGTTCCTGGTCTCTAAGCAGATGGCGGACGGTGGTTGGGGGGAGAAGTTTGAATCCTGCGAGACGAGGACGTA
CGTGCAGAGCGAGACATCCCAAGGCGTCCAAACCTGTTGGGCATTGATGGCGCTTATGGCAGTCAGGTATCCCGA
TCGCACAGTGATTGACCGTGGTATCAAGCTGATCATGGACACACAACTAGAGAACGGGGACTGGCCTCAGGCGAA
CATCATGGGGGTGTTCAACAAGTCTTGCGCCATCAGCTACACCAGCTACCGCAATGTCTTCCCCATCTGGTGTCT
GGGGCGCTACGCCAAACTCTACCCCACCCCGAGCCCCGACTCCACCCCTTCTGCAAGCACAGCCAGCACACCACT
GTCAGATAGGTCGGACAGCAACGAAGAAGGCTGGGAGAAATTGTAACGGGTTTGACATCACCTTGATAACCAATC
CTACGAGGAGTGTAAGAAACAGATCCTCCTCTCCGTAACAAAAACATTTGACAAAACAAAACAAACAAACAAACA
AACTTAATAAAACAAGAACAGATTAATTCCCGAGCCCTACCCCTCAGTTCTATTACTCCCATTCCCTCTCACCTG
ATTATTAGCTTCTTACTTTGTGGAATGCAATTAGGAAGGGTGATACGAATTTAAAGGTTTGGTGTTGCCATAGTA
ACCATTATTATATTTTTTAGCAAAACAGTTATCTGAAATGACTCGGACATGCATGTATGGTTGAATAAATGAATT
TAAATGCAATTTTATAACAGAAACTACTACTTTATGGCATAACCTTAGTCACCAGTCCTACTAGTGTCTTGGTAT
ATTGGTTCGGGCCTTCGTCTTTCTATGGAAAGCTCGTTGGTTTGATTCCCAACCGCGGCACGTGTGTCCTTCAGC
AAGACACTTTATACACACTTACTGCTCTCAACCCAGGTGTAGCAAACGGGGTACATTTATTCATTGTATGCTAGA
CTCGGATGCGCCTGCCAGGCGCCTCAGAATAGAATTTCTAGATATAAATGCTGGTCTATTATTAT
>gi|638027504|gb|GAVF01081820.1|
AATCCGAAGACGGTAACTCACGATGTCGACACCGATATCGTGACGCGAGAACCATGTCGTACAAGAAGAATTTTG
GTGGGCCTTACAAGACTGATCCAGCTACTGACCTCACCCGATGGCGTCTGACCAATGTCAATGGCCGGCAGACAT
GGCGGTACATACCGGAGGGAGAGGAGCCCGACCGGCCACAGAACTTTGTGGAGAAATTTGCTCTTGGATTGAGCA
TAGATGAGTTGACCCCGCCTCTCTCAAAGGCAAAGACGGCGAAGGAAGCAGCCAAGAATGGGATGCGATTCTACT
CCAAACTTCAGACCGAGGATGGCCATTGGTCTGGTGATTACGGAGGACCTCTCTTCCTCCTTCCAGGCCTGATCA
TTGTGTGTTACATCACAGGAGTGGTTCTCCCCGAGGCTTCCAAGAAGGAGATGATTCGTTATCTGAGGTCCGTGC
AGTGTCCGGATGGCGGCTGGGGCCTTCACATTGAAGACCACGCCACAGTGTTTGGGACAGCTATGAACTACGTGA
CTATGAGACTTCTTGGTGTATCGAAAGATGATAAGGATCTAAAAAAGGCCAGAAAACTGCTCATGGAAATGGGTG
GAGCAGAGAGTATTCCTTCTTGGGGCAAGTTCTGGCTGTGTGTGCTCAACCTATACAAATGGGAGGGCATGCACT
GTCTGTTTCCAGAAATATGGCTGTTCCCATCTTGGATGCCAGCCCACCCCTCATCCATCTGGTGCCACTGCAGAC
AGGTCTATCTTCCGATGGGCTACTTCTATGGGGCTAAGTTCCAGGCCCAAGAGGATGGCCTCATCAGAGAACTCA
GGAACGAGATATTCAAAGAGGATTTTGCAACCATCAACTGGCCAGCGCAGAGAGATAACATCTCCAAGTATGATC
TGTACACTCCGCACAGCTGGCTTTACACCATTGCCATGGCCATTTTGGATTTCTATGAGAAGTTTCACTCCACCT
GGTTGAGACAAAAGGCTCTGGATTTCTGCTATGATCACATCAAAGCGGATGACACTTTCACAAAGGGCATCAGTA
TAGGACCGATCTCGAAGGTGATTCAGATGCTGGTCAGATGGCATGTAGATGGGCCAGAGTCAGATGCCTTCAAGA
TGCACAGGGATCGTATCCCAGACTATCTATGGATTGGATTGGATGGGATGAAAATGCAGGGAACAAATGGCTCAC
AACTCTGGGACACCGCTTTTGCAGTCCAAGCATTCATTGAGGCTGGGGCAAACACAGAAGAGGAATTCCGCGACA
CTCTACGCAACGCTCACGGCTTCCTACGCAACACGCAAATCCCAGACAACCCTCCAGACTACCAGAAGTACTACC
GACAAATGAACAAGGGAGGGTATCCTTTCTCTACCAAAGACTGTGGCTGGATTGTAGCCGATTGCACGGCAGAAG
GTCTCAAATCCGCCATGCTACTGGAGGAGAAGTGTCCTTTTGTTACTGACCTCATTGGCAAGGAGAGGCACTGTT
TGGCTGTCGATGTCTTGATAGACATGAGGAATCCCGACGGTGGTTATGCAACGTACGAAACTACCAGAGGAGGTT
ACATACTGGAAAAGCTCAACCCCTCCGAGGTCTTCGGTGATATCATGATCGACTACACCTATGTTGAGTGTACAG
CAGCTGCCATGCTTGCATTGAAGCACTTCCAGGACCAGCACCCAGAGTACAGACGAGATGAAATCAACTCTGTGC
TGGATACTGGATTGGATTTCATCAAGGGAATTCAACGTCCTGATGGATCATGGGAAGGGTCGTGGGGTGTTTGCT
TCACCTACGGAGCTTGGTTTGGTCTGGAGGGACTCGGATGCATGGGAATGCGGTACGATCGCGACACTGCTACGC
CAGAAGTGAAGAAAGCGTGTGCGTTCCTGGTCTCTAAGCAGATGGCGGACGGTGGTTGGGGGGAGAAGTTTGAAT
CCTGCGAGACGAGGACGTACGTGCAGAGCGAGACATCCCAAGGCGTCCAAACCTGTTGGGCATTGATGGCGCTTA
TGGCAGTCAGGTATCCCGATCGCACAGTGATTGACCGTGGTATCAAGCTGATCATGGACACACAACTAGAGAACG
GGGACTGGCCTCAGGCGAACATCATGGGGGTGTTCAACAAGTCTTGCGCCATCAGCTACACCAGCTACCGCAATG
TCTTCCCCATCTGGTGTCTGGGGCGCTACGCCAAACTCTACCCCACCCCGAGCCCCGACTCCACCCCTTCTGCAA
GCACAGCCAGCACACCACTGTCAGATAGGTCGGACAGCAACGAAGAAGGCTGGGAGAAATTGTAACGGGTTTGAC
ATCACCTTGATAACCAATCCTACGAGGAGTGTAAGAAACAGATCCTCCTCTCCGTAACAAAAACATTTGACAAAA

CAAAACAAACAAACAAACAAACTTAATAAAACAAGAACAGATTAATTCCCGAGCCCTACCCCTCAGTTCTATTAC
TCCCATTCCCTCTCACCTGATTATTAGCTTCTTACTTTGTGGAATGCAATTAGGAAGGGTGATACGAATTTAAAG
GTTTGGTGTTGCCATAGTAACCATTATTATATTTTTTAGCAAAACAGTTATCTGAAATGACTCGGACATGCATGT
ATGGTTGAATAAATGAATTTAAATGCAATTTTATAACAGAAACTACTACTTTATGGCATAACCTTAGTCACCAGT
CCTACTAGTGTCTTGGTATATTGGTTCGGGCCTTCGTCTTTCTATGGAAAGCTCGTTGGTTTGATTCCCAACCGC
GGCACGTGTGTCCTTCAGCAAGACACTTTATACACACTTACTGCTCTCAACCCAGGTGTAGCAAACGGGGTACAT
TTATTCATTGTATGCTAGACTCGGATGCGCCTGCCAGGCGCCTCAGAATAGAATTTCTAGATATAAATGCTGGTC
TATTATTAT
>gi|638027505|gb|GAVF01081819.1|
CTGGCCCCATATTCATAAAACCCCACTAGCTTAGTTCCATGCTTAGGCTATTTTGGAAAGCAACGAGTAGTCTTC
TCACTAGGGTTAGTTGGAGTCACATTTGTTACTACCCAGGAACTCTAGTTGGTAACTAAAATGACCACTAGTTGC
ATACTTTGTCCTATCATTCAAAACCGACTTAGACATGAAACTAAGCTAGCTGGGTATCATGAGTATCGGCCCTTG
CATGATCTATTTTTAAAAGTTCCAATCAACACTACTACATTTGCTCTTAAGTGTTGTTTGGAACATACTTCAAAT
GTTAACTTTGACACAAAGAGAATAATATAGTATACAATTGTATACATGAGACTATTTTATTGATATTTTTTTGTA
TTTTGTTTATTTTTACCTAAATAGAGAGTAATGCTGTTGTTCATGTCAGATTTAGAGCAGTTTGGAATTATGTTT
ATCGGTGTTCCTTCATTCAGTGCACATTGTGAACATTTTATTAGTGGTAACATTTTATATGGTTTCAATTGTCTT
TTTAAAAGTGAGTTTAAAATTTACGAAAATGTATTTCCAAAATGAGATAATTTACGTTTTTAGAGAAAAAAAGAG
AGAAGTAAATGTGTACATGATTTATTCAATAGTTTTTGATGAATTTGGAAGACAGATATGTACATAAATTTAAAT
GTACATGCATACTCCTGACTAGGTGTCATTTATGAATGGGGAGGGCGTGATAGGCAGTTCAGATTTTGATGCATA
TATTTGGGTTTGGTGTTCAATTTTTGTTAAATGAAAGTAAACTGCTGAAAACAAAACGCAACATACAGTTACGCA
CAAAGTGATTAAAAGAGATGCGATGCATTTAGTAGTGATATTACAATTTCCATGTCCTTGTGGGATAAGGATATT
GTAATTGATTCAATTTTGATTGAAAATATTATGAATTATGCAATCTGACTCGCGTTGAGTGTATTCATGTATTAC
CTCATATTGAGTGTCGGGTACTTTTTCATCCCAAAGATTTCAAACAAAGATAATGTGGTAATAATAATACTACTA
ATAATAATAATTATACATTTATATCGCGCGCACATACTAAGATAATAACACAATGAAATATATACATACTACATT
ACAACAAAGATATCAATACATATATCATAAATATCAGTCATAATAATACTCATGGACATGAACTTTTGTAGGTAC
ATTTATATTTGAATATGAATAAGAAAAAAAATAATGGAAAAAACGAATAATAAGAAGACTATTTGTAATCGATCG
ATCACATGTTATGATCGTTCATGATCGATCACAAACCGGCTATCGCGCGTTACGTCCGACCCCTTCTCGTCGCGT
CGGATGTCGACACCGATATCGTGACGCGAGAACCATGTCGTACAAGAAGAATTTTGGTGGGCCTTACAAGACTGA
TCCAGCTACTGACCTCACCCGATGGCGTCTGACCAATGTCAATGGCCGGCAGACATGGCGGTACATACCGGAGGG
AGAGGAGCCCGACCGGCCACAGAACTTTGTGGAGAAATTTGCTCTTGGATTGAGCATAGATGAGTTGACCCCGCC
TCTCTCAAAGGCAAAGACGGCGAAGGAAGCAGCCAAGAATGGGATGCGATTCTACTCCAAACTTCAGACCGAGGA
TGGCCATTGGTCTGGTGATTACGGAGGACCTCTCTTCCTCCTTCCAGGCCTGATCATTGTGTGTTACATCACAGG
AGTGGTTCTCCCCGAGGCTTCCAAGAAGGAGATGATTCGTTATCTGAGGTCCGTGCAGTGTCCGGATGGCGGCTG
GGGCCTTCACATTGAAGACCACGCCACAGTGTTTGGGACAGCTATGAACTACGTGACTATGAGACTTCTTGGTGT
ATCGAAAGATGATAAGGATCTAAAAAAGGCCAGAAAACTGCTCATGGAAATGGGTGGAGCAGAGAGTATTCCTTC
TTGGGGCAAGTTCTGGCTGTGTGTGCTCAACCTATACAAATGGGAGGGCATGCACTGTCTGTTTCCAGAAATATG
GCTGTTCCCATCTTGGATGCCAGCCCACCCCTCATCCATCTGGTGCCACTGCAGACAGGTCTATCTTCCGATGGG
CTACTTCTATGGGGCTAAGTTCCAGGCCCAAGAGGATGGCCTCATCAGAGAACTCAGGAACGAGATATTCAAAGA
GGATTTTGCAACCATCAACTGGCCAGCGCAGAGAGATAACATCTCCAAGTATGATCTGTACACTCCGCACAGCTG
GCTTTACACCATTGCCATGGCCATTTTGGATTTCTATGAGAAGTTTCACTCCACCTGGTTGAGACAAAAGGCTCT
GGATTTCTGCTATGATCACATCAAAGCGGATGACACTTTCACAAAGGGCATCAGTATAGGACCGATCTCGAAGGT
GATTCAGATGCTGGTCAGATGGCATGTAGATGGGCCAGAGTCAGATGCCTTCAAGATGCACAGGGATCGTATCCC
AGACTATCTATGGATTGGATTGGATGGGATGAAAATGCAGGGAACAAATGGCTCACAACTCTGGGACACCGCTTT
TGCAGTCCAAGCATTCATTGAGGCTGGGGCAAACACAGAAGAGGAATTCCGCGACACTCTACGCAACGCTCACGG
CTTCCTACGCAACACGCAAATCCCAGACAACCCTCCAGACTACCAGAAGTACTACCGACAAATGAACAAGGGAGG
GTATCCTTTCTCTACCAAAGACTGTGGCTGGATTGTAGCCGATTGCACGGCAGAAGGTCTCAAATCCGCCATGCT
ACTGGAGGAGAAGTGTCCTTTTGTTACTGACCTCATTGGCAAGGAGAGGCACTGTTTGGCTGTCGATGTCTTGAT
AGACATGAGGAATCCCGACGGTGGTTATGCAACGTACGAAACTACCAGAGGAGGTTACATACTGGAAAAGCTCAA
CCCCTCCGAGGTCTTCGGTGATATCATGATCGACTACACCTATGTTGAGTGTACAGCAGCTGCCATGCTTGCATT
GAAGCACTTCCAGGACCAGCACCCAGAGTACAGACGAGATGAAATCAACTCTGTGCTGGATACTGGATTGGATTT
CATCAAGGGAATTCAACGTCCTGATGGATCATGGGAAGGGTCGTGGGGTGTTTGCTTCACCTACGGAGCTTGGTT
TGGTCTGGAGGGACTCGGATGCATGGGAATGCGGTACGATCGCGACACTGCTACGCCAGAAGTGAAGAAAGCGTG
TGCGTTCCTGGTCTCTAAGCAGATGGCGGACGGTGGTTGGGGGGAGAAGTTTGAATCCTGCGAGACGAGGACGTA
CGTGCAGAGCGAGACATCCCAAGGCGTCCAAACCTGTTGGGCATTGATGGCGCTTATGGCAGTCAGGTATCCCGA
TCGCACAGTGATTGACCGTGGTATCAAGCTGATCATGGACACACAACTAGAGAACGGGGACTGGCCTCAGGCGAA
CATCATGGGGGTGTTCAACAAGTCTTGCGCCATCAGCTACACCAGCTACCGCAATGTCTTCCCCATCTGGTGTCT
GGGGCGCTACGCCAAACTCTACCCCACCCCGAGCCCCGACTCCACCCCTTCTGCAAGCACAGCCAGCACACCACT
GTCAGATAGGTCGGACAGCAACGAAGAAGGCTGGGAGAAATTGTAACGGGTTTGACATCACCTTGATAACCAATC
CTACGAGGAGTGTAAGAAACAGATCCTCCTCTCCGTAACAAAAACATTTGACAAAACAAAACAAACAAACAAACA
AACTTAATAAAACAAGAACAGATTAATTCCCGACCCCTACCCCTCAACTAGCACTACCATTACGAGACACACAGA
TAAAGTTAGATTGGGCAAGGATCAGGTAGGGTGAAGACAATTAGATGTAGACTTGAATTATATCTTTGGATAACA

GTCAACAACTAATGGCATTACCCTAGTGACGCTGTTCCACCTAATTATTAGCTTCTTACTTTGTGGAATGCAATT
AGGAAGGGTGATACGAATTTAAAGGTTTGGTGTTGCCATAGTAACCATTATTATATTTTTTAGCAAAACAGTTAT
CTGAAATGACTCGGACATGCATGTATGGTTGAATAAATGAATTTAAATGCAATTTTATAACAGAAACTACTACTT
TATGGCATAACCTTAGTCACCAGTCCTACTAGTGTCTTGGTATATTGGTTCGGGCCTTCGTCTTTCTATGGAAAG
CTCGTTGGTTTGATTCCCAACCGCGGCACGTGTGTCCTTCAGCAAGGCACTTTATCCACATTTGCTGCTCTCAAC
CCAGGTGTAGTAAATGGGTACCTGGCAGGGAGGGATTTATTCCTTGAAATGCACAAACATGTCGGGACATTGAAC
GGGACCCCATCTTAAGAAGTCGGGCACTCGTCTGATCATGCCTCAAAAGCAGTAACTATTTCACCGACATAAAGA
GCTTTGTGTGTCATGCTTTGTGTGTCATGGTACTACAGGTA
>gi|638027506|gb|GAVF01081818.1|
AATCCGAAGACGGTAACTCACGATGTCGACACCGATATCGTGACGCGAGAACCATGTCGTACAAGAAGAATTTTG
GTGGGCCTTACAAGACTGATCCAGCTACTGACCTCACCCGATGGCGTCTGACCAATGTCAATGGCCGGCAGACAT
GGCGGTACATACCGGAGGGAGAGGAGCCCGACCGGCCACAGAACTTTGTGGAGAAATTTGCTCTTGGATTGAGCA
TAGATGAGTTGACCCCGCCTCTCTCAAAGGCAAAGACGGCGAAGGAAGCAGCCAAGAATGGGATGCGATTCTACT
CCAAACTTCAGACCGAGGATGGCCATTGGTCTGGTGATTACGGAGGACCTCTCTTCCTCCTTCCAGGCCTGATCA
TTGTGTGTTACATCACAGGAGTGGTTCTCCCCGAGGCTTCCAAGAAGGAGATGATTCGTTATCTGAGGTCCGTGC
AGTGTCCGGATGGCGGCTGGGGCCTTCACATTGAAGACCACGCCACAGTGTTTGGGACAGCTATGAACTACGTGA
CTATGAGACTTCTTGGTGTATCGAAAGATGATAAGGATCTAAAAAAGGCCAGAAAACTGCTCATGGAAATGGGTG
GAGCAGAGAGTATTCCTTCTTGGGGCAAGTTCTGGCTGTGTGTGCTCAACCTATACAAATGGGAGGGCATGCACT
GTCTGTTTCCAGAAATATGGCTGTTCCCATCTTGGATGCCAGCCCACCCCTCATCCATCTGGTGCCACTGCAGAC
AGGTCTATCTTCCGATGGGCTACTTCTATGGGGCTAAGTTCCAGGCCCAAGAGGATGGCCTCATCAGAGAACTCA
GGAACGAGATATTCAAAGAGGATTTTGCAACCATCAACTGGCCAGCGCAGAGAGATAACATCTCCAAGTATGATC
TGTACACTCCGCACAGCTGGCTTTACACCATTGCCATGGCCATTTTGGATTTCTATGAGAAGTTTCACTCCACCT
GGTTGAGACAAAAGGCTCTGGATTTCTGCTATGATCACATCAAAGCGGATGACACTTTCACAAAGGGCATCAGTA
TAGGACCGATCTCGAAGGTGATTCAGATGCTGGTCAGATGGCATGTAGATGGGCCAGAGTCAGATGCCTTCAAGA
TGCACAGGGATCGTATCCCAGACTATCTATGGATTGGATTGGATGGGATGAAAATGCAGGGAACAAATGGCTCAC
AACTCTGGGACACCGCTTTTGCAGTCCAAGCATTCATTGAGGCTGGGGCAAACACAGAAGAGGAATTCCGCGACA
CTCTACGCAACGCTCACGGCTTCCTACGCAACACGCAAATCCCAGACAACCCTCCAGACTACCAGAAGTACTACC
GACAAATGAACAAGGGAGGGTATCCTTTCTCTACCAAAGACTGTGGCTGGATTGTAGCCGATTGCACGGCAGAAG
GTCTCAAATCCGCCATGCTACTGGAGGAGAAGTGTCCTTTTGTTACTGACCTCATTGGCAAGGAGAGGCACTGTT
TGGCTGTCGATGTCTTGATAGACATGAGGAATCCCGACGGTGGTTATGCAACGTACGAAACTACCAGAGGAGGTT
ACATACTGGAAAAGCTCAACCCCTCCGAGGTCTTCGGTGATATCATGATCGACTACACCTATGTTGAGTGTACAG
CAGCTGCCATGCTTGCATTGAAGCACTTCCAGGACCAGCACCCAGAGTACAGACGAGATGAAATCAACTCTGTGC
TGGATACTGGATTGGATTTCATCAAGGGAATTCAACGTCCTGATGGATCATGGGAAGGGTCGTGGGGTGTTTGCT
TCACCTACGGAGCTTGGTTTGGTCTGGAGGGACTCGGATGCATGGGAATGCGGTACGATCGCGACACTGCTACGC
CAGAAGTGAAGAAAGCGTGTGCGTTCCTGGTCTCTAAGCAGATGGCGGACGGTGGTTGGGGGGAGAAGTTTGAAT
CCTGCGAGACGAGGACGTACGTGCAGAGCGAGACATCCCAAGGCGTCCAAACCTGTTGGGCATTGATGGCGCTTA
TGGCAGTCAGGTATCCCGATCGCACAGTGATTGACCGTGGTATCAAGCTGATCATGGACACACAACTAGAGAACG
GGGACTGGCCTCAGGCGAACATCATGGGGGTGTTCAACAAGTCTTGCGCCATCAGCTACACCAGCTACCGCAATG
TCTTCCCCATCTGGTGTCTGGGGCGCTACGCCAAACTCTACCCCACCCCGAGCCCCGACTCCACCCCTTCTGCAA
GCACAGCCAGCACACCACTGTCAGATAGGTCGGACAGCAACGAAGAAGGCTGGGAGAAATTGTAACGGGTTTGAC
ATCACCTTGATAACCAATCCTACGAGGAGTGTAAGAAACAGATCCTCCTCTCCGTAACAAAAACATTTGACAAAA
CAAAACAAACAAACAAACAAACTTAATAAAACAAGAACAGATTAATTCCCGACCCCTACCCCTCAACTAGCACTA
CCATTACGAGACACACAGATAAAGTTAGATTGGGCAAGGATCAGGTAGGGTGAAGACAATTAGATGTAGACTTGA
ATTATATCTTTGGATAACAGTCAACAACTAATGGCATTACCCTAGTGACGCTGTTCCACCTAATTATTAGCTTCT
TACTTTGTGGAATGCAATTAGGAAGGGTGATACGAATTTAAAGGTTTGGTGTTGCCATAGTAACCATTATTATAT
TTTTTAGCAAAACAGTTATCTGAAATGACTCGGACATGCATGTATGGTTGAATAAATGAATTTAAATGCAATTTT
ATAACAGAAACTACTACTTTATGGCATAACCTTAGTCACCAGTCCTACTAGTGTCTTGGTATATTGGTTCGGGCC
TTCGTCTTTCTATGGAAAGCTCGTTGGTTTGATTCCCAACCGCGGCACGTGTGTCCTTCAGCAAGGCACTTTATC
CACATTTGCTGCTCTCAACCCAGGTGTAGTAAATGGGTACCTGGCAGGGAGGGATTTATTCCTTGAAATGCACAA
ACATGTCGGGACATTGAACGGGACCCCATCTTAAGAAGTCGGGCACTCGTCTGATCATGCCTCAAAAGCAGTAAC
TATTTCACCGACATAAAGAGCTTTGTGTGTCATGCTTTGTGTGTCATGGTACTACAGGTA
>gi|639028563|gb|GAVR01073116.1|
AACTCTTTGTAAGACGGGGCCCAGCGCCCAGAACTCAGATTCAGAAGCTAAGTAAAGTTCTAGATAAGAAAACCT
GCGCCGGCGCAACTGGCTGCTGCTGCTCGCTCATCCATGTCATCATGCTCGTCACAGAGAGTTAGGAATCACAAC
TTTAAACAAGAATCATGTCTGATAAAAAGAATCGTGGAGGACCTTACAGAACCACACCGGCCACTGATCTCTCTC
GATGGCGCCTCACCAATGTGAATGGACGACAGACCTGGAGATACTACCCAGAAGGGGACGAGCCTGGACGTCCAC
AAAATTTTGTCGAAAAGTTCTCCCTTGGACTTGATATAAATGGTGAAGCCCCACCATTGCCCAAAGCTAGGAATG
CCAAAGAAGCTGCAAAAAATGGCATGGAGTTTTACTCCAAGCTACAAACTGAAGATGGTCATTGGTCAGGAGACT
ATGGTGGACCTCTCTTTCTTCTTCCAGGTCTGGTCATAGTGTGTTTCATAACCGGGATAGCGCTCCCAGATGCTT
CCAAGAAGGAGATGGTGCGATACCTTCGATCAGTGCAATGTCCTGATGGCGGGTGGGGATTGCACATTGAGGACC
ATCCTACAGTGTTTGGTACAGCAATGAACTATGTGACAATGAGACTACTGGGTGTTTCTAAGGATGATACAGATC

TCAAGAAAGCAAGGAAACTGCTTATGGAGATGGGTGGTGCAGAGAGTATTCCATCTTGGGGTAAATTCTGGCTCT
GTGTTCTCAATCTTTACAAATGGGAAGGAATGCACTGTTTGTTTCCTGAAATATGGTTGTTTCCTGAGTGGTTTC
CTGCTCACCCATCTTCTATCTGGTGTCATTGTCGTCAGGTTTACCTTCCTATGGGATTCTTCTATGGCATTAAGT
TTCAGGCAGAAGAAAATGACCTTATCAGAGAACTCAGGAAGGAAATCTTCAAGGAAGACTTCTCCAGCATACATT
GGCCATCCCAGAGAAATAACATTTCCAAATTTGATCTTTACACACCTCATAGTTGGATGTACAATATTGCCATGG
CCATCTTGGATTTTTATGAGACTTTCCACTCCACCTGGTTAAGAAAGAAGGCATTGGATCACTGCTATGATCACA
TCAAAGCTGATGACGAATTCACAAAAGGAATCAGCATTGGTCCAATCTCTAAAGTGATTCAGATGTTGGTGAGAT
GGCATATTGACGGGCCTGATTCTGAAGCTTTTAAGATGCACAAGGATCGCATCCCAGACTACTTATGGATTGGCT
TGGATGGCATGAAAATGCAGGGAACCAATGGCTCTCAGCTATGGGATACAGCCTTTGTCATACAAGCTTTCTTAG
AGGCCGGAGCCGTTCCAGATTTCAAGGACACGTTAATCAACGCTCATGACTTCTTGAAGAACACGCAGATCCCTG
ACAACCCTCCAAATTATCAGAAATACTATCGCCAGATGAACAAGGGAGGCTACCCATTTTCAACCAAGGACTGTG
GGTGGATTGTTTCTGACTGTACGGCAGAAGGTCTTAAGTCCGCTATGATGCTGGAGGAGATGTGTCCTTATATCT
>gi|639028568|gb|GAVR01073111.1|
CTCTCTCGGGAGCTGAAAAAAATGAATGGAGAAATCGTTCGATGCGATGTGACTTAGTGAGCAAGCCAGCGCCAA
AGTACCCATCCAAATTCCATACTTAGATCTATACTGATTAATCATGTCTGATAAAAAGAATCGTGGAGGACCTTA
CAGAACCACACCGGCCACTGATCTCTCTCGATGGCGCCTCACCAATGTGAATGGACGACAGACCTGGAGATACTA
CCCAGAAGGGGACGAGCCTGGACGTCCACAAAATTTTGTCGAAAAGTTCTCCCTTGGACTTGATATAAATGGTGA
AGCCCCACCATTGCCCAAAGCTAGGAATGCCAAAGAAGCTGCAAAAAATGGCATGGAGTTTTACTCCAAGCTACA
AACTGAAGATGGTCATTGGTCAGGAGACTATGGTGGACCTCTCTTTCTTCTTCCAGGTCTGGTCATAGTGTGTTT
CATAACCGGGATAGCGCTCCCAGATGCTTCCAAGAAGGAGATGGTGCGATACCTTCGATCAGTGCAATGTCCTGA
TGGCGGGTGGGGATTGCACATTGAGGACCATCCTACAGTGTTTGGTACAGCAATGAACTATGTGACAATGAGACT
ACTGGGTGTTTCTAAGGATGATACAGATCTCAAGAAAGCAAGGAAACTGCTTATGGAGATGGGTGGTGCAGAGAG
TATTCCATCTTGGGGTAAATTCTGGCTCTGTGTTCTCAATCTTTACAAATGGGAAGGAATGCACTGTTTGTTTCC
TGAAATATGGTTGTTTCCTGAGTGGTTTCCTGCTCACCCATCTTCTATCTGGTGTCATTGTCGTCAGGTTTACCT
TCCTATGGGATTCTTCTATGGCATTAAGTTTCAGGCAGAAGAAAATGACCTTATCAGAGAACTCAGGAAGGAAAT
CTTCAAGGAAGACTTCTCCAGCATACATTGGCCATCCCAGAGAAATAACATTTCCAAATTTGATCTTTACACACC
TCATAGTTGGATGTACAATATTGCCATGGCCATCTTGGATTTTTATGAGACTTTCCACTCCACCTGGTTAAGAAA
GAAGGCATTGGATCACTGCTATGATCACATCAAAGCTGATGACGAATTCACAAAAGGAATCAGCATTGGTCCAAT
CTCTAAAGTGATTCAGATGTTGGTGAGATGGCATATTGACGGGCCTGATTCTGAAGCTTTTAAGATGCACAAGGA
TCGCATCCCAGACTACTTATGGATTGGCTTGGATGGCATGAAAATGCAGGGAACCAATGGCTCTCAGCTATGGGA
TACAGCCTTTGTCATACAAGCTTTCTTAGAGGCCGGAGCCGTTCCAGATTTCAAGGACACGTTAATCAACGCTCA
TGACTTCTTGAAGAACACGCAGATCCCTGACAACCCTCCAAATTATCAGAAATACTATCGCCAGATGAACAAGGG
AGGCTACCCATTTTCAACCAAGGACTGTGGGTGGATTGTTTCTGACTGTACGGCAGAAGGTCTTAAGTCCGCTAT
GATGCTGGAGGAGATGTGTCCTTATATCT
>gi|639028561|gb|GAVR01073118.1|
CTCTCTCGGGAGCTGAAAAAAATGAATGGAGAAATCGTTCGATGCGATGTGACTTAGTGAGCAAGCCAGCGCCAA
AGTACCCATCCAAATTCCATACTTAGATCTATACTGATTAATCATGTCTGATAAAAAGAATCGTGGAGGACCTTA
CAGAACCACACCGGCCACTGATCTCTCTCGATGGCGCCTCACCAATGTGAATGGACGACAGACCTGGAGATACTA
CCCAGAAGGGGACGAGCCTGGACGTCCACAAAATTTTGTCGAAAAGTTCTCCCTTGGACTTGATATAAATGGTGA
AGCCCCACCATTGCCCAAAGCTAGGAATGCCAAAGAAGCTGCAAAAAATGGCATGGAGTTTTACTCCAAGCTACA
AACTGAAGATGGTCATTGGTCAGGAGACTATGGTGGACCTCTCTTTCTTCTTCCAGGTCTGGTCATAGTGTGTTT
CATAACCGGGATAGCGCTCCCAGATGCTTCCAAGAAGGAGATGGTGCGATACCTTCGATCAGTGCAATGTCCTGA
TGGCGGGTGGGGATTGCACATTGAGGACCATCCTACAGTGTTTGGTACAGCAATGAACTATGTGACAATGAGACT
ACTGGGTGTTTCTAAGGATGATACAGATCTCAAGAAAGCAAGGAAACTGCTTATGGAGATGGGTGGTGCAGAGAG
TATTCCATCTTGGGGTAAATTCTGGCTCTGTGTTCTCAATCTTTACAAATGGGAAGGAATGCACTGTTTGTTTCC
TGAAATATGGTTGTTTCCTGAGTGGTTTCCTGCTCACCCATCTTCTATCTGGTGTCATTGTCGTCAGGTTTACCT
TCCTATGGGATTCTTCTATGGCATTAAGTTTCAGGCAGAAGAAAATGACCTTATCAGAGAACTCAGGAAGGAAAT
CTTCAAGGAAGACTTCTCCAGCATACATTGGCCATCCCAGAGAAATAACATTTCCAAATTTGATCTTTACACACC
TCATAGTTGGATGTACAATATTGCCATGGCCATCTTGGATTTTTATGAGACTTTCCACTCCACCTGGTTAAGAAA
GAAGGCATTGGATCACTGCTATGATCACATCAAAGCTGATGACGAATTCACAAAAGGAATCAGCATTGGTCCAAT
CTCTAAAGTGATTCAGATGTTGGTGAGATGGCATATTGACGGGCCTGATTCTGAAGCTTTTAAGATGCACAAGGA
TCGCATCCCAGACTACTTATGGATTGGCTTGGATGGCATGAAAATGCAGGGAACCAATGGCTCTCAGCTATGGGA
TACAGCCTTTGTCATACAAGCTTTCTTAGAGGCCGGAGCCGTTCCAGATTTCAAGGACACGTTAATCAACGCTCA
TGACTTCTTGAAGAACACGCAGATCCCTGACAACCCTCCAAATTATCAGAAATACTATCGCCAGATGAACAAGGG
AGGCTACCCATTTTCAACCAAGGACTGTGGATGGATTGTTTCC
>Locus_10303_Transcript_8/65
ATGCAGTCCGATAGCGACCAGAAACCTCGCTCTGAACGCCCAAATAAGGAGTACTCCGATCTTACAAGATGGCGG
CTGTCCTGTACAGAGGGAAAGAGAATCTGGCATTACGTAACAGAGGATGAGACTCCAGAAAGACCACAAAATATG
GTAGAGAAGTACTCGCTTGGACTGGATTACTCTAATGAAGCTGAGAAACTCCCTCGAGCTCAAAACCCGAAGGAA
GCTGCAGAAAATGGAATCAAATTTTTCTCTTTAATGCAAGCGGAAGATGGTCACTGGCCCAACGATTATTCAGGA
CCGTTGTTTCTAATGCCAGGTCTAATCATTGTACATTACGTTACCAAGACTAAATTCCCTGAAGCATTCAAACAG

GAGTTTATCAGGTATCTGAGGAGAGTTCAGGCTAAAGATGGAGGATGGAGTTTACATATTGAGGGCGATGCCACC
GTCTTTGGTACAGCGTTAAACTACATCTCCATGCGACTCCTCGGTGTGAGCCCAGAAGATGGAGACTTGAAAAGA
GCAAGGAAGGTCCTCCATCACCATGGAGGAGCAGCTGCCATCCCATCCTGGGGGAAGTTTTGGTTGTGCATACTC
AATTGTTATAAATGGGAAGGCATGCACACGATGTTTCCGGAACTATGGTTAATGCCCTCTTGGATTCCAGCGCAC
CCGTCCACTCTCTGGATTCATTGTCGAATGGTTTACATCGGTATGGCATTCCTCTATGGTAAGAGGTACTACGCG
CAAGAAGATGAACTGATAATGGAACTTAGGAAGGAATTGTTTATTGAAGATTTCGACCAAATTGACTGGTCATCC
CAGAGAGACAATATCGCTGAGATAGATTTATATACTCCGCATAGCTGGTTATTTAACATTACCTTTGGTATTTTA
GATAAGTATGAGCCGTTTAGGTTGACCCGGTTCAGGGAACAAGCACTCGATGTCTGCCTTGATCATATTAAACAG
GATGATCTCATGACAAGCTTTATTAGCATTGGTCCAATTTCAAAGATGATTAACATGCTCATTAGATGGCTGGAG
GATGGACCCGAGTCTGAGGCCTTCAAGAAACACGTGGAGAGAGTGTATGATTATGTTTGGATGGGACTTGATGGC
ACAAATGTGCAGGGTACTAACGGTAATCAAGTGTGGGACACTTCCTTTGCTGCTATGGCTATGCTCGATGTGGGC
GCGCAGGACGACCCACAATTCCACGAGGTTTTATCTAAAACCTATTCATATCTTGAAATCTCGCAAGTGATAGAA
AGCTCACCAGATTGTGTCAAATACTATCGGCAATACAACAAGGGAGGATGGGCTCAGACAATGAGAGAGCACGGA
TTAGTTGTGTCCGACACATCGGCAGAGGCTCTGAAAGCGGTCCTCCTGATGAACGATAAATGTCCCTTCATCACC
GAGAGGGTCTCCAAGAGACGTCTGAGAGATGCAGTGGATATGTTATTGACTATCGTAAATCCAAACGGGGGATTC
TCGTCGTATGAAAATCTTCGCGGAGGAACAATCCTGGAGTTATTAAATCCCTCCGAAGTATTTGGTGACATCATG
GTGGATTACACCTACACTGAGTGTACATCTTCAGTCTTACAAGCTCTGAGACATTTTGTCGATTCTGATCCTGAC
TACCGCCAAGATGAGATCTGGGCTGTCCTAAGAAACGCAATGGAATACATAAGATCCAATCAGTTACCGGACGGT
TCATTCGAGGGATCATGGGGTGTATGTTTCACATATGGTACATGGTTCGCTCTGGAGGCGTTTGCCTGCATGGGG
AAGAACTATCAAGACAACACTGCTAGCATCGACGTCAAGAAAGCCTGCTCGTTTCTAGTCTCCAGGCAGATGGAG
GATGGAGGCTGGGGTGAGAAGTTTGCCTCCTGCAGCGAGAGGAGATACGTGCAGAGTGAGAAATCACTGGTCGTC
AATACGTCTTGGGCTCTTCTGGGACTAATGGCAGTGAGGTATCCTGACGAAGAGGTTTTGTCGAGAGGTGTCAAA
GTCTTACTCGATAGACAGATCGATGATGGCGACTGGCCTCAGGAGAGCATATGCGGAGTTTTTAATAAATCTTGT
GCAATTGGTTACACTGCATTCAAGAATATCTTTCCAAGTTGGGCTTTAAAGGGGGGGTGTGGTCAGCTGTCAACC
TGA
>Locus_10303_Transcript_47/65
ATGCAGTCCGATAGCGACCAGAAACCTCGCTCTGAACGCCCAAATAAGGAGTACTCCGATCTTACAAGATGGCGG
CTGTCCTGTACAGAGGGAAAGAGAATCTGGCATTACGTAACAGAGGATGAGACTCCAGAAAGACCACAAAATATG
GTAGAGAAGTACTCGCTTGGACTGGATTACTCTAATGAAGCTGAGAAACTCCCTCGAGCTCAAAACCCGAAGGAA
GCTGCAGAAAATGGAATCAAATTTTTCTCTTTAATGCAAGCGGAAGATGGTCACTGGCCCAACGATTATTCAGGA
CCGTTGTTTCTAATGCCAGGTCTAATCATTGTACATTACGTTACCAAGACTAAATTCCCTGAAGCATTCAAACAG
GAGTTTATCAGGTATCTGAGGAGAGTTCAGGCTAAAGATGGAGGATGGAGTTTACATATTGAGGGCGATGCCACC
GTCTTTGGTACAGCGTTAAACTACATCTCCATGCGACTCCTCGGTGTGAGCCCAGAAGATGGAGACTTGAAAAGA
GCAAGGAAGGTCCTCCATCACCATGGAGGAGCAGCTGCCATCCCATCCTGGGGGAAGTTTTGGTTGTGCATACTC
AATTGTTATAAATGGGAAGGCATGCACACGATGTTTCCGGAACTATGGTTAATGCCCTCTTGGATTCCAGCGCAC
CCGTCCACTCTCTGGATTCATTGTCGAATGGTTTACATCGGTATGGCATTCCTCTATGGTAAGAGGTACTACGCG
CAAGAAGATGAACTGATAATGGAACTTAGGAAGGAATTGTTTATTGAAGATTTCGACCAAATTGACTGGTCATCC
CAGAGAGACAATATCGCTGAGATAGATTTATATACTCCGCATAGCTGGTTATTTAACATTACCTTTGGTATTTTA
GATAAGTATGAGCCGTTTAGGTTGACCCGGTTCAGGGAACAAGCACTCGATGTCTGCCTTGATCATATTAAACAG
GATGATCTCATGACAAGCTTTATTAGCATTGGTCCAATTTCAAAGATGATTAACATGCTCATTAGATGGCTGGAG
GATGGACCCGAGTCTGAGGCCTTCAAGAAACACGTGGAGAGAGTGTATGATTATGTTTGGATGGGACTTGATGGC
ACAAATGTGCAGGGTACTAACGGTAATCAAGTGTGGGACACTTCCTTTGCTGCTATGGCTATGCTCGATGTGGGC
GCGCAGGACGACCCACAATTCCACGAGGTTTTATCTAAAACCTATTCATATCTTGAAATCTCGCAAGTGATAGAA
AGCTCACCAGATTGTGTCAAATACTATCGGCAATACAACAAGGGAGGATGGGCTCAGACAATGAGAGAGCACGGA
TTAGTTGTGTCCGACACATCGGCAGAGGCTCTGAAAGCGGTCCTCCTGATGAACGATAAATGTCCCTTCATCACC
GAGAGGGTCTCCAAGAGACGTCTGAGAGATGCAGTGGATATGTTATTGACTATCGTAAATCCAAACGGGGGATTC
TCGTCGTATGAAAATCTTCGCGGAGGAACAATCCTGGAGTTATTAAATCCCTCCGAAGTATTTGGTGACATCATG
GTGGATTACACCTACACTGAGTGTACATCTTCAGTCTTACAAGCTCTGAGACATTTTGTCGATTCTGATCCTGAC
TACCGCCAAGATGAGATCTGGGCTGTCCTAAGAAACGCAATGGAATACATAAGATCCAATCAGTTACCGGACGGT
TCATTCGAGGGATCATGGGGTGTATGTTTCACATATGGTACATGGTTCGCTCTGGAGGCGTTTGCCTGCATGGGG
AAGAACTATCAAGACAACACTGCTAGCATCGACGTCAAGAAAGCCTGCTCGTTTCTAGTCTCCAGGCAGATGGAG
GATGGAGGCTGGGGTGAGAAGTTTGCCTCCTGCAGCGAGAGGAGATACGTGCAGAGTGAGAAATCACTGGTCGTC
AATACGTCTTGGGCTCTTCTGGGACTAATGGCAGTGAGGTATCCTGACCAATCTGTCTTAGAGAGAGCAATTCAA
GTTTTACGTGATCGCCAACATGAAGACGGCGACTGGCCCCAGGAAACCATTTCTGGAGTTTTTAACAGGTCATGT
GCGATTTCGTATCCGGCATTTAAGAACATTTTCCCGATATGGGCTCTGGGACGTTATTCTCAGCATTACACGTCT
TCCAAGGAAACTCAAGACACTTGTGTACGAAATGACGATTGGGAAAAGTTGTCAAACAACTGGCAGCTCGCTAAA
TTGGTATTTTAG
>Locus_2545_Transcript_7/30
AGCCCAACTTGGAAAGATATTCTTGAATGCAGTGTAACTAATTTCACAACCCTGATTTATTAAAAAATTTAATAA
AAAAGAATATTATTTGCATTTTAAAGCTATAAAAGTGCTTATACATGGGGGATCTTTTCCCAGTCCTTGTCGGAG
ACACTTTCTGCTGAGAACGTCCTGGACGAGGCGGGGTAAAGCTGAGCGTGCCTCCCCAAGGCCCAGATTGGAAAG

ATATTCTTGAATGCAGTGTAACCAATTGCACAAGATTTATTAAAAACTCCGCATATGCTCTCCTGAGGCCAGTCG
CCATCATCGATCTGTCTATCGAGTAAGACTTTGACACCTCTCGACAAAACCTCTTCGTCAGGATACCTCACTGCC
ATTAGTCCCAGAAGAGCCCAAGCCGTATTGACGACCAGTGATTTCTCACTCTGCACGTATCTCCTCTCGCTGCAG
GAGGCAAACTTCTCACCCCAACCTCCGTCCTCCATCTGCCTCGAGACTAGAAACGAGCAGGCTTTCTTGACTTGC
ATGGAGGCAGTGTTATCATTGTAATTCTGACCCATGCAGGCAAAAGCTTCTAGTGCAAACCAGGTTCCATACGTA
AAACAGACTCCCCAGGAACCTTCAAATGAGCCATCAGGGAGTTGATTTTGTTTGATATATCCCAGGCCATTTTTC
AGCACATTCCAGATCTCGTCTGTTCTATAGTTTGGATCGTAGTCCACGAAATGTCTCAGAGCTTGTAAGACCGAA
GATGTGCACTCAGTGTAGGTGTAATCCACCATGATGTCACCGAATACTTCAGAGGGATTAAGTAACTCCAACAAC
TTTTCTCCGCCTCGAAGGGTCTCGTAACTGGAGTACCCGCCGTTCGGGTTAACCATGTCTAGAAGCAGGTCGACT
GCTTCGTGAAGACGCTCTCTGGATATTTTCGCTTCGCTACCGATGAAGGGACATTTCTCTTCGATCAGCATTGCG
GCTTTCAATGCCTCGGCTGTCGTATCCGACACAATCAGACCGTGATCTCTCGTCGTGAGAGGCCAGCCTCCTTTG
TTATATTGGCGATAATACTGCACACATTTTGGAGAGCTCTCAACCATCTGTGAAACTTCAAGATAAGAGTAAGCT
TTCCTCAGAGCCTCTTGAAACTCCGGCTTGGTACCGGCGCCCGCCTCGAGCATAGCCATCGCAGCATAGGAGATG
TCCCATATCTGGGTTCCGTTTGTACCCTGCATATTTACGCCATCTCGGCCCATCCAGATGTAGTCATAGACCCTA
TCCACATGTTGCAGAAATGCTTTCGAATCTGGACCGTCTTCAAGCCACCGAATCAGCATATTGATCATTTTGGAG
ATTGGCCCAATACTGATGAAGTTGGTCATAATATCATCTTGTTTGATATGATCCAAGCAAACCTTGAGAGCACTT
TCTCTGTACCAGCTGCTGTGAAAGGGTTCATAGGTATCTAACATCCCGAATACCATGGAGTAAAGCCAGCTATGA
GGAGTGTAAATGTCAATTTTGGCAATGTTTCTCTCTGTGCCGGCCAATCAATCGTTTCAAAGTCCTCGACGAAT
AATTCCTTTCTCAGGCTTAAGATAAGACTATCCTCTGGAGCGTAGTATCTTTTGCCGTAGAGATACGACATCCCA
AGATAAACGATACGGCAGTGAACCCAGAGTGTTGATGGATGGGCGGGTAACCACGAGGGCAGCAACCATAGTTCG
GGGAACAGCGTGTGCATGCCTTCCCACTTATAACAGTTCAGTATGCACAACCAAAACTTCCCCCAGGATGGGATG
GCAGCTGCCCCTCCATGGTGATGGAGGACCTTCCTTGCTCTTTTCAAGTCTCCATCTTCTGGGCTCACACCGAGG
AGTCGCATGGAGATGTAGTTTAACGCTGTACCAAAGACGGTGGCATCGCCCTCAATATGTAAACTCCATCCTCCA
TCTTTAGCCTGAACTCTCCTCAGATACCTGATAAACTCCTGTTTGAATGCTTCAGGGAATTTAGTCTTGGTAACG
TAATGTACAATGATTAGACCTGGCATTAGAAACAACGGTCCTGAATAATCGTTGGGCCAGTGACCATCTTCCGCT
TGCATTAAAGAGAAAAATTTGATTCCATTTTCTGCAGCTTCCTTCGGGTTTTGAGCTCGAGGGAGTTTCTCAGCT
TCATTAGAGTAATCCAGTCCAAGCGAGTACTTCTCTACCATATTTTGTGGTCTTTCTGGAGTCTCATCCTCTGTT
ACGTAATGCCAGATTCTCTTTCCCTCTGTACAGGACAGCCGCCATCTTGTAAGATCGGAGTACTCCTTATTTGGG
CGTTCAGAGCGAGGTTTCTGGTCGCTATCGGACTGCATTGTTCATGTAAGACGTACGCCCTTTAACTTTATCGTT
GGTTACGTCAGGAAAAGACAGTGAATAATCAAAACTTCTTCTCATCCAGAATAAAAAAAACAAATTTACTGGACT
ACCGATAAAACTGATGAATGATTAACTTGGAAGCCCCGTCTGCTAGAAGAAATCACTGATTGAAAAGTTGGTAAA
AGAAGGCGAGTTCCAACGAAGACTGATTGATGTGCATTGGCCACTGTCTGAATTCTACACCGTGCAACACCAACT
CGAAT
>Locus_2545_Transcript_27/30
TTCAATACTGCATTAGCTTGCTGTAATATAAACAGTCTGATGGTCTTAACTCACAATTGATGGTAAGTTTTTTAC
CTTAAAGGAAAATTTTGATAAACTTAGAACCCTGTGCAATGCAATATATATAAAAAGTAGTTGAACTGCACATCA
AACAAATAGGTCAAAGAAATCATGAAAAAGAAAGGCGAGCAAAAAAGGATAAGTTTTAAAGGCGCAATTCAAGTC
TGTCACAAAATTAGGAATATTTTACCAAGCAACCAAGCAAGCAAGAAACCAACCAAAATGAGAAATCTCTCTGGC
ACACTTTCAAAAATTTAATTTGATCAGTCTCGAGGTTACATACTAATTAACATGCGCCTCTGAGCACTGTAAGGG
ATCTGATTTCATCAACACAGTCCTCTGTGCTGCATATAAAACAAAAATGTCATTCCCACTAGACCCAGCAGCACT
GATAGTATTTTATAACATGAAGGCCCAATTGTCCTCTGTCCTCACTTTGTCAATTAGTCGTACATATCTTTCATG
CCCCCCAAAAATGGGGGAGGGGGGCATGTCCCCCCGGCTTCTACCCCCTGATGACGTTCCCCAGGGATGAACTGA
CAAATAAGGCAGTGCCCAAGGACTCAATCTTTGTCAGGTGGAAAATAAGAGAAAGGAACCAACCTCGACCAGTTA
ATTTATTCAATTTTCTGGCAATGAAATAATCAGCCTCTCATACTAGTTTGGGACTCTTTTTTCAGCCTCGCTATG
CTGAGAATGTCCAGGTTGACAGCTGACCACACCCCCCCCTTTAAAGCCCAACTTGGAAAGATATTCTTGAATGCAG
TGTAACTAATTTCACAACCCTGATTTATTAAAAAATTTAATAAAAAAGAATATTATTTGCATTTTAAAGCTATAA
AAGTGCTTATACATGGGGGATCTTTTCCCAGTCCTTGTCGGAGACACTTTCTGCTGAGAACGTCCTGGACGAGGC
GGGGTAAAGCTGAGCGTGCCTCCCCAAGGCCCAGATTGGAAAGATATTCTTGAATGCAGTGTAACCAATTGCACA
AGATTTATTAAAAACTCCGCATATGCTCTCCTGAGGCCAGTCGCCATCATCGATCTGTCTATCGAGTAAGACTTT
GACACCTCTCGACAAAACCTCTTCGTCAGGATACCTCACTGCCATTAGTCCCAGAAGAGCCCAAGCCGTATTGAC
GACCAGTGATTTCTCACTCTGCACGTATCTCCTCTCGCTGCAGGAGGCAAACTTCTCACCCCAACCTCCGTCCTC
CATCTGCCTCGAGACTAGAAACGAGCAGGCTTTCTTGACTTGCATGGAGGCAGTGTTATCATTGTAATTCTGACC
CATGCAGGCAAAAGCTTCTAGTGCAAACCAGGTTCCATACGTAAAACAGACTCCCCAGGAACCTTCAAATGAGCC
ATCAGGGAGTTGATTTTGTTTGATATATCCCAGGCCATTTTTCAGCACATTCCAGATCTCGTCTGTTCTATAGTT
TGGATCGTAGTCCACGAAATGTCTCAGAGCTTGTAAGACCGAAGATGTGCACTCAGTGTAGGTGTAATCCACCAT
GATGTCACCGAATACTTCAGAGGGATTAAGTAACTCCAACAACTTTTCTCCGCCTCGAAGGGTCTCGTAACTGGA
GTACCCGCCGTTCGGGTTAACCATGTCTAGAAGCAGGTCGACTGCTTCGTGAAGACGCTCTCTGGATATTTTCGC
TTCGCTACCGATGAAGGGACATTTCTCTTCGATCAGCATTGCGGCTTTCAATGCCTCGGCTGTCGTATCCGACAC
AATCAGACCGTGATCTCTCGTCGTGAGAGGCCAGCCTCCTTTGTTATATTGGCGATAATACTGCACACATTTTGG
AGAGCTCTCAACCATCTGTGAAACTTCAAGATAAGAGTAAGCTTTCCTCAGAGCCTCTTGAAACTCCGGCTTGGT
ACCGGCGCCCGCCTCGAGCATAGCCATCGCAGCATAGGAGATGTCCCATATCTGGGTTCCGTTTGTACCCTGCAT

ATTTACGCCATCTCGGCCCATCCAGATGTAGTCATAGACCCTATCCACATGTTGCAGAAATGCTTTCGAATCTGG
ACCGTCTTCAAGCCACCGAATCAGCATATTGATCATTTTGGAGATTGGCCCAATACTGATGAAGTTGGTCATAAT
ATCATCTTGTTTGATATGATCCAAGCAAACCTTGAGAGCACTTTCTCTGTACCAGCTGCTGTGAAAGGGTTCATA
GGTATCTAACATCCCGAATACCATGGAGTAAAGCCAGCTATGAGGAGTGTAAATGTCAATTTTGGCAATGTTTTC
TCTCTGTGCCGGCCAATCAATCGTTTCAAAGTCCTCGACGAATAATTCCTTTCTCAGGCTTAAGATAAGACTATC
CTCTGGAGCGTAGTATCTTTTGCCGTAGAGATACGACATCCCAAGATAAACGATACGGCAGTGAACCCAGAGTGT
TGATGGATGGGCGGGTAACCACGAGGGCAGCAACCATAGTTCGGGGAACAGCGTGTGCATGCCTTCCCACTTATA
ACAGTTCAGTATGCACAACCAAAACTTCCCCCAGGATGGGATGGTGGCAGCCCCACCATGGTGATGGAGAAGCTT
CCTGGCTTTAACCAAGTCCTGGTCTTCTGGGCTGACACCAAGGAGTCTCATCACCACATAGTTCAAAGCTGTCCC
AAAGACTGTGGCATTATCTTCTGTATGCAGACCCCAGCCTCCGTCCGCAAGCTGTACTGATCTCAAGTAACGAAT
GGACTCTTTCACGAAAGCCTCCGGTAGTTTGGTCTTGGTGATGTAAAGTACTATAATGAGACCTGGCATCAAAAA
CAGAGGACCTGAATAATCATTAGGCCAATGACCATCGTCGCATTGCAGCAACGAGAAAAACTTCATCCCGTTGAT
GGCCGCCTCTGACGCGGTCTCCGCATCCGTTAGCTTATTTGCCTCATCCGAATAGTCAAGTCCCAACGAATATTT
TTCAATTACGTTCTGCTCCCTTTCGAGTGTACCATCAATAGATATAAAGTGCCAGCGTCTTCTGCCATCTATGCT
AGTAAGTCTCCATCTGGTTAGATCAGTCACGGGATTAATTCTCCTTGACCCAGACATAGCTTTGATAACTGTCAG
CTTCCAGGGTCTACGTTTGATTAACGGGAATTCCCTACCAGGTTGCTGTTCGTCGTTCATGGAACGCCGTAGAAG
TTGAACTGAATATAGGAACGGGCAGTCTGAAGT
>gi|638417372|gb|GAVS01052291.1|
TCAGACGTCTCTTGGAGACCCTCTCGGTGATGAAGGGACATTTATCGTTCATCAGGAGGACCGCTTTCAGAGCCT
CTGCCGATGTGTCGGACACCACTAAGCCGTGCTCCCTCATTGTCTGAGCCCATCCTCCCTTGTTGTATTGCCGAT
AGTATTTGACACAATCTGGTGAACTTTCTATCACTTGCGAGATTTCAAGATATGAATAGGTTTTACAAAACACCT
CGTGGAATTGTGGGTCGTCCTGCGCGCCCACATCGAGCATAGCCATAGCAGCAAAGGAAGTGTCCCAAACTTGAT
TACCGTTAGTGCCCTGCACATTTGTGCCATCAAGACCCATCCAAACATAATCATACACTCTCTCCACGTGTTTCT
TGAAGGCCTCAGACTCGGGTCCATCCTCCAGCCATCTAATGAGCATGTTAATCATCTTTGAAATTGGACCAATGC
TTATAAAGCTTGTCATGAGATCATCCTGTTTAATATGATCAAGGCAGACATCGAGTGCTTGTTTCCTGAACCGGG
TCAACCTAAACGGCTCATACTTATCTAAAATACCAAAGGCAATGTTAAACAACCAGCTATGCGGAGTATATAAAT
CTATCTTAGCGATATTGTCTCTCTGGGATGGCCAGTCAATTTGGTCGAAATCTTCAATAAATAATTCCTTCCTAA
GGTCCATTATCAGTTCATCTTCTTGCGCGTAGTACCTCTTACCATAGAGGAATGCCATACCGATGTAAACCATTC
GACAATGAATCCAGAGAGTGGACGGGTGTGCTGGAATCCAGGAGGGCATTAACCATAGTTCAGGAAACATTGTGT
GCATGCCTTCCCATTTATAACAATTGAGTATGCACAACCAAAACTTCCCCCAGGATGGGATGGCAGATGCTCCTC
CATGGTGATGGAGGACCTTCCTTGCTCTTTTCATGTCTCCATCTTCTGGGCTCACACCGAGGAGTCTCATGGAGA
TGTAGTTCAACGCTGTACCAAAAACGGTGGCATCGCCCTCAATATGTAAACTCCACCCTCCATCTTTAGCCTGAA
CTCTCCTCAGATACCTGACAAACTCTTGTTTGAATGCTTCAGGAAATTCAGTCTTGGTAATGTAAAGTACAATGA
TTAGACCTGGCATTAAAAACAACGGCCCTGAATAATCGTTGGGCCAGTGACCATCTTCTGCTTGCATTAAAGAGA
AAAATTTGATTCCATTCTCAGCAGCTTCCTTCGGGTTTTGAGCTCGAGGGAGTTTCTCAGCTTCATTAGAGTAAT
CCAGTCCAAGCGAGTACTTCTCGACCATATTTTGTGGTCTTTCTGGAGTCTCGTCCTCTGTTACGTAATGCCAGA
TTCTCTTTCCTTCTGTACAGGACAGCCGCCATCTTGTAAGATCGGAATACTCACTAATTGTGCGTTCAGAGCGAG
GTTTGTGGTCATTATCGGACTGCATTGTTTATGTAAGACGTACGTCTTTAACTTCTTCTCATCCAGAATAAAAAC
AAAACTGGACTACCGATAAAACTGATGAATGATTAACTTAGAGGCCCCGTCTGCTAGAAGAAATCACTGATTGAA
AAGTTGGTAAAAGAAGGCGAGTTCCACGAAGACTCATTGATGTGCATTGGCCACTGTCTGAATACTACACCGTAC
AACACCAACTCGAATTTAAACCTTTTTTTTTGTCGTCTTTCTTTGGACTGTCATGCCAACAGTAACCTGTTCAT
G
>gi|638417366|gb|GAVS01052297.1|
GGGCCGAGATGGCGCAAATATGCAGGGTACAAATGGAACGCAGATATGGGACGTCTCGTACGCTGCGATGGCTAT
GCTTGAGGCCGGTGCTGGTACCAAGCCGGAGTTTCAAGAGGCTCTAAGGAAAGCGTACTCTTATCTTGAAGTTTC
ACAGATGGTTGAGAGCTCTCCAAAATGTGTGCAGTATTATCGCCAATATAACAAAGGAGGCTGGCCTCTCACGAC
AAGAGATCACGGTCTGATCGTGTCCGATACGACAGCCGAGGCATTGAAAGCCGCCATGCTGCTCGAAGAGAAATG
TCCCTTCATCGGCAGCGAAGCGAAAATATCCAGAGAGCGTCTTCACGAAGCAGTCGACCTGCTTCTAGACATGGT
TAACCCGAATGGCGGGTACTCCAGTTACGAGACCCTTCGAGGCGGAGAAAAGTTGTTGGAGTTACTTAATCCCTC
TGAAGTATTCGGTGACATCATGGTGGATTACACCTACACCGAGTGCACATCTTCGGTCTTACAAGCTCTGAGACA
TTTCGTGGACTACGATCCAAAGTATAGAACAGACGAGATCTGGAATGTGCTGAAAAATGGCCTGGGATATATCAA
ACAAAATCAACTCCCCGATGGCTCATTCGAAGGTTCCTGGGGAGTCTGTTTTACGTATGGAACCTGGTTTGCACT
GGAAGCTTTTGCCTGCATGGGTCAGAATTACAATGATAACACTGCCTCCATGCAAGTCAAGAAAGCCTGCGCGTT
TCTAGTCTCGAGGCAGATGGAGGACGGAGGATGGGGTGAGAAGTTTGCCTCCTGCAGCGAGAGGAGATATGTGCA
GAGTGAGAAATCACTGGTTGTCAATACGTCTTGGGCTCTTCTAGGACTAATGGCAGTGAGGTATCCTGACGAAGA
GGTTTTGTCGAGAGGTGTCAAAGTCTTACTCGATAGACAGATTGATGATGGTGACTGGCCTCAGGAGAGCATATG
CGGAGTTTTTAATAAATCTTGTGCAATTGGTTACACTGCATTCAAGAATATCTTTCCAATCTGGGCCTTGGGGAG
GCACGCTCAGCTTTACCCCTCCTCGCCCAGGACGTTCTCAGCAGAAAGTGTCTCCGACAAGGACTGGGAAAAGAT
CCCCCATGTATAACCACTTTTATAGTTTCAAAATGCAAATAAATATTCTTTTTTATTAAATTTTTGAATAAATCA
GGGTTTTGCAATTA
>gi|638417369|gb|GAVS01052294.1|

GGGCCGAGATGGCGCAAATATGCAGGGTACAAATGGAACGCAGATATGGGACGTCTCGTACGCTGCGATGGCTAT
GCTTGAGGCCGGTGCTGGTACCAAGCCGGAGTTTCAAGAGGCTCTAAGGAAAGCGTACTCTTATCTTGAAGTTTC
ACAGATGGTTGAGAGCTCTCCAAAATGTGTGCAGTATTATCGCCAATATAACAAAGGAGGCTGGCCTCTCACGAC
AAGAGATCACGGTCTGATCGTGTCCGATACGACAGCCGAGGCATTGAAAGCCGCCATGCTGCTCGAAGAGAAATG
TCCCTTCATCGGCAGCGAAGCGAAAATATCCAGAGAGCGTCTTCACGAAGCAGTCGACCTGCTTCTAGACATGGT
TAACCCGAATGGCGGGTACTCCAGTTACGAGACCCTTCGAGGCGGAGAAAAGTTGTTGGAGTTACTTAATCCCTC
TGAAGTATTCGGTGACATCATGGTGGATTACACCTACACCGAGTGCACATCTTCGGTCTTACAAGCTCTGAGACA
TTTCGTGGACTACGATCCAAAGTATAGAACAGACGAGATCTGGAATGTGCTGAAAAATGGCCTGGGATATATCAA
ACAAAATCAACTCCCCGATGGCTCATTCGAAGGTTCCTGGGGAGTCTGTTTTACGTATGGAACCTGGTTTGCACT
GGAAGCTTTTGCCTGCATGGGTCAGAATTACAATGATAACACTGCCTCCATGCAAGTCAAGAAAGCCTGCGCGTT
TCTAGTCTCGAGGCAGATGGAGGACGGAGGATGGGGTGAGAAGTTTGCCTCCTGCAGCGAGAGGAGATATGTGCA
GAGTGAGAAATCACTGGTTGTCAATACGTCTTGGGCTCTTCTAGGACTAATGGCAGTGAGGTATCCAGACCAAGC
TGTCTTAGAGAGAGGAATTCAAGTTTTACGTGATCGCCAACATGAAGACGGCGACTGGCCACAGGAAACCATTTC
TGGAGTTTTTAACAGGTCATGTGCGATTTCGTATCCGGCTTTTAAGAACATTTTCCCGATATGGGCTCTGGGACG
TTATTCTCAGCTTTACACGTCTTCCAAGGAAAACCAAGACTCTTGGGTACGAGATGACGATTGGGAAAAGTTGTC
AAACAACTGGCAGATTGCTAAATTGGTATTTTAGAGTACGAGAGATTAATACAGCATAAGAACGAACGGTTGGGT
GTATAAGAAGGTGGCGGCAAAAAGCGTTTTCATATTAACAAATTTCTTATCGAAACACATTTTTTTATGTATTAT
TTTTTTTAGGGGGGAGGGGAGGGGGGGTTGACATACCAAGACAAACATTCTGGTTACAATCATCCATTTAACAGTG
AAAGTTACATACGAATCTCTTGTTGCCATGGTAAGGCTACATAACATCACATTACGCATGTGCAGATGAAAACAT
AACAATAGCTGAAACCCTTACGTAGGCGTGACGTCTTGAACAAACCACTGTCAGGCGGGGAGAGGGGGGTTGGGGA
GAATTTTGTACTGCAGTGGACGTTTTGCACAAATTTCTGATATTTTGTTTTTCCTCATAAAAAAAATGTATCAAC
ATTTACCACGAACCTCTGCTCGTAGTGAAACTGATACCGGCCATACGTTGATGTAGTAATTAGTATACACCATTA
ACACTTTATTGCTCCCTATTGACTAATATTACTTGTATTATTATATTCATGCCAAGTTCCCAAGTAAACCTATAC
TGATCCACGACTCCGATGAAATCTGATTTTTGACTATTTAGTGTTAAGTAGTATTTAACGTGGCTAATAAAATTA
TAATTAGCAGCTAAATTCAGAGAAAAAACAGAAATTTGGATAAGTCTCACTGGGAATTAATTTCGAAATATTCTA
AGATATTATGCTATTCTATGCTTATTCTATATGATATTCTTAATATCTGCACATGCAATCTCCAAGGTTAAGCGC
GGGCCATGCACGTGTCACTCTGAAAAGGGAAAATACTTCCATGCCCTGTGGATGCTTCGGAATAGTTGGTCTTTG
ACTTCCCCTGGTTTAGTTCAGATGTTGTTTATACGTGAGTGTGTGGAAACGTTACAATGTCGTCAAAATTTTGGA
ATCTTTTATATTAAAAATATGGTAGCAATTTTCAACAAAATGGTTATTTTTTTCAGTTCACATATTTATTTCACA
TTTTTGCAACAAAATGGCAGATTCCCTAAATCTGATAAGTGATAAGCTCGACATTGTGCTAAAAAATGATTACAC
ATGCCAGTATATGTTATGATGTCAACAATTTTTAACAGATTGATTACTTATAGTAATCAAGCTCGTCATCCAACA
ATTTTTGACAAAAAAAATTATTAATTACGCTCGCGTCATCATGTCATCATTTCACAATTTTTGCAGCAAATTTAC
CGACGACTTTTTAACGGCACCTTAAGGTTGCCATACTTGAAAGAGTGCAGTTACCTTAAGGTTGCCATACTTGAG
AGAGTGTATATAGTTCCTTTAAGTTGCCATACTTGAAAGAGTGTATATTTACCTATATAGGTGGTCATACTTGAA
AGAGTGTA
>gi|638417371|gb|GAVS01052292.1|
CGCCATCTCGGCCCATCCAGATGTAGTCATAGACCCTATCCACATGTTGCAGAAATGCTTTAGAGTCTGGACCGT
CTTCAAGCCACCGAATCAGCATATTGATCATTTTGGAGATTGGCCCAATACTGATGAAGTTGGTCATAATATCAT
CTTGTTTGATATGATCCAAGCAAACCTTGAGAGCACTTTCTCTGTACCAGCTGCTGTGAAAGGGTTCATACGTAT
CTAACATCCCGAATACCATGGAGTAAAGCCAGCTATGAGGAGTGTAAATGTCAATTTTGGCAATGTTTTCTCTCT
GTGCCGGCCAATCAATCGTTTCAAAGTCCTCGACAAATAATTCCTTTCTCAGGCTTAAGATAAGACTATCCTCTG
GAGCGTAGTATCTTTTGCCGTAGAGATACGACATTCCAAGATAAACGATACGGCAGTGAACCCAGAGTGTTGACG
GATGGGCGGGTAACCAGGAGGGCAGCAACCATAGTTCGGGGAACAGCGTGTGCATGCCTTCCCACTTATAACAGT
TCAGTATGCACAACCAAAACTTCCCCCAGGATGGGATGGTGGCAGCCCCACCATGGTGATGGAGAAGCTTCCTGG
CTTTAACCAAGTCCTGGTCTTCTGGGCTGACTCCAAGGAGTCTCATCACCACATAGTTCAATGCTGTCCCAAAAA
CTGTGGCATTATCTTCTGTATGCAGACCCCAGCCTCCGTCCGCAAGCTGTACTGATCTCAAGTAACGAATGGACT
CTTTCACGAAAGCATCGGGTAGTTTGGTCTTGGTGATGTAAAGTACTATAAAGAGACCTGGCATCAAAAACAGAG
GACCTGAATAATCATTAGGCCAATGACCATCGTCGGCTTGCAGCAACGAGAAAAACGTCATTCCGTTGATGGCAG
CCTCTGCCGCGCTCTCCGCATCTGGGAGCTTATTTGCCTCATCCGAACAGTCAAGTCCCAACGAATATTTTTCAA
GTACATTCTGCTCCCTTTCGAGTGTACCATCAATAGATATAAAGTGCCAGCGTCTTCTGCCATCTATGCTGGTGA
GTCTCCATCTGGTTAGATCAGTCACGGGATCAATTCTCCTCGACCCAGGCATAGCTGTCAGCTTCCAGGGTCTAC
TGTACGTTCGATTAACGGGAATTCCCTACCACTG
>gi|638417368|gb|GAVS01052295.1|
TCAGACGTCTCTTGGAGACCCTCTCGGTGATGAAGGGACATTTATCGTTCATCAGGAGGACCGCTTTCAGAGCCT
CTGCCGATGTGTCGGACACCACTAAGCCGTGCTCCCTCATTGTCTGAGCCCATCCTCCCTTGTTGTATTGCCGAT
AGTATTTGACACAATCTGGTGAACTTTCTATCACTTGCGAGATTTCAAGATATGAATAGGTTTTACAAAACACCT
CGTGGAATTGTGGGTCGTCCTGCGCGCCCACATCGAGCATAGCCATAGCAGCAAAGGAAGTGTCCCAAACTTGAT
TACCGTTAGTGCCCTGCACATTTGTGCCATCAAGACCCATCCAAACATAATCATACACTCTCTCCACGTGTTTCT
TGAAGGCCTCAGACTCGGGTCCATCCTCCAGCCATCTAATGAGCATGTTAATCATCTTTGAAATTGGACCAATGC
TTATAAAGCTTGTCATGAGATCATCCTGTTTAATATGATCAAGGCAGACATCGAGTGCTTGTTTCCTGAACCGGG
TCAACCTAAACGGCTCATACTTATCTAAAATACCAAAGGCAATGTTAAACAACCAGCTATGCGGAGTATATAAAT

CTATCTTAGCGATATTGTCTCTCTGGGATGGCCAGTCAATTTGGTCGAAATCTTCAATAAATAATTCCTTCCTAA
GGTCCATTATCAGTTCATCTTCTTGCGCGTAGTACCTCTTACCATAGAGGAATGCCATACCGATGTAAACCATTC
GACAATGAATCCAGAGAGTGGACGGGTGTGCTGGAATCCAGGAGGGCATTAACCATAGTTCAGGAAACATTGTGT
GCATGCCTTCCCATTTATAACAATTGAGTATGCACAACCAAAACTTCCCCCAGGATGGGATGGCAGATGCTCCTC
CATGGTGATGGAGGACCTTCCTTGCTCTTTTCATGTCTCCATCTTCTGGGCTCACACCGAGGAGTCTCATGGAGA
TGTAGTTCAACGCTGTACCAAAAACGGTGGCATCGCCCTCAATATGTCTGTTAAAATACAAGTACCCTTCCTTGC
T
>gi|638417367|gb|GAVS01052296.1|
GAGAGGGTCTCCAAGAGACGTCTGAGAGATGCAGTGGACATGTTATTGACTATGGTAAATCCAAACGGGGGTTTC
TCGTCGTATGAAAATCTTCGCGGAGGAACAATCCTGGAGTTATTAAACCCCTCCGAAGTATTTGGTGACATCATG
GTGGATTACACCTACACTGAGTGTACATCTTCAGTCTTACAAGCTCTGAGACATTTTGTCGATTCTGATCCTGAC
TACCGCCAAGATGAGATATGGGCTGTCCTTAGGAACGCAATGGAATACAAGATCCAATCAGTTACCGGACGGTTC
CTTCGAGGGATCCTGGGGTGTGTGTTTCACATATGGTACATGGTTCGCTTTGGAGGCGTTTGCCTGCATGGGGAA
GAACTACCAAGACAACACTGCTAGCATCGACGTCAAGAAAGCCTGCGCGTTTCTAGTCTCGAGGCAGATGGAGGA
CGGAGGATGGGGTGAGAAGTTTGCCTCCTGCAGCGAGAGGAGATATGTGCAGAGTGAGAAATCACTGGTTGTCAA
TACGTCTTGGGCTCTTCTAGGACTAATGGCAGTGAGGTATCCTGACGAAGAGGTTTTGTCGAGAGGTGTCAAAGT
CTTACTCGATAGACAGATTGATGATGGTGACTGGCCTCAGGAGAGCATATGCGGAGTTTTTAATAAATCTTGTGC
AATTGGTTACACTGCATTCAAGAATATCTTTCCAATCTGGGCCTTGGGGAGGCACGCTCAGCTTTACCCCTCCTC
GCCCAGGACGTTCTCAGCAGAAAGTGTCTCCGACAAGGACTGGGAAAAGATCCCCCATGTATAACCACTTTTATA
GTTTCAAAATGCAAATAAATATTCTTTTTTATTAAATTTTTGAATAAATCAGGGTTTTGCAATTA
>gcontig_1106221709355
ATGCCGCACGATCCCACTGCCCGCGCGGCCGCGCGCCTGACCGAGACCCAGCAGCCCGCCGGGTGCTGGGAAGGC
GAGATGATCTGGTGCCCGGTCGTCACGGCACAAGTCGCGATCACGCGGCACGTTGTCGGGATGCCCTTCTCGGAC
GCCGACGCGGCCAAGATCATCCGGCACTTCGAGTTCTCCCAACTGCCGAACGGCGCGTTCGGCTTGCACCCGGAA
CACCCCGGCTCGGTGTTCGTCACCACGCTCGTGTACGTCGCGGCGCGGTGCCTCGGTGTGAGTGCAGAACACGCC
GTCACCGCGAAGGCGCGCGGCTGGCTGCACGCGCAACCGGGCGGGGTGTTGTCCGCGCCGACGTGGGGTAAGTTC
TGGCTCACGCTGCTCGGGCTGTACGGCCGCGACGGGCTGCGCCCGCTGCTCCCTGAACTGGCGCTGCTACCCAAA
GCGTTCCCCGTCCATCCGGTCCGTTTTTACTGCCACACGCGGTACGTGTACCTGGTGATGTCGCTGCTCCAGGGC
GCGCACGCGACGTTCGACCTCGGCCCGCTTCGCGCGGAACTGGAACGCGAGTTGTACGGCCCGCTGGCGGTGCCC
GAGTCGTTCCGCCAGTACCGCTACCGCCTCGCCGAAACCGACGCCTTCGAGCCGCCGAACCTGTTCATCCGGGTC
GCCGAGCGTGTGATGGGCTGGTACGATCGCGTTGCGCTGCCCGGGCTGCGCCGCGCGGCACTCAAGCGGTGCGCG
GACCTGATCGACCTGGACCTCGACGCGAACGGGTATCTGACCCTGTCGCCGGTGAACGGCACGCTCAACGCACTC
GCGCTGTTCGCACGCGGCGCGGACCGCGAGGTGATCGCCAAGTGCGTGAGCGGGTTCGAGTTCTATCGTTTCGAC
GACCCCGAGCGCGGGCTGCGGTACTCCGGCGGCAGTACACGCACCTGGGACACGGGGTTCGCCTTGGAGGCGCTG
CTCGCGAACCCGGCGGTGGCCTCCGTGTACCGCGATGTGGTCCATCGGGGCTATCGGTTCCTGGCCGCGCACCAG
ATGTCGAAGTCGGTTGCGGGTCGCGATCCGTCGTTCCCGGACACCGCCCGCGGCGGCTGGTGCCTTGGCGACGGG
GGGCACGCGTGGCCGGTGAGTGATTGCACCGCGGAAGCGCTCTCGGCCGTTCTGAGTGCGCACACGCACGGCATG
GCACCCGAGGAACGCATCCCGGACGCCCGGCTGATCCAGGCCGCCGAGTTCATGCTGACGCGCCAGAACCGGGAC
GGCGGGTTCGGCTCCTACGAACGCGCCCGCAGCCCGCGCTGGCTCGAACGGATGAACCCGTCGGAGATGTTCACC
CGGTGCATGACGGACCAGTCGTACATCGAATGTACGGGGTCGTGTCTCGTGGCGCTCGGGCGGTTCCGAAAGGCG
ATCCCGCACCACGCGGCCGGACGCATCACCCGGGCGACGAACCGCGGCGCCCGGTTCCTGTTGAGCCGGCAGCGC
CCGGATGGCGCGTTCCCCGGAGCGTGGGGGGTGTACCTCACCTACGGAACGTTCCATGCGGTGCGCGGGCTTCGT
GCCGCTGGCTACGCGCCCTCCCACCGGGCACTTCAACGGGCCGCGAACTGGCTCATCGCTACCCAAAAGCGGGAC
GGCGGCTGGGGCGAGGACTACCACGGTTGTCTTCGGCAGGAGTACGTCGAGCACCCCGAGTCGCAGGCCACGATG
ACGAGTTGGGCGATTGTAGCGTTGTGCGAGACCGTCGGGACCGGGCACCCGGCCGTGCAAAAAGGGGCCGCGTGG
CTCGCGTCACGCCAGCGGGCCGACGGCTCGTACCCGCGCGAGGCCGTTAATGGGGTGTTCTTCGGAACGGCGATG
CTCGATTACGATCTGTATCGCGCGTACTTTCCGACATGGGCGCTCGCACTGGCTTCGGGTACAACTGCGAAAAGT
TGA
>oki.2.251.t1,
ATGAGTAGTCGACGCAACAGAGGAGGCCCACACAAGACACAGCCAGTCACTGACCTAACGAGATGGCGTCTGTCA
AACGTCGATGGCCGACAATGCTGGAGGTACATCGAGGAAGGCCAACCCATCGAAAGAGAGCAGAACTTTGTGGAA
AAGTTCAGCTTAGGACTGGATATTAGCAAAGAGGCTCCACCCCTCCCTAAGCCTACCACGCCCCAAGAGGTAGCA
ACAAACGGGATGCTTTTCTACTCCAAGCTTCAGACAGAGGATGGCCACTGGAGTGGAGACTATGGTGGCCCTCTC
TTCCTCATGCCAGGACTCCTGATTGTGTGCTACATTACCAAGACAGAGTTACAGGATGCAGTCAAGAAAGAGATG
GTCCGGTACCTGCGCTCCGTACAATGCCCGGATGGTGGATGGGGATTACATATCGAAGGCCCAGCTACAGTCTTT
GGTTGTGCCACTAACTACGTGGCCATGAGATTGCTCGGTGTGTCTGCTGATGACCCAGATCTGGTCAAATGCCGG
AAGTTACTTCATTCATTAGACTATAACAGCGTCTTTGGCCTTATCAAACTGAGCATGCTGTTTCCGACGTGGATT
CCAGCCCATCCATCCACCATCTGGTGCCATTGTCGGCAAGTCTACCTACCCATGAGCTACTGTTACGGGGTCAAG
TATCAAGCTGAGGAAGATGATTTGATCAGAGAACTGCGAAAGGAACTCTACATACAAGATTACAATACCATCAAT
TGGCCTGCACAGAGGGACAACATCGCTAAGATAGATCTCTACACACCCCACAGCTGGCTCTACAATCTAGCTTTT
GGTTTCCTTGACGTCTACGAGCCGTATCATTCCACTTACTTTCGCCAGATGGCTCTTGATGAGTGCCTTGATCAC

ATTCGAGCTGATGATCAGTTCACCAAGTGCATTAGCATTGGGCCTATTTCTAAAGTCATCAACATGTTGGTGAGA
TGGTTGACGGATGGACCTGACTCTCAGGTCTTTAAACAACACGTGGAACGTATCCCAGATTACCTCTGGATTGGT
TTAGATGGCATGAAAATGCAGGGAACCAATGGGTCACAGCTCTGGGACACGGCTTTCGCTGCCCAGGCCTTCTTG
GAAGCTGGAGCAGCAAAGAACAAGGAGCTACATGATTGCCTGCAGCACACCCATGACTTCCTGAAGCTTACACAG
ATCCCTGACAATCCACCCAACTATCAAAAGTACTATCGTCAAATGAACAAGGGGGGCTATCCATTCAGTACCCGT
GACTGTGGCTGGATTGTAGCTGACTGTACGGCGGAAGGTCTCAAGGCAGCATTGATGCTGGAGACAATGTGCCCC
TCTGTCAGAGACCATATAGAGAAGGAGAGGCACTGTCAAGCTGTGGATGTGATTCTGAATATGCGTAACAAAGAT
GGAGGATTTGCGACCTACGAGACCATGCGAGGGGGAGTGATCCTTGAAAAACTTAATCCATCTGAAGTGTTTGGT
GATATCATGATTGACTACACCTACGTGGAGTTGACCTCAGCTGTCATGCAAACCTTGAAGAAATTCAACACCCTG
TATCCTGACTATCGCAAGGAGGACATCAGATCCACGTTGGACAAGGGCTTGAAGTACATTGCCATGAAGCAAAGG
ATTGATGGATCCTGGGAAGGGTCGTGGGGTGTGTGCTTTACCTACGCCGGCTGGTTTGGTCTGGAAGCCTTTGCC
TGCATGGGATACAGCTATGAAACAGGGGCGGTGACCATGCAGATCAGGAGGGCGTGCATGTTCCTCAAATCCAAG
CAGATGGAAGATGGCGGATGGGGGGAGGAGTTTGAATCCTGCGAAGAGAGGCGGTACGTCCAAAGCAAGACATCA
CAGGTCGTCAACACGGCTTGGGCGGTCCTAGCTCTCATGGCAGTCAGATTTCCAGAGCGTGATGTCATTGATCGA
GGCATCAAAGTTATTATGGACCGACAGCTACCAAATGGAGATTGGGCGCAGACAACCTCCTATGAAGGCTGTGGG
TATGCCCCAGGAAAAGCAGACCATGATGCTGATGAGAAGAGCGCCCTCGCCTCCTTCAACCTGGAGAGAAGGAAG
ATGGAGGACAAGACCCTATGA
>1621490
AGGTTACGATTATCATCGAAACCACAAATTGACAAGGTTTATGCAAAGAGCATTTTACTAGTTGTAAGACACATG
TTACTTAAATATAAATACAGTCTATTCACTCAGAAAGTAGACCCCAAATATCAGAACAAAATCCATTTTCCAGGC
ACTGTCTGAGTTTTAGCCTATGGCATTAAGATAAAACAAAAGCATGACTTCTCAGAAATTTGAACATTCAAGATA
TGTCACTGGTCAGTTGGCCTTCGGAGAAGAAACTGTCTACGTTGCTGATGGGTAGAGTTGTGCGTACCTCCCTAA
CGCCCATATTGGGAATATGTTTTTGAAGGAGGTGTAGCTAATAGCGCATGATCTGTTGAATACACCACTAATGGT
CTCCTGGGGCCAATCGCCGTCAGGAGTTTGTCGATCTCGTAAGACTTTGACACCTCTGGACAAGATGGTTTCATC
AGGATACCTGACAGCCATAAGACCCAGCAGGGCCCATGCAGTATTGACCACTAATGATTTTTCGCTTTGTACATA
TCTCCTTTCACTGCAAGAGGCAAATTTTTCACCCCATCCTCCATCCTCCATCTGTCTGGAAACAAGGAATGAGCA
GGCTTTTTTGACCTCAAGTGTGGCAGTGTTGTCATCATAATTCTGTCCCATGCATGCAAAGGCTTCCAGAGCAAA
CCAGGTACCATAAGTGAAACAGACTCCCCAGGACCCTTCAAATGAACCATCTGGCAGCTGGTTTTGTTTAATGTA
CTGCAGCCCGTTTCTGAGCACGTCCCAGATTTCCCCTTGACGATAGCTGGGGTCGTAGTTGACAAAGTGTCTCAG
AGCCTGCATGACAGAGGATGTGCACTCTGTGTAGGTGTAGTCAACCATTATGTCACCAAAGACTTCAGATGGGTT
CAGCAGTTCCAAGACCTTATCACTGCCTCTAAGGATCTCATATGTGGCAAAGCCTCCATTGGGATTTGCCATAGC
AAGAAGCAAGTCAACTGCATCATGAATACGTTCTCTGGATATTCTGGCACTGCTTTTGATGAAAGGACACTTTTC
CTCTATGAGAAGAGCGGCCTTTAGTGCTTCTGCCGTGGTGTCTGATACTATCAGTCCATGGTCTCTTGTAGTAAG
AGGCCAACCTCCCTTGTTGAATTGCCGATGATACTGCAGACACTTTGGAGAGTTCTCAACCATCTGGGAGACTTC
GAGATACAAGTAAGCTTTTTGCAATACATTCTGAAACTGGGGTCTCAAGTTGGCGCCACACTCCAGCATTGCCAT
CACAGCAAAGGAAACATCCCATACTTGTGTTCCATTGGTACCCTGCATGTTGACTCCATCTCGGCCCATCCAAAT
GTAGTCATAGACCCTGTCGATGTGTTGTTGGTATGCTTTGGACTCTGGACCATCTTCTAACCACCTGATAAGCAT
GTTCATCATTTTGGACACTGGACCAATGCTGATAAAGTTGGTCATGATGTCATCTTGCTTTATATGATCCAAGCA
GATTTCCAAGGCCTTTTCCCGATACCACTTATGATGGATAGATTCATATATATCTAAAAGCCCAAATGCCAAAGT
GTGAAGCCAACTGTGAGAGGTATACAGGTCAATCTCTGCAATGTTGTCTCTCTGAGAGGGCCAATTGATGTGATC
AAAATCCTCCACAAATAGCTCCTTTCGTAAGCTGATAATGAGGTCATCTTCTGGTGCATAAAAGTGCTTGCCATA
CAAGTATGCCATGCCAATATATACTGCACGACAGTGGACCCACAGGGATGATGGATGTGCTGGAACCCATGTGGG
CAATAACCACAATTCGGGAAACAATGTGTGCATTCCTTCCCATCTGTAACAGTTAAGAATGCACAGCCAAAATTT
TCCCCAGGATGGAATGGAAGCAGCACCTCCATGGTAATGAAGGAACTTCCTGGCTCTTTGGAGATCCTCATCTTC
AGGACCCACACCAAGAAGTCTCATGGTCACGTAATTTAAAGCTGTGCCAAAGACTGTACCCTTATCTTCTGTGTG
CCTGATCGAGAGAAAATCGCACAAGGTACATTGAACCTGTTTCTATTAAAAGCGAAGTAATAATTGCCAAAAATC
GGTTCATTTTG
>1593798
GGTACCATAAGTGAAACAGACTCTCTCCTTTCACTGCAAGAGGCAAATTTTTCACCCCATCCTCCATCCTCCATC
TGTCTGGAAACAAGGAATGAGCAGGCTTTTTTGACCTCAAGTGTGGCAGTGTTGTCATCATAATTCTGTCCCATG
CATGCAAAGGCTTCCAGAGCAAACCAGGTACCATAAGTGAAACAGACTCCCCAGGACCCTTCAAATGAACCATCT
GGCAGCTGGTTTTGTTTAATGTACTGCAGCCCGTTTCTGAGCACGTCCCAGATTTCCCCTTGACGATAGCTGGGG
TCGTAGTTGACAAAGTGTCTCAGAGCCTGCATGACAGAGGATGTGCACTCTGTGTAGGTGTAGTCAACCATTATG
TCACCAAAGACTTCAGATGGGTTCAGCAGTTCCAAGACCTTATCACTGCCTCTAAGGATCTCATATGTGGCAAAG
CCTCCATTGGGATTTGCCATAGCAAGAAGCAAGTCAACTGCATCATGAATACGTTCTCTGGATATTCTGGCACTG
CTTTTGATGAAAGGACACTTTTCCTCTATGAGAAGAGCGGCCTTTAGTGCTTCTGCCGTGGTGTCTGATACTATC
AGTCCATGGTCTCTTGTAGTAAGAGGCCAACCTCCCTTGTTGAATTGCCGATGATACTGCAGACACTTTGGAGAG
TTCTCAACCATCTGGGAGACTTCGAGATACAAGTAAGCTTTTTGCAATACATTCTGAAACTGGGGTCTCAAGTTG
GCGCCACACTCCAGCATTGCCATCACAGCAAAGGAAACATCCCATACTTGTGTTCCATTGGTACCCTGCATGTTG
ACTCCATCTCGGCCCATCCAAATGTAGTCATAGACCCTGTCGATGTGTTGTTGGTATGCTTTGGACTCTGGACCA
TCTTCTAACCACCTGATAAGCATGTTCATCATTTTGGACACTGGACCAATGCTGATAAAGTTGGTCATGATGTCA

TCTTGCTTTATATGATCCAAGCAGATTTCCAAGGCCTTTTCCCGATACCACTTATGATGGATAGATTCATATATA
TCTAAAAGCCCAAATGCCAAAGTGTGAAGCCAACTGTGAGAGGTATACAGGTCAATCTCTGCAATGTTGTCTCTC
TGAGAGGGCCAATTGATGTGATCAAAATCCTCCACAAATAGCTCCTTTCGTAAGCTGATAATGAGGTCATCTTCT
GGTGCATAAAAGTGCTTGCCATACAAGTATGCCATGCCAATATATACTGCACGACAGTGGACCCACAGGGATGAT
GGATGTGCTGGAACCCATGTGGGCAATAACCACAATTCGGGAAACAATGTGTGCATTCCTTCCCATCTGTAACAG
TTAAGAATGCACAGCCAAAATTTTCCCCAGGATGGAATGGAAGCAGCACCTCCATGGTAATGAAGGAACTTCCTG
GCTCTTTGGAGATCCTCATCTTCAGGACCCACACCAAGAAGTCTCATGGTCACGTAATTTAAAGCTGTGCCAAAG
ACTGTACCCTTATCTTCTGTGTGCAAACCCCAGCCTCCTTCTTTCAGCTGCAGTGTCCTCAGATAACGCACAGAC
TCTTTCATGAATGCCTCTGGTACTTTGGTCTTTGTGATGTACAGGACAATAAAGAGACCTGGCATTAAAAAGAGC
GGTCCAGAGTAGTCGTTCGGCCAGTGTCCATCGTCAGCTTGAAGCAGAGAGAAAAATGTCATCCCATTTGTGG
>1619393
AACATTGGTTTTAAAAATATGATTTATAATATTTATGATATTTCCCTGTAAGAACAGACAGTGTTAAAAATTGAG
TAATGGGTTTAGAATGATTTTGTCACTCTTCGGCGTTGAGTCATTTTTATCAATATAGTGCAGAAGGCATCTTTC
CTCTTACGATTGGGAAAATATTGTTGTTTATTAAGGTGGTAAATGTAACTCCATTAAAAAGAACTATTTGCCTTT
GTTGTAATGTAAGCGAGTAGTAGTAACTATGTTTTTTGCTTTGCTTCTTATTTTCGGTAGGGGTAGAAGGAAGGC
GATCTCTTTCCTTTTCTTCAGCGTCAAATCCACGTTTCATCAATCCATGTTTCATCAAGAAAAAAGTACTGGCCT
CATCATCGATGTCAATTTAATATTTTTCGTGTGCTTACTCCTTTGGTGGAAGCATTGTGCATTCCCGTAAGAAAC
ACTCTTTTCGATTAAGAAGTCAAATTAATAATTATTCAGAAAAGATAGAAACATGAAACTTAAAAACATACTGTA
GGATTTTAATAATTATTCTTAAGTCGAGATCATTCTTCAAACTTGAGTTGTTCGAGATGTGTATGTTAATGTCAA
TGACCATATCAGAATACCCAACGGATTCTCTACCAACAAATCCGCCATGTGCAACAGTAATCTTGTGATCGTGTT
ATATTTCTGATCTTTTGCTCTTGCCGTCAATTGCAATTGTACCATAAAGTTGAGAATAACGTCCCAAAGCCCATA
TAGGAAATATATTTTTAAAAGCTGGATAAGAGATTGCGCAAGACTTGTTGAAAACTCCGGAAATGGTCTCCTGTG
GCCAATCACCATCATCCTGCTGACGATTCAATAGTACCTGGATCCCCTTATCCATGACATTCTTGTCCGGGTATC
CGACAGCCATAAGACCCAGCAGGGCCCATGCAGTATTGACCACTAATGATTTTTCGCTTTGTACATATCTCCTTT
CACTGCAAGAGGCAAATTTTTCACCCCATCCTCCATCCTCCATCTGTCTGGAAACAAGGAATGAGCAGGCTTTTT
TGACCTCAAGTGTGGCAGTGTTGTCATCATAATTCTGTCCCATGCATGCAAAGGCTTCCAGAGCAAACCAGGTAC
CATAAGTGAAACAGACTCCCCAGGACCCTTCAAATGAACCATCTGGCAGCTGGTTTTGTTTAATGTACTGCAGCC
CGTTTCTGAGCACGTCCCAGATTTCCCCTTGACGATAGCTGGGGTCGTAGTTGACAAAGTGTCTCAGAGCCTGCA
TGACAGAGGATGTGCACTCTGTGTAGGTGTAGTCAACCATTATGTCACCAAAGACTTCAGATGGGTTCAGCAGTT
CCAAGACCTTATCACTGCCTCTAAGGATCTCATATGTGGCAAAGCCTCCATTGGGATTTGCCATAGCAAGAAGCA
AGTCAACTGCATCATGAATACGTTCTCTGGATATTCTGGCACTGCTTTTGATGAAAGGACACTTTTCCTCTATGA
GAAGAGCGGCCTTTAGTGCTTCTGCCGTGGTGTCTGATACTATCAGTCCATGGTCTCTTGTAGTAAGAGGCCAAC
CTCCCTTGTTGAATTGCCGATGATACTGCAGACACTTTGGAGAGTTCTCAACCATCTGGGAGACTTCGAGATACA
AGTAAGCTTTTTGCAATACATTCTGAAACTGGGGTCTCAAGTTGGCGCCACACTCCAGCATTGCCATCACAGCAA
AGGAAACATCCCATACTTGTGTTCCATTGGTACCCTGCATGTTGACTCCATCTCGGCCCATCCAAATGTAGTCAT
AGACCCTGTCGATGTGTTGTTGGTATGCTTTGGACTCTGGACCATCTTCTAACCACCTGATAAGCATGTTCATCA
TTTTGGACACTGGACCAATGCTGATAAAGTTGGTCATGATGTCATCTTGCTTTATATGATCCAAGCAGATTTCCA
AGGCCTTTTCCCGATACCACTTATGATGGATAGATTCATATATATCTAAAAGCCCAAATGCCAAAGTGTGAAGCC
AACTGTGAGAGGTATACAGGTCAATCTCTGCAATGTTGTCTCTCTGAGAGGGCCAATTGATGTGATCAAAATCCT
CCACAAATAGCTCCTTTCGTAAGCTGATAATGAGGTCATCTTCTGGTGCATAAAAGTGCTTGCCATACAAGTATG
CCATGCCAATATATACTGCACGACAGTGGACCCACAGGGATGATGGATGTGCTGGAACCCATGTGGGCAATAACC
ACAATTCGGGAAACAATGTGTGCATTCCTTCCCATCTGTAACAGTTAAGAATGCACAGCCAAAATTTTCCCCAGG
ATGGAATGGAAGCAGCACCTCCATGGTAATGAAGGAACTTCCTGGCTCTTTGGAGATCCTCATCTTCAGGACCCA
CACCAAGAAGTCTCATGGTCACGTAATTTAAAGCTGTGCCAAAGACTGTACCCTTATCTTCTGTGTGCCCTGAAG
AACTGACTTCTGATGATAAGAAAGATAGAAGTGCCACAATTTCACCATCAAGGATGGGTTCCTCTTTCATGTTTG
ATCCAAGCAAGGTACGTTTTACTCTATTTTTTCTCTCATCATTGTACGTAATTCAACAAGCAGAAAGCCCAAGGG
CATTGTGGTATCGATGATTCAATTAGAAGTATTTGCTGAATATTGGTGAGGATGTGCAACATGCATGGTTATTAA
AATCTTTCAGTAATTTTCTGCATAAAAATTGTTACAACTATATATATATACACAACATCCGTTACCTTAAGATAA
TGACTGATAAGTAGCTGTGGGTCTATGTAACTCAGCATGCACACCTACATATGTAACAAATGATAGATATGTCCA
AGGATCTTGTTAATGGAATTCCATGTACACATGGTGTTAAAAGATTGACCTTTACTTAGGCCAAATGCCCTGAGC
CAGCAAATACAGT
>1579729
AACATTGGTTTTAAAAATATGATTTATAATATTTATGATATTTCCCTGTAAGAACAGACAGTGTTAAAAATTGAG
TAATGGGTTTAGAATGATTTTGTCACTCTTCGGCGTTGAGTCATTTTTATCAATATAGTGCAGAAGGCATCTTTC
CTCTTACGATTGGGAAAATATTGTTGTTTATTAAGGTGGTAAATGTAACTCCATTAAAAAGAACTATTTGCCTTT
GTTGTAATGTAAGCGAGTAGTAGTAACTATGTTTTTTGCTTTGCTTCTTATTTTCGGTAGGGGTAGAAGGAAGGC
GATCTCTTTCCTTTTCTTCAGCGTCAAATCCACGTTTCATCAATCCATGTTTCATCAAGAAAAAAGTACTGGCCT
CATCATCGATGTCAATTTAATATTTTTCGTGTGCTTACTCCTTTGGTGGAAGCATTGTGCATTCCCGTAAGAAAC
ACTCTTTTCGATTAAGAAGTCAAATTAATAATTATTCAGAAAAGATAGAAACATGAAACTTAAAAACATACTGTA
GGATTTTAATAATTATTCTTAAGTCGAGATCATTCTTCAAACTTGAGTTGTTCGAGATGTGTATGTTAATGTCAA
TGACCATATCAGAATACCCAACGGATTCTCTACCAACAAATCCGCCATGTGCAACAGTAATCTTGTGATCGTGTT

ATATTTCTGATCTTTTGCTCTTGCCGTCAATTGCAATTGTACCATAAAGTTGAGAATAACGTCCCAAAGCCCATA
TAGGAAATATATTTTTAAAAGCTGGATAAGAGATTGCGCAAGACTTGTTGAAAACTCCGGAAATGGTCTCCTGTG
GCCAATCACCATCATCCTGCTGACGATTCAATAGTACCTGGATCCCCTTATCCATGACATTCTTGTCCGGGTATC
CGACAGCCATAAGACCCAGCAGGGCCCATGCAGTATTGACCACTAATGATTTTTCGCTTTGTACATATCTCCTTT
CACTGCAAGAGGCAAATTTTTCACCCCATCCTCCATCCTCCATCTGTCTGGAAACAAGGAATGAGCAGGCTTTTT
TTGACCTCAATGCTTGCGGTGTTGTATTTGAAGCTTTTTCCCATGCATGCAAAGGCCTCTAGAGCAAACCAAGTA
CCATACGTAAAGCAAACTCCCCAAGACCCTTCAAAGGAGCCATCCGGTAGCTGATTGGACCTTATGTATTCTAGC
GAATTCTTCAGAACGGTCCAGATTTCATCGTGTCTGTAGTCGGGATCAGAATCTACGAATTGTCTCAGAGCTTGT
AAGACTGAGGAAGTGCACTCAGTGTAAGTGTAGTCGACCATTATATCACCAAATACTTCTGATGGATTCAGTAGT
TCTAGTATTTTTCCTCCTCGAAGATTTTCGTAAGATGAAAATCCACCATTTGGGTTCACCATTGTCAGCAACATG
TCCACGGCATCTCTCAAACGTCTCTTAGACACCCTTTTGCTGATGAAGGGACATTTCTCGTTCATCATAAGAACG
GCTTTCAAGGCCTCTGCAGAAGTATCAGAAACTACCAAACCATGTTCCCTCATAGTCTGAGCCCATCCGCCCTTA
TTGTATTGTCGATAGTATTTGACACAATCCGGGGCACTCTCTATAACTTGTGAGATTTCGAGATAGGAGTAGGTT
TTACGTAAAACTTCGTGGAATCTTGGGTCATCTTGGACCCCCACATCGAGCATGGCCATTCCAGCAAAGGAAACG
TCCCAGACCTGGTTTCCGTTCGTACCCTGCATATTTGAGCCGTCGAGTCCCATCCAGACATAATCATACACCCTC
TCAACGTGTTTCTTGAATGCCTCAGAGTCGGGTCCGTCCTCAAGGTATCTTATCAACATATTGATCATCTTTGAA
ATCGGACCAATGCTTATAAAGCTGGTCATCAAATCATCTTGTTTAATATGATCAAGACAAGCATCCAAGGCATTA
TTTCTGTATCTATTCAATCTGAAAGACTCATACTGATCCAATATACCAAAGGCTATTTTAAACAACCAACTGTGA
GGTGTATAAAGGTCCACTTCCGCAATGTTATTTCTCTGAGACGGCCAGTCGATTTCATCAAATTCCTCCAAAAAT
AATTCCTTTCTTAGTTCCAGGATCACTTCATCTTCTTGTGCGTAATACCGTTTACCGTAGAGGAAAGCCATCCCT
ATGTAAACCATTCGACAGTGAATCCACAGAGTAGACGGATGGGCTGGAATCCATGATGGCAGTAACCACAGTTCA
GGAAACAATGTATGCATTCCTTCCCATCTGTAACAGTTAAGAATGCACAGCCAAAATTTTCCCCAGGATGGAATG
GAAGCAGCACCTCCATGGTAATGAAGGAACTTCCTGGCTCTTTGGAGATCCTCATCTTCAGGACCCACACCAAGA
AGTCTCATGGTCACGTAATTTAAAGCTGTGCCAAAGACTGTACCCTTATCTTCTGTGTGCAAACCCCAGCCTCCT
TCTTTCAGCTGCAGTGTCCTCAGATAACGCACAGACTCTTTCATGAATGCCTCTGGTACTTTGGTCTTTGTGATG
TACAGGACAATAAAGAGACCTGGCATTAAAAAGAGCGGTCCAGAGTAGTCGTTCGGCCAGTGTCCATCGTCAGCT
TGAAGCAGAGAGAAAAATGTCATCCCATTTGTGG
>3222879
AGGTTACGATTATCATCGAAACCACAAATTGACAAGGTTTATGCAAAGAGCATTTTACTAGTTGTAAGACACATG
TTACTTAAATATAAATACAGTCTATTCACTCAGAAAGTAGACCCCAAATATCAGAACAAAATCCATTTTCCAGGC
ACTGTCTGAGTTTTAGCCTATGGCATTAAGATAAAACAAAAGCATGACTTCTCAGAAATTTGAACATTCAAGATA
TGTCACTGGTCAGTTGGCCTTCGGAGAAGAAACTGTCTACGTTGCTGATGGGTAGAGTTGTGCGTACCTCCCTAA
CGCCCATATTGGGAATATGTTTTTGAAGGAGGTGTAGCTAATAGCGCATGATCTGTTGAATACACCACTAATGGT
CTCCTGGGGCCAATCGCCGTCAGGAGTTTGTCGATCTCGTAAGACTTTGACACCTCTGGACAAGATGGTTTCATC
AGGATACCTGACAGCCATAAGACCCAGCAGGGCCCATGCAGTATTGACCACTAATGATTTTTCGCTTTGTACATA
TCTCCTTTCACTGCAAGAGGCAAATTTTTCACCCCATCCTCCATCCTCCATCTGTCTGGAAACAAGGAATGAGCA
GGCTTTTTTTGACCTCAATGCTTGCGGTGTTGTATTTGAAGCTTTTTCCCATGCATGCAAAGGCCTCTAGAGCAA
ACCAAGTACCATACGTAAAGCAAACTCCCCAAGACCCTTCAAAGGAGCCATCCGGTAGCTGATTGGACCTTATGT
ATTCTAGCGAATTCTTCAGAACGGTCCAGATTTCATCGTGTCTGTAGTCGGGATCAGAATCTACGAATTGTCTCA
GAGCTTGTAAGACTGAGGAAGTGCACTCAGTGTAAGTGTAGTCGACCATTATATCACCAAATACTTCTGATGGAT
TCAGTAGTTCTAGTATTTTTCCTCCTCGAAGATTTTCGTAAGATGAAAATCCACCATTTGGGTTCACCATTGTCA
GCAACATGTCCACGGCATCTCTCAAACGTCTCTTAGACACCCTTTTGCTGATGAAGGGACATTTCTCGTTCATCA
TAAGAACGGCTTTCAAGGCCTCTGCAGAAGTATCAGAAACTACCAAACCATGTTCCCTCATAGTCTGAGCCCATC
CGCCCTTATTGTATTGTCGATAGTATTTGACACAATCCGGGGCACTCTCTATAACTTGTGAGATTTCGAGATAGG
AGTAGGTTTTACGTAAAACTTCGTGGAATCTTGGGTCATCTTGGACCCCCACATCGAGCATGGCCATTCCAGCAA
AGGAAACGTCCCAGACCTGGTTTCCGTTCGTACCCTGCATATTTGAGCCGTCGAGTCCCATCCAGACATAATCAT
ACACCCTCTCAACGTGTTTCTTGAATGCCTCAGAGTCGGGTCCGTCCTCAAGGTATCTTATCAACATATTGATCA
TCTTTGAAATCGGACCAATGCTTATAAAGCTGGTCATCAAATCATCTTGTTTAATATGATCAAGACAAGCATCCA
AGGCATTATTTCTGTATCTATTCAATCTGAAAGACTCATACTGATCCAATATACCAAAGGCTATTTTAAACAACC
AACTGTGAGGTGTATAAAGGTCCACTTCCGCAATGTTATTTCTCTGAGACGGCCAGTCGATTTCATCAAATTCCT
CCAAAAATAATTCCTTTCTTAGTTCCAGGATCACTTCATCTTCTTGTGCGTAATACCGTTTACCGTAGAGGAAAG
CCATCCCTATGTAAACCATTCGACAGTGAATCCACAGAGTAGACGGATGGGCTGGAATCCATGATGGCAGTAACC
ACAGTTCAGGAAACAATGTATGCATTCCTTCCCATCTGTAACAGTTAAGAATGCACAGCCAAAATTTTCCCCAGG
ATGGAATGGAAGCAGCACCTCCATGGTAATGAAGGAACTTCCTGGCTCTTTGGAGATCCTCATCTTCAGGACCCA
CACCAAGAAGTCTCATGGTCACGTAATTTAAAGCTGTGCCAAAGACTGTACCCTTATCTTCTGTGTGCCTGATCG
AGAGAAAATCGCACAAGGTACATTGAACCTGTTTCTATTAAAAGCGAAGTAATAATTGCCAAAAATCGGTTCATT
TTG
>1613179
AACATTGGTTTTAAAAATATGATTTATAATATTTATGATATTTCCCTGTAAGAACAGACAGTGTTAAAAATTGAG
TAATGGGTTTAGAATGATTTTGTCACTCTTCGGCGTTGAGTCATTTTTATCAATATAGTGCAGAAGGCATCTTTC
CTCTTACGATTGGGAAAATATTGTTGTTTATTAAGGTGGTAAATGTAACTCCATTAAAAAGAACTATTTGCCTTT

GTTGTAATGTAAGCGAGTAGTAGTAACTATGTTTTTTGCTTTGCTTCTTATTTTCGGTAGGGGTAGAAGGAAGGC
GATCTCTTTCCTTTTCTTCAGCGTCAAATCCACGTTTCATCAATCCATGTTTCATCAAGAAAAAAGTACTGGCCT
CATCATCGATGTCAATTTAATATTTTTCGTGTGCTTACTCCTTTGGTGGAAGCATTGTGCATTCCCGTAAGAAAC
ACTCTTTTCGATTAAGAAGTCAAATTAATAATTATTCAGAAAAGATAGAAACATGAAACTTAAAAACATACTGTA
GGATTTTAATAATTATTCTTAAGTCGAGATCATTCTTCAAACTTGAGTTGTTCGAGATGTGTATGTTAATGTCAA
TGACCATATCAGAATACCCAACGGATTCTCTACCAACAAATCCGCCATGTGCAACAGTAATCTTGTGATCGTGTT
ATATTTCTGATCTTTTGCTCTTGCCGTCAATTGCAATTGTACCATAAAGTTGAGAATAACGTCCCAAAGCCCATA
TAGGAAATATATTTTTAAAAGCTGGATAAGAGATTGCGCAAGACTTGTTGAAAACTCCGGAAATGGTCTCCTGTG
GCCAATCACCATCATCCTGCTGACGATTCAATAGTACCTGGATCCCCTTATCCATGACATTCTTGTCCGGGTATC
CGACAGCCATAAGACCCAGCAGGGCCCATGCAGTATTGACCACTAATGATTTTTCGCTTTGTACATATCTCCTTT
CACTGCAAGAGGCAAATTTTTCACCCCATCCTCCATCCTCCATCTGTCTGGAAACAAGGAATGAGCAGGCTTTTT
TTGACCTCAATGCTTGCGGTGTTGTATTTGAAGCTTTTTCCCATGCATGCAAAGGCCTCTAGAGCAAACCAAGTA
CCATACGTAAAGCAAACTCCCCAAGACCCTTCAAAGGAGCCATCCGGTAGCTGATTGGACCTTATGTATTCTAGC
GAATTCTTCAGAACGGTCCAGATTTCATCGTGTCTGTAGTCGGGATCAGAATCTACGAATTGTCTCAGAGCTTGT
AAGACTGAGGAAGTGCACTCAGTGTAAGTGTAGTCGACCATTATATCACCAAATACTTCTGATGGATTCAGTAGT
TCTAGTATTTTTCCTCCTCGAAGATTTTCGTAAGATGAAAATCCACCATTTGGGTTCACCATTGTCAGCAACATG
TCCACGGCATCTCTCAAACGTCTCTTAGACACCCTTTTGCTGATGAAGGGACATTTCTCGTTCATCATAAGAACG
GCTTTCAAGGCCTCTGCAGAAGTATCAGAAACTACCAAACCATGTTCCCTCATAGTCTGAGCCCATCCGCCCTTA
TTGTATTGTCGATAGTATTTGACACAATCCGGGGCACTCTCTATAACTTGTGAGATTTCGAGATAGGAGTAGGTT
TTACGTAAAACTTCGTGGAATCTTGGGTCATCTTGGACCCCCACATCGAGCATGGCCATTCCAGCAAAGGAAACG
TCCCAGACCTGGTTTCCGTTCGTACCCTGCATATTTGAGCCGTCGAGTCCCATCCAGACATAATCATACACCCTC
TCAACGTGTTTCTTGAATGCCTCAGAGTCGGGTCCGTCCTCAAGGTATCTTATCAACATATTGATCATCTTTGAA
ATCGGACCAATGCTTATAAAGCTGGTCATCAAATCATCTTGTTTAATATGATCAAGACAAGCATCCAAGGCATTA
TTTCTGTATCTATTCAATCTGAAAGACTCATACTGATCCAATATACCAAAGGCTATTTTAAACAACCAACTGTGA
GGTGTATAAAGGTCCACTTCCGCAATGTTATTTCTCTGAGACGGCCAGTCGATTTCATCAAATTCCTCCAAAAAT
AATTCCTTTCTTAGTTCCAGGATCACTTCATCTTCTTGTGCGTAATACCGTTTACCGTAGAGGAAAGCCATCCCT
ATGTAAACCATTCGACAGTGAATCCACAGAGTAGACGGATGGGCTGGAATCCATGATGGCAGTAACCACAGTTCA
GGAAACAATGTATGCATTCCTTCCCATCTGTAACAGTTAAGAATGCACAGCCAAAATTTTCCCCAGGATGGAATG
GAAGCAGCACCTCCATGGTAATGAAGGAACTTCCTGGCTCTTTGGAGATCCTCATCTTCAGGACCCACACCAAGA
AGTCTCATGGTCACGTAATTTAAAGCTGTGCCAAAGACTGTACCCTTATCTTCTGTGTGCCCTGAAGAACTGACT
TCTGATGATAAGAAAGATAGAAGTGCCACAATTTCACCATCAAGGATGGGTTCCTCTTTCATGTTTGATCCAAGC
AAGGTACGTTTTACTCTATTTTTTCTCTCATCATTGTACGTAATTCAACAAGCAGAAAGCCCAAGGGCATTGTGG
TATCGATGATTCAATTAGAAGTATTTGCTGAATATTGGTGAGGATGTGCAACATGCATGGTTATTAAAATCTTTC
AGTAATTTTCTGCATAAAAATTGTTACAACTATATATATATACACAACATCCGTTACCTTAAGATAATGACTGAT
AAGTAGCTGTGGGTCTATGTAACTCAGCATGCACACCTACATATGTAACAAATGATAGATATGTCCAAGGATCTT
GTTAATGGAATTCCATGTACACATGGTGTTAAAAGATTGACCTTTACTTAGGCCAAATGCCCTGAGCCAGCAAAT
ACAGT

Supplementary Notes 2: OSC sequences and alignments used in Extended Data Figs. 8b and 9a-b.

```
>S-cinnamoneus
MNAGDPVPDVSAAYEAACGRLLELQSDDGSWEGEMEWNTTILSQYVIVTRVLGRPPDETSRQGMLTYFRNTRTDQGGWGMHPAGPPSPYATLLA
YVALRLLGVGPEESPAADARKWLGTQPGGARSVPQWGMFWLAVLGLVPYREVAPFFPPEAMLLPRWVPLHPERMLGWTRMLYQAMSYLYGARFRA
DLGSLADELGRELFPATPRPAGPGSDVVLPRSRLLRALQLGLRGWERMHSRRLRRAALDRCHRAVVDEQHASPVHGLSSVNALAECLVLFAHDR
RHPLLDAAVARLEYWVWSDDKQGLRLCGDRTAVWETSFAVSALLASGAEHRSLQTGPALLRASAYLRDAQITTYPGLVPPVRTVVGGWALSDRD
SRWPVGDCTAEAVNALLAAEDAQHLRTWSLPAALDIILDRQNRDGGFGTLDRQRAGRWLEALNPTEMFANCMVDSSSVDCTGSALTALARIRPL
LSPDGRRRADAAIKRGAAFLRSAQNPDGSFTSSWGINYTYAAFHAARGLRAAGASPGDPSLRALGQWLVATQLADGGWGEDWRSCPERRYIALG
HSLPGMTSWAVLAGLDALGTAHPSVADGVRWLCSAQRPDGSWSEDQVNGVFFTTMMINYRMYPAYFPTLALGRYLQAVTSLTPDSGTPQGAA
>G-sp.SH-PL17
MIWCPVVTAQVAITRHVVGLPFSAEDTAKILTHFERTQTSDGAFGLHLEHSGSVFVTSLVYVAMRCLGVSADHPLAVRARNWLHAQPGGILSAP
TWGKFWLTLLGLYGRDGLRPLVPELALLPRAFPVHPIRFYCHTRYVYLAMSLLQGSHARFALGSLGDELRHELYGSAGPPASFREHRYHLAPSD
AFEPPNLLIRAAERVMGWYDRFPIRRLRESALQRCAHLIQLDLDVSDRLTLSPVNGVLNVLALHARGADRTLIAKCVEGFEAYRWDDAARGLRY
SGGRTRVWDTGFAVESLLADPGAVRANRDALLRAYRFLAEQQVMKPVAGRDPMFPDRALGGWCLGDSAHSWPVSDCTAEALSAVLGMHAAPELE
LDSRERISDTRLALAADFILSRQNPDGGFGSYERARSPRWLERLNPSEMFTRCMTDQSYIECTGSCLVALSRFRVAVPHHATNRIDRAIRRGAR
FLLGRQHSGGAFPGAWGVYFTYGTFHAVRGLRAAGYGSNPALQRAADWLIRHQKPDGGWGEHYTSCLRQECVEHPESQATMTSWAVLALCEVVG
AKHTAVCRGVEWLAAHRSGAGRHPREAVNGVFFGTAMLDYDLYREYFPVWALAAAL
>Coffea
AISFYSTLQTHDGHWAGDYGGPMFLMPGLIITLSITGALNAVLSKEHKLEMCRYIYNHQNPDGGWGLHIEGPSTMFGSALNYVTLRLLGEGPND
GDGAMEKGRKWILDHGGATAITSWGKMWLSVLGAYEWSGNNPLPPEIWLLPYALPVHPGRMWCHCRMVYLPMSYLYGKRFVGPITPTVLSLRKE
IYAVPYHEIDWNLARNQCAKEDLYYPHPLVQDILWASLHKVLEPILMHWPGKKLREKAVSVAMEHVHYEDENTRYLCIGPVNKVLNMLCCWVED
PNSEAFKLHLPRIYDYLWIAEDGMKMQGYNGSQLWDTAFAVQAIISTNLAEEYGPTLRKAYTFIKNSQVLDDCPGDLDSWYRHISKGAWPFSTA
DHGWPISDCTAEGLKAYFSLSKLPSELVGETIDVKRLYDSVNVILSLQNSDGGFATYELTRSYAWLETINPAETFGDIVIDYPYVECTSAAIQA
LTAFKKLYPGHRREEVQRCIERAALFIEKIQATDGSWYGSWGVCFTYGLWFGVKGLVASGRNFNNCSAIRKACDFLLSKQLPSGGWGESYLSCQ
NKVYSNLEGNRSHMVNTAWAMLALIDAGQTERDPTPLHIAAKVLINAQFENGDFPQEEIMGVFNKNCMITYAAYRNIFPIWALGEY
>Glycine
AVSFHSTLQCHDGHWPGDYGGPMFLMPGLVITLSITGALNTVLTEEHRKEICRYLYNHQNKDGGWGLHIEGPSTMFGSVLSYITLRLLGEGPND
GQGEMEKARDWILGHGGATYITSWGKMWLSVLGVYEWSGNNPLPPEIWLLPYMLPFHPGRMWCHCRMVYLPMSYLYGKRFVGPISPTVLSLRKE
LYTVPYHDIDWDQARNLCAKEDLYYPHPLVQDILWASLHKFLEPILMHWPGKRLREKAIISALEHIHYEDENTRYICIGPVNKVLNMLCCWVED
PNSEAFKLHLPRIYDYLWIAEDGMKMQGYNGSQSWDTSFAIQAIISTNLGEDYGPTLRKAHTYMKDTQVLEDCPGNLDFWYRHISKGAWPFSTAD
HGWPVSDCTAEGLKAALLLSNMPFDIVGEAISPVHLYDAVNWILSLQNCTGGFASYELTRSYAWLELLNPAETFGDIVIDYQYVE
DHGWPISDCTAEGLKAVLLLSKIAPEIVGEPIDVKRLYDSVNVVLSLQNEDGGFATYELKRSYNWLEIINPAETFGDIVIDYPYVECTSAAIQA
LASFRKLYPGHRREEIQRCIDKATTFIEKIQASDGSWYGSWGVCFTYGAWFGVKGLIAAGRSFSNCSSIRKACEFLLSKQLPSGGWGESYLSCQ
NKVYSNLEGNRSHVVNTGWAMLALIDAGQAKRDSQPLHRAAAYLINSQLEDGDFPQQEIMGVFNKNCMITYAAYRNIFPIWALGEY
>Nicotiana
ALSFYSTLQAHDGHWAGDYGGPMFLMPGLVIALSVTGALSAVLSEEHKREICRYLYNHQNSDGGWGLHVESPSTMFGSVLSYVTLRLLGEGTNG
GEGAMEKGRKWILDHGSATAITSWGKMWLSVLGIFEWSGNNPLPPEIWLLPYILPFHPGRMWCHCRMVYLPMSYLYGKRFVGPITPTVSSVRNE
LFTVPYHEINWNKARNECAKEDLYYPHPLVQDILWASLDKLVEPIFMHWPGKKLEKALRTVMDHIHYEDENTRYICIGPVNKVLNMLCCWAEDP
NSEAFKLHIPRIYDYLWIAEDGMKMQGYNGSQLWDTAFAVQAIIASNLIEEFGPTIRKAHAYIKNSQVLEDCPGDLNKWYRHISKGAWPFSTAD
HGWPISDCTAEGLKVCLQLSKLPMEIVGEPLEAKRLYDAVNVMLSLLNPDGGIATYELSRSYPWLEIVNPAETFGDIVIDYPYVECTSAVIQAL
AAFKKLYPGYRKEEVDHCIRKGASYIEKIQAADGSWYGSWGVCFTYGTWFGVKGLLAAWRSFNNSSSIRKACDFLLSKQVLSGGWGESYLSCQN
KVYTNLEGNRSHIVNTAWAMLALIEAGQGKRDPAPLHRAAKVLINSQLENGDFPQQEIIGVFNKNCMISYSAYRNSFPIWALGEY
>Panax
ALKFYSTIQADDGHWPGDYGGPLFLLPGLVIGLYVMGVMDTILAKEHQREMCRYIYNHQNVDGGWGLHIEGCSTMLCTALNYITLRLLIRGDEE
EEIRDEAANGGSLEKARRWIIDHGGATYIPSWGKFWLSILGVYEWSGNNPLPPEMWLLPYFLPLHPGRMWNHCRMVYLPMSYLYGRRFVGPINS
TVLSLRRELYTHPYHQINWDLARNQCAQEDLYYPHPLIQDMLWSCLHKGVERLIMQWPLSKIRQRALTTAMQHIHYEDENTSYICLGPVNKVLN
MVCCWVEDPNSMANILHLSRIKDYLWVAEDGMKMKGYNGSQLWDVGFAVQAILSTGLVDEYGSMLKKAHDFIKISQVREDSPGNLSSWNRHISK
GGWPFSTPDNGWPVSDCTAEGLKAALLLSNMPFDIVGEAISPVHLYDAVNWILSLQNCTGGFASYELTRSYAWLELLNPAETFGDIVIDYQYVE
CTSAAIQGLKSFMRLYPGYRRKEIEACIAKATNFIESIQLPDGSWYGSWGICYTYGTWFGIKGLVAAGRTNRNCYSIRRACDFLLSKQLGSGGW
GESYLSCQNKVYTSIEGNISHVANTGWAMLALIEAGQAQRDPSPLHRAAKVLMNSQMKNGVFPQQEIVGVFNKNCMISYSAYRNIFPIWALGEY
>Cucumis
ALSFYSAVQTSDGNWASDLGGPMFLLPGLVIALYVTGVLNSVLSKHHRQEMCRYIYNHQNEDGGWGLHIEGSSTMFGSALNYVALRLLGEAADG
GEHGAMTKARSWILERGGATAITSWGKLWLSVLGVYEWSGNNPLPPEFWLLPYSLPFHPGRMWCHCRMVYLPMSYLYGKRFVGPITPIVLSLRK
ELYTIPYHEIDWNRSRNTCAKEDLYYPHPKMQDILWGSIYHVYEPLFSGWPGKRLREKAMKIAMEHIHYEDENSRYICLGPVNKVLNMLCCWVE
DPYSDAFKFHLQRIPDYLWLAEDGMRMQGYNGSQLWDTAFSIQAIISTKLIDTFGPTLRKAHHFVKHSQIQEDCPGDPNVWFRHIHKGAWPFST
RDHGWLISDCTAEGLKASLMLSKLPSKIVGEPLEKNRLRCDAVNVLLSLQNENGGFASYELTRSYPWLELINPAETFGDIVIDYSYVECTSATME
ALALFKKLHPGHRTKEIDAAIAKAANFLENMQKTDGSWYGCWGVCFTYAGWFGIKGLVAAGRTYNNCVAIRKACNFLLSKELPGGGWGESYLSC
QNKVYTNLEGNKPHLVNTAWVMMALIEAGQGERDPAPLHRAARLLINSQLESGDFPQQEIMGVFNKNCMITYAAYRNIFPIWALGEY
>Volvox
LNGAISFYECLQQDDGHWPGDYGGPMFLLPGLVIGLYTTGALDQVFTPHHKQEALRYLANHQNDDGGFGLHIEGGSTMFGTGLNYVMARLLGMG
PDEDLTRRAREWVNSRPRGGYLHHQLGQVLAGSARGVPWDGMNPLTPEMWLLAVQQMDGHRLPAPGPLLVYLPMSYVYGMRGTCKETALTAAIS
QPLPPRSHLRNSPPGSHHIRTWPTHMPYRQELYPMPYSKIDWNAARNQCAKEDLYYPHPLVQDVLWWALYRAENVLQGSFLRRMALKECMKHIH
YEDENTRYIDIGPVNKVVNMLCCWLEDPNGLPYKKHLVRVADYLWVAEDGLKMQGYNGSQLWDTSFAVQALAEAGLLDVTAASLARAHAYVEQS
QVVEEAAPPLDRYYRHISKGAWPFSTRDHGWPISDCSSEGLKAALALAGLPADKVGEPIPAERLYDCVNVILSYQNSDGGMATYENTRSFHWLE
ILNPAETFGDIIVDYSYVECTSACITALAAFRKRHPDHRPSEISAALGRAEAFIRSIQRADGSWYGSWGVCFTYACWFGITGLVALGHNYHNDP
AVRRCCEFLAVRQREDGGWGESYLSCQDKVYSQLDGDSHVVNTSWAMLALLAAGYHRVDPAPFHRAARFLLRMQLPSGDWPQQHISGVFNRNCM
ITYANYRNIFPIWALGHY
>Chlorella
NGVSFYEGLQAEDGHWPGDYGGPMFLMPGMVIALYTTGTLDSVLSPQHKAEMVRYLRNHQNADGGYGLHIEGTSTMFGTVLSYVTLRLLGVGPD
DATLAPARTWIHERGGAHAITSWGKFWLAVLGVYSWEGLNPLSPEMWLLPYASWTGIGWLHPGRFWCHCRMVYLPMSYVYGVRGTCRATPLTEA
LRQELYPLPYAKIDWNQARNLCAKEDLYYPHPLIQDVLWWALYKAEPLLLGSRLRGAALAECMKHIHYEDENTRYVDIGPVNKVINMLACWFED
PGSQAFKRHLPRLLDYLWVAEDGMKMQGYNGSQLWDTAFAVQAIASTGMAGEFSRCLKRAHEYLEQSQVVEEAQQPLSEYYRHISKGAWPFSTR
DHGWPISDCSSEGLKAALVLAQMDPKLVGPPIPEPRLCDCVNVVLSYQNGDGGWATYENKRSFEMLEIINPSETFGEIVVDYNHVECSSACITA
```

LTAFAGRYPAHRAHEIGAALRRGIKYLKSIQRPDGSWYGNWGVCFTYGTWFGCEALAAVGETHGSSASARAACAFLLQKQRSDGGWGESYLSCQ
DKAYSQLEGEQPHAVNTAWAMLALLAAGYEQVDRKPLDAAARCLIRLQEESGDWPQQHISGVFNRNCMITYANYRNIFPIWALGVY
>Micromonas
GVEFYQGLQDEDGHWASDYGGPLFLLPGLIIALYVMGQLDHVLPDYVQVEMRRYLLNHQNEDGGFGLHIEGSSTMFGTTLSYVSMRLLGMTATT
EAVVNARSWILSRGGAINVPSWGKFYLCILGIYDWEGLNPVPPECWLLPYEYNPIHPGRFWCHCRMVYLPMSYLYGCRASGASSFLTEELKSEL
FVGNYKTINWDKTRNTCASEDLYYSHPRIQDALWWGLTKVEPFFLRWWPGRWIRANALKLTMNHIHYEDENTRYINIGPVNKVMNMLSCWFQDP
SEFGSWRKHTPRVADYLWLAEDGMKMAGYNGSQLWDCAFAVQAIVATGLHVEYSACLRSAHKYIRDSQVLDDCPGQLSRRFRHISKGAWPFSTR
DHGWPISDCSSEGLKAALELEAMGSERAGPSVPVGLLQECVNVILSYQNMGGGWATYENTRSYEWVEIINPAETFGDIMIDYPYVECSSASMQA
LAKFHQRYPTYRKQDIKKSLHRGRKFLLSIQRRDGSWYGSWAICFTYGTWFGIKGLMSTGSTFETCEALRRAVKFLLSKQMPCGGWGESYLSSQ
TKQYVQLEGGVSHVVNTAWAMLALLASGQISRDPLPLHRGARSLMRAQCSNGDWPQQTIMGVFNNNCMITYANYRNIFPLWALGEY
>Chondrus
KGLSFFATLQTGDGHWAGDYAGPMFLLPGLVIACYVSRTPLPPAHRAEMLRYLRNHQNPDGGFGLHIEHKSTMFSTALNYVAMRILGAGSADSD
SRSARAWIRAHGGPAGCPGWGKFWLAVLGVYEWQGMDPLTPEFWLLPYALPCHPARFWCHCRVVYLPMSYLYGRRAVGEVTELVRELREELYEE
PYESIEWPAWRGRCCEEDVYVRRPKLQRVLWGALALWESIWFPGKQWLRERALKETLMQITAEDENTNYICIGPVNKVINFLCRWFDDPDGVAV
DKHRDRLTDYLWLAEDGMKMQGYNGSQLWDTAFASQAFMAAGRDIVEPFTKTLSLAHDYVEMTQVLVDVPNRERFYRHMSKGAWPFSTRDHGWP
IADCTGEGLAAALLLQGKKGTWIPKKKMISNDRLQDAVKMILSYQNPDGGWATYELQRGPAWLEFLNPSEVFGDIMVDYSYVECTASALKGIGE
FRAEFPDHPLVPKLNESLAKGIRYIESIQKQDGSWYGSWGICFLYAIWFAIDAYTSMGLTLETSPSMERACQFVLNKQREDGGWGESYLSSETM
VYTQSEESLVVSTGWALVALSMARWPDREPLEKASQFLIRSQDENGDWPQQNICGVFNRNCMISYSQYRNIFPIWALAEY
>Galdieria
TAIQYYRRLQMSDGHWPGDYGGPMFLLPGLVIVCYITETDLGNETKKEMKRYLYNHQNEDGGWGLHIEAPSSMFGTAMNYVALRILGVDRDDSA
AIAARNWILQRGGALGIPSWGKLYLAVLGLYHWDGLNPLTPEMWILPYWVPIHPGRFWCHCRIVYLPMSYLYGRRATAKETSLIRELKEELYLD
NFDQIDWNAQRENCCKEDIYTHRPKVQSWLWTILSWYEKFPIPGKSYLRNLALEETLLQVKKEDEYTDFICIGPVNKVLNMLCCYFDDPYSEHF
KKHIPRLKDYLWLAEDGMKMQGYNGSQLWDTAFSAQALCEAGSITRHHFPSTLQLAHHYLDIAQVRENVPQGERYYRHISKGAWPFSTRDHGWP
ISDCTAEGLKAVLALEACGSIPKEQFFSHERLFDAVDVSLSLQNKDGGWATYENTRSYSWLEWINPSEVFGDIMIDYSCVECTSSSIQGLAAFR
ARHPGHRRAQVDIAIERGARYIESIQRPDGSWYGSWGVCFTYGTWFGVEGLVAAGRTFESCESLPKACRFLCSKQKSDGSWGESYRSCTDKVWM
EAEQGQVVHTAWAVIALIKAICTSHSMWHSDVHVSHQAYHSSIRKGIDFLLESQLENGDWPQQRISGVFNRNCMISYSNYRNIFPLWAIALY
>Leishmania
DGIRFLLKLQDPFSGHWPNGYSGCLFLCAGFVITKYIVAGGETRRMFPPFSDHHHVKLASSSSDTKQRSSCGDYPERVLGVPAAENAGEERCQC
GEAMRQELIRYIRNHQNLDGGWGQHTEGHSTMMGTVLNYVSLRLLGVPASDPQATCGRSWILAHGGATTTPMWGRVWLSILGVYSWDGVNPISP
EMLLMPDWVPFSLGKMWCHSRVIAIPFSYFYGLRWSAPAFPTTLALRKELYTEPYATIPWRSFRGVVCELDVYSPTSPLFRVAMGLLDLYERHP
IPFLRRYALEVSWRHMAYDDENTHFICLGPVNKWLNMLATWLREGEHSARFQEHFDRVSDYFYMGETGLSMSGYNGSQLWDTSFAVQAICACRR
EMMFPAEVELAHHYIDVAQVQEDPMAAPYFYRHRTKGAWNFSTRSQGWQVSDCTAEGLRALLLLPQYAFPMRRIFDGVDEVLSLRNSGLGGDGG
WASYEPSRAPAYCELLDCSELFKDVMIDYSYVECSSSCIHTLSLFRERYPHYRRRDVDRAISEGIAYVLGQQQPDGGFYGSWAVCYTYAAWLVA
DALQASKELPEMASHPHCRRLIDFLLSHQAADGGWSEDISASARQTWVDSPDGSQVVNTAWAVMAIISAAGKAASTEPTRQRCIRRAVDRGVQL
IMSRQLASGDWRQERISGVFNGNNPIHYPGYKNSMPVWALGKY
>Leptomonas
DGIRFLLKLQDPFSGHWPNDYSGCMFLVAGLIYTKYIIADGEAHRMFPPCPEHHHVKLSGKRRKAQNGNASMGDYAERVLGVPAAENGGEEPCR
CGEATRQELIRYLRNYQNQDGGWGQHTEGHSTMLGTVLNYVSMRLLGVPAKDPQAASARDWIHAHGGATTTPMWGRVWLSILGVYSWEGVNPIP
PEMILLPDWVPFSLGKAWCHSRVIAIPFSYLYGLRWSAPAFPTTLALREELYTQQYSAIPWRSFRGAVCASDLYTPTSSLFRMAMSLLDLYERH
PLPFLRRYALEVNWRHMAYDDENTHFICLGPVNKSLNMLVTWIREGEHSARFQEHCDRVADYLYMGPTGMRMSGYNGSQLWDTSFAVQAICACG
REMMYPMEMALAHHYVDVAQVQEDPMAAAAFYRHRTKGAWNFSTSAQGWQVSDCTAEGLRVMLLLPQYDFPVRRICDGVDEILSLRNSGCGGDG
GWATYEPTRGPSYCELLDCAELFKDVMIDYSYAECSSSCIHTLSLFREQYPHYRRSEVDRAISEGIACVLGKQQPDGGFYGSWAVCFTYGALDR
RGCAACV
>Phytomonas
RLELIRYIRNYQNDDGGWGQHTEGHSTMLGTVLNYIALRMMGVAAADLSMASARRWIHAEGGAVYTPLWGRIWLCILGLFSYDGVNPLSPELVL
LPRWISFSIGRSWCHSRVIAMSFLYFYGLRWSASRHPLLEALKEELFVQPFHTICWRRYRYEVCPKDLYTPVSRTYKVFCKVLELYERRPISFL
RRYALEKNWQHIAYDDENTHFICLGPVNKSLNMLVTWIREGENSVRYQLHRQRVDDYFFMSPYGMHMSGYNGSQLWDTSFAVQATCACKSEMLF
ANEMALAHHYIDIAQVRDDPIQKDYFYRHRTKGAWNFSTKDQSWQVSDCTAEGLRAVLLLRHMPFPTARIFDAVDEILSLRNSSGDGGWASYEP
SRAPHYCELLNCSEMFKDVMVEYSYAECSSSSIHTLSLFREQFPYYRRSSIDTAISEGIAYIFSTQNPDGSFYGSWGVCFTYAAWLVSDALRIS
KEFSNIADHPRCRLLADFLMSHQNNDGGWGEDINACVRQEWVDNPDGSQVVNTAWAVMAIMNVSGDPNHSSYARWHQIASAVERGIQLIMSRQL
ATGDWAQERLSGVFNGNNAIHYPGYKNTMTVWALGMY
>Trypanosoma
DGVEFLLRLQDPYSGHWPNDYSGPLFLTPGFIFTKFIVAGGDIRKMFPPHRDHQHKNDEPCRCGEAERVEMIRYLRNYMNKDGGFGQHTEGHST
MLGTALNYVALRFMGVPADDADATRARAWIRSHGGAVSVPTWGKVWLCIVGLYSWDGINPIPPELSLLPAWFPLSQGRLWCHSRVISVPFSYLY
GIRWAPEPHPLLEALRQELYTEPYDQIQWDQHQSNICNLDCYTPISSTYKLVAVLLKLYEKWHIKSLRRHALEVAWSHVAYDDEDTKFICLGPV
NKALDMLLTWIREGENSGRYQNHVSRLADYIYMGPEGMRVCGYNGSQLWDTSFAVQAVCACNMELLYPQQMSLAHHYVDVAQVQKDPKSAAHFY
RHRTKGAWNFSTASQSWQVSDCTAEGLRVVLLLRHNPFSVSRIRDAVDEILSLRNAKGGWASYEPTRAPLYVELFNSSDVFRDVMTDYGYAECS
SSCIHTLALFREHYPGYRRAEINAAIREGLKFVLSLQRPDGSFYGSWGVCFTYAAWIVASALCISREIPDMANHPSCVRLIDFLLSHQNADGGW
GEDVTASVRSLWVDNPSGSQVVNTAWAVMAIMSAAGEAARSLPRWREQISVAVERGIRLIMSRQLVTGDWAQERISGVFNGNNPIHYPGYKNTM
PVWALGMY
>Bodo
SGAAFLMKLQHPTSGHWPNDYSGPMFLLPGAIFVKFIIARGDASKMFKFPQQRTEFIRYLRNMQNPDGGWGMHTESHSTMFGSVLNFVSLRLLG
VDANDSAAKAGSRWILERGGALSIPSWGKVWLCVLGLYEYDGINSIPPELSILPDWLPFSQGMYWCHARIVTTPFSWFYGKRWKADSHPLLEAI
KTEIYVQPYAKISWAKHRSDVFAPDIYTPHSWVYTVANKILLAYEKVHSKKLREIALKRALEHIRYDDESTDYICLGPVNKVLDMLITWLVDGE
DSAAFKRHVERLEDYFYIGYDGMRMSGYNGSQLWDTSFAVQAAVACGVEQQFEREMAKAYHYVDVAQVREDPPAAHAFYRARTKGAWNFSTRAQ
SWQVSDCTAEGLRVALLLRNKLSVLRHTDTMDDSRLFDGVDEILTLRWAAGDGGWGSYEAPRGPRYLELLNCSEIYKDIMVDYTYSECTSSCVH
TLCLFRKQFPQYRIEEVNRAIDEGSRCVVSKQGPDGSFYGSWAVCFTYGAWIVTDALHMAGFDETSPVFKKACAFLLGKQRADGGWGEDFNSCV
RQVWVENPDGSQVVNTAWAVMALMAAGGATHRQEVERGIRFIMSRQLANGDWPQERISGVFNGNAAIHYPGYKNSMPVWALGKY
>Aphanomyces
QAVDYYKLLQSEDGSWHGDYGGPMFLLPGLVITSYITGHDLGKSVRDGMIVYLRNHQQYDGGWGIHIEEGSTMFGTVLNYVALRLLGAAADDEA
CLEARTFIKHHGGATLVPSWGKFWLAVLNVYDWRGVDALPPEMWLLPRWLPFHPGRTWVHCRMVYLPMSYLYGIRFQAKETPLIQAIRDEIYTT
PYQSVSWRNARGAYSKMDEYHTPSPIIRTLNYLLSWYELLPTVTSLRKHGLEYTLAFVRADDEESNYCNIGPVNKMINMLVQWVVDPDSDEFKR
HAQRVEDYIWVAEDGVKMQGYVGSQTWDSSFAVQAFVDAGVAADPAFQTTFKLAYRFLTEAQNVHDPKDAATWWRRVQKGGWGFGIASNGYPVS
DCTAEAVKALLLMEERANLPAFPDDRLRDAVDFILALQNSDGGFPPYERSRGFDWYEHLNPAVVFGAIMHDYSYVECSSSSLSALQAFHARHPT
YRPEAIKRATTKADTFIRSLQYQDGSFFGKWGVCYTYGTMFAIKGMRAAGASDKDEDVQDAVSFLVNKQRNDGGWSESFFACSTRRYLETPSSL
VVNTAWALIGIMKGIEGGDPQMHVKEWDAVKKGIEFLVAKQLPSGDWAQERISGVFNRTCGITYANYRNIFPIWAIGLY
>Myxococcus

QEADGSWKGDYSGPLFLSPLYLIGLYAMDRAPDPGHRDGLLANIRAHQNADGGWGLSPGAPSQVFTSVLNYVAQRLLGVDAKDADLARARAWFL
PRGGPLGSGSWGKAVLALLGLYEYDGLTPTPPELWLLPKALPFHPSRMWCHCRMVYLPMGWLYGRKARVRQTPLLAELRRELYPEPYESVDWKA
ARQRVADTDAYTPRSVWLRAASRVLGLYERLHSKRLRARAMAESLAQIRGEDEATNFLCIAFINKMLDTVVWHLEKPDGPEVRAHLAKLPTYLQ
PTPEGLALNSYNSSQLWDTTFAIQALVASGADHAREALARAGRFVEAQQVREDSPHPERFYRHPSRGAWPFSTREHGWPISDCTGEAVKACLLL
EPLGLNRVPRERLAQAVDFILSLQNRDGGWATYEPTRAPPWLERLNASDVFANIMVDISYVECTSSCVQALVAWKKVQPEAPVEDAITRGLDYL
RRTQRGDGSWEGSWGVCFSYGTWFAVSGLVAGGATRADPALRRAVRFLEEHQREDGAWSETLQSCWERRWVEGVTGHAVTTSWALLALFACGEA
DSKSTRRGVAWLRARQGADGRWPREPLAGIYNRTGGIHYDTYLRTFPLWALS
>Corallococcus
RARDMLAGTQAADGSWKGDYSGPLFLGPVYVAGLYVMGRTPEASVRDGMVAHMRAHQNADGGWGLDVESPSLVFTSVLNYVAQRLLGVGADDPG
LVRARAWFLPRGGPLSSASWGKFLLALLGLYEYEGLAPVPPELWLLPRGLPFHPSRLWCHCRMVYLPMGWLYGRRARAPETPLLAELRRELYPQ
PYADVDWKAARGRVARTDAYSPHGLGLRAVHRVLGWYERFHSKRLRERALEESLELIRGEDEATHFVCIGPINKVLDMVVWHVARPDGPEVRAH
LERLPDYLQHTHEGVAVNGYNSSQLWDTAFAVQALVAAGESAWARDTLERAGRFLEAQQVLEDSPDAARHHRHPSRGGWPFSTRAHGWPISDCT
AEALKACLLLEPLGLNRVPRERLEQAVAFILSLQNRDGGWATYEQQRGPRWLERFNPSDVFAGIMVDPSYVECTSACIQALAAWRGAWPHAPVG
QSIARGADFLRRQQRPDGSWEGAWGVCFSYGTWFGVTGLVASGAGTGDPALRKAVTFLKAHQREDGAWSETIQACRERRWVEGRTGHAVMTSWS
VLSLVAAGEANAEATRRGVAWLRERQEAEGQWPREPLAGVFSRTCAIHYDAYLRIFPLWALS
>Stigmatella
MGRTPEPEQRDGLIAYLRNHQNADGGWGLDVEAPSQVFTSVLNYVALRLLGVGKDDAGLRRARQWFLPRGGPLGSGAWGKIILALLGLYEYGGL
QPVPPELWLLPESLPFHPSRLWCHCRMVYLPMSWLYGRRARAPETPLLAAIRQEIFDGGYGQVDWVAARERVSPTDVFTPRTFWLKAANQVMYG
YERLAGKQLRARALDFALEQIRAEDEATHYICIGPINKVLNMVVWHFVNPDGPEVRAHLERLPDYFYEGDDGVRMNGYNSSELWDTAFAVQAVA
ATGETGRHRRMLEEAARFIEANQVLEDTREPQRFFRHPSKGGWPFSTRDHGWPISDCTAEGLKASLVLEPLGLNRVPQARLQDAVQLILSMQNE
DGGWATYELQRGPKVLELLNPSDVFSTIMVDVSYVECTSACVQALAAWRKHHPVPDARVDRAISRGVEFIRRTQREDGSWMGSWGVCFTYGTWF
GVMGLIAAGASPDDMALRRATAFLRSYQRADGAWSEVVESCRQARWVEGKQGHAVNTSWALLTLAAAGEGGSDAAQRGVRWLRERQQEDGRWPP
EPIAGIFNRTCAIHYDAYLRIFPVWALA
>Cystobacter
AMTHLSGLLSPEGSLKGDYGGPLFMLPMYVGTAHAVGLELDAATREGMVRYLKSVQNKDGGFGLHVEASSYVFTSTLCYVALRLLGVSAEDPAA
TSARQWILAHGGALTSAPWGKFFLSVLRLHEYEGLDPLLPELWLLPEALPMHPSRFWCHCRMVYLPMSWLYGKKARIPDSPLLQQLRRELYPTP
YEQIDWPAHRTRVSPTDSQVPRSALVLAANHLMGLYESQASTRLRERALSFVLDHIRQEDENTRYICIGPVNKLLHVLVWHFERPGGAELRAHL
AQLPDYLWKGPDGVKMNGYNSSELWDTAFAAQAVVASGRIQENLPFLRSAFDFIDRSQVREDTPNAERYYRHRSKGGWPFSTRDHGWPISDCTA
EGLKAALALEPFVDSPLSQERLTDAVDLLLSMQNEDGGWATYELTRGPKWLELLNPSDCFSDIMIDPSYVECTSSCMQALARFRERLPGVRAKE
IDTAMKRGARYVERAQRPDGSWEGSWGICFTYGAWFGVWGLVAAGYSPSHPALQRACDFLISKQNADGSWGETPESCRQRRYVQAEQGQAVMTS
WAVLALSKAGRRDSPEVQRALGFLVQRQRPDGSYPEEHIAGMFNKTSGIHYDHYLDVFPLWALS
>Labilithrix
QGEDGAWAGAYGGPMFLLPMYLALCHAAKRPPSETQRARIIAYFERAQNEDGSVGVHAEDTHGSLFCSVLAYVALRMLEVPKDDARVRRMLAFI
HRHGSPLAAAQWAKVTLSLVGLYRWDGLTPLLPELWLLPYAATPFHPARLWCHCRQVYLPMSWLYGRRARIDDDELIRALRDELYDGAWSSIDW
SAHRDTVAQVDAYRPPSRLLALANAAQRAVERITPDRVRAMALGKVREHIAYEDEVTNFIDIGPVNKVLNAFVAFFDDPKSEAFERAFATCESY
LFDNELGTMMQGYNSSKLWDVAFATQAIVATPLGDEHRTMLERAYGYVRDNQILDDVPRRKRFYRDASRGGWPFSNRAHGWPITDCTAEGLKCA
LALEGRFAPRIPEPLLRDAVMLILDWQNDDGGWATYEKRRGGAWLEKLNPSQVFGDIMVDYSYVECTSACMQALRASLPRFGRRLAARARRAIA
KGERFLRNAQRPDGSFEGSWGVCFTYGTWFGVTGLLAAGVPTSDRAIRSACDFLLAYQRSDGGWGEEGDSCRERRYIPSDTAGVVQTSWALSTL
VRAQHPNRQAKARAAKLLVDRQESDGSWAREPLVGVFNKTCLIDYDNYRHYFPLWALSEF
>S-caatingaensis
QRQDGSWEGEMESNTTGTAQYVIVLRALGRPLDETTRRGIVQHFRRTRRPGGGWALHPQGPPSPYATTLAYLALRLLGTAPADPLAAEAAAWLR
SLPGAPALPPWGRFWLAVLGLIPYEETDPLPPEALLLPAWTPLHPSRLYGRTRLLHQAMALVHGTRFRTDLGPLTAELRRELLPHGGRTGAPVG
PDAPLPPGRPPRLLTRLLRGWEHIHSRALRRVALERCHRAVADEQHASPRHGLSSAGALVECLALYARDPGHPLLEGAVTRLSHWRWTDARDGV
RLRDDRSTVRDTAFAVQALLAADPGHVPDAVHRARTRLAAARVTTYTGLVPPLRTVLGGWALSGGDCPWPAADCTAEALNALLHPEGSAPPPLP
PHALRAALEVMLDRQNRDGGFGILDRQRAGRWLEALDPAGMFPGRMTDTSCADCTGSVLTALGRLRRHLGPGDRRRAEAATGRAVAYLRAAQNP
DGSFAGTRGIHPTYAAFHAARGLRAAGVRRDDPAPAALGRWLAAAQLADGGWGEDWRGVVEGRHLPLGHGLPETTGWAVLAALDTLGPRHPVVD
RGVRWLCDHQRPDGSWENGHVNGVLFTAGMVNHRLGAACFPALALGRY
>S-alboviridis
LQGRDGSWEGEMAWSVMILSQYVITRHLLGRPLPPGEIGPIVQHYRVKRLRQTGWGLHAASAGPSPYCTSLAYIALRLLGLAPDHPLCRPARQW
LRTQPGGVAAIPSWGKFWLALLGLYDYRAMHPLLPELFLLPKWLPLHPDRLYCHTRTISQAMTYLYGTRFQGPQGGVVEDLRRELFDRPLRRSD
RHRTGIDAAVSPTVPLRLLQRALALYERRPPLALRRRALDRCRARVEHEHAVTGRLGLSPVNSLLDILVLHASGEKPDKVDRSLTAFDYWRWTD
PHEGSRYAGARSQTWDTAFAVEALLADHAPCPDTARAVARANRFLTGAQITTEIPTPHLTARSPARGGWCFSEGGHSWPVSDCTAEAVSALLSG
SDAGRADPTFDISAATEFLLARQNRDGGFGTYEARRASRLMEHLNPAEMFTHCMVEGSYTECTGSAAVALVHLQGRVNGTQRHACKRALSRART
FLLSTQDADGSWPAAWGINRIYGTLFAIRGLLATGVPRTHPCFVRAGWWLESIQLADGGWGEDHTGCIDNRYVPGATSQPVSTAWALLALLELT
GGRSRAVLSGIAWLCDRQLPDGSWPETAATGVFFGTAMLDYRLYREYFPLWALGRW
>G-obscuriglobus
MPHDPTARAAARLTETQQPAGCWEGEMIWCPVVTAQVAITRHVVGMPFSDADAAKIIRHFEFSQLPNGAFGLHPEHPGSVFVTTLVYVAARCLG
VSAEHAVTAKARGWLHAQPGGVLSAPTWGKFWLTLLGLYGRDGLRPLLPELALLPKAFPVHPVRFYCHTRYVYLVMSLLQGAHATFDLGPLRAE
LERELYGPLAVPESFRQYRYRLAETDAFEPPNLFIRVAERVMGWYDRVALPGLRRAALKRCADLIDLDLDANGYLTLSPVNGTLNALALFARGA
DREVIAKCVSGFEFYRFDDPERGLRYSGGSTRTWDTGFALEALLANPAVASVYRDVVHRGYRFLAAHQMSKSVAGRDPSFPDTARGGWCLGDGG
HAWPVSDCTAEALSAVLSAHTHGMAPEERIPDARLIQAAEFMLTRQNRDGGFGSYERARSPRWLERMNPSEMFTRCMTDQSYIECTGSCLVALG
RFRKAIPHHAAGRITRATNRGARFLLSRQRPDGAFPGAWGVYLTYGTFHAVRGLRAAGYAPSHRALQRAANWLIATQKRDGGWGEDYHGCLRQE
YVEHPESQATMTSWAIVALCETVGTGHPAVQKGAAWLASRQRADGSYPREAVNGVFFGTAMLDYDLYRAYFPTWALALASGTTAKS
>G-sp-IIL30
RAAEHLRALQTLDGYWEGEMIWCPVVTAQVAITRHVVGLPFSAEDTAKILTHFERTQTSDGAFGLHLEHSGSVFVTSLVYVAMRCLGVSADHPL
AVRARNWLHAQPGGILSAPTWGKFWLTLLGLYGRDGLRPLVPELALLPRAFPVHPIRFYCHTRYVYLAMSLLQGSHARFALGSLGDELRHELYG
SAGPPASFREHRYHLAPSDAFEPPNLLIRAAERVMGWYDRFPIRRLRESALQRCAHLIQLDLDVSDRLTLSPVNGVLNVLALHARGADRALIAK
CVEGFEAYRWDDAARGLRYSGGRTRVWDTGFAVESLLADPGAVRANRDALLRAYRFLAEQQVMKPVAGRDPMFPDRALGGWCLGDSAHSWPVSD
CTAEALSAVLGMHAAPELELDSRERISDTRLALAADFILSRQNPDGGFGSYERARSPRWLERLNPSEMFTRCMTDQSYIECTGSCLVALSRFRV
AVPHHATNRIDRAIRRGARFLLGRQHSGGAFPGAWGVYFTYGTFHAVRGLRAAGYGSNPALQRAADWLIRHQKPDGGWGEHYTSCLRQECVEHP
ESQATMTSWAVLALCEVVGAKHTAVCRGVEWLAAHRSGAGRHPREAVNGVFFGTAMLDYDLYREYFPVWALA
>Methylococcus
MKHLLSLQRSAGDWEGEMVWCTMILAQAVIVRTVVGRPYDARERAAIIRHFELSQLADGAWGMHPESRGYVFFTVLAYVALRLLGLGPETSMLA
RARAWLHAQPEGVKAVPTWGKFWLMLLGLYGREGVNAVPPELFLLPRWLPFHPSRFYCHTRLIYLGIAYLSGVGFSASLSDPLRDALRSELYAE
PYESVDFGAFRHTVARTDLYVPISRVLRLVYDLLARYERRPWKALRQRALTLCFEQILREQRSTRYQGISPVSGLLNCLAIFAHDPRHPDLAPS
LEGVEAWRWEDEAEGLRYVGARSNAWDTAFAVQALAELPELDEEAKHALSRAHAFLDQAQMTAELADYREAWRDPALGGWCFSDGRHCWPVSDC
AAEAMSALFALYERGDVRISEALGADRLRLGVEFILSRQNADGGFGTYERRRGGRLLELVNPSEMFGQCMTELSYVECTASSLGALAHYLRNYP

DLPGGKITAAIRKAERFLRSRQLDDGSFPGFWGINYTYAVFHVAKGLRMAGVEPADPVLQAAAGWLLEKQRSDGGWGEHYSSCLEGRYVESRHS
QTVMTAWALLALMEVYPAAHEAVERGIAWLCSQQGEDGGWPRQGMNGVFFGAAMLDYRLYPVYFPTWALARY
>Methylocaldum
RALRHLLDLQGPNGDWEGEMVWCTMILAQAVIVRTIVGRPYGDAEKARIILYFEKSQRDDGSWGMHPESQGYVFFTTLGYVALRLIGVPATSPM
LVMARRWLHSQPYGVKGIPTWGKFWLAMLDLYAYEGLNGIPPELFLLPEWVPIHPRRYYCHTRQIYLGLAFLYGMRFRASLPDKLRDQLRQELY
REPYETIDFPALRNTLAATDVYVPISPVLRKIYRLLAAYERRHSATLRQKAIDLCFERILYEQRVTRYQGISPVSGLLNCLAIFARDPTHPDLG
PSIEGVEAWRWEDEENGIRYAGARSNTWDTAFAIQALVDAPANVEGTAEALERAHEFLKNAQTTEELPDYEAAWRDPALGGWCFSDGLHRWPVS
DCTAEALIALLALYEHPLYTVASPIEPERLRQAVTFILSRQNADGGFGTYERRRGGKLLETINPSEMFGQCMTELSYIECTGSALAALGHYRKH
YPDFSGGAIERAIRNAVAFLRRRQLADGSYPGFWGINYTYAIYMASKGLRAAGMPASDPTLQAAARWLVRKQRPDGGWGEHYSGCLKGRYVAHV
RSQVVMTSWALLALLEILPPDHESVRRGFEWLIRQQRHDGGWPRQAVNGVFFGSAMLDYRLYHVCFPTRALSRY
>Plesiocystis
RALGALERAQQARGAWAGEVVWNPMLICQYVIAMHVLGREIPAERRRNIRRQLEVTRKRDGGWGMHPDPPEREPKPEPAFEPGPAPVSEHDSPD
AEAPAETMAGKFDSWMFHTVLGYVALRLLGDAHEPGEDPQRTAAMLEESLAWIHAHGGPERAPTWGRIWLALLGLYPWDLVQPLLPELWLLPDD
APMHPRRLYCHMRLIYLGLSYLWGARVQAPSGPVLDALAAELYPGGPSRARFEAARDDIAPTDLFEPVGAVLQWAFAAGRTLDRATDLPLLRPP
LAALRRRALDRAWAHIEFEFHSTDWVCLSPVNGMLFCLAMAKRDPDHPDLDRALAGLEYWVWEDAEEGARICGARSDIWDTSFALQALAEGPEL
ELGRALAQRAAAWLPKAQVMRELSTTAPGALPNYRSPTRGGWGFADERHPWPVSDCTAEALEALLHVEARGWIGEGQATPALSPARKLAAAEFI
LLRQNDDGGFGSYEERRGSMALIHFNPAEMYGNCMLEYSYAECSASCVRALAVLREREPALLASAGDLRARVDAAVDAGVRFLLEAVDPKAGAW
RGFWGVNYTYGTYFAVSALLAAGVEREHLVVRRAVRFLLDRQRADGGWAEDYRGLLERDSGTLLSRTLGAKREPRRGAPSWDLADDEASRVTQT
AWAVATLALAAPQRARQSVDAGLAYLLERQQADGTWEHDASVGVFFNTAVLDYRLYRQVFPTWALAR
>Enhygromyxa
QQPNGAWAGEVVWNPMLICQYVICSHIVGREIPEPRKQRIRLSLELQRKRDGGWGMHPDVPPAPGDHDHHPNHDRDPAGLAGAHDSWMFHTVLG
YVALRLLGADPNEPLLQDVRAWIHAHGGPYGVPTWGRAWLALLGLYPWSELQPLLPELWLLGDDSPMHPRRLYCHMRLIYLGLSYLYGARTQAD
ASPLVEAIRGELYPEGWDVSRFRATRDQIASTDLYEPIGQSLEWLFAGARALSRAIPAVVRRRALARAWEHIEFEFASTNWVCLSPVNGMLFCL
AMWSRDRRDPRLAQALQGLEYWMWEDDSEGLRICGARSDIWDTSFALQALCEGPQIDEAARAVDRASAWLPRAQLRADILGGREHYRESARGGW
GFANEHHPWPVSDCTAEALEALLHAEHRGWDHAAARLDLSDKLAAVEFVLLRQNDDGGFGSYEPRRGSMLLARFNPAEMYGNCMLEYSYTECTA
SCVRGLAVALEALGANMPSELRARVQAGVDAGVQFLLGSQASNGAWPGFWGVNYTYGTFFSVAGLLAAGLDGEHVAVRRAVRHLISAQRDDGGW
GERYEGVLESRERPLDADQPSRIVQTAWALLTLQQAAPERGREAIERGLRFLLDQQQPDGTWPHDAAVGVFFNTAVLDYRLYKLVFPTWALSRW
>Sandaracinus
RGARALIAEQADDGSFEGEVVWCPMLAAQYVMGWHAMGRPLSAERRASVLKHFERTRLADGTWGLHEKSEPYLFVTLLVFVACRLLGLAKDDPL
IARAHEFIRREGGAVAVPSWGKLWLAVLNLYSWEGVSAVLPEAWRAPRWLPFHPSRFYCHTRLIYLGMAVLYGEKWSAPVTPRILAIRDEIFPG
GWESVDWAKARETLRTAEIHTPWTPALHVGYRVLGAVDRLQSREKRATVLAELREHIRYELRSTNHTCISPVSGLLDQVALFIEDPNDPDLRIA
AERFEGWVWEDELDGARVTGARSASWDTAFAAQAMAAAAPHCGNDVRDALRRADTFLVTQQIPRGTGRERHHDRIDPTGGYCFAGVWHGWPVSD
CTAEAMLARLESPEGSPTREAMEAAARFVLRCQNTDGGFGSYEARRTDVSLEWINPAEMFGDSMTEKSYVECTASCVTALAAFVHRWPQSELAH
ECETAIARAVASLTRTQRPDGSWPGMWGVHFVYGTMFGVRGLLAGGVPPHDPRIRRACRFLEERQRADGAWGEHRSSVIVGRYVDHDEGQAVQT
AWAMTALLEARHPDFAPIERAARWLASKQSDDGAWPKQEAEGIFFHTALLDYVLYRRYFPVWALGLY
>SHC-Candidatus
ILERQLPDGGFNIYAGGPSEVSATIKAYCALKLAGLDPHSPPLRRARERILALGGLQAANSYVKINLSLFGLYPRKHVPSVPPEIVMLPGNVLY
EMSSWTRSILVPLSIVQARGSNRRAPNGFNLDELLLPGVKLALPKRKGLAVLFHHLDRMFKVWEKRGSERIRGAAIREAERWLIARTHYTEGLG
AIYPAMMYFIMALDALGYAEDHPDRSEAIRHFESLLIETDDRFLFQPCVSPVWDTAICAFALGEAGNTDDPRMTLAADWLISKEVRRKGDWSIK
RPDTEPSGWAFEFANEFYPDIDDTAMVLLALMHANGSNPEAQAAAERRAVNWLLAMQSSDGGWAAFDVDNNWAMLNQVPFADHNAMLDPTCPDI
TGRVLECLCRRGMAGHDAARRGVAYLLQAQEKDGSWYGRWGVNYIYGSFLAMRGLTTSGAPGSQDAVDRAARWLRAIQNPDGGWGESCASYARD
GYVAAPSSASQTAWALLGLCAAGDRDSAQFRRGVEYLLTLQAPDGKWPEGATTGTGFPNVFYLTYAMYRDYFPLLALSQ
>SHC-Hyphomicrobium
LQRPDGHFVFELEADATIPAEYVLMRHYLGEPVDTVLEEKIARYLRRIQSDDGGWPLFHDGVSNISAAVKAYYALKMIGDSPDAPHMKKARAWI
LAQGGASHSNVFTRNLLALFGSIPWSGVPVMPVEIMLLPRWFPFHIDKISYWARTVLIPLTVLNALKPRARNPKGIGIAELFATPPEQVRNWPK
GPHQKFPWSQVFGGIDRVLRLVEPAFPKSLRKKSIDKAVAFVTERLNGEDGLGAIFPAMVNSLLVYDALGYPQDHPDYVTARGSIEKLLVVKDD
EAYCQPCLSPVWDTALAAHALMESGGAQTEQSVDRALAWLKPLQVLDTVGDWAATRPGVRPGGWAFQYANPYYPDVDDTAVVVMAMDRAAGRDA
SKREQYRESMARAREWIAGVQSKNGGWGAFDADNTYEYLNQIPFSDHGALLDPPTADVSARCVSMLAQLGERRDTSAVLDKALAYLENTQEKDG
SWYGRWGMNYIYGTWSVLCALNAAGVDPSAPPMRKAVDWLVSIQNSDGGWGEDGESYSLDYKGYERAPSTASQTAWALMGLMAAGEVDHPAVKK
GVTYLTSKQGSDGFWGEERFTATGFPRVFYLRYHGYSKFFPLWALARY
>SHC-Leptospirillum
PLDPVRRDKIVRAILSVQGKEGAWPLFHDGDPDISATVKAYQALKLCGFDPSHPALVRAREWVLSQGGAGKVNVFTRIALAIFGQYSWTKIPAL
PAEMVLLPSWFPFSIYSVSYWSRTVIVPLLFIYHHKPLVRLSPERGISELFDPARPDGESFAPSPDFFSLRNLFLLLDKVLQVWNRHPPGFLRK
KALSFAMEWMVPRLKGEGGLGAIYPAMANSAVALSLEGYELDHPLMQRVLASIDDLLIEGEKEVLVQPCVSPVWDTALAMGALIEAGISPDSPT
VDRAMEWFCAREVRTRGDWAIRAPDCEPGGWAFQFENDYYPDVDDTAMVLMGMAKILPARPDLAARMEGVFRRATLWVMAMQGTDGGWGAFDRD
NDLLFLNHIPFADHGALLDPSTADLTGRVLELLGALGYGPDFPPAARAIRYLRREQEEDGSWFGRWGVNYIYGTWSVVAGLKSIGVPMSEPWVM
RSMEFLLARQNPDGGWGEDCLSYASRDFAGRGASTPSQTAWALIALLHGGHAGHMAVRQGVDYLIQQMTPEGTWNEELFTGTGFPRVFYLRYHM
YRHYFPLWALALY
>SHC-Chlorogloeopsis
NYLLSIQNPSGYWWAELESNVTITAEVVLLHKIWGTDRSRALHKVETYLRSQQRQHGGWELFYGDGGELSTSVEAYMALKLLGVPQNDPAMIKA
REFILERGGISKTRIFTKLHLALIGCYSWEGIPSLPPWVMLLPENFLFNIYEMSSWARSSTVPLLIVCDRKPVYKVEPAITLNELYAEGAEQIK
FTLPSKGDWTDLFISLDSAFKFAEKLNLVPFREEGINAAERWILERQEATGDWGGIIPAMLNSLLALRSLDYDVNDPIVERGLQAVDNFAIETE
DSYRVQPCISPVWDTAWAIRALVDSGLSPAHQALVKAGEWLLNKQILDYGDWAIKNRQGKPGAWAFEFDNRFYPDVDDTAVVVMALNAVHLPNE
KLKHRAIARAVNWIVSMQCRAGGWAAFDLNNDQDWLNLIPYGDLKAMIDPNTADVTARVLEMVGSCNLSMDASNLQRAINYLIAEQEPQGCWFG
RWGVNYIYGTSGVLTALSVIAPQTYQNNIERGAAWLIECQNPDGGWGETCRSYDDPSLKGKGRSTASQSAWALIGLIAAGKATGNFAKPVLERG
INYLLATQAADGTWDEADFTGTGFPCHFYLKYHLYQEYFPLIALSQY
>SHC-Streptomyces
QDERGWWKGDLETNVTMDAEDLLLRQFLGIQDERITRATARFIRAQQRPDGTWATFHGGPGELSTTIEAYVALRLAGDAPEDAHMAAASAWVRE
QGGIAASRVFTRIWLALFGWWRWDDLPEMPPEIMYLPKWLPLNIYDFGCWARQTIVPLTVVGAHRPVRPAPFALDELHTDPRRPNPPRPLASPA
TWDGFFQRLDKVLRAYRKVSIGPLRRAAMNAASRWIIERQENDGAWGGIQPPAVYSIIALHLLGYDLDHPVLRAGLASLDRFTVWDKTEDGEPV
RMVEACQSPVWDTCLATVALADAGLEPDHPALVKAADWMLEEQITRPGDWSVRRPELPSGGWAFEFHNDNYPDIDDTAEVVLALKRVRHPQPRR
VEAAVERAMRWTEGMQSRNGAWGAFDVDNTSPFPNRLPFCDFGEVIDPPSADVTAHVVEALAVLGRQHGERARRGIAYLLAEQEENGAWFGRWG
VNYLYGTGAVVPALVTAGLPVSHPAIRRAVGWLESVQNDDGGWGEDLRSYRDRDWIGRGASTPSQTAWALLALLAAGERDGEAVRRGIDHLVRT
QREDGSWDEPYFTGTGFPWDFSINYHLYRQVFPLTALGRY
>Verrucomicrobia
VITTYLTRQPVTEHDQPRFVAGLLQPQLPDGSVGLHEESVRGAVFTSAISYVALRLLGEKPSRPELAKMRDWIEKAGTPVKAAAWGKFILSILN
LYDWSGVTPVPPELYLLPKWVPVQPINISGYVRIVYLPMAYFYGRRWQAPLDPLLREIRRELFPQGFDQIDWPKHRADLAPTDHIVPETLLVRI

AMPIVRYLEKWIPSSVRRKALRLTYEHICYEDEQSDYIRQAPVNACYNTLAHFVEGQTSRVARSWEQLPRYLWNHPDHIACQGFTSSKVWDTAF
TLQGMSHLEPSLAPKQSIQEGCRYLVENQVIDELPDPRRYHRLPRKGGWPFSERKNGWSIADCTAESMLALIAAKPFLAQPPSPKILEDGLRFI
LSYQNRDGGWGSCDRVVGPLWIEKFNASHVFADIMVDHSFAECTGSVLSALALVRKEYPHLETKRVDHAIREGVRYLTDTQRPDGSWEAVWGIC
FNYGTSFAIPGLLSAGLPQDDIRIVRGRKFLLQQQLPDGGWGEHPDSCLERRPIPTPKSLVEPTALAVLALLGCGPKEDPSVRKGIEFLLQQQQ
ADGDFPPQPIPGLFYRTTLIRYDHYKRAFPLKAFAKY
>Eudoraea
QTEEGTWPVPYDGPFFLLPLYIFAMRICGRHMSVETKTKMSRYILRHQLDNGSFGIHQESKSGTVFTSVINYVALRFLDHSASEPELKKALNWI
HTKGGPLYSASWCKFILSFLNLYSYKGVAPVPPELYMLPGWFPFHPRKISGYVRIIYLPMCYFYRKRLSIETDELILELRKELYRQPYDQINFA
KHRGTFADTDNIFPETTIFKISMWWLKRIDPLIPRFIKKKALALVYEHIEYEDENSNFIRQAPVNAVYNTLVYHFKGEKEKLEKSWAKLPLYLW
ENDDQILMQGFTNTYTWDCGFYLQAIGKDATEPALKPLAIKTRKFLLENQVTKELKNPFKYHRLPRAGGWTFSFQDNGWVVSDCTAEAIKALLE
TEGLGDEPLSDERINKAISFIMLLQGKDGGWTSVDKAIGSPKLEWFNAANVFVDIMVDHSYVECTASIIQCFAQIKRSRPHLFTSEMESAMERA
VKYLLKSQYEDGSWEALWGLCFTYGTCFVLEGLSMYGMNKDHEAIQRACTYLWSKQKDDGGWGEAQESALAREYIQAENSVVDQTAWSIIALLN
GGYAEDPRLIKAVNWLIDQQLEDGDWPVQPMSGLFYKTTMISYRNYKRYFSLLALKKY
>Ochromonas
ASKGISFYQMLQSEDGHWAGDYGGPMFLMPGLICVLYITKTPFPPGRREGMIAYLRNHQQKDGGWGTHIECASTMFGTVLNYVALRLLGDEADA
PHMEKARSFIHRYGGALYAPSWAKFWLAVIGVYDWDAVNSIPVEMWLLPRWFPFHPGKLWCHCRMVYLPMGYLYSKRFTPDVANDPTLTSLRTE
LYLQKYDTIKWDDFRQTCADIDEYSPLNPIMKVAQDFLAIYEKVLPHIPFLKNLRQKGLEFAIDYIHAEDLQTNFIDIGPVNKALNMLSVWVDN
GENSNEEKFLRHAARVDDYLWVAEDGMKMQGYNGSQCWDTAFATQAIVESGLAPHFKDCLTKSYIYFNRTQIKNDEDNRDYYYRHISKGGWPFS
TAAHGWPISDCTAEGLKAVLCMHSSEKLKALVPADKLLTPERFYDACNVILSYQNEDGGWATYENNRGYGWYELLNPSEVFGDIMIDYSYVECS
SACITALKALQREFPDHRKEEVIQSIAKGREFLKSIQRSDGSWYGSWGNCFTYGTWFGIEGLIAAGEPLDSKNMRRAVQFVLSKQNANGGWGEN
YLACVNKHYPEDGTGKNLGDEGSGVVQTAWALLSLMAAESSDLKAIQRGVQFLISKQLPSGDWDQEGITGVFNRSCGITYTAYRNVFPIWALAR
F
>Chromulina
AEKALAYYQMLQCEDGHWAGDYGGPMFLMPGLICVLYVTGQLHTTFPQYKIDAMILYLKNHQQEDGGWGTHIECASTMFGTILSYISLRLLGIS
SNEDFMKLGRKFLLDHGGARYAPSWAKFWLASIGVYDWKGINSVPVEMWLLPQWFPFHPSKLWCHCRMVYLPMGYVYCKRFTPDVDNDELLQSL
RKEIYLEDYDTIDWDGSRQLCADIDEYSPLNPVMKLAQDFLSIYERVLPHIPFLKQLRQRGSDFAIDYINAEDIQTNYIDIGPVNKSLNMLSVY
ISSNGDSNNERFKRHIPRIDDYLWVAEDGMKMQGYNGSQCWDTSFTIQAIIESNLSNKFSNCLTKAYSYLNRTQIAEDEVNREYYYRHVSKGGW
PFSTAAHGWPISDCTAEGLKSVLLVHTLDTISTSDRISSNRLQDACDVILSLHNKDGGWATYENNRGYGWYEMLNPSEVFGDIMIDYSYVECTS
AAITALVKFSKQYPKYRANEVWNAIQSGRHFIKSIQRYDGSWYGSWGTCFCYGTWFAIEGLIAAGESTTSPAIIKATEFLVSKQNPNGGWGESY
LACVNKSYPYDGTGETLGEDSSGVVQTAWAVLSLIKADYQDKLVIDRGIKFLLDKQLPNGDWDQEGITGVFNRSCGITYSQYRNIFPLWALGRY
>Chattonella
KGIEFYQMLQTEDGHWAGDYGGPMFLMPGLIICHYVTGTPLAAYKRDAMIAYLRNHQQTDGGWGTHIESASTMFGTVLSYVALRLLGVKAYDIG
ACRAREFIHEHGGALYTPSWGKFWLAILGAMEWDCINSVPPEMWLLPRWFPFHPGKLWCHCRMVYLPMCFVYGKRFTYPKAEQDPLITELRHEI
YCQEYAAIKWGRHRQTVSKLDEYDPVGLLMRTLQSILAVYEKYIPFKLGRESALKFAFDYIEAEDRQTNYVDIGPVNKSLNMLCVWMERGSDAE
EFQRHICRVDDYLWVAEDGMKMQGYNGSQCWDTSFAIQGIVESGLAEEFPEMCLKVYEYLDNTQIKTNEENREHWFRHISLGGWPFSTAAHGWP
ISDCTSEGLKGILSLNSLQFIKDSGKVLSDERMFQAANVLLSYQNHDGGWATYENNRGYGWYEYLNPSDVFGDIMIDYSYVECSTACISALSNF
QKAYPEHRKDEIKNSIEQGRKFVKSIQRPDGSWYGSWGVCFTYATWFGIEGLVVSGEPIDSPSIKKACEFLVQHQNENGGWGESYLSCVDKAYA
EDGAGELGNGKSGVVQTAWALLGLMEAHYPDRAVIDRGINYLRAQQNQIGDWAQEGITGVFNRNCGITYTAYRNIFPIWALGRY
>Synchroma
ARKGVAFYQMLQCDDGHWAGDYGGPMFLMPGLIAVLYLTGSMPPRYKQNAMALYLLNHQQSDGGWGTHIEGASTMFGTVLSYVALRLLGEGPDA
PHMAAARAFIHENGGALYTASWAKFWLAVLGLYEWEGINSIPAEMWMLPRAFPFQPGRLWCHCRMVYLPMCYAYSSRFSARAEDDELLSALRRE
LYLQPYETIDWDAHRFSICPFDNYSPVEPVMRLACNALALYERYLVGLAPFRWLRSAGLRFVMDYIHAEDAQTNYVCIGPVNKALNMLAVWVDA
GRDPTCEAFRRHLQRVDDFLWVAEDGMKMQGYNGSQCWDTSFAIQGIVEAGLDAEFPDMVRRVYSYLDRTQIATDEDRAEFWFRHVSKGGWPFS
TSQHGWPISDCTAEGLKAVLALADSPCVRDHVPLLSEERICDAVNVILTLHNADGGWATYENNRGFGWYELLNPSETFGDIMIDYSYVECTAAC
MQALAAFRARYPAHRAAEVGSAIAAGARFIKSIQREDGSWYGSWGVCFTYGTWFGIEGLAAVGEADGPEVARAVRFLLAHQNDDGGWGESYLSC
MDKAYAEEGTGGGALGQDGSGVVQTSWALLGLLAAGCEDRDAMRRAAELLMRRQRDDGDWDQEAMTGVFNRSCGITYTAYRNVFTTWALGRY
>Phaeodactylum
FYSMLQTSDGHFSGDYGGPHFLMPGLIVVWYVMGQPSLMLNPAQTALMKHYLIVHQQADGGWGTHVESPSTMFGTTLSYVALRLLGMDAEEPVC
QRGRAFIREQGGAVMTSSWAKLYLCILGCMEWDGHNSVPPELWLLPNWFPFHPSRMWCHARMVYLPMGYVYGARLKYDKAEEDPLVQALRRELY
CEPYNSIEWMQTRHMVAPMDNYSPVAWMMKTVQNGLARYETWPMLQPFKNDVRKLGLAFCVDYMAAEDLQTNFIDIGPVNKVLNMLSAFHHAGN
DLHHSTVMNHMIRVQDYLWVAEDGMKMKGYNGSQCWDTSFAIQAVFEAGLLDDFPELSNKVWTYLERCQILSTEVSQASPAFKYEAALYRRKFY
RHISEGGWPFSTSAHGWPISDCTGEGLKGVLCMLKAKSVREGLEDGSLREISEVRLQKAANILLSYQNEDGGFPTYENNRGFGFYESLNPSEVF
GDIMIDYSYVECSMASLTALADFHEDYPDHRTEEIVHAIEKGRDFLKDLQREDGSWYGSWACCFCYGSWFGIEGLVKCGEPVSSEFIAKACKFL
LQHQRSNGGWGEDFTSCYDKEYAANGMEAYGDDGSGVVNTSWALMALSTAKCNDIEAIKRGVQYLMKRQLPCGDWPQEGVAGVFNRACGITYTA
YRNIFPIWALGR
>Helicotheca
AKKAIHFYSMLQTEDGHWAGDYGGPHFLMPGVIIAWYVMGQPEEMLDKHQREMMMHYIAVHQQSDGGWGTHIESPSTMFGTVLNYVALRLLDRD
ETHKSKYEKAVSFISAQGGAIMTSSWAKFWLCLLGCMEWDGHNSVPPEMWLLPNWTPFHPGRLWCHCRMVYLPMGYLYGARFVYPDAEKDPLIS
SLREELYIEPYESIDWIRTRHFVAPMDNYSPIPWTMEFLQNCLARYETWFIFQPFKNWVRKKALKFSVEYMDAEDLSTNYIDIGPVNKALNMVS
QFHAAGNDVTAPKVKKHMMRIPDYLWLAEDGMKMQGYNGSQCWDTSFAIQAVYECDLLDEFPELSKKVWSYLERTQILSTEVSQNTPAFEGESA
INRSKYYRHVSLGGWPFSTSAHGWPISDCTGEGLKGVLSLTHSKIVSESIKEGRLKPISRSRLEDAVNVLLMYQNEDGGWATYENNRGFGWYEQ
LNPSEVFGDIMIDYSYVECSMASLTALVEFHEMYPDHRAMDISAAVGRGKDFMKSIQREDGSWYGSWACCFTYGCWFGIEGLIKAGESNTCPEI
QRCCAYLISKQRENGGWGEDFTSCYDKDYAVNGMKDYGDDGSGVVNTGWALLALSAAKCDDVAAIRKGVQYLMKRQLPCGDWPQEGIAGVFNRS
CGITYTSYRNVFPIWALGR
>Aureococcus
KALAFYQQLQCDDGHWGGDYGGPHFLSPGLVVVWYVTGRRDDVLDEHQRRAMVRYYENHQQTDGGWGTHVESPSTMFGSVLTYVALRLLGEPAD
APACAAGRKLILEQGGACYTSSWAKFALCLLGAMDWEGHESVPPEMWLLPCWCPFHPCRMWCHARMVYLPMGYLWGKRWTYENADSDPVVLALR
DELYPASPAYGAIPWRATRSWVAPMDDYSPVHPLMVAAQRFLRVYEDLGGPLRRYARRKGLAFSADYCRAEDLQTNYVCIGPVNKVYNMLVAYD
DRHADGGEALARHALRVPDYLWVAEDGMKMQGYNGSQCWDASFATQAIAESDLGDDARFRDCAKKAWSYLERTQILSTTTSQASPAFAFEAPKL
RERYFRHVSKGGWPFSTSAHGWPISDCTAEGLKSVLALRSLACVGECAPIGYERLCDAADVVLALQNADGGYATYENTRGYGWYEWLNPSEVFG
DIMIDYSYVECSMALARFREACPDHRAAEISAALKRGNAFLRSIQRADGSWYGSWACCFTYAGWFGIEGLVDSGEDLREDPKTSEPVARACAFL
LRHQRPNGGWGEDFTSCFDKAYAKHGMEAYGDAEGAGVVCTGWALLGLMAGACADADAVARGVAYLEARQLPDGDWPQEGISGVFNRSCGITYT
AYRNVFPMWALARY
>Gymnodinium
ALYAPSWAKCWCCILGVYEWKGMAPIPPELWLLPSWFPIHPGRFWCHARMVYLSMCYLYGRRFAYDAESDPVTAALRSELYPGREYAKIDWDNH
MHSIADVDNYSPIHWAMRALQRALTVYERLGAVRCVRDRALRFVEEYMISEDIETNYLDIGPVSKAMHIVCMWVIEGGETDPKAAGNSKAFRAH
VARVPAEAGLDGEFSEMCCKAHGWFVREQVRALPNGDWRHWRQPIRGGWGFSTAEQAWPVSDTTAEAFLAVLALRRKDCIASCCPMSDEQCFDA

VQFLLSYQNDDGGWATYENRRGWKWYECLNPSEVFGDIMIDYSYVECSSSAMGALSVFRQHFPHHRRGEIDRAIRRGGRFIKQMQREDGSWYGC
WGNCFTYACWFGVDGLLHSGETPECSDAIKRCMRFLIGKQNEDGGWGEDFASCFNREYAAMDQLYGSDSGSTAVQTAWALLALMAGDCADTTAV
RRGIRFLMRRQLSSGDWAQENIAGVFNRSIGITYTSFRNVFPLWALGRF
>Alexandrium
ARRGFAFYQTLQCDDGQWAGDYGGPHFLLPGFVIAAYIVGRSRVFSDAHCRAIEAYLRNHQQEDGGWGTHIESPSTMFGSVLNYTALRLIGVPE
EDAACIQARNFLAKHGGALHAPSWAKFWLALLGVYEWEGIAPVPPEMWLLPHWFPVHPGRFWCHCRMVYLPMCYLYARRFSYDAAADPLTASLR
RELYPTKCAYSEIRWGDYMHAVADIDNYSPIHWAMRLIQNLLCVYERWGPWRLIRDRSSRFAEEYVHSEDVETNYLTIGPVSKALHIVVSWASA
GGQRDPETASQSKAFLAHVARVPAYLWIAEDGMKVQGYNGSMAWDTSFAVQAVAEAKLQDEFPEMCAKACGFLAREQVRVLPNGDWRHWRQPIR
GGWGFSTAEQAWPVSDTTAEAFKAVLCLRGEQCTADAPALSGEQLFDSVQFLLSYQNGDGGWATYENCRGWKWYELLNPSEVFGDIMIDYSYVE
CSASSMGALSLFAKQFPDHRKAEIARAIRRGAQFIKSMQRQDGSWYGCWGCCFTYGCWFGIEGLVCAGEDPARCTAIQRCKTFLLEKQNDDGGG
ARTSQAVSTXEYASRKKLYGCEAGSTVVQTAWALLGLMAGECEDTAAVRRGIEFLMRRQLPSGDWAQENVAGVFNRSVGITYTAFRNVFPLWA
>Dinophysis
ARKGFAFYQMLQCDDGQWAGDYGGPHFLLPGFVIAAYITGGGDLTEVMAAPYWRAAEAYLRNHQQADGGWGTHLEAPSTMFCSVLNYVALRLVG
APPDDAACVRGRGFLHEHGGALYTAPWTKFWLAFLGVYDLRGIAPVPPELWLLPSWFPLHPGRFWCHCRMVYIPMCYLYGVRFAYDAAADPVDR
RPAGGAVPAGRGVRQDRVAQAMFSIAGMDNYSPVHWVMRALETVFAFYEWTTGPIGFLRRWSLRFVEEYMHAEDVETNYLTIGPVSKAFHVLTS
WVAAGGAEDPGLARKSDAFRAHVARIPAYSWIAEDGLKVQGYNGSMAWDTSFAVQAAVEAGLQGEFPDMCARAWGWIARDRTATGGTGARPSEE
AGGFSTAEQAWPVSDTTAEAFKAVLALRGDACVASCPEFPDEHCFDTVQFLLSYQNADGGWATYENNRGWAWYELLNPACVFGNIMIDYSYVEC
SASAMQALMDFHGQFPGHRAREIKRAVARGATFIEAMQRHDGSWYGCWGSCFTYGCWFSVEGLVCAGRDPVASPAIRRCVEFLLSKQNADGGWG
EDFASCFNREYASRERLYGCDSGSTVVCTAWALLALTAGQCHDRSAVRRGAEFLMRRQLPSGDWAQENICGVFNRSVGITYTA
>Scrippsiella
ARQGFAFYQKIQCDDGQWAGDYGGPHFLLPGFVIAAYITGGLTKTMPAPYWSAAEVYLRNHQQADGGWGTHLESPSTMFCSALNYVALRLVGAP
ADDPACVKGRAFIHKHGGALYTAPWTKFWLTFLGVYDYRGLAPVPPELFLLPSWFPVHPGRFWCHCRMVYLPVCYLYGRRFAYDAAADPLTAAL
RRELYLPGTEYSKIDWHNHMFSIADIDNYSPVHWFMKVAERIFAMYEWSIGPWKFLRDPALRFAEEYMHAEDMETNYLTIGPVSKAFHIVTAWV
AAGGEKDALAASKSKAFRAHLARIPAYMWVAEDGLKVQGYNGSMAWDTSFAIQAASEAGLQKEFPEMCAKAWGFLAREQVRALPEGDWRHWRQP
IRGGWGFSTAEQAWPVSDTTAEAFKAVLALRGDVCLGSVGEAPPFPDEHCFDSVEFLLSYQNADGGWATYENCRGWAWYELLNPACVFGNIMID
YSYVECSSSAMQALVDFRRQFPEHRAAEIIVSVERGARFIEAMQRDDGSWYGCWGNCFTYGCWFSIEGLRCAGRDPATNSAIKRCVAFLLSKQN
ADGGWGEDFLSCFNREYASRDKIYGCDSGSTVVCTAWALLGLMAGDCADSAAVHRGIKLLMRRQLPSGDWAQENISGVFNRSVGITYTAFRNVF
PLWALGRF
>Prorocentrum
LFGSVLNYVALRLVGVPPSDPACVEGRRFLRQHGGALFAPSWAKFWLCVLGVYDYRGIAPVPPEMWLLPAWFPLHPGRFWCHCRMVYLPMCFLY
GRRFVYPAASEDPVTLSLREELYAVDERYSDIRWGNFLHTIADVDNYSPIHWAMRLLQNALAAYEAIGAWAWPRRASLRFAEEYIASEDLETNY
LTMASGGEADAASARESRAFRAHVERIPAYLWVAEDGMKAQGYNGSMMWDTSFAVQAVAEAGLQGEFADMSKRAFEFICREQVRALPRGDWRHW
LGFSTAEQAWPVSDTTAEAFKAVLALRKDGCTKGSNDMPDQHYFDTVQFLLSYQNADGGWATYENCRGWKWYEALNPSEVFGEIMIDYSYVECS
ASSMSALAEFSKQFPDHRRQEIQRAIRRGRDFIKAIQRDDGSWYGCWGNCFTYGCWFGVEGLLAAGXAAALRAHPPCFLLQRQEPDGGWGEDFL
SCFNREYTTCDRLYGCDSGSSVVCTAWALLALMAGECDDTDAVRRGVDFLMRRQLPTGDWAQENIAGVFNRSIGISYTSFRNVYPLWALGRF
>Symbiodinium
GTHIESPSTMFGTVLNYVALRLVGVDAKTLECEKGREFMRQHGGALYAPSWAKFWLAVLGVYEWKGIAPVPPEMWLLPSWFPLHPGRFWCHCRM
VYLPMCWLYARRFTYKAADDPVTSALRCELFPDQTYAQIDWRRHVHSVAAIDNYSPIHPFMRWLQEALLXXXXXXXXIHPFMRWLQEALLVYER
FGPWKWLRKISSDFALEYINSEDLETNFLTIGPVSKALHLLVSWVEAGGDAKASESRSFQAHVQRVPAYLWVAEDGMKVQGYNGSMAWDTSFAM
QAAVDSDLVSEFKDMSKKAWSWLVKEQVRSLPYGDWKHWRQAIQGGWGFSTAEQAWPVSDTTAEAFKAVLLLRKQSCMKGESGQMPDQHLFDTV
RFLLSYQNGDGGWATYENNRGWSWYELMNPSEVFGAIMIDYSYVECSSSAMQALMLFTEQFPQHRAGEIARAVQRGARFIEAMQRNDGSWYGCW
GNCFTYGCWFGIEGLLCAGRPRSCAAIQKCVKFLLGKQNADGGWGEDFSSCFDREYAARDKLYGCDSGSTVVQTSWALLALMAAKCEDSDAIQR
GISLLWRRQLPTGDWPQENIAGVFNRSVGITYTSFRNVFPLWALGHF
>DreLSS
MTEGTCLRRRGGPYKTEPATDLSRWRLSNVDGRQSWRYIEETDSLDRPQSMLERHSLGLDTSEFISASPAAHTAVEAALKGMDFYSRLQAEDGH
WAGDYGGPLFLLPGLLITCHIAKIPLPDAWKKEMVRYLRSVQLPDGGWGLHIEDKSTVFGTALSYTTLRILGVGPDDPDMVRARNALHNRGGAV
GIPSWGKFWLAILNVYSWEGMNTLFPEMWLLPSWMPAHPSTLWCHCRQVYLPMSYCYAVRLSADEDPLVLSLRQELYVQDYSTIDWPAQRNNVA
TCDLYTPHSNLLTFAYFFLNVYEAHHSTILREKAVKELYDHIKADDRFTKCISIGPISKTINMLVRWYVDGPTSPAFQKHVSRIPDYLWLGLDG
MKMQGTNGSQLWDTAFAVQAFLEAGAQDIPRFTECLTQAHHFLDLTQVKDNPPEYEKYYRQMNKGGFPFSTRDCGWIVADCVSEGLKSVMLLQE
QCNFLKENIPKERLFDAVNVLLSMRNPDGGFATYETKRGGKLLELLNPSEVFGDIMIDYTYVECTSAVLQALKHFHSVYPEHRAEEIRSTLQRG
LDYCRRVQRPDGSWEGSWGVCFTYGAWFGLEAFACMGHTFQNGSVCEEVKRACEFLLAKQMEDGGWGEDFESCEQRRYVQSSSSQIHNTCWALL
GLMAVRYPGTKVIERGIQLLIDKQLPNGDWPQENISGVFNKSCAISYTSYRNVFPVWTLGRFTRLYPCNALTGKLKL
>Haplochromis
AVKGMHFYSQLQAEDGHWAGDYGGPLFLLPGLLITCHVAKISLPEAWKKEMVRYLRSVQLPDGGWGLHVEDKSTVFGTALSYTSLRILGVDPDD
PDMVRARNNLHSKGGAVGIPSWGKFWLAILNVYSWEGMNTLLPEMWLFPTWMPAHPSTLWCHCRQVYLPMSYCYAVRLAADEDPLVLSLRQELY
VQNYAFINWPAQRNNVAACDMYTPHSTLLTVAYMVLNVYEAHHSTTLRGKAVKELYDHIEADDRFTKCISIGPISKTINMLVRWYVDGPSSAAF
QEHVSRIPDYLWLGLDGMKMQGTNGSQLWDTCFAVQAYLEAGAQDDPKLAECLRDAHQFLTITQIPENPPQYQKYYRQMNKGGFPFSTRDCGWI
VADCTAEGLKSLMLLQELCPSIRQPVPSERLCDAVNVLLSMKNTDGGFATYETKRGGRLLELLNPSEVFGDIMIDYTYVECTSAVMQALRHFQK
VYPDHRAEEIRSTLREGLEYCRKVQRPDGSWEGSWGVCFTYGMWFGLEAFACMGHVYENGHVCEEVQKACQFLLDRQMPEGGWGEDFESCEQRR
YIQSGSAQIHNTCWALLGLMAVRHPDRKSIERGVQMLIDKQLPNGDWPQENIAGVFNKSCAISYTSYRNVFPIWTLGRF
>Anolis
AINGVQFYSGLQAEDGHWAGDYGGPLFLLPGLLITCHAAKIPLPEESKKEMVRYLRSVQLPDGGWGLHIEDKSTVFSIALNYTAMRILGVSPDD
PDLVRARNNLHSKGGAVKIPSWGKFWLAVLNVYSWEGMNTLFPEMWLFPSWMPAHPSTLWCHCRQVYLPMAYCYGRRLTAEEDELILSLRQELY
VQDYSTIDWPAQRNNVASGDIYTPHSWLLTVAYAILNTYERFHSTSLRQHALEVLYDHIRADDRFTKAISIGPISKTINMLVSWYVEGDKSPAF
QEHVSRIPDYLWLGLDGLKMQGTNGSQLWDTAFAVQAFLEAGAHKKPEFNSCLLHAHEFFRISQIPDNPPDYEKYYRQMNKGGFPFSTRDCGWI
VADCTAEGMKSVMLMQEKCPFIKDHIPPSRLFDAVNVLLNMQNADGGFSTYETMRGGWLLELLNPSEVFGNIMVDYTYVECTSAVMQALKHFHE
RFPEHRALEIREVLQKGLQYCRRLQRADGSWEGSWGVCFTYGMWFALEAFACMQHTYRDGVACKEISRACEFLISKQMEDGGWGEDFESCEQRT
YVQSATSQIHNTCWALLGLMAVRYPDVKVLEKGIKLLIDKQLPNGDWPQENISGVFNKSCAISYTSYRNVFPIWTLGRF
>Alligator
ALNGMQFYSGLQAEDGHWAGDYGGPLFLLPGLLITCHVAKIPLPEGYREEMVRYLRSVQLPDGGWGLHVEDKSTVFGTALNYTSLRILGVGPDD
PDLVRARINLHSKGGAVGIPSWGKFWLAVLNVYSWEGMNTLFPEMWLLPAWFPAHPSTLWCHCRQVYLPMSYCYASRLTAEEDELVQSLRQELY
IQDYSSIDWPAQRNNVAACDLYTPHSWLLTVAYAILNLYEAYHSYHLRQRAIEELYDHIKADDRFTKAISIGPISKTINMLVRWHVEGSSSLAF
QEHVSRIPDYLWLGLDGMKMQGTNGSQLWDTAFAIQAFLEAGAQDNPEFSSCLRRAHEFLRISQIPDNPPDYQKYYRQMNKGGFPFSTRDCGWI
VADCTAEGLKSVMLLQEKCPFITEHVPPQRLFDAVNVLLSMRNSDGGFATYETRRGGRLLELLNPSEVFGDIMIDYTYVECTSAVMQALKHFHE
EFPEHRAQEIRETLRKGLEYCRRMQRSDGSWEGSWGVCFTYGTWFGLEALACMQHMYHDRTACKEVAQACQFLVSKQMEDGGWGEDFESCEQRR
YVQSATSQIHNTCWALLGLMAVRYPDVEVLEKGIKLLMDKQLPNGDWPQENIAGVFNKSCAISYTSYRNVFPIWTLGCF
>Charadrius

MRFYAALQAEDGHWAGDYGGPLFLLPGLLITCHTVKIQLPEGFRKEMVRYLRSVQLPDGGWGLHVEDKSTVFGTALNYIALRILGLGPDDPDIV
RARVNLHSKGGAVGIPSWGKFWLAVLNVYSWEGMNTLLPEMWLLPTWFPAHPSRLWCHCRQVYLPMSYCYARRLSAEEDELVRSLRQELYVEDY
ASIDWPAQRNNVAACDVYTPHSWLLGVAYTIMNVYEAHHSTHLRQRAITELYDHIKADDRFTKCISIGPISKTINMLVRWFVDGKNSPAFQEHV
SRIPDYLWLGLDGMKMQGTNGSQLWDTAFAIQAFLEAEAQKMPEFTSCLQNAYEFLRFTQIPENPPDYQKYYRHLNKGGFPFSTRDCGWIVADC
TAEGLKSVMLLQEKCPFIAKLVPPERLFDAVNVLLSMRNSDGGFATYETKRGGHLLELLTPSEVFGKGSCDIMIDYTYVECTSAVMQALRHFHD
QFPEHRAPEIRETLQKGLDFCRKKQRADGSWEGSWGVCFTYGTWFGLEAFASMQHTYQDRAACREVAQACQFLISKQMADGGWGEDFESCEQRT
YVESAMSQIHNTCWALLGLMAVRYPDINVLERGIKVLIDKQLPNGDWPQENIAGVFNKSCAISYTAYRNVFPIWTLGR
>Xenopus
AYNGITFYSALQAEDGHWAGDYGGPLFLLPGLLIACHVTKTSLPDATKKEMIRYLRSVQLPDGGWGLHIEDKSTVFGTALSYTSLRLLGVSQDD
LDLTRARNNLLSKGGAVGIPSWGKFWLAVLNVYSWEGMNTLFPEMWLLPHWFPAHPSTLWCHCRQVYLPMSYCYATRLSAHEDDLIRSLRQELY
LEDYSSINWPAQRNNVASCDIYTPHSTLLHIAYAFLNVYESYHIPALRRRAVHELYDHIAADDRFTKCISIGPISKVINMLVRWHVDGSESSVF
REHVDRIPDYLWLGLDGMKMQGTNGSQLWDTAFAVQAYLEAGAHRRKEFQNCLEKAHEFLRISQIPDNPPDYKKYYRQMNKGGFPFSTRDCGWI
VADCTAEGLKSVMLLQEQCPFLTDLVPPERLRDAVDVLLSMRNSDRGFATYETKRGGLLLELLNPSEVFGDIMIDYTYVECTSAVMQALKHFQA
RDPNYRAQEIRETLQKGLDYCCSVQRQDGSWEGSWGVCFTYGIWFGLEAFACMGHTYKEGCPEIIRACNFLLSHQMEDGGWGEDFESCEQRRYV
QSAGSQIHNTCWALMGLMAVGFPDVTVLERGVRLLLDKQLSNGDWPQENISGVFNKSCAISYTSYRNVFPIWTLGRF
>HumanLSS
MTEGTCLRRRGGPYKTEPATDLGRWRLNCERGRQTWTYLQDERAGREQTGLEAYALGLDTKNYFKDLPKAHTAFEGALNGMTFYVGLQAEDGHW
TGDYGGPLFLLPGLLITCHVARIPLPAGYREEIVRYLRSVQLPDGGWGLHIEDKSTVFGTALNYVSLRILGVGPDDPDLVRARNILHKKGGAVA
IPSWGKFWLAVLNVYSWEGLNTLFPEMWLFPDWAPAHPSTLWCHCRQVYLPMSYCYAVRLSAAEDPLVQSLRQELYVEDFASIDWLAQRNNVAP
DELYTPHSWLLRVVYALLNLYEHHHSAHLRQRAVQKLYEHIVADDRFTKSISIGPISKTINMLVRWYVDGPASTAFQEHVSRIPDYLWMGLDGM
KMQGTNGSQIWDTAFAIQALLEAGGHHRPEFSSCLQKAHEFLRLSQVPDNPPDYQKYYRQMRKGGFSFSTLDCGWIVSDCTAEALKAVLLLQEK
CPHVTEHIPRERLCDAVAVLLNMRNPDGGFATYETKRGGHLLELLNPSEVFGDIMIDYTYVECTSAVMQALKYFHKRFPEHRAAEIRETLTQGL
EFCRRQQRADGSWEGSWGVCFTYGTWFGLEAFACMGQTYRDGTACAEVSRACDFLLSRQMADGGWGEDFESCEERRYLQSAQSQIHNTCWAMMG
LMAVRHPDIEAQERGVRCLLEKQLPNGDWPQENIAGVFNKSCAISYTSYRNIFPIWALGRFSQLYPERALAGHP
>Pteropus
ALNGMTFYTGLQAEDGHWAGDYGGPLFLLPGLLITCHVARIPLPAGYRREIVRYLRSVQLPDGGWGLHIEDKSTVFGTALNYVSLRILGVGPDD
PDLARARNVLHEKGGAVAIPSWGKFWLAVLNVYSWEGLHTVLPEMWLLPSWVPAHPSTIWCHCRMVYLPMSYCYATRLRAEEDPLVQSLRQELY
VEDYASIDWPAQRDNVAPGDLYTPHSWLLRAAYAILDLYERHHSTSLRQRAVQRLYEHIAADDRFSKCLSIGPISKTINMLVRWHMDGPASSVF
QEHISRIPDYLWLGLDGMKMQGTNGSQVWDTSFTIQALLEADAQHRPEFASCLQKAHEFLQMSQVPDNPPDYQKYYRQMCKGAFPFSTLDCGWI
VADCTAEALKSILLVQEKCPFVTTHTSKQQLFDAVAVLLSMRNADGGFATYETKRGGHLLELLNPSEVFGDIMIDYTYVECTSSVMQALTVFHT
QFPEHRPGEIRETLEQGLEYCRRKQRADGSWEGSWGVCFTYGTWFGLEAFACMGQTYHDGTAGMDVSRACNFLLSRQMADGGWGEDFESCEQRR
YVQSAQSQIHNTCWALMGLMAVRHPDVEALERGVRCLLRKQLPNGDWPQENVAGVFNKSCAISYSNYKNVFPIWALSRF
>Callorhinchus
LKGLRFYAPLQAEDGHWAGDYGGPLFLLPGLLIACHVTGAGLADSQRQEMVRYLRSVQLLDGGWGLHVEDSSTVLGTALNYTSLRLLGVGADDP
DLVRARNNLHTKGGAVGIPSWGKFWLAVLNVYSWEGLNSLFPEMWLLPRWMPAHPSTLWCHCRQVYLPMSYCFAVRLTAQEDTLIHSLRQELYV
QDYCSIDWPAQRNNVAPCDLYTPHSWLLNTAYAVLNVYEDHHSERLRCWAITEVYDHITADDRFTKCISIGPISKTINMLVRWSVDGPQSAAFQ
QHVERITDYLWLGVDGMKMQGTNGSQLWDTAFTVQAFLEAGAQNCPEFTTSLTLAHQYLRITQVQEAPPDYDKYYRQLNKGGFSFSTRDCGWVV
ADCTAEALKSLLLLQETCPFISDHVIDQQLHQAVHVLLTMRNTDGGFSTYEXKTGGRLLELLNPSEVFGDIMIDYTYVECTSAVIQALRHFQQK
HPHHRAFEIRDTLNKGLEFCRGCQRPDGSWEGSWGVCFTYGTWFGMEAFACMGHTYQRGAACREITRACEFLLSKQMVDGGWGEDFESCESRQY
VQSSSSQVHNTCWALLALMAARYPGTRAIEKGIKTLINKQQLNGDWPQENIAGVFNKSCAISYSAYRNIFPIWTLGRF
>Branchiostoma-LSS
MGDSVIRRRGGPYKTEPVTDLTRWRLSNVGGRQTWRYYGEGETPEREQNMVERHALGLDTSAFAPALPKAQTAREAVRNGMKFYSKLQTEDGHW
AGDYGGPLFLMPGLVIVCHVTKARLSEPQRLEMIRYLRSVQLPDGGWGLHIEDHSTVFGTALNYVTLRLLGVPADDKDVVRARNCLHAKGRLLP
SWTPAHPSTLWCHCRQVYLPMSYCYAVQLTAEVDSLVRELRTELYLEDYERINWPAQRNNVSACDVYTPHSWLLNLAYVGINLYESWHSSSLRQ
WAIDELYDHIKADDSFTKCISIGPISKVIQMLVRWHADGPTSPAFREHASRVADYLWLGLDGMKMQIPDNPPDYETYYRQMNKGGFPFSTRDCG
WIVTDCTAEGLKSMMMLQERCQGVQDPAPDHRLFDAVDVLLNMRNSDGGFATYETKRGGKLLELLNPSEVFGDIMIDYTYVECTSAVMQALKHF
QDQYPDHRAEEIRSWGVCFTYGTWFGLEAMACMGHRYDIGTATEAVTRACNFLKSHQMKAEAGGGWGENFESCEERKYVQSDTSQVVNTCWALL
GLMAVRYPDLSVLEPGVKFLMERQMENGDWPQENICGVFNKSCAISYTSYRNVFTIWALGRFCRLYPHSALTGGAAKS
>Saccoglossus-LSS
MACSGGRRRRGGPYKTNPATDLTRWRLKNVEGRQTWYYVEKNKKLLREQNMVELHSLGLDTSMHAPDLPRPKTVKDAAHNGMMFYSKLQSEDGH
WSGDYGGPLFLMPGLLIVCHITNVQLSDAQKAEMIRYLRSIHLDKVTLLFGTSLNYTAMRVLGVGSDDPDLVKARNILHSLGGAVYIPSWGKFW
LAVLNCYSWDGMNTLLPEMWLFPKWVPAHPSTLWCHCRQVYLPMSYCYATRLTAQEDDLIRSLRKSCCLFGDWISQRAVDKSCCLFGDRISQRV
VGKSCCLFGDRISQRVVGKSCCLFVLNVYESWHSKRLRQKAIQECYDHIQADDRFTKCISIGPVSKVINMLVRWHVDGPQSIVFKQHVDRIADY
LWLGLDGMKMNGTNGSQLWDTAFVVQAFIEAEAHKNTMFNDTLEHAHSFFKLTQIPENPADYEKYYRQMNKGGFPFSTRDCGWIVGDCTAEGLK
SVMMLQEKCANIKNPIEKEKLYEAVNVLLSLRNSDKGFATYETKRGGKLLELLNPSEVFGDIMIDYTYVECTSAVMQSLKHFQDQYPEYRKDEI
RTTLDDGLAYIKDKQRSDGSWEGSWGVCFTYGAWFGLEAYSCMGCRYDLGTATNEVKLACEFLCKKQMSDGGWGENFESCEKRQYIESDNSQIV
NTAWALLGLMAVRYPDISVLEKGVQLIMSRQLPNGDWPQENISGVFNKSCAISYTSYRNVFPIWTLGRFARLYPDSNLVTPSFNGR
>Capitella
AYNGLQFYSKLQAEDGHWAGDYGGPLFLTPGLVIVCYITNTPFTDEQTKEMIRYLKSVECPDGGWGLHTEGPPTVFGCALNYICLRLLGLPPDD
PVLIRARALLHKLGGATGIPSWGKFWLAVLNVYKWEGIHNLFPEIWTLPEWLPIHPSHMWCHCRQVYLPMAYCYGARITAKETDLIRELREEIY
VESYDSIEWSKQRSNVASADLYSPHSWILDTAYYFLDWYEMYHSKSWRQKSLDKIYEHICADDEFTKCISIGPISKTIQMLVRWHRDGPSHPVF
LLHQDRVKDYLWLGLDGMKMTGTNGSQLWDTAFAIQMYMGCGANKRKEFRDTLDNAHAWLKLTQIPENPPEYSKFYRQMSRGGFPFSTKDCGWI
VADCTADGLKAVLALQENCDFLSQEIPEEQLNQAIDVLLSMSNLLYGYSWVGMGYATYETKRGGKMLEMLNPSEVFGDIMIDYPYVELTSSVIQ
ALKKFSNKHAYRNMEILWTVHLGLQYIKREQRKDGSWYGSWGVCFTYGAWFALEAFAAMSFRYDYNNVTEEMKKGCQWLVDHRLHDGGWGENFE
ACEIKEYVPAKKSQTVNTAWALMALMAVRYPDMEVLEGGIKILLKRQYSNGDFPQENIMGVFNKSCAIHYESYRNIFPVWALGRF
>Lingula
AYNGIKFYSKLQAEDGHWAGDYGGPLFLMPGLVIVCHITRTPFTPEQKLEMIRYLRSVQCPDGGWGLHIEGPPTVFGCALNYVVMRLLGVGPED
RDLVKARRLLHRLGGAAAIPSWGKFWLAVMNLYSWEGMHSLFPEMWLFPTWIPIHPSKLWCHCRQVYLPMAYCYGVRLSADEDDLIRELRKELY
TESYESIQWSSQRDNVSSADLYTPHSWFLDLAYYFLDFYESHHNSSWRQASLHECYTHICADDEFTKGISIGPISKVIQMLIRWYVDGPSSAAF
KQHQDRVQDYLWLGLDGMKMTGTNGSQLWDTAFAVQAFMEAEAFEYRDFQNGLNHAHKFLKNTQIEENPPEYQKIYRHQNKGGFPFSTRDCGWI
VTDCTAEGLKGVMLLQEKCDFLTQTVSEERLYQAVDVLLSMKNADGGFATYETTRGGKLLELLNPSEVFGDIMIDYTYVECTSASMQALEHFTS
QYPAYRTEEIRQTLESGLQYVKSKQRNDGSWEGSWGVCFTYGTWFGLEAFAAMGYRYDTENVPPEVRKACNFLISKQMADGGWGENFESCELRE
YVQSDTSQVVNTCWALLGLMAVRYPDVALIEHGIRVIMQRQLDNGDWPQENISGVFNKSCAISYTSYRNVFPIWTLGRF
>Aplysia
AKNGMTFYSKMQAEDGHWAGDYGGPLFLMPGLIIVCYITKTPFTEAQRVEMIRYLKSVTCPDGGWGLHIEGPPTVFGCALNYAALRMLGLPADD
PDLVRARDLLHKLGGASAIPSWGKFWLAVLNCYSWEGLNSLFPEMWILPTWLPFHPSKLWCHCRQVYLPMAYCFGRRISAPEDDLIRSLRKELF
VEDFDKINWPAQRNNVSSADLYSPHAIILDIAYTILNIYEKVHLGFLRKWALADMYDHISADDRFTKCISIGPISKVINMVIRYHEEGPTSKAF

MLHQDRVQDYLWIGLDGMKMTGTNGSQSWDTSFAATAFLEAGAYKYEELKKCLTACHDFLRITQVPENPPDYKKYYRQRNEGAFPFSTRDCGWI
VSDCTAEGLKAVIKLEEKCQITEAVPKQRIYRGIDVLLEMRCDDNGWATYEDKRGGVLLEVLNASEVFGDIMIDYTYVECTSACMQCMTTFTKA
HPEYRKDEIQAALTGGLDYVRGKQRPDGSWEGSWAVCFTYGAWFALEAYACMGHVYGGGEGQDSGKVPEEVEKGCQYLLSIQMEDGGWGENFES
CEKRQYVPSETSQIINTCWALLALMAVKYPDVEVLEKGIKLIMSRQLENGDWPQENISGVFNKSCAISYTSYRNVFPIWTLGRF
>PpLSS
MSGRRNRGGPHKTEPVTDLTRWRLSNVDGRQTWRYIEEGEPIEREQNLVEKFSLGLDISKEAPPFPKPTTPQEAATNGMVFYSRLQTEDGHWSG
DYGGPLFLMPGLLIVCYITKTELQDAVKKEMVRYLRSVQCQDGGWGLHIEGPATVFGCATNYVAMRLLGVAADDPDLIKCRKLLHSLGGAAAIP
SWGKFWLSVLNVYSWDGMHTLFPELWLFPTWIPAHPSTIWCHCRQVYLPMSYCYGIKYQAEEDELIRELRKELYTQDYHTIDWPAQRDNIAKVD
LYTPHSWLYNLAFGFLDVYEPYHSTAFRQRALDECLDHIRADDRFTKCISIGPISKVINMLVRWITEGADSEIFKQHVERIPDYLWIGLDGMKM
QGTNGSQLWDTAFTAQAFLEAGAATNKELHECLQRTHDFLKLTQIPDNPPNYQ
>PmLSS
MSGRRNRGGPHKTEPVTDLTRWRLSNVDGRQTWRYIEEGEPIEREQNLVEKFSLGLDISKEAPPFPKPTTPQEAATNGMVFYSRLQTEDGHWSG
DYGGPLFLMPGLLIVCYITKTELQDAVKKEMVRYLRSVQCQDGGWGLHIEGPPTVFGCATNYVAMRLLGVAADDPDLIKCRKLLHSLGGAAAIP
SWGKFWLSVLNVYSWDGMHTLFPELWLFPTWIPAHPSTIWCHCRQVYLPMSYCYGIKYQAEEDDLIRELRKELYTQDYHTIDWPAQRDNIAKVD
LYTPHSWLYNLAFGFLDVYEPYHSTAFRQRALDECLDHIRADDRFTKCISIGPISKVINMLVRWITEGADSEVFKQHVERIPDYLWIGLDGMKM
QGTNGSQLWDTAFAAQAFLEAGAATNKELHECLQRTHDFLKLTQIPDNPPNYQKYYRQMNKGGYPFSTRDCGWIVADCTAEGLKAALMLETMCP
TVKDHIEKDRHYEAVDVILNMRNKDGGFATYETMRGGIILEKLNPSEVFGDIMIDYTYVELTSAVMQCLKKFSELYPEYRKDEMGYSYEKRTNT
MQIRRACDFLVSKQMEDGGWGEEFESCEERRYIQSKTSQVVNTAWAVLALMAVRFPQREVIDRGIKVIMDRQLPNGDWAQENIKGVFNKSCAIS
YTSYRNVFPIWALGRYARIYGNSS
>ApLSS
MSSRRNRGGPHKTQPVTDLTRWRLSNVDGRQCWRYIEEGQPIEREQNFVEKFSLGLDISKEAPPLPKPTTPQEVATNGMLFYSKLQTEDGHWSG
DYGGPLFLMPGLLIVCYITKTELQDAVKKEMVRYLRSVQCPDGGWGLHIEGPATVFGCATNYVAMRLLGVSADDPDLVKCRKLLHSLGGAAAIP
SWGKFWLSVLNVYSWEGMHTLFPELWLFPTWIPAHPSTIWCHCRQVYLPMSYCYGVKYQAEEDDLIRELRKELYIQDYNTIDWPAQRDNIAKID
LYTPHSWLYNLAFGFLDVYEPYHSTYFRQMALDECLDHIRADDQFTKCISIGPISKVINMLVRWLTDGPDSQVFKQHVERIPDYLWIGLDGMKM
QGTNGSQLWDTAFAAQAFLEAGAAKNKELHDCLQHTHDFLKLTQIPDNPPNYQKYYRQMNKGGYPFSTRDCGWIVADCTAEGLKAALMLETMCP
SVRDHIEKERHCQAVDVILNMRNKDGGFATYETMRGGVILEKLNPSEVFGDIMIDYTYVELTSAVMQTLKKFNTLYPDYRKEDIRSTLDKGLKY
IAMKQRIDGSWEGSWGVCFTYAVWFGLEAFACMGYSYETGAVTMQIRRACTFLKSKQMEDGGWGEEFESCEERRYVQSKTSQVVNTAWAVLALM
AVRFPERDVIDRGIKVIMDRQLPNGDWAQENIKGVFNKSCAISYTSYRNIFPIWALGRYARIYGNST
>MgLSS
HPSTIWCHCRQVYLPMSYCYGIKFQAEPDDLIMDLRMELYVEDYYTINWPAQRSNIAKVDLYTPHSWLYNLAFGCLDIYEKYHFADWRQRALDE
CLDHIKADDKFTKCISIGPISKVINMLVRWITDGPDSEIFKQHVERIPDYLWIGLDGMKMQGTNGSQLWDTAFAAQAFLEGGASKIESLHQSLQ
RTHDFFKLTQIPENPPNYQKYYRQMNKGGYPFSTRDCGWIVADCTAEGLKSALMIEQLCPFLKDHITQERHQEAVDVMLNMRNSDGGFATYETM
RGGVILEKLNPSEVFGDIMIDYTYVELTSAVMQSLKKFSDIYPDYRQAEIRATLDRGLLYIARKQRTDGSWEGSWGVCFTYAAWFGLEAYACMG
YSYETSTASSEVKRACAFLLSRQMEDGGWGEEFESCEERRYIQSKTSQVVNTAWAVLALMAVRFPEREVIDRGIRVIRDRQLSNGDWAQENIKG
VFNKSCAISYTSYRNLFPIWALGRYARIYGDDA
>LspLSS
MSGRRNRGGPHKTEPATDLTKWRLSNVDGRQTWRFIEEGEAVDREQNFVEKFSLGLDISEEAPPLAKPATAQEAATNGMLFYSKLQTEDGHWSG
DYGGPLFLMPGLLTVCYITKTELPDAVKKEMVRYLRSVQCQDGGWGLHVEGPPTVFGCATNYVAMRLLGIGPEDPDLMKCRKLLHSLGGAAAIP
SWGKFWLSVLNVYEWDGMHTLFPEIWNFPTFFPAHPSTIWCHCRQVYLPMSYCYGIKFQAEPDDLIMDLRMELYVEDYYTIDWPAQRSNIAKVD
LYTPHSWLYSLTFGCLDKYEKYHIAKGRQRALEECLDHIKADDEFTQCISIGPISKVINMLVRWITDGPDSEIFKQHVERISDYLWIGLDGMKM
QGTNGSQLWDTAFAAQAFLEGGAAKNESLHQCLQRTHDFFKLTQIPENPPNYQKYYRQMNKGGYPFSTRDCGWIVADCTAEGLKSALMIEQHCP
FLKDHITQERHQEAVDVMLNMRNSDGGFATYETMRGGIILEKLNPSEVFGDIMIDYTYVELTSAVMQSLKKFSDIYPEYRQAEIRATLDRGLLY
IALKQRRDGSWEGSWGVCFTYAAWFGLEAYACMGYSYETSTASSEVKRACAFLLSRQMEDGGWGEEFESCEERRYIQSKTSQVVNTAWSVLALM
AVRFPERDVIDRGIQVIRDRQLPNGDWAQENIKGVFNKSCAISYTSYRNLFPIWALGRYAQIYGDHA
>AfLSS
MSGRRNRGGPHKTEPATDLTRWRLSNVDGRQTWRFIEEGEPVDREQNFVEKFSLGLDISEEAPPLAKPATAQEAATNGMLFYSKLQTEDGHWSG
DYGGPLFLMPGLLIVCYITKTELPDAVKKEMVRYLRSVQCQDGGWGLHIEGPPTVFGCATNYVAMRLLGIGPEDPDLMKCRKLLHSLGGAAAIP
SWGKFWLSVLNVYQWDGMHTLFPELWNFPTYTPAHPSTIWCHCRQVYLPMSYCYGIKFQAEPDDLIMDLRMELYVEDYYTINWPAQRSNIAQVD
LYTPHSWLYNLAFGCLDIYETYHSTKWRQSALDECLDHIKADDEFTQCISIGPISKVINMLVRWMTDGPDSAIFKQHVERIPDYLWIGLDGMKM
QGTNGSQLWDTAFAAQAFLEGGAAKNESLHECLQRTHDFLKLTQIPENPPNYQKYYRQMNKGGYPFSTRDCGWIVADCTAEGLKSALMIEQRCP
FLKDHITQERHQEAVDIMLNMRNSDGGFATYETMRGGVILEKLNPSEVFGDIMIDYTYVELTSAVLQSLKKFSDIYPDYRQAEIRATLDRGLLY
IADKQKRDGSWEGSWGVCFTYAAWFGLEAYACMGYSYKTSTASSEVKRACAFLLSRQMEDGGWGEEFESCEERRYIQSKTSQVVNTAWSVLALM
AVRFPEREVIDRGIRVIRDRQLPNGDWAQESIKGVFNKSCAISYTSYRNLFPIWALGRYARIYGDDA
>ArLSS
MSGRRNRGGPHKTEPATDLTRWRLSNVDGRQTWRFIEEGEPVDREQNFVEKFSLGLDISEEAPPLAKPATAHKAATNGMLFYSKLQTEDGHWSG
DYGGPLFLMPGLLIVCYITKTELPDAVKKEMVRYLRSVQCQDGGWGLHVEGPPTVFGCATNYVAMRLLGIGPEDPDLMKCRKLLHSLGGAAAIP
SWGKFWLSVLNVYQWDGMHTLFPELWNFPTYTPAHPSTIWCHCRQVYLPMSYCYGIKFQAEPDDLIMDLRMELYVEDYYTINWPAQRSNIAQVD
LYTPHSWLYNLAFGCLDIYEKYHSTKWRQSALDECLDHIKADDEFTQCISIGPISKVINMLVRWMTDGPDSAIFKQHVERIPDYLWIGLDGMKM
QGTNGSQLWDTAFAAQAFLEGGAAKNESLHECLQRTHDFFKLTQIPENPPNYQKYYRQMNKGGYPFSTRDCGWIVADCTAEGLKSALMIEQQCP
FLKDHITQERHQEAVDVMLNMRNSDGGFATYETMRGGVILEKLNPSEVFGDIMIDYTYVELTSAVLQSLKKFSDIYPDYRQAEIRATLDRGLLY
IADKQKRDGSWEGSWGVCFTYAAWFGLEAYACMGYSYKTSTASSEVKRACAFLLSRQMEDGGWGEEFESCEERRYIQSKTSQVVNTAWSVLALM
AVRFPEREVIDRGIRVIRDRQLPNGDWAQESIKGVFNKSCAISYTSYRNLFPIWALGRYARIYGDDA
>AaLSS
MLVRWMTDGPDSAIFKQHVERIPDYLWIGLDGMKMQGTNGSQLWDTAFAAQAFLEGGAAKNESLHECLQRTHDFFKLTQIPENPPNYQKYYRQM
NKGGYPFSTRDCGWIVADCTAEGLKSALMIEQRCPFLKDHISQERHQEAVDVMLNMRNSDGGFATYETMRGGVILEKLNPSEVFGDIMIDYTYV
ELTSAVMQSLKKFSDIYPDYRQAEIRATLDRGLLYIADKQKRDGSWEGSWGVCFTYAAWFGLEAYACMGYSYETSTASSEVKRACAFLLSRQME
DGGWGEEFESCEERRYIQSKTSQVVNTAWSVLALMAVRFPEREVIDRGIRVIRDRQLPNGDWAQESIKGVFNKSCAISYTSYRNLFPIWALGRY
ARIYCDDA
>HspLSS
MSGRRNRGGPHKTEPVTDLTRWRLSNVDGRQTWRYIEEGETIDREQNLVEKFSLGLDISKEAPPFPKPTSAQEAATNGSIFYSRLQTEDGHWSG
DYGGPLFLMPGLLIVCYITKTELPEAFRKEMVRYLRSVQCQDGGWGLHIEGPPTVFGCATNYVAMRLLDVSPDDPDLMKCRKLLHSLGGAVAIP
SWGKFWLSVLNVYSWDGMHTLFPELWLFPQWIPAHPSTIWCHCRQVYLPMSYCYGRKFQAEEDDLIRELRKELYVEDYSTINWSKQRNNIAKVD
LYTPHSWLYNVVFGTLDVYESFHFSAFRQQALDECLDHIRADDQFTKCISIGPISKVINMLVRWMTDGPDSEVFKKHVQRIPDYLWIGLDGMKM
QGTNGSQLWDTAFAAQALLDAGASTNADLHDCLQRTHDFLKLSQIPENPPNYQKYYRQMNKGGYPFSTRDCGWIVADCTAEGLKAALMLETQGT
AVKEPISKDRHCQAVDVMLNMRNPDGGFATYETMRGGMILEQMNPSEVFGDIMIDYTYVELTSAVMQSLKKFSELYPEYRKDDVRSTLDNGLSY
IAKKQKTDGSWEGSWGVCFTYATWFGLEAYACMGHTYETTTATPEIKRACAFLLSRQMEDGGWGEEFESCEVRRYVQSKTSQVVNTAWAVMGLM
AVRFPEKDVIDRGIKVIMDRQLPNGDWAQENIKGVFNKSCAISYTNYRNVFPIWALGRYARLYGNKE

```
>EsLSS
MSGRRNRGGPHKTEPATDLTRWRLSNVEGRQTWRYIEDGETIDREQNLVEKFSLGLDISQEAPPFAKPTSAQEAATNGSIFYSKLQTEDGHWSG
DYGGPLFLMPGLLIVCYITKTELPEAFRKEMVRYLRSVQCQDGGWGLHIEGPPTVFGCATNYVAMRLLDVPPDDPDMVKCRKLLHSLGGAVAIP
SWGKFWLSVLNVYSWDGMHTLFPELWLFPQWIPAHPSTLWCHCRQVYLPMSYCYGRKFQAEEDDLIRELRKELYVEDYSTIDWPKQRNNIAKVD
LYTPHSWLYNVLFGILDVYESFHFPAFRQQALDECLDHIRADDQFTNCISIGPISKVINMLIRWMTDGPDSEVFKQHVQRIPDYLWIGLDGMKM
QGTNGSQLWDTAFAAQALLEAGASTNPDLQDCLQRTHDFLKLTQIPENPPNYQKYYRQMNKGGYPFSTRDCGWIVADCTAEGLKAALMLETMGT
VIKEPISNDRHCQAVDVMLNMRNNDGGFATYETTRGGMLLEQLNPSEVFGDIMIDYTYVELTSAVMQSLKKFSDLYPDYRKDDIRSTLDNGLSY
IAKKQKADGSWEGSWGVCFTYATWFGLEAYACMGHSYETMTATPEVKRACAFVLSRQMEDGGWGEEFESCEERRYIQSKTSQVVNTAWAVMGLM
AVRFPDKDVIDRGIKVIMDRQLPNGDWAQENIKGVFNKSCAISYTSYRNVFPIWALGRYARLYGNKE
>SpLSS
MSEKKNRGGPYKTTPATDLSRWRLTNTNGRQTWQYYPEGEEPGRPQNFVEKFSLGLDIDDEAPPLPRAKTAEEAAKNGMKFYSKLQTEDGHWAG
DYGGPLFLLPGLVIVCFITGVVLPDASKKEMVRYLRSVQCPDGGWGLHIEDHATVFGTAMNYVTMRLLGVSKDDEDLKKARKLLLEMGGAESIP
SWGKFWLCVLNLYKWEGMHCLFPEIWLFPTWLPAHPSTIWCHCRQVYLPMGFFYGIKFQAEENQLTRELRKEIFKADYSSIHWPSQRDNISKFD
LYTPHSWLYNIAMAILDLYEKFHSTWLKKKALDHCYEHIKADDEFTNGISVGPISKVIQMLARWHIDGADSEAFKMHKDRLPDYLWIGLDGMKM
QGTNGSQLWDTAFVIQAFLEAGGEGEFKDTLSKAHDFLKNTQIPENPPNYQKYYRQMSKGGYPFSTKDCGWIVSDCTAEGLKSAMLLEEKCPFI
SDHIGKERHCQAIDVLIDMRNPDGGFATYETTRGGYILENLNPSEVFGDIMIDYTYVECTVAVLLALHHFHTQYPEYRTDEIRSVMDQALDYIK
GKQQPDGSWEGSWGVCFTYAAWFGLEGFGSMGLRYDKDLASSEVKKACQFLVSKQMEDGGWGEKFESCEERCYVQCETSQVINTCWALLGLMAV
RYPDRRVMDRGIQLIMSRQSENGDWPQENINGVFNKSCAISYTNYRNIFPIWTLGRYAKLYPNDASPLEDKSDSNSSDWEKL
>SgLSS
MSDKKNRGGPYRTTPATDLSRWRLTNVNGRQTWRYYPEGDEPGRPQNFVEKFSLGLDINGEAPPLPKARNAKEAAKNGMEFYSKLQTEDGHWSG
DYGGPLFLLPGLVIVCFITGIALPDASKKEMVRYLRSVQCPDGGWGLHIEDHPTVFGTAMNYVTMRLLGVSKDDTDLKKARKLLMEMGGAESIP
SWGKFWLCVLNLYKWEGMHCLFPEIWLFPEWFPAHPSSIWCHCRQVYLPMGFFYGIKFQAEENDLIRELRKEIFKEDFSSIHWPSQRNNISKFD
LYTPHSWMYNIAMAILDFYETFHSTWLRKKALDHCYDHIKADDEFTKGISIGPISKVIQMLVRWHIDGPDSEAFKMHKDRIPDYLWIGLDGMKM
QGTNGSQLWDTAFVIQAFLEAGAVPDFKDTLINAHDFLKNTQIPDNPPNYQKYYRQMNKGGYPFSTKDCGWIVSDCTAE
>EpLSS
MSYKKNFGGPYKTDPATDLTRWRLTNVNGRQTWRYIPEGEEPDRPQNFVEKFALGLSIDELTPPLSKAKTAKEAAKNGMRFYSKLQTEDGHWSG
DYGGPLFLLPGLIIVCYITGVVLPEASKKEMIRYLRSVQCPDGGWGLHIEDHATVFGTAMNYVTMRLLGVSKDDKDLKKARKLLMEMGGAESIP
SWGKFWLCVLNLYKWEGMHCLFPEIWLFPSWMPAHPSSIWCHCRQVYLPMGYFYGAKFQAQEDGLIRELRNEIFKEDFATINWPAQRDNISKYD
LYTPHSWLYTIAMAILDFYEKFHSTWLRQKALDFCYDHIKADDTFTKGISIGPISKVIQMLVRWHVDGPESDAFKMHRDRIPDYLWIGLDGMKM
QGTNGSQLWDTAFAVQAFIEAGANTEEEFRDTLRNAHGFLRNTQIPDNPPDYQKYYRQMNKGGYPFSTKDCGWIVADCTAEGLKSAMLLEEKCP
FVTDLIGKERHCLAVDVLIDMRNPDGGYATYETTRGGYILEKLNPSEVFGDIMIDYTYVECTAAAMLALKHFQDQHPEYRRDEINSVLDTGLDF
IKGIQRPDGSWEGSWGVCFTYGAWFGLEGLGCMGMRYDRDTATPEVKKACAFLVSKQMADGGWGEKFESCETRTYVQSETSQGVQTCWALMALM
AVRYPDRTVIDRGIKLIMDTQLENGDWPQANIMGVFNKSCAISYTSYRNVFPIWCLGRYAKLYPTPDSTPSASTASTPLSDRSDSNEEGWEKL
>Amphimedon
GLDYYTRLQSDDGHWSGDYGGPLFLLPGLIIVYHVTGLQFEDHQRLEMIRYLRNVQNPDGGWGLHIAGKSTVFGTALNYVSLRLLGVGPDDNEL
IKARELLHQMGGAVCIPSWGKFWLSVLNVYDWSGVHTLLPELWILPQCVPVHPSKMWCHCRQVYLPMGFVYSKRIKAKPTKIILELRQEIYVQS
YESIDWPSQRNNVSSTDLYTPHSLALDWSYLLLDAYESHHISRLRAWADEEILRQIKADDSFTNCISIGPISKVIQMLVRWFVDGPDSTSFKEH
LSRVPDYLWMGRDGIKMQGTNGSQLWDTAFAVQAILEASSFDTNKHSHSLSMAHSFLLSTQVPDNPPDYKKYYRQMSKGGFPFSTLECGWIVSD
CTAEGLKSLMLLEEECRSFISNGVTVSKMEDTINVLLNMQNSNGGFSSYETNRGGAILELLNPSEVFGDIMVDYTYVECTSASLQAINHFNKRY
PQHRPKEIQECLSRGLEYILNIQRPDGSWEGSWGVCFTYGTWFGLEALASMNRRYDYGTAGSEVKRACQFLVDHQMSDGGWGEDFESCEKRVYV
QSEESQVVNTCWALLGLMAVRYPHTDVIKNGIKLIVSRQLDSGEWKQEGIKGVFNKTCAITYTSYKNVFPLWTLGRF
>AjPS-b
MPGLRRIDPVTDLTRWRLTSIDGRRRWHFISIDGTLEREQNVLEKYSLGLDCSDEAKKLPDAESAAEAAINGMTFFSLLQADDGHWPNDYSGPL
FLMPGLFIVLYITKTKLPDAFVKESIRYLRSVQLADGGWGLHTEDNATVFGTALNYVVMRLLGVSPEDQDLVKARKLLHHHGGAATIPSWGKFW
LCILNCYKWEGMHTLFPELWLLPSWLPAHPSTLWVHCRIVYLGMSYLYGKRYYAPEDSLILSLRKELFVEDFETIDWPAQRENIAKIDIYTPHS
WLYSMVFGMLDTYEPFHSSWYRESALKVCLDHIKQDDIMTNFISIGPISKMINMLIRWLEDGPDSKAFLQHVDRVYDYIWMGRDGVNMQGTNGT
QIWDVSYAAMAMLEAGAGTKPEFQEALRKAYSYLEVSQMVESSPKCVQYYRQYNKGGWPLTTRDHGLIVSDTTAEALKAAMLLEEKCPFIGSEA
KISRERLHEAVDLLLDMVNPNGGYSSYETLRGGEKLLELLNPSEVFGDIMVDYTYTECTSSVLQALRHFVDYDPKYRTDEIWNVLKNGLGYIKQ
NQLPDGSFEGSWGVCFTYGTWFALEAFACMGQNYNDNTASMQVKKACSFLVSRQMEDGGWGEKFASCSERRYVQSEKSLVVNTSWALLGLMAVR
YPDEEVLSRGVKVLLDRQIDDGDWPQESICGVFNKSCAIGYTAFKNIFPIWALGRHAQLYPCSSRTFSAESVSDKDWEKIPHV*
>AjPS-a
MPGSRRIDPVTDLTRWRLTSIDGRRRWHFISIDGTLEREQNVLEKYSLGLDCSDEANKLPDAESAAEAAINGMTFFSLLQADDGHWPNDYSGPL
FLMPGLFIVLYITKTKLPDAFVKESIRYLRSVQLADGGWGLHTEDNATVFGTALNYVVMRLLGVSPEDQDLVKARKLLHHHGGAATIPSWGKFW
LCILNCYKWEGMHTLFPELWLLPSWLPAHPSTLWVHCRIVYLGMSYLYGKRYYAPEDSLILSLRKELFVEDFETIDWPAQRENIAKIDIYTPHS
WLYSMVFGMLDTYEPFHSSWYRESALKVCLDHIKQDDIMTNFISIGPISKMINMLIRWLEDGPDSKAFLQHVDRVYDYIWMGRDGANMQGTNGT
QIWDVSYAAMAMLEAGAGTKPEFQEALRKAYSYLEVSQMVESSPKCVQYYRQYNKGGWPLTTRDHGLIVSDTTAEALKAAMLLEEKCPFIGSEA
KISRERLHEAVDLLLDMVNPNGGYSSYETLRGGEKLLELLNPSEVFGDIMVDYTYTECTSSVLQALRHFVDYDPKYRTDEIWNVLKNGLGYIKQ
NQLPDGSFEGSWGVCFTYGTWFALEAFACMGQNYNDNTASMQVKKACAFLVSRQMEDGGWGEKFASCSERRYVQSEKSLVVNTSWALLGLMAVR
YPDEEVLSRGVKVLLDRQIDDGDWPQESICGVFNKSCAIGYTAFKNIFPIWALGRHAQLYPSSPRTFSAESVSDKDWEKIPHV*
>PpPS
MSGSRRINPVTDLTRWRLTSIDGRRRWHFISIDGTLEREQNVIEKYSLGLDYSDEANKLPDAESAAEAAINGMKFFSLLQCDDGHWPNDYSGPL
FLMPGLIIVLYITKTKLPDAFVKESIRYLRSVQLADGGWGLHTEDNATVFGTALNYVVMRLLGVSPEDQDLVKARKLLHHHGGAATIPSWGKFW
LCILNCYKWEGMHTLFPELWLLPSWLPAHPSTLWVHCRIVYLGMSYLYGKRYYAPEDSLILSLRKGLFVEDFETIDWPAQRENIAKIDIYTPHS
WLYSMVFGMLDTYEPFHSSWYRESALKVCLDHIKQDDIMTNFISIGPISKMINMLIRWLEDGPDSKAFLQHVDRVYDYIWMGRDGVNMQGTNGT
QIWDVSYAAMAMLEAGAGTKPEFQEALRKAYSYLEVSQMVESSPKCVQYYRQYNKGGWPLTTRDHGLIVSDTTAEALKAAMLIEEKCPFIGSEA
KISRERLHEAVDLLLDMVNPNGGYSSYETLRGGEKLLELLNPSEVFGDIMVDYTYTECTSSVLQALRHFVDYDPNYRTDEIWNVLKNGLGYIKQ
NQLPDGSFEGSWGVCFTYGTWFALEAFACMGQNYNDNTASMQVKKACSFLVSRQMEDGGWGEKFASCSERRYVQSEKSLVVNTSWALLGLMAVR
YPDEEVLSRGVKVLLDRQIDDGDWPQESICGVFNKSCAIGYTAFKNIFPIWALGRHAQLYPSSFRTFSAESVSDKDWEKIPHV
>ScPS
TNGMTFFSLLQADDGHWPNDYSGPLFLMPGLFIVLYITKTKVPEAFMKESVRYLRTLQLKEGGWGLHTEDKGTVFGTALNYVTMRLLGVGPEDE
DLQRARKFLHYHGGAASIPSWGKFWLCILNCYRWEGMHTLFPELWLLPTWVPAHPSSLWVHCRAVYIGMAYLYGKHFYAPEDDLIISLRKELFV
EDFDHINWPSQRDNIAEIDLYTSHSWLHTLAFGLLDIYESIHHKWYREKALEICLDHIKQDDIMTNFISIGPVSKMMNMLIRWLEDGPESKAYQ
QHIDRVYDYIWMGRDGVNMQGTNGTQVWDVSFAVMAMLECGANLRPQFQNVLQKAYLYLEVSQMVENSPKCLQYHRQFNKGGWPLTTRDHGLIV
SDTTAEALKAALLIEEKCPFIKSSARISRERIHDAVDLLLAMANPNGGFATYEILRGSDKVLELLNPSEVFGDIMVDYTYTECTSSVMQALRHF
VNYDPSYRQGEIWDVLRNGLQYIKQNQLPDGSFEGSWGVCFTYGTWFALEAFACMGQNYDDNTATLEVKKACSFLVSRQMEDGGWGEKFASCSE
RRYVQSEKSLVVNTAWALLGLMAVRYPDETILSRGVKVLRDRQTPDGDWPQETISGVFNRSCAISYTSFKNIFPIWALGRYAQLYPSAT
>AjLDS-b
```

MQSDNDHKPRSERTISEYSDLTRWRLSCTEGKRIWHYVTEDETPERPQNMVEKYSLGLDYSNEAEKLPRAQNPKEAAENGIKFFSLMQAEDGHW
PNDYSGPLFLMPGLIIVLYITKTEFPEAFKQEFVRYLRRVQAKDGGWSLHIEGDATVFGTALNYISMRLLGVSPEDGDMKRARKVLHHHGGASA
IPSWGKFWLCILNCYKWEGMHTMFPELWLMPSWIPAHPSTLWIHCRMVYIGMAFLYGKRYYAQEDELIMDLRKELFMEDFDQIDWPSQRDNIAK
IDLYTPHSWLFNIAFGILDKYEPFRLTRFRKQALDVCLDHIKQDDLMTSFISIGPISKMINMLIRWLEDGPESEAFKKHVERVYDYVWMGLDGT
NVQGTNGNQVWDTSFAAMAMLDVGAQDDPQFHEVLCKTYSYLEISQVIESSPDCVKYYRQYNKGGWAQTMREHGLVVSDTSAEALKAVLLMNDK
CPFITERVSKRRLRDAVDMLLTMVNPNGGFSSYENLRGGTILELLNPSEVFGDIMVDYTYTECTSSVLQALRHFVDSDPDYRQDEIWAVLRNAM
EYIRSNQLPDGSFEGSWGVCFTYGTWFALEAFACMGKNYQDNTASIDVKKACSFLVSRQMEDGGWGEKFASCSERRYVQSEKSLVVNTSWALLG
LMAVRYPDQAVLERGIQVLRDRQHEDGDWPQETISGVFNRSCAISYPAFKNIFPIWALGRYSQLYTSSKENQDSWVRDDDWEKLSNNSQIAKLV
F*
>AjLDS-a
MQSDNDHKPRSERTISEYSDLTRWRLSCTEGKRIWHYVTEDETPERPQNMVEKYSLGLDYSNEAEKLPRAQNPKEAAENGIKFFSLMQAEDGHW
PNDYSGPLFLMPGLIIVLYITKTEFPEAFKQEFVRYLRRVQAKDGGWSLHIEGDATVFGTALNYISMRLLGVSPEDGDMKRARKVLHHHGGASA
IPSWGKFWLCILNCYKWEGMHTMFPELWLMPSWIPAHPSTLWIHCRMVYIGMAFLYGKRYYAQEDELIMDLRKELFIEDFDQIDWPSQRDNIAK
IDLYTPHSWLFNIAFGILDKYEPFRLTRFRKQALDVCLDHIKQDDLMTSFISIGPISKMINMLIRWLEDGPESEAFKKHVERVYDYVWMGLDGT
NVQGTNGNQVWDTSFAAMAMLDVGAQDDPQFHEVFCKTYSYLEISQVIESSPDCVKYYRQYNKGGWAQTMREHGLVVSDTSAEALKAVLLMNDK
CPFITERVSKRRLRDAVDMLLTMVNPNGGFSSYENLRGGTILELLNPSEVFGDIMVDYTYTECTSSVLQALRHFVDSDPDYRQDEIWAVLRNAM
EYIRSNQLPDGSFEGSWGVCFTYGTWFALEAFACMGKNYQDNTASIDVKKACAFLVSRQMEDGGWGEKFASCSERRYVQSEKSLVVNTSWALLG
LMAVRYPDQAVLERGIQVLRDRQHEDGDWPQETISGVFNRSCAISYPAFKNIFPIWALGRYSQLYTSSKENQDSWVRDDDWEKLSNNWQIAKLV
F*
>PpLDS
MQSDSDQKPRSERPNKEYSDLTRWRLSCTEGKRIWHYVTEDETPERPQNMVEKYSLGLDYSNEAEKLPRAQNPKEAAENGIKFFSLMQAEDGHW
PNDYSGPLFLMPGLIIVHYVTKTKFPEAFKQEFIRYLRRVQAKDGGWSLHIEGDATVFGTALNYISMRLLGVSPEDGDLKRARKVLHHHGGAAA
IPSWGKFWLCILNCYKWEGMHTMFPELWLMPSWIPAHPSTLWIHCRMVYIGMAFLYGKRYYAQEDELIMELRKELFIEDFDQIDWSSQRDNIAE
IDLYTPHSWLFNITFGILDKYEPFRLTRFREQALDVCLDHIKQDDLMASFISIGPISKMINMLIRWLEDGPESEAFKKHVERVYDYVWMGLDGT
NVQGTNGNQVWDTSFAAMAMLDVGAQDDPQFHEVLSKTYSYLEISQVIESSPDCVKYYRQYNKGGWAQTMREHGLVVSDTSAEALKAVLLMNDK
CPFITERVSKRRLRDAVDMLLTIVNPNGGFSSYENLRGGTILELLNPSEVFGDIMVDYTYTECTSSVLQALRHFVDSDPDYRQDEIWAVLRNAM
EYIRSNQLPDGSFEGSWGVCFTYGTWFALEAFACMGKNYQDNTASIDVKKACSFLVSRQMEDGGWGEKFASCSERRYVQSEKSLVVNTSWALLG
LMAVRYPDQSVLERAIQVLRDRQHEDGDWPQETISGVFNRSCAISYPAFKNIFPIWALGRYSQLYTSSKETQDSWVQNDDWEKLSNNWQLAKLV
F
>ScLDS
TNGMTFFSLLQADDGHWPNDYSGPLFLMPGLFIVLYITKTKVPEAFMKESVRYLRTLQLKEGGWGLHTEDKGTVFGTALNYVTMRLLGVGPEDE
DLQRARKFLHYHGGAASIPSWGKFWLCILNCYRWEGMHTLFPELWLLPSWIPAHPSTLWIHCRMVYIGMAFLYGKRYYAQEDEVILELRKELFL
EEFDEIDWPSQRNNIAEVDLYTPHSWLFKIAFGILDQYESFRLNRYRNNALDACLDHIKQDDLMTSFISIGPISKMINMLIRYLEDGPDSEAFK
KHVERVYDYVWMGLDGSNMQGTNGNQVWDVSFAGMAMLDVGVQDDPRFHEVLRKTYSYLEISQVIESAPDCVKYYRQYNKGGWAQTMREHGLVV
SDTSAEALKAVLMMNEKCPFISKRVSKRRLRDAVDMLLTMVNPNGGFSSYENLRGGKILELLNPSEVFGDIMVDYTYTECTSSVLQALRQFVDS
DPDYRHDEIWTVLKNSLEYIRSNQLPDGSFEGSWGVCFTYGTWFALEAFACMGKSFKYNTASIEVKKSLLIPCFQTDGGWRMG
>Helobdella
MENANFLRLIQADDGHWPADYGGPLFLMPGLVIACHVTNTTLQREKKLEMVRYLRSVQCREGGWGLHTEAPPTVLGTALNYVTMRLLGVSARDL
DLVRALGILRSLGGTLSIPSWGKFWLAVLNVYSWDGLHSLAPELWSLPECLPFHPSKMWCHCRQVYLGMSYCYAVRFKVDDSEIVRQLREELYQ
DNYDVIVWRHHRSNISLADLHTPHTYLLKSAYYLIDVYEKFHMTYLREKSLNLIYEHVVFDDDVTNFISIGPISKVIQMIVRYHRNDNEALKKH
RKRIDDYLWLGTDGLKMNGTNGSQLWDCTFAMQALLEVTLKNGLKFLLATQIDGLNNNNNNNNRTSVYRDFSKYYRHPNKGGFPFSTYECGWIV
ADCTAEALKTMMMMRRRRKKKMMMVEKSVNTDNDLDTGDVALLTLQNDDGGFATYERKRGGTFLESLNPSEVFGDVMLDYSYVECTSAAMQAF
HFFTEYIDRNYKKEIIKQALEKCLEFIVNSQQVDGSYYGSWAVCYTYGTWFALEGLSCVGYHYVRAPHPSLTKACDWLASKQQPTNGGWGEKFE
SCELKRYIDSTSSATATTKTTSSSSSSTEEPQVVNTSWALLGLMAAGYPDVNCIERGVRYLLGMQNDDGSYPKQNVSGVFNKTCSIHYDLYRNI
FPMWVIARY
>Capsaspora
ANRGMHFYTNLQCETGHWPGDYGGPHFLLPGLLITAHITGIQLGEFRKAEMMRYLRNTVTAEGGWGLHTADKATVFGTGLNYVAFRLLGGSRDD
PLAVKARTFLHKHDGVLGIPSWGKFWLALLNVYDYDGMNAVPPELWLLPEWAPLHPKYMWCHCRQVYLPMSYCYGVKLRAPVTPIIAELREELY
TQAYDTINWPAQRFRCAKIDLYNPHSWIMDWSFSALNVYEKFHSTSIRKRALDMALDHIRAEDENTQCVGIGPISKNINMICQWYAHGPQSPLF
RQHVSRFPDYLWLGIDGMKLNGTNGSQLWDTAFAVQAFLEAGGDKNAQLMASLRKAHSFFKLTQIRKNVPEHEKYFRQMSKGGFPFSTRDCGWI
VADCTAEGIKSTLMLENTGQISSPFEEERYHDAIDVLLSMQNSDGGYATYETKRGPEWLELFNPSEVFGAIMIDYTYVELTSAVVQALASLTVQ
FPKYRTAEISATMKRAVHFIRSIQRKDGSWEGCWAVCFTYGTWFGIEALATAGEGYYKGTASPSMRSACDFLVSKQRADGSWSESYMSCVERRY
IEHTESQVINTAWAVLGLMAAEYPDRTVVERGIQFIMSKQLMNGDWPDDEIKGVFNKNCMIVYPNYKNAFTIWALGRY
>Mixia
AQNGFEFYKHLQSPDGHWAGEYGGPHFLIPGLVIACYVTRTELPEEWRIEIARYLANLQRDNGPGDQGWGIHIEAVSSVFGSVMNYVALRLLGV
DAEEPMMQRARATFLHHGGASFIPSWGKFWLSVLNVHEWQGFNPTPPELWLLPTWLPMHPSKWWVHTRAVYIPFGYLSGKQFKAPLDALTKAIR
QEIYAEPYSMIDWNKARNQVAQVDMYCPHSTWLDYGFDALVQYEKHRPDWLRQRGIDYVYKLIVMEDENTGYQTIGPVSKAMNMICRWDREGPD
SEAFRLHKEKIRDFFWISRDGMMMTGTNGSQLWDAGFIGQAIADTGLADEEENHASALKLLDWLDRAQIRTNPRHYEQAYRHTTQGAWPFSTKE
QGYTVSDCTAEGLKAVIYLSKLKYAPKPVSYKRLCDAVDVLLSLQNSDGGFGSYELVRGPKWLELANCAEVFGNIMVEYTYPECTTACLTALTL
FSNEFPGYRSSTIERVSRRAIEYIHGTQRPDGSWFGSWAICFTYATYFAVESLAVNGETYKSSERVKRACDFILSKQKEDGGWGESYKSCETGE
YVQHERSQVVNTSWAVLTLLTAKCPDKAAVKRGVGLIMARQMPDGSWAQEAIEGIFNKNCAISYPNYKFSWTIWALGR
>Microbotryum
AQNGFEFYKRIQASDGHWSGEYGGPLFLLPGIVIAMYVTKTPIPEEWKIEIARYLSNVQRTNGPGDEGWGIHIEAQSSVFGTGLNYVTLRLLGV
DAEEPMMIRARATLHHLGGCTGIPSWGKFWLAILNVHSWSGLNPTPTELWLLPEFLPIHPYRWWIHTRNVYIPMGYLNGRYFQADLDPLILSLR
QEIYVEPYESIKWSSCRNNVCPIDLYAPHSAVANGLFAILNVYDRFAPAFIRNRGLARAYELIKMEDDNTSYQSVGPVSKAMHMICRWLEEGPD
SDAFKAHLSCIRDFMWVSSQGMLMSGTNGSQLWDTSFIGQALVESNLALEPSNKKSLEKMLDWLDHSQIRENPPHFEKAYRHRTMGAWPFSTKE
QGYTVSDCTAEGLKTVIMLQGLPGVKERVSKRRLCDAIDTILTMQNADGGFASYELIRGPHWLEWLNPAEVFGNIMTEYSYPECTTACVTALTI
FKRKHPDYRADDIDRVSKRAIEYIHKKQRPDGSWYGSWAICFTYAGMFAIESLSLAGEHFDNSESVKRACEFLLSKQMEDGGWGESYKSCETEQ
YVQHAQSQVTQTAWAVIGLITAQYPDTKPIRRGCRLIMSRQLPSGEWPQEAIEGVFNKNAAISYPNYKFAWTINALGQ
>Puccinia
AKNGFEFYKKLQMPDGHWSYLIDVLSGEFSGLVIACYITKTPLAEEVKIEIARGLANDQRQGQNVRDRGWGLHTSGKSTVFGTVLNYVACRLLG
IDAEHPMLVRARATLHALGGATGVPTWGKVWLALLGVYDWEGVNPVPPELWVLPEMLPFHPWRWWVHSRQVYLPISYLCGKRLQAQSDPTLASL
RNELYTQPYESIDWPRCRNSIAKEDLYSPRHPIANGLFWVLGYWERVCPSWIRNIGLDRAHELCKMEDENTDFNDLAPVNKVLNLIVCWDRYGP
ESEEFRQHQLKLKNFLWMNKNGMGMSSTNGSQLWDLAFITQALVESGLAKTDEPSTRDSVIKALEWIDRCQILDNPKHFKSGYRHQTKGAWPFS
TKSQGYTVSDCTAEALKSVLCLQEQLSYTPKLVSKDRLCLAVDVILSLQNPNGGFASYELVRGPSWLEYLSPAEVFGKTMIEVTYPECTTACLT
AMSLFSRCYPDYRAADISRAREGAIKFIHSAQRGDGSWYGSWGVCFTYATMFALESLSLNNETYKNSLSVKKACRFLLDRQMADGGWGESFKSC
EQGVYIHHQTSQVFQTAWAVLALLAAKYPEPEPIRRACRLIISRQTADGQWLDDAVEGVFNKTTSVTYPNYK

>Dacryopinax
ARKGFSFYKHLQSPDGHWAGEYGGPMFLIPGLVIGTYVTGQDFKDEEKIELIRYLFNTAHAEDGGWGLHVEHHSTCFGTVMNYVALRLLGVPPE
HPVCVKARGTMHSFGGAAALPQWGKWWLCLLNCYDWAGCIAIPAELWLFPQFLPIHPWRWWIHTRNVYIPMGFLFARKWQAPEDPLILQLREEI
YTTDYDHIDWPAQQFNVAPVDLYFPHTTLLHALGAVTHAWEHCFIPFIRRRAMEYTYQLVVQEDENTQCQDLGPVNKMLNQIVRFVVDGPESSA
FRLHVEKCRDFMWMTGAGMLMTGTNGSQLWDIAFICQAVVETGLGKEPGNEKATVGALKWLDGAQMRENPKHMQEAFRHQTKGAWPFSTPEQGY
TVSDCTGEGLKAVIYLQNHLDYTPKLVSERRMCDAIDVILSLQNPDGGCASYEPIRGPRWLEWINPAEVFGNIMIEYNYPECTTSALTALSIFT
NEYPQYRAEDIRKFKSKAIQWLHTSQHKDGTWFGSWGICFTYATMFALESLSLAGETYGNSPAVRKACDFLVSKQMPDGGWGESYKACETGEWV
DHAQSQVVNTSWATLALVYAQYPHRLPIERAVKLVISRQLPDGSWKQEAIEGVFNKNCAIAYPNFKFSFTIWMLGR
>Fistulina
AANGYKFYKHLQSHDGHWAGEYGGPMFLLPGLVIGSYISGMSFTDYERREMIRYLMNRAHPEDGGWGIHVEGPSTVFGTGLNYTAIRILGMDAD
HPVCLKARATLHKLGGAAGIPAWGKFWLSLLNCYEWAGNNPVPPELWVLPEFLPIHPHRWWIHTRAVYLPMSYLYGVRFKMPENDLIMSLRKEL
YPQDYYSIHWPSQRNNVAAIDLYAPHSALFDTINVALSAYEGCAFPPLRRRGIERAYELVVYEDENTSYQTLAPVNKMFNLVVRAHVDGPESTA
YKMHDIRRRDFMWLGAEGMMCCGTNGSQVWDTGFICQALQETGIGALEENRDDVVRALQWFEQAQILENPKHYHSAYRHATTGAWGFSTKEQGY
TVSDCTSEGLKAVMLLQHELDFTPKLVSEERMRMSVDLLLTMQNPGGGFASYEPIRAPHWLELLNPAEVFGNIMTEYCYPECTTSVITALSIFR
RYYPDYRAEDIDRTIRDAIGYLHREQTPEGGWVGSWGICFTYAAFFATESLALAGETYSSSPYQRRACDFLLSKQRTDGGWGESYKSCEQSKWI
EHENTQLVQTCWAAMALMHAKYPKAEPIERAVKLVMSRQKPDGSWAQEAIEGVFNKTCAIAYPNFKFSFPIWMLGK
>Ceraceosorus
ARNGFKFYRRIQSNDGHWVGEYGGPLFLLPGLVIGMYVTSTPVPAEWAIEIKRYLVNRANKDGGWGLHIAGESTAFGTATNYTVCRLLGMEADH
PAMIRARGVLHKLGGARGVPAWGKLWLAVLNVYDWEGMHPIPPELWCLPDWIPFHPWRWWIHTRMVYLPMGYLWAKRFKCPVDPLIESLRQELY
TEPYESIHWPSQRNHVAEVDIWAPHTKTMEGLMAVLGVYESCGSIPPLRRAGVKRAYQLLAMEDENTSYQTVGPVSKMLNYICRWIEEGPDSDV
MAKHREKLKDFMWMGRDGMMMTGTNGSQLWDTAFIAQAMVDSGLVDDDDTKNSCYRILDWLDEHQIRDNPKHYNAAYRYSSKGAWPFSTKEQSY
TVSDCTGEGMKATIMLQNDAKLPQRIAIERLHDSVDLLLTMQNRSGGFASYETINGPAVLEWLNPAEVFGDIMIEYDYPECTTSVVTGLLKFQQ
ISNYRKKDIDRCVKSAFGYIMKAQRPDGSWFGSWAVCFTYAMMFALESLHLAGQSCENSEPVRRACQFLLGKQREDGGWGEDFKSCVEGRYVQA
ATSRVVQTSWAVIALLHAGCEDKDAIKRGVSLIMGRQLDDGSWADEETVGVFNRNCSISYPNYVHSFTIWALGK
>Ustilago
AKNGLSFYRNLQSSDGHWAGEYGGPMFLLPGIVIGMYVTKTPIPEEWRIEIARYLWHRANKDDGGWGIHIEASSTVFGTALNYTLLRLIRVSAD
HPMMVKARGTLHKLGGATGIPSWGKLWLAILNCYEWEGMNPIPPELWLLPDWLPIHPWRWWIHTRMVYIPMGYLWGRRFKAEMDPLIASLREEL
YVQPYESIDWPSQRGNIAQVDTFYPHTKTLKTLMALVGAYDHCHIPALRKAGIQRAYDLLVLEDENTSYQCLGPVNKMLNYIARWDVQGHDSHA
MRMHREKLKDFMWIGAEGMMMTGTNGSQLWDTAFIAQAMVDSGLAASPENKQLTQKLLDWLDDCQIRSNPQHHKTCYRFATKGAWPFSTKEQGY
VVSDCTGEGLKAVIMLQENVAYLGKQVSRERMHDSIDLLLTMQNSGGGFASYERINGPAMLELINPAEVFGDIMIEYAYPECTTSVVTALLKFT
KIDDYRKQDIKSTVHSAVKYILKAQRKDGSWFGSWAICFTYATMFAVESLMLAGHTYENSDAIRKACEFLESKQMLDGGWGETYESCVKGVYTH
AAKSQVVQTAWVVITLLHAKYPKKDRIKKAVKVIMDRQLSDGSWAQEQIEGIFNRNCAISYPNYKFSFTIWALGK
>Malassezia_
AENGLAFYRHLQSSDGHFAGEYGGPMFLLPGLVIGMYVTETPIPEPWRIEIARYLWHRRHPEDGGWGIHIEGHSTVFGTALNYVVLRLVGVPAE
HPMMVQARGTLWKLGGAAGIPSWGKLWLAVLNVYDWEGLNPIPPELWVLPDFVPIHPWRWWVHTRMVYLPMGFLYGKRFTAPVTPLIESLRAEL
YPTAYKDIDWPAQRNHVASVDLYAPHTRVVNALFYVLGKYEHIHIPSLREAGMKRAYELIVKEDSNTSFQCLGPVNKMLNYIVRWIVDGPNSEA
MERHREKLKDFAWMGAEGLMMTGTNGSQLWDTSFIAQAMCESGLSAKPANHEICRRILSWLDMCQIRENPRFHRTAYRFATKGAWPFSTREQGY
TVSDCTAEGLKGVLMLQEASGASLGRPVSQRRLRDTVDLLLSMQNPGGGYASYETINGPSFTEWLNPAEVFGNIMVEYAYPECTTSVVSGLRMF
QQYDDYRSAEIDAVERAVKYILGAQRPDGSWYGSWAICFTYAAMFALESLKHAGYTYENSEPVRRACTFLLSKQREDGGWGESYKSCETHEYV
QSDSQVVQTSWAIIGLLHAGYPEAKPIQRAVTLIMQRQLPDGSWAQEQIEGIFNQ
>Wallemia
ARNGFKFYKQIQSVDGHWAGEYGGPMFLLPGLVISNYISGVKLAEEQRVEIIRYLFNRAHEVDGGWGIHIESPSTVFGTALNYTALRLLGVEAD
HPVMTKARGTLHKLGGATGSPAWGKFWLACLGVYDWDGVNPVPPELWLLPDWLPIHPTKWWIHCRQIYLPMSYLYGKRFIGRSTELTEQLKQEL
YVDPYASIDWSAQRNNVNKIDLFQPHSAVMDALNAALYYGYEPCSIPPLRQAALRKAYTHIVHEDENTCYQGLAPINKAFNQLCRLDADGPDSN
AVKKHLEKTNDFLWLGPDGLRETGTNGSQLWDAAFIAQALVETGLGEEEENKQATEKLLEWIDKCQIRQDPLYPAYRQPTKGAWPFSTAEQGYT
VSDCTAEGIKSVIYLQEYLKHTKHHVSYERLHDSVDLLLGMQNADGGFASYELTRGSKHLELLNPAEVFGKIMVEYSYPECTTSVVTALSLFKK
HQPDYRREDIERTTNAAIKYIHAAQREDGSWYGSWAICFTYAAMFALESLSSVGEHYENSEKVRKACEFLLSKQKEDGGWGESYLSCETEEYTQ
HQDSQVVQTSWVVLALLHARYPHKEPIKRAVRLIMSRQNKDGSWTQEAIEGIFNKNCAISYPNYKFSFTIWALGK
>Trichosporon
ARNGLRYYRELQSEDGHFATEYGGPLFLTPGLIIALQVCGVELPQPKKDELKRYLLNKLRPEGGWGLHTAAPPTVYGTVMNYVCLRMLGMGPDE
GPMTAIRAKIHEFGGAVAIPTWGKRRANNRCLPDWVPFAPWRWWIHVRLVFTPMAWLWGARFVAPQTELVRELRKEIYVQPYESIDWPRQRNNT
HKVDLYSPHHPVYDALNAILGVYEKMPYLPCVPGLRKAGMRAAYRQIVYEDENTGYQTVGPVSKAFNMICRFAQEGPDSEAVRMHKSRVDDFLW
LSKDGLFMTGTNGSQLWDISFLAQAVVETGLADEAENKQTVLGMLDWLDKCQIRHNPKWYRAGYRQSSKGAWPFSTPEQSYTVSDCTAEGLKGV
IALQSLSYTDKPVTIDRMRDAVDVLLSMQNPSGGFASYELMRGSAKLEALNAAEVFGDIMVDYMYPECTTSALSALTYFKKLDAQYRAADIEKC
VEKAIRWIHSVQRPDGSWYGSWGICFTYATMFALESLAIAGETYENSESVRRACSFLLSKQMEDGGWGETYMSCVSLQYAQHEQSQVVQTAWAL
LALIYGRCPDTAAIRRGCQLIMSRQRPDGSWLQEDTEGIFNKNCAIDYPAFKFIFCIWALGR
>Spizellomyces
GLKFYKQLQCSDGHFAGAYGGPQFLTPGYVITMYISGEVIDEARRVEMIRYLLGTAKEDGGWGIHVEGRSTVFGTALNYVTCRILGVERDHPAM
IKARGTLHRLGSATAIPAWGKFWLSVLGVYSYEGMNPVPPELWLLPYALPIHPGKWWIHTRMVYLPMGFIYGQRLVCRETNLIRELRREELYTQP
YESIKWSKMCNNVAESDIYYPHTRLLDGLNGICWLYEKLGPSRLRKWAIEECWLQLQMEDENTKFGNLASVNHPLNMLCTFFRLGQDSEEFKKH
KEHCLDYLWMSESGMHFNGTDGSQVWDTSFLSQAVCEAGLAEEPEFQTTMQRALDFLDSQQIRTDPINRKRCYRDATKGAWPFSRRDQGYTVSD
CTAEGMKSVLYLQDLEYTTKNVGQERFRDAVDVLLRMQNTDGGFASYELTRGPKILEWINPAEVFGDIMVEYSYPECTTAVMLGLLAFQKRDPA
YRSAEIQSALNRGLGYIKKSQKEDGSWFGSWAICFTYGTWFGLEALSHMGETYKNSERAQRACHFLLDHQMADGGWGEAYKSCETNVYHHHEKS
QVVNTAWACMGLMAAGYPDPTPIKRGIRLIMSRQKPDGRWEQEGIEGVFNRNAMIAYPNYKFIFSIWALGRF
>Pneumocystis
ARNGFLFYKHLQTKEGNWACEYGGPMFLLPGLIIAMYISKIPFSDEMRIEVIQYLVNHANPKDGGWGIHIEGKSTVFGTALNYVVLRILGLGPD
HPVTMKARIKLNELGGAIGCPQWGKFWLAVLNCYSWEGINPILPEFWMLPEWFPIHPSRWWVHTRAVYLPMGYIYGEKFTAPLDPLIESLREEL
YTQPYSSINFSKHRNTVSSVDVYVPHTTFLRVINSILTFYHTTFRLSWIKNIASKYTYELIEYENKNTDFLCIGPVNFCIHILAVYWREGPNSY
AFKSHKERLTDFLWVSKKGMMMNGTNGVQLWDTSFAVQALVESGLAEDPEFKDSMIRALDFLDKCQIQKNCDDQQKCYRHQRKGAWPFSTRQQG
YTVSDCTAEGLKAVLLLQNLKSFPKKVSYDRLKDSVDVILSLQNKDGGFASYELIRGPSWLESINPAEVFGNIMIEHSYTECTTSVVTALCYFR
SLCSHYRGSEINKSVKKAIQFIKESQRPDGSWYGSWAICFTYATMFALESLSCVKDFYENSFHSRRACDFLLNKQEKDGGWSESYQSCTDGIWT
RHPNGSQVVQTAWACIGLMYANYPDKTPIKRGIDLIMSRQQSNGEWKQEAIEGVFNKNCMISYPNYKFNFTIKALGMY
>Arthrobotrys_
ARNGLTFFSKLQLPGGNWACDYSGPMFQLAGMVMCWYSTGIPFPEDWAIEIIRYLCNRAHPEDGGWGLHTEGESSVFGTTINYTTLRLLGMPAD
HPVAVKARQKLHEMGGALGVPHWGKAWLAMIGCFEWEGMNPIPPELWLLPDWIPLHPYRWWIHTRAVYLPMSYIWSKRHSRPLNDLTRALRAEL
FIQPYSSINFAAHRNTIRAEDIYHPHTLVLDLFNTACTFWSKYLLPNWLKKWAEDYVYSLVVREDENTLYVNLGPVNNPLQMLCRYIAEGPDSP
AVLGHMDRLQDYLWVNEEGMLLNGTNGVQVWDTSFAVQSVVECGFSTQPEWKDMLNSAHQFLEDQQISLEISDEKQKCYRQQRKGAWAFSTRDQ

GYTVSDCTAEALKAVIQIQMTPGFPELVSNQRMRDAVDVMLALRNPSGGFASYEVIRGIKQLEWFNAAEVFGRIMIEYEYPECTTAVVTALVYF
QKVDKVYRAEEIKQTIKNAVDWIISAQRKDGSWEGSWGICFSYAMMFALESLRLVGYTYESSERVRLACQFLVDKQMPDGGWGESYMSCVKGQW
IDHEKSQVVNTSWALIALLQAGYPGKEVIKRGVDLLKSRQQKNGEWLQEGIEGVFNRSCMITYPNYKFAFPIKALGMY
>Aspergillus
AENGLEFFSKLQLPPGNWACEYGGPMFLLPGLIITYYVTNTPIPPEYATEIKRYLFARQHPEDGGWGLHIEAHSSVFGTCMNYVALRLIGVSED
DPRMIKARGLLHKFGGAIYGPHWAKFWLSVLGVMEWECVNPVPPELWLLPDWVPFTPWRWWIHIRQVFLPMSYLWSKKFTHPLDPLTKQLRQEL
YTQPYDSINFANHRNSIHAADNYYPKTWLLNLINQLLVSVWNPYFRIPALVKRAEEWTWELIRMEDENTDYAGLGPVSNPMNMVACYLHDGPDS
YSVRRHRERLNDYMWMKNEGMLMNGTNGVQVWDTAFITQAIVVAGFADDPKWRPMLTKALEFLEDHQLRENVPDQEKCYRQHRKGAWPFSNKTQ
GYTVSDCTAEGLRSTIQLQEMHNYPRLISVERLKDSVDCLLLMQNPSGGFTEYETTRGSEKLEWLNAAEVFGGIMIGYDYPECTTASVTALSLF
SRFYPDYRADEIKAAKDKAVRYIKRVQRPDGSWYGSWGICFTYAAMFALESLASVGETYETSEYARRGCEFLLSKQKEDGGWGESYLSSEKHVY
VQHEKSQVVQTAWACLALMEAEYPHKEPLQKAMKLLMSRQQPNGEWLQESIEGVFNQSCMISYPNYKFYWPIRALGLY
>Yeast-erg7
MTEFYSDTIGLPKTDPRLWRLRTDELGRESWEYLTPQQAANDPPSTFTQWLLQDPKFPQPHPERNKHSPDFSAFDACHNGASFFKLLQEPDSGI
FPCQYKGPMFMTIGYVAVNYIAGIEIPEHERIELIRYIVNTAHPVDGGWGLHSVDKSTVFGTVLNYVILRLLGLPKDHPVCAKARSTLLRLGGA
IGSPHWGKIWLSALNLYKWEGVNPAPPETWLLPYSLPMHPGRWWVHTRGVYIPVSYLSLVKFSCPMTPLLEELRNEIYTKPFDKINFSKNRNTV
CGVDLYYPHSTTLNIANSLVVFYEKYLRNRFIYSLSKKKVYDLIKTELQNTDSLCIAPVNQAFCALVTLIEEGVDSEAFQRLQYRFKDALFHGP
QGMTIMGTNGVQTWDCAFAIQYFFVAGLAERPEFYNTIVSAYKFLCHAQFDTECVPGSYRDKRKGAWGFSTKTQGYTVADCTAEAIKAIIMVKN
SPVFSEVHHMISSERLFEGIDVLLNLQNIGSFEYGSFATYEKIKAPLAMETLNPAEVFGNIMVEYPYVECTDSSVLGLTYFHKYFDYRKEEIRT
RIRIAIEFIKKSQLPDGSWYGSWGICFTYAGMFALEALHTVGETYENSSTVRKGCDFLVSKQMKDGGWGESMKSSELHSYVDSEKSLVVQTAWA
LIALLFAEYPNKEVIDRGIDLLKNRQEESGEWKFESVEGVFNHSCAIEYPSYRFLFPIKALGMYSRAYETHTL
>Candida
ARKGADFLKLLQLDNGIFPCQYKGPMFMTIGYVTANYYSKTEIPEPYRVEMIRYIVNTAHPVDGGWGLHSVDKSTCFGTTMNYVCLRLLGMEKD
HPVLVKARKTLHRLGGAIKNPHWGKAWLSILNLYEWEGVNPAPPELWRLPYWLPIHPAKWWVHTRAIYLPLGYTSANRVQCELDPLLKEIRNEI
YVPSQLPYESIKFGNQRNNVCGVDLYYPHTKILDFANSILSKWEAVRPKWLLNWVNKKVYDLIVKEYQNTEYLCIAPVSFAFNMVVTCHYEGSE
SENFKKLQNRMNDVLFHGPQGMTVMGTNGVQVWDAAFMVQYFFMTGLVDDPKYHDMIRKSYLFLVRSQFTENCVDGSFRDRRKGAWPFSTKEQG
YTVSDCTAEAMKAIIMVRNHASFADIRDEIKDENLFDAVEVLLQIQNVGEWEYGSFSTYEGIKAPLLLEKLNPAEVFNNIMVEYPYVECTDSSV
LGLTYFAKYYPDYKPELIQKTISSAIQYILDSQDNIDGSWYGCWGICYTYASMFALEALHTVGLDYESSSAVKKGCDFLISKQLPDGGWSESMK
GCETHSYVNGENSLVVQSAWALIGLILGNYPDEEPIKRGIQFLMKRQLPTGEWKYEDIEGVFNHSCAIEYPSYRFLFPIKALGLY
>Methylobacter
IKGINYYSYLQSEDGHWPGDYGGPLFLLPGLLIASYISGTPFPRAHREMMKLYLFNHQNQDAGWGMHIEGQSTLFGTVMQYVSLRLLGVDKDHQ
QLIKARAWIKNNGGATGIPSWGKFYLAVLNCYDWQGFNSLFPEMWLFPKWLPVHPWRYWCHTRMVYLPMAYCYAQRIKAPENELILSLREELYN
QDFAAIDWPKQRDAVCEKDRYTTLSPILKWMNFFTNSYEQFKCAWLRKKSTDYILKYLNAEDEQTNYINIGPVNQAINSICIWHAYGKDSTQFK
KHTARWYDYLWVAEDGMKMNGYNGSQLWDTAFATRAMLESDLGKLFPATIANSYRFIDQSQIKAEHPTHAEFFRHPMIGSWPFSTPDNGWPVAD
CTAEGLSAALAVHHSGLVHPAIDVMRIKKAVDIILSYQNSDGGWATYELTRAPKWLEKLNPSEVFADIMIDYSWTECTAACVISLLEIQETYPD
FKNSEIRKAIGAGLDFILKQQKADGSWYGGWAVCFTYATWFGVEAISKARGKGYHDDAILTDRINIASGFLAGKQKADGGWGETFESCSKLVYT
EAAKSQVVNTAWALLALMAADFGDRKVIESGITLLLNRQTHTGDWPQESISGVFNYNCMITYANYRNIFPVWALNRY
>Methylobactes
IKGINYYSYLQSEDGHWPGDYGGPLFLLPGLLIASYISGTPFPRAHREMMKLYLFNHQNQDAGWGMHIEGQSTLFGTVMQYVSLRLLGVDKDHQ
QLIKARAWIKNNGGATGIPSWGKFYLAVLNCYDWQGFNSLFPEMWLFPKWLPVHPWRYWCHTRMVYLPMAYCYAQRIKAPENELILSLREELYN
QDFAAIDWPKQRDAVCEKDRYTTLSPILKWMNFFTNSYEQFKCAWLRKKSTDYILKYLNAEDEQTNYINIGPVNQAINSICIWHAYGKDSTQFK
KHTARWYDYLWVAEDGMKMNGYNGSQLWDTAFATRAMLESDLGKLFPATIANSYRFIDQSQIKAEHPTHAEFFRHPMIGSWPFSTPDNGWPVAD
CTAEGLSAALAVHHSGLVHPAIDVMRIKQAVDIILSYQNSDGGWATYELTRAPKWLEKLNPSEVFADIMIDYSWTECTAACVLSLLEIQETYPD
FKNSEIRKAIGAGLDFILKQQKADGSWYGGWAVCFTYATWFGVEAISKARGKGYHDDAILTDRINIACGFLAGKQKADGGWGETFESCSKLVYT
EAAKSQVVNTAWALLALMAADFGDRKVIESGITLLLNRQTNIGDWPQESISGVFNYNCMITYANYRNIFPVWALNRY
>Methylosarcina
IKGINYFSTLQSEDGHWPGDYGGPLFLLPGLLIASYLAETPFPKAHREMMKLYLFNHQNKDAGWGMHIEGESTMFGTVMQYVSLRLLGVDKNHQ
QLVEARNWIKSHGGATGIPSWGKFYLAVLNLYDWQGFNSLFPEMWLFPKWLPVHPWRYWCHTRMVYLPMAYCYAQRIKAPENELILSLREEIYN
EDFAAIDWPKQRNAVCEKDCYTTPSPVLKWMNFFTNNYEKFKCSWLRKKSIDYILKYLHAEDQQTHYINIGPVNQAINSICMWHACGKDSEPFK
RHVARWYDYLWVAEDGMKMSGYNGSQLWDTAFATRAMLESDLGKLFPATIAKSYRFIELSQIQSEHSTHAEFFRHPMIGSWPFSTAENGWPVAD
CTAEGLSAALAVHHSGLIRPTIDATRIKKAVDVILSYQNDDGGWATYELTRAPKWLEKLNPSEVFADIMIDYSWTECTAACVISLLEIQETDPA
YRSNEIRQAISAGLNFILKQQKPDGSWYGGWAVCFTYATWFGVEALSKAKGKGYYDDAALAASISKACAFLVGKQKADGGWGETFKSCSTLVYT
EAETSQVVNTAWALLALMAAEFGEKEVIEAGIQVLLSRQSRIGDWPQESISGVFNYNCMITYANYRNAFPIWALNRY
>Fluviicola
LNGFSFYETLQEPDGNWAGDYGGPLFLIPGLVIASYITETPFEKPMQVLIKRNLWNHQNEDGGWGLHIEGHSTMFGTVMQYVTLRILGEDLSNP
QMKRAQDWILRHGGAIKVPQWGKFYLSVLNVYDWKGNDALLPEMWKFPKWVPFHPGKYWCHNRMIFLPMTYCFGKKVKAELTPLVQELRKELYT
ADYDSINWKKARREACEIDIYHPVNKWYYRLSNIINGYERIHSNRFRKKALKYVEDYIDFEDTYTRYINIGPVNQAINSICVWHNHGRDSEQFK
SHVKRWKDYLWIAEDGAKMSGYNGSQLWDTGFAGQALIEADMETDFPEMAEKIYRFIDSTQIERNAKDYSKYWGDVTLGCWPFSTIDHGWAITD
CTSEGMKTAILYNSREHIQKKNHVHIERLKPAVDWLLKMQNKDGGWASYEKQRAPKWIEVLNPAMLFENIMTEATYTECSSATIQGLKEFTKEH
DYRQEDIKRAIDRGAKFLESKQDPDGSWYGCWGVCYTYGTWFGIEGLLTAGHKHYENGTPSPEIQKACDFLLSKQRADGSWGESFQSCVEHRYV
EHEDGQIVNTAWALLALMKAKHPNKAVIEKGIEFILSRQESTGDFPQEGVSGIFNGNCAITYTSYRNVFPLWAIGRY
>Naegleria
LKAIDFYSSVQTQDGHWAGDYGGPMFLLPGIVIVLYICQKRLPKPFEYEIVRYILSKQNQDGGYGLHIEGHSTIFGTVLNYVALRLLGVSPDHS
CMKLTLEFLSKEPANGALGAPQWAKLYLCCLGLMDWDCIDPIPCELWLLPDWFPYIQPGKWWCHCRVVYLPMAYLYGIKKVYANAETDPIISQL
RNELYDQVSKTSYQNQPWSKYRSYVNPRDNYHPFTQLYSKFASVLTVYEKYINFSFLRNASLKKCIEHIKYEDSVTNHICIGPVNKVLNMLSVF
FSDGSDSELFNKHLDRVYDYLWLSDDGMKFQGYNGSQLWDTAFASQAICEYYKRFKNQDNQISCQGSFKNEHLNSLLNAYNFINFTQVKEDVPN
RMEYYRHQSKGGWPFSTRDHGWPISDCTAEGLKAVLTLYDFDELILSEERLTDAVRVILSMFNGGTNGGWATYELSRTHSWIEIINPAALYGEI
MIDYPHTECTSACITALLQFKKHFPQSPYVPMIDDSIKHAIKVIEGKQMEEGGWYGGWAVCFCYGTWFATTAIVSADPTLNYGNSQALKRGCDF
IVAKQMEDGGWGESYLSCVTHRYSHAETSRVISTAWSLLALMTSNYPNLKVIEKGIYCLMRKQLPNGDFPQESISGVFNHNCMITYTNYRNIFP
IWALSMY
>Polysphondylium
AVQYFTQVQTEDGHWAGDYGGPMFLLPGLVITCYVTGYKLPEPHVQEIIRYLLNRQNPKDGGWGLHIEAHSDIFGTALQYVSLRLLGLPVDHPG
VERARKFLRDNGGATGIPSWGKFWLATLNVYSWDGLNPIPIEFWLLPYSVPICPGRWWCHCRMVYLPMSYLYARRTTAAETPLIRELRKELYVT
PYSEINWPAQRDHINKLDMYAPHSYLLKSVNGALNLYERMHSKWLRDKAIDFTFDHIRFEDEQTKYIDIGPVNKTLNMLVVWDREGQSPNFFKH
ADRLYDYLWLASDGMKMQGYNGSQLWDTAFTIQAFVESGISHQFPEAMRMANHYLDITQVPDNAPDGYFRHISKGAWPFSTVDHGWPISDCTAE
GIKAALALRSLPNIVPISLDRVAEGVNVILSLQNSDGGWASYENKRGPNWLELFNPSEVFQNIMIDYSYVECSAACIQAMSSFLKHAPEHPRAR
EIRRSIDRGIKFIKSIQRDDGSWLGSWGICFTYGTWFGVEGLVASGEPLNSPHLVKACKFLISKQREDGGWGESFRSNVTKNYVQHEQSQIVNT
GWALLSLMAAKYPDREPIERGIKYLISKQYPNGDFPQESIIGVFNFNCMISYSNYKNIFPLWAISRY

>Acytostelium
YFSAVQTEDGHWAGDYGGPMFLLPGLVITCYVTGYSLPEAHCREIIRYMLNRQNPKDGGWGLHIEAHSDIFGTALQYVSLRILGLPAAHPGVTR
ARDFLRANGGAVGIPSWGKFWLAVLNVYSWDGLNPIPIEFWLVPYAFPICPGRWWCHCRMVYLPMSYLYARRTTAAETPLIRELRQELYVTDYS
TINWPAQKNSINKLDMYAPHSTLLKGINAALGVYEGVHSKWLRDKAIDFTFDHIRYEDEQTKYIDIGPVNKTLNMLCVWDREGQSPNFFKHADR
LQDYLWLANDGMKMQGYNGSQLWDTAFTIQAFVETGIAGQFPDTMRLANHYLDISQVPDNSPNMNHYFRHISKGAWPFSTVDHGWPISDCTAEG
VKAALSLRSLPFHIAPISIDRVAEGINVILSLQNKDGGWASYENKRGPNWLEKFNPSEVFQNIMIDYSYVECSAACIQAMCAFRSQAPNHPRIK
EVNGSIERGVRFIKSIQRDNGSWLGSWGICFTYGTWFGVEGLVAAGEPLTSPHIVKACKFLLSKQRDDGGWGESFMSNVTKEYVHNDQSQIVNT
GWALLTLMAAKYPQREPIERGIKFLISRQYPNGDFPQESIIGVFNFNCMISYSNYKNIFPLWALARY
>Dictyostelium
YFSKVQTEDGHWAGDYGGPMFLLPGLVITCYVTGYQLPESTQREIIRYLFNRQNPVDGGWGLHIEAHSDIFGTTLQYVSLRLLGVPADHPSVVK
ARTFLLQNGGATGIPSWGKFWLATLNAYDWNGLNPIPIEFWLLPYNLPIAPGRWWCHCRMVYLPMSYIYAKKTTGPLTDLVKDLRREIYCQEYE
KINWSEQRNNISKLDMYYEHTSLLNVINGSLNAYEKVHSKWLRDKAIDYTFDHIRYEDEQTKYIDIGPVNKTVNMLCVWDREGKSPAFYKHADR
LKDYLWLSFDGMKMQGYNGSQLWDTAFTIQAFMESGIANQFQDCMKLAGHYLDISQVPEDARDMKHYHRHYSKGAWPFSTVDHGWPISDCTAEG
IKSALALRSLPFIEPISLDRIADGINVLLTLQNGDGGWASYENTRGPKWLEKFNPSEVFQNIMIDYSYVECSAACIQAMSAFRKHAPNHPRIKE
INRSIARGVKFIKSIQRQDGSWLGSWGICFTYGTWFGIEGLVASGEPLTSPSIVKACKFLASKQRADGGWGESFKSNVTKEYVQHETSQVVNTG
WALLSLMSAKYPDRECIERGIKFLIQRQYPNGDFPQESIIGVFNFNCMISYSNYKNIFPLWALSRY
>Acanthamoeba
AEKAVAFYQQIQSEDGHWAMDYGGPMFLMPGLLIACHVTGVELPRPVQIEMVRYLTNRQNADGGWGLHIESPSTIFGTGMNYTAMRLLGVPMED
DRMKRAREFLAANDGCKGIPSWGKFWLAVMGVYDWEGLHPIPPELWLLPYMLPIHPGRWWCHCRMVYLPMGYVYGRRITAPESPLVLSLRKELY
PNDDYAKINWYSIRSYVSPLDLYYPHSTLLECLYVILDNYEKVHSSWLREKSVLLTAEHVAAEDKFTDWVCIGPVNKTINMLCSWHAQGKDSKE
FQRHVDRVPDYLWLAEDGMKMQGYNGSQLWDTAFSVQAIIETGLGDQFQECLQKAYSYIDITQVREDVEQMEYFYRHISKGAWPFSTRHHGWPI
SDCTAEGLKASLLLKQFSWVTPFEDQRYFDAVNVILSLQNSDGGWATYELQRGPSILEYINPAEVFDAIMVDYPYVECTSACVQALTMFVQHYP
RHRATEITTAVKKAVDLIKSKQRPDGSWYGSWGVCFTYGTWFGVEGLMAAGEPSDSPYIQRACQFLLSKQNHEGGWGETFESCSTKQYVQNEET
QVVNTAWAVLTLLKAQWPDRRPVDRAVQVLMKRQLPNGNWPQEDIKGVFNANCAISYTAYKNIFPIWALGLY
>Physcomitrella
MRFYSTLQAHDGHWPGDYGGPMFLMPGLVIALYVTGALNTVLTPAHQSEMRRYLYNHQNKDGGWGLHIEGHSTMFGSVLTYVTLRILGDGPEGG
DFDALRRGRKWILDHGGATYITSWGKFWLTVLGVFEWSGNNPLPPETWILPYFLPMHPGRMWCHCRMVYLPMSYIYGKRFTGKITELVKAIREE
IFVQKYTEVNWNEARNLCAKEDLYYPHPWIQDVLWGTLDKVVEPILTRWPGSLLRKKALARTMEHIHYEDENTRYLCIGPVNKVMNMLSCWIED
PNSEAFKLHLARVVDYLWVAEDGMKMQGYNGSQLWDTTFAVQALAATKLPDESMSMLKKANSYIDNSQVREDSPGDMAYWYRHISKGAWPFSSR
DHGWPISDCSSEGLKATLILADFPKELVGNPIAAERLYDAVNVILSYQNEDGGSATYERTRSYPWLEVINPAETFGDIVIDYSYVECTSACMQA
LASFQKRYPHHRTKEIAKSIQRARKYIESIQKDDGSWYGSWAVCFTYAIWFGVLGLIAAGQTYESSFHIRMACKFLLSKQLPDGGWGESYRSCQ
DKVYSNLPGGKSHVVNTSWAMLTLIAAGQWERNPEPLHRAASVLINKQMESGDFPQEEIMGVFNRNCMISYSAYRCIFPIWALGEY
>Selaginella
ALRFYSTIQAEDGHWPGDYGGPMFLMPGLIIALYVTGAINVVLSPEHHREMCRYFFNHQNEDGGWGLHIEGHSTMFGTVLVYVSLRLLGQEPDK
PAMKSALSWIFGHGGTIAIPSWGKFWLAVLGVFDWSGVNPLPPEMYLLPYAIPLHPGRMWCHCRMVYLPMSYIYGRRFTGKITPKVEALRKELF
NGPFEDVDWNRARNSCAKEDLYYPHPLIQDVLWGVLHKGVEPLLMRWPGSLLRKKALDTVLEHIHYEDVNTRYICIGPVNKVINMLCCWAEDPN
SEAFKQHLPRIHDYLWVAEDGLKMQGYNGSQLWDTAFAVQAIISTDMLEESREMLKKAHSYIEKSQVREDCPGDLDYFHRHISNGAWPFSNRDH
GWPISDCTAEGLKSVLLLSRISSDVVGKPLSPERLYDCVNMLISYQNANGGVATYELTRSYAWIELFNPSETFGDITIDYSYVECTSACVQALC
AFSRLYPDHRSKEIELFVAKGCQYIESIQRPDGSWYGSWGVCFTYGIWFGVLGLVAAGKSYKHSAAIRKACDFLVSKQLPSGGWGESYLSSQNK
VYMNLEGGKHHLVNTAWAMLALIAGGQASLDNLAERDSTPLHKAAALLVNGQLEDGDFPQEEIIGVFNGNCMISYSAYRCIFPIWALGQY
>Adiantum
AVLFYSTIQAEDGHWAGDYGGPMFLMPGLVIALYVTGSLNVVLSEAHKKEMVRYLYNHQNKDGGWGLHIEGHSTMFGSVLSYVTLRLLGQELSD
GEDQAMERGRAWILQHGGATTIPSWGKFWLSVLGTFEWAGNNPLPPEIWLLPYFLPIHPGRMWCHCRMVYLPMCYIYGNRFTGKITETVLALRK
ELFKVPYEDIDWNKARNECAKEDLYYPHPMIQDVLWATLHKLVEPALMNWPCSSLRKKALDTVIKHVHYEDENTRYICIGPVNKVLNMLCCWIE
DPNSEAFKCHLPRIPDYLWVAEDGMKMQGYNGSQLWDTAFAVQALISTGLLETCGPMLKKAHHFIDRSQVRNDCPGDLQFWYRHISKGAWPFST
RDHGWPISDCTAEGFKAALALSQLPSDIVGESLQAERFYDAVNTMLSYQNGNGGVATYELTRSYPWLELINPAETFGDIVIDYQYVECTSAVIQ
ALAAFKKLYPKHRTEEVNACIQHAAKYIESIQREDGSWYGSWGVCFTYAGWFGVIGLLSAGRTYESETLKKACNFLLSKKLSSGGWGESYLSCQ
DKVYTNLPNDRPHVVHTSWAMLALLYAGQAERDPRPLHEAATVLINSQLENGDYPQQEITGVFNRNCMISYSAYRNIFPIWALGEY
>Abies
VRAIRFYATIQAHDGHWPGDYGGPMFLMPGLVIALYVTGALNAVLSEMHKKEICRYLYNHQNEDGGWGLHIEGHSTMFGTVLNYVTLRLLGQAP
DGGQGAMEKGCAWILDHGGATAIPSWGKMWLSVLGVFDWTGNNPLPPEMWLLPYFLPTHPGRMWCHCRMVYLPMSYIYGRRFVGPLTGIVMSLR
EELYTVPYEKIDWNQARSMCAKEDLYYPHPFLQDILWGTLHKVVEPALMHWPGSMLRERALQSVMKHIHYEDENTRYICIGPVNKVLNMLCCWV
EDSNSEAFKRHLARVVDYLWVAEDGMKMQGYNGSQLWDTAFATQALISTNLLDDCGPLLKKAHIYIERSQVQEDCPGDLNFWYRHISNGAWPFS
TRDHGWPISDCSSEGLKAALALSQLPQDIVGKPIPSQRIFDCVNLMLSMQNSDGGFATYELTRSYPWLEKINPAETFGDIVIDYSYVECTSAIT
QALVSFKKLYPEHRHKEIETCILKATRYIENIQRPDGSWYGSWGVCFTYGTWFGVLGLAAAGKTYQNCSNIRKACEFLLSKQLPSGGWGESYLS
CQEKVYTHLEGGRSHIVSTAWAMLALIYAGQALRDPKPLHRAAIVLVNYQMENGDFPQQEIMGVFNRNCMISYSAYRNIFPIWALGEY
>Amborella
AIGFHTTIQAHDGHWPGDYGGPMFLMPGLVIVLYVTGALNTVLSTEHRKEICRYLYNHQNKDGGWGLHIEGHSTMFGTALTYVTLRLLGEGPED
GEDGAMEKGRKWVLDHGGLTAISSWGKMWLSVLGAFEWSGNNPLPPEIWLLPYFLPLHPGRMWCHCRMVYLPMSYLYGKKFVGPITTTVLSLRK
ELYTIPYNKVDWNDARNLCAKEDLYYPHPLVQDILWGFLHKVGEPIFMHWPGSKLREKALHTVMQHIHYEDENTRYICIGPVNKVLNMLCCWVE
DPNSEAFKLHLPRILDYLWVAEDGMKMQGYNGSQLWDTAFAVQAIISSNLVQEYYPALKKAHDYIKYSQVLENCYGDLQFWYRHISKGAWPFST
RDHGWPISDCTGEGLKVALLLSQISHDAVGEPIPANRFYDAVNVIISMQNSGGGFATYELTRSYAWLELINPAETFGDIVIDYPYVECTSAAIQ
ALASFKKLYPAHRTKEIETCISKAVHFIESIQRPDGSWYGSWGVCFTYATWFGVKGLVSAGKTYQTSPSIRKACDFLLSKQLPSGGWSESYLSC
QDKVYSNLKGNRLHLVNTAWAMLALIDAGQAERGPTPLHRASRVLINSQMDNGDFPQQEIMGVFNRNCMISYSAYRNIFPIWALGEY
>Malus
ALSFYSTIQAHDGHWAGDYGGPMFLLPGLIITLSITGALNAVLSKEHQREMCRYLYNHQNEDGGWGLHIEGPSTMFGTALNYVTLRLLGEGADD
GQGAIELARKWILDHGGVTAITSWGKMWLSVLGAYEWSGNNPLPPEVWLCPYSLPFHPGRMWCHCRMVYLPMSYLYGKFVGPITPTIRSLRKEL
YTAPYHEVDWNKARNLCAKEDLYYPHPMVQDVLWGSLHYVYEPVFTRWPAKELRDSALKTVMQHIHYEDENTRYICIGPVNKVLNMLCCWAEDP
NSEAFKLHLPRIPDYLWIAEDGMKMQGYNGSQAWDTSFAIQAIISTNLVEEYGPTLRKAHQYIKDSQVVEDCPGDLDYWYRHISKGAWPFSTAD
HGWPISDCTAEGLKAALLLSKLPTETVGESLDIKRFYDAVNVTLSLQNDDGGFATYELTRSYQWLELINPAETFGDIVIDYPYVECTSAAIQAL
ALFKRLCPAHRSDEIENCIARAAKFIETIQATDGSWYGSWGVCFTYAGWFGIKGLVAAGRTYEDCSSIRKACDFLLSKELPSGGWGESYLSCQN
KVYTNLKDNRPHIVHTAWAMLALIGAGQAKRDPTPLHAARVLINSQMKNGDFPQKEITGVFNKNCMISYSAYRNIFPIWALGEY
>AtCAS1
MWKLKIAEGGSPWLRTTNNHVGRQFWEFDPNLGTPEDLAAVEEARKSFSDNRFVQKHSADLLMRLQFSRENLISPVLPQVKIEDTDDVTEEMVE
TTLKRGLDFYSTIQAHDGHWPGDYGGPMFLLPGLIITLSITGALNTVLSEQHKQEMRRYLYNHQNEDGGWGLHIEGPSTMFGSVLNYVTLRLLG
EGPNDGDGDMEKGRDWILNHGGATNITSWGKMWLSVLGAFEWSGNNPLPPEIWLLPYFLPIHPGRMWCHCRMVYLPMSYLYGKRFVGPITSTVL
SLRKELFTVPYHEVNWNEARNLCAKEDLYYPHPLVQDILWASLHKIVEPVLMRWPGANLREKAIRTAIEHIHYEDENTRYICIGPVNKVLNMLC

CWVEDPNSEAFKLHLPRIHDFLWLAEDGMKMQGYNGSQLWDTGFAIQAILATNLVEEYGPVLEKAHSFVKNSQVLEDCPGDLNYWYRHISKGAW
PFSTADHGWPISDCTAEGLKAALLLSKVPKAIVGEPIDAKRLYEAVNVIISLQNADGGLATYELTRSYPWLELINPAETFGDIVIDYPYVECTS
AAIQALISFRKLYPGHRKKEVDECIEKAVKFIESIQAADGSWYGSWAVCFTYGTWFGVKGLVAVGKTLKNSPHVAKACEFLLSKQQPSGGWGES
YLSCQDKVYSNLDGNRSHVVNTAWAMLALIGAGQAEVDRKPLHRAARYLINAQMENGDFPQQEIMGVFNRNCMITYAAYRNIFPIWALGEYRCQ
VLLQQGE
>Populus
ALNFYSSIQAHDGHWPGDYGGPMFLLPGLVITLSITGALNAVLSDEHKKEMIRYLYNHQNRDGGWGLHIEGPSTMFGSVLNYVTLRLLGEGPND
GDGAMDKGHDWILNHGSATMITSWGKMWLSVLGVFEWSGNNPMPPEMWLLPYLLPVHPGRMWCHCRMVYLPMSYLYGKRFVGPITPIVLSLRKE
LFTVPYHEIDWNQARNLCAKEDLYYPHPLVQDALWALLDKAAEPVLMHWPGKKLREQALHTAMEHMHYEDENTRYICIGPVNKVLNMLCCWVED
PNSEAFKLHLPRIQDYLWLAEDGMKMQGYNGSQLWDTSFAVQAIISTKLVEEYGPTLRKAHAYIKNSQVLEDCPGDLSFWYRHISKGAWPFSTA
DHGWPISDCTAEGLKAALLLSKITPEIVGEPLAANRFYDAVNVILSLQNGDGGFATYELTRSYSWLELINPAETFGDIVIDYPYVECTSAAIQA
LVSFKKLYPGHRQEEIERCIRKATKFIESIQEKDGSWYGSWGVCFTYGTWFGINGLVAAGNFFNDNSSIRKACDFLLSKQCSSGGWGESYLSCQ
NKVYSNLEGNKTHVVNTAWAMLALIEAGQAERDPEPLHRAARSLINSQMENGDFPQQEIMGVFNRNCMITYAAYRDIFPIWALGEY
>Vitis
AINFHSTLQAHDGHWPGDYGGPMFLLPGLIITLSITGALNAVLSKEHRQEMCRYLYNHQNKDGGWGLHIEGPSTMFGTVLNYVTLRLLGEGANDADGAMEKG
RDWILNHGGATAITSWGKMWLSVLGVFEWSGNNPLPPEIWLLPYILPVHPGRMWCHCRMVYLPMSYLYGKRFVGPLTPTVLSLRKELYTVPYHEIDWNQARN
LCAKEDLYYPHPLVQDILWTSLDKVVEPILMHWPGKKLREKALRTVLEHVHYEDENTRYICIGPVNKVLNMLCCWVEDPNSEAFKLHLPRIFDFLWLAEDGMKM
QGYNGSQLWDTAFCVQAIISTDLVEEYGPTLRKAHAYLKKSQVLEDCPGNLDYWYRHISKGAWPFSTGDHGWPISDCTAEGLKAILLLSKIPSEIVGEPLDAKQLY
DAVNVILSLQNGDGGFATYELTRSYAWLELINPAETFGDIVIDYPYVECTSAALQALTSFNKFYPGHRREEIEHCIKKATMFIEKIQASDGSWYGSWGVCFTYAIWF
GIKGLVAAGKNYNNCSSICKACDFLLSKQLASGGWGESYLSCQNKVYSNLDGNRSHVVNTAWAMLALIDAGQAERDPTPLHRAARVLINSQMENGDFPQEEI
MGVFNRNCMITYAAYRNIFPIWALGEY

```
Helobdella               ------------------------------------------------------------ 0
ScLDS                    ------------------------------------------------------------ 0
AjLDS-b                  -MQSDNDHKPRSERTISEYSDLTRWRLSC-------------TEGKRIWHYVTEDETPER 46
AjLDS-a                  -MQSDNDHKPRSERTISEYSDLTRWRLSC-------------TEGKRIWHYVTEDETPER 46
PpLDS                    -MQSDSDQKPRSERPNKEYSDLTRWRLSC-------------TEGKRIWHYVTEDETPER 46
ScPS                     ------------------------------------------------------------ 0
PpPS                     ---------MSGSRRINPVTDLTRWRLTS-------------IDGRRRWHFISIDGTLER 38
AjPS-b                   ---------MPGLRRIDPVTDLTRWRLTS-------------IDGRRRWHFISIDGTLER 38
AjPS-a                   ---------MPGSRRIDPVTDLTRWRLTS-------------IDGRRRWHFISIDGTLER 38
Capsaspora               ------------------------------------------------------------ 0
Saccoglossus-LSS         MACSGGRRRRGGPYKTNPATDLTRWRLKN-------------VEGRQTWYYVEKNKKLLR 47
EpLSS                    ---MSYKKNFGGPYKTDPATDLTRWRLTN-------------VNGRQTWRYIPEGEEPDR 44
SpLSS                    ---MSEKKNRGGPYKTTPATDLSRWRLTN-------------TNGRQTWQYYPEGEEPGR 44
SgLSS                    ---MSDKKNRGGPYRTTPATDLSRWRLTN-------------VNGRQTWRYYPEGDEPGR 44
LspLSS                   ---MSGRRNRGGPHKTEPATDLTKWRLSN-------------VDGRQTWRFIEEGEAVDR 44
MgLSS                    ------------------------------------------------------------ 0
AaLSS                    ------------------------------------------------------------ 0
AfLSS                    ---MSGRRNRGGPHKTEPATDLTRWRLSN-------------VDGRQTWRFIEEGEPVDR 44
ArLSS                    ---MSGRRNRGGPHKTEPATDLTRWRLSN-------------VDGRQTWRFIEEGEPVDR 44
HspLSS                   ---MSGRRNRGGPHKTEPVTDLTRWRLSN-------------VDGRQTWRYIEEGETIDR 44
EsLSS                    ---MSGRRNRGGPHKTEPATDLTRWRLSN-------------VEGRQTWRYIEDGETIDR 44
ApLSS                    ---MSSRRNRGGPHKTQPVTDLTRWRLSN-------------VDGRQCWRYIEEGQPIER 44
PpLSS                    ---MSGRRNRGGPHKTEPVTDLTRWRLSN-------------VDGRQTWRYIEEGEPIER 44
PmLSS                    ---MSGRRNRGGPHKTEPVTDLTRWRLSN-------------VDGRQTWRYIEEGEPIER 44
Amphimedon               ------------------------------------------------------------ 0
Aplysia                  ------------------------------------------------------------ 0
Capitella                ------------------------------------------------------------ 0
Branchiostoma-LSS        -MGDSVIRRRGGPYKTEPVTDLTRWRLSN-------------VGGRQTWRYYGEGETPER 46
Lingula                  ------------------------------------------------------------ 0
Callorhinchus            ------------------------------------------------------------ 0
HumanLSS                 MTEGTCLRRRGGPYKTEPATDLGRWRLNC-------------ERGRQTWTYLQDER-AGR 46
Pteropus                 ------------------------------------------------------------ 0
DreLSS                   MTEGTCLRRRGGPYKTEPATDLSRWRLSN-------------VDGRQSWRYIEETDSLDR 47
Haplochromis             ------------------------------------------------------------ 0
Xenopus                  ------------------------------------------------------------ 0
Charadrius               ------------------------------------------------------------ 0
Anolis                   ------------------------------------------------------------ 0
Alligator                ------------------------------------------------------------ 0
Pneumocystis             ------------------------------------------------------------ 0
Arthrobotrys_            ------------------------------------------------------------ 0
Aspergillus              ------------------------------------------------------------ 0
Spizellomyces            ------------------------------------------------------------ 0
Trichosporon             ------------------------------------------------------------ 0
Puccinia                 ------------------------------------------------------------ 0
Mixia                    ------------------------------------------------------------ 0
Microbotryum             ------------------------------------------------------------ 0
Dacryopinax              ------------------------------------------------------------ 0
Fistulina                ------------------------------------------------------------ 0
Wallemia                 ------------------------------------------------------------ 0
Ceraceosorus             ------------------------------------------------------------ 0
Ustilago                 ------------------------------------------------------------ 0
Malassezia_              ------------------------------------------------------------ 0
Fluviicola               ------------------------------------------------------------ 0
Methylobacter            ------------------------------------------------------------ 0
Methylobactes            ------------------------------------------------------------ 0
Methylosarcina           ------------------------------------------------------------ 0
Aphanomyces              ------------------------------------------------------------ 0
Cystobacter              ------------------------------------------------------------ 0
Galdieria                ------------------------------------------------------------ 0
Selaginella              ------------------------------------------------------------ 0
Panax                    ------------------------------------------------------------ 0
Physcomitrella           ------------------------------------------------------------ 0
Cucumis                  ------------------------------------------------------------ 0
Adiantum                 ------------------------------------------------------------ 0
Abies                    ------------------------------------------------------------ 0
Malus                    ------------------------------------------------------------ 0
Amborella                ------------------------------------------------------------ 0
Nicotiana                ------------------------------------------------------------ 0
AtCAS1                   -----------------------MWKLKIAEGGSPWLRTTNNHVGRQFWEFDPNLGTPED 37
Glycine                  ------------------------------------------------------------ 0
Coffea                   ------------------------------------------------------------ 0
Populus                  ------------------------------------------------------------ 0
Vitis                    ------------------------------------------------------------ 0
Micromonas               ------------------------------------------------------------ 0
Volvox                   ------------------------------------------------------------ 0
Chlorella                ------------------------------------------------------------ 0
Synchroma                ------------------------------------------------------------ 0
Chattonella              ------------------------------------------------------------ 0
Ochromonas               ------------------------------------------------------------ 0
Chromulina               ------------------------------------------------------------ 0
Chondrus                 ------------------------------------------------------------ 0
Acanthamoeba             ------------------------------------------------------------ 0
Dictyostelium            ------------------------------------------------------------ 0
Polysphondylium          ------------------------------------------------------------ 0
Acytostelium             ------------------------------------------------------------ 0
SHC-Candidatus           ------------------------------------------------------------ 0
SHC-Chlorogloeopsis      ------------------------------------------------------------ 0
SHC-Streptomyces         ------------------------------------------------------------ 0
```

```
SHC-Hyphomicrobium        ------------------------------------------------------------ 0
SHC-Leptospirillum        ------------------------------------------------------------ 0
Naegleria                 ------------------------------------------------------------ 0
Symbiodinium              ------------------------------------------------------------ 0
Prorocentrum              ------------------------------------------------------------ 0
Gymnodinium               ------------------------------------------------------------ 0
Alexandrium               ------------------------------------------------------------ 0
Dinophysis                ------------------------------------------------------------ 0
Scrippsiella              ------------------------------------------------------------ 0
Aureococcus               ------------------------------------------------------------ 0
Phaeodactylum             ------------------------------------------------------------ 0
Helicotheca               ------------------------------------------------------------ 0
Yeast-erg7                MT------EFYSDTIGLPKTDPRLWRLRT------------DELGRESWEYLTPQQAAND 42
Candida                   ------------------------------------------------------------ 0
Verrucomicrobia           ------------------------------------------------------------ 0
Eudoraea                  ------------------------------------------------------------ 0
Plesiocystis              ------------------------------------------------------------ 0
Enhygromyxa               ------------------------------------------------------------ 0
Bodo                      ------------------------------------------------------------ 0
Trypanosoma               ------------------------------------------------------------ 0
Phytomonas                ------------------------------------------------------------ 0
Leishmania                ------------------------------------------------------------ 0
Leptomonas                ------------------------------------------------------------ 0
Labilithrix               ------------------------------------------------------------ 0
Stigmatella               ------------------------------------------------------------ 0
Myxococcus                ------------------------------------------------------------ 0
Corallococcus             ------------------------------------------------------------ 0
S-cinnamoneus             ------------------------------------------------------------ 0
S-caatingaensis           ------------------------------------------------------------ 0
Sandaracinus              ------------------------------------------------------------ 0
S-alboviridis             ------------------------------------------------------------ 0
G-obscuriglobus           ------------------------------------------------------------ 0
G-sp.SH-PL17              ------------------------------------------------------------ 0
G-sp-IIL30                ------------------------------------------------------------ 0
Methylococcus             ------------------------------------------------------------ 0
Methylocaldum             ------------------------------------------------------------ 0


Helobdella                ------------------------------------------------------------ 0
ScLDS                     ------------------------------------------------------------ 0
AjLDS-b                   ------------PQNMVEKYSLGLDY----SN---EAEKLP----------RAQNPKEAA 77
AjLDS-a                   ------------PQNMVEKYSLGLDY----SN---EAEKLP----------RAQNPKEAA 77
PpLDS                     ------------PQNMVEKYSLGLDY----SN---EAEKLP----------RAQNPKEAA 77
ScPS                      ------------------------------------------------------------ 0
PpPS                      ------------EQNVIEKYSLGLDY----SD---EANKLP----------DAESAAEAA 69
AjPS-b                    ------------EQNVLEKYSLGLDC----SD---EAKKLP----------DAESAAEAA 69
AjPS-a                    ------------EQNVLEKYSLGLDC----SD---EANKLP----------DAESAAEAA 69
Capsaspora                -----------------------------------------------------------A 1
Saccoglossus-LSS          ------------EQNMVELHSLGLDT----SM---HAPDLP----------RPKTVKDAA 78
EpLSS                     ------------PQNFVEKFALGLSI----DE---LTPPLS----------KAKTAKEAA 75
SpLSS                     ------------PQNFVEKFSLGLDI----DD---EAPPLP----------RAKTAEEAA 75
SgLSS                     ------------PQNFVEKFSLGLDI----NG---EAPPLP----------KARNAKEAA 75
LspLSS                    ------------EQNFVEKFSLGLDI----SE---EAPPLA----------KPATAQEAA 75
MgLSS                     ------------------------------------------------------------ 0
AaLSS                     ------------------------------------------------------------ 0
AfLSS                     ------------EQNFVEKFSLGLDI----SE---EAPPLA----------KPATAQEAA 75
ArLSS                     ------------EQNFVEKFSLGLDI----SE---EAPPLA----------KPATAHKAA 75
HspLSS                    ------------EQNLVEKFSLGLDI----SK---EAPPFP----------KPTSAQEAA 75
EsLSS                     ------------EQNLVEKFSLGLDI----SQ---EAPPFA----------KPTSAQEAA 75
ApLSS                     ------------EQNFVEKFSLGLDI----SK---EAPPLP----------KPTTPQEVA 75
PpLSS                     ------------EQNLVEKFSLGLDI----SK---EAPPFP----------KPTTPQEAA 75
PmLSS                     ------------EQNLVEKFSLGLDI----SK---EAPPFP----------KPTTPQEAA 75
Amphimedon                ------------------------------------------------------------ 0
Aplysia                   -----------------------------------------------------------A 1
Capitella                 -----------------------------------------------------------A 1
Branchiostoma-LSS         ------------EQNMVERHALGLDT----SA---FAPALP----------KAQTAREAV 77
Lingula                   -----------------------------------------------------------A 1
Callorhinchus             ------------------------------------------------------------ 0
HumanLSS                  ------------EQTGLEAYALGLDT----KN---YFKDLP----------KAHTAFEGA 77
Pteropus                  -----------------------------------------------------------A 1
DreLSS                    ------------PQSMLERHSLGLDT----SE---FISASP----------AAHTAVEAA 78
Haplochromis              -----------------------------------------------------------A 1
Xenopus                   -----------------------------------------------------------A 1
Charadrius                ------------------------------------------------------------ 0
Anolis                    -----------------------------------------------------------A 1
Alligator                 -----------------------------------------------------------A 1
Pneumocystis              -----------------------------------------------------------A 1
Arthrobotrys_             -----------------------------------------------------------A 1
Aspergillus               -----------------------------------------------------------A 1
Spizellomyces             ------------------------------------------------------------ 0
Trichosporon              -----------------------------------------------------------A 1
Puccinia                  -----------------------------------------------------------A 1
Mixia                     -----------------------------------------------------------A 1
Microbotryum              -----------------------------------------------------------A 1
Dacryopinax               -----------------------------------------------------------A 1
Fistulina                 -----------------------------------------------------------A 1
Wallemia                  -----------------------------------------------------------A 1
Ceraceosorus              -----------------------------------------------------------A 1
```

```
Ustilago                 -----------------------------------------------------------A 1
Malassezia_              -----------------------------------------------------------A 1
Fluviicola               ------------------------------------------------------------ 0
Methylobacter            ------------------------------------------------------------ 0
Methylobactes            ------------------------------------------------------------ 0
Methylosarcina           ------------------------------------------------------------ 0
Aphanomyces              ------------------------------------------------------------ 0
Cystobacter              ------------------------------------------------------------ 0
Galdieria                ------------------------------------------------------------ 0
Selaginella              ------------------------------------------------------------ 0
Panax                    ------------------------------------------------------------ 0
Physcomitrella           ------------------------------------------------------------ 0
Cucumis                  ------------------------------------------------------------ 0
Adiantum                 ------------------------------------------------------------ 0
Abies                    ------------------------------------------------------------ 0
Malus                    ------------------------------------------------------------ 0
Amborella                ------------------------------------------------------------ 0
Nicotiana                ------------------------------------------------------------ 0
AtCAS1                   LAAVEEARKSFSDNRFVQKHSADLLMRLQFSRENLISPVLPQVKIEDTDDVTEEMVETTL 97
Glycine                  ------------------------------------------------------------ 0
Coffea                   ------------------------------------------------------------ 0
Populus                  ------------------------------------------------------------ 0
Vitis                    ------------------------------------------------------------ 0
Micromonas               ------------------------------------------------------------ 0
Volvox                   -----------------------------------------------------------L 1
Chlorella                ------------------------------------------------------------ 0
Synchroma                -----------------------------------------------------------A 1
Chattonella              ------------------------------------------------------------ 0
Ochromonas               -----------------------------------------------------------A 1
Chromulina               -----------------------------------------------------------A 1
Chondrus                 ------------------------------------------------------------ 0
Acanthamoeba             -----------------------------------------------------------A 1
Dictyostelium            ------------------------------------------------------------ 0
Polysphondylium          ------------------------------------------------------------ 0
Acytostelium             ------------------------------------------------------------ 0
SHC-Candidatus           ------------------------------------------------------------ 0
SHC-Chlorogloeopsis      ------------------------------------------------------------ 0
SHC-Streptomyces         ------------------------------------------------------------ 0
SHC-Hyphomicrobium       ------------------------------------------------------------ 0
SHC-Leptospirillum       ------------------------------------------------------------ 0
Naegleria                ------------------------------------------------------------ 0
Symbiodinium             ------------------------------------------------------------ 0
Prorocentrum             ------------------------------------------------------------ 0
Gymnodinium              ------------------------------------------------------------ 0
Alexandrium              -----------------------------------------------------------A 1
Dinophysis               -----------------------------------------------------------A 1
Scrippsiella             -----------------------------------------------------------A 1
Aureococcus              ------------------------------------------------------------ 0
Phaeodactylum            ------------------------------------------------------------ 0
Helicotheca              -----------------------------------------------------------A 1
Yeast-erg7               ------------PPST-------------FTQWLLQDPKFPQPHPERNKHSPDFSAFDAC 77
Candida                  -----------------------------------------------------------A 1
Verrucomicrobia          ------------------------------------------------------------ 0
Eudoraea                 ------------------------------------------------------------ 0
Plesiocystis             ------------------------------------------------------------ 0
Enhygromyxa              ------------------------------------------------------------ 0
Bodo                     ------------------------------------------------------------ 0
Trypanosoma              ------------------------------------------------------------ 0
Phytomonas               ------------------------------------------------------------ 0
Leishmania               ------------------------------------------------------------ 0
Leptomonas               ------------------------------------------------------------ 0
Labilithrix              ------------------------------------------------------------ 0
Stigmatella              ------------------------------------------------------------ 0
Myxococcus               ------------------------------------------------------------ 0
Corallococcus            ------------------------------------------------------------ 0
S-cinnamoneus            ----------------------------------------------MNAGDPVPDVSAAY 14
S-caatingaensis          ------------------------------------------------------------ 0
Sandaracinus             ------------------------------------------------------------ 0
S-alboviridis            ------------------------------------------------------------ 0
G-obscuriglobus          ----------------------------------------------------MP--HDPT 6
G-sp.SH-PL17             ------------------------------------------------------------ 0
G-sp-IIL30               ------------------------------------------------------------ 0
Methylococcus            ------------------------------------------------------------ 0
Methylocaldum            ------------------------------------------------------------ 0


Helobdella               MENANFLRLIQAD-DGHWPADYGGPLFLMPGLVIACHVTN---TT--------L------ 42
ScLDS                    TNGMTFFSLLQAD-DGHWPNDYSGPLFLMPGLFIVLYITK---TK--------V------ 42
AjLDS-b                  ENGIKFFSLMQAE-DGHWPNDYSGPLFLMPGLIIVLYITK---TE--------F------ 119
AjLDS-a                  ENGIKFFSLMQAE-DGHWPNDYSGPLFLMPGLIIVLYITK---TE--------F------ 119
PpLDS                    ENGIKFFSLMQAE-DGHWPNDYSGPLFLMPGLIIVHYVTK---TK--------F------ 119
ScPS                     TNGMTFFSLLQAD-DGHWPNDYSGPLFLMPGLFIVLYITK---TK--------V------ 42
PpPS                     INGMKFFSLLQCD-DGHWPNDYSGPLFLMPGLIIVLYITK---TK--------L------ 111
AjPS-b                   INGMTFFSLLQAD-DGHWPNDYSGPLFLMPGLFIVLYITK---TK--------L------ 111
AjPS-a                   INGMTFFSLLQAD-DGHWPNDYSGPLFLMPGLFIVLYITK---TK--------L------ 111
Capsaspora               NRGMHFYTNLQCE-TGHWPGDYGGPHFLLPGLLITAHITG---IQ--------L------ 43
Saccoglossus-LSS         HNGMMFYSKLQSE-DGHWSGDYGGPLFLMPGLLIVCHITN---VQ--------L------ 120
EpLSS                    KNGMRFYSKLQTE-DGHWSGDYGGPLFLLPGLIIVCYITG---VV--------L------ 117
```

```
SpLSS                   KNGMKFYSKLQTE-DGHWAGDYGGPLFLLPGLVIVCFITG---VV--------L------ 117
SgLSS                   KNGMEFYSKLQTE-DGHWSGDYGGPLFLLPGLVIVCFITG---IA--------L------ 117
LspLSS                  TNGMLFYSKLQTE-DGHWSGDYGGPLFLMPGLLTVCYITK---TE--------L------ 117
MgLSS                   ------------------------------------------------------------ 0
AaLSS                   ------------------------------------------------------------ 0
AfLSS                   TNGMLFYSKLQTE-DGHWSGDYGGPLFLMPGLLIVCYITK---TE--------L------ 117
ArLSS                   TNGMLFYSKLQTE-DGHWSGDYGGPLFLMPGLLIVCYITK---TE--------L------ 117
HspLSS                  TNGSIFYSRLQTE-DGHWSGDYGGPLFLMPGLLIVCYITK---TE--------L------ 117
EsLSS                   TNGSIFYSKLQTE-DGHWSGDYGGPLFLMPGLLIVCYITK---TE--------L------ 117
ApLSS                   TNGMLFYSKLQTE-DGHWSGDYGGPLFLMPGLLIVCYITK---TE--------L------ 117
PpLSS                   TNGMVFYSRLQTE-DGHWSGDYGGPLFLMPGLLIVCYITK---TE--------L------ 117
PmLSS                   TNGMVFYSRLQTE-DGHWSGDYGGPLFLMPGLLIVCYITK---TE--------L------ 117
Amphimedon              --GLDYYTRLQSD-DGHWSGDYGGPLFLLPGLIIVYHVTG---LQ--------F------ 40
Aplysia                 KNGMTFYSKMQAE-DGHWAGDYGGPLFLMPGLIIVCYITK---TP--------F------ 43
Capitella               YNGLQFYSKLQAE-DGHWAGDYGGPLFLTPGLVIVCYITN---TP--------F------ 43
Branchiostoma-LSS       RNGMKFYSKLQTE-DGHWAGDYGGPLFLMPGLVIVCHVTK---AR--------L------ 119
Lingula                 YNGIKFYSKLQAE-DGHWAGDYGGPLFLMPGLVIVCHITR---TP--------F------ 43
Callorhinchus           LKGLRFYAPLQAE-DGHWAGDYGGPLFLLPGLLIACHVTG---AG--------L------ 42
HumanLSS                LNGMTFYVGLQAE-DGHWTGDYGGPLFLLPGLLITCHVAR---IP--------L------ 119
Pteropus                LNGMTFYTGLQAE-DGHWAGDYGGPLFLLPGLLITCHVAR---IP--------L------ 43
DreLSS                  LKGMDFYSRLQAE-DGHWAGDYGGPLFLLPGLLITCHIAK---IP--------L------ 120
Haplochromis            VKGMHFYSQLQAE-DGHWAGDYGGPLFLLPGLLITCHVAK---IS--------L------ 43
Xenopus                 YNGITFYSALQAE-DGHWAGDYGGPLFLLPGLLIACHVTK---TS--------L------ 43
Charadrius              ---MRFYAALQAE-DGHWAGDYGGPLFLLPGLLITCHTVK---IQ--------L------ 39
Anolis                  INGVQFYSGLQAE-DGHWAGDYGGPLFLLPGLLITCHAAK---IP--------L------ 43
Alligator               LNGMQFYSGLQAE-DGHWAGDYGGPLFLLPGLLITCHVAK---IP--------L------ 43
Pneumocystis            RNGFLFYKHLQTK-EGNWACEYGGPMFLLPGLIIAMYISK---IP--------F------ 43
Arthrobotrys_           RNGLTFFSKLQLP-GGNWACDYSGPMFQLAGMVMCWYSTG---IP--------F------ 43
Aspergillus             ENGLEFFSKLQLP-PGNWACEYGGPMFLLPGLIITYYVTN---TP--------I------ 43
Spizellomyces           --GLKFYKQLQCS-DGHFAGAYGGPQFLTPGYVITMYISG---EV--------I------ 40
Trichosporon            RNGLRYYRELQSE-DGHFATEYGGPLFLTPGLIIALQVCG---VE--------L------ 43
Puccinia                KNGFEFYKKLQMP-DGHWSYLIDVLSGEFSGLVIACYITK---TP--------L------ 43
Mixia                   QNGFEFYKHLQSP-DGHWAGEYGGPHFLIPGLVIACYVTR---TE--------L------ 43
Microbotryum            QNGFEFYKRIQAS-DGHWSGEYGGPLFLLPGIVIAMYVTK---TP--------I------ 43
Dacryopinax             RKGFSFYKHLQSP-DGHWAGEYGGPMFLIPGLVIGTYVTG---QD--------F------ 43
Fistulina               ANGYKFYKHLQSH-DGHWAGEYGGPMFLLPGLVIGSYISG---MS--------F------ 43
Wallemia                RNGFKFYKQIQSV-DGHWAGEYGGPMFLLPGLVISNYISG---VK--------L------ 43
Ceraceosorus            RNGFKFYRRIQSN-DGHWVGEYGGPLFLLPGLVIGMYVTS---TP--------V------ 43
Ustilago                KNGLSFYRNLQSS-DGHWAGEYGGPMFLLPGIVIGMYVTK---TP--------I------ 43
Malassezia_             ENGLAFYRHLQSS-DGHFAGEYGGPMFLLPGLVIGMYVTE---TP--------I------ 43
Fluviicola              LNGFSFYETLQEP-DGNWAGDYGGPLFLIPGLVIASYITE---TP--------F------ 42
Methylobacter           IKGINYYSYLQSE-DGHWPGDYGGPLFLLPGLLIASYISG---TP--------F------ 42
Methylobactes           IKGINYYSYLQSE-DGHWPGDYGGPLFLLPGLLIASYISG---TP--------F------ 42
Methylosarcina          IKGINYFSTLQSE-DGHWPGDYGGPLFLLPGLLIASYLAE---TP--------F------ 42
Aphanomyces             -QAVDYYKLLQSE-DGSWHGDYGGPMFLLPGLVITSYITG---HD--------L------ 41
Cystobacter             --AMTHLSGLLSP-EGSLKGDYGGPLFMLPMYVGTAHAVG---LE--------L------ 40
Galdieria               -TAIQYYRRLQMS-DGHWPGDYGGPMFLLPGLVIVCYITE---TD--------L------ 41
Selaginella             --ALRFYSTIQAE-DGHWPGDYGGPMFLMPGLIIALYVTGAINVV--------L------ 43
Panax                   --ALKFYSTIQAD-DGHWPGDYGGPLFLLPGLVIGLYVMGVMDTI--------L------ 43
Physcomitrella          ---MRFYSTLQAH-DGHWPGDYGGPMFLMPGLVIALYVTGALNTV--------L------ 42
Cucumis                 --ALSFYSAVQTS-DGNWASDLGGPMFLLPGLVIALYVTGVLNSV--------L------ 43
Adiantum                --AVLFYSTIQAE-DGHWAGDYGGPMFLMPGLVIALYVTGSLNVV--------L------ 43
Abies                   VRAIRFYATIQAH-DGHWPGDYGGPMFLMPGLVIALYVTGALNAV--------L------ 45
Malus                   --ALSFYSTIQAH-DGHWAGDYGGPMFLLPGLIITLSITGALNAV--------L------ 43
Amborella               --AIGFHTTIQAH-DGHWPGDYGGPMFLMPGLVIVLYVTGALNTV--------L------ 43
Nicotiana               --ALSFYSTLQAH-DGHWAGDYGGPMFLMPGLVIALSVTGALSAV--------L------ 43
AtCAS1                  KRGLDFYSTIQAH-DGHWPGDYGGPMFLLPGLIITLSITGALNTV--------L------ 142
Glycine                 --AVSFHSTLQCH-DGHWPGDYGGPMFLMPGLVITLSITGALNTV--------L------ 43
Coffea                  --AISFYSTLQTH-DGHWAGDYGGPMFLMPGLIITLSITGALNAV--------L------ 43
Populus                 --ALNFYSSIQAH-DGHWPGDYGGPMFLLPGLVITLSITGALNAV--------L------ 43
Vitis                   --AINFHSTLQAH-DGHWPGDYGGPMFLLPGLIITLSITGALNAV--------L------ 43
Micromonas              --GVEFYQGLQDE-DGHWASDYGGPLFLLPGLIIALYVMGQLDHV--------L------ 43
Volvox                  NGAISFYECLQQD-DGHWPGDYGGPMFLLPGLVIGLYTTGALDQV--------F------ 46
Chlorella               -NGVSFYEGLQAE-DGHWPGDYGGPMFLMPGMVIALYTTGTLDSV--------L------ 44
Synchroma               RKGVAFYQMLQCD-DGHWAGDYGGPMFLMPGLIAVLYLTG---SM--------P------ 43
Chattonella             -KGIEFYQMLQTE-DGHWAGDYGGPMFLMPGLIICHYVTG---TP--------L------ 41
Ochromonas              SKGISFYQMLQSE-DGHWAGDYGGPMFLMPGLICVLYITK---TP--------F------ 43
Chromulina              EKALAYYQMLQCE-DGHWAGDYGGPMFLMPGLICVLYVTGQLHTT--------F------ 46
Chondrus                -KGLSFFATLQTG-DGHWAGDYAGPMFLLPGLVIACYVSR---TP--------L------ 41
Acanthamoeba            EKAVAFYQQIQSE-DGHWAMDYGGPMFLMPGLLIACHVTG---VE--------L------ 43
Dictyostelium           -----YFSKVQTE-DGHWAGDYGGPMFLLPGLVITCYVTG---YQ--------L------ 37
Polysphondylium         --AVQYFTQVQTE-DGHWAGDYGGPMFLLPGLVITCYVTG---YK--------L------ 40
Acytostelium            -----YFSAVQTE-DGHWAGDYGGPMFLLPGLVITCYVTG---YS--------L------ 37
SHC-Candidatus          ------------------------------------------------------------ 0
SHC-Chlorogloeopsis     ----NYLLSIQNP-SGYWWAELESNVTITAEVVLLHKIWG---TDR-------------- 38
SHC-Streptomyces        ----------QDE-RGWWKGDLETNVTMDAEDLLLRQFLG---IQD-------------- 32
SHC-Hyphomicrobium      ---------LQRP-DGHFVFELEADATIPAEYVLMRHYLG---EPV-------------- 33
SHC-Leptospirillum      --------------------------------------------PL-------------- 2
Naegleria               LKAIDFYSSVQTQ-DGHWAGDYGGPMFLLPGIVIVLYICQ---K-----RLPK------- 44
Symbiodinium            ------------------------------------------------------------ 0
Prorocentrum            ------------------------------------------------------------ 0
Gymnodinium             ------------------------------------------------------------ 0
Alexandrium             RRGFAFYQTLQCD-DGQWAGDYGGPHFLLPGFVIAAYIVG---RSR---VFSD------- 47
Dinophysis              RKGFAFYQMLQCD-DGQWAGDYGGPHFLLPGFVIAAYITG---GGDLTEVMAA------- 50
Scrippsiella            RQGFAFYQKIQCD-DGQWAGDYGGPHFLLPGFVIAAYITG---G--LTKTMPA------- 48
Aureococcus             -KALAFYQQLQCD-DGHWGGDYGGPHFLSPGLVVVWYVTG---RRD--DVLDE------- 46
Phaeodactylum           -----FYSMLQTS-DGHFSGDYGGPHFLMPGLIVVWYVMG---QPS--LMLNP------- 42
Helicotheca             KKAIHFYSMLQTE-DGHWAGDYGGPHFLMPGVIIAWYVMG---QPE--EMLDK------- 48
```

```
Yeast-erg7             HNGASFFKLLQEPDSGIFPCQYKGPMFMTIGYVAVNYIAG---IE--------I------ 120
Candida                RKGADFLKLLQ-LDNGIFPCQYKGPMFMTIGYVTANYYSK---TE--------I------ 43
Verrucomicrobia        ---------------------------VI-----TTYLTR---QP--------V------ 11
Eudoraea               ----------QTE-EGTWPVPYDGPFFLLPLYIFAMRICG---RH--------M------ 32
Plesiocystis           -RALGALERAQQA-RGAWAGEVVWNPMLICQYVIAMHVLG---RE--------I------ 41
Enhygromyxa            ----------QQP-NGAWAGEVVWNPMLICQYVICSHIVG---RE--------I------ 32
Bodo                   -SGAAFLMKLQHPTSGHWPNDYSGPMFLLPGAIFVKFIIA---RGDASKMFKFP------ 50
Trypanosoma            -DGVEFLLRLQDPYSGHWPNDYSGPLFLTPGFIFTKFIVA---GGDIRKMFPPHRDHQHK 56
Phytomonas             ------------------------------------------------------------ 0
Leishmania             -DGIRFLLKLQDPFSGHWPNGYSGCLFLCAGFVITKYIVA---GGETRRMFPPFSDHHHV 56
Leptomonas             -DGIRFLLKLQDPFSGHWPNDYSGCMFLVAGLIYTKYIIA---DGEAHRMFPPCPEHHHV 56
Labilithrix            ----------QGE-DGAWAGAYGGPMFLLPMYLALCHAAK---RP--------P------ 32
Stigmatella            --------------------------------------MG---RT--------P------ 5
Myxococcus             ----------QEA-DGSWKGDYSGPLFLSPLYLIGLYAMD---RA--------P------ 32
Corallococcus          -RARDMLAGTQAA-DGSWKGDYSGPLFLGPVYVAGLYVMG---RT--------P------ 41
S-cinnamoneus          EAACGRLLELQSD-DGSWEGEMEWNTTILSQYVIVTRVLG---RP--------P------ 56
S-caatingaensis        ----------QRQ-DGSWEGEMESNTTGTAQYVIVLRALG---RP--------L------ 32
Sandaracinus           -RGARALIAEQAD-DGSFEGEVVWCPMLAAQYVMGWHAMG---RP--------L------ 41
S-alboviridis          ---------LQGR-DGSWEGEMAWSVMILSQYVITRHLLG---RP--------L------ 33
G-obscuriglobus        ARAAARLTETQQP-AGCWEGEMIWCPVVTAQVAITRHVVG---MP--------F------ 48
G-sp.SH-PL17           ---------------------MIWCPVVTAQVAITRHVVG---LP--------F------ 22
G-sp-IIL30             -RAAEHLRALQTL-DGYWEGEMIWCPVVTAQVAITRHVVG---LP--------F------ 41
Methylococcus          ---MKHLLSLQRS-AGDWEGEMVWCTMILAQAVIVRTVVG---RP--------Y------ 39
Methylocaldum          -RALRHLLDLQGP-NGDWEGEMVWCTMILAQAVIVRTIVG---RP--------Y------ 41


Helobdella             ---------------------------------------QREKKLEMVRYLRSVQ----C 59
ScLDS                  ---------------------------------------PEAFMKESVRYLRTLQ----L 59
AjLDS-b                ---------------------------------------PEAFKQEFVRYLRRVQ----A 136
AjLDS-a                ---------------------------------------PEAFKQEFVRYLRRVQ----A 136
PpLDS                  ---------------------------------------PEAFKQEFIRYLRRVQ----A 136
ScPS                   ---------------------------------------PEAFMKESVRYLRTLQ----L 59
PpPS                   ---------------------------------------PDAFVKESIRYLRSVQ----L 128
AjPS-b                 ---------------------------------------PDAFVKESIRYLRSVQ----L 128
AjPS-a                 ---------------------------------------PDAFVKESIRYLRSVQ----L 128
Capsaspora             ---------------------------------------GEFRKAEMMRYLRNTV----T 60
Saccoglossus-LSS       ---------------------------------------SDAQKAEMIRYLRSI------ 135
EpLSS                  ---------------------------------------PEASKKEMIRYLRSVQ----C 134
SpLSS                  ---------------------------------------PDASKKEMVRYLRSVQ----C 134
SgLSS                  ---------------------------------------PDASKKEMVRYLRSVQ----C 134
LspLSS                 ---------------------------------------PDAVKKEMVRYLRSVQ----C 134
MgLSS                  ------------------------------------------------------------ 0
AaLSS                  ------------------------------------------------------------ 0
AfLSS                  ---------------------------------------PDAVKKEMVRYLRSVQ----C 134
ArLSS                  ---------------------------------------PDAVKKEMVRYLRSVQ----C 134
HspLSS                 ---------------------------------------PEAFRKEMVRYLRSVQ----C 134
EsLSS                  ---------------------------------------PEAFRKEMVRYLRSVQ----C 134
ApLSS                  ---------------------------------------QDAVKKEMVRYLRSVQ----C 134
PpLSS                  ---------------------------------------QDAVKKEMVRYLRSVQ----C 134
PmLSS                  ---------------------------------------QDAVKKEMVRYLRSVQ----C 134
Amphimedon             ---------------------------------------EDHQRLEMIRYLRNVQ----N 57
Aplysia                ---------------------------------------TEAQRVEMIRYLKSVT----C 60
Capitella              ---------------------------------------TDEQTKEMIRYLKSVE----C 60
Branchiostoma-LSS      ---------------------------------------SEPQRLEMIRYLRSVQ----L 136
Lingula                ---------------------------------------TPEQKLEMIRYLRSVQ----C 60
Callorhinchus          ---------------------------------------ADSQRQEMVRYLRSVQ----L 59
HumanLSS               ---------------------------------------PAGYREEIVRYLRSVQ----L 136
Pteropus               ---------------------------------------PAGYRREIVRYLRSVQ----L 60
DreLSS                 ---------------------------------------PDAWKKEMVRYLRSVQ----L 137
Haplochromis           ---------------------------------------PEAWKKEMVRYLRSVQ----L 60
Xenopus                ---------------------------------------PDATKKEMIRYLRSVQ----L 60
Charadrius             ---------------------------------------PEGFRKEMVRYLRSVQ----L 56
Anolis                 ---------------------------------------PEESKKEMVRYLRSVQ----L 60
Alligator              ---------------------------------------PEGYREEMVRYLRSVQ----L 60
Pneumocystis           ---------------------------------------SDEMRIEVIQYLVNHA----N 60
Arthrobotrys_          ---------------------------------------PEDWAIEIIRYLCNRA----H 60
Aspergillus            ---------------------------------------PPEYATEIKRYLFARQ----H 60
Spizellomyces          ---------------------------------------DEARRVEMIRYLLGTAK---- 57
Trichosporon           ---------------------------------------PQPKKDELKRYLLNKLR---- 60
Puccinia               ---------------------------------------AEEVKIEIARGLANDQRQGQN 64
Mixia                  ---------------------------------------PEEWRIEIARYLANLQRD-NG 63
Microbotryum           ---------------------------------------PEEWKIEIARYLSNVQRT-NG 63
Dacryopinax            ---------------------------------------KDEEKIELIRYLFNTA----H 60
Fistulina              ---------------------------------------TDYERREMIRYLMNRA----H 60
Wallemia               ---------------------------------------AEEQRVEIIRYLFNRA----H 60
Ceraceosorus           ---------------------------------------PAEWAIEIKRYLVNRA----N 60
Ustilago               ---------------------------------------PEEWRIEIARYLWHRA----N 60
Malassezia_            ---------------------------------------PEPWRIEIARYLWHRR----H 60
Fluviicola             ---------------------------------------EKPMQVLIKRNLWNHQ----N 59
Methylobacter          ---------------------------------------PRAHREMMKLYLFNHQ----N 59
Methylobactes          ---------------------------------------PRAHREMMKLYLFNHQ----N 59
Methylosarcina         ---------------------------------------PKAHREMMKLYLFNHQ----N 59
Aphanomyces            ---------------------------------------GKSVRDGMIVYLRNHQ----Q 58
Cystobacter            ---------------------------------------DAATREGMVRYLKSVQ----N 57
Galdieria              ---------------------------------------GNETKKEMKRYLYNHQ----N 58
Selaginella            ---------------------------------------SPEHHREMCRYFFNHQ----N 60
Panax                  ---------------------------------------AKEHQREMCRYIYNHQ----N 60
Physcomitrella         ---------------------------------------TPAHQSEMRRYLYNHQ----N 59
```

```
Cucumis                 ---------------------------------------SKHHRQEMCRYIYNHQ----N 60
Adiantum                ---------------------------------------SEAHKKEMVRYLYNHQ----N 60
Abies                   ---------------------------------------SEMHKKEICRYLYNHQ----N 62
Malus                   ---------------------------------------SKEHQREMCRYLYNHQ----N 60
Amborella               ---------------------------------------STEHRKEICRYLYNHQ----N 60
Nicotiana               ---------------------------------------SEEHKREICRYLYNHQ----N 60
AtCAS1                  ---------------------------------------SEQHKQEMRRYLYNHQ----N 159
Glycine                 ---------------------------------------TEEHRKEICRYLYNHQ----N 60
Coffea                  ---------------------------------------SKEHKLEMCRYIYNHQ----N 60
Populus                 ---------------------------------------SDEHKKEMIRYLYNHQ----N 60
Vitis                   ---------------------------------------SKEHRQEMCRYLYNHQ----N 60
Micromonas              ---------------------------------------PDYVQVEMRRYLLNHQ----N 60
Volvox                  ---------------------------------------TPHHKQEALRYLANHQ----N 63
Chlorella               ---------------------------------------SPQHKAEMVRYLRNHQ----N 61
Synchroma               ---------------------------------------PRYKQNAMALYLLNHQ----Q 60
Chattonella             ---------------------------------------AAYKRDAMIAYLRNHQ----Q 58
Ochromonas              ---------------------------------------PPGRREGMIAYLRNHQ----Q 60
Chromulina              ---------------------------------------PQYKIDAMILYLKNHQ----Q 63
Chondrus                ---------------------------------------PPAHRAEMLRYLRNHQ----N 58
Acanthamoeba            ---------------------------------------PRPVQIEMVRYLTNRQ----N 60
Dictyostelium           ---------------------------------------PESTQREIIRYLFNRQ----N 54
Polysphondylium         ---------------------------------------PEPHVQEIIRYLLNRQ----N 57
Acytostelium            ---------------------------------------PEAHCREIIRYMLNRQ----N 54
SHC-Candidatus          --------------------------------------------------ILERQ----L 6
SHC-Chlorogloeopsis     ---------------------------------------SRA-LHKVETYLRSQQ----R 54
SHC-Streptomyces        ---------------------------------------ERI-TRATARFIRAQQ----R 48
SHC-Hyphomicrobium      ---------------------------------------DTVLEEKIARYLRRIQ----S 50
SHC-Leptospirillum      ---------------------------------------DPVRRDKIVRAILSVQ----G 19
Naegleria               -----------------------------------------PFEYEIVRYILSKQ----N 59
Symbiodinium            ------------------------------------------------------------ 0
Prorocentrum            ------------------------------------------------------------ 0
Gymnodinium             ------------------------------------------------------------ 0
Alexandrium             -----------------------------------------AHCRAIEAYLRNHQ----Q 62
Dinophysis              -----------------------------------------PYWRAAEAYLRNHQ----Q 65
Scrippsiella            -----------------------------------------PYWSAAEVYLRNHQ----Q 63
Aureococcus             -----------------------------------------HQRRAMVRYYENHQ----Q 61
Phaeodactylum           -----------------------------------------AQTALMKHYLIVHQ----Q 57
Helicotheca             -----------------------------------------HQREMMMHYIAVHQ----Q 63
Yeast-erg7              ---------------------------------------PEHERIELIRYIVNTA----H 137
Candida                 ---------------------------------------PEPYRVEMIRYIVNTA----H 60
Verrucomicrobia         ---------------------------------------TEHDQPRFVAGLLQPQ----L 28
Eudoraea                ---------------------------------------SVETKTKMSRYILRHQ----L 49
Plesiocystis            ---------------------------------------PAERRRNIRRQLEVTR----K 58
Enhygromyxa             ---------------------------------------PEPRKQRIRLSLELQR----K 49
Bodo                    -----------------------------------------QQRTEFIRYLRNMQ----N 65
Trypanosoma             ---------------------------------NDEPCRCGEAERVEMIRYLRNYM---N 80
Phytomonas              -------------------------------------------RLELIRYIRNYQ----N 13
Leishmania              KLASSSSDT-KQRSSCGDYPERVLGVPAAENAGEERCQCGEAMRQELIRYIRNHQ----N 111
Leptomonas              KLSGKRRKAQNGNASMGDYAERVLGVPAAENGGEEPCRCGEATRQELIRYLRNYQ----N 112
Labilithrix             ---------------------------------------SETQRARIIAYFERAQ----N 49
Stigmatella             ---------------------------------------EPEQRDGLIAYLRNHQ----N 22
Myxococcus              ---------------------------------------DPGHRDGLLANIRAHQ----N 49
Corallococcus           ---------------------------------------EASVRDGMVAHMRAHQ----N 58
S-cinnamoneus           ---------------------------------------DETSRQGMLTYFRNTR----T 73
S-caatingaensis         ---------------------------------------DETTRRGIVQHFRRTR----R 49
Sandaracinus            ---------------------------------------SAERRASVLKHFERTR----L 58
S-alboviridis           ---------------------------------------PPGEIGPIVQHYRVKR----L 50
G-obscuriglobus         ---------------------------------------SDADAAKIIRHFEFSQ----L 65
G-sp.SH-PL17            ---------------------------------------SAEDTAKILTHFERTQ----T 39
G-sp-IIL30              ---------------------------------------SAEDTAKILTHFERTQ----T 58
Methylococcus           ---------------------------------------DARERAAIIRHFELSQ----L 56
Methylocaldum           ---------------------------------------GDAEKARIILYFEKSQ----R 58


Helobdella              -REGGWGLHTEA-P------------------------------------PTVLGTALNY 81
ScLDS                   -KEGGWGLHTED-K------------------------------------GTVFGTALNY 81
AjLDS-b                 -KDGGWSLHIEG-D------------------------------------ATVFGTALNY 158
AjLDS-a                 -KDGGWSLHIEG-D------------------------------------ATVFGTALNY 158
PpLDS                   -KDGGWSLHIEG-D------------------------------------ATVFGTALNY 158
ScPS                    -KEGGWGLHTED-K------------------------------------GTVFGTALNY 81
PpPS                    -ADGGWGLHTED-N------------------------------------ATVFGTALNY 150
AjPS-b                  -ADGGWGLHTED-N------------------------------------ATVFGTALNY 150
AjPS-a                  -ADGGWGLHTED-N------------------------------------ATVFGTALNY 150
Capsaspora              -AEGGWGLHTAD-K------------------------------------ATVFGTGLNY 82
Saccoglossus-LSS        --------HLDK-V------------------------------------TLLFGTSLNY 150
EpLSS                   -PDGGWGLHIED-H------------------------------------ATVFGTAMNY 156
SpLSS                   -PDGGWGLHIED-H------------------------------------ATVFGTAMNY 156
SgLSS                   -PDGGWGLHIED-H------------------------------------PTVFGTAMNY 156
LspLSS                  -QDGGWGLHVEG-P------------------------------------PTVFGCATNY 156
MgLSS                   ------------------------------------------------------------ 0
AaLSS                   ------------------------------------------------------------ 0
AfLSS                   -QDGGWGLHIEG-P------------------------------------PTVFGCATNY 156
ArLSS                   -QDGGWGLHVEG-P------------------------------------PTVFGCATNY 156
HspLSS                  -QDGGWGLHIEG-P------------------------------------PTVFGCATNY 156
EsLSS                   -QDGGWGLHIEG-P------------------------------------PTVFGCATNY 156
ApLSS                   -PDGGWGLHIEG-P------------------------------------ATVFGCATNY 156
PpLSS                   -QDGGWGLHIEG-P------------------------------------ATVFGCATNY 156
PmLSS                   -QDGGWGLHIEG-P------------------------------------PTVFGCATNY 156
```

```
Amphimedon               -PDGGWGLHIAG-K------------------------------------STVFGTALNY 79
Aplysia                  -PDGGWGLHIEG-P------------------------------------PTVFGCALNY 82
Capitella                -PDGGWGLHTEG-P------------------------------------PTVFGCALNY 82
Branchiostoma-LSS        -PDGGWGLHIED-H------------------------------------STVFGTALNY 158
Lingula                  -PDGGWGLHIEG-P------------------------------------PTVFGCALNY 82
Callorhinchus            -LDGGWGLHVED-S------------------------------------STVLGTALNY 81
HumanLSS                 -PDGGWGLHIED-K------------------------------------STVFGTALNY 158
Pteropus                 -PDGGWGLHIED-K------------------------------------STVFGTALNY 82
DreLSS                   -PDGGWGLHIED-K------------------------------------STVFGTALSY 159
Haplochromis             -PDGGWGLHVED-K------------------------------------STVFGTALSY 82
Xenopus                  -PDGGWGLHIED-K------------------------------------STVFGTALSY 82
Charadrius               -PDGGWGLHVED-K------------------------------------STVFGTALNY 78
Anolis                   -PDGGWGLHIED-K------------------------------------STVFSIALNY 82
Alligator                -PDGGWGLHVED-K------------------------------------STVFGTALNY 82
Pneumocystis             PKDGGWGIHIEG-K------------------------------------STVFGTALNY 83
Arthrobotrys_            PEDGGWGLHTEG-E------------------------------------SSVFGTTINY 83
Aspergillus              PEDGGWGLHIEA-H------------------------------------SSVFGTCMNY 83
Spizellomyces            -EDGGWGIHVEG-R------------------------------------STVFGTALNY 79
Trichosporon             -PEGGWGLHTAA-P------------------------------------PTVYGTVMNY 82
Puccinia                 VRDRGWGLHTSG-K------------------------------------STVFGTVLNY 87
Mixia                    PGDQGWGIHIEA-V------------------------------------SSVFGSVMNY 86
Microbotryum             PGDEGWGIHIEA-Q------------------------------------SSVFGTGLNY 86
Dacryopinax              AEDGGWGLHVEH-H------------------------------------STCFGTVMNY 83
Fistulina                PEDGGWGIHVEG-P------------------------------------STVFGTGLNY 83
Wallemia                 EVDGGWGIHIES-P------------------------------------STVFGTALNY 83
Ceraceosorus             -KDGGWGLHIAG-E------------------------------------STAFGTATNY 82
Ustilago                 KDDGGWGIHIEA-S------------------------------------STVFGTALNY 83
Malassezia_              PEDGGWGIHIEG-H------------------------------------STVFGTALNY 83
Fluviicola               -EDGGWGLHIEG-H------------------------------------STMFGTVMQY 81
Methylobacter            -QDAGWGMHIEG-Q------------------------------------STLFGTVMQY 81
Methylobactes            -QDAGWGMHIEG-Q------------------------------------STLFGTVMQY 81
Methylosarcina           -KDAGWGMHIEG-E------------------------------------STMFGTVMQY 81
Aphanomyces              -YDGGWGIHIEE-G------------------------------------STMFGTVLNY 80
Cystobacter              -KDGGFGLHVEA-S------------------------------------SYVFTSTLCY 79
Galdieria                -EDGGWGLHIEA-P------------------------------------SSMFGTAMNY 80
Selaginella              -EDGGWGLHIEG-H------------------------------------STMFGTVLVY 82
Panax                    -VDGGWGLHIEG-C------------------------------------STMLCTALNY 82
Physcomitrella           -KDGGWGLHIEG-H------------------------------------STMFGSVLTY 81
Cucumis                  -EDGGWGLHIEG-S------------------------------------STMFGSALNY 82
Adiantum                 -KDGGWGLHIEG-H------------------------------------STMFGSVLSY 82
Abies                    -EDGGWGLHIEG-H------------------------------------STMFGTVLNY 84
Malus                    -EDGGWGLHIEG-P------------------------------------STMFGTALNY 82
Amborella                -KDGGWGLHIEG-H------------------------------------STMFGTALTY 82
Nicotiana                -SDGGWGLHVES-P------------------------------------STMFGSVLSY 82
AtCAS1                   -EDGGWGLHIEG-P------------------------------------STMFGSVLNY 181
Glycine                  -KDGGWGLHIEG-P------------------------------------STMFGSVLSY 82
Coffea                   -PDGGWGLHIEG-P------------------------------------STMFGSALNY 82
Populus                  -RDGGWGLHIEG-P------------------------------------STMFGSVLNY 82
Vitis                    -KDGGWGLHIEG-P------------------------------------STMFGTVLNY 82
Micromonas               -EDGGFGLHIEG-S------------------------------------STMFGTTLSY 82
Volvox                   -DDGGFGLHIEG-G------------------------------------STMFGTGLNY 85
Chlorella                -ADGGYGLHIEG-T------------------------------------STMFGTVLSY 83
Synchroma                -SDGGWGTHIEG-A------------------------------------STMFGTVLSY 82
Chattonella              -TDGGWGTHIES-A------------------------------------STMFGTVLSY 80
Ochromonas               -KDGGWGTHIEC-A------------------------------------STMFGTVLNY 82
Chromulina               -EDGGWGTHIEC-A------------------------------------STMFGTILSY 85
Chondrus                 P-DGGFGLHIEH-K------------------------------------STMFSTALNY 80
Acanthamoeba             -ADGGWGLHIES-P------------------------------------STIFGTGMNY 82
Dictyostelium            PVDGGWGLHIEA-H------------------------------------SDIFGTTLQY 77
Polysphondylium          PKDGGWGLHIEA-H------------------------------------SDIFGTALQY 80
Acytostelium             PKDGGWGLHIEA-H------------------------------------SDIFGTALQY 77
SHC-Candidatus           -PDGGFNIYAGG-P------------------------------------SEVSATIKAY 28
SHC-Chlorogloeopsis      -QHGGWELFYGD-G------------------------------------GELSTSVEAY 76
SHC-Streptomyces         -PDGTWATFHGG-P------------------------------------GELSTTIEAY 70
SHC-Hyphomicrobium       -DDGGWPLFHDG-V------------------------------------SNISAAVKAY 72
SHC-Leptospirillum       -KEGAWPLFHDG-D------------------------------------PDISATVKAY 41
Naegleria                -QDGGYGLHIEG-H------------------------------------STIFGTVLNY 81
Symbiodinium             ------GTHIES-P------------------------------------STMFGTVLNY 17
Prorocentrum             ----------------------------------------------------LFGSVLNY 8
Gymnodinium              ------------------------------------------------------------ 0
Alexandrium              -EDGGWGTHIES-P------------------------------------STMFGSVLNY 84
Dinophysis               -ADGGWGTHLEA-P------------------------------------STMFCSVLNY 87
Scrippsiella             -ADGGWGTHLES-P------------------------------------STMFCSALNY 85
Aureococcus              -TDGGWGTHVES-P------------------------------------STMFGSVLTY 83
Phaeodactylum            -ADGGWGTHVES-P------------------------------------STMFGTTLSY 79
Helicotheca              -SDGGWGTHIES-P------------------------------------STMFGTVLNY 85
Yeast-erg7               PVDGGWGLHSVD-K------------------------------------STVFGTVLNY 160
Candida                  PVDGGWGLHSVD-K------------------------------------STCFGTTMNY 83
Verrucomicrobia          -PDGSVGLHEESVR------------------------------------GAVFTSAISY 51
Eudoraea                 -DNGSFGIHQESKS------------------------------------GTVFTSVINY 72
Plesiocystis             -RDGGWGMHPDPPEREPKPEPAFEPGPAPVSEHDSPDAEAPAETMAGKFDSWMFHTVLGY 117
Enhygromyxa              -RDGGWGMHPDVPPAP-------------GDHDHHPNHDRDPAGLAGAHDSWMFHTVLGY 95
Bodo                     -PDGGWGMHTES-H------------------------------------STMFGSVLNF 87
Trypanosoma              -KDGGFGQHTEG-H------------------------------------STMLGTALNY 102
Phytomonas               -DDGGWGQHTEG-H------------------------------------STMLGTVLNY 35
Leishmania               -LDGGWGQHTEG-H------------------------------------STMMGTVLNY 133
Leptomonas               -QDGGWGQHTEG-H------------------------------------STMLGTVLNY 134
Labilithrix              -EDGSVGVHAEDTH------------------------------------GSLFCSVLAY 72
```

```
Stigmatella            -ADGGWGLDVEA-P------------------------------------SQVFTSVLNY 44
Myxococcus             -ADGGWGLSPGA-P------------------------------------SQVFTSVLNY 71
Corallococcus          -ADGGWGLDVES-P------------------------------------SLVFTSVLNY 80
S-cinnamoneus          -DQGGWGMHPAGPP------------------------------------S-PYATLLAY 95
S-caatingaensis        -PGGGWALHPQGPP------------------------------------S-PYATTLAY 71
Sandaracinus           -ADGTWGLHEKSEP------------------------------------Y-LFVTLLVF 80
S-alboviridis          -RQTGWGLHAASAG------------------------------------PSPYCTSLAY 73
G-obscuriglobus        -PNGAFGLHPEHPG------------------------------------S-VFVTTLVY 87
G-sp.SH-PL17           -SDGAFGLHLEHSG------------------------------------S-VFVTSLVY 61
G-sp-IIL30             -SDGAFGLHLEHSG------------------------------------S-VFVTSLVY 80
Methylococcus          -ADGAWGMHPESRG------------------------------------Y-VFFTVLAY 78
Methylocaldum          -DDGSWGMHPESQG------------------------------------Y-VFFTTLGY 80


Helobdella             VTMRLLGVSA--------R-D----------LDLVR-ALGILR--SLGGTLS-IPSWGKF 118
ScLDS                  VTMRLLGVGP--------E-D----------EDLQR-ARKFLH--YHGGAAS-IPSWGKF 118
AjLDS-b                ISMRLLGVSP--------E-D----------GDMKR-ARKVLH--HHGGASA-IPSWGKF 195
AjLDS-a                ISMRLLGVSP--------E-D----------GDMKR-ARKVLH--HHGGASA-IPSWGKF 195
PpLDS                  ISMRLLGVSP--------E-D----------GDLKR-ARKVLH--HHGGAAA-IPSWGKF 195
ScPS                   VTMRLLGVGP--------E-D----------EDLQR-ARKFLH--YHGGAAS-IPSWGKF 118
PpPS                   VVMRLLGVSP--------E-D----------QDLVK-ARKLLH--HHGGAAT-IPSWGKF 187
AjPS-b                 VVMRLLGVSP--------E-D----------QDLVK-ARKLLH--HHGGAAT-IPSWGKF 187
AjPS-a                 VVMRLLGVSP--------E-D----------QDLVK-ARKLLH--HHGGAAT-IPSWGKF 187
Capsaspora             VAFRLLGGSR--------D-D----------PLAVK-ARTFLH--KHDGVLG-IPSWGKF 119
Saccoglossus-LSS       TAMRVLGVGS--------D-D----------PDLVK-ARNILH--SLGGAVY-IPSWGKF 187
EpLSS                  VTMRLLGVSK--------D-D----------KDLKK-ARKLLM--EMGGAES-IPSWGKF 193
SpLSS                  VTMRLLGVSK--------D-D----------EDLKK-ARKLLL--EMGGAES-IPSWGKF 193
SgLSS                  VTMRLLGVSK--------D-D----------TDLKK-ARKLLM--EMGGAES-IPSWGKF 193
LspLSS                 VAMRLLGIGP--------E-D----------PDLMK-CRKLLH--SLGGAAA-IPSWGKF 193
MgLSS                  ------------------------------------------------------------ 0
AaLSS                  ------------------------------------------------------------ 0
AfLSS                  VAMRLLGIGP--------E-D----------PDLMK-CRKLLH--SLGGAAA-IPSWGKF 193
ArLSS                  VAMRLLGIGP--------E-D----------PDLMK-CRKLLH--SLGGAAA-IPSWGKF 193
HspLSS                 VAMRLLDVSP--------D-D----------PDLMK-CRKLLH--SLGGAVA-IPSWGKF 193
EsLSS                  VAMRLLDVPP--------D-D----------PDMVK-CRKLLH--SLGGAVA-IPSWGKF 193
ApLSS                  VAMRLLGVSA--------D-D----------PDLVK-CRKLLH--SLGGAAA-IPSWGKF 193
PpLSS                  VAMRLLGVAA--------D-D----------PDLIK-CRKLLH--SLGGAAA-IPSWGKF 193
PmLSS                  VAMRLLGVAA--------D-D----------PDLIK-CRKLLH--SLGGAAA-IPSWGKF 193
Amphimedon             VSLRLLGVGP--------D-D----------NELIK-ARELLH--QMGGAVC-IPSWGKF 116
Aplysia                AALRMLGLPA--------D-D----------PDLVR-ARDLLH--KLGGASA-IPSWGKF 119
Capitella              ICLRLLGLPP--------D-D----------PVLIR-ARALLH--KLGGATG-IPSWGKF 119
Branchiostoma-LSS      VTLRLLGVPA--------D-D----------KDVVR-ARNCLH--AKGR----------- 185
Lingula                VVMRLLGVGP--------E-D----------RDLVK-ARRLLH--RLGGAAA-IPSWGKF 119
Callorhinchus          TSLRLLGVGA--------D-D----------PDLVR-ARNNLH--TKGGAVG-IPSWGKF 118
HumanLSS               VSLRILGVGP--------D-D----------PDLVR-ARNILH--KKGGAVA-IPSWGKF 195
Pteropus               VSLRILGVGP--------D-D----------PDLAR-ARNVLH--EKGGAVA-IPSWGKF 119
DreLSS                 TTLRILGVGP--------D-D----------PDMVR-ARNALH--NRGGAVG-IPSWGKF 196
Haplochromis           TSLRILGVDP--------D-D----------PDMVR-ARNNLH--SKGGAVG-IPSWGKF 119
Xenopus                TSLRLLGVSQ--------D-D----------LDLTR-ARNNLL--SKGGAVG-IPSWGKF 119
Charadrius             IALRILGLGP--------D-D----------PDIVR-ARVNLH--SKGGAVG-IPSWGKF 115
Anolis                 TAMRILGVSP--------D-D----------PDLVR-ARNNLH--SKGGAVK-IPSWGKF 119
Alligator              TSLRILGVGP--------D-D----------PDLVR-ARINLH--SKGGAVG-IPSWGKF 119
Pneumocystis           VVLRILGLGP--------D-H----------PVTMK-ARIKLN--ELGGAIG-CPQWGKF 120
Arthrobotrys_          TTLRLLGMPA--------D-H----------PVAVK-ARQKLH--EMGGALG-VPHWGKA 120
Aspergillus            VALRLIGVSE--------D-D----------PRMIK-ARGLLH--KFGGAIY-GPHWAKF 120
Spizellomyces          VTCRILGVER--------D-H----------PAMIK-ARGTLH--RLGSATA-IPAWGKF 116
Trichosporon           VCLRMLGMGP--------D-E----------GPMTA-IRAKIH--EFGGAVA-IPTWGKR 119
Puccinia               VACRLLGIDA--------E-H----------PMLVR-ARATLH--ALGGATG-VPTWGKV 124
Mixia                  VALRLLGVDA--------E-E----------PMMQR-ARATFL--HHGGASF-IPSWGKF 123
Microbotryum           VTLRLLGVDA--------E-E----------PMMIR-ARATLH--HLGGCTG-IPSWGKF 123
Dacryopinax            VALRLLGVPP--------E-H----------PVCVK-ARGTMH--SFGGAAA-LPQWGKW 120
Fistulina              TAIRILGMDA--------D-H----------PVCLK-ARATLH--KLGGAAG-IPAWGKF 120
Wallemia               TALRLLGVEA--------D-H----------PVMTK-ARGTLH--KLGGATG-SPAWGKF 120
Ceraceosorus           TVCRLLGMEA--------D-H----------PAMIR-ARGVLH--KLGGARG-VPAWGKL 119
Ustilago               TLLRLIRVSA--------D-H----------PMMVK-ARGTLH--KLGGATG-IPSWGKL 120
Malassezia_            VVLRLVGVPA--------E-H----------PMMVQ-ARGTLW--KLGGAAG-IPSWGKL 120
Fluviicola             VTLRILGEDL--------S-N----------PQMKR-AQDWIL--RHGGAIK-VPQWGKF 118
Methylobacter          VSLRLLGVDK--------D-H----------QQLIK-ARAWIK--NNGGATG-IPSWGKF 118
Methylobactes          VSLRLLGVDK--------D-H----------QQLIK-ARAWIK--NNGGATG-IPSWGKF 118
Methylosarcina         VSLRLLGVDK--------N-H----------QQLVE-ARNWIK--SHGGATG-IPSWGKF 118
Aphanomyces            VALRLLGAAA--------D-D----------EACLE-ARTFIK--HHGGATL-VPSWGKF 117
Cystobacter            VALRLLGVSA--------E-D----------PAATS-ARQWIL--AHGGALT-SAPWGKF 116
Galdieria              VALRILGVDR--------D-D----------SAAIA-ARNWIL--QRGGALG-IPSWGKL 117
Selaginella            VSLRLLGQEP--------D-K----------PAMKS-ALSWIF--GHGGTIA-IPSWGKF 119
Panax                  ITLRLLIRGD--------E-EEEIRDEAANGGSLEK-ARRWII--DHGGATY-IPSWGKF 129
Physcomitrella         VTLRILGDGP--------E-G-------GDFDALRR-GRKWIL--DHGGATY-ITSWGKF 121
Cucumis                VALRLLGEAA--------D-G-------GEHGAMTK-ARSWIL--ERGGATA-ITSWGKL 122
Adiantum               VTLRLLGQEL--------S-D-------GEDQAMER-GRAWIL--QHGGATT-IPSWGKF 122
Abies                  VTLRLLGQAP--------D-G-------G-QGAMEK-GCAWIL--DHGGATA-IPSWGKM 123
Malus                  VTLRLLGEGA--------D-D-------G-QGAIEL-ARKWIL--DHGGVTA-ITSWGKM 121
Amborella              VTLRLLGEGP--------E-D-------GEDGAMEK-GRKWVL--DHGGLTA-ISSWGKM 122
Nicotiana              VTLRLLGEGT--------N-G-------G-EGAMEK-GRKWIL--DHGSATA-ITSWGKM 121
AtCAS1                 VTLRLLGEGP--------N-D-------G-DGDMEK-GRDWIL--NHGGATN-ITSWGKM 220
Glycine                ITLRLLGEGP--------N-D-------G-QGEMEK-ARDWIL--GHGGATY-ITSWGKM 121
Coffea                 VTLRLLGEGP--------N-D-------G-DGAMEK-GRKWIL--DHGGATA-ITSWGKM 121
Populus                VTLRLLGEGP--------N-D-------G-DGAMDK-GHDWIL--NHGSATM-ITSWGKM 121
Vitis                  VTLRLLGEGA--------N-D-------A-DGAMEK-GRDWIL--NHGGATA-ITSWGKM 121
Micromonas             VSMRLLGMTA--------T-T----------EAVVN-ARSWIL--SRGGAIN-VPSWGKF 119
```

```
Volvox                  VMARLLGMGP--------D-E----------DLTRR-AREWVN--SRPRGGYLHHQLGQV 123
Chlorella               VTLRLLGVGP--------D-D----------ATLAP-ARTWIH--ERGGAHA-ITSWGKF 120
Synchroma               VALRLLGEGP--------D-A----------PHMAA-ARAFIH--ENGGALY-TASWAKF 119
Chattonella             VALRLLGVKA--------Y-D----------IGACR-AREFIH--EHGGALY-TPSWGKF 117
Ochromonas              VALRLLGDEA--------D-A----------PHMEK-ARSFIH--RYGGALY-APSWAKF 119
Chromulina              ISLRLLGISS--------N-E----------DFMKL-GRKFLL--DHGGARY-APSWAKF 122
Chondrus                VAMRILGAGS--------A-D----------SDSRS-ARAWIR--AHGGPAG-CPGWGKF 117
Acanthamoeba            TAMRLLGVPM--------E-D----------DRMKR-AREFLA--ANDGCKG-IPSWGKF 119
Dictyostelium           VSLRLLGVPA--------D-H----------PSVVK-ARTFLL--QNGGATG-IPSWGKF 114
Polysphondylium         VSLRLLGLPV--------D-H----------PGVER-ARKFLR--DNGGATG-IPSWGKF 117
Acytostelium            VSLRILGLPA--------A-H----------PGVTR-ARDFLR--ANGGAVG-IPSWGKF 114
SHC-Candidatus          CALKLAGLDP--------H-S----------PPLRR-ARERIL--ALGGLQA-ANSYVKI 65
SHC-Chlorogloeopsis     MALKLLGVPQ--------N-D----------PAMIK-AREFIL--ERGGISK-TRIFTKL 113
SHC-Streptomyces        VALRLAGDAP--------E-D----------AHMAA-ASAWVR--EQGGIAA-SRVFTRI 107
SHC-Hyphomicrobium      YALKMIGDSP--------D-A----------PHMKK-ARAWIL--AQGGASH-SNVFTRN 109
SHC-Leptospirillum      QALKLCGFDP--------S-H----------PALVR-AREWVL--SQGGAGK-VNVFTRI 78
Naegleria               VALRLLGVSP--------DH------------SCMKLTLEFLSKEPANGALG-APQWAKL 120
Symbiodinium            VALRLVGVDA--------K-T----------LECEK-GREFMR--QHGGALY-APSWAKF 54
Prorocentrum            VALRLVGVPP--------S-D----------PACVE-GRRFLR--QHGGALF-APSWAKF 45
Gymnodinium             -------------------------------------------------ALY-APSWAKC 10
Alexandrium             TALRLIGVPE--------E-D----------AACIQ-ARNFLA--KHGGALH-APSWAKF 121
Dinophysis              VALRLVGAPP--------D-D----------AACVR-GRGFLH--EHGGALY-TAPWTKF 124
Scrippsiella            VALRLVGAPA--------D-D----------PACVK-GRAFIH--KHGGALY-TAPWTKF 122
Aureococcus             VALRLLGEPA--------D-A----------PACAA-GRKLIL--EQGGACY-TSSWAKF 120
Phaeodactylum           VALRLLGMDA--------EE-----------PVCQR-GRAFIR--EQGGAVM-TSSWAKL 116
Helicotheca             VALRLLDRDE--------THK----------SKYEK-AVSFIS--AQGGAIM-TSSWAKF 123
Yeast-erg7              VILRLLGLPK--------D-H----------PVCAK-ARSTLL-R-LGGAIG-SPHWGKI 197
Candida                 VCLRLLGMEK--------D-H----------PVLVK-ARKTLH-R-LGGAIK-NPHWGKA 120
Verrucomicrobia         VALRLLGEKP--------S-R----------PELAK-MRDWIE-K-AGTPVK-AAAWGKF 88
Eudoraea                VALRFLDHSA--------S-E----------PELKK-ALNWIH-T-KGGPLY-SASWCKF 109
Plesiocystis            VALRLLGDAHEPGEDPQRT-A----------AMLEE-SLAWIH-A-HGGPER-APTWGRI 162
Enhygromyxa             VALRLLGADP--------N-E----------PLLQD-VRAWIH-A-HGGPYG-VPTWGRA 132
Bodo                    VSLRLLGVDA--------N-D----------SAAKA-GSRWIL-E-RGGALS-IPSWGKV 124
Trypanosoma             VALRFMGVPA--------D-D----------ADATR-ARAWIR-S-HGGAVS-VPTWGKV 139
Phytomonas              IALRMMGVAA--------A-D----------LSMAS-ARRWIH-A-EGGAVY-TPLWGRI 72
Leishmania              VSLRLLGVPA--------S-D----------PQATC-GRSWIL-A-HGGATT-TPMWGRV 170
Leptomonas              VSMRLLGVPA--------K-D----------PQAAS-ARDWIH-A-HGGATT-TPMWGRV 171
Labilithrix             VALRMLEVPK--------D-D----------ARVRR-MLAFIH-R-HGSPLA-AAQWAKV 109
Stigmatella             VALRLLGVGK--------D-D----------AGLRR-ARQWFL-P-RGGPLG-SGAWGKI 81
Myxococcus              VAQRLLGVDA--------K-D----------ADLAR-ARAWFL-P-RGGPLG-SGSWGKA 108
Corallococcus           VAQRLLGVGA--------D-D----------PGLVR-ARAWFL-P-RGGPLS-SASWGKF 117
S-cinnamoneus           VALRLLGVGP--------E-E----------SPAAD-ARKWLG-TQPGGARS-VPQWGMF 133
S-caatingaensis         LALRLLGTAP--------A-D----------PLAAE-AAAWLR-SLPG-APA-LPPWGRF 108
Sandaracinus            VACRLLGLAK--------D-D----------PLIAR-AHEFIR-R-EGGAVA-VPSWGKL 117
S-alboviridis           IALRLLGLAP--------D-H----------PLCRP-ARQWLR-TQPGGVAA-IPSWGKF 111
G-obscuriglobus         VAARCLGVSA--------E-H----------AVTAK-ARGWLH-AQPGGVLS-APTWGKF 125
G-sp.SH-PL17            VAMRCLGVSA--------D-H----------PLAVR-ARNWLH-AQPGGILS-APTWGKF 99
G-sp-IIL30              VAMRCLGVSA--------D-H----------PLAVR-ARNWLH-AQPGGILS-APTWGKF 118
Methylococcus           VALRLLGLGP--------E-T----------SMLAR-ARAWLH-AQPEGVKA-VPTWGKF 116
Methylocaldum           VALRLIGVPA--------T-S----------PMLVM-ARRWLH-SQPYGVKG-IPTWGKF 118


Helobdella              WLAVLNVYSWDGLHSLAPELWSLPE-C-----LPF-HPSKMWCHCRQVYLGMSYCYAVRF 171
ScLDS                   WLCILNCYRWEGMHTLFPELWLLPS-W-----IPA-HPSTLWIHCRMVYIGMAFLYGKRY 171
AjLDS-b                 WLCILNCYKWEGMHTMFPELWLMPS-W-----IPA-HPSTLWIHCRMVYIGMAFLYGKRY 248
AjLDS-a                 WLCILNCYKWEGMHTMFPELWLMPS-W-----IPA-HPSTLWIHCRMVYIGMAFLYGKRY 248
PpLDS                   WLCILNCYKWEGMHTMFPELWLMPS-W-----IPA-HPSTLWIHCRMVYIGMAFLYGKRY 248
ScPS                    WLCILNCYRWEGMHTLFPELWLLPT-W-----VPA-HPSSLWVHCRAVYIGMAYLYGKHF 171
PpPS                    WLCILNCYKWEGMHTLFPELWLLPS-W-----LPA-HPSTLWVHCRIVYLGMSYLYGKRY 240
AjPS-b                  WLCILNCYKWEGMHTLFPELWLLPS-W-----LPA-HPSTLWVHCRIVYLGMSYLYGKRY 240
AjPS-a                  WLCILNCYKWEGMHTLFPELWLLPS-W-----LPA-HPSTLWVHCRIVYLGMSYLYGKRY 240
Capsaspora              WLALLNVYDYDGMNAVPPELWLLPE-W-----APL-HPKYMWCHCRQVYLPMSYCYGVKL 172
Saccoglossus-LSS        WLAVLNCYSWDGMNTLLPEMWLFPK-W-----VPA-HPSTLWCHCRQVYLPMSYCYATRL 240
EpLSS                   WLCVLNLYKWEGMHCLFPEIWLFPS-W-----MPA-HPSSIWCHCRQVYLPMGYFYGAKF 246
SpLSS                   WLCVLNLYKWEGMHCLFPEIWLFPT-W-----LPA-HPSTIWCHCRQVYLPMGFFYGIKF 246
SgLSS                   WLCVLNLYKWEGMHCLFPEIWLFPE-W-----FPA-HPSSIWCHCRQVYLPMGFFYGIKF 246
LspLSS                  WLSVLNVYEWDGMHTLFPEIWNFPT-F-----FPA-HPSTIWCHCRQVYLPMSYCYGIKF 246
MgLSS                   ------------------------------------HPSTIWCHCRQVYLPMSYCYGIKF 24
AaLSS                   ------------------------------------------------------------ 0
AfLSS                   WLSVLNVYQWDGMHTLFPELWNFPT-Y-----TPA-HPSTIWCHCRQVYLPMSYCYGIKF 246
ArLSS                   WLSVLNVYQWDGMHTLFPELWNFPT-Y-----TPA-HPSTIWCHCRQVYLPMSYCYGIKF 246
HspLSS                  WLSVLNVYSWDGMHTLFPELWLFPQ-W-----IPA-HPSTIWCHCRQVYLPMSYCYGRKF 246
EsLSS                   WLSVLNVYSWDGMHTLFPELWLFPQ-W-----IPA-HPSTLWCHCRQVYLPMSYCYGRKF 246
ApLSS                   WLSVLNVYSWEGMHTLFPELWLFPT-W-----IPA-HPSTIWCHCRQVYLPMSYCYGVKY 246
PpLSS                   WLSVLNVYSWDGMHTLFPELWLFPT-W-----IPA-HPSTIWCHCRQVYLPMSYCYGIKY 246
PmLSS                   WLSVLNVYSWDGMHTLFPELWLFPT-W-----IPA-HPSTIWCHCRQVYLPMSYCYGIKY 246
Amphimedon              WLSVLNVYDWSGVHTLLPELWILPQ-C-----VPV-HPSKMWCHCRQVYLPMGFVYSKRI 169
Aplysia                 WLAVLNCYSWEGLNSLFPEMWILPT-W-----LPF-HPSKLWCHCRQVYLPMAYCFGRRI 172
Capitella               WLAVLNVYKWEGIHNLFPEIWTLPE-W-----LPI-HPSHMWCHCRQVYLPMAYCYGARI 172
Branchiostoma-LSS       ---------------------LLPS-W-----TPA-HPSTLWCHCRQVYLPMSYCYAVQL 217
Lingula                 WLAVMNLYSWEGMHSLFPEMWLFPT-W-----IPI-HPSKLWCHCRQVYLPMAYCYGVRL 172
Callorhinchus           WLAVLNVYSWEGLNSLFPEMWLLPR-W-----MPA-HPSTLWCHCRQVYLPMSYCFAVRL 171
HumanLSS                WLAVLNVYSWEGLNTLFPEMWLFPD-W-----APA-HPSTLWCHCRQVYLPMSYCYAVRL 248
Pteropus                WLAVLNVYSWEGLHTVLPEMWLLPS-W-----VPA-HPSTIWCHCRMVYLPMSYCYATRL 172
DreLSS                  WLAILNVYSWEGMNTLFPEMWLLPS-W-----MPA-HPSTLWCHCRQVYLPMSYCYAVRL 249
Haplochromis            WLAILNVYSWEGMNTLLPEMWLFPT-W-----MPA-HPSTLWCHCRQVYLPMSYCYAVRL 172
Xenopus                 WLAVLNVYSWEGMNTLFPEMWLLPH-W-----FPA-HPSTLWCHCRQVYLPMSYCYATRL 172
Charadrius              WLAVLNVYSWEGMNTLLPEMWLLPT-W-----FPA-HPSRLWCHCRQVYLPMSYCYARRL 168
```

```
Anolis                    WLAVLNVYSWEGMNTLFPEMWLFPS-W-----MPA-HPSTLWCHCRQVYLPMAYCYGRRL 172
Alligator                 WLAVLNVYSWEGMNTLFPEMWLLPA-W-----FPA-HPSTLWCHCRQVYLPMSYCYASRL 172
Pneumocystis              WLAVLNCYSWEGINPILPEFWMLPE-W-----FPI-HPSRWWVHTRAVYLPMGYIYGEKF 173
Arthrobotrys_             WLAMIGCFEWEGMNPIPPELWLLPD-W-----IPL-HPYRWWIHTRAVYLPMSYIWSKRH 173
Aspergillus               WLSVLGVMEWECVNPVPPELWLLPD-W-----VPF-TPWRWWIHIRQVFLPMSYLWSKKF 173
Spizellomyces             WLSVLGVYSYEGMNPVPPELWLLPY-A-----LPI-HPGKWWIHTRMVYLPMGFIYGQRL 169
Trichosporon              R---------------ANNRCLPD-W-----VPF-APWRWWIHVRLVFTPMAWLWGARF 156
Puccinia                  WLALLGVYDWEGVNPVPPELWVLPE-M-----LPF-HPWRWWVHSRQVYLPISYLCGKRL 177
Mixia                     WLSVLNVHEWQGFNPTPPELWLLPT-W-----LPM-HPSKWWVHTRAVYIPFGYLSGKQF 176
Microbotryum              WLAILNVHSWSGLNPTPTELWLLPE-F-----LPI-HPYRWWIHTRNVYIPMGYLNGRYF 176
Dacryopinax               WLCLLNCYDWAGCIAIPAELWLFPQ-F-----LPI-HPWRWWIHTRNVYIPMGFLFARKW 173
Fistulina                 WLSLLNCYEWAGNNPVPPELWVLPE-F-----LPI-HPHRWWIHTRAVYLPMSYLYGVRF 173
Wallemia                  WLACLGVYDWDGVNPVPPELWLLPD-W-----LPI-HPTKWWIHCRQIYLPMSYLYGKRF 173
Ceraceosorus              WLAVLNVYDWEGMHPIPPELWCLPD-W-----IPF-HPWRWWIHTRMVYLPMGYLWAKRF 172
Ustilago                  WLAILNCYEWEGMNPIPPELWLLPD-W-----LPI-HPWRWWIHTRMVYIPMGYLWGRRF 173
Malassezia_               WLAVLNVYDWEGLNPIPPELWVLPD-F-----VPI-HPWRWWVHTRMVYLPMGFLYGKRF 173
Fluviicola                YLSVLNVYDWKGNDALLPEMWKFPK-W-----VPF-HPGKYWCHNRMIFLPMTYCFGKKV 171
Methylobacter             YLAVLNCYDWQGFNSLFPEMWLFPK-W-----LPV-HPWRYWCHTRMVYLPMAYCYAQRI 171
Methylobactes             YLAVLNCYDWQGFNSLFPEMWLFPK-W-----LPV-HPWRYWCHTRMVYLPMAYCYAQRI 171
Methylosarcina            YLAVLNLYDWQGFNSLFPEMWLFPK-W-----LPV-HPWRYWCHTRMVYLPMAYCYAQRI 171
Aphanomyces               WLAVLNVYDWRGVDALPPEMWLLPR-W-----LPF-HPGRTWVHCRMVYLPMSYLYGIRF 170
Cystobacter               FLSVLRLHEYEGLDPLLPELWLLPE-A-----LPM-HPSRFWCHCRMVYLPMSWLYGKKA 169
Galdieria                 YLAVLGLYHWDGLNPLTPEMWILPY-W-----VPI-HPGRFWCHCRIVYLPMSYLYGRRA 170
Selaginella               WLAVLGVFDWSGVNPLPPEMYLLPY-A-----IPL-HPGRMWCHCRMVYLPMSYIYGRRF 172
Panax                     WLSILGVYEWSGNNPLPPEMWLLPY-F-----LPL-HPGRMWNHCRMVYLPMSYLYGRRF 182
Physcomitrella            WLTVLGVFEWSGNNPLPPETWILPY-F-----LPM-HPGRMWCHCRMVYLPMSYIYGKRF 174
Cucumis                   WLSVLGVYEWSGNNPLPPEFWLLPY-S-----LPF-HPGRMWCHCRMVYLPMSYLYGKRF 175
Adiantum                  WLSVLGTFEWAGNNPLPPEIWLLPY-F-----LPI-HPGRMWCHCRMVYLPMCYIYGNRF 175
Abies                     WLSVLGVFDWTGNNPLPPEMWLLPY-F-----LPT-HPGRMWCHCRMVYLPMSYIYGRRF 176
Malus                     WLSVLGAYEWSGNNPLPPEVWLCPY-S-----LPF-HPGRMWCHCRMVYLPMSYLYGK-F 173
Amborella                 WLSVLGAFEWSGNNPLPPEIWLLPY-F-----LPL-HPGRMWCHCRMVYLPMSYLYGKKF 175
Nicotiana                 WLSVLGIFEWSGNNPLPPEIWLLPY-I-----LPF-HPGRMWCHCRMVYLPMSYLYGKRF 174
AtCAS1                    WLSVLGAFEWSGSNPLPPEIWLLPY-F-----LPI-HPGRMWCHCRMVYLPMSYLYGKRF 273
Glycine                   WLSVLGVYEWSGNNPLPPEIWLLPY-M-----LPF-HPGRMWCHCRMVYLPMSYLYGKRF 174
Coffea                    WLSVLGAYEWSGNNPLPPEIWLLPY-A-----LPV-HPGRMWCHCRMVYLPMSYLYGKRF 174
Populus                   WLSVLGVFEWSGNNPMPPEMWLLPY-L-----LPV-HPGRMWCHCRMVYLPMSYLYGKRF 174
Vitis                     WLSVLGVFEWSGNNPLPPEIWLLPY-I-----LPV-HPGRMWCHCRMVYLPMSYLYGKRF 174
Micromonas                YLCILGIYDWEGLNPVPPECWLLPY-EY----NPI-HPGRFWCHCRMVYLPMSYLYGCRA 173
Volvox                    LAGSARGVPWDGMNPLTPEMWLLAV-QQMDGHRLP-APGPL-----LVYLPMSYVYGMRG 176
Chlorella                 WLAVLGVYSWEGLNPLSPEMWLLPY-ASWTGIGWL-HPGRFWCHCRMVYLPMSYVYGVRG 178
Synchroma                 WLAVLGLYEWEGINSIPAEMWMLPR-A-----FPF-QPGRLWCHCRMVYLPMCYAYSSRF 172
Chattonella               WLAILGAMEWDCINSVPPEMWLLPR-W-----FPF-HPGKLWCHCRMVYLPMCFVYGKRF 170
Ochromonas                WLAVIGVYDWDAVNSIPVEMWLLPR-W-----FPF-HPGKLWCHCRMVYLPMGYLYSKRF 172
Chromulina                WLASIGVYDWKGINSVPVEMWLLPQ-W-----FPF-HPSKLWCHCRMVYLPMGYVYCKRF 175
Chondrus                  WLAVLGVYEWQGMDPLTPEFWLLPY-A-----LPC-HPARFWCHCRVVYLPMSYLYGRRA 170
Acanthamoeba              WLAVMGVYDWEGLHPIPPELWLLPY-M-----LPI-HPGRWWCHCRMVYLPMGYVYGRRI 172
Dictyostelium             WLATLNAYDWNGLNPIPIEFWLLPY-N-----LPI-APGRWWCHCRMVYLPMSYIYAKKT 167
Polysphondylium           WLATLNVYSWDGLNPIPIEFWLLPY-S-----VPI-CPGRWWCHCRMVYLPMSYLYARRT 170
Acytostelium              WLAVLNVYSWDGLNPIPIEFWLVPY-A-----FPI-CPGRWWCHCRMVYLPMSYLYARRT 167
SHC-Candidatus            NLSLFGLYPRKHVPSVPPEIVMLPG-N-----V----LYEMSSWTRSILVPLSIVQARGS 115
SHC-Chlorogloeopsis       HLALIGCYSWEGIPSLPPWVMLLPE-N-----FLF-NIYEMSSWARSSTVPLLIVCDRKP 166
SHC-Streptomyces          WLALFGWWRWDDLPEMPPEIMYLPK-W-----LPL-NIYDFGCWARQTIVPLTVVGAHRP 160
SHC-Hyphomicrobium        LLALFGSIPWSGVPVMPVEIMLLPR-W-----FPF-HIDKISYWARTVLIPLTVLNALKP 162
SHC-Leptospirillum        ALAIFGQYSWTKIPALPAEMVLLPS-W-----FPF-SIYSVSYWSRTVIVPLLFIYHHKP 131
Naegleria                 YLCCLGLMDWDCIDPIPCELWLLPD-W-----FPYIQPGKWWCHCRVVYLPMAYLYGIKK 174
Symbiodinium              WLAVLGVYEWKGIAPVPPEMWLLPS-W-----FPL-HPGRFWCHCRMVYLPMCWLYARRF 107
Prorocentrum              WLCVLGVYDYRGIAPVPPEMWLLPA-W-----FPL-HPGRFWCHCRMVYLPMCFLYGRRF 98
Gymnodinium               WCCILGVYEWKGMAPIPPELWLLPS-W-----FPI-HPGRFWCHARMVYLSMCYLYGRRF 63
Alexandrium               WLALLGVYEWEGIAPVPPEMWLLPH-W-----FPV-HPGRFWCHCRMVYLPMCYLYARRF 174
Dinophysis                WLAFLGVYDLRGIAPVPPELWLLPS-W-----FPL-HPGRFWCHCRMVYIPMCYLYGVRF 177
Scrippsiella              WLTFLGVYDYRGLAPVPPELFLLPS-W-----FPV-HPGRFWCHCRMVYLPVCYLYGRRF 175
Aureococcus               ALCLLGAMDWEGHESVPPEMWLLPC-W-----CPF-HPCRMWCHARMVYLPMGYLWGKRW 173
Phaeodactylum             YLCILGCMEWDGHNSVPPEMWLLPN-W-----FPF-HPSRMWCHARMVYLPMGYVYGARL 169
Helicotheca               WLCLLGCMEWDGHNSVPPEMWLLPN-W-----TPF-HPGRLWCHCRMVYLPMGYLYGARF 176
Yeast-erg7                WLSALNLYKWEGVNPAPPETWLLPY-S-----LPM-HPGRWWVHTRGVYIPVSYLSLVKF 250
Candida                   WLSILNLYEWEGVNPAPPELWRLPY-W-----LPI-HPAKWWVHTRAIYLPLGYTSANRV 173
Verrucomicrobia           ILSILNLYDWSGVTPVPPELYLLPK-W-----VPV-QPINISGYVRIVYLPMAYFYGRRW 141
Eudoraea                  ILSFLNLYSYKGVAPVPPELYMLPG-W-----FPF-HPRKISGYVRIIYLPMCYFYRKRL 162
Plesiocystis              WLALLGLYPWDLVQPLLPELWLLPD-D-----APM-HPRRLYCHMRLIYLGLSYLWGARV 215
Enhygromyxa               WLALLGLYPWSELQPLLPELWLLGD-D-----SPM-HPRRLYCHMRLIYLGLSYLYGART 185
Bodo                      WLCVLGLYEYDGINSIPPELSILPD-W-----LPF-SQGMYWCHARIVTTPFSWFYGKRW 177
Trypanosoma               WLCIVGLYSWDGINPIPPELSLLPA-W-----FPL-SQGRLWCHSRVISVPFSYLYGIRW 192
Phytomonas                WLCILGLFSYDGVNPLSPELVLLPR-W-----ISF-SIGRSWCHSRVIAMSFLYFYGLRW 125
Leishmania                WLSILGVYSWDGVNPISPEMLMPD-W-----VPF-SLGKMWCHSRVIAIPFSYFYGLRW 223
Leptomonas                WLSILGVYSWEGVNPIPPEMILLPD-W-----VPF-SLGKAWCHSRVIAIPFSYLYGLRW 224
Labilithrix               TLSLVGLYRWDGLTPLLPELWLLPYAA-----TPF-HPARLWCHCRQVYLPMSWLYGRRA 163
Stigmatella               ILALLGLYEYGGLQPVPPELWLLPE-S-----LPF-HPSRLWCHCRMVYLPMSWLYGRRA 134
Myxococcus                VLALLGLYEYDGLTPTPPELWLLPK-A-----LPF-HPSRMWCHCRMVYLPMGWLYGRKA 161
Corallococcus             LLALLGLYEYEGLAPVPPELWLLPR-G-----LPF-HPSRLWCHCRMVYLPMGWLYGRRA 170
S-cinnamoneus             WLAVLGLVPYREVAPFPPEAMLLPR-W-----VPL-HPERMLGWTRMLYQAMSYLYGARF 186
S-caatingaensis           WLAVLGLIPYEETDPLPPEALLLPA-W-----TPL-HPSRLYGRTRLLHQAMALVHGTRF 161
Sandaracinus              WLAVLNLYSWEGVSAVLPEAWRAPR-W-----LPF-HPSRFYCHTRLIYLGMAVLYGEKW 170
S-alboviridis             WLALLGLYDYRAMHPLLPELFLLPK-W-----LPL-HPDRLYCHTRTISQAMTYLYGTRF 164
G-obscuriglobus           WLTLLGLYGRDGLRPLLPELALLPK-A-----FPV-HPVRFYCHTRYVYLVMSLLQGAHA 178
G-sp.SH-PL17              WLTLLGLYGRDGLRPLVPELALLPR-A-----FPV-HPIRFYCHTRYVYLAMSLLQGSHA 152
G-sp-IIL30                WLTLLGLYGRDGLRPLVPELALLPR-A-----FPV-HPIRFYCHTRYVYLAMSLLQGSHA 171
Methylococcus             WLMLLGLYGREGVNAVPPELFLLPR-W-----LPF-HPSRFYCHTRLIYLGIAYLSGVGF 169
Methylocaldum             WLAMLDLYAYEGLNGIPPELFLLPE-W-----VPI-HPRRYYCHTRQIYLGLAFLYGMRF 171
```

```
Helobdella               K---VDDSEIVRQ-----------------------------LREELYQD----NYDVIV 195
ScLDS                    Y---AQEDEVILE-----------------------------LRKELFLE----EFDEID 195
AjLDS-b                  Y---AQEDELIMD-----------------------------LRKELFME----DFDQID 272
AjLDS-a                  Y---AQEDELIMD-----------------------------LRKELFIE----DFDQID 272
PpLDS                    Y---AQEDELIME-----------------------------LRKELFIE----DFDQID 272
ScPS                     Y---APEDDLIIS-----------------------------LRKELFVE----DFDHIN 195
PpPS                     Y---APEDSLILS-----------------------------LRKGLFVE----DFETID 264
AjPS-b                   Y---APEDSLILS-----------------------------LRKELFVE----DFETID 264
AjPS-a                   Y---APEDSLILS-----------------------------LRKELFVE----DFETID 264
Capsaspora               R---APVTPIIAE-----------------------------LREELYTQ----AYDTIN 196
Saccoglossus-LSS         T---AQEDDLIRS-----------------------------LRKSCCLF--------GD 260
EpLSS                    Q---AQEDGLIRE-----------------------------LRNEIFKE----DFATIN 270
SpLSS                    Q---AEENQLTRE-----------------------------LRKEIFKA----DYSSIH 270
SgLSS                    Q---AEENDLIRE-----------------------------LRKEIFKE----DFSSIH 270
LspLSS                   Q---AEPDDLIMD-----------------------------LRMELYVE----DYYTID 270
MgLSS                    Q---AEPDDLIMD-----------------------------LRMELYVE----DYYTIN 48
AaLSS                    ------------------------------------------------------------ 0
AfLSS                    Q---AEPDDLIMD-----------------------------LRMELYVE----DYYTIN 270
ArLSS                    Q---AEPDDLIMD-----------------------------LRMELYVE----DYYTIN 270
HspLSS                   Q---AEEDDLIRE-----------------------------LRKELYVE----DYSTIN 270
EsLSS                    Q---AEEDDLIRE-----------------------------LRKELYVE----DYSTID 270
ApLSS                    Q---AEEDDLIRE-----------------------------LRKELYIQ----DYNTID 270
PpLSS                    Q---AEEDELIRE-----------------------------LRKELYTQ----DYHTID 270
PmLSS                    Q---AEEDDLIRE-----------------------------LRKELYTQ----DYHTID 270
Amphimedon               K---AKPTKIILE-----------------------------LRQEIYVQ----SYESID 193
Aplysia                  S---APEDDLIRS-----------------------------LRKELFVE----DFDKIN 196
Capitella                T---AKETDLIRE-----------------------------LREEIYVE----SYDSIE 196
Branchiostoma-LSS        T---AEVDSLVRE-----------------------------LRTELYLE----DYERIN 241
Lingula                  S---ADEDDLIRE-----------------------------LRKELYTE----SYESIQ 196
Callorhinchus            T---AQEDTLIHS-----------------------------LRQELYVQ----DYCSID 195
HumanLSS                 S---AAEDPLVQS-----------------------------LRQELYVE----DFASID 272
Pteropus                 R---AEEDPLVQS-----------------------------LRQELYVE----DYASID 196
DreLSS                   S---ADEDPLVLS-----------------------------LRQELYVQ----DYSTID 273
Haplochromis             A---ADEDPLVLS-----------------------------LRQELYVQ----NYAFIN 196
Xenopus                  S---AHEDDLIRS-----------------------------LRQELYLE----DYSSIN 196
Charadrius               S---AEEDELVRS-----------------------------LRQELYVE----DYASID 192
Anolis                   T---AEEDELILS-----------------------------LRQELYVQ----DYSTID 196
Alligator                T---AEEDELVQS-----------------------------LRQELYIQ----DYSSID 196
Pneumocystis             T---APLDPLIES-----------------------------LREELYTQ----PYSSIN 197
Arthrobotrys_            S---RPLNDLTRA-----------------------------LRAELFIQ----PYSSIN 197
Aspergillus              T---HPLDPLTKQ-----------------------------LRQELYTQ----PYDSIN 197
Spizellomyces            V---CRETNLIRE-----------------------------LREELYTQ----PYESIK 193
Trichosporon             V---APQTELVRE-----------------------------LRKEIYVQ----PYESID 180
Puccinia                 Q---AQSDPTLAS-----------------------------LRNELYTQ----PYESID 201
Mixia                    K---APLDALTKA-----------------------------IRQEIYAE----PYSMID 200
Microbotryum             Q---ADLDPLILS-----------------------------LRQEIYVE----PYESIK 200
Dacryopinax              Q---APEDPLILQ-----------------------------LREEIYTT----DYDHID 197
Fistulina                K---MPENDLIMS-----------------------------LRKELYPQ----DYYSIH 197
Wallemia                 I---GRSTELTEQ-----------------------------LKQELYVD----PYASID 197
Ceraceosorus             K---CPVDPLIES-----------------------------LRQELYTE----PYESIH 196
Ustilago                 K---AEMDPLIAS-----------------------------LREELYVQ----PYESID 197
Malassezia_              T---APVTPLIES-----------------------------LRAELYPT----AYKDID 197
Fluviicola               K---AELTPLVQE-----------------------------LRKELYTA----DYDSIN 195
Methylobacter            K---APENELILS-----------------------------LREELYNQ----DFAAID 195
Methylobactes            K---APENELILS-----------------------------LREELYNQ----DFAAID 195
Methylosarcina           K---APENELILS-----------------------------LREEIYNE----DFAAID 195
Aphanomyces              Q---AKETPLIQA-----------------------------IRDEIYTT----PYQSVS 194
Cystobacter              R---IPDSPLLQQ-----------------------------LRRELYPT----PYEQID 193
Galdieria                T---AKETSLIRE-----------------------------LKEELYLD----NFDQID 194
Selaginella              T---GKITPKVEA-----------------------------LRKELFNG----PFEDVD 196
Panax                    V---GPINSTVLS-----------------------------LRRELYTH----PYHQIN 206
Physcomitrella           T---GKITELVKA-----------------------------IREEIFVQ----KYTEVN 198
Cucumis                  V---GPITPIVLS-----------------------------LRKELYTI----PYHEID 199
Adiantum                 T---GKITETVLA-----------------------------LRKELFKV----PYEDID 199
Abies                    V---GPLTGIVMS-----------------------------LREELYTV----PYEKID 200
Malus                    V---GPITPTIRS-----------------------------LRKELYTA----PYHEVD 197
Amborella                V---GPITTTVLS-----------------------------LRKELYTI----PYNKVD 199
Nicotiana                V---GPITPTVSS-----------------------------VRNELFTV----PYHEIN 198
AtCAS1                   V---GPITSTVLS-----------------------------LRKELFTV----PYHEVN 297
Glycine                  V---GPISPTVLS-----------------------------LRKELYTV----PYHDID 198
Coffea                   V---GPITPTVLS-----------------------------LRKEIYAV----PYHEID 198
Populus                  V---GPITPIVLS-----------------------------LRKELFTV----PYHEID 198
Vitis                    V---GPLTPTVLS-----------------------------LRKELYTV----PYHEID 198
Micromonas               S---GASSFLTEE-----------------------------LKSELFVG----NYKTIN 197
Volvox                   T---CKETALTAAISQPLPPRSHLRNSPPGSHHIRTWPTHMPYRQELYPM----PYSKID 229
Chlorella                T---CRATPLTEA-----------------------------LRQELYPL----PYAKID 202
Synchroma                S-ARAEDDELLSA-----------------------------LRRELYLQ----PYETID 198
Chattonella              TYPKAEQDPLITE-----------------------------LRHEIYCQ----EYAAIK 197
Ochromonas               T-PDVANDPTLTS-----------------------------LRTELYLQ----KYDTIK 198
Chromulina               T-PDVDNDELLQS-----------------------------LRKEIYLE----DYDTID 201
Chondrus                 V---GEVTELVRE-----------------------------LREELYEE----PYESIE 194
Acanthamoeba             T---APESPLVLS-----------------------------LRKELYPN---DDYAKIN 197
Dictyostelium            T---GPLTDLVKD-----------------------------LRREIYCQ----EYEKIN 191
Polysphondylium          T---AAETPLIRE-----------------------------LRKELYVT----PYSEIN 194
Acytostelium             T---AAETPLIRE-----------------------------LRQELYVT----DYSTIN 191
SHC-Candidatus           NRRA---PNG--F-----------------------------NLDELLL----PGVKL-- 135
```

```
SHC-Chlorogloeopsis      VYKV---EPA--I-----------------------------TLNELYA----EGAEQIK 188
SHC-Streptomyces         VRPA---PF---------------------------------ALDELHT----DPRRP-- 178
SHC-Hyphomicrobium       RARN---PKG--I-----------------------------GIAELFA----TPPEQVR 184
SHC-Leptospirillum       LVRL---SPE--R-----------------------------GISELFD----PARPDGE 153
Naegleria                VYANAETDPIISQ-----------------------------LRNELYDQVSKTSYQNQP 205
Symbiodinium             TYKAA-DDPVTSA-----------------------------LRCELFP---DQTYAQID 134
Prorocentrum             VYPAASEDPVTLS-----------------------------LREELYAV--DERYSDIR 127
Gymnodinium              AYDAE-SDPVTAA-----------------------------LRSELYP---GREYAKID 90
Alexandrium              SYDAA-ADPLTAS-----------------------------LRRELYPT--KCAYSEIR 202
Dinophysis               AYDAA-ADPVDRR-----------------------------PAGGAVPA--GRGVRQDR 205
Scrippsiella             AYDAA-ADPLTAA-----------------------------LRRELYLP--GTEYSKID 203
Aureococcus              TYENADSDPVVLA-----------------------------LRDELYPA--SPAYGAIP 202
Phaeodactylum            KYDKAEEDPLVQA-----------------------------LRRELYCE----PYNSIE 196
Helicotheca              VYPDAEKDPLISS-----------------------------LREELYIE----PYESID 203
Yeast-erg7               SCPM---TPLLEE-----------------------------LRNEIYT----KPFDKIN 274
Candida                  QCEL---DPLLKE-----------------------------IRNEIYVP-SQLPYESIK 200
Verrucomicrobia          QAPL---DPLLRE-----------------------------IRRELFP----QGFDQID 165
Eudoraea                 SIET---DELILE-----------------------------LRKELYR----QPYDQIN 186
Plesiocystis             QAPS---GPVLDA-----------------------------LAAELYP----GGPSRAR 239
Enhygromyxa              QADA---SPLVEA-----------------------------IRGELYP----EGWDVSR 209
Bodo                     KADS---HPLLEA-----------------------------IKTEIYV----QPYAKIS 201
Trypanosoma              APEP---HPLLEA-----------------------------LRQELYT----EPYDQIQ 216
Phytomonas               SASR---HPLLEA-----------------------------LKEELFV----QPFHTIC 149
Leishmania               SAPA---FPTTLA-----------------------------LRKELYT----EPYATIP 247
Leptomonas               SAPA---FPTTLA-----------------------------LREELYT----QQYSAIP 248
Labilithrix              RIDD---DELIRA-----------------------------LRDELYD----GAWSSID 187
Stigmatella              RAPE---TPLLAA-----------------------------IRQEIFD----GGYGQVD 158
Myxococcus               RVRQ---TPLLAE-----------------------------LRRELYP----EPYESVD 185
Corallococcus            RAPE---TPLLAE-----------------------------LRRELYP----QPYADVD 194
S-cinnamoneus            RADL---GSLADE-----------------------------LGRELFP----ATPRPAG 210
S-caatingaensis          RTDL---GPLTAE-----------------------------LRRELLP----HGGRTGA 185
Sandaracinus             SAPV---TPRILA-----------------------------IRDEIFP----GGWESVD 194
S-alboviridis            QGPQ---GGVVED-----------------------------LRRELFD----RPLRRSD 188
G-obscuriglobus          TFDL---GPLRAE-----------------------------LERELYG----PLAVPES 202
G-sp.SH-PL17             RFAL---GSLGDE-----------------------------LRHELYG----SAGPPAS 176
G-sp-IIL30               RFAL---GSLGDE-----------------------------LRHELYG----SAGPPAS 195
Methylococcus            SASL--SDPLRDA-----------------------------LRSELYA----EPYESVD 194
Methylocaldum            RASL--PDKLRDQ-----------------------------LRQELYR----EPYETID 196


Helobdella               WRHHRSNISLADLHTP--------------------HTYLLKSAY--------------- 220
ScLDS                    WPSQRNNIAEVDLYTP--------------------HSWLFKIAF--------------- 220
AjLDS-b                  WPSQRDNIAKIDLYTP--------------------HSWLFNIAF--------------- 297
AjLDS-a                  WPSQRDNIAKIDLYTP--------------------HSWLFNIAF--------------- 297
PpLDS                    WSSQRDNIAEIDLYTP--------------------HSWLFNITF--------------- 297
ScPS                     WPSQRDNIAEIDLYTS--------------------HSWLHTLAF--------------- 220
PpPS                     WPAQRENIAKIDIYTP--------------------HSWLYSMVF--------------- 289
AjPS-b                   WPAQRENIAKIDIYTP--------------------HSWLYSMVF--------------- 289
AjPS-a                   WPAQRENIAKIDIYTP--------------------HSWLYSMVF--------------- 289
Capsaspora               WPAQRFRCAKIDLYNP--------------------HSWIMDWSF--------------- 221
Saccoglossus-LSS         WISQRAVDKSCCLFGDRISQRVVGKSCCLFGDRISQRVVGKSCCL--------------- 305
EpLSS                    WPAQRDNISKYDLYTP--------------------HSWLYTIAM--------------- 295
SpLSS                    WPSQRDNISKFDLYTP--------------------HSWLYNIAM--------------- 295
SgLSS                    WPSQRNNISKFDLYTP--------------------HSWMYNIAM--------------- 295
LspLSS                   WPAQRSNIAKVDLYTP--------------------HSWLYSLTF--------------- 295
MgLSS                    WPAQRSNIAKVDLYTP--------------------HSWLYNLAF--------------- 73
AaLSS                    ------------------------------------------------------------ 0
AfLSS                    WPAQRSNIAQVDLYTP--------------------HSWLYNLAF--------------- 295
ArLSS                    WPAQRSNIAQVDLYTP--------------------HSWLYNLAF--------------- 295
HspLSS                   WSKQRNNIAKVDLYTP--------------------HSWLYNVVF--------------- 295
EsLSS                    WPKQRNNIAKVDLYTP--------------------HSWLYNVLF--------------- 295
ApLSS                    WPAQRDNIAKIDLYTP--------------------HSWLYNLAF--------------- 295
PpLSS                    WPAQRDNIAKVDLYTP--------------------HSWLYNLAF--------------- 295
PmLSS                    WPAQRDNIAKVDLYTP--------------------HSWLYNLAF--------------- 295
Amphimedon               WPSQRNNVSSTDLYTP--------------------HSLALDWSY--------------- 218
Aplysia                  WPAQRNNVSSADLYSP--------------------HAIILDIAY--------------- 221
Capitella                WSKQRSNVASADLYSP--------------------HSWILDTAY--------------- 221
Branchiostoma-LSS        WPAQRNNVSACDVYTP--------------------HSWLLNLAY--------------- 266
Lingula                  WSSQRDNVSSADLYTP--------------------HSWFLDLAY--------------- 221
Callorhinchus            WPAQRNNVAPCDLYTP--------------------HSWLLNTAY--------------- 220
HumanLSS                 WLAQRNNVAPDELYTP--------------------HSWLLRVVY--------------- 297
Pteropus                 WPAQRDNVAPGDLYTP--------------------HSWLLRAAY--------------- 221
DreLSS                   WPAQRNNVATCDLYTP--------------------HSNLLTFAY--------------- 298
Haplochromis             WPAQRNNVAACDMYTP--------------------HSTLLTVAY--------------- 221
Xenopus                  WPAQRNNVASCDIYTP--------------------HSTLLHIAY--------------- 221
Charadrius               WPAQRNNVAACDVYTP--------------------HSWLLGVAY--------------- 217
Anolis                   WPAQRNNVASGDIYTP--------------------HSWLLTVAY--------------- 221
Alligator                WPAQRNNVAACDLYTP--------------------HSWLLTVAY--------------- 221
Pneumocystis             FSKHRNTVSSVDVYVP--------------------HTTFLRVIN--------------- 222
Arthrobotrys_            FAAHRNTIRAEDIYHP--------------------HTLVLDLFN--------------- 222
Aspergillus              FANHRNSIHAADNYYP--------------------KTWLLNLIN--------------- 222
Spizellomyces            WSKMCNNVAESDIYYP--------------------HTRLLDGLN--------------- 218
Trichosporon             WPRQRNNTHKVDLYSP--------------------HHPVYDALN--------------- 205
Puccinia                 WPRCRNSIAKEDLYSP--------------------RHPIANGLF--------------- 226
Mixia                    WNKARNQVAQVDMYCP--------------------HSTWLDYGF--------------- 225
Microbotryum             WSSCRNNVCPIDLYAP--------------------HSAVANGLF--------------- 225
Dacryopinax              WPAQQFNVAPVDLYFP--------------------HTTLLHALG--------------- 222
Fistulina                WPSQRNNVAAIDLYAP--------------------HSALFDTIN--------------- 222
```

```
Wallemia                 WSAQRNNVNKIDLFQP--------------------HSAVMDALN--------------- 222
Ceraceosorus             WPSQRNHVAEVDIWAP--------------------HTKTMEGLM--------------- 221
Ustilago                 WPSQRGNIAQVDTFYP--------------------HTKTLKTLM--------------- 222
Malassezia_              WPAQRNHVASVDLYAP--------------------HTRVVNALF--------------- 222
Fluviicola               WKKARREACEIDIYHP--------------------VNKWYYRLS--------------- 220
Methylobacter            WPKQRDAVCEKDRYTT--------------------LSPILKWMN--------------- 220
Methylobactes            WPKQRDAVCEKDRYTT--------------------LSPILKWMN--------------- 220
Methylosarcina           WPKQRNAVCEKDCYTT--------------------PSPVLKWMN--------------- 220
Aphanomyces              WRNARGAYSKMDEYHT--------------------PSPIIRTLN--------------- 219
Cystobacter              WPAHRTRVSPTDSQVP--------------------RSALVLAAN--------------- 218
Galdieria                WNAQRENCCKEDIYTH--------------------RPKVQSWLW--------------- 219
Selaginella              WNRARNSCAKEDLYYP--------------------HPLIQDVLW--------------- 221
Panax                    WDLARNQCAQEDLYYP--------------------HPLIQDMLW--------------- 231
Physcomitrella           WNEARNLCAKEDLYYP--------------------HPWIQDVLW--------------- 223
Cucumis                  WNRSRNTCAKEDLYYP--------------------HPKMQDILW--------------- 224
Adiantum                 WNKARNECAKEDLYYP--------------------HPMIQDVLW--------------- 224
Abies                    WNQARSMCAKEDLYYP--------------------HPFLQDILW--------------- 225
Malus                    WNKARNLCAKEDLYYP--------------------HPMVQDVLW--------------- 222
Amborella                WNDARNLCAKEDLYYP--------------------HPLVQDILW--------------- 224
Nicotiana                WNKARNECAKEDLYYP--------------------HPLVQDILW--------------- 223
AtCAS1                   WNEARNLCAKEDLYYP--------------------HPLVQDILW--------------- 322
Glycine                  WDQARNLCAKEDLYYP--------------------HPLVQDILW--------------- 223
Coffea                   WNLARNQCAKEDLYYP--------------------HPLVQDILW--------------- 223
Populus                  WNQARNLCAKEDLYYP--------------------HPLVQDALW--------------- 223
Vitis                    WNQARNLCAKEDLYYP--------------------HPLVQDILW--------------- 223
Micromonas               WDKTRNTCASEDLYYS--------------------HPRIQDALW--------------- 222
Volvox                   WNAARNQCAKEDLYYP--------------------HPLVQDVLW--------------- 254
Chlorella                WNQARNLCAKEDLYYP--------------------HPLIQDVLW--------------- 227
Synchroma                WDAHRFSICPFDNYSP--------------------VEPVMRLAC--------------- 223
Chattonella              WGRHRQTVSKLDEYDP--------------------VGLLMRTLQ--------------- 222
Ochromonas               WDDFRQTCADIDEYSP--------------------LNPIMKVAQ--------------- 223
Chromulina               WDGSRQLCADIDEYSP--------------------LNPVMKLAQ--------------- 226
Chondrus                 WPAWRGRCCEEDVYVR--------------------RPKLQRVLW--------------- 219
Acanthamoeba             WYSIRSYVSPLDLYYP--------------------HSTLLECLY--------------- 222
Dictyostelium            WSEQRNNISKLDMYYE--------------------HTSLLNVIN--------------- 216
Polysphondylium          WPAQRDHINKLDMYAP--------------------HSYLLKSVN--------------- 219
Acytostelium             WPAQKNSINKLDMYAP--------------------HSTLLKGIN--------------- 216
SHC-Candidatus           ---------ALPKRKG--------------------LAVLFHHLD--------------- 151
SHC-Chlorogloeopsis      F--------TLPSKGD--------------------WTDLFISLD--------------- 205
SHC-Streptomyces         N-----PPRPLASPAT--------------------WDGFFQRLD--------------- 198
SHC-Hyphomicrobium       N-----WPKGPHQKFP--------------------WSQVFGGID--------------- 204
SHC-Leptospirillum       S-----FAPSP-DFFS--------------------LRNLFLLLD--------------- 172
Naegleria                WSKYRSYVNPRDNYHP--------------------FTQLYSKFA--------------- 230
Symbiodinium             WRRHVHSVAAIDNYSP--------------------IHPFMRWLQEALLXXXXXXXXIHP 174
Prorocentrum             WGNFLHTIADVDNYSP--------------------IHWAMRLLQ--------------- 152
Gymnodinium              WDNHMHSIADVDNYSP--------------------IHWAMRALQ--------------- 115
Alexandrium              WGDYMHAVADIDNYSP--------------------IHWAMRLIQ--------------- 227
Dinophysis               VAQAMFSIAGMDNYSP--------------------VHWVMRALE--------------- 230
Scrippsiella             WHNHMFSIADIDNYSP--------------------VHWFMKVAE--------------- 228
Aureococcus              WRATRSWVAPMDDYSP--------------------VHPLMVAAQ--------------- 227
Phaeodactylum            WMQTRHMVAPMDNYSP--------------------VAWMMKTVQ--------------- 221
Helicotheca              WIRTRHFVAPMDNYSP--------------------IPWTMEFLQ--------------- 228
Yeast-erg7               FSKNRNTVCGVDLYYP--------------------HSTTLNIAN--------------- 299
Candida                  FGNQRNNVCGVDLYYP--------------------HTKILDFAN--------------- 225
Verrucomicrobia          WPKHRADLAPTDHIVP--------------------ETLLVRIAM--------------- 190
Eudoraea                 FAKHRGTFADTDNIFP--------------------ETTIFKISM--------------- 211
Plesiocystis             FEAARDDIAPTDLFEP--------------------VGAVLQWAF--------------- 264
Enhygromyxa              FRATRDQIASTDLYEP--------------------IGQSLEWLF--------------- 234
Bodo                     WAKHRSDVFAPDIYTP--------------------HSWVYTVAN--------------- 226
Trypanosoma              WDQHQSNICNLDCYTP--------------------ISSTYKLVA--------------- 241
Phytomonas               WRRYRYEVCPKDLYTP--------------------VSRTYKVFC--------------- 174
Leishmania               WRSFRGVVCELDVYSP--------------------TSPLFRVAM--------------- 272
Leptomonas               WRSFRGAVCASDLYTP--------------------TSSLFRMAM--------------- 273
Labilithrix              WSAHRDTVAQVDAYRP--------------------PSRLLALAN--------------- 212
Stigmatella              WVAARERVSPTDVFTP--------------------RTFWLKAAN--------------- 183
Myxococcus               WKAARQRVADTDAYTP--------------------RSVWLRAAS--------------- 210
Corallococcus            WKAARGRVARTDAYSP--------------------HGLGLRAVH--------------- 219
S-cinnamoneus            -------P-GSDVVLP--------------------RSRLLRALQ--------------- 227
S-caatingaensis          -------PVGPDAPLP--------------------PGRPPRLLT--------------- 203
Sandaracinus             WAKARETLRTAEIHTP--------------------WTPALHVGY--------------- 219
S-alboviridis            -----RHRTGIDAAVS--------------------PTVPLRLLQ--------------- 208
G-obscuriglobus          FRQYRYRLAETDAFEP--------------------PNLFIRVAE--------------- 227
G-sp.SH-PL17             FREHRYHLAPSDAFEP--------------------PNLLIRAAE--------------- 201
G-sp-IIL30               FREHRYHLAPSDAFEP--------------------PNLLIRAAE--------------- 220
Methylococcus            FGAFRHTVARTDLYVP--------------------ISRVLRLVY--------------- 219
Methylocaldum            FPALRNTLAATDVYVP--------------------ISPVLRKIY--------------- 221


Helobdella               ------YLI-DVYEKF--------HMTYLREKSLNLIYEHVVFDDDVTNFISIGPISKVI 265
ScLDS                    ------GIL-DQYESF--------RLNRYRNNALDACLDHIKQDDLMTSFISIGPISKMI 265
AjLDS-b                  ------GIL-DKYEPF--------RLTRFRKQALDVCLDHIKQDDLMTSFISIGPISKMI 342
AjLDS-a                  ------GIL-DKYEPF--------RLTRFRKQALDVCLDHIKQDDLMTSFISIGPISKMI 342
PpLDS                    ------GIL-DKYEPF--------RLTRFREQALDVCLDHIKQDDLMASFISIGPISKMI 342
ScPS                     ------GLL-DIYESI--------HHKWYREKALEICLDHIKQDDIMTNFISIGPVSKMM 265
PpPS                     ------GML-DTYEPF--------HSSWYRESALKVCLDHIKQDDIMTNFISIGPISKMI 334
AjPS-b                   ------GML-DTYEPF--------HSSWYRESALKVCLDHIKQDDIMTNFISIGPISKMI 334
AjPS-a                   ------GML-DTYEPF--------HSSWYRESALKVCLDHIKQDDIMTNFISIGPISKMI 334
Capsaspora               ------SAL-NVYEKF--------HSTSIRKRALDMALDHIRAEDENTQCVGIGPISKNI 266
```

```
Saccoglossus-LSS         ------FVL-NVYESW--------HSKRLRQKAIQECYDHIQADDRFTKCISIGPVSKVI 350
EpLSS                    ------AIL-DFYEKF--------HSTWLRQKALDFCYDHIKADDTFTKGISIGPISKVI 340
SpLSS                    ------AIL-DLYEKF--------HSTWLKKKALDHCYEHIKADDEFTNGISVGPISKVI 340
SgLSS                    ------AIL-DFYETF--------HSTWLRKKALDHCYDHIKADDEFTKGISIGPISKVI 340
LspLSS                   ------GCL-DKYEKY--------HIAKGRQRALEECLDHIKADDEFTQCISIGPISKVI 340
MgLSS                    ------GCL-DIYEKY--------HFADWRQRALDECLDHIKADDKFTKCISIGPISKVI 118
AaLSS                    ------------------------------------------------------------ 0
AfLSS                    ------GCL-DIYETY--------HSTKWRQSALDECLDHIKADDEFTQCISIGPISKVI 340
ArLSS                    ------GCL-DIYEKY--------HSTKWRQSALDECLDHIKADDEFTQCISIGPISKVI 340
HspLSS                   ------GTL-DVYESF--------HFSAFRQQALDECLDHIRADDQFTKCISIGPISKVI 340
EsLSS                    ------GIL-DVYESF--------HFPAFRQQALDECLDHIRADDQFTNCISIGPISKVI 340
ApLSS                    ------GFL-DVYEPY--------HSTYFRQMALDECLDHIRADDQFTKCISIGPISKVI 340
PpLSS                    ------GFL-DVYEPY--------HSTAFRQRALDECLDHIRADDRFTKCISIGPISKVI 340
PmLSS                    ------GFL-DVYEPY--------HSTAFRQRALDECLDHIRADDRFTKCISIGPISKVI 340
Amphimedon               ------LLL-DAYESH--------HISRLRAWADEEILRQIKADDSFTNCISIGPISKVI 263
Aplysia                  ------TIL-NIYEKV--------HLGFLRKWALADMYDHISADDRFTKCISIGPISKVI 266
Capitella                ------YFL-DWYEMY--------HSKSWRQKSLDKIYEHICADDEFTKCISIGPISKTI 266
Branchiostoma-LSS        ------VGI-NLYESW--------HSSSLRQWAIDELYDHIKADDSFTKCISIGPISKVI 311
Lingula                  ------YFL-DFYESH--------HNSSWRQASLHECYTHICADDEFTKGISIGPISKVI 266
Callorhinchus            ------AVL-NVYEDH--------HSERLRCWAITEVYDHITADDRFTKCISIGPISKTI 265
HumanLSS                 ------ALL-NLYEHH--------HSAHLRQRAVQKLYEHIVADDRFTKSISIGPISKTI 342
Pteropus                 ------AIL-DLYERH--------HSTSLRQRAVQRLYEHIAADDRFSKCLSIGPISKTI 266
DreLSS                   ------FFL-NVYEAH--------HSTILREKAVKELYDHIKADDRFTKCISIGPISKTI 343
Haplochromis             ------MVL-NVYEAH--------HSTTLRGKAVKELYDHIEADDRFTKCISIGPISKTI 266
Xenopus                  ------AFL-NVYESY--------HIPALRRRAVHELYDHIAADDRFTKCISIGPISKVI 266
Charadrius               ------TIM-NVYEAH--------HSTHLRQRAITELYDHIKADDRFTKCISIGPISKTI 262
Anolis                   ------AIL-NTYERF--------HSTSLRQHALEVLYDHIRADDRFTKAISIGPISKTI 266
Alligator                ------AIL-NLYEAY--------HSYHLRQRAIEELYDHIKADDRFTKAISIGPISKTI 266
Pneumocystis             ------SI-LTFYHTT-------FRLSWIKNIASKYTYELIEYENKNTDFLCIGPVNFCI 268
Arthrobotrys_            ------TA-CTFWSKY-------LLPNWLKKWAEDYVYSLVVREDENTLYVNLGPVNNPL 268
Aspergillus              ------QLLVSVWNPY-------FRIPALVKRAEEWTWELIRMEDENTDYAGLGPVSNPM 269
Spizellomyces            ------GIC-WLYEKL--------GPSRLRKWAIEECWLQLQMEDENTKFGNLASVNHPL 263
Trichosporon             ------AIL-GVYEKM----PYLPCVPGLRKAGMRAAYRQIVYEDENTGYQTVGPVSKAF 254
Puccinia                 ------WVL-GYWERV--------CPSWIRNIGLDRAHELCKMEDENTDFNDLAPVNKVL 271
Mixia                    ------DAL-VQYEKH--------RPDWLRQRGIDYVYKLIVMEDENTGYQTIGPVSKAM 270
Microbotryum             ------AIL-NVYDRF--------APAFIRNRGLARAYELIKMEDDNTSYQSVGPVSKAM 270
Dacryopinax              ------AVT-HAWEHC--------FIPFIRRRAMEYTYQLVVQEDENTQCQDLGPVNKML 267
Fistulina                ------VAL-SAYEGC--------AFPPLRRRGIERAYELVVYEDENTSYQTLAPVNKMF 267
Wallemia                 ------AALYYGYEPC--------SIPPLRQAALRKAYTHIVHEDENTCYQGLAPINKAF 268
Ceraceosorus             ------AVL-GVYESC-------GSIPPLRRAGVKRAYQLLAMEDENTSYQTVGPVSKML 267
Ustilago                 ------ALV-GAYDHC--------HIPALRKAGIQRAYDLLVLEDENTSYQCLGPVNKML 267
Malassezia_              ------YVL-GKYEHI--------HIPSLREAGMKRAYELIVKEDSNTSFQCLGPVNKML 267
Fluviicola               ------NII-NGYERI--------HSNRFRKKALKYVEDYIDFEDTYTRYINIGPVNQAI 265
Methylobacter            ------FFT-NSYEQF--------KCAWLRKKSTDYILKYLNAEDEQTNYINIGPVNQAI 265
Methylobactes            ------FFT-NSYEQF--------KCAWLRKKSTDYILKYLNAEDEQTNYINIGPVNQAI 265
Methylosarcina           ------FFT-NNYEKF--------KCSWLRKKSIDYILKYLHAEDQQTHYINIGPVNQAI 265
Aphanomyces              ------YLL-SWYELLP-------TVTSLRKHGLEYTLAFVRADDEESNYCNIGPVNKMI 265
Cystobacter              ------HLM-GLYESQ--------ASTRLRERALSFVLDHIRQEDENTRYICIGPVNKLL 263
Galdieria                ------TIL-SWYEKFP---IP--GKSYLRNLALEETLLQVKKEDEYTDFICIGPVNKVL 267
Selaginella              ------GVL-HKGVEPL---LMRWPGSLLRKKALDTVLEHIHYEDVNTRYICIGPVNKVI 271
Panax                    ------SCL-HKGVERL---IMQWPLSKIRQRALTTAMQHIHYEDENTSYICLGPVNKVL 281
Physcomitrella           ------GTL-DKVVEPI---LTRWPGSLLRKKALARTMEHIHYEDENTRYLCIGPVNKVM 273
Cucumis                  ------GSI-YHVYEPL---FSGWPGKRLREKAMKIAMEHIHYEDENSRYICLGPVNKVL 274
Adiantum                 ------ATL-HKLVEPA---LMNWPCSSLRKKALDTVIKHVHYEDENTRYICIGPVNKVL 274
Abies                    ------GTL-HKVVEPA---LMHWPGSMLRERALQSVMKHIHYEDENTRYICIGPVNKVL 275
Malus                    ------GSL-HYVYEPV---FTRWPAKELRDSALKTVMQHIHYEDENTRYICIGPVNKVL 272
Amborella                ------GFL-HKVGEPI---FMHWPGSKLREKALHTVMQHIHYEDENTRYICIGPVNKVL 274
Nicotiana                ------ASL-DKLVEPI---FMHWPGKKL-EKALRTVMDHIHYEDENTRYICIGPVNKVL 272
AtCAS1                   ------ASL-HKIVEPV---LMRWPGANLREKAIRTAIEHIHYEDENTRYICIGPVNKVL 372
Glycine                  ------ASL-HKFLEPI---LMHWPGKRLREKAIISALEHIHYEDENTRYICIGPVNKVL 273
Coffea                   ------ASL-HKVLEPI---LMHWPGKKLREKAVSVAMEHVHYEDENTRYLCIGPVNKVL 273
Populus                  ------ALL-DKAAEPV---LMHWPGKKLREQALHTAMEHMHYEDENTRYICIGPVNKVL 273
Vitis                    ------TSL-DKVVEPI---LMHWPGKKLREKALRTVLEHVHYEDENTRYICIGPVNKVL 273
Micromonas               ------WGL-TKVEPFF---LRWWPGRWIRANALKLTMNHIHYEDENTRYINIGPVNKVM 272
Volvox                   ------WAL-YRAENVL---Q----GSFLRRMALKECMKHIHYEDENTRYIDIGPVNKVV 300
Chlorella                ------WAL-YKAEPLL---L----GSRLRGAALAECMKHIHYEDENTRYVDIGPVNKVI 273
Synchroma                ------NAL-ALYERYL---VGLAPFRWLRSAGLRFVMDYIHAEDAQTNYVCIGPVNKAL 273
Chattonella              ------SIL-AVYEKYI---PF----KLGRESALKFAFDYIEAEDRQTNYVDIGPVNKSL 268
Ochromonas               ------DFL-AIYEKVL---PHIPFLKNLRQKGLEFAIDYIHAEDLQTNFIDIGPVNKAL 273
Chromulina               ------DFL-SIYERVL---PHIPFLKQLRQRGSDFAIDYINAEDIQTNYIDIGPVNKSL 276
Chondrus                 ------GAL-ALWESIW-----FPGKQWLRERALKETLMQITAEDENTNYICIGPVNKVI 267
Acanthamoeba             ------VIL-DNYEKV--------HSSWLREKSVLLTAEHVAAEDKFTDWVCIGPVNKTI 267
Dictyostelium            ------GSL-NAYEKV--------HSKWLRDKAIDYTFDHIRYEDEQTKYIDIGPVNKTV 261
Polysphondylium          ------GAL-NLYERM--------HSKWLRDKAIDFTFDHIRFEDEQTKYIDIGPVNKTL 264
Acytostelium             ------AAL-GVYEGV--------HSKWLRDKAIDFTFDHIRYEDEQTKYIDIGPVNKTL 261
SHC-Candidatus           ------RMF-KVWE--------KRGSERIRGAAIREAERWLIARTHYTEGLGAI-YPAMM 195
SHC-Chlorogloeopsis      ------SAF-KFAE--------KLNLVPFREEGINAAERWILERQEATGDWGGI-IPAML 249
SHC-Streptomyces         ------KVL-RAYR--------KVSIGPLRRAAMNAASRWIIERQENDGAWGGI-QPPAV 242
SHC-Hyphomicrobium       ------RVL-RLVE--------PAFPKSLRKKSIDKAVAFVTERLNGEDGLGAI-FPAMV 248
SHC-Leptospirillum       ------KVL-QVWN--------RHPPGFLRKKALSFAMEWMVPRLKGEGGLGAI-YPAMA 216
Naegleria                ------SVL-TVYEKYI---N----FSFLRNASLKKCIEHIKYEDSVTNHICIGPVNKVL 276
Symbiodinium             FMRWLQEAL-LVYE------RFGP-WKWLRKISSDFALEYINSEDLETNFLTIGPVSKAL 226
Prorocentrum             ------NAL-AAYE------AIGA-WAWPRRASLRFAEEYIASEDLETNYLTM------- 191
Gymnodinium              ------RAL-TVYE------RLGA-VRCVRDRALRFVEEYMISEDIETNYLDIGPVSKAM 161
Alexandrium              ------NLL-CVYE------RWGP-WRLIRDRSSRFAEEYVHSEDVETNYLTIGPVSKAL 273
Dinophysis               ------TVF-AFYEW-----TTGP-IGFLRRWSLRFVEEYMHAEDVETNYLTIGPVSKAF 277
Scrippsiella             ------RIF-AMYEW-----SIGP-WKFLRDPALRFAEEYMHAEDMETNYLTIGPVSKAF 275
Aureococcus              ------RFL-RVYED-----LGGPLRRYARRKGLAFSADYCRAEDLQTNYVCIGPVNKVY 275
```

Phaeodactylum	------NGL-ARYETWP---MLQPFKNDVRKLGLAFCVDYMAAEDLQTNFIDIGPVNKVL	271
Helicotheca	------NCL-ARYETWF---IFQPFKNWVRKKALKFSVEYMDAEDLSTNYIDIGPVNKAL	278
Yeast-erg7	------SLV-VFYEKY-------LRNRFIYSLSKKKVYDLIKTELQNTDSLCIAPVNQAF	345
Candida	------SIL-SKWEA--------VRPKWLLNWVNKKVYDLIVKEYQNTEYLCIAPVSFAF	270
Verrucomicrobia	------PIV-RYLE--------KWIPSSVRRKALRLTYEHICYEDEQSDYIRQAPVNACY	235
Eudoraea	------WWL-KRID--------PLIPRFIKKKALALVYEHIEYEDENSNFIRQAPVNAVY	256
Plesiocystis	------AAG-RTLDRATDLPLLRPPLAALRRRALDRAWAHIEFEFHSTDWVCLSPVNGML	317
Enhygromyxa	------AGA-RALSRAI--------PAVVRRRALARAWEHIEFEFASTNWVCLSPVNGML	279
Bodo	------KIL-LAYE--------KVHSKKLREIALKRALEHIRYDDESTDYICLGPVNKVL	271
Trypanosoma	------VLL-KLYE--------KWHIKSLRRHALEVAWSHVAYDDEDTKFICLGPVNKAL	286
Phytomonas	------KVL-ELYE--------RRPISFLRRYALEKNWQHIAYDDENTHFICLGPVNKSL	219
Leishmania	------GLL-DLYE--------RHPIPFLRRYALEVSWRHMAYDDENTHFICLGPVNKWL	317
Leptomonas	------SLL-DLYE--------RHPLPFLRRYALEVNWRHMAYDDENTHFICLGPVNKSL	318
Labilithrix	------AAQ-RAVE--------RITPDRVRAMALGKVREHIAYEDEVTNFIDIGPVNKVL	257
Stigmatella	------QVM-YGYE--------RLAGKQLRARALDFALEQIRAEDEATHYICIGPINKVL	228
Myxococcus	------RVL-GLYE--------RLHSKRLRARAMAESLAQIRGEDEATNFLCIAFINKML	255
Corallococcus	------RVL-GWYE--------RFHSKRLRERALEESLELIRGEDEATHFVCIGPINKVL	264
S-cinnamoneus	------LGL-RGWE--------RMHSRRLRRAALDRCHRAVVDEQHASPVHGLSSVNALA	272
S-caatingaensis	------RLL-RGWE--------HIHSRALRRVALERCHRAVADEQHASPRHGLSSAGALV	248
Sandaracinus	------RVL-GAVD--------RLQSREKRATVLAELREHIRYELRSTNHTCISPVSGLL	264
S-alboviridis	------RAL-ALYE--------RRPPLALRRRALDRCRARVEHEHAVTGRLGLSPVNSLL	253
G-obscuriglobus	------RVM-GWYD--------RVALPGLRRAALKRCADLIDLDLDANGYLTLSPVNGTL	272
G-sp.SH-PL17	------RVM-GWYD--------RFPIRRLRESALQRCAHLIQLDLDVSDRLTLSPVNGVL	246
G-sp-IIL30	------RVM-GWYD--------RFPIRRLRESALQRCAHLIQLDLDVSDRLTLSPVNGVL	265
Methylococcus	------DLL-ARYE--------RRPWKALRQRALTLCFEQILREQRSTRYQGISPVSGLL	264
Methylocaldum	------RLL-AAYE--------RRHSATLRQKAIDLCFERILYEQRVTRYQGISPVSGLL	266

Helobdella	QMIVRYHRND----------NEALKKHRKRIDDYLWL-----GTDGLKMNGTNGSQLWDC	310
ScLDS	NMLIRYLEDGPD--------SEAFKKHVERVYDYVWM-----GLDGSNMQGTNGNQVWDV	312
AjLDS-b	NMLIRWLEDGPE--------SEAFKKHVERVYDYVWM-----GLDGTNVQGTNGNQVWDT	389
AjLDS-a	NMLIRWLEDGPE--------SEAFKKHVERVYDYVWM-----GLDGTNVQGTNGNQVWDT	389
PpLDS	NMLIRWLEDGPE--------SEAFKKHVERVYDYVWM-----GLDGTNVQGTNGNQVWDT	389
ScPS	NMLIRWLEDGPE--------SKAYQQHIDRVYDYIWM-----GRDGVNMQGTNGTQVWDV	312
PpPS	NMLIRWLEDGPD--------SKAFLQHVDRVYDYIWM-----GRDGVNMQGTNGTQIWDV	381
AjPS-b	NMLIRWLEDGPD--------SKAFLQHVDRVYDYIWM-----GRDGVNMQGTNGTQIWDV	381
AjPS-a	NMLIRWLEDGPD--------SKAFLQHVDRVYDYIWM-----GRDGANMQGTNGTQIWDV	381
Capsaspora	NMICQWYAHGPQ--------SPLFRQHVSRFPDYLWL-----GIDGMKLNGTNGSQLWDT	313
Saccoglossus-LSS	NMLVRWHVDGPQ--------SIVFKQHVDRIADYLWL-----GLDGMKMNGTNGSQLWDT	397
EpLSS	QMLVRWHVDGPE--------SDAFKMHRDRIPDYLWI-----GLDGMKMQGTNGSQLWDT	387
SpLSS	QMLARWHIDGAD--------SEAFKMHKDRLPDYLWI-----GLDGMKMQGTNGSQLWDT	387
SgLSS	QMLVRWHIDGPD--------SEAFKMHKDRIPDYLWI-----GLDGMKMQGTNGSQLWDT	387
LspLSS	NMLVRWITDGPD--------SEIFKQHVERISDYLWI-----GLDGMKMQGTNGSQLWDT	387
MgLSS	NMLVRWITDGPD--------SEIFKQHVERIPDYLWI-----GLDGMKMQGTNGSQLWDT	165
AaLSS	-MLVRWMTDGPD--------SAIFKQHVERIPDYLWI-----GLDGMKMQGTNGSQLWDT	46
AfLSS	NMLVRWMTDGPD--------SAIFKQHVERIPDYLWI-----GLDGMKMQGTNGSQLWDT	387
ArLSS	NMLVRWMTDGPD--------SAIFKQHVERIPDYLWI-----GLDGMKMQGTNGSQLWDT	387
HspLSS	NMLVRWMTDGPD--------SEVFKKHVQRIPDYLWI-----GLDGMKMQGTNGSQLWDT	387
EsLSS	NMLIRWMTDGPD--------SEVFKQHVQRIPDYLWI-----GLDGMKMQGTNGSQLWDT	387
ApLSS	NMLVRWLTDGPD--------SQVFKQHVERIPDYLWI-----GLDGMKMQGTNGSQLWDT	387
PpLSS	NMLVRWITEGAD--------SEIFKQHVERIPDYLWI-----GLDGMKMQGTNGSQLWDT	387
PmLSS	NMLVRWITEGAD--------SEVFKQHVERIPDYLWI-----GLDGMKMQGTNGSQLWDT	387
Amphimedon	QMLVRWFVDGPD--------STSFKEHLSRVPDYLWM-----GRDGIKMQGTNGSQLWDT	310
Aplysia	NMVIRYHEEGPT--------SKAFMLHQDRVQDYLWI-----GLDGMKMTGTNGSQSWDT	313
Capitella	QMLVRWHRDGPS--------HPVFLLHQDRVKDYLWL-----GLDGMKMTGTNGSQLWDT	313
Branchiostoma-LSS	QMLVRWHADGPT--------SPAFREHASRVADYLWL-----GLDGMKM-----------	347
Lingula	QMLIRWYVDGPS--------SAAFKQHQDRVQDYLWL-----GLDGMKMTGTNGSQLWDT	313
Callorhinchus	NMLVRWSVDGPQ--------SAAFQQHVERITDYLWL-----GVDGMKMQGTNGSQLWDT	312
HumanLSS	NMLVRWYVDGPA--------STAFQEHVSRIPDYLWM-----GLDGMKMQGTNGSQIWDT	389
Pteropus	NMLVRWHMDGPA--------SSVFQEHISRIPDYLWL-----GLDGMKMQGTNGSQVWDT	313
DreLSS	NMLVRWYVDGPT--------SPAFQKHVSRIPDYLWL-----GLDGMKMQGTNGSQLWDT	390
Haplochromis	NMLVRWYVDGPS--------SAAFQEHVSRIPDYLWL-----GLDGMKMQGTNGSQLWDT	313
Xenopus	NMLVRWHVDGSE--------SSVFREHVDRIPDYLWL-----GLDGMKMQGTNGSQLWDT	313
Charadrius	NMLVRWFVDGKN--------SPAFQEHVSRIPDYLWL-----GLDGMKMQGTNGSQLWDT	309
Anolis	NMLVSWYVEGDK--------SPAFQEHVSRIPDYLWL-----GLDGLKMQGTNGSQLWDT	313
Alligator	NMLVRWHVEGSS--------SLAFQEHVSRIPDYLWL-----GLDGMKMQGTNGSQLWDT	313
Pneumocystis	HILAVYWREGPN--------SYAFKSHKERLTDFLWV-----SKKGMMMNGTNGVQLWDT	315
Arthrobotrys_	QMLCRYIAEGPD--------SPAVLGHMDRLQDYLWV-----NEEGMLLNGTNGVQVWDT	315
Aspergillus	NMVACYLHDGPD--------SYSVRRHRERLNDYMWM-----KNEGMLMNGTNGVQVWDT	316
Spizellomyces	NMLCTFFRLGQD--------SEEFKKHKEHCLDYLWM-----SESGMHFNGTDGSQVWDT	310
Trichosporon	NMICRFAQEGPD--------SEAVRMHKSRVDDFLWL-----SKDGLFMTGTNGSQLWDI	301
Puccinia	NLIVCWDRYGPE--------SEEFRQHQLKLKNFLWM-----NKNGMGMSSTNGSQLWDL	318
Mixia	NMICRWDREGPD--------SEAFRLHKEKIRDFFWI-----SRDGMMMTGTNGSQLWDA	317
Microbotryum	HMICRWLEEGPD--------SDAFKAHLSCIRDFMWV-----SSQGMLMSGTNGSQLWDT	317
Dacryopinax	NQIVRFVVDGPE--------SSAFRLHVEKCRDFMWM-----TGAGMLMTGTNGSQLWDI	314
Fistulina	NLVVRAHVDGPE--------STAYKMHDIRRRDFMWL-----GAEGMMCCGTNGSQVWDT	314
Wallemia	NQLCRLDADGPD--------SNAVKKHLEKTNDFLWL-----GPDGLRETGTNGSQLWDA	315
Ceraceosorus	NYICRWIEEGPD--------SDVMAKHREKLKDFMWM-----GRDGMMMTGTNGSQLWDT	314
Ustilago	NYIARWDVQGHD--------SHAMRMHREKLKDFMWI-----GAEGMMMTGTNGSQLWDT	314
Malassezia_	NYIVRWIVDGPN--------SEAMERHREKLKDFAWM-----GAEGLMMTGTNGSQLWDT	314
Fluviicola	NSICVWHNHGRD--------SEQFKSHVKRWKDYLWI-----AEDGAKMSGYNGSQLWDT	312
Methylobacter	NSICIWHAYGKD--------STQFKKHTARWYDYLWV-----AEDGMKMNGYNGSQLWDT	312
Methylobactes	NSICIWHAYGKD--------STQFKKHTARWYDYLWV-----AEDGMKMNGYNGSQLWDT	312
Methylosarcina	NSICMWHACGKD--------SEPFKRHVARWYDYLWV-----AEDGMKMSGYNGSQLWDT	312
Aphanomyces	NMLVQWVVDPDS--------D-EFKRHAQRVEDYIWV-----AEDGVKMQGYVGSQTWDS	311
Cystobacter	HVLVWHFERPGG--------A-ELRAHLAQLPDYLWK-----GPDGVKMNGYNSSELWDT	309
Galdieria	NMLCCYFDDPYS--------E-HFKKHIPRLKDYLWL-----AEDGMKMQGYNGSQLWDT	313
Selaginella	NMLCCWAEDPNS--------E-AFKQHLPRIHDYLWV-----AEDGLKMQGYNGSQLWDT	317

```
Panax                  NMVCCWVEDPNS--------M-ANILHLSRIKDYLWV-----AEDGMKMKGYNGSQLWDV 327
Physcomitrella         NMLSCWIEDPNS--------E-AFKLHLARVVDYLWV-----AEDGMKMQGYNGSQLWDT 319
Cucumis                NMLCCWVEDPYS--------D-AFKFHLQRIPDYLWL-----AEDGMRMQGYNGSQLWDT 320
Adiantum               NMLCCWIEDPNS--------E-AFKCHLPRIPDYLWV-----AEDGMKMQGYNGSQLWDT 320
Abies                  NMLCCWVEDSNS--------E-AFKRHLARVVDYLWV-----AEDGMKMQGYNGSQLWDT 321
Malus                  NMLCCWAEDPNS--------E-AFKLHLPRIPDYLWI-----AEDGMKMQGYNGSQAWDT 318
Amborella              NMLCCWVEDPNS--------E-AFKLHLPRILDYLWV-----AEDGMKMQGYNGSQLWDT 320
Nicotiana              NMLCCWAEDPNS--------E-AFKLHIPRLYDYLWI-----AEDGMKMQGYNGSQSWDT 318
AtCAS1                 NMLCCWVEDPNS--------E-AFKLHLPRIHDFLWL-----AEDGMKMQGYNGSQLWDT 418
Glycine                NMLCCWVEDPNS--------E-AFKLHLPRIYDYLWI-----AEDGMKMQGYNGSQLWDT 319
Coffea                 NMLCCWVEDPNS--------E-AFKLHLPRIYDYLWI-----AEDGMKMQGYNGSQLWDT 319
Populus                NMLCCWVEDPNS--------E-AFKLHLPRIQDYLWL-----AEDGMKMQGYNGSQLWDT 319
Vitis                  NMLCCWVEDPNS--------E-AFKLHLPRIFDFLWL-----AEDGMKMQGYNGSQLWDT 319
Micromonas             NMLSCWFQDPSE--------FGSWRKHTPRVADYLWL-----AEDGMKMAGYNGSQLWDC 319
Volvox                 NMLCCWLEDPNG--------L-PYKKHLVRVADYLWV-----AEDGLKMQGYNGSQLWDT 346
Chlorella              NMLACWFEDPGS--------Q-AFKRHLPRLLDYLWV-----AEDGMKMQGYNGSQLWDT 319
Synchroma              NMLAVWVDAGRDPT-----CE-AFRRHLQRVDDFLWV-----AEDGMKMQGYNGSQCWDT 322
Chattonella            NMLCVWMERGS--D-----AE-EFQRHICRVDDYLWV-----AEDGMKMQGYNGSQCWDT 315
Ochromonas             NMLSVWVDNGENSN-----EE-KFLRHAARVDDYLWV-----AEDGMKMQGYNGSQCWDT 322
Chromulina             NMLSVYISSNGDSN-----NE-RFKRHIPRIDDYLWV-----AEDGMKMQGYNGSQCWDT 325
Chondrus               NFLCRWFDDPDG--------V-AVDKHRDRLTDYLWL-----AEDGMKMQGYNGSQLWDT 313
Acanthamoeba           NMLCSWHAQGKD-------SK-EFQRHVDRVPDYLWL-----AEDGMKMQGYNGSQLWDT 314
Dictyostelium          NMLCVWDREGKS--------P-AFYKHADRLKDYLWL-----SFDGMKMQGYNGSQLWDT 307
Polysphondylium        NMLVVWDREGQS--------P-NFFKHADRLYDYLWL-----ASDGMKMQGYNGSQLWDT 310
Acytostelium           NMLCVWDREGQS--------P-NFFKHADRLQDYLWL-----ANDGMKMQGYNGSQLWDT 307
SHC-Candidatus         YFIMALDALGYAED------HPDRSEAIRHFESLLIE-----TDDR-FLFQPCVSPVWDT 243
SHC-Chlorogloeopsis    NSLLALRSLDYDVN------DPIVERGLQAVDNFAIE-----TEDS-YRVQPCISPVWDT 297
SHC-Streptomyces       YSIIALHLLGYDLD------HPVLRAGLASLDRFTVWDKTEDGEPV-RMVEACQSPVWDT 295
SHC-Hyphomicrobium     NSLLVYDALGYPQD------HPDYVTARGSIEKLLVV-----KDDE-AYCQPCLSPVWDT 296
SHC-Leptospirillum     NSAVALSLEGYELD------HPLMQRVLASIDDLLIE-----GEKE-VLVQPCVSPVWDT 264
Naegleria              NMLSVFFSDGSD--------SELFNKHLDRVYDYLWL-----SDDGMKFQGYNGSQLWDT 323
Symbiodinium           HLLVSWVEAGGDAK---ASESRSFQAHVQRVPAYLWV-----AEDGMKVQGYNGSMAWDT 278
Prorocentrum           -------ASGGEADAASARESRAFRAHVERIPAYLWV-----AEDGMKAQGYNGSMMWDT 239
Gymnodinium            HIVCMWVIEGGETDPKAAGNSKAFRAHVARVPA--------------------------- 194
Alexandrium            HIVVSWASAGGQRDPETASQSKAFLAHVARVPAYLWI-----AEDGMKVQGYNGSMAWDT 328
Dinophysis             HVLTSWVAAGGAEDPGLARKSDAFRAHVARIPAYSWI-----AEDGLKVQGYNGSMAWDT 332
Scrippsiella           HIVTAWVAAGGEKDALAASKSKAFRAHLARIPAYMWV-----AEDGLKVQGYNGSMAWDT 330
Aureococcus            NMLVAYDDRHADG-------GEALARHALRVPDYLWV-----AEDGMKMQGYNGSQCWDA 323
Phaeodactylum          NMLSAFHHAGNDLH------HSTVMNHMIRVQDYLWV-----AEDGMKMKGYNGSQCWDT 320
Helicotheca            NMVSQFHAAGNDVT------APKVKKHMMRIPDYLWL-----AEDGMKMQGYNGSQCWDT 327
Yeast-erg7             CALVTLIEEGVD--------SEAFQRLQYRFKDALFH-----GPQGMTIMGTNGVQTWDC 392
Candida                NMVVTCHYEGSE--------SENFKKLQNRMNDVLFH-----GPQGMTVMGTNGVQVWDA 317
Verrucomicrobia        NTLAHFV-EGQ---------TSRVARSWEQLPRYLWN-----HPDHIACQGFTSSKVWDT 280
Eudoraea               NTLVYHF-KGE---------KEKLEKSWAKLPLYLWE-----NDDQILMQGFTNTYTWDC 301
Plesiocystis           FCLAMAK-RDPD--------HPDLDRALAGLEYWVWE-----DAEEGARICGARSDIWDT 363
Enhygromyxa            FCLAMWS-RDRR--------DPRLAQALQGLEYWMWE-----DDSEGLRICGARSDIWDT 325
Bodo                   DMLITWLVDGED--------SAAFKRHVERLEDYFYI-----GYDGMRMSGYNGSQLWDT 318
Trypanosoma            DMLLTWIREGEN--------SGRYQNHVSRLADYIYM-----GPEGMRVCGYNGSQLWDT 333
Phytomonas             NMLVTWIREGEN--------SVRYQLHRQRVDDYFFM-----SPYGMHMSGYNGSQLWDT 266
Leishmania             NMLATWLREGEH--------SARFQEHFDRVSDYFYM-----GETGLSMSGYNGSQLWDT 364
Leptomonas             NMLVTWIREGEH--------SARFQEHCDRVADYLYM-----GPTGMRMSGYNGSQLWDT 365
Labilithrix            NAFVAFF-DDPK--------SEAFERAFATCESYLFD-----NELGTMMQGYNSSKLWDV 303
Stigmatella            NMVVWHF-VNPD--------GPEVRAHLERLPDYFYE-----GDDGVRMNGYNSSELWDT 274
Myxococcus             DTVVWHL-EKPD--------GPEVRAHLAKLPTYLQP-----TPEGLALNSYNSSQLWDT 301
Corallococcus          DMVVWHV-ARPD--------GPEVRAHLERLPDYLQH-----THEGVAVNGYNSSQLWDT 310
S-cinnamoneus          ECLVLFA-HDRR--------HPLLDAAVARLEYWVWS-----DDKQGLRLCGDRTAVWET 318
S-caatingaensis        ECLALYA-RDPG--------HPLLEGAVTRLSHWRWT-----DARDGVRLRDDRSTVRDT 294
Sandaracinus           DQVALFI-EDPN--------DPDLRIAAERFEGWVWE-----DELDGARVTGARSASWDT 310
S-alboviridis          DILVLHA-SGEK--------PDKVDRSLTAFDYWRWT-----DPHEGSRYAGARSQTWDT 299
G-obscuriglobus        NALALFA-RGAD--------REVIAKCVSGFEFYRFD-----DPERGLRYSGGSTRTWDT 318
G-sp.SH-PL17           NVLALHA-RGAD--------RTLIAKCVEGFEAYRWD-----DAARGLRYSGGRTRVWDT 292
G-sp-IIL30             NVLALHA-RGAD--------RALIAKCVEGFEAYRWD-----DAARGLRYSGGRTRVWDT 311
Methylococcus          NCLAIFA-HDPR--------HPDLAPSLEGVEAWRWE-----DEAEGLRYVGARSNAWDT 310
Methylocaldum          NCLAIFA-RDPT--------HPDLGPSIEGVEAWRWE-----DEENGIRYAGARSNTWDT 312


Helobdella             TFAMQALLEVTLK-N--GLKF-----------------------LLATQIDGLNNNNNNN 344
ScLDS                  SFAGMAMLDVGVQ-D--DPRFHEV-------------LRKTYSYLEISQVI--------- 347
AjLDS-b                SFAAMAMLDVGAQ-D--DPQFHEV-------------LCKTYSYLEISQVI--------- 424
AjLDS-a                SFAAMAMLDVGAQ-D--DPQFHEV-------------FCKTYSYLEISQVI--------- 424
PpLDS                  SFAAMAMLDVGAQ-D--DPQFHEV-------------LSKTYSYLEISQVI--------- 424
ScPS                   SFAVMAMLECGAN-L--RPQFQNV-------------LQKAYLYLEVSQMV--------- 347
PpPS                   SYAAMAMLEAGAG-T--KPEFQEA-------------LRKAYSYLEVSQMV--------- 416
AjPS-b                 SYAAMAMLEAGAG-T--KPEFQEA-------------LRKAYSYLEVSQMV--------- 416
AjPS-a                 SYAAMAMLEAGAG-T--KPEFQEA-------------LRKAYSYLEVSQMV--------- 416
Capsaspora             AFAVQAFLEAGGD-K--NAQLMAS-------------LRKAHSFFKLTQIR--------- 348
Saccoglossus-LSS       AFVVQAFIEAEAH-K--NTMFNDT-------------LEHAHSFFKLTQIP--------- 432
EpLSS                  AFAVQAFIEAGAN-T--EEEFRDT-------------LRNAHGFLRNTQIP--------- 422
SpLSS                  AFVIQAFLEAGG-----EGEFKDT-------------LSKAHDFLKNTQIP--------- 420
SgLSS                  AFVIQAFLEAGA-----VPDFKDT-------------LINAHDFLKNTQIP--------- 420
LspLSS                 AFAAQAFLEGGAA-K--NESLHQC-------------LQRTHDFFKLTQIP--------- 422
MgLSS                  AFAAQAFLEGGAS-K--IESLHQS-------------LQRTHDFFKLTQIP--------- 200
AaLSS                  AFAAQAFLEGGAA-K--NESLHEC-------------LQRTHDFFKLTQIP--------- 81
AfLSS                  AFAAQAFLEGGAA-K--NESLHEC-------------LQRTHDFLKLTQIP--------- 422
ArLSS                  AFAAQAFLEGGAA-K--NESLHEC-------------LQRTHDFFKLTQIP--------- 422
HspLSS                 AFAAQALLDAGAS-T--NADLHDC-------------LQRTHDFLKLSQIP--------- 422
EsLSS                  AFAAQALLEAGAS-T--NPDLQDC-------------LQRTHDFLKLTQIP--------- 422
ApLSS                  AFAAQAFLEAGAA-K--NKELHDC-------------LQHTHDFLKLTQIP--------- 422
```

```
PpLSS                    AFTAQAFLEAGAA-T--NKELHEC-------------LQRTHDFLKLTQIP--------- 422
PmLSS                    AFAAQAFLEAGAA-T--NKELHEC-------------LQRTHDFLKLTQIP--------- 422
Amphimedon               AFAVQAILEASSF-D--TNKHSHS-------------LSMAHSFLLSTQVP--------- 345
Aplysia                  SFAATAFLEAGAY-K--YEELKKC-------------LTACHDFLRITQVP--------- 348
Capitella                AFAIQMYMGCGAN-K--RKEFRDT-------------LDNAHAWLKLTQIP--------- 348
Branchiostoma-LSS        ------------------------------------------------QIP--------- 350
Lingula                  AFAVQAFMEAEAF-E--YRDFQNG-------------LNHAHKFLKNTQIE--------- 348
Callorhinchus            AFTVQAFLEAGAQ-N--CPEFTTS-------------LTLAHQYLRITQVQ--------- 347
HumanLSS                 AFAIQALLEAGGH-H--RPEFSSC-------------LQKAHEFLRLSQVP--------- 424
Pteropus                 SFTIQALLEADAQ-H--RPEFASC-------------LQKAHEFLQMSQVP--------- 348
DreLSS                   AFAVQAFLEAGAQ-D--IPRFTEC-------------LTQAHHFLDLTQVK--------- 425
Haplochromis             CFAVQAYLEAGAQ-D--DPKLAEC-------------LRDAHQFLTITQIP--------- 348
Xenopus                  AFAVQAYLEAGAH-R--RKEFQNC-------------LEKAHEFLRISQIP--------- 348
Charadrius               AFAIQAFLEAEAQ-K--MPEFTSC-------------LQNAYEFLRFTQIP--------- 344
Anolis                   AFAVQAFLEAGAH-K--KPEFNSC-------------LLHAHEFFRISQIP--------- 348
Alligator                AFAIQAFLEAGAQ-D--NPEFSSC-------------LRRAHEFLRISQIP--------- 348
Pneumocystis             SFAVQALVESGLA-E--DPEFKDS-------------MIRALDFLDKCQIQ--------- 350
Arthrobotrys_            SFAVQSVVECGFS-T--QPEWKDM-------------LNSAHQFLEDQQIS--------- 350
Aspergillus              AFITQAIVVAGFA-D--DPKWRPM-------------LTKALEFLEDHQLR--------- 351
Spizellomyces            SFLSQAVCEAGLA-E--EPEFQTT-------------MQRALDFLDSQQIR--------- 345
Trichosporon             SFLAQAVVETGLA-D--EAENKQT-------------VLGMLDWLDKCQIR--------- 336
Puccinia                 AFITQALVESGLA-KTDEPSTRDS-------------VIKALEWIDRCQIL--------- 355
Mixia                    GFIGQAIADTGLA-D--EEENHAS-------------ALKLLDWLDRAQIR--------- 352
Microbotryum             SFIGQALVESNLA-L--EPSNKKS-------------LEKMLDWLDHSQIR--------- 352
Dacryopinax              AFICQAVVETGLG-K--EPGNEKA-------------TVGALKWLDGAQMR--------- 349
Fistulina                GFICQALQETGIG-A--LEENRDD-------------VVRALQWFEQAQIL--------- 349
Wallemia                 AFIAQALVETGLG-E--EEENKQA-------------TEKLLEWIDKCQIR--------- 350
Ceraceosorus             AFIAQAMVDSGLV-D--DDDTKNS-------------CYRILDWLDEHQIR--------- 349
Ustilago                 AFIAQAMVDSGLA-A--SPENKQL-------------TQKLLDWLDDCQIR--------- 349
Malassezia_              SFIAQAMCESGLS-A--KPANHEI-------------CRRILSWLDMCQIR--------- 349
Fluviicola               GFAGQALIEADME-T----DFPEM-------------AEKIYRFIDSTQIE--------- 345
Methylobacter            AFATRAMLESDLG-K----LFPAT-------------IANSYRFIDQSQIK--------- 345
Methylobactes            AFATRAMLESDLG-K----LFPAT-------------IANSYRFIDQSQIK--------- 345
Methylosarcina           AFATRAMLESDLG-K----LFPAT-------------IAKSYRFIELSQIQ--------- 345
Aphanomyces              SFAVQAFVDAGVA-A--DPAFQTT-------------FKLAYRFLTEAQNV--------- 346
Cystobacter              AFAAQAVVASGRI-Q----ENLPF-------------LRSAFDFIDRSQVR--------- 342
Galdieria                AFSAQALCEAGSI-T--RHHFPST-------------LQLAHHYLDIAQVR--------- 348
Selaginella              AFAVQAIISTDML-E----ESREM-------------LKKAHSYIEKSQVR--------- 350
Panax                    GFAVQAILSTGLV-D----EYGSM-------------LKKAHDFIKISQVR--------- 360
Physcomitrella           TFAVQALAATKLP-D----ESMSM-------------LKKANSYIDNSQVR--------- 352
Cucumis                  AFSIQAIISTKLI-D----TFGPT-------------LRKAHHFVKHSQIQ--------- 353
Adiantum                 SFAVQALISTGLL-E----TCGPM-------------LKKAHHFIDRSQVR--------- 353
Abies                    AFATQALISTNLL-D----DCGPL-------------LKKAHIYIERSQVQ--------- 354
Malus                    SFAIQAIISTNLV-E----EYGPT-------------LRKAHQYIKDSQVV--------- 351
Amborella                AFAVQAIISSNLV-Q----EYYPA-------------LKKAHDYIKYSQVL--------- 353
Nicotiana                SFAIQAIISTNLG-E----DYGPT-------------LRKAHTYMKDTQVL--------- 351
AtCAS1                   GFAIQAILATNLV-E----EYGPV-------------LEKAHSFVKNSQVL--------- 451
Glycine                  AFAVQAIIASNLI-E----EFGPT-------------IRKAHAYIKNSQVL--------- 352
Coffea                   AFAVQAIISTNLA-E----EYGPT-------------LRKAYTFIKNSQVL--------- 352
Populus                  SFAVQAIISTKLV-E----EYGPT-------------LRKAHAYIKNSQVL--------- 352
Vitis                    AFCVQAIISTDLV-E----EYGPT-------------LRKAHAYLKKSQVL--------- 352
Micromonas               AFAVQAIVATGLH-V----EYSAC-------------LRSAHKYIRDSQVL--------- 352
Volvox                   SFAVQALAEAGLL-D----VTAAS-------------LARAHAYVEQSQVV--------- 379
Chlorella                AFAVQAIASTGMA-G----EFSRC-------------LKRAHEYLEQSQVV--------- 352
Synchroma                SFAIQGIVEAG---L--DAEFPDM-------------VRRVYSYLDRTQIA--------- 355
Chattonella              SFAIQGIVESG---L--AEEFPEM-------------CLKVYEYLDNTQIK--------- 348
Ochromonas               AFATQAIVESG---L--APHFKDC-------------LTKSYIYFNRTQIK--------- 355
Chromulina               SFTIQAIIESN---L--SNKFSNC-------------LTKAYSYLNRTQIA--------- 358
Chondrus                 AFASQAFMAAGRD-I--VEPFTKT-------------LSLAHDYVEMTQVL--------- 348
Acanthamoeba             AFSVQAIIETG---L--GDQFQEC-------------LQKAYSYIDITQVR--------- 347
Dictyostelium            AFTIQAFMESG---I--ANQFQDC-------------MKLAGHYLDISQVP--------- 340
Polysphondylium          AFTIQAFVESG---I--SHQFPEA-------------MRMANHYLDITQVP--------- 343
Acytostelium             AFTIQAFVETG---I--AGQFPDT-------------MRLANHYLDISQVP--------- 340
SHC-Candidatus           AICAFALGEAGNT-------DDPR-------------MTLAADWLISKE-VRR------- 275
SHC-Chlorogloeopsis      AWAIRALVDSGLS-----P-AHQA-------------LVKAGEWLLNKQ-ILD------- 330
SHC-Streptomyces         CLATVALADAGLE-----P-DHPA-------------LVKAADWMLEEQ-ITR------- 328
SHC-Hyphomicrobium       ALAAHALMESGGA-----Q-TEQS-------------VDRALAWLKPLQVLDT------- 330
SHC-Leptospirillum       ALAMGALIEAGIS-----P-DSPT-------------VDRAMEWFCARE-VRT------- 297
Naegleria                AFASQAICEYYKR-F--KNQDNQISCQGSFKNEHLNSLLNAYNFINFTQVK--------- 371
Symbiodinium             SFAMQAAVDSDLV-S--E--FKDM-------------SKKAWSWLVKEQVRSLPY----- 315
Prorocentrum             SFAVQAVAEAGLQ-G--E--FADM-------------SKRAFEFICREQVRALPR----- 276
Gymnodinium              --------EAGLD-G--E--FSEM-------------CCKAHGWFVREQVRALPN----- 223
Alexandrium              SFAVQAVAEAKLQ-D--E--FPEM-------------CAKACGFLAREQVRVLPN----- 365
Dinophysis               SFAVQAAVEAGLQ-G--E--FPDM-------------CARAWGWIARDRTATGGT----- 369
Scrippsiella             SFAIQAASEAGLQ-K--E--FPEM-------------CAKAWGFLAREQVRALPE----- 367
Aureococcus              SFATQAIAESDLG-D--DARFRDC-------------AKKAWSYLERTQILSTTTSQASP 367
Phaeodactylum            SFAIQAVFEAGLL-D--D--FPEL-------------SNKVWTYLERCQILSTEVSQASP 362
Helicotheca              SFAIQAVYECDLL-D--E--FPEL-------------SKKVWSYLERTQILSTEVSQNTP 369
Yeast-erg7               AFAIQYFFVAGLA-E--RPEFYNT-------------IVSAYKFLCHAQFDTE------- 429
Candida                  AFMVQYFFMTGLV-D--DPKYHDM-------------IRKSYLFLVRSQFTEN------- 354
Verrucomicrobia          AFTLQGMSHLE---P--SLAPKQS-------------IQEGCRYLVENQVIDE------- 315
Eudoraea                 GFYLQAIGKDA---T--EPALKPL-------------AIKTRKFLLENQVTKE------- 336
Plesiocystis             SFALQALAEGP---E--LELGRAL-------------AQRAAAWLPKAQVMRELS----- 400
Enhygromyxa              SFALQALCEGP---Q--IDEAARA-------------VDRASAWLPRAQLRAD------- 360
Bodo                     SFAVQAAVACG---V--EQQFERE-------------MAKAYHYVDVAQVRED------- 353
Trypanosoma              SFAVQAVCACN---M--ELLYPQQ-------------MSLAHHYVDVAQVQKD------- 368
Phytomonas               SFAVQATCACK---S--EMLFANE-------------MALAHHYIDIAQVRDD------- 301
Leishmania               SFAVQAICACR---R--EMMFPAE-------------VELAHHYIDVAQVQED------- 399
```

```
Leptomonas          SFAVQAICACG---R--EMMYPME-------------MALAHHYVDVAQVQED------- 400
Labilithrix         AFATQAIVATP---L--GDEHRTM-------------LERAYGYVRDNQILDD------- 338
Stigmatella         AFAVQAVAATG---E--TGRHRRM-------------LEEAARFIEANQVLED------- 309
Myxococcus          TFAIQALVASG---A--D-HAREA-------------LARAGRFVEAQQVRED------- 335
Corallococcus       AFAVQALVAAG---E--SAWARDT-------------LERAGRFLEAQQVLED------- 345
S-cinnamoneus       SFAVSALLASGAEHR--SLQTGPA-------------LLRASAYLRDAQITTY------- 356
S-caatingaensis     AFAVQALLAADPGHV--P----DA-------------VHRARTRLAAARVTTY------- 328
Sandaracinus        AFAAQAMAAA-APHC--GNDVRDA-------------LRRADTFLVTQQIPRG------- 347
S-alboviridis       AFAVEALLAD-HA-P--CPDTARA-------------VARANRFLTGAQITTE------- 335
G-obscuriglobus     GFALEALLAN-PA-V--ASVYRDV-------------VHRGYRFLAAHQMSKS------- 354
G-sp.SH-PL17        GFAVESLLAD-PG-A--VRANRDA-------------LLRAYRFLAEQQVMKP------- 328
G-sp-IIL30          GFAVESLLAD-PG-A--VRANRDA-------------LLRAYRFLAEQQVMKP------- 347
Methylococcus       AFAVQALAEL-PE-L--DEEAKHA-------------LSRAHAFLDQAQMTAE------- 346
Methylocaldum       AFAIQALVDA-PA-N--VEGTAEA-------------LERAHEFLKNAQTTEE------- 348
                                                                   .
```

```
Helobdella              N-RTSVYRD-FSKYYRHPNKGGFPFSTYECGWIVADCTAEALKTMMMMRRRRKKKKM--M 400
ScLDS                   ----ESAPD-CVKYYRQYNKGGWAQTMREHGLVVSDTSAEALKAVLMMNEKCP-F----- 396
AjLDS-b                 ----ESSPD-CVKYYRQYNKGGWAQTMREHGLVVSDTSAEALKAVLLMNDKCP-F----- 473
AjLDS-a                 ----ESSPD-CVKYYRQYNKGGWAQTMREHGLVVSDTSAEALKAVLLMNDKCP-F----- 473
PpLDS                   ----ESSPD-CVKYYRQYNKGGWAQTMREHGLVVSDTSAEALKAVLLMNDKCP-F----- 473
ScPS                    ----ENSPK-CLQYHRQFNKGGWPLTTRDHGLIVSDTTAEALKAALLIEEKCP-F----- 396
PpPS                    ----ESSPK-CVQYYRQYNKGGWPLTTRDHGLIVSDTTAEALKAAMLIEEKCP-F----- 465
AjPS-b                  ----ESSPK-CVQYYRQYNKGGWPLTTRDHGLIVSDTTAEALKAAMLLEEKCP-F----- 465
AjPS-a                  ----ESSPK-CVQYYRQYNKGGWPLTTRDHGLIVSDTTAEALKAAMLLEEKCP-F----- 465
Capsaspora              ----KNVPE-HEKYFRQMSKGGFPFSTRDCGWIVADCTAEGIKSTLMLENTGQ------- 396
Saccoglossus-LSS        ----ENPAD-YEKYYRQMNKGGFPFSTRDCGWIVGDCTAEGLKSVMMLQEKCA-N----- 481
EpLSS                   ----DNPPD-YQKYYRQMNKGGYPFSTKDCGWIVADCTAEGLKSAMLLEEKCP-F----- 471
SpLSS                   ----ENPPN-YQKYYRQMSKGGYPFSTKDCGWIVSDCTAEGLKSAMLLEEKCP-F----- 469
SgLSS                   ----DNPPN-YQKYYRQMNKGGYPFSTKDCGWIVSDCTAE-------------------- 455
LspLSS                  ----ENPPN-YQKYYRQMNKGGYPFSTRDCGWIVADCTAEGLKSALMIEQHCP-F----- 471
MgLSS                   ----ENPPN-YQKYYRQMNKGGYPFSTRDCGWIVADCTAEGLKSALMIEQLCP-F----- 249
AaLSS                   ----ENPPN-YQKYYRQMNKGGYPFSTRDCGWIVADCTAEGLKSALMIEQRCP-F----- 130
AfLSS                   ----ENPPN-YQKYYRQMNKGGYPFSTRDCGWIVADCTAEGLKSALMIEQRCP-F----- 471
ArLSS                   ----ENPPN-YQKYYRQMNKGGYPFSTRDCGWIVADCTAEGLKSALMIEQQCP-F----- 471
HspLSS                  ----ENPPN-YQKYYRQMNKGGYPFSTRDCGWIVADCTAEGLKAALMLETQGT-A----- 471
EsLSS                   ----ENPPN-YQKYYRQMNKGGYPFSTRDCGWIVADCTAEGLKAALMLETMGT-V----- 471
ApLSS                   ----DNPPN-YQKYYRQMNKGGYPFSTRDCGWIVADCTAEGLKAALMLETMCP-S----- 471
PpLSS                   ----DNPPN-YQ------------------------------------------------ 429
PmLSS                   ----DNPPN-YQKYYRQMNKGGYPFSTRDCGWIVADCTAEGLKAALMLETMCP-T----- 471
Amphimedon              ----DNPPD-YKKYYRQMSKGGFPFSTLECGWIVSDCTAEGLKSLMLLEEECRSF----- 395
Aplysia                 ----ENPPD-YKKYYRQRNEGAFPFSTRDCGWIVSDCTAEGLKAVIKLEEKCQ------- 396
Capitella               ----ENPPE-YSKFYRQMSRGGFPFSTKDCGWIVADCTADGLKAVLALQENCD-F----- 397
Branchiostoma-LSS       ----DNPPD-YETYYRQMNKGGFPFSTRDCGWIVTDCTAEGLKSMMMLQERCQ-G----- 399
Lingula                 ----ENPPE-YQKIYRHQNKGGFPFSTRDCGWIVTDCTAEGLKGVMLLQEKCD-F----- 397
Callorhinchus           ----EAPPD-YDKYYRQLNKGGFSFSTRDCGWVVADCTAEALKSLLLLQETCP-F----- 396
HumanLSS                ----DNPPD-YQKYYRQMRKGGFSFSTLDCGWIVSDCTAEALKAVLLLQEKCP-H----- 473
Pteropus                ----DNPPD-YQKYYRQMCKGAFPFSTLDCGWIVADCTAEALKSILLVQEKCP-F----- 397
DreLSS                  ----DNPPE-YEKYYRQMNKGGFPFSTRDCGWIVADCVSEGLKSVMLLQEQCN-F----- 474
Haplochromis            ----ENPPQ-YQKYYRQMNKGGFPFSTRDCGWIVADCTAEGLKSLMLLQELCP-S----- 397
Xenopus                 ----DNPPD-YKKYYRQMNKGGFPFSTRDCGWIVADCTAEGLKSVMLLQEQCP-F----- 397
Charadrius              ----ENPPD-YQKYYRHLNKGGFPFSTRDCGWIVADCTAEGLKSVMLLQEKCP-F----- 393
Anolis                  ----DNPPD-YEKYYRQMNKGGFPFSTRDCGWIVADCTAEGMKSVMLMQEKCP-F----- 397
Alligator               ----DNPPD-YQKYYRQMNKGGFPFSTRDCGWIVADCTAEGLKSVMLLQEKCP-F----- 397
Pneumocystis            ----KNCD-DQQKCYRHQRKGAWPFSTRQQGYTVSDCTAEGLKAVLLLQNL---KS---- 398
Arthrobotrys_           ----LEISDEKQKCYRQQRKGAWAFSTRDQGYTVSDCTAEALKAVIQIQMT---PG---- 399
Aspergillus             ----ENVP-DQEKCYRQHRKGAWPFSNKTQGYTVSDCTAEGLRSTIQLQEM---HN---- 399
Spizellomyces           ----TDPI-NRKRCYRDATKGAWPFSRRDQGYTVSDCTAEGMKSVLYLQDL---EY---- 393
Trichosporon            ----HNPK-WYRAGYRQSSKGAWPFSTPEQSYTVSDCTAEGLKGVIALQSL---SY---- 384
Puccinia                ----DNPK-HFKSGYRHQTKGAWPFSTKSQGYTVSDCTAEALKSVLCLQEQL--SY---- 404
Mixia                   ----TNPR-HYEQAYRHTTQGAWPFSTKEQGYTVSDCTAEGLKAVIYLSKL---KY---- 400
Microbotryum            ----ENPP-HFEKAYRHRTMGAWPFSTKEQGYTVSDCTAEGLKTVIMLQGL---PG---- 400
Dacryopinax             ----ENPK-HMQEAFRHQTKGAWPFSTPEQGYTVSDCTGEGLKAVIYLQNHL--DY---- 398
Fistulina               ----ENPK-HYHSAYRHATTGAWGFSTKEQGYTVSDCTSEGLKAVMLLQHEL--DF---- 398
Wallemia                ----QDPL-Y--PAYRQPTKGAWPFSTAEQGYTVSDCTAEGIKSVIYLQEYL--KH---- 397
Ceraceosorus            ----DNPK-HYNAAYRYSSKGAWPFSTKEQSYTVSDCTGEGMKATIMLQND---AK---- 397
Ustilago                ----SNPQ-HHKTCYRFATKGAWPFSTKEQGYVVSDCTGEGLKAVIMLQENV--AY---- 398
Malassezia_             ----ENPR-FHRTAYRFATKGAWPFSTREQGYTVSDCTAEGLKGVLMLQEASG-AS---- 399
Fluviicola              ----RNAKD-YSKYWGDVTLGCWPFSTIDHGWAITDCTSEGMKTAILYNSREH-IQ---- 395
Methylobacter           ----AEHPT-HAEFFRHPMIGSWPFSTPDNGWPVADCTAEGLSAALAVHHSGL-V----- 394
Methylobactes           ----AEHPT-HAEFFRHPMIGSWPFSTPDNGWPVADCTAEGLSAALAVHHSGL-V----- 394
Methylosarcina          ----SEHST-HAEFFRHPMIGSWPFSTAENGWPVADCTAEGLSAALAVHHSGL-I----- 394
Aphanomyces             ----HDPKD-AATWWRRVQKGGWGFGIASNGYPVSDCTAEAVKALLLMEERAN------- 394
Cystobacter             ----EDTPN-AERYYRHRSKGGWPFSTRDHGWPISDCTAEGLKAALALEPF--------- 388
Galdieria               ----ENVPQG-ERYYRHISKGAWPFSTRDHGWPISDCTAEGLKAVLALEACGS-IP---- 398
Selaginella             ----EDCPGDLDYFHRHISNGAWPFSNRDHGWPISDCTAEGLKSVLLLSRISS-DV---- 401
Panax                   ----EDSPGNLSSWNRHISKGGWPFSTPDNGWPVSDCTAEGLKAALLLSNMPF-DI---- 411
Physcomitrella          ----EDSPGDMAYWYRHISKGAWPFSSRDHGWPISDCSSEGLKATLILADFPK-EL---- 403
Cucumis                 ----EDCPGDPNVWFRHIHKGAWPFSTRDHGWLISDCTAEGLKASLMLSKLPS-KI---- 404
Adiantum                ----NDCPGDLQFWYRHISKGAWPFSTRDHGWPISDCTAEGFKAALALSQLPS-DI---- 404
Abies                   ----EDCPGDLNFWYRHISNGAWPFSTRDHGWPISDCSSEGLKAALALSQLPQ-DI---- 405
Malus                   ----EDCPGDLDYWYRHISKGAWPFSTADHGWPISDCTAEGLKAALLLSKLPT-ET---- 402
Amborella               ----ENCYGDLQFWYRHISKGAWPFSTRDHGWPISDCTGEGLKVALLLSQISH-DA---- 404
Nicotiana               ----EDCPGNLDFWYRHISKGAWPFSTADHGWPISDCTAEGLKVCLQLSKLPM-EI---- 402
AtCAS1                  ----EDCPGDLNYWYRHISKGAWPFSTADHGWPISDCTAEGLKAALLLSKVPK-AI---- 502
Glycine                 ----EDCPGDLNKWYRHISKGAWPFSTADHGWPISDCTAEGLKAVLLLSKIAP-EI---- 403
Coffea                  ----DDCPGDLDSWYRHISKGAWPFSTADHGWPISDCTAEGLKAYFSLSKLPS-EL---- 403
Populus                 ----EDCPGDLSFWYRHISKGAWPFSTADHGWPISDCTAEGLKAALLLSKITP-EI---- 403
Vitis                   ----EDCPGNLDYWYRHISKGAWPFSTGDHGWPISDCTAEGLKAILLLSKIPS-EI---- 403
Micromonas              ----DDCPGQLSRRFRHISKGAWPFSTRDHGWPISDCSSEGLKAALELEAMGS-ER---- 403
Volvox                  ----EEAAPPLDRYYRHISKGAWPFSTRDHGWPISDCSSEGLKAALALAGLPA-DK---- 430
Chlorella               ----EEAQQPLSEYYRHISKGAWPFSTRDHGWPISDCSSEGLKAALVLAQMDP-KL---- 403
Synchroma               ----TDEDR-AEFWFRHVSKGGWPFSTSQHGWPISDCTAEGLKAVLALADSPC-VR---- 405
Chattonella             ----TNEEN-REHWFRHISLGGWPFSTAAHGWPISDCTSEGLKGILSLNSLQF-IK---- 398
Ochromonas              ----NDEDN-RDYYYRHISKGGWPFSTAAHGWPISDCTAEGLKAVLCMHSSEK-LKA--L 407
Chromulina              ----EDEVN-REYYYRHVSKGGWPFSTAAHGWPISDCTAEGLKSVLLVHTLDT-IS---- 408
Chondrus                ----VDVPN-RERFYRHMSKGAWPFSTRDHGWPIADCTGEGLAAALLLQGKKG-TW---- 398
Acanthamoeba            ----EDVEQ-MEYFYRHISKGAWPFSTRHHGWPISDCTAEGLKASLLLKQFSW------- 395
Dictyostelium           ----EDARD-MKHYHRHYSKGAWPFSTVDHGWPISDCTAEGIKSALALRSLPF------- 388
Polysphondylium         ----DNAPD---GYFRHISKGAWPFSTVDHGWPISDCTAEGIKAALALRSLPN------- 389
Acytostelium            ----DNSPN-MNHYFRHISKGAWPFSTVDHGWPISDCTAEGVKAALSLRSLPF-H----- 389
SHC-Candidatus          ------KGD-WSIKRPDTEPSGWAFEFANEFYPDIDDTAMVLLALMHANGSNP-E----- 322
SHC-Chlorogloeopsis     ------YGD-WAIKNRQGKPGAWAFEFDNRFYPDVDDTAVVVMALNAVHLPNE-K----- 377
SHC-Streptomyces        ------PGD-WSVRRPELPSGGWAFEFHNDNYPDIDDTAEVVLALKRVRHPQP------- 374
```

```
SHC-Hyphomicrobium       ------VGD-WAATRPGVRPGGWAFQYANPYYPDVDDTAVVVMAMDRAAGRDA-S----- 377
SHC-Leptospirillum       ------RGD-WAIRAPDCEPGGWAFQFENDYYPDVDDTAMVLMGMAKILPARP-D----- 344
Naegleria                ----EDVPN-RMEYYRHQSKGGWPFSTRDHGWPISDCTAEGLKAVLTLYDFDE------- 419
Symbiodinium             --------G-DWKHWRQAIQGGWGFSTAEQAWPVSDTTAEAFKAVLLLRKQSC-MKG--- 362
Prorocentrum             --------G-DWRHWL-------GFSTAEQAWPVSDTTAEAFKAVLALRKDGC-TKG--- 316
Gymnodinium              --------G-DWRHWRQPIRGGWGFSTAEQAWPVSDTTAEAFLAVLALRRKDC-IAS--- 270
Alexandrium              --------G-DWRHWRQPIRGGWGFSTAEQAWPVSDTTAEAFKAVLCLRGEQC-TAD--- 412
Dinophysis               --------G------ARPSEEAGGFSTAEQAWPVSDTTAEAFKAVLALRGDAC-VAS--- 411
Scrippsiella             --------G-DWRHWRQPIRGGWGFSTAEQAWPVSDTTAEAFKAVLALRGDVC-LGS--- 414
Aureococcus              AFAFEAPKL-RERYFRHVSKGGWPFSTSAHGWPISDCTAEGLKSVLALRSLAC-V----- 420
Phaeodactylum            AFKYEAALY-RRKFYRHISEGGWPFSTSAHGWPISDCTGEGLKGVLCMLKAKS-VREGLE 420
Helicotheca              AFEGESAIN-RSKYYRHVSLGGWPFSTSAHGWPISDCTGEGLKGVLSLTHSKI-VSESIK 427
Yeast-erg7               --------C-VPGSYRDKRKGAWGFSTKTQGYTVADCTAEAIKAIIMVKNSPV-FSE--- 476
Candida                  --------C-VDGSFRDRRKGAWPFSTKEQGYTVSDCTAEAMKAIIMVRNHAS-FAD--- 401
Verrucomicrobia          ------LPD-PRRYHRLPRKGGWPFSERKNGWSIADCTAESMLALIAAKPFL-------- 360
Eudoraea                 ------LKN-PFKYHRLPRAGGWTFSFQDNGWVVSDCTAEAIKALLETEGLG-------- 381
Plesiocystis             ----TTAPG-ALPNYRSPTRGGWGFADERHPWPVSDCTAEALEALLHVEARGW-IGEG-- 452
Enhygromyxa              ------ILG-GREHYRESARGGWGFANEHHPWPVSDCTAEALEALLHAEHRGW-DHA--- 409
Bodo                     ------PPA-AHAFYRARTKGAWNFSTRAQSWQVSDCTAEGLRVALLLRNKLS--VLR-- 402
Trypanosoma              ------PKS-AAHFYRHRTKGAWNFSTASQSWQVSDCTAEGLRVVLLLRHN--------- 412
Phytomonas               ------PIQ-KDYFYRHRTKGAWNFSTKDQSWQVSDCTAEGLRAVLLLRHM--------- 345
Leishmania               ------PMA-APYFYRHRTKGAWNFSTRSQGWQVSDCTAEGLRALLLLPQY--------- 443
Leptomonas               ------PMA-AAAFYRHRTKGAWNFSTSAQGWQVSDCTAEGLRVMLLLPQY--------- 444
Labilithrix              ------VPR-RKRFYRDASRGGWPFSNRAHGWPITDCTAEGLKCALALEGRF-------- 383
Stigmatella              ------TRE-PQRFFRHPSKGGWPFSTRDHGWPISDCTAEGLKASLVLEPLG-------- 354
Myxococcus               ------SPH-PERFYRHPSRGAWPFSTREHGWPISDCTGEAVKACLLLEPLG-------- 380
Corallococcus            ------SPD-AARHHRHPSRGGWPFSTRAHGWPISDCTAEALKACLLLEPLG-------- 390
S-cinnamoneus            ------PGL-VPP--VRTVVGGWALSDRDSRWPVGDCTAEAVNALLAAEDAQ-------- 399
S-caatingaensis          ------TGL-VPP--LRTVLGGWALSGGDCPWPAADCTAEALNALLHPEGSA-------- 371
Sandaracinus             ------TGR-ERHHDRIDPTGGYCFAGVWHGWPVSDCTAEAMLARLESPEGS-------- 392
S-alboviridis            ------IPT-PHLTARSPARGGWCFSEGGHSWPVSDCTAEAVSALLSGSDAGR-A----- 382
G-obscuriglobus          ------VAG-RDPSFPDTARGGWCLGDGGHAWPVSDCTAEALSAVLSAHTH----GMA-- 401
G-sp.SH-PL17             ------VAG-RDPMFPDRALGGWCLGDSAHSWPVSDCTAEALSAVLGMHAAPE-LELD-- 378
G-sp-IIL30               ------VAG-RDPMFPDRALGGWCLGDSAHSWPVSDCTAEALSAVLGMHAAPE-LELD-- 397
Methylococcus            ------LAD-YREAWRDPALGGWCFSDGRHCWPVSDCAAEAMSALFALYERGD-VRIS-- 396
Methylocaldum            ------LPD-YEAAWRDPALGGWCFSDGLHRWPVSDCTAEALIALLALYEHPL-YTVA-- 398


Helobdella               MVEKSVNTDNDLDTGDVALLTLQND-------DGGFATYERKRGG-TFLESLNPSEVFG- 451
ScLDS                    ISK--RVSKRRLRDAVDMLLTMVNP-------NGGFSSYENLRGG-KILELLNPSEVFG- 445
AjLDS-b                  ITE--RVSKRRLRDAVDMLLTMVNP-------NGGFSSYENLRGG-TILELLNPSEVFG- 522
AjLDS-a                  ITE--RVSKRRLRDAVDMLLTMVNP-------NGGFSSYENLRGG-TILELLNPSEVFG- 522
PpLDS                    ITE--RVSKRRLRDAVDMLLTIVNP-------NGGFSSYENLRGG-TILELLNPSEVFG- 522
ScPS                     IKSSARISRERIHDAVDLLLAMANP-------NGGFATYEILRGSDKVLELLNPSEVFG- 448
PpPS                     IGSEAKISRERLHEAVDLLLDMVNP-------NGGYSSYETLRGGEKLLELLNPSEVFG- 517
AjPS-b                   IGSEAKISRERLHEAVDLLLDMVNP-------NGGYSSYETLRGGEKLLELLNPSEVFG- 517
AjPS-a                   IGSEAKISRERLHEAVDLLLDMVNP-------NGGYSSYETLRGGEKLLELLNPSEVFG- 517
Capsaspora               ISS--PFEEERYHDAIDVLLSMQNS-------DGGYATYETKRGP-EWLELFNPSEVFG- 445
Saccoglossus-LSS         IKN--PIEKEKLYEAVNVLLSLRNS-------DKGFATYETKRGG-KLLELLNPSEVFG- 530
EpLSS                    VTD--LIGKERHCLAVDVLIDMRNP-------DGGYATYETTRGG-YILEKLNPSEVFG- 520
SpLSS                    ISD--HIGKERHCQAIDVLIDMRNP-------DGGFATYETTRGG-YILENLNPSEVFG- 518
SgLSS                    ------------------------------------------------------------ 455
LspLSS                   LKD--HITQERHQEAVDVMLNMRNS-------DGGFATYETMRGG-IILEKLNPSEVFG- 520
MgLSS                    LKD--HITQERHQEAVDVMLNMRNS-------DGGFATYETMRGG-VILEKLNPSEVFG- 298
AaLSS                    LKD--HISQERHQEAVDVMLNMRNS-------DGGFATYETMRGG-VILEKLNPSEVFG- 179
AfLSS                    LKD--HITQERHQEAVDIMLNMRNS-------DGGFATYETMRGG-VILEKLNPSEVFG- 520
ArLSS                    LKD--HITQERHQEAVDVMLNMRNS-------DGGFATYETMRGG-VILEKLNPSEVFG- 520
HspLSS                   VKE--PISKDRHCQAVDVMLNMRNP-------DGGFATYETMRGG-MILEQMNPSEVFG- 520
EsLSS                    IKE--PISNDRHCQAVDVMLNMRNN-------DGGFATYETTRGG-MLLEQLNPSEVFG- 520
ApLSS                    VRD--HIEKERHCQAVDVILNMRNK-------DGGFATYETMRGG-VILEKLNPSEVFG- 520
PpLSS                    ------------------------------------------------------------ 429
PmLSS                    VKD--HIEKDRHYEAVDVILNMRNK-------DGGFATYETMRGG-IILEKLNPSEVFG- 520
Amphimedon               ISN--GVTVSKMEDTINVLLNMQNS-------NGGFSSYETNRGG-AILELLNPSEVFG- 444
Aplysia                  ITE--AVPKQRIYRGIDVLLEMRCD-------DNGWATYEDKRGG-VLLEVLNASEVFG- 445
Capitella                LSQ--EIPEEQLNQAIDVLLSMSNLLYGYSWVGMGYATYETKRGG-KMLEMLNPSEVFG- 453
Branchiostoma-LSS        VQD--PAPDHRLFDAVDVLLNMRNS-------DGGFATYETKRGG-KLLELLNPSEVFG- 448
Lingula                  LTQ--TVSEERLYQAVDVLLSMKNA-------DGGFATYETTRGG-KLLELLNPSEVFG- 446
Callorhinchus            ISD--HVIDQQLHQAVHVLLTMRNT-------DGGFSTYEXKTGG-RLLELLNPSEVFG- 445
HumanLSS                 VTE--HIPRERLCDAVAVLLNMRNP-------DGGFATYETKRGG-HLLELLNPSEVFG- 522
Pteropus                 VTT--HTSKQQLFDAVAVLLSMRNA-------DGGFATYETKRGG-HLLELLNPSEVFG- 446
DreLSS                   LKE--NIPKERLFDAVNVLLSMRNP-------DGGFATYETKRGG-KLLELLNPSEVFG- 523
Haplochromis             IRQ--PVPSERLCDAVNVLLSMKNT-------DGGFATYETKRGG-RLLELLNPSEVFG- 446
Xenopus                  LTD--LVPPERLRDAVDVLLSMRNS-------DRGFATYETKRGG-LLLELLNPSEVFG- 446
Charadrius               IAK--LVPPERLFDAVNVLLSMRNS-------DGGFATYETKRGG-HLLELLTPSEVFGK 443
Anolis                   IKD--HIPPSRLFDAVNVLLNMQNA-------DGGFSTYETMRGG-WLLELLNPSEVFG- 446
Alligator                ITE--HVPPQRLFDAVNVLLSMRNS-------DGGFATYETRRGG-RLLELLNPSEVFG- 446
Pneumocystis             --FPKKVSYDRLKDSVDVILSLQNK-------DGGFASYELIRGP-SWLESINPAEVFG- 447
Arthrobotrys_            --FPELVSNQRMRDAVDVMLALRNP-------SGGFASYEVIRGI-KQLEWFNAAEVFG- 448
Aspergillus              --YPRLISVERLKDSVDCLLLMQNP-------SGGFTEYETTRGS-EKLEWLNAAEVFG- 448
Spizellomyces            --TTKNVGQERFRDAVDVLLRMQNT-------DGGFASYELTRGP-KILEWINPAEVFG- 442
Trichosporon             --TDKPVTIDRMRDAVDVLLSMQNP-------SGGFASYELMRGS-AKLEALNAAEVFG- 433
Puccinia                 --TPKLVSKDRLCLAVDVILSLQNP-------NGGFASYELVRGP-SWLEYLSPAEVFG- 453
Mixia                    --APKPVSYKRLCDAVDVLLSLQNS-------DGGFGSYELVRGP-KWLELANCAEVFG- 449
Microbotryum             --VKERVSKRRLCDAIDTILTMQNA-------DGGFASYELIRGP-HWLEWLNPAEVFG- 449
Dacryopinax              --TPKLVSERRMCDAIDVILSLQNP-------DGGCASYEPIRGP-RWLEWINPAEVFG- 447
Fistulina                --TPKLVSEERMRMSVDLLLTMQNP-------GGGFASYEPIRAP-HWLELLNPAEVFG- 447
Wallemia                 --TKHHVSYERLHDSVDLLLGMQNA-------DGGFASYELTRGS-KHLELLNPAEVFG- 446
Ceraceosorus             --LPQRIAIERLHDSVDLLLTMQNR-------SGGFASYETINGP-AVLEWLNPAEVFG- 446
```

```
Ustilago                  --LGKQVSRERMHDSIDLLLTMQNS-------GGGFASYERINGP-AMLELINPAEVFG- 447
Malassezia_               --LGRPVSQRRLRDTVDLLLSMQNP-------GGGYASYETINGP-SFTEWLNPAEVFG- 448
Fluviicola                --KKNHVHIERLKPAVDWLLKMQNK-------DGGWASYEKQRAP-KWIEVLNPAMLFE- 444
Methylobacter             ---HPAIDVMRIKKAVDIILSYQNS-------DGGWATYELTRAP-KWLEKLNPSEVFA- 442
Methylobactes             ---HPAIDVMRIKQAVDIILSYQNS-------DGGWATYELTRAP-KWLEKLNPSEVFA- 442
Methylosarcina            ---RPTIDATRIKKAVDVILSYQND-------DGGWATYELTRAP-KWLEKLNPSEVFA- 442
Aphanomyces               ---LPAFPDDRLRDAVDFILALQNS-------DGGFPPYERSRGF-DWYEHLNPAVVFG- 442
Cystobacter               --VDSPLSQERLTDAVDLLLSMQNE-------DGGWATYELTRGP-KWLELLNPSDCFS- 437
Galdieria                 --KEQFFSHERLFDAVDVSLSLQNK-------DGGWATYENTRSY-SWLEWINPSEVFG- 447
Selaginella               --VGKPLSPERLYDCVNMLISYQNA-------NGGVATYELTRSY-AWIELFNPSETFG- 450
Panax                     --VGEAISPVHLYDAVNWILSLQNC-------TGGFASYELTRSY-AWLELLNPAETFG- 460
Physcomitrella            --VGNPIAAERLYDAVNVILSYQNE-------DGGSATYERTRSY-PWLEVINPAETFG- 452
Cucumis                   --VGEPLEKNRLCDAVNVLLSLQNE-------NGGFASYELTRSY-PWLELINPAETFG- 453
Adiantum                  --VGESLQAERFYDAVNTMLSYQNG-------NGGVATYELTRSY-PWLELINPAETFG- 453
Abies                     --VGKPIPSQRIFDCVNLMLSMQNS-------DGGFATYELTRSY-PWLEKINPAETFG- 454
Malus                     --VGESLDIKRFYDAVNVTLSLQND-------DGGFATYELTRSY-QWLELINPAETFG- 451
Amborella                 --VGEPIPANRFYDAVNVIISMQNS-------GGGFATYELTRSY-AWLELINPAETFG- 453
Nicotiana                 --VGEPLEAKRLYDAVNVMLSLLNP-------DGGIATYELSRSY-PWLEIVNPAETFG- 451
AtCAS1                    --VGEPIDAKRLYEAVNVIISLQNA-------DGGLATYELTRSY-PWLELINPAETFG- 551
Glycine                   --VGEPIDVKRLYDSVNVVLSLQNE-------DGGFATYELKRSY-NWLEIINPAETFG- 452
Coffea                    --VGETIDVKRLYDSVNVILSLQNS-------DGGFATYELTRSY-AWLETINPAETFG- 452
Populus                   --VGEPLAANRFYDAVNVILSLQNG-------DGGFATYELTRSY-SWLELINPAETFG- 452
Vitis                     --VGEPLDAKQLYDAVNVILSLQNG-------DGGFATYELTRSY-AWLELINPAETFG- 452
Micromonas                --AGPSVPVGLLQECVNVILSYQNM-------GGGWATYENTRSY-EWVEIINPAETFG- 452
Volvox                    --VGEPIPAERLYDCVNVILSYQNS-------DGGMATYENTRSF-HWLEILNPAETFG- 479
Chlorella                 --VGPPIPEPRLCDCVNVVLSYQNG-------DGGWATYENKRSF-EMLEIINPSETFG- 452
Synchroma                 -DHVPLLSEERICDAVNVILTLHNA-------DGGWATYENNRGF-GWYELLNPSETFG- 455
Chattonella               -DSGKVLSDERMFQAANVLLSYQNH-------DGGWATYENNRGY-GWYEYLNPSDVFG- 448
Ochromonas                VPADKLLTPERFYDACNVILSYQNE-------DGGWATYENNRGY-GWYELLNPSEVFG- 458
Chromulina                --TSDRISSNRLQDACDVILSLHNK-------DGGWATYENNRGY-GWYEMLNPSEVFG- 457
Chondrus                  IPKKKMISNDRLQDAVKMILSYQNP-------DGGWATYELQRGP-AWLEFLNPSEVFG- 449
Acanthamoeba              ---VTPFEDQRYFDAVNVILSLQNS-------DGGWATYELQRGP-SILEYINPAEVFD- 443
Dictyostelium             ---IEPISLDRIADGINVLLTLQNG-------DGGWASYENTRGP-KWLEKFNPSEVFQ- 436
Polysphondylium           ---IVPISLDRVAEGVNVILSLQNS-------DGGWASYENKRGP-NWLELFNPSEVFQ- 437
Acytostelium              ---IAPISIDRVAEGINVILSLQNK-------DGGWASYENKRGP-NWLEKFNPSEVFQ- 437
SHC-Candidatus            ---AQ---AAAERRAVNWLLAMQSS-------DGGWAAFDVDNNW-AMLNQVPFADH--- 365
SHC-Chlorogloeopsis       ---LK---HRAIARAVNWIVSMQCR-------AGGWAAFDLNNDQ-DWLNLIPYGDL--- 420
SHC-Streptomyces          -----RRVEAAVERAMRWTEGMQSR-------NGAWGAFDVDNTS-PFPNRLPFCDF--- 418
SHC-Hyphomicrobium        ---KREQYRESMARAREWIAGVQSK-------NGGWGAFDADNTY-EYLNQIPFSDH--- 423
SHC-Leptospirillum        ---LAARMEGVFRRATLWVMAMQGT-------DGGWGAFDRDNDL-LFLNHIPFADH--- 390
Naegleria                 ----LILSEERLTDAVRVILSMFNG-----GTNGGWATYELSRTH-SWIEIINPAALYG- 468
Symbiodinium              --ESGQMPDQHLFDTVRFLLSYQNG-------DGGWATYENNRGW-SWYELMNPSEVFG- 411
Prorocentrum              ---SNDMPDQHYFDTVQFLLSYQNA-------DGGWATYENCRGW-KWYEALNPSEVFG- 364
Gymnodinium               ---CCPMSDEQCFDAVQFLLSYQND-------DGGWATYENRRGW-KWYECLNPSEVFG- 318
Alexandrium               ---APALSGEQLFDSVQFLLSYQNG-------DGGWATYENCRGW-KWYELLNPSEVFG- 460
Dinophysis                ---CPEFPDEHCFDTVQFLLSYQNA-------DGGWATYENNRGW-AWYELLNPACVFG- 459
Scrippsiella              VGEAPPFPDEHCFDSVEFLLSYQNA-------DGGWATYENCRGW-AWYELLNPACVFG- 465
Aureococcus               -GECAPIGYERLCDAADVVLALQNA-------DGGYATYENTRGY-GWYEWLNPSEVFG- 470
Phaeodactylum             DGSLREISEVRLQKAANILLSYQNE-------DGGFPTYENNRGF-GFYESLNPSEVFG- 471
Helicotheca               EGRLKPISRSRLEDAVNVLLMYQNE-------DGGWATYENNRGF-GWYEQLNPSEVFG- 478
Yeast-erg7                --VHHMISSERLFEGIDVLLNLQNI---GSFEYGSFATYEKIKAP-LAMETLNPAEVFG- 529
Candida                   --IRDEIKDENLFDAVEVLLQIQNV---GEWEYGSFSTYEGIKAP-LLLEKLNPAEVFN- 454
Verrucomicrobia           ---AQPPSPKILEDGLRFILSYQNR-------DGGWGSCDRVVGP-LWIEKFNASHVFA- 408
Eudoraea                  ---DEPLSDERINKAISFIMLLQGK-------DGGWTSVDKAIGS-PKLEWFNAANVFV- 429
Plesiocystis              -QATPALSPARKLAAAEFILLRQND-------DGGFGSYEERRGS-MALIHFNPAEMYG- 502
Enhygromyxa               ---AARLDLSDKLAAVEFVLLRQND-------DGGFGSYEPRRGS-MLLARFNPAEMYG- 457
Bodo                      --HTDTMDDSRLFDGVDEILTLRWA-----AGDGGWGSYEAPRGP-RYLELLNCSEIYK- 453
Trypanosoma               -----PFSVSRIRDAVDEILSLRNA-------KGGWASYEPTRAP-LYVELFNSSDVFR- 458
Phytomonas                -----PFPTARIFDAVDEILSLRNS-----SGDGGWASYEPSRAP-HYCELLNCSEMFK- 393
Leishmania                -----AFPMRRIFDGVDEVLSLRNS---GLGGDGGWASYEPSRAP-AYCELLDCSELFK- 493
Leptomonas                -----DFPVRRICDGVDEILSLRNS---GCGGDGGWATYEPTRGP-SYCELLDCAELFK- 494
Labilithrix               ---APRIPEPLLRDAVMLILDWQND-------DGGWATYEKRRGG-AWLEKLNPSQVFG- 431
Stigmatella               ---LNRVPQARLQDAVQLILSMQNE-------DGGWATYELQRGP-KVLELLNPSDVFS- 402
Myxococcus                ---LNRVPRERLAQAVDFILSLQNR-------DGGWATYEPTRAP-PWLERLNASDVFA- 428
Corallococcus             ---LNRVPRERLEQAVAFILSLQNR-------DGGWATYEQQRGP-RWLERFNPSDVFA- 438
S-cinnamoneus             -----HLRTWSLPAALDIILDRQNR-------DGGFGTLDRQRAG-RWLEALNPTEMFA- 445
S-caatingaensis           ---PPPLPPHALRAALEVMLDRQNR-------DGGFGILDRQRAG-RWLEALDPAGMFP- 419
Sandaracinus              ------PTREAMEAAARFVLRCQNT-------DGGFGSYEARRTD-VSLEWINPAEMFG- 437
S-alboviridis             ------DPTFDISAATEFLLARQNR-------DGGFGTYEARRAS-RLMEHLNPAEMFT- 427
G-obscuriglobus           --PEERIPDARLIQAAEFMLTRQNR-------DGGFGSYERARSP-RWLERMNPSEMFT- 450
G-sp.SH-PL17              --SRERISDTRLALAADFILSRQNP-------DGGFGSYERARSP-RWLERLNPSEMFT- 427
G-sp-IIL30                --SRERISDTRLALAADFILSRQNP-------DGGFGSYERARSP-RWLERLNPSEMFT- 446
Methylococcus             ----EALGADRLRLGVEFILSRQNA-------DGGFGTYERRRGG-RLLELVNPSEMFG- 443
Methylocaldum             ----SPIEPERLRQAVTFILSRQNA-------DGGFGTYERRRGG-KLLETINPSEMFG- 445


Helobdella                ---DVMLDYSYVECTSAAMQAFHFFTEYIDRNY-------KKEIIKQALEKCLEFIVNS- 500
ScLDS                     ---DIMVDYTYTECTSSVLQALRQFVDSD-PDY-------RHDEIWTVLKNSLEYIRSN- 493
AjLDS-b                   ---DIMVDYTYTECTSSVLQALRHFVDSD-PDY-------RQDEIWAVLRNAMEYIRSN- 570
AjLDS-a                   ---DIMVDYTYTECTSSVLQALRHFVDSD-PDY-------RQDEIWAVLRNAMEYIRSN- 570
PpLDS                     ---DIMVDYTYTECTSSVLQALRHFVDSD-PDY-------RQDEIWAVLRNAMEYIRSN- 570
ScPS                      ---DIMVDYTYTECTSSVMQALRHFVNYD-PSY-------RQGEIWDVLRNGLQYIKQN- 496
PpPS                      ---DIMVDYTYTECTSSVLQALRHFVDYD-PNY-------RTDEIWNVLKNGLGYIKQN- 565
AjPS-b                    ---DIMVDYTYTECTSSVLQALRHFVDYD-PKY-------RTDEIWNVLKNGLGYIKQN- 565
AjPS-a                    ---DIMVDYTYTECTSSVLQALRHFVDYD-PKY-------RTDEIWNVLKNGLGYIKQN- 565
Capsaspora                ---AIMIDYTYVELTSAVVQALASLTVQF-PKY-------RTAEISATMKRAVHFIRSI- 493
Saccoglossus-LSS          ---DIMIDYTYVECTSAVMQSLKHFQDQY-PEY-------RKDEIRTTLDDGLAYIKDK- 578
EpLSS                     ---DIMIDYTYVECTAAAMLALKHFQDQH-PEY-------RRDEINSVLDTGLDFIKGI- 568
```

```
SpLSS                     ---DIMIDYTYVECTVAVLLALHHFHTQY-PEY-------RTDEIRSVMDQALDYIKGK- 566
SgLSS                     ------------------------------------------------------------ 455
LspLSS                    ---DIMIDYTYVELTSAVMQSLKKFSDIY-PEY-------RQAEIRATLDRGLLYIALK- 568
MgLSS                     ---DIMIDYTYVELTSAVMQSLKKFSDIY-PDY-------RQAEIRATLDRGLLYIARK- 346
AaLSS                     ---DIMIDYTYVELTSAVMQSLKKFSDIY-PDY-------RQAEIRATLDRGLLYIADK- 227
AfLSS                     ---DIMIDYTYVELTSAVLQSLKKFSDIY-PDY-------RQAEIRATLDRGLLYIADK- 568
ArLSS                     ---DIMIDYTYVELTSAVLQSLKKFSDIY-PDY-------RQAEIRATLDRGLLYIADK- 568
HspLSS                    ---DIMIDYTYVELTSAVMQSLKKFSELY-PEY-------RKDDVRSTLDNGLSYIAKK- 568
EsLSS                     ---DIMIDYTYVELTSAVMQSLKKFSDLY-PDY-------RKDDIRSTLDNGLSYIAKK- 568
ApLSS                     ---DIMIDYTYVELTSAVMQTLKKFNTLY-PDY-------RKEDIRSTLDKGLKYIAMK- 568
PpLSS                     ------------------------------------------------------------ 429
PmLSS                     ---DIMIDYTYVELTSAVMQCLKKFSELY-PEY-------RKDE---------------- 553
Amphimedon                ---DIMVDYTYVECTSASLQAINHFNKRY-PQH-------RPKEIQECLSRGLEYILNI- 492
Aplysia                   ---DIMIDYTYVECTSACMQCMTTFTKAH-PEY-------RKDEIQAALTGGLDYVRGK- 493
Capitella                 ---DIMIDYPYVELTSSVIQALKKFSNKH--AY-------RNMEILWTVHLGLQYIKRE- 500
Branchiostoma-LSS         ---DIMIDYTYVECTSAVMQALKHFQDQY-PDH-------RAEEIR-------------- 483
Lingula                   ---DIMIDYTYVECTSASMQALEHFTSQY-PAY-------RTEEIRQTLESGLQYVKSK- 494
Callorhinchus             ---DIMIDYTYVECTSAVIQALRHFQQKH-PHH-------RAFEIRDTLNKGLEFCRGC- 493
HumanLSS                  ---DIMIDYTYVECTSAVMQALKYFHKRF-PEH-------RAAEIRETLTQGLEFCRRQ- 570
Pteropus                  ---DIMIDYTYVECTSSVMQALTVFHTQF-PEH-------RPGEIRETLEQGLEYCRRK- 494
DreLSS                    ---DIMIDYTYVECTSAVLQALKHFHSVY-PEH-------RAEEIRSTLQRGLDYCRRV- 571
Haplochromis              ---DIMIDYTYVECTSAVMQALRHFQKVY-PDH-------RAEEIRSTLREGLEYCRKV- 494
Xenopus                   ---DIMIDYTYVECTSAVMQALKHFQARD-PNY-------RAQEIRETLQKGLDYCCSV- 494
Charadrius                GSCDIMIDYTYVECTSAVMQALRHFHDQF-PEH-------RAPEIRETLQKGLDFCRKK- 494
Anolis                    ---NIMVDYTYVECTSAVMQALKHFHERF-PEH-------RALEIREVLQKGLQYCRRL- 494
Alligator                 ---DIMIDYTYVECTSAVMQALKHFHEEF-PEH-------RAQEIRETLRKGLEYCRRM- 494
Pneumocystis              ---NIMIEHSYTECTTSVVTALCYFRSLC-SHY-------RGSEINKSVKKAIQFIKES- 495
Arthrobotrys_             ---RIMIEYEYPECTTAVVTALVYFQKVD-KVY-------RAEEIKQTIKNAVDWIISA- 496
Aspergillus               ---GIMIGYDYPECTTASVTALSLFSRFY-PDY-------RADEIKAAKDKAVRYIKRV- 496
Spizellomyces             ---DIMVEYSYPECTTAVMLGLLAFQKRD-PAY-------RSAEIQSALNRGLGYIKKS- 490
Trichosporon              ---DIMVDYMYPECTTSALSALTYFKKLD-AQY-------RAADIEKCVEKAIRWIHSV- 481
Puccinia                  ---KTMIEVTYPECTTACLTAMSLFSRCY-PDY-------RAADISRAREGAIKFIHSA- 501
Mixia                     ---NIMVEYTYPECTTACLTALTLFSNEF-PGY-------RSSTIERVSRRAIEYIHGT- 497
Microbotryum              ---NIMTEYSYPECTTACVTALTIFKRKH-PDY-------RADDIDRVSKRAIEYIHKK- 497
Dacryopinax               ---NIMIEYNYPECTTSALTALSIFTNEY-PQY-------RAEDIRKFKSKAIQWLHTS- 495
Fistulina                 ---NIMTEYCYPECTTSVITALSIFRRYY-PDY-------RAEDIDRTIRDAIGYLHRE- 495
Wallemia                  ---KIMVEYSYPECTTSVVTALSLFKKHQ-PDY-------RREDIERTTNAAIKYIHAA- 494
Ceraceosorus              ---DIMIEYDYPECTTSVVTGLLKFQQ-I-SNY-------RKKDIDRCVKSAFGYIMKA- 493
Ustilago                  ---DIMIEYAYPECTTSVVTALLKFTK-I-DDY-------RKQDIKSTVHSAVKYILKA- 494
Malassezia_               ---NIMVEYAYPECTTSVVSGLRMFQQ-Y-DDY-------RSAEIDAAVERAVKYILGA- 495
Fluviicola                ---NIMTEATYTECSSATIQGLKEFT-KE-HDY-------RQEDIKRAIDRGAKFLESK- 491
Methylobacter             ---DIMIDYSWTECTAACVISLLEIQETY-PDF-------KNSEIRKAIGAGLDFILKQ- 490
Methylobactes             ---DIMIDYSWTECTAACVLSLLEIQETY-PDF-------KNSEIRKAIGAGLDFILKQ- 490
Methylosarcina            ---DIMIDYSWTECTAACVISLLEIQETD-PAY-------RSNEIRQAISAGLNFILKQ- 490
Aphanomyces               ---AIMHDYSYVECSSSSLSALQAFHARH-PTY-------RPEAIKRATTKADTFIRSL- 490
Cystobacter               ---DIMIDPSYVECTSSCMQALARFRERL-PGV-------RAKEIDTAMKRGARYVERA- 485
Galdieria                 ---DIMIDYSCVECTSSSIQGLAAFRARH-PGH-------RRAQVDIAIERGARYIESI- 495
Selaginella               ---DITIDYSYVECTSACVQALCAFSRLY-PDH-------RSKEIELFVAKGCQYIESI- 498
Panax                     ---DIVIDYQYVECTSAAIQGLKSFMRLY-PGY-------RRKEIEACIAKATNFIESI- 508
Physcomitrella            ---DIVIDYSYVECTSACMQALASFQKRY-PHH-------RTKEIAKSIQRARKYIESI- 500
Cucumis                   ---DIVIDYSYVECTSATMEALALFKKLH-PGH-------RTKEIDAAIAKAANFLENM- 501
Adiantum                  ---DIVIDYQYVECTSAVIQALAAFKKLY-PKH-------RTEEVNACIQHAAKYIESI- 501
Abies                     ---DIVIDYSYVECTSAITQALVSFKKLY-PEH-------RHKEIETCILKATRYIENI- 502
Malus                     ---DIVIDYPYVECTSAAIQALALFKRLC-PAH-------RSDEIENCIARAAKFIETI- 499
Amborella                 ---DIVIDYPYVECTSAAIQALASFKKLY-PAH-------RTKEIETCISKAVHFIESI- 501
Nicotiana                 ---DIVIDYPYVECTSAVIQALAAFKKLY-PGY-------RKEEVDHCIRKGASYIEKI- 499
AtCAS1                    ---DIVIDYPYVECTSAAIQALISFRKLY-PGH-------RKKEVDECIEKAVKFIESI- 599
Glycine                   ---DIVIDYPYVECTSAAIQALASFRKLY-PGH-------RREEIQRCIDKATTFIEKI- 500
Coffea                    ---DIVIDYPYVECTSAAIQALTAFKKLY-PGH-------RREEVQRCIERAALFIEKI- 500
Populus                   ---DIVIDYPYVECTSAAIQALVSFKKLY-PGH-------RQEEIERCIRKATKFIESI- 500
Vitis                     ---DIVIDYPYVECTSAALQALTSFNKFY-PGH-------RREEIEHCIKKATMFIEKI- 500
Micromonas                ---DIMIDYPYVECSSASMQALAKFHQRY-PTY-------RKQDIKKSLHRGRKFLLSI- 500
Volvox                    ---DIIVDYSYVECTSACITALAAFRKRH-PDH-------RPSEISAALGRAEAFIRSI- 527
Chlorella                 ---EIVVDYNHVECSSACITALTAFAGRY-PAH-------RAHEIGAALRRGIKYLKSI- 500
Synchroma                 ---DIMIDYSYVECTAACMQALAAFRARY-PAH-------RAAEVGSAIAAGARFIKSI- 503
Chattonella               ---DIMIDYSYVECSTACISALSNFQKAY-PEH-------RKDEIKNSIEQGRKFVKSI- 496
Ochromonas                ---DIMIDYSYVECSSACITALKALQREF-PDH-------RKEEVIQSIAKGREFLKSI- 506
Chromulina                ---DIMIDYSYVECTSAAITALVKFSKQY-PKY-------RANEVWNAIQSGRHFIKSI- 505
Chondrus                  ---DIMVDYSYVECTASALKGIGEFRAEF-PDH------PLVPKLNESLAKGIRYIESI- 498
Acanthamoeba              ---AIMVDYPYVECTSACVQALTMFVQHY-PRH-------RATEITTAVKKAVDLIKSK- 491
Dictyostelium             ---NIMIDYSYVECSAACIQAMSAFRKHA-PNH------PRIKEINRSIARGVKFIKSI- 485
Polysphondylium           ---NIMIDYSYVECSAACIQAMSSFLKHA-PEH------PRAREIRRSIDRGIKFIKSI- 486
Acytostelium              ---NIMIDYSYVECSAACIQAMCAFRSQA-PNH------PRIKEVNGSIERGVRFIKSI- 486
SHC-Candidatus            ---NAMLDPTCPDITGRVLECLCRRGM---AGH-------------DAARRGVAYLLQA- 405
SHC-Chlorogloeopsis       ---KAMIDPNTADVTARVLEMVGSCNLSM-DAS-------------N-LQRAINYLIAE- 461
SHC-Streptomyces          ---GEVIDPPSADVTAHVVEALAVLGRQH-G---------------ERARRGIAYLLAE- 458
SHC-Hyphomicrobium        ---GALLDPPTADVSARCVSMLAQLGERR-DTS-------------AVLDKALAYLENT- 465
SHC-Leptospirillum        ---GALLDPSTADLTGRVLELLGALGYGP-DFP-------------P-AARAIRYLRRE- 431
Naegleria                 ---EIMIDYPHTECTSACITALLQFKKHF-PQS------PYVPMIDDSIKHAIKVIEGK- 517
Symbiodinium              ---AIMIDYSYVECSSSAMQALMLFTEQF-PQH------RAG-EIARAVQRGARFIEAM- 459
Prorocentrum              ---EIMIDYSYVECSASSMSALAEFSKQF-PDH------RRQ-EIQRAIRRGRDFIKAI- 412
Gymnodinium               ---DIMIDYSYVECSSSAMGALSVFRQHF-PHH------RRG-EIDRAIRRGGRFIKQM- 366
Alexandrium               ---DIMIDYSYVECSASSMGALSLFAKQF-PDH------RKA-EIARAIRRGAQFIKSM- 508
Dinophysis                ---NIMIDYSYVECSASAMQALMDFHGQF-PGH------RAR-EIKRAVARGATFIEAM- 507
Scrippsiella              ---NIMIDYSYVECSSSAMQALVDFRRQF-PEH------RAA-EIIVSVERGARFIEAM- 513
Aureococcus               ---DIMIDYSYVECSMA----LARFREAC-PDH------RAA-EISAALKRGNAFLRSI- 514
Phaeodactylum             ---DIMIDYSYVECSMASLTALADFHEDY-PDH------RTE-EIVHAIEKGRDFLKDL- 519
Helicotheca               ---DIMIDYSYVECSMASLTALVEFHEMY-PDH------RAM-DISAAVGRGKDFMKSI- 526
```

```
Yeast-erg7               ---NIMVEYPYVECTDSSVLGLTYFHKYF-D-Y------RKE-EIRTRIRIAIEFIKKS- 576
Candida                  ---NIMVEYPYVECTDSSVLGLTYFAKYY-PDY------KPE-LIQKTISSAIQYILDSQ 503
Verrucomicrobia          ---DIMVDHSFAECTGSVLSALALVRKEY-PHL------ETK-RVDHAIREGVRYLTDT- 456
Eudoraea                 ---DIMVDHSYVECTASIIQCFAQIKRSR-PHL------FTS-EMESAMERAVKYLLKS- 477
Plesiocystis             ---NCMLEYSYAECSASCVRALAVLRERE-PALLASAGDLRA-RVDAAVDAGVRFLLEAV 557
Enhygromyxa              ---NCMLEYSYTECTASCVRGLAVALEAL-GAN--MPSELRA-RVQAGVDAGVQFLLGS- 509
Bodo                     ---DIMVDYTYSECTSSCVHTLCLFRKQF-PQY------RIE-EVNRAIDEGSRCVVSK- 501
Trypanosoma              ---DVMTDYGYAECSSSCIHTLALFREHY-PGY------RRA-EINAAIREGLKFVLSL- 506
Phytomonas               ---DVMVEYSYAECSSSSIHTLSLFREQF-PYY------RRS-SIDTAISEGIAYIFST- 441
Leishmania               ---DVMIDYSYVECSSSCIHTLSLFRERY-PHY------RRR-DVDRAISEGIAYVLGQ- 541
Leptomonas               ---DVMIDYSYAECSSSCIHTLSLFREQY-PHY------RRS-EVDRAISEGIACVLGK- 542
Labilithrix              ---DIMVDYSYVECTSACMQALRASLPRF-GRR------LAA-RARRAIAKGERFLRNA- 479
Stigmatella              ---TIMVDVSYVECTSACVQALAAWRKHH--PV------PDA-RVDRAISRGVEFIRRT- 449
Myxococcus               ---NIMVDISYVECTSSCVQALVAWKKVQ----------PEA-PVEDAITRGLDYLRRT- 473
Corallococcus            ---GIMVDPSYVECTSACIQALAAWRGAW----------PHA-PVGQSIARGADFLRRQ- 483
S-cinnamoneus            ---NCMVDSSSVDCTGSALTALARIRPLL-SPD------GRR-RADAAIKRGAAFLRSA- 493
S-caatingaensis          ---GRMTDTSCADCTGSVLTALGRLRRHL-GPG------DRR-RAEAATGRAVAYLRAA- 467
Sandaracinus             ---DSMTEKSYVECTASCVTALAAFVHRW-PQS-----ELAH-ECETAIARAVASLTRT- 486
S-alboviridis            ---HCMVEGSYTECTGSAAVALVHLQGRV-NGT------QRH-ACKRALSRARTFLLST- 475
G-obscuriglobus          ---RCMTDQSYIECTGSCLVALGRFRKAI-PHH------AAG-RITRATNRGARFLLSR- 498
G-sp.SH-PL17             ---RCMTDQSYIECTGSCLVALSRFRVAV-PHH------ATN-RIDRAIRRGARFLLGR- 475
G-sp-IIL30               ---RCMTDQSYIECTGSCLVALSRFRVAV-PHH------ATN-RIDRAIRRGARFLLGR- 494
Methylococcus            ---QCMTELSYVECTASSLGALAHYLRNY-PDL------PGG-KITAAIRKAERFLRSR- 491
Methylocaldum            ---QCMTELSYIECTGSALAALGHYRKHY-PDF------SGG-AIERAIRNAVAFLRRR- 493


Helobdella               QQVDGSYYGSWAVCYTYGTWFALEGLSCVGYHYVR-------APHPSLTKACDWLASKQQ 553
ScLDS                    QLPDGSFEGSWGVCFTYGTWFALEAFACMGKSFKY------NTASIEVKKS--LLIPCFQ 545
AjLDS-b                  QLPDGSFEGSWGVCFTYGTWFALEAFACMGKNYQD------NTASIDVKKACSFLVSRQM 624
AjLDS-a                  QLPDGSFEGSWGVCFTYGTWFALEAFACMGKNYQD------NTASIDVKKACAFLVSRQM 624
PpLDS                    QLPDGSFEGSWGVCFTYGTWFALEAFACMGKNYQD------NTASIDVKKACSFLVSRQM 624
ScPS                     QLPDGSFEGSWGVCFTYGTWFALEAFACMGQNYDD------NTATLEVKKACSFLVSRQM 550
PpPS                     QLPDGSFEGSWGVCFTYGTWFALEAFACMGQNYND------NTASMQVKKACSFLVSRQM 619
AjPS-b                   QLPDGSFEGSWGVCFTYGTWFALEAFACMGQNYND------NTASMQVKKACSFLVSRQM 619
AjPS-a                   QLPDGSFEGSWGVCFTYGTWFALEAFACMGQNYND------NTASMQVKKACAFLVSRQM 619
Capsaspora               QRKDGSWEGCWAVCFTYGTWFGIEALATAGEGYYK------GTASPSMRSACDFLVSKQR 547
Saccoglossus-LSS         QRSDGSWEGSWGVCFTYGAWFGLEAYSCMGCRYDL------GTATNEVKLACEFLCKKQM 632
EpLSS                    QRPDGSWEGSWGVCFTYGAWFGLEGLGCMGMRYDR------DTATPEVKKACAFLVSKQM 622
SpLSS                    QQPDGSWEGSWGVCFTYAAWFGLEGFGSMGLRYDK------DLASSEVKKACQFLVSKQM 620
SgLSS                    ------------------------------------------------------------ 455
LspLSS                   QRRDGSWEGSWGVCFTYAAWFGLEAYACMGYSYET------STASSEVKRACAFLLSRQM 622
MgLSS                    QRTDGSWEGSWGVCFTYAAWFGLEAYACMGYSYET------STASSEVKRACAFLLSRQM 400
AaLSS                    QKRDGSWEGSWGVCFTYAAWFGLEAYACMGYSYET------STASSEVKRACAFLLSRQM 281
AfLSS                    QKRDGSWEGSWGVCFTYAAWFGLEAYACMGYSYKT------STASSEVKRACAFLLSRQM 622
ArLSS                    QKRDGSWEGSWGVCFTYAAWFGLEAYACMGYSYKT------STASSEVKRACAFLLSRQM 622
HspLSS                   QKTDGSWEGSWGVCFTYATWFGLEAYACMGHTYET------TTATPEIKRACAFLLSRQM 622
EsLSS                    QKADGSWEGSWGVCFTYATWFGLEAYACMGHSYET------MTATPEVKRACAFVLSRQM 622
ApLSS                    QRIDGSWEGSWGVCFTYAVWFGLEAFACMGYSYET------GAVTMQIRRACTFLKSKQM 622
PpLSS                    ------------------------------------------------------------ 429
PmLSS                    ----------------------------MGYSYEK------RTNTMQIRRACDFLVSKQM 579
Amphimedon               QRPDGSWEGSWGVCFTYGTWFGLEALASMNRRYDY------GTAGSEVKRACQFLVDHQM 546
Aplysia                  QRPDGSWEGSWAVCFTYGAWFALEAYACMGHVYGGGEGQDSGKVPEEVEKGCQYLLSIQM 553
Capitella                QRKDGSWYGSWGVCFTYGAWFALEAFAAMSFRYDY------NNVTEEMKKGCQWLVDHRL 554
Branchiostoma-LSS        ---------SWGVCFTYGTWFGLEAMACMGHRYDI------GTATEAVTRACNFLKSHQM 528
Lingula                  QRNDGSWEGSWGVCFTYGTWFGLEAFAAMGYRYDT------ENVPPEVRKACNFLISKQM 548
Callorhinchus            QRPDGSWEGSWGVCFTYGTWFGMEAFACMGHTYQR------GAACREITRACEFLLSKQM 547
HumanLSS                 QRADGSWEGSWGVCFTYGTWFGLEAFACMGQTYRD------GTACAEVSRACDFLLSRQM 624
Pteropus                 QRADGSWEGSWGVCFTYGTWFGLEAFACMGQTYHD------GTAGMDVSRACNFLLSRQM 548
DreLSS                   QRPDGSWEGSWGVCFTYGAWFGLEAFACMGHTFQN------GSVCEEVKRACEFLLAKQM 625
Haplochromis             QRPDGSWEGSWGVCFTYGMWFGLEAFACMGHVYEN------GHVCEEVQKACQFLLDRQM 548
Xenopus                  QRQDGSWEGSWGVCFTYGIWFGLEAFACMGHTYKE--------GCPEIIRACNFLLSHQM 546
Charadrius               QRADGSWEGSWGVCFTYGTWFGLEAFASMQHTYQD------RAACREVAQACQFLISKQM 548
Anolis                   QRADGSWEGSWGVCFTYGMWFALEAFACMQHTYRD------GVACKEISRACEFLISKQM 548
Alligator                QRSDGSWEGSWGVCFTYGTWFGLEALACMQHMYHD------RTACKEVAQACQFLVSKQM 548
Pneumocystis             QRPDGSWYGSWAICFTYATMFALESLSCVKDFY---------ENSFHSRRACDFLLNKQE 546
Arthrobotrys_            QRKDGSWEGSWGICFSYAMMFALESLRLVGYTY---------ESSERVRLACQFLVDKQM 547
Aspergillus              QRPDGSWYGSWGICFTYAAMFALESLASVGETY---------ETSEYARRGCEFLLSKQK 547
Spizellomyces            QKEDGSWFGSWAICFTYGTWFGLEALSHMGETY---------KNSERAQRACHFLLDHQM 541
Trichosporon             QRPDGSWYGSWGICFTYATMFALESLAIAGETY---------ENSESVRRACSFLLSKQM 532
Puccinia                 QRGDGSWYGSWGVCFTYATMFALESLSLNNETY---------KNSLSVKKACRFLLDRQM 552
Mixia                    QRPDGSWFGSWAICFTYATYFAVESLAVNGETY---------KSSERVKRACDFILSKQK 548
Microbotryum             QRPDGSWYGSWAICFTYAGMFAIESLSLAGEHF---------DNSESVKRACEFLLSKQM 548
Dacryopinax              QHKDGTWFGSWGICFTYATMFALESLSLAGETY---------GNSPAVRKACDFLVSKQM 546
Fistulina                QTPEGGWVGSWGICFTYAAFFATESLALAGETY---------SSSPYQRRACDFLLSKQR 546
Wallemia                 QREDGSWYGSWAICFTYAAMFALESLSSVGEHY---------ENSEKVRKACEFLLSKQK 545
Ceraceosorus             QRPDGSWFGSWAVCFTYAMMFALESLHLAGQSC---------ENSEPVRRACQFLLGKQR 544
Ustilago                 QRKDGSWFGSWAICFTYATMFAVESLMLAGHTY---------ENSDAIRKACEFLESKQM 545
Malassezia_              QRPDGSWYGSWAICFTYAAMFALESLKHAGYTY---------ENSEPVRRACTFLLSKQR 546
Fluviicola               QDPDGSWYGCWGVCYTYGTWFGIEGLLTAGHKHY-E----NGTPSPEIQKACDFLLSKQR 546
Methylobacter            QKADGSWYGGWAVCFTYATWFGVEAISKARGKGYHD----DAILTDRINIASGFLAGKQK 546
Methylobactes            QKADGSWYGGWAVCFTYATWFGVEAISKARGKGYHD----DAILTDRINIACGFLAGKQK 546
Methylosarcina           QKPDGSWYGGWAVCFTYATWFGVEALSKAKGKGYYD----DAALAASISKACAFLVGKQK 546
Aphanomyces              QYQDGSFFGKWGVCYTYGTMFAIKGMRAAGASD----------KDEDVQDAVSFLVNKQR 540
Cystobacter              QRPDGSWEGSWGICFTYGAWFGVWGLVAAGYSP----------SHPALQRACDFLISKQN 535
Galdieria                QRPDGSWYGSWGVCFTYGTWFGVEGLVAAGRTF---------ESCESLPKACRFLCSKQK 546
Selaginella              QRPDGSWYGSWGVCFTYGIWFGVLGLVAAGKSY---------KHSAAIRKACDFLVSKQL 549
Panax                    QLPDGSWYGSWGICYTYGTWFGIKGLVAAGRTN---------RNCYSIRRACDFLLSKQL 559
Physcomitrella           QKDDGSWYGSWAVCFTYAIWFGVLGLIAAGQTY---------ESSFHIRMACKFLLSKQL 551
```

```
Cucumis                   QKTDGSWYGCWGVCFTYAGWFGIKGLVAAGRTY---------NNCVAIRKACNFLLSKEL 552
Adiantum                  QREDGSWYGSWGVCFTYAGWFGVIGLLSAGRTY---------ES-ETLKKACNFLLSKKL 551
Abies                     QRPDGSWYGSWGVCFTYGTWFGVLGLAAAGKTY---------QNCSNIRKACEFLLSKQL 553
Malus                     QATDGSWYGSWGVCFTYAGWFGIKGLVAAGRTY---------EDCSSIRKACDFLLSKEL 550
Amborella                 QRPDGSWYGSWGVCFTYATWFGVKGLVSAGKTY---------QTSPSIRKACDFLLSKQL 552
Nicotiana                 QAADGSWYGSWGVCFTYGTWFGVKGLLAAWRSF---------NNSSSIRKACDFLLSKQV 550
AtCAS1                    QAADGSWYGSWAVCFTYGTWFGVKGLVAVGKTL---------KNSPHVAKACEFLLSKQQ 650
Glycine                   QASDGSWYGSWGVCFTYGAWFGVKGLIAAGRSF---------SNCSSIRKACEFLLSKQL 551
Coffea                    QATDGSWYGSWGVCFTYGLWFGVKGLVASGRNF---------NNCSAIRKACDFLLSKQL 551
Populus                   QEKDGSWYGSWGVCFTYGTWFGINGLVAAGNFF---------NDNSSIRKACDFLLSKQC 551
Vitis                     QASDGSWYGSWGVCFTYAIWFGIKGLVAAGKNY---------NNCSSICKACDFLLSKQL 551
Micromonas                QRRDGSWYGSWAICFTYGTWFGIKGLMSTGSTF---------ETCEALRRAVKFLLSKQM 551
Volvox                    QRADGSWYGSWGVCFTYACWFGITGLVALGHNY---------HNDPAVRRCCEFLAVRQR 578
Chlorella                 QRPDGSWYGNWGVCFTYGTWFGCEALAAVGETH---------GSSASARAACAFLLQKQR 551
Synchroma                 QREDGSWYGSWGVCFTYGTWFGIEGLAAVGEA-----------DGPEVARAVRFLLAHQN 552
Chattonella               QRPDGSWYGSWGVCFTYATWFGIEGLVVSGEPI----------DSPSIKKACEFLVQHQN 546
Ochromonas                QRSDGSWYGSWGNCFTYGTWFGIEGLIAAGEPL----------DSKNMRRAVQFVLSKQN 556
Chromulina                QRYDGSWYGSWGTCFCYGTWFAIEGLIAAGEST----------TSPAIIKATEFLVSKQN 555
Chondrus                  QKQDGSWYGSWGICFLYAIWFAIDAYTSMGLTL---------ETSPSMERACQFVLNKQR 549
Acanthamoeba              QRPDGSWYGSWGVCFTYGTWFGVEGLMAAGEPS----------DSPYIQRACQFLLSKQN 541
Dictyostelium             QRQDGSWLGSWGICFTYGTWFGIEGLVASGEPL----------TSPSIVKACKFLASKQR 535
Polysphondylium           QRDDGSWLGSWGICFTYGTWFGVEGLVASGEPL----------NSPHLVKACKFLISKQR 536
Acytostelium              QRDNGSWLGSWGICFTYGTWFGVEGLVAAGEPL----------TSPHIVKACKFLLSKQR 536
SHC-Candidatus            QEKDGSWYGRWGVNYIYGSFLAMRGLTTSGA----------PGSQDAVDRAARWLRAIQN 455
SHC-Chlorogloeopsis       QEPQGCWFGRWGVNYIYGTSGVLTALSVIAP----------QTYQNNIERGAAWLIECQN 511
SHC-Streptomyces          QEENGAWFGRWGVNYLYGTGAVVPALVTAGL----------PVSHPAIRRAVGWLESVQN 508
SHC-Hyphomicrobium        QEKDGSWYGRWGMNYIYGTWSVLCALNAAGV----------DPSAPPMRKAVDWLVSIQN 515
SHC-Leptospirillum        QEEDGSWFGRWGVNYIYGTWSVVAGLKSIGV----------PMSEPWVMRSMEFLLARQN 481
Naegleria                 QMEEGGWYGGWAVCFCYGTWFATTAIVSADPTLN-------YGNSQALKRGCDFIVAKQM 570
Symbiodinium              QRNDGSWYGCWGNCFTYGCWFGIEGLLCAGRP----------RSCAAIQKCVKFLLGKQN 509
Prorocentrum              QRDDGSWYGCWGNCFTYGCWFGVEGLLAAGXA---------AALR---AHPPCFLLQRQE 460
Gymnodinium               QREDGSWYGCWGNCFTYACWFGVDGLLHSGET---------PECSDAIKRCMRFLIGKQN 417
Alexandrium               QRQDGSWYGCWGCCFTYGCWFGIEGLVCAGED---------PARCTAIQRCKTFLLEKQN 559
Dinophysis                QRHDGSWYGCWGSCFTYGCWFSVEGLVCAGRD---------PVASPAIRRCVEFLLSKQN 558
Scrippsiella              QRDDGSWYGCWGNCFTYGCWFSIEGLRCAGRD---------PATNSAIKRCVAFLLSKQN 564
Aureococcus               QRADGSWYGSWACCFTYAGWFGIEGLVDSGEDLRE-----DPKTSEPVARACAFLLRHQR 569
Phaeodactylum             QREDGSWYGSWACCFCYGSWFGIEGLVKCGE----------PVSSEFIAKACKFLLQHQR 569
Helicotheca               QREDGSWYGSWACCFTYGCWFGIEGLIKAGE----------SNTCPEIQRCCAYLISKQR 576
Yeast-erg7                QLPDGSWYGSWGICFTYAGMFALEALHTVGET---------YENSSTVRKGCDFLVSKQM 627
Candida                   DNIDGSWYGCWGICYTYASMFALEALHTVGLD---------YESSSAVKKGCDFLISKQL 554
Verrucomicrobia           QRPDGSWEAVWGICFNYGTSFAIPGLLSAGL----------PQDDIRIVRGRKFLLQQQL 506
Eudoraea                  QYEDGSWEALWGLCFTYGTCFVLEGLSMYGM----------NKDHEAIQRACTYLWSKQK 527
Plesiocystis              DPKAGAWRGFWGVNYTYGTYFAVSALLAAGV----------EREHLVVRRAVRFLLDRQR 607
Enhygromyxa               QASNGAWPGFWGVNYTYGTFFSVAGLLAAGL----------DGEHVAVRRAVRHLISAQR 559
Bodo                      QGPDGSFYGSWAVCFTYGAWIVTDALHMAGF----------DETSPVFKKACAFLLGKQR 551
Trypanosoma               QRPDGSFYGSWGVCFTYAAWIVASALCISREIPD-------MANHPSCVRLIDFLLSHQN 559
Phytomonas                QNPDGSFYGSWGVCFTYAAWLVSDALRISKEFSN-------IADHPRCRLLADFLMSHQN 494
Leishmania                QQPDGGFYGSWAVCYTYAAWLVADALQASKELPE-------MASHPHCRRLIDFLLSHQA 594
Leptomonas                QQPDGGFYGSWAVCFTYGALDRRGCAACV------------------------------- 571
Labilithrix               QRPDGSFEGSWGVCFTYGTWFGVTGLLAAGV----------PTSDRAIRSACDFLLAYQR 529
Stigmatella               QREDGSWMGSWGVCFTYGTWFGVMGLIAAGA----------SPDDMALRRATAFLRSYQR 499
Myxococcus                QRGDGSWEGSWGVCFSYGTWFAVSGLVAGGA----------TRADPALRRAVRFLEEHQR 523
Corallococcus             QRPDGSWEGAWGVCFSYGTWFGVTGLVASGA----------GTGDPALRKAVTFLKAHQR 533
S-cinnamoneus             QNPDGSFTSSWGINYTYAAFHAARGLRAAGA----------SPGDPSLRALGQWLVATQL 543
S-caatingaensis           QNPDGSFAGTRGIHPTYAAFHAARGLRAAGV----------RRDDPAPAALGRWLAAAQL 517
Sandaracinus              QRPDGSWPGMWGVHFVYGTMFGVRGLLAGGV----------PPHDPRIRRACRFLEERQR 536
S-alboviridis             QDADGSWPAAWGINRIYGTLFAIRGLLATGV----------PRTHPCFVRAGWWLESIQL 525
G-obscuriglobus           QRPDGAFPGAWGVYLTYGTFHAVRGLRAAGY----------APSHRALQRAANWLIATQK 548
G-sp.SH-PL17              QHSGGAFPGAWGVYFTYGTFHAVRGLRAAGY----------GS-NPALQRAADWLIRHQK 524
G-sp-IIL30                QHSGGAFPGAWGVYFTYGTFHAVRGLRAAGY----------GS-NPALQRAADWLIRHQK 543
Methylococcus             QLDDGSFPGFWGINYTYAVFHVAKGLRMAGV----------EPADPVLQAAAGWLLEKQR 541
Methylocaldum             QLADGSYPGFWGINYTYAIYMASKGLRAAGM----------PASDPTLQAAARWLVRKQR 543


Helobdella                P--TNGGWGEKFESCELKRYIDSTSSATAT----TKTTSSSSSST-EEPQVVNTSWALLG 606
ScLDS                     T---DGGWRMG------------------------------------------------- 553
AjLDS-b                   E---DGGWGEKFASCSERRYVQSE-----------------------KSLVVNTSWALLG 658
AjLDS-a                   E---DGGWGEKFASCSERRYVQSE-----------------------KSLVVNTSWALLG 658
PpLDS                     E---DGGWGEKFASCSERRYVQSE-----------------------KSLVVNTSWALLG 658
ScPS                      E---DGGWGEKFASCSERRYVQSE-----------------------KSLVVNTAWALLG 584
PpPS                      E---DGGWGEKFASCSERRYVQSE-----------------------KSLVVNTSWALLG 653
AjPS-b                    E---DGGWGEKFASCSERRYVQSE-----------------------KSLVVNTSWALLG 653
AjPS-a                    E---DGGWGEKFASCSERRYVQSE-----------------------KSLVVNTSWALLG 653
Capsaspora                A---DGSWSESYMSCVERRYIEHT-----------------------ESQVINTAWAVLG 581
Saccoglossus-LSS          S---DGGWGENFESCEKRQYIESD-----------------------NSQIVNTAWALLG 666
EpLSS                     A---DGGWGEKFESCETRTYVQSE-----------------------TSQGVQTCWALMA 656
SpLSS                     E---DGGWGEKFESCEERCYVQCE-----------------------TSQVINTCWALLG 654
SgLSS                     ------------------------------------------------------------ 455
LspLSS                    E---DGGWGEEFESCEERRYIQSK-----------------------TSQVVNTAWSVLA 656
MgLSS                     E---DGGWGEEFESCEERRYIQSK-----------------------TSQVVNTAWAVLA 434
AaLSS                     E---DGGWGEEFESCEERRYIQSK-----------------------TSQVVNTAWSVLA 315
AfLSS                     E---DGGWGEEFESCEERRYIQSK-----------------------TSQVVNTAWSVLA 656
ArLSS                     E---DGGWGEEFESCEERRYIQSK-----------------------TSQVVNTAWSVLA 656
HspLSS                    E---DGGWGEEFESCEVRRYVQSK-----------------------TSQVVNTAWAVMG 656
EsLSS                     E---DGGWGEEFESCEERRYIQSK-----------------------TSQVVNTAWAVMG 656
ApLSS                     E---DGGWGEEFESCEERRYVQSK-----------------------TSQVVNTAWAVLA 656
PpLSS                     ------------------------------------------------------------ 429
PmLSS                     E---DGGWGEEFESCEERRYIQSK-----------------------TSQVVNTAWAVLA 613
```

```
Amphimedon                S---DGGWGEDFESCEKRVYVQSE-----------------------ESQVVNTCWALLG 580
Aplysia                   E---DGGWGENFESCEKRQYVPSE-----------------------TSQIINTCWALLA 587
Capitella                 H---DGGWGENFEACEIKEYVPAK-----------------------KSQTVNTAWALMA 588
Branchiostoma-LSS         KAEAGGGWGENFESCEERKYVQSD-----------------------TSQVVNTCWALLG 565
Lingula                   A---DGGWGENFESCELREYVQSD-----------------------TSQVVNTCWALLG 582
Callorhinchus             V---DGGWGEDFESCESRQYVQSS-----------------------SSQVHNTCWALLA 581
HumanLSS                  A---DGGWGEDFESCEERRYLQSA-----------------------QSQIHNTCWAMMG 658
Pteropus                  A---DGGWGEDFESCEQRRYVQSA-----------------------QSQIHNTCWALMG 582
DreLSS                    E---DGGWGEDFESCEQRRYVQSS-----------------------SSQIHNTCWALLG 659
Haplochromis              P---EGGWGEDFESCEQRRYIQSG-----------------------SAQIHNTCWALLG 582
Xenopus                   E---DGGWGEDFESCEQRRYVQSA-----------------------GSQIHNTCWALMG 580
Charadrius                A---DGGWGEDFESCEQRTYVESA-----------------------MSQIHNTCWALLG 582
Anolis                    E---DGGWGEDFESCEQRTYVQSA-----------------------TSQIHNTCWALLG 582
Alligator                 E---DGGWGEDFESCEQRRYVQSA-----------------------TSQIHNTCWALLG 582
Pneumocystis              K---DGGWSESYQSCTDGIWTR----------------------HPNGSQVVQTAWACIG 581
Arthrobotrys_             P---DGGWGESYMSCVKGQWID----------------------HE-KSQVVNTSWALIA 581
Aspergillus               E---DGGWGESYLSSEKHVYVQ----------------------HE-KSQVVQTAWACLA 581
Spizellomyces             A---DGGWGEAYKSCETNVYHH----------------------HE-KSQVVNTAWACMG 575
Trichosporon              E---DGGWGETYMSCVSLQYAQ----------------------HE-QSQVVQTAWALLA 566
Puccinia                  A---DGGWGESFKSCEQGVYIH----------------------HQ-TSQVFQTAWAVLA 586
Mixia                     E---DGGWGESYKSCETGEYVQ----------------------HE-RSQVVNTSWAVLT 582
Microbotryum              E---DGGWGESYKSCETEQYVQ----------------------HA-QSQVTQTAWAVIG 582
Dacryopinax               P---DGGWGESYKACETGEWVD----------------------HA-QSQVVNTSWATLA 580
Fistulina                 T---DGGWGESYKSCEQSKWIE----------------------HE-NTQLVQTCWAAMA 580
Wallemia                  E---DGGWGESYLSCETEEYTQ----------------------HQ-DSQVVQTSWVVLA 579
Ceraceosorus              E---DGGWGEDFKSCVEGRYVQ----------------------AA-TSRVVQTSWAVIA 578
Ustilago                  L---DGGWGETYESCVKGVYTH----------------------AA-KSQVVQTAWVVIT 579
Malassezia_               E---DGGWGESYKSCETHEYVQ-----------------------S-DSQVVQTSWAIIG 579
Fluviicola                A---DGSWGESFQSCVEHRYVE----------------------HE-DGQIVNTAWALLA 580
Methylobacter             A---DGGWGETFESCSKLVYTE----------------------AA-KSQVVNTAWALLA 580
Methylobactes             A---DGGWGETFESCSKLVYTE----------------------AA-KSQVVNTAWALLA 580
Methylosarcina            A---DGGWGETFKSCSTLVYTE----------------------AE-TSQVVNTAWALLA 580
Aphanomyces               N---DGGWSESFFACSTRRYLE----------------------TP-SSLVVNTAWALIG 574
Cystobacter               A---DGSWGETPESCRQRRYVQ----------------------AE-QGQAVMTSWAVLA 569
Galdieria                 S---DGSWGESYRSCTDKVWME--------------------AE---QGQVVHTAWAVIA 580
Selaginella               P---SGGWGESYLSSQNKVYMN--------------------LEGG-KHHLVNTAWAMLA 585
Panax                     G---SGGWGESYLSCQNKVYTS--------------------IEGN-ISHVANTGWAMLA 595
Physcomitrella            P---DGGWGESYRSCQDKVYSN--------------------LPGG-KSHVVNTSWAMLT 587
Cucumis                   P---GGGWGESYLSCQNKVYTN--------------------LEGN-KPHLVNTAWVMMA 588
Adiantum                  S---SGGWGESYLSCQDKVYTN--------------------LPND-RPHVVHTSWAMLA 587
Abies                     P---SGGWGESYLSCQEKVYTH--------------------LEGG-RSHIVSTAWAMLA 589
Malus                     P---SGGWGESYLSCQNKVYTN--------------------LKDN-RPHIVHTAWAMLA 586
Amborella                 P---SGGWSESYLSCQDKVYSN--------------------LKGN-RLHLVNTAWAMLA 588
Nicotiana                 L---SGGWGESYLSCQNKVYTN--------------------LEGN-RSHIVNTAWAMLA 586
AtCAS1                    P---SGGWGESYLSCQDKVYSN--------------------LDGN-RSHVVNTAWAMLA 686
Glycine                   P---SGGWGESYLSCQNKVYSN--------------------LEGN-RSHVVNTGWAMLA 587
Coffea                    P---SGGWGESYLSCQNKVYSN--------------------LEGN-RSHMVNTAWAMLA 587
Populus                   S---SGGWGESYLSCQNKVYSN--------------------LEGN-KTHVVNTAWAMLA 587
Vitis                     A---SGGWGESYLSCQNKVYSN--------------------LDGN-RSHVVNTAWAMLA 587
Micromonas                P---CGGWGESYLSSQTKQYVQ--------------------LEGG-VSHVVNTAWAMLA 587
Volvox                    E---DGGWGESYLSCQDKVYSQ--------------------LDGD--SHVVNTSWAMLA 613
Chlorella                 S---DGGWGESYLSCQDKAYSQ--------------------LEGE-QPHAVNTAWAMLA 587
Synchroma                 D---DGGWGESYLSCMDKAYAEEGTGG-------------GALGQD-GSGVVQTSWALLG 595
Chattonella               E---NGGWGESYLSCVDKAYAEDGAG--------------E-LGNG-KSGVVQTAWALLG 587
Ochromonas                A---NGGWGENYLACVNKHYPEDGTG--------------KNLGDE-GSGVVQTAWALLS 598
Chromulina                P---NGGWGESYLACVNKSYPYDGTG--------------ETLGED-SSGVVQTAWAVLS 597
Chondrus                  E---DGGWGESYLSSETMVYTQ----------------------SE-ESLVVSTGWALVA 583
Acanthamoeba              H---EGGWGETFESCSTKQYVQ----------------------NE-ETQVVNTAWAVLT 575
Dictyostelium             A---DGGWGESFKSNVTKEYVQ----------------------HE-TSQVVNTGWALLS 569
Polysphondylium           E---DGGWGESFRSNVTKNYVQ----------------------HE-QSQIVNTGWALLS 570
Acytostelium              D---DGGWGESFMSNVTKEYVH----------------------ND-QSQIVNTGWALLT 570
SHC-Candidatus            P---DGGWGESCASYARDG------------------------YVAAPSSASQTAWALLG 488
SHC-Chlorogloeopsis       P---DGGWGETCRSYDDPSL-----------------------KGKGRSTASQSAWALIG 545
SHC-Streptomyces          D---DGGWGEDLRSYRDRDW-----------------------IGRGASTPSQTAWALLA 542
SHC-Hyphomicrobium        S---DGGWGEDGESYSLDYK----------------------GYERAPSTASQTAWALMG 550
SHC-Leptospirillum        P---DGGWGEDCLSYASRDF-----------------------AGRGASTPSQTAWALIA 515
Naegleria                 E---DGGWGESYLSCVTHRYSH-----------------------AETSRVISTAWSLLA 604
Symbiodinium              A---DGGWGEDFSSCFDREYAARD-KL---------------YGCDSGSTVVQTSWALLA 550
Prorocentrum              P---DGGWGEDFLSCFNREYTTCD-RL---------------YGCDSGSSVVCTAWALLA 501
Gymnodinium               E---DGGWGEDFASCFNREYAAMD-QL---------------YGSDSGSTAVQTAWALLA 458
Alexandrium               D---DGGGARTSQAVSTXEYASRK-KL---------------YGCEAGSTVVQTAWALLG 600
Dinophysis                A---DGGWGEDFASCFNREYASRE-RL---------------YGCDSGSTVVCTAWALLA 599
Scrippsiella              A---DGGWGEDFLSCFNREYASRD-KI---------------YGCDSGSTVVCTAWALLG 605
Aureococcus               P---NGGWGEDFTSCFDKAYAKHGMEA---------------YGDAEGAGVVCTGWALLG 611
Phaeodactylum             S---NGGWGEDFTSCYDKEYAANGMEA---------------YGD-DGSGVVNTSWALMA 610
Helicotheca               E---NGGWGEDFTSCYDKDYAVNGMKD---------------YGD-DGSGVVNTGWALLA 617
Yeast-erg7                K---DGGWGESMKSSELHSY----------------------VDSEK-SLVVQTAWALIA 661
Candida                   P---DGGWSESMKGCETHSY----------------------VNGEN-SLVVQSAWALIG 588
Verrucomicrobia           P---DGGWGEHPDSCLERRP----------------------IPTPK-SLVEPTALAVLA 540
Eudoraea                  D---DGGWGEAQESALAREY----------------------IQAEN-SVVDQTAWSIIA 561
Plesiocystis              A---DGGWAEDYRGLLERDSGTLLSRTLGAKREPRRGAPSWDLADDEASRVTQTAWAVAT 664
Enhygromyxa               D---DGGWGERYEGVLESRERP--------------------LDADQPSRIVQTAWALLT 596
Bodo                      A---DGGWGEDFNSCVRQVW----------------------VENPDGSQVVNTAWAVMA 586
Trypanosoma               A---DGGWGEDVTASVRSLW----------------------VDNPSGSQVVNTAWAVMA 594
Phytomonas                N---DGGWGEDINACVRQEW----------------------VDNPDGSQVVNTAWAVMA 529
Leishmania                A---DGGWSEDISASARQTW----------------------VDSPDGSQVVNTAWAVMA 629
Leptomonas                ------------------------------------------------------------ 571
Labilithrix               S---DGGWGEEGDSCRERRY----------------------IPSDT-AGVVQTSWALST 563
```

```
Stigmatella              A---DGAWSEVVESCRQARW----------------------VEGKQ-GHAVNTSWALLT 533
Myxococcus               E---DGAWSETLQSCWERRW----------------------VEGVT-GHAVTTSWALLA 557
Corallococcus            E---DGAWSETIQACRERRW----------------------VEGRT-GHAVMTSWSVLS 567
S-cinnamoneus            A---DGGWGEDWRSCPERRY----------------------IALGH-SLPGMTSWAVLA 577
S-caatingaensis          A---DGGWGEDWRGVVEGRH----------------------LPLGH-GLPETTGWAVLA 551
Sandaracinus             A---DGAWGEHRSSVIVGRY----------------------VDHDE-GQAVQTAWAMTA 570
S-alboviridis            A---DGGWGEDHTGCIDNRY----------------------VPGAT-SQPVSTAWALLA 559
G-obscuriglobus          R---DGGWGEDYHGCLRQEY----------------------VEHPE-SQATMTSWAIVA 582
G-sp.SH-PL17             P---DGGWGEHYTSCLRQEC----------------------VEHPE-SQATMTSWAVLA 558
G-sp-IIL30               P---DGGWGEHYTSCLRQEC----------------------VEHPE-SQATMTSWAVLA 577
Methylococcus            S---DGGWGEHYSSCLEGRY----------------------VESRH-SQTVMTAWALLA 575
Methylocaldum            P---DGGWGEHYSGCLKGRY----------------------VAHVR-SQVVMTSWALLA 577


Helobdella               LMAAGYP---------------DVNCIERGVRYLLGMQNDDGSYPKQNVSGV-FNKTCSI 650
ScLDS                    ------------------------------------------------------------ 553
AjLDS-b                  LMAVRYP---------------DQAVLERGIQVLRDRQHEDGDWPQETISGV-FNRSCAI 702
AjLDS-a                  LMAVRYP---------------DQAVLERGIQVLRDRQHEDGDWPQETISGV-FNRSCAI 702
PpLDS                    LMAVRYP---------------DQSVLERAIQVLRDRQHEDGDWPQETISGV-FNRSCAI 702
ScPS                     LMAVRYP---------------DETILSRGVKVLRDRQTPDGDWPQETISGV-FNRSCAI 628
PpPS                     LMAVRYP---------------DEEVLSRGVKVLLDRQIDDGDWPQESICGV-FNKSCAI 697
AjPS-b                   LMAVRYP---------------DEEVLSRGVKVLLDRQIDDGDWPQESICGV-FNKSCAI 697
AjPS-a                   LMAVRYP---------------DEEVLSRGVKVLLDRQIDDGDWPQESICGV-FNKSCAI 697
Capsaspora               LMAAEYP---------------DRTVVERGIQFIMSKQLMNGDWPDDEIKGV-FNKNCMI 625
Saccoglossus-LSS         LMAVRYP---------------DISVLEKGVQLIMSRQLPNGDWPQENISGV-FNKSCAI 710
EpLSS                    LMAVRYP---------------DRTVIDRGIKLIMDTQLENGDWPQANIMGV-FNKSCAI 700
SpLSS                    LMAVRYP---------------DRRVMDRGIQLIMSRQSENGDWPQENINGV-FNKSCAI 698
SgLSS                    ------------------------------------------------------------ 455
LspLSS                   LMAVRFP---------------ERDVIDRGIQVIRDRQLPNGDWAQENIKGV-FNKSCAI 700
MgLSS                    LMAVRFP---------------EREVIDRGIRVIRDRQLSNGDWAQENIKGV-FNKSCAI 478
AaLSS                    LMAVRFP---------------EREVIDRGIRVIRDRQLPNGDWAQESIKGV-FNKSCAI 359
AfLSS                    LMAVRFP---------------EREVIDRGIRVIRDRQLPNGDWAQESIKGV-FNKSCAI 700
ArLSS                    LMAVRFP---------------EREVIDRGIRVIRDRQLPNGDWAQESIKGV-FNKSCAI 700
HspLSS                   LMAVRFP---------------EKDVIDRGIKVIMDRQLPNGDWAQENIKGV-FNKSCAI 700
EsLSS                    LMAVRFP---------------DKDVIDRGIKVIMDRQLPNGDWAQENIKGV-FNKSCAI 700
ApLSS                    LMAVRFP---------------ERDVIDRGIKVIMDRQLPNGDWAQENIKGV-FNKSCAI 700
PpLSS                    ------------------------------------------------------------ 429
PmLSS                    LMAVRFP---------------QREVIDRGIKVIMDRQLPNGDWAQENIKGV-FNKSCAI 657
Amphimedon               LMAVRYP---------------HTDVIKNGIKLIVSRQLDSGEWKQEGIKGV-FNKTCAI 624
Aplysia                  LMAVKYP---------------DVEVLEKGIKLIMSRQLENGDWPQENISGV-FNKSCAI 631
Capitella                LMAVRYP---------------DMEVLEGGIKILLKRQYSNGDFPQENIMGV-FNKSCAI 632
Branchiostoma-LSS        LMAVRYP---------------DLSVLEPGVKFLMERQMENGDWPQENICGV-FNKSCAI 609
Lingula                  LMAVRYP---------------DVALIEHGIRVIMQRQLDNGDWPQENISGV-FNKSCAI 626
Callorhinchus            LMAARYP---------------GTRAIEKGIKTLINKQQLNGDWPQENIAGV-FNKSCAI 625
HumanLSS                 LMAVRHP---------------DIEAQERGVRCLLEKQLPNGDWPQENIAGV-FNKSCAI 702
Pteropus                 LMAVRHP---------------DVEALERGVRCLLRKQLPNGDWPQENVAGV-FNKSCAI 626
DreLSS                   LMAVRYP---------------GTKVIERGIQLLIDKQLPNGDWPQENISGV-FNKSCAI 703
Haplochromis             LMAVRHP---------------DRKSIERGVQMLIDKQLPNGDWPQENIAGV-FNKSCAI 626
Xenopus                  LMAVGFP---------------DVTVLERGVRLLLDKQLSNGDWPQENISGV-FNKSCAI 624
Charadrius               LMAVRYP---------------DINVLERGIKVLIDKQLPNGDWPQENIAGV-FNKSCAI 626
Anolis                   LMAVRYP---------------DVKVLEKGIKLLIDKQLPNGDWPQENISGV-FNKSCAI 626
Alligator                LMAVRYP---------------DVEVLEKGIKLLMDKQLPNGDWPQENIAGV-FNKSCAI 626
Pneumocystis             LMYANYP---------------DKTPIKRGIDLIMSRQQSNGEWKQEAIEGV-FNKNCMI 625
Arthrobotrys_            LLQAGYP---------------GKEVIKRGVDLLKSRQQKNGEWLQEGIEGV-FNRSCMI 625
Aspergillus              LMEAEYP---------------HKEPLQKAMKLLMSRQQPNGEWLQESIEGV-FNQSCMI 625
Spizellomyces            LMAAGYP---------------DPTPIKRGIRLIMSRQKPDGRWEQEGIEGV-FNRNAMI 619
Trichosporon             LIYGRCP---------------DTAAIRRGCQLIMSRQRPDGSWLQEDTEGI-FNKNCAI 610
Puccinia                 LLAAKYP---------------EPEPIRRACRLIISRQTADGQWLDDAVEGV-FNKTTSV 630
Mixia                    LLTAKCP---------------DKAAVKRGVGLIMARQMPDGSWAQEAIEGI-FNKNCAI 626
Microbotryum             LITAQYP---------------DTKPIRRGCRLIMSRQLPSGEWPQEAIEGV-FNKNAAI 626
Dacryopinax              LVYAQYP---------------HRLPIERAVKLVISRQLPDGSWKQEAIEGV-FNKNCAI 624
Fistulina                LMHAKYP---------------KAEPIERAVKLVMSRQKPDGSWAQEAIEGV-FNKTCAI 624
Wallemia                 LLHARYP---------------HKEPIKRAVRLIMSRQNKDGSWTQEAIEGI-FNKNCAI 623
Ceraceosorus             LLHAGCE---------------DKDAIKRGVSLIMGRQLDDGSWADEETVGV-FNRNCSI 622
Ustilago                 LLHAKYP---------------KKDRIKKAVKVIMDRQLSDGSWAQEQIEGI-FNRNCAI 623
Malassezia_              LLHAGYP---------------EAKPIQRAVTLIMQRQLPDGSWAQEQIEGI-FNQ---- 619
Fluviicola               LMKAKHP---------------NKAVIEKGIEFILSRQESTGDFPQEGVSGI-FNGNCAI 624
Methylobacter            LMAADFG---------------DRKVIESGITLLLNRQTHTGDWPQESISGV-FNYNCMI 624
Methylobactes            LMAADFG---------------DRKVIESGITLLLNRQTNIGDWPQESISGV-FNYNCMI 624
Methylosarcina           LMAAEFG---------------EKEVIEAGIQVLLSRQSRIGDWPQESISGV-FNYNCMI 624
Aphanomyces              IMKGIEGGDP-------QMHVKEWDAVKKGIEFLVAKQLPSGDWAQERISGV-FNRTCGI 626
Cystobacter              LSKAGRR---------------DSPEVQRALGFLVQRQRPDGSYPEEHIAGM-FNKTSGI 613
Galdieria                LIKAICTSHSMWHSDVHVSHQAYHSSIRKGIDFLLESQLENGDWPQQRISGV-FNRNCMI 639
Selaginella              LIAGGQASLDN-------LAERDSTPLHKAAALLVNGQLEDGDFPQEEIIGV-FNGNCMI 637
Panax                    LIEAGQA-------------QRDPSPLHRAAKVLMNSQMKNGVFPQQEIVGV-FNKNCMI 641
Physcomitrella           LIAAGQW-------------ERNPEPLHRAASVLINKQMESGDFPQEEIMGV-FNRNCMI 633
Cucumis                  LIEAGQG-------------ERDPAPLHRAARLLINSQLESGDFPQQEIMGV-FNKNCMI 634
Adiantum                 LLYAGQA-------------ERDPRPLHEAATVLINSQLENGDYPQQEITGV-FNRNCMI 633
Abies                    LIYAGQA-------------LRDPKPLHRAAIVLVNYQMENGDFPQQEIMGV-FNRNCMI 635
Malus                    LIGAGQA-------------KRDPTPLHA-ARVLINSQMKNGDFPQKEITGV-FNKNCMI 631
Amborella                LIDAGQA-------------ERGPTPLHRASRVLINSQMDNGDFPQQEIMGV-FNRNCMI 634
Nicotiana                LIEAGQG-------------KRDPAPLHRAAKVLINSQLENGDFPQQEIIGV-FNKNCMI 632
AtCAS1                   LIGAGQA-------------EVDRKPLHRAARYLINAQMENGDFPQQEIMGV-FNRNCMI 732
Glycine                  LIDAGQA-------------KRDSQPLHRAAAYLINSQLEDGDFPQQEIMGV-FNKNCMI 633
Coffea                   LIDAGQT-------------ERDPTPLHIAAKVLINAQFENGDFPQEEIMGV-FNKNCMI 633
Populus                  LIEAGQA-------------ERDPEPLHRAARSLINSQMENGDFPQQEIMGV-FNRNCMI 633
Vitis                    LIDAGQA-------------ERDPTPLHRAARVLINSQMENGDFPQEEIMGV-FNRNCMI 633
Micromonas               LLASGQI-------------SRDPLPLHRGARSLMRAQCSNGDWPQQTIMGV-FNNNCMI 633
```

```
Volvox                   LLAAGYH-------------RVDPAPFHRAARFLLRMQLPSGDWPQQHISGV-FNRNCMI 659
Chlorella                LLAAGYE-------------QVDRKPLDAAARCLIRLQEESGDWPQQHISGV-FNRNCMI 633
Synchroma                LLAAGCE---------------DRDAMRRAAELLMRRQRDDGDWDQEAMTGV-FNRSCGI 639
Chattonella              LMEAHYP---------------DRAVIDRGINYLRAQQNQIGDWAQEGITGV-FNRNCGI 631
Ochromonas               LMAAESS---------------DLKAIQRGVQFLISKQLPSGDWDQEGITGV-FNRSCGI 642
Chromulina               LIKADYQ---------------DKLVIDRGIKFLLDKQLPNGDWDQEGITGV-FNRSCGI 641
Chondrus                 LSMARWP---------------DREPLEKASQFLIRSQDENGDWPQQNICGV-FNRNCMI 627
Acanthamoeba             LLKAQWP---------------DRRPVDRAVQVLMKRQLPNGNWPQEDIKGV-FNANCAI 619
Dictyostelium            LMSAKYP---------------DRECIERGIKFLIQRQYPNGDFPQESIIGV-FNFNCMI 613
Polysphondylium          LMAAKYP---------------DREPIERGIKYLISKQYPNGDFPQESIIGV-FNFNCMI 614
Acytostelium             LMAAKYP---------------QREPIERGIKFLISRQYPNGDFPQESIIGV-FNFNCMI 614
SHC-Candidatus           LCAAGDRD----S-----------AQFRRGVEYLLTLQAPDGKWPEGATTGTGFPNVFYL 533
SHC-Chlorogloeopsis      LIAAGKATGNFAK-----------PVLERGINYLLATQAADGTWDEADFTGTGFPCHFYL 594
SHC-Streptomyces         LLAAGERD----G-----------EAVRRGIDHLVRTQREDGSWDEPYFTGTGFPWDFSI 587
SHC-Hyphomicrobium       LMAAGEVD----H-----------PAVKKGVTYLTSKQGSDGFWGEERFTATGFPRVFYL 595
SHC-Leptospirillum       LLHGGHAG----H-----------MAVRQGVDYLIQQMTPEGTWNEELFTGTGFPRVFYL 560
Naegleria                LMTSNYPN----L-----------KVIEKGIYCLMRKQLPNGDFPQESISGV-FNHNCMI 648
Symbiodinium             LMAAKCED----S-----------DAIQRGISLLWRRQLPTGDWPQENIAGV-FNRSVGI 594
Prorocentrum             LMAGECDD----T-----------DAVRRGVDFLMRRQLPTGDWAQENIAGV-FNRSIGI 545
Gymnodinium              LMAGDCAD----T-----------TAVRRGIRFLMRRQLSSGDWAQENIAGV-FNRSIGI 502
Alexandrium              LMAGECED----T-----------AAVRRGIEFLMRRQLPSGDWAQENVAGV-FNRSVGI 644
Dinophysis               LTAGQCHD----R-----------SAVRRGAEFLMRRQLPSGDWAQENICGV-FNRSVGI 643
Scrippsiella             LMAGDCAD----S-----------AAVHRGIKLLMRRQLPSGDWAQENISGV-FNRSVGI 649
Aureococcus              LMAGACAD----A-----------DAVARGVAYLEARQLPDGDWPQEGISGV-FNRSCGI 655
Phaeodactylum            LSTAKCND----I-----------EAIKRGVQYLMKRQLPCGDWPQEGVAGV-FNRACGI 654
Helicotheca              LSAAKCDD----V-----------AAIRKGVQYLMKRQLPCGDWPQEGIAGV-FNRSCGI 661
Yeast-erg7               LLFAEYPN----K-----------EVIDRGIDLLKNRQEESGEWKFESVEGV-FNHSCAI 705
Candida                  LILGNYPD----E-----------EPIKRGIQFLMKRQLPTGEWKYEDIEGV-FNHSCAI 632
Verrucomicrobia          LLGCGPKE----D-----------PSVRKGIEFLLQQQQADGDFPPQPIPGL-FYRTTLI 584
Eudoraea                 LLNGGYAE----D-----------PRLIKAVNWLIDQQLEDGDWPVQPMSGL-FYKTTMI 605
Plesiocystis             LALAAPQR----A----------RQSVDAGLAYLLERQQADGTWEHDASVGV-FFNTAVL 709
Enhygromyxa              LQQAAPER----G----------REAIERGLRFLLDQQQPDGTWPHDAAVGV-FFNTAVL 641
Bodo                     LMAAGGAT----H----------RQEVERGIRFIMSRQLANGDWPQERISGV-FNGNAAI 631
Trypanosoma              IMSAAGEA----ARSLPRWREQISVAVERGIRLIMSRQLVTGDWAQERISGV-FNGNNPI 649
Phytomonas               IMNVSGDP----NHSSYARWHQIASAVERGIQLIMSRQLATGDWAQERLSGV-FNGNNAI 584
Leishmania               IISAAGKA----ASTEPTRQRCIRRAVDRGVQLIMSRQLASGDWRQERISGV-FNGNNPI 684
Leptomonas               ------------------------------------------------------------ 571
Labilithrix              LVRAQHPN----R-----------QAKARAAKLLVDRQESDGSWAREPLVGV-FNKTCLI 607
Stigmatella              LAAAGEGG----S-----------DAAQRGVRWLRERQQEDGRWPPEPIAGI-FNRTCAI 577
Myxococcus               LFACGEAD----S-----------KSTRRGVAWLRARQGADGRWPREPLAGI-YNRTGGI 601
Corallococcus            LVAAGEAN----A-----------EATRRGVAWLRERQEAEGQWPREPLAGV-FSRTCAI 611
S-cinnamoneus            GLDALGTA----H-----------PSVADGVRWLCSAQRPDGSWSEDQVNGV-FFTTMMI 621
S-caatingaensis          ALDTLGPR----H-----------PVVDRGVRWLCDHQRPDGSWENGHVNGV-LFTAGMV 595
Sandaracinus             LLEARHPD----F-----------APIERAARWLASKQSDDGAWPKQEAEGI-FFHTALL 614
S-alboviridis            LLELTGGR----S-----------RAVLSGIAWLCDRQLPDGSWPETAATGV-FFGTAML 603
G-obscuriglobus          LCETVGTG----H-----------PAVQKGAAWLASRQRADGSYPREAVNGV-FFGTAML 626
G-sp.SH-PL17             LCEVVGAK----H-----------TAVCRGVEWLAAHRSGAGRHPREAVNGV-FFGTAML 602
G-sp-IIL30               LCEVVGAK----H-----------TAVCRGVEWLAAHRSGAGRHPREAVNGV-FFGTAML 621
Methylococcus            LMEVYPAA----H-----------EAVERGIAWLCSQQGEDGGWPRQGMNGV-FFGAAML 619
Methylocaldum            LLEILPPD----H-----------ESVRRGFEWLIRQQRHDGGWPRQAVNGV-FFGSAML 621


Helobdella               HYDLYRNIFPMWVIARY------------------------------------------- 667
ScLDS                    ------------------------------------------------------------ 553
AjLDS-b                  SYPAFKNIFPIWALGRYSQLYTSSKEN-QD----------SWVRDDDWEKLSNNSQIAKL 751
AjLDS-a                  SYPAFKNIFPIWALGRYSQLYTSSKEN-QD----------SWVRDDDWEKLSNNWQIAKL 751
PpLDS                    SYPAFKNIFPIWALGRYSQLYTSSKET-QD----------SWVQNDDWEKLSNNWQLAKL 751
ScPS                     SYTSFKNIFPIWALGRYAQLYPSAT----------------------------------- 653
PpPS                     GYTAFKNIFPIWALGRHAQLYPSSFRTFSA----------ESVSDKDWEKIPHV------ 741
AjPS-b                   GYTAFKNIFPIWALGRHAQLYPCSSRTFSA----------ESVSDKDWEKIPHV*----- 741
AjPS-a                   GYTAFKNIFPIWALGRHAQLYPSSPRTFSA----------ESVSDKDWEKIPHV*----- 741
Capsaspora               VYPNYKNAFTIWALGRY------------------------------------------- 642
Saccoglossus-LSS         SYTSYRNVFPIWTLGRFARLYPDSNLVTPSFNGR-------------------------- 744
EpLSS                    SYTSYRNVFPIWCLGRYAKLYPTPDSTPSASTASTPLSDRSDSNEEGWEKL--------- 751
SpLSS                    SYTNYRNIFPIWTLGRYAKLYPNDA---------SPLEDKSDSNSSDWEKL--------- 740
SgLSS                    ------------------------------------------------------------ 455
LspLSS                   SYTSYRNLFPIWALGRYAQIYGDHA----------------------------------- 725
MgLSS                    SYTSYRNLFPIWALGRYARIYGDDA----------------------------------- 503
AaLSS                    SYTSYRNLFPIWALGRYARIYCDDA----------------------------------- 384
AfLSS                    SYTSYRNLFPIWALGRYARIYGDDA----------------------------------- 725
ArLSS                    SYTSYRNLFPIWALGRYARIYGDDA----------------------------------- 725
HspLSS                   SYTNYRNVFPIWALGRYARLYGNKE----------------------------------- 725
EsLSS                    SYTSYRNVFPIWALGRYARLYGNKE----------------------------------- 725
ApLSS                    SYTSYRNIFPIWALGRYARIYGNST----------------------------------- 725
PpLSS                    ------------------------------------------------------------ 429
PmLSS                    SYTSYRNVFPIWALGRYARIYGNSS----------------------------------- 682
Amphimedon               TYTSYKNVFPLWTLGRF------------------------------------------- 641
Aplysia                  SYTSYRNVFPIWTLGRF------------------------------------------- 648
Capitella                HYESYRNIFPVWALGRF------------------------------------------- 649
Branchiostoma-LSS        SYTSYRNVFTIWALGRFCRLYPHSALTGGAAKS--------------------------- 642
Lingula                  SYTSYRNVFPIWTLGRF------------------------------------------- 643
Callorhinchus            SYSAYRNIFPIWTLGRF------------------------------------------- 642
HumanLSS                 SYTSYRNIFPIWALGRFSQLYPERALAGHP------------------------------ 732
Pteropus                 SYSNYKNVFPIWALSRF------------------------------------------- 643
DreLSS                   SYTSYRNVFPVWTLGRFTRLYPCNALTGKLKL---------------------------- 735
Haplochromis             SYTSYRNVFPIWTLGRF------------------------------------------- 643
Xenopus                  SYTSYRNVFPIWTLGRF------------------------------------------- 641
Charadrius               SYTAYRNVFPIWTLGR-------------------------------------------- 642
```

```
Anolis                   SYTSYRNVFPIWTLGRF------------------------------------------- 643
Alligator                SYTSYRNVFPIWTLGCF------------------------------------------- 643
Pneumocystis             SYPNYKFNFTIKALGMY------------------------------------------- 642
Arthrobotrys_            TYPNYKFAFPIKALGMY------------------------------------------- 642
Aspergillus              SYPNYKFYWPIRALGLY------------------------------------------- 642
Spizellomyces            AYPNYKFIFSIWALGRF------------------------------------------- 636
Trichosporon             DYPAFKFIFCIWALGR-------------------------------------------- 626
Puccinia                 TYPNYK------------------------------------------------------ 636
Mixia                    SYPNYKFSWTIWALGR-------------------------------------------- 642
Microbotryum             SYPNYKFAWTINALGQ-------------------------------------------- 642
Dacryopinax              AYPNFKFSFTIWMLGR-------------------------------------------- 640
Fistulina                AYPNFKFSFPIWMLGK-------------------------------------------- 640
Wallemia                 SYPNYKFSFTIWALGK-------------------------------------------- 639
Ceraceosorus             SYPNYVHSFTIWALGK-------------------------------------------- 638
Ustilago                 SYPNYKFSFTIWALGK-------------------------------------------- 639
Malassezia_              ------------------------------------------------------------ 619
Fluviicola               TYTSYRNVFPLWAIGRY------------------------------------------- 641
Methylobacter            TYANYRNIFPVWALNRY------------------------------------------- 641
Methylobactes            TYANYRNIFPVWALNRY------------------------------------------- 641
Methylosarcina           TYANYRNAFPIWALNRY------------------------------------------- 641
Aphanomyces              TYANYRNIFPIWAIGLY------------------------------------------- 643
Cystobacter              HYDHYLDVFPLWALS--------------------------------------------- 628
Galdieria                SYSNYRNIFPLWAIALY------------------------------------------- 656
Selaginella              SYSAYRCIFPIWALGQY------------------------------------------- 654
Panax                    SYSAYRNIFPIWALGEY------------------------------------------- 658
Physcomitrella           SYSAYRCIFPIWALGEY------------------------------------------- 650
Cucumis                  TYAAYRNIFPIWALGEY------------------------------------------- 651
Adiantum                 SYSAYRNIFPIWALGEY------------------------------------------- 650
Abies                    SYSAYRNIFPIWALGEY------------------------------------------- 652
Malus                    SYSAYRNIFPIWALGEY------------------------------------------- 648
Amborella                SYSAYRNIFPIWALGEY------------------------------------------- 651
Nicotiana                SYSAYRNSFPIWALGEY------------------------------------------- 649
AtCAS1                   TYAAYRNIFPIWALGEYRCQVLLQQGE--------------------------------- 759
Glycine                  TYAAYRNIFPIWALGEY------------------------------------------- 650
Coffea                   TYAAYRNIFPIWALGEY------------------------------------------- 650
Populus                  TYAAYRDIFPIWALGEY------------------------------------------- 650
Vitis                    TYAAYRNIFPIWALGEY------------------------------------------- 650
Micromonas               TYANYRNIFPLWALGEY------------------------------------------- 650
Volvox                   TYANYRNIFPIWALGHY------------------------------------------- 676
Chlorella                TYANYRNIFPIWALGVY------------------------------------------- 650
Synchroma                TYTAYRNVFTTWALGRY------------------------------------------- 656
Chattonella              TYTAYRNIFPIWALGRY------------------------------------------- 648
Ochromonas               TYTAYRNVFPIWALARF------------------------------------------- 659
Chromulina               TYSQYRNIFPLWALGRY------------------------------------------- 658
Chondrus                 SYSQYRNIFPIWALAEY------------------------------------------- 644
Acanthamoeba             SYTAYKNIFPIWALGLY------------------------------------------- 636
Dictyostelium            SYSNYKNIFPLWALSRY------------------------------------------- 630
Polysphondylium          SYSNYKNIFPLWAISRY------------------------------------------- 631
Acytostelium             SYSNYKNIFPLWALARY------------------------------------------- 631
SHC-Candidatus           TYAMYRDYFPLLALSQ-------------------------------------------- 549
SHC-Chlorogloeopsis      KYHLYQEYFPLIALSQY------------------------------------------- 611
SHC-Streptomyces         NYHLYRQVFPLTALGRY------------------------------------------- 604
SHC-Hyphomicrobium       RYHGYSKFFPLWALARY------------------------------------------- 612
SHC-Leptospirillum       RYHMYRHYFPLWALALY------------------------------------------- 577
Naegleria                TYTNYRNIFPIWALSMY------------------------------------------- 665
Symbiodinium             TYTSFRNVFPLWALGHF------------------------------------------- 611
Prorocentrum             SYTSFRNVYPLWALGRF------------------------------------------- 562
Gymnodinium              TYTSFRNVFPLWALGRF------------------------------------------- 519
Alexandrium              TYTAFRNVFPLWA----------------------------------------------- 657
Dinophysis               TYTA-------------------------------------------------------- 647
Scrippsiella             TYTAFRNVFPLWALGRF------------------------------------------- 666
Aureococcus              TYTAYRNVFPMWALARY------------------------------------------- 672
Phaeodactylum            TYTAYRNIFPIWALGR-------------------------------------------- 670
Helicotheca              TYTSYRNVFPIWALGR-------------------------------------------- 677
Yeast-erg7               EYPSYRFLFPIKALGMYSRAYETHTL---------------------------------- 731
Candida                  EYPSYRFLFPIKALGLY------------------------------------------- 649
Verrucomicrobia          RYDHYKRAFPLKAFAKY------------------------------------------- 601
Eudoraea                 SYRNYKRYFSLLALKKY------------------------------------------- 622
Plesiocystis             DYRLYRQVFPTWALAR-------------------------------------------- 725
Enhygromyxa              DYRLYKLVFPTWALSRW------------------------------------------- 658
Bodo                     HYPGYKNSMPVWALGKY------------------------------------------- 648
Trypanosoma              HYPGYKNTMPVWALGMY------------------------------------------- 666
Phytomonas               HYPGYKNTMTVWALGMY------------------------------------------- 601
Leishmania               HYPGYKNSMPVWALGKY------------------------------------------- 701
Leptomonas               ------------------------------------------------------------ 571
Labilithrix              DYDNYRHYFPLWALSEF------------------------------------------- 624
Stigmatella              HYDAYLRIFPVWALA--------------------------------------------- 592
Myxococcus               HYDTYLRTFPLWALS--------------------------------------------- 616
Corallococcus            HYDAYLRIFPLWALS--------------------------------------------- 626
S-cinnamoneus            NYRMYPAYFFPTLALGRYLQAVTSLTPDSGTPQGAA------------------------ 656
S-caatingaensis          NHRLGAACFPALALGRY------------------------------------------- 612
Sandaracinus             DYVLYRRYFPVWALGLY------------------------------------------- 631
S-alboviridis            DYRLYREYFPLWALGRW------------------------------------------- 620
G-obscuriglobus          DYDLYRAYFPTWALALASGTTAKS------------------------------------ 650
G-sp.SH-PL17             DYDLYREYFPVWALAAAL------------------------------------------ 620
G-sp-IIL30               DYDLYREYFPVWALA--------------------------------------------- 636
Methylococcus            DYRLYPVYFPTWALARY------------------------------------------- 636
Methylocaldum            DYRLYHVCFPTRALSRY------------------------------------------- 638
```

Helobdella	---	667
ScLDS	---	553
AjLDS-b	VF*	753
AjLDS-a	VF*	753
PpLDS	VF-	753
ScPS	---	653
PpPS	---	741
AjPS-b	---	741
AjPS-a	---	741
Capsaspora	---	642
Saccoglossus-LSS	---	744
EpLSS	---	751
SpLSS	---	740
SgLSS	---	455
LspLSS	---	725
MgLSS	---	503
AaLSS	---	384
AfLSS	---	725
ArLSS	---	725
HspLSS	---	725
EsLSS	---	725
ApLSS	---	725
PpLSS	---	429
PmLSS	---	682
Amphimedon	---	641
Aplysia	---	648
Capitella	---	649
Branchiostoma-LSS	---	642
Lingula	---	643
Callorhinchus	---	642
HumanLSS	---	732
Pteropus	---	643
DreLSS	---	735
Haplochromis	---	643
Xenopus	---	641
Charadrius	---	642
Anolis	---	643
Alligator	---	643
Pneumocystis	---	642
Arthrobotrys_	---	642
Aspergillus	---	642
Spizellomyces	---	636
Trichosporon	---	626
Puccinia	---	636
Mixia	---	642
Microbotryum	---	642
Dacryopinax	---	640
Fistulina	---	640
Wallemia	---	639
Ceraceosorus	---	638
Ustilago	---	639
Malassezia_	---	619
Fluviicola	---	641
Methylobacter	---	641
Methylobactes	---	641
Methylosarcina	---	641
Aphanomyces	---	643
Cystobacter	---	628
Galdieria	---	656
Selaginella	---	654
Panax	---	658
Physcomitrella	---	650
Cucumis	---	651
Adiantum	---	650
Abies	---	652
Malus	---	648
Amborella	---	651
Nicotiana	---	649
AtCAS1	---	759
Glycine	---	650
Coffea	---	650
Populus	---	650
Vitis	---	650
Micromonas	---	650
Volvox	---	676
Chlorella	---	650
Synchroma	---	656
Chattonella	---	648
Ochromonas	---	659
Chromulina	---	658
Chondrus	---	644
Acanthamoeba	---	636
Dictyostelium	---	630
Polysphondylium	---	631
Acytostelium	---	631
SHC-Candidatus	---	549

SHC-Chlorogloeopsis	---	611
SHC-Streptomyces	---	604
SHC-Hyphomicrobium	---	612
SHC-Leptospirillum	---	577
Naegleria	---	665
Symbiodinium	---	611
Prorocentrum	---	562
Gymnodinium	---	519
Alexandrium	---	657
Dinophysis	---	647
Scrippsiella	---	666
Aureococcus	---	672
Phaeodactylum	---	670
Helicotheca	---	677
Yeast-erg7	---	731
Candida	---	649
Verrucomicrobia	---	601
Eudoraea	---	622
Plesiocystis	---	725
Enhygromyxa	---	658
Bodo	---	648
Trypanosoma	---	666
Phytomonas	---	601
Leishmania	---	701
Leptomonas	---	571
Labilithrix	---	624
Stigmatella	---	592
Myxococcus	---	616
Corallococcus	---	626
S-cinnamoneus	---	656
S-caatingaensis	---	612
Sandaracinus	---	631
S-alboviridis	---	620
G-obscuriglobus	---	650
G-sp.SH-PL17	---	620
G-sp-IIL30	---	636
Methylococcus	---	636
Methylocaldum	---	638

Supplementary Notes 3: List of echinoderm sterol biosynthetic enzymes.

>HumanCYP51
MLLLGLLQAGGSVLGQAMEKVTGGNLLSMLLIACAFTLSLVYLIRLAAGHLVQLPAGVKSPPYIFSPI
PFLGHAIAFGKSPIEFLENAYEKYGPVFSFTMVGKTFTYLLGSDAAALLFNSKNEDLNAEDVYSRLTT
PVFGKGVAYDVPNPVFLEQKKMLKSGLNIAHFKQHVSIIEKETKEYFESWGESGEKNVFEALSELIIL
TASHCLHGKEIRSQLNEKVAQLYADLDGGFSHAAWLLPGWLPLPSFRRRDRAHREIKDIFYKAIQKRR
QSQEKIDDILQTLLDATYKDGRPLTDDEVAGMLIGLLLAGQHTSSTTSAWMGFFLARDKTLQKKCYLE
QKTVCGENLPPLTYDQLKDLNLLDRCIKETLRLRPPIMIMMRMARTPQTVAGYTIPPGHQVCVSPTVN
QRLKDSWVERLDFNPDRYLQDNPASGEKFAYVPFGAGRHRCIGENFAYVQIKTIWSTMLRLYEFDLID
GYFPTVNYTTMIHTPENPVIRYKRRSK
>SpCYP51
MANSVNIFESVGGVFGEMTLATMILVSTIFVLGIAWAFKSLLGPQNKDVKLPPRLPTGIPFLGQAVAF
NQSPIDFLEDAYEKYGDVFSFTMVGKTFTYLIGSEASALLFNSKNENLNAEEVYSNLTVPVFGKGVAY
DVPNPVFVEQKKMLKTGLNIQQFKRHIPLIEEETREYFNRWGDSGEKNLFVALSELIILTASRCLHGK
EIRSMLDEEVAQLYADLDGGFTHLAWLAPSWIPFPSFLRRDRAHRKIKQIFYKAIAKRRETGVENDML
QTLIDSKYKSGRPLSDDEIAGMCIGLLLAGQHTSSTTSAWLGFFLARDKEVQDRCNAEQIKVCGDAST
EVSYDQLKDMQLLDHCVKEALRLRPPIMTMMRVAKSPLTYKDMTIPAGHQVCVSPTVNQRLKDNWMPG
PKEFNPDRFLDESKSNSEKFSYVPFGAGRHRCIGENFAYVQIKTIWSVMLRIFEFELVDGYFPGINYQ
TMIHTPLNPIIRYKRRTESL
>PmCYP51
MTIAGAGSSLVEGAIGRWQDTSTMTMVLVTTAFVLVVGAVFRKMLGGTQGKSDIKLPPSIPGIPFLGK
AVEFGENPIKFLEDSYKKYGEIFSFTMIGQTFTYLIGPESSAVLFNSKNEQLNAEEVYGRLTTPVFGK
GVAYDVPNAKFLEQKKILKMGLNIAQFRKHVPLVQDEANNYLKRWKEEKGQDNLFVAMSELIILTASR
CLHGKEIRSQLDEHVAQLYMDLDGGFSHLAWLFPSWIPFPSFRKRDRAHLEVKKIFYKAIQQRRACED
PDDDMLQTLITTTYKTGELLTDDEVAGMCIGLLLAGQHTSSTTSSWLGFFLAKDKDLQERCYQEQLRI
CGGDIDEPLTYDEVKEMTLLDRCLKETLRIRPPIMTMMRVAKEPVTFKDYVIPPGHQVCVSPTVNQRI
EDQWMPEPLNFNPDRFIDENASNSDKFSYVPFGAGRHRCIGENFAYVQIKTLMSVLIRKYELTLVDGY
FPTINYNTMIHTPYKPIIGYKLRK
>PpCYP51-hit-PPA_006648
MLVLMLLKYAVAVLLVAILAFCLAVAAYIHYLHMKFSHLPGPKRDSFLFGHQFVRKAILEKKKSFGEI
FLALHSEYGPVAVVFFLHFPFVAVADPKTVKEILLGSKYLKPAQDYEGFRALFGSRFMGRGLVSEVDH
SRWEFHRRIMNPAFHRKYLMELTDTFGESADRLVENLASKADGKSEIKVMQELERVTLDVICKVAFSM
EGDMINNDTPFTKAISVSLGAMLTSLSPLLHFDIRQKARAYRKEVRDSVKLLRDTGRDIITQRINDMK
HGKELPKDILSFVVKVAQAEENYSLEEMVDEFVTFFVAGQETTSNLLAFTLLSLGQNPHIMERLLEEV
DTVIGHKENIEYEDLGKLEYMSLVFKETLRLYPPVGGSTRVIPDEMDVVGFKMPAGTTLFVVSFAMCR
MEQYYKDPLTYNPERFKLNEDQTLYTYFPFSLGPRSCIGQQFAMIEAKIILSKLLQKLTFKLVPDQDL
GMYEQLTLKPSGHCANYITLREK
>AjCYP51-hit
MLFWLLLQYGMIVFAVVVGLLFTAAVCYLYYVHQKYDHLPGPKRDAFFQGHSSILKKVLAKEECFAEV
YAKLHREYGPLVVLFFYHMPFLSAVDPKVVKELLLNNKYPKPSLNYDAFRSLFGVPFMGNGLVSECNH
EKWAIHRRILNPAFHRQYLKELTGTFNESADRLVQYLSLKADGKTEVKLIHAFERVTLDVICKVAFSM
EGDMIQQESLFGDAIHLCLDSMYQAGSPIIKIDPRKKYRDYRRDVKKAARLLRGTGLKVINDHLEDVR
QGRELKKDILSHVIKSAVVDGNFTMEEMIDEFVTLFVGGYETTSTLLSFTMVCLGQNPHVLKKLLEEV
DSVIGDKEDITYDDIIKMEYMMLVLKETLRLYPPVIGSTRLLPEDADILGYKVPAGSSVSILSYVMAR
MEEFFPDPLTFDPDRFKDSDNRTMYAYFPFSMGARSCIGQQFAMIEARVILCKILQKLDFKLVPNQDF
GIYDQLTIRPSGDCANYISLRNK
>HumanLbr
MPSRKFADGEVVRGRWPGSSLYYEVEILSHDSTSQLYTVKYKDGTELELKENDIKPLTSFRQRKGGST
SSSPSRRRGSRSRSRSRSPGRPPKSARRSASASHQADIKEARREVEVKLTPLILKPFGNSISRYNGEP
EHIERNDAPHKNTQEKFSLSQESSYIATQYSLRPRREEVKLKEIDSKEEKYVAKELAVRTFEVTPIRA
KDLEFGGVPGVFLIMFGLPVFLFLLLLMCKQKDPSLLNFPPPLPALYELWETRVFGVYLLWFLIQVLF
YLLPIGKVVEGTPLIDGRRLKYRLNGFYAFILTSAVIGTSLFQGVEFHYVYSHFLQFALAATVFCVVL
SVYLYMRSLKAPRNDLSPASSGNAVYDFFIGRELNPRIGTFDLKYFCELRPGLIGWVVINLVMLLAEM
KIQDRAVPSLAMILVNSFQLLYVVDALWNEEALLTTMDIIHDGFGFMLAFGDLVWVPFIYSFQAFYLV

SHPNEVSWPMASLIIVLKLCGYVIFRGANSQKNAFRKNPSDPKLAHLKTIHTSTGKNLLVSGWWGFVR
HPNYLGDLIMALAWSLPCGFNHILPYFYIIYFTMLLVHREARDEYHCKKKYGVAWEKYCQRVPYRIFP
YIY
>SpLbr
MPSTTSFGDGDAVMSRWPGSTLWFKSKILRVSEDGYKVQFEDGTEEEIPLTDVKSESYFTRSRSRSRS
RSPGRRRSRSPARKSSPARTQQSRKPRSPGRKPAAVKKEVVETPVSRTQEKRTESKLISRVIGSLVFR
SPSKRARKTQTVTKTEHHSYTTRAQTRSGKQQLELPVKMKAGKVAKTTHYEFGGPIGTFLMIFGLPLV
VYFLYFTCLPSGCKLVYNPPFSLDWRDYYDQEAYLFYVGWFLFQAILALLPFGKVVQGQPLRSGQRLS
YRTNGLFALIVTCATFGGMIYYKCPVTLIVDKILPLMTASALFSLLLSTLLYIKARCGPNSALATGGN
SGNFFYDFFMGHELNPRLGSLDLKFFCELRPGLFLWALINMACLTKVWTEFPDNPPWNLILVCVFQFL
YVFDALLYESAILTTMDIIQDGFGFMLVFGDLTWVPFTYTLQARFLADHPPAFPDYCLIPVALLFSLG
YFIFRMSNSEKNAYRQNPYGKNVAGLQTIPTDTGKRLLVSGWWGFVRKPNYLGDLLMALSWSLCTGFV
SIVPYFYPIYFFVLLVHRERRDDASCRQKYGGAWTKYCATVKYRIIPYIY
>PmLbr
MPASHKYDVGEKVMVKWPGSALWYPAKVLAIQGEEYKVKFEEGTEDEVTADYIRTVGSFRRRSSSKSP
SRRRSRSRSPARSPSRQRVSRSPSRKSVAPKQQQQQQQQSSSKTATKSTTSTSTVVTQSKSMPKPDL
TPTRTPSKRILTSRSTTEVHSYTTRSSTKSGQQILEPMVEVKTGRVPKTNSYEFGGPIGAFFIMVSLP
LFIYYLYFTCSKTLCKLQLLPACTHDWRDYYDRDAYLIFLGWMVLQVFLFMLPVGSVVKGLPLRTGQR
LEYRLNGFFAFIVSLILFGGLVYKKYPVTVVYDKFLPLLTAAMIFSLLLSVFLYVKARKASGHALAPS
GNSGNVVYDFFMGHELNPRIGSFDFKFFCELRPGLIGWVLLDLAFVVKVWTDFPENPPWPLLLLTFFQ
LLYVADSLLYEHAILSTMDIIHDGFGFMLVFGDLVWVPFTYTLQARYLADNPSRSTMPNYCLIPILIL
GLIGYYIFRASNSQKNAFRENPYSSRFAASNILITASKKRLLMSGWWGWVRHPNYLVHREMRDSAHCK
QKYGTVWAKYCENVPYRIFPRVY
>PpLbr
MKGFNVGEQVRAKWPGSRLWYKAKILEELSDGFKVKFEDGTEDELDFSDVAHEARFKRRSRSRSKSPS
RRRSRSRSPARSPARTTQKKTPARSQTKKTVTQRVTVTKKPEETPAEPSQVKSLPRRLLTPTVTQTRT
QTKTTEEHRTYTTRSATRSGQQTLEPPTDIHQRSTVPKTKYYEFGGPIGVVLMMFLLPLVVYYLYFPC
HKGGCQFVLYPKCEGDWKKYFDVEAFLLVFGWIVFQVLIYFIPVGRIVQGLPLRSGKRLKYYVNAFYA
LVLSLLFFGLLVYYKMPFDVVIKKYLAVITTCMVLSIVMSIALYIKAKRAPSSELAPSGNSGNFFYDF
FMGHELNPRIDMLDLKYFCELRPGMIGWIIIDHIFLFSSWKDFPQNPPLPLLLLTIFHTLYVADALWF
ESAILSTMDIITEGFGFMLAFGDLVWVPFVFTVQARYLSMYPPEISNIGLGIVVVMNLFGYYIFRSAN
GEKNLLRRNPYSPDIAHLETISTSSGKKLLVSGWWGFVRRPNYLGDIIMAFSWALLCGFDSCIPYFYP
VYLTILLIHRERRDSHYCRQKYGRDWDIYCSRVPYRIIPYIY
>AjLbr
MEATGAPAWNMKGFNVGEQVRAKWPGSRLWYKATILEELSDGFKVKFEDGTEDELDFNDVAIEISFKV
TEQEAEPLAVAVASEVAGENHTEEDTGTTVTQTRTQTKSTEEHRTYTTRSATRSGQQTLEPPTDIHQR
STVPKTKYYEFGGPIGVVLMMFLLPLVVYYLYFACNKGKCQFDLYPTCEGDWKKYFDVEAFLLVIGWI
VFQVLIYFIPVGRIVQGLPLRSGKRLKYYVNAFYALVLSLVFFGLLVYYEMPFDVVIKKYLAVITTCM
VLSIVMSIALYIKAKRAPSSELAPSGNSGNFFYDFFMGHELNPRIGMLDLKYFCELRPGMIGWIIIDH
IFLFSAWKDFPQNPPLPLLLLTIFHTLYVADALWFEAAILSTMDIITEGFGSCWPLAI
>HumanDHCR24
MEPAVSLAVCALLFLLWVRLKGLEFVLIHQRWVFVCLFLLPLSLIFDIYYYVRAWVVFKLSSAPRLHE
QRVRDIQKQVREWKEQGSKTFMCTGRPGWLTVSLRVGKYKKTHKNIMINLMDILEVDTKKQIVRVEPL
VTMGQVTALLTSIGWTLPVLPELDDLTVGGLIMGTGIESSSHKYGLFQHICTAYELVLADGSFVRCTP
SENSDLFYAVPWSCGTLGFLVAAEIRIIPAKKYVKLRFEPVRGLEAICAKFTHESQRQENHFVEGLLY
SLDEAVIMTGVMTDEAEPSKLNSIGNYYKPWFFKHVENYLKTNREGLEYIPLRHYYHRHTRSIFWELQ
DIIPFGNNPIFRYLFGWMVPPKISLLKLTQGETLRKLYEQHHVVQDMLVPMKCLQQALHTFQNDIHVY
PIWLCPFILPSQPGLVHPKGNEAELYIDIGAYGEPRVKHFEARSCMRQLEKFVRSVHGFQMLYADCYM
NREEFWEMFDGSLYHKLREKLGCQDAFPEVYDKICKAARH
>SpDHCR24
MDTELSFNVLMSGLAALPVISVLFLIQFKGIEYVIVKQRWIFVCLFLLPLSAVYDVYYFMRNWVVFRM
SSAPKLHAERVQDIQSQVKKWKSDKSQQLMCTGRPGWQTISLRVGKYKLTHRKIFINLVDILDIDTER
QTMKVEPLATMGQITAMLNPLGWTLPVLPELDDLTVGGLIMGVGIESSSHKYGLFQHVCVSFELVLAD
GSVAQCSKDENPELFYSVPWSYGTLGFLVSAEIKIVPAKQYVRLEYKPVHCFDDVSNVFAKCSKEAQE
NEFVEGLMYSKDKAVIMTGQLTDQAEPAKINAIGNFWKPWFFKHVESFLKTGPAVEYIPLRHYYHRHS
RSIFWEIQDIVPFGNHPIFRYLLGWLTPPKVSLLKLTQGEIIRELYEKKHVVQDMLVPLKDMKSSLMC

FHDEMEMYPLWLCPFVLPALPGMVHPLGNQEELFVDIGAYGNPKNPNFHFRDSTRKVEEHVRKVNGFQ
MLYADSYMTREEFREMFDHSLYDKLRKNLKCEGAFPEVYDKINKSARH
>PmDHCR24
MSYELWATLCVAPIISLLLWIRVKGLEYTIVHQRWIFVCLFLLPLSVVYDAFSFVRNWIVFRMNTAPL
KHDERVKDIQRQVRQWKADGAKRPMCTARPGWQTVSLRVGKYKKTHHKININLVDILEVDTKRRVVRV
EPLVNMGQVTAMLNPMGWTLPVLPELDVLTDENPDLFYAVPWSYGTLGLLVAAEIQIVPAKKYVKVEY
KPAHSMDEVIKVFSEEITKKSGNEFVEALMYNKEEAVVMTANITDNAEESKVNSIGNFWKPWFFKHVQ
SYLRTGPGVEYIPLRQYYHRHTRSIFWELQDIVPFGNNPLFRYLFGWMVPPKISLLKLTQGETVRKLY
EQNHVVQDMLLYPLWLCPFLLPSVPGLVHPKGNKDEMYVDLGAYGTPKRKGFHFVETTRRVEEYVRSV
HGFQMLYADSHMTREEFRQMFDHTLYDNLRDKLGCKKAFPEIYDKISKAARI
>PpDHCR24
MLWLSLGTGLLSSFILFSYVKGTEHMMKHYRWVFVCFGLLPISLVYSAVFNIRNWIVFKLKSAPKNHK
SKVEQVCKQIKDWRASGSKQKLCTSRASWESHSLREGKYKKTDRKINLNLVDILEINTEKGYVRAEAM
ATIGELTATLIPMGWTVPVIPELDDCCVGGLVMGSGVESTSHKYGLFQHICKSFEIVLATGEVKRCSK
DENSDLFYSVPWSCGTLGFLVSADILIVPSKKYVRLEYYPAKTRQEVVRVFKEQTLRTEGNEFVEGLM
YNENEAVIMTGSQTDDAEPDKINNIGNFYKPWFYKHVEGILKTGSCVEYIPLRDYYHRHSRSLFWEMQ
DIIPFGNHPIFRYLFGWLLPIKISLLKLTQTKTLRELYKKRHIVQDMLVPLDDFEDALKCFHQEIKMY
PLWICPFKLPANPGMVHPHGNQEELYVDIGAYGNPGTANFHYSDTVRRIEKFVRDRHGFQMLYADCFM
TREEFRVMFDHSLYDKVRKDLQCEKAFPEVYEKICQSARY
>AjDHCR241
MSPLPQPRPPPRLYHSRRLVHAWAVYRLIGRLNDCQQGPGRIYKHSFAPNKIAKMLWLSLGTGLLSSF
MLFSYVKGTEHMMKHYRWVFVCFGLLPISLVYSAVFNIRNWIVFKLKSAPKNHKSKVEQVSKQIKDWR
ASGSKQKLCTSRASWESHSLREGKYKKTDRKINLNLVDILEINTEKGYVRAEAMATIGELTATLIPMG
WTVPVIPELDDCCVGGLVMGSGVESTSHKYGLFQHICKSFEIVLATGEVKRCSKDENSDLFYSVPWSC
GTLGFLVSADILIVPSKKYVRLEYYPAKTREEVVRVFKEQTLRTEGNEFVEGLMYNENEAVIMTGSQT
DDAEPDKINNIGNFYKPWFYKHVEGILKTGFCVEYIPLRDYYHRHSRSIFWEMQDIIPFGNNVFFRYL
FGWLVPPKISLLKLTQTDTLRELYRKHHLVQDIFVPLDKMEDTLKFIHKEMKMYPLWLCPFVLPNQPG
MFHPPGDEDGLYIDIGAFGTPKAENYSALKTIRKLEEYEREVNGFQMLYADCHMTREEFRDMFDHTLY
DKLRKELKCEDAFPEVYDKISKDARY
>AjDHCR242
MLWLSLGTGLLSSFILFSYVKGTEHMMKHYRWIFVCLGLLPISLVYSAVLYLRNLLVFKLKSAPQRHQ
WRVDGVSQQIEDWIKSGSQKKLCTARPGWQTTSLRVGLYKKTHRNIHINMVDILDVDTDGRIVRCEPL
VTMGQITALLSPMGWSLPVLPELDDLTVGNELLSNFIFVCKMGKFKVECNGVGIESSSHKHGLFQHIC
EAFEVVLGNGDVVKCSKEENADLFHSIPWSYGTLCFLVSVDIRIVPIQSYVRLEYRPTKTRQQTVQVF
KEQTCKEDGNDFVEGLMFGENEAVVMTGTLTEDLEADKINKIGYFYKPWFYKHVEGIMKTGYCVEYIP
LRDYYHRHSRSIFWEMQDIIPFGNHPIFRYLFGWLLPIKISLLKLTQTKTLRELYKKRHIVQDMLVPL
DDFEDALKCFHQEIKMYPLWICPFKLPANPGMVHPHSNQEELYVDIGAYGNPGTADFHYSDTVRRIEK
FVRDRHGFQMLYADCFMTREEFRVMFDHSLYDKVRKDLQCEDAFPEVYDKICQSARY
>HumanC4mso
MATNESVSIFSSASLAVEYVDSLLPENPLQEPFKNAWNYMLNNYTKFQIATWGSLIVHEALYFLFCLP
GFLFQFIPYMKKYKIQKDKPETWENQWKCFKVLLFNHFCIQLPLICGTYYFTEYFNIPYDWERMPRWY
FLLARCFGCAVIEDTWHYFLHRLLHHKRIYKYIHKVHHEFQAPFGMEAEYAHPLETLILGTGFFIGIV
LLCDHVILLWAWVTIRLLETIDVHSGYDIPLNPLNLIPFYAGSRHHDFHHMNFIGNYASTFTWWDRIF
GTDSQYNAYNEKRKKFEKKTE
>SpSC4MOL
MAAESVLNDLPIVENATDGGMGAFEEGWRYMNSNYSRFQISTYMSACLHIVTYFLFCSPSFIFQFFRF
MDRYKIQQDKPTTWDQEWKCFKLVIANQVLIQTPFFSGAYFFCQYMNIPFDYESMPVWYMTLAHCFGS
LVLEDAWHYFLHRALHHKSIYKYIHKIHHNFQAPFGMTAEYAHPMETMILGMGFMWGMLLFCDHLIFL
WCWMCVRLIETIDVHSGYDFPINPLHVIPFYGGARFHDFHHKNFNGNYSSTFTWWDKIFGTDMQYKDY
YAKLQDQKTEKKAN
>PmSC4MOL-GAWB01029932.1
MGEEGIIGTMRLSEFWDYVPGNPLKPPVEMAWTYMTDNYTRFQIATLGSVLVHMLSYYLISTPSILCQ
FLPFMQRFKIQNDKPETAKNQWKCVKLLLMSQLFIQLPIICASYLYCEMFSIPFEWDKMPAWYVLLAQ
FYCCLVIEDTWHYFLHRALHDKRIYKYIHKVHHNFQAPFGMVAEYAHPAETMILGMGTMWGILLMGNH
LAFLWGWMVVRLLETIDVHSGYDIPMSPLHLLPFYGGARFHDFHHMNFNGNYSSTFTWWDKIFGTDRQ
YKDYIKEKTDKKD

>PpSC4MOL
MENFWFSSNVTETVIGASYYLPNNPLQSHMEDGWNYMNNHFSRFQIACWGSLLVHEVAFVLLNLPLFV
AQFVPFLQRYKVQTDKPESYVDQWKALKLIMFSHVFIDTPFTCGTYVFTEFFNIPFSWETMPCWYSIV
GYIFLCLVVEDTWHYFLHRIMHDKRFYKHCHKIHHTFQAPFSIAAEYAHPLETFILGMGTMWGILFFA
NHLVLIWAWAVARMLESYDVHSGYEFPFNPLHLIPFYAGTRFHDFHHKNFHGNYSSTFTWWDKLFGTD
VQYKQFIEKQQVDKEK
>AjSC4MOL
MWGILFFANHLVLIWAWAVARMLESYDVHSGYEFPFNPLHLIPFYAGTRFHDFHHKNFHGNYSSTFTW
WDKLFGTDVQYKQFIEKQQVDKEK
>HumanFXDC2
MKGEAGHMLHNEKSKQEGHIWGSMRRTAFILGSGLLSFVAFWNSVTWHLQRFWGASGYFWQAQWERLL
TTFEGKEWILFFIGAIQVPCLFFWSFNGLLLVVDTTGKPNFISRYRIQVGKNEPVDPVKLRQSIRTVL
FNQCMISFPMVVFLYPFLKWWRDPCRRELPTFHWFLLELAIFTLIEEVLFYYSHRLLHHPTFYKKIHK
KHHEWTAPIGVISLYAHPIEHAVSNMLPVIVGPLVMGSHLSSITMWFSLALIITTISHCGYHLPFLPS
PEFHDYHHLKFNQCYGVLGVLDHLHGTDTMFKQTKAYERHVLLLGFTPLSESIPDSPKRME
>SpFXDC2
MGDNESRDETCCYSGKASLEKEAAIDIKDAEQEQKSRLSRFCEVFAKIALIVGGFIIVLGALRNTLTY
YLQLFWGVSGNFWMRQWGKVYDVFEGDILNISISNDNVLFFLLNIRSNRLNYVGAHHCPVLASVCIFP
DSGSCEAIIHHQVQDTTNPRMTLIPEGKLRKAVLTVLANQTVVVFPIAVGVYHLMGWRGCGFSAAELP
SFQWVMLELFVFLIVEEFGFYYSHRLSHHPRLYKYVHKKHHEWTAPISVVAIYAHPIEHIFSNTLPVV
LGPLIMGSHIATLTMWAMLAQASAINSHCGYHLPLMPSPEAHDFHHLKFTNNFGTLGFLDRLHGTDEL
FRKTKPYHRHFLLLGLTPVSQTFPDDSRCKNIECDKDE
>PmFXDC2
MAQMELEIRDKDNPENQDKGCPKNVVNQQQEKKCSHDYQWTETLSILLFVIGGIILSLATIWNSLTWH
LHWFWGASGNFWDEQWFKLHTLFGGNTFMLGVVCTQVITFVMYLVVNCVFVYVDVTGGPAFIVKYKIQ
QDQNVPVDWERLKKDFRSVLFNITVVNFVFLVCMYPMGLWCGVNADPNLPSFHRVLAEFVVYLIFEEI
GFYYFHRLLHHPRLYKHFHKQHHEWTAPIGILTLYAHPVEHVLANLLPTVIGPFLMASHVAVVWVWLV
IAQTSAVISHCGYHLPLLPSPEAHDFHHLKFVNCFGALGVLDRLHGTDAIFRQAKQYSRHCVLLGTTP
LSQTYPDGTKGQKVIKEGE
>PpFXDC2
MTNWNELTGIFSGIWRRQFGIKSQVSKKCLESCPPGPSGHAQQTLLVEDESLLKRFSLQQWLEVLRKV
TYAIGGVVVFAAFRNSLTWHLEKFWGASGDFWQSHWGKIYTFFGEDDFTVGFYGTAWVSFLSYWFVCL
LFLIIDLTGQPAFIFKYKIQDDQNIPVPRGKLLHAIAVVLYNQVVVGGPTMLLLYHVMQWRGCSFGRE
LPSIYWVMVEAVVFLIFEEIGFYYSHRVFHHPRVYRYIHKIHHEWTAPISVVAIYCHPLEHLFANILP
AVIGPVVMGSHIATLMLWINVAQTSAIISHCGYHLPFLPSPEAHDFHHLKFTNNFGTLGILDRLHGTD
SLFRQSRQYQRHVLLLGLTPVKQAFPDDPKEQKCD
