# Supplementary material for: Biosynthesis of saponin defensive compounds in sea cucumbers
Source: Nat Chem Biol. 2022 Jun 27;18(7):774–81. doi: 10.1038/s41589-022-01054-y (PMC9236903; doi:10.1038/s41589-022-01054-y)
Supplement: Supplementary file 2 — Reporting Summary [file 41589_2022_1054_MOESM2_ESM.pdf]

nature research

Corresponding author(s): Ramesha Thimmappa and Anne Osbourn

Last updated by author(s): Apr 9, 2022

Reporting Summary

Nature Research wishes to improve the reproducibility of the work that we publish. This form provides structure for consistency and transparency in reporting. For further information on Nature Research policies, see our Editorial Policies and the Editorial Policy Checklist.

Statistics

For all statistical analyses, confirm that the following items are present in the figure legend, table legend, main text, or Methods section.

n/a	Confirmed	
☐	☒	The exact sample size (*n*) for each experimental group/condition, given as a discrete number and unit of measurement
☐	☒	A statement on whether measurements were taken from distinct samples or whether the same sample was measured repeatedly
☒	☐	The statistical test(s) used AND whether they are one- or two-sided<br>*Only common tests should be described solely by name; describe more complex techniques in the Methods section.*
☒	☐	A description of all covariates tested
☒	☐	A description of any assumptions or corrections, such as tests of normality and adjustment for multiple comparisons
☐	☒	A full description of the statistical parameters including central tendency (e.g. means) or other basic estimates (e.g. regression coefficient) AND variation (e.g. standard deviation) or associated estimates of uncertainty (e.g. confidence intervals)
☒	☐	For null hypothesis testing, the test statistic (e.g. *F*, *t*, *r*) with confidence intervals, effect sizes, degrees of freedom and *P* value noted<br>*Give P values as exact values whenever suitable.*
☒	☐	For Bayesian analysis, information on the choice of priors and Markov chain Monte Carlo settings
☒	☐	For hierarchical and complex designs, identification of the appropriate level for tests and full reporting of outcomes
☒	☐	Estimates of effect sizes (e.g. Cohen's *d*, Pearson's *r*), indicating how they were calculated

Our web collection on statistics for biologists contains articles on many of the points above.

Software and code

Policy information about availability of computer code

Data collection	No customised aglorithms or software were used .
Data analysis	ContigExpress tool in Vector NTI (v11.5.3); FGENESH; MEGA7 (10.2.6); CLC Genomics Workbench v9.5.3; GC-MS MassHunter Chemstation (Agilent) (B.07.00); HTSeq (ver 0.6.1); ClustalW (ver2.1); Phyre2 (ver2.0); I-TASSER (ver5.0); Chimera (ver 1.15); PyMOL (version 2.0); STAR aligner (ver2.1.3); Leica DFC420 C camera on a Leica DMI4000B; Thermo Scientific™ Xcalibur software (ver4.3.73.11); NISTv8 library; LC-MS LabSolutions (ver3.0) (Shimadzu).

For manuscripts utilizing custom algorithms or software that are central to the research but not yet described in published literature, software must be made available to editors and reviewers. We strongly encourage code deposition in a community repository (e.g. GitHub). See the Nature Research guidelines for submitting code & software for further information.

Data

Policy information about availability of data

All manuscripts must include a data availability statement. This statement should provide the following information, where applicable:

- Accession codes, unique identifiers, or web links for publicly available datasets
- A list of figures that have associated raw data
- A description of any restrictions on data availability

Data supporting the findings of this work are available within the paper and its Supplementary Information files. A reporting summary for this article is available as a supplementary Information file. The datasets, constructs and chemical standards generated and analyzed during the current study are available from the corresponding author upon request. Sea cucumber material is available from co-authors Veronica Hinman (Carnegie Mellon University, Pittsburgh, US; email: veronica@cmu.edu) and Shi Wang (Ocean University of China, Qingdao, China; email: swang@ouc.edu.cn). All associated raw data is provided in data files S1 to S7. The sequences of genes characterized within this project will be deposited in the European Nucleotide Archive and accession numbers shared before publication.

Field-specific reporting

Please select the one below that is the best fit for your research. If you are not sure, read the appropriate sections before making your selection.

☒ Life sciences ☐ Behavioural & social sciences ☐ Ecological, evolutionary & environmental sciences

For a reference copy of the document with all sections, see nature.com/documents/nr-reporting-summary-flat.pdf

Life sciences study design

All studies must disclose on these points even when the disclosure is negative.

Sample size	No sample size calculation was performed and it was not required in our study. Details on biological replicates or technical replicates are given in the manuscript.
Data exclusions	No data were excluded.
Replication	Details of biological or technical replicates are given in figure legends, methods and supplementary information sections of the manuscript. Raw data is given in source data files.
Randomization	Not applicable in our study.
Blinding	Blinding was not relevant in our study.

Reporting for specific materials, systems and methods

We require information from authors about some types of materials, experimental systems and methods used in many studies. Here, indicate whether each material, system or method listed is relevant to your study. If you are not sure if a list item applies to your research, read the appropriate section before selecting a response.

Materials & experimental systems

n/a	Involved in the study	
☐	☒	Antibodies
☒	☐	Eukaryotic cell lines
☒	☐	Palaeontology and archaeology
☒	☐	Animals and other organisms
☒	☐	Human research participants
☒	☐	Clinical data
☒	☐	Dual use research of concern

Methods

n/a	Involved in the study	
☒	☐	ChIP-seq
☒	☐	Flow cytometry
☒	☐	MRI-based neuroimaging

Antibodies

Antibodies used	anti-DIG alkaline phosphatase-conjugated antibody (Roche) was used in whole mount in situ hybridization (WMISH) experiments.
Validation	Anti-DIG alkaline phosphatase-conjugated antibody (Roche) was used in sea cucumber whole mount in situ hybridization (WMISH) experiments in the manuscript.
